# Group-level cortical functional connectivity patterns using fNIRS: assessing the effect of bilingualism in young infants

**DOI:** 10.1117/1.NPh.8.2.025011

**Published:** 2021-06-12

**Authors:** Borja Blanco, Monika Molnar, Manuel Carreiras, Liam H. Collins-Jones, Ernesto Vidal, Robert J. Cooper, César Caballero-Gaudes

**Affiliations:** a Basque Center on Cognition, Brain, and Language, Donostia/San Sebastián, Spain; b University College London, Biomedical Optics Research Laboratory, DOT-HUB, London, United Kingdom; c University of Toronto, Faculty of Medicine, Department of Speech-Language Pathology, Toronto, Ontario, Canada; d Ikerbasque, Basque Foundation for Science, Bilbao, Spain

**Keywords:** functional connectivity, functional near-infrared spectroscopy, bilingualism, resting-state, language acquisition, connectome

## Abstract

**Significance:** Early monolingual versus bilingual experience induces adaptations in the development of linguistic and cognitive processes, and it modulates functional activation patterns during the first months of life. Resting-state functional connectivity (RSFC) is a convenient approach to study the functional organization of the infant brain. RSFC can be measured in infants during natural sleep, and it allows to simultaneously investigate various functional systems. Adaptations have been observed in RSFC due to a lifelong bilingual experience. Investigating whether bilingualism-induced adaptations in RSFC begin to emerge early in development has important implications for our understanding of how the infant brain’s organization can be shaped by early environmental factors.

**Aims:** We attempt to describe RSFC using functional near-infrared spectroscopy (fNIRS) and to examine whether it adapts to early monolingual versus bilingual environments. We also present an fNIRS data preprocessing and analysis pipeline that can be used to reliably characterize RSFC in development and to reduce false positives and flawed results interpretations.

**Methods:** We measured spontaneous hemodynamic brain activity in a large cohort (N=99) of 4-month-old monolingual and bilingual infants using fNIRS. We implemented group-level approaches based on independent component analysis to examine RSFC, while providing proper control for physiological confounds and multiple comparisons.

**Results:** At the group level, we describe the functional organization of the 4-month-old infant brain in large-scale cortical networks. Unbiased group-level comparisons revealed no differences in RSFC between monolingual and bilingual infants at this age.

**Conclusions:** High-quality fNIRS data provide a means to reliably describe RSFC patterns in the infant brain. The proposed group-level RSFC analyses allow to assess differences in RSFC across experimental conditions. An effect of early bilingual experience in RSFC was not observed, suggesting that adaptations might only emerge during explicit linguistic tasks, or at a later point in development.

## 1 Introduction

Language acquisition begins about 3 months prior to birth when infants are able to hear spoken language.^1^ In a bilingual learning environment, infants are exposed to the linguistic statistical regularities (e.g., speech sounds, words, and grammar) of not one but two linguistic inputs. Therefore, differently from monolinguals, bilinguals need to discriminate between two languages. Although bilingual infants’ overall linguistic exposure should be comparable to that of monolinguals, bilingual infants likely receive less exposure to each of their languages compared to their monolingual counterparts, as their exposure time is divided between two linguistic inputs.

The potential complexity of the bilingual input has consequences on infants’ behavior and brain responses. These consequences have been conceptualized as dissimilar adaptation patterns to monolingual versus bilingual environments. From the perspective of bilingual infants, functional adaptations across multiple cognitive domains might facilitate the acquisition of two languages as opposed to one. For example, at the behavioral level, bilingualism has an impact when attention allocation to languages is considered: bilingual 4-month-olds orient slower to their native languages,^2^^,^^3^ and they exhibit longer sustained attention periods than monolinguals when processing spoken language.^4^ It has also been proposed that bilingual infants possess increased executive control abilities^5^^,^^6^ (but see also Ref. 7). Neuroimaging studies have demonstrated dissimilar patterns of brain activation in monolingual and bilingual infants in a language discrimination/recognition task^8^ and in early phonetic processing.^9^ In a cohort of 4- to 8-month-old infants, Mercure et al.^10^ showed that brain activation responses to spoken and signed language showed no lateralization effects in monolingual and bimodal bilingual infants. However, unimodal bilingual infants’ brain responses were right lateralized over posterior temporal regions for both conditions. Differential neural responses have also been reported in older monolingual and bilingual infants when their sensitivity to native and non-native speech sounds^11^^,^^12^ or words^13^ were assessed. Despite the differences described above, language acquisition trajectories are not fundamentally different between monolingual and bilingual infants,^14^^–^^16^ suggesting that specific cognitive and/or functional adaptations that take place during the bilingual learning process might help these infants compensate the increased complexity of a bilingual environment.

In this work, we aim to assess whether growing up in a bilingual environment (i.e., simultaneously acquiring two languages from birth) might shape the intrinsic functional organization of the infant brain. To answer this question, we measured resting-state functional connectivity (RSFC) in 4-month-old infants using functional near-infrared spectroscopy (fNIRS).^17^^,^^18^ RSFC can be defined as synchronized cerebral activity between brain regions that share a common role in supporting functionally relevant sensory and cognitive processes.^19^^,^^20^ RSFC can be measured in infants, children, and adults, providing a window into neural specialization across the life span. The intrinsic functional organization of the infant brain described by RSFC can be modulated by various pre- and postnatal factors.^21^^–^^23^ As measured by functional magnetic resonance imaging (fMRI), premature and full-term infants show different RSFC patterns.^24^^,^^25^ It has been suggested that the configuration and maturational course of functional connectivity differs in typical and atypical functional brain development.^26^^,^^27^ In addition, it has been proposed that early environmental factors can modify RSFC, including caregivers’ education level, or socioeconomic status.^28^ Based on these observations, the present study aims to assess whether the brain’s functional connectivity begins to adapt to a bilingual environment as early as 4 months of age, by the time neural and behavioral responses to external stimuli already differ across monolingual and bilingual infants. MRI studies in adults suggest that a long-term exposure to two languages might alter the brain’s functional^29^^–^^31^ and structural connectivity.^32^^,^^33^ Stronger functional connectivity in bilingual adults, as compared to monolinguals, has been observed in long-range bilateral and anterior–posterior connections on both hemispheres^29^ and in brain networks associated with language and executive control processes.^30^^,^^31^ Studying RSFC in monolingual and bilingual infants can elucidate the extent to which a long-term environmental factor such as early bilingual experience might lead to specific adaptations in the intrinsic properties of different functional brain systems, while avoiding potential confounds due to task interference. Based on previous task-based studies with similar age groups^8^^–^^10^ and considering the spatial resolution and coverage of our fNIRS setup, differences in functional connectivity are expected to emerge over brain regions overlapping the auditory and the language networks with bilinguals showing stronger interhemispheric connectivity in these networks. Tentatively, bilinguals might also show a stronger functional connectivity in networks involving frontal regions.

When attempting to describe RSFC at the group level and to quantitatively compare RSFC patterns across experimental groups, traditional fNIRS-RSFC data analysis methods such as seed-based correlation analysis,^34^^–^^37^ independent component analysis (ICA),^38^^,^^39^ or clustering methods^18^^,^^40^ present some limitations. For example, group-level functional connectivity studies based on ICA have often been computed by averaging subject-specific independent components (ICs) that match *a priori* spatial configurations (e.g., bilateral and covering sensorimotor regions) or exhibit high similarity across subjects. However, individual data are usually affected by noise components of different characteristics which might result in an ICA separation that differs across subjects, making it difficult to match components. The ultimate consequence of these limitations is that previous fNIRS-RSFC studies have evaluated group differences using qualitative comparisons^18^^,^^40^ or performing statistical analysis on specific connectivity indices only.^41^

Hence, an additional goal of this work is to overcome the aforementioned limitations by implementing two data-driven methodologies based on ICA to extract group-level large-scale functional connectivity patterns from fNIRS data. First, we used temporal group ICA (tGICA) with dual regression to compute temporally independent patterns of spontaneous hemodynamic activity.^42^^,^^43^ By decomposing the concatenated fNIRS channel time series of multiple subjects, tGICA generates a set of group-level maximally independent temporal time courses and its common aggregated spatial maps [i.e., functional networks (FN)], which quantify the presence of each particular IC on each channel. Group-level spatial maps, which spatially represent the FN of interest, can be regressed out to the subject level to obtain subject-specific spatial maps using spatiotemporal or dual regression, in which statistical analyses can be performed to assess group differences. Second, we implemented an fNIRS-tailored version of the connectome-based ICA (connICA) method.^44^ In the connICA method, the individual functional connectivity matrices or connectomes are jointly decomposed to obtain latent group-level independent functional connectome components (FCCs) and its associated weights, which quantify the relative prominence of the FCC on each subject. These values can also be used to perform statistical comparisons between groups. The two proposed ICA-based analyses provide complementary information to study RSFC. tGICA searches for independence between signal subcomponents based on their temporal dynamics, resulting in temporally independent patterns of hemodynamic brain activity. In contrast, connICA relies on a precomputed similarity index (e.g., Pearson correlation) to perform this separation, and the extracted components represent independent whole-cortex functional connectomes. In addition to describing cortical network organization at the group level, the two methods allow a quantitative investigation of the link between particular experimental variables (i.e., early bilingualism in the current work) and the relative presence of the extracted functional connectivity patterns on each experimental group under assessment. Here the presence of the identified FN and FCC was quantified in each participant, and results were compared across two monolingual groups of infants and one bilingual group of infants by means of conventional frequentist and Bayesian statistical frameworks.^45^ Finally, we present a framework to determine the optimal number of components (i.e., ICA model order) considering their computational consistency, the neurophysiological properties of the fNIRS signal, and the amount of data variance explained.

## 2 Methods

### 2.1 Participants

One hundred and twenty-three healthy, full-term infants participated in this study. Sixteen of these participants were not tested as these infants were unable to fall asleep. The data of one participant were discarded for receiving a regular exposure to English. Two infants were excluded before data preprocessing because their datasets were shorter than 600 s. Five infants (n=2 Basque–Spanish bilingual infants, n=1 Spanish monolingual infant, and n=2 Basque monolingual infants) were excluded during data preprocessing due to insufficient data quality. In the final sample, for which data were analyzed and results are presented, 99 participants were included: 36 Basque–Spanish bilingual (BIL) infants (21 girls; mean age=125±4 days), 30 Spanish (SP) monolingual infants (13 girls; mean age=123±3 days), and 33 Basque (BQ) monolingual infants (17 girls; mean age=122±4 days). Participants’ language background was assessed with a questionnaire filled by the parents, in which infants’ percentage of exposure to each language (SP and BQ) during the first months of life was measured. Participants exposed to a single language (SP or BQ), or <10% of the time to a second language (SP or BQ), were included in each of the monolingual groups. Infants exposed to two native languages (Spanish–Basque) from birth formed the bilingual group. Participants were recruited from the same region of the Basque Country (Gipuzkoa); a socioeconomic status questionnaire was completed to ensure similar levels of education, parental occupation, and household income across groups. Parents were informed about the procedure of the study and signed a written informed consent before starting the experiment. This study was approved by the local ethical committee.

### 2.2 Data Acquisition

**Fig. 1:**
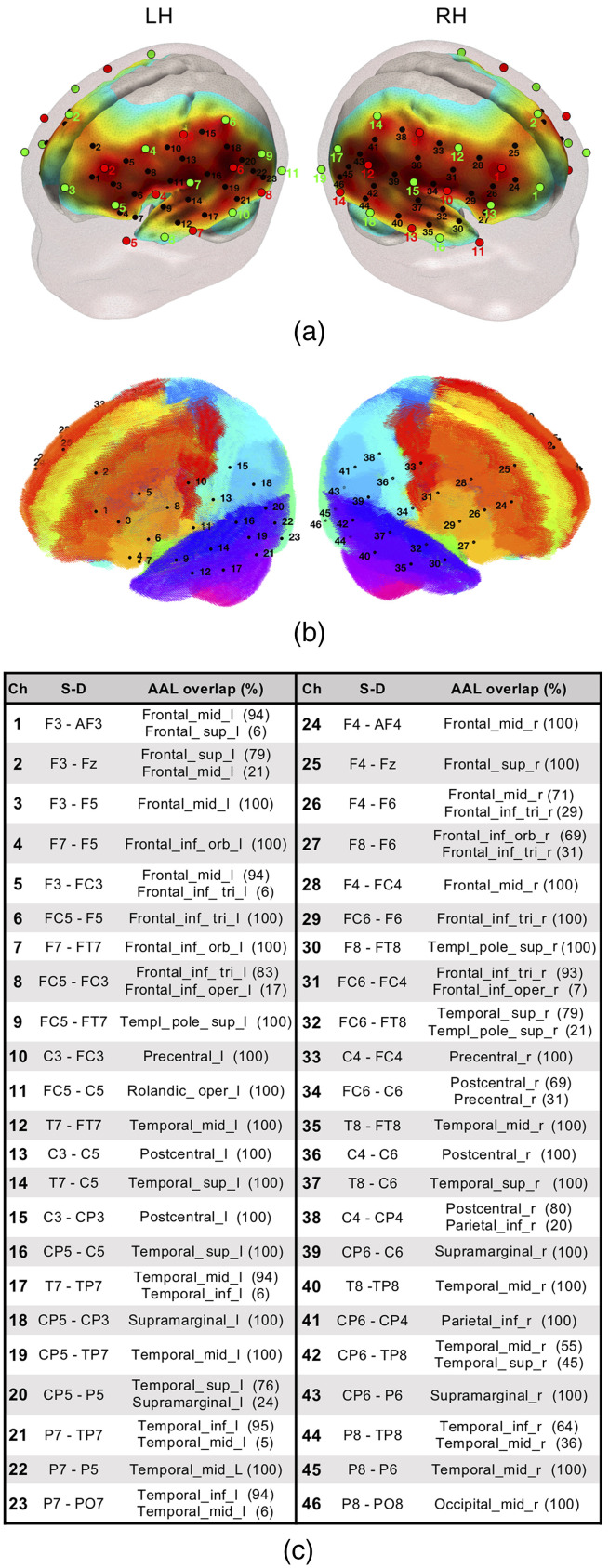
(a) fNIRS optode (sources in red and detectors in green) and channel (black) localization in the current experimental setup. The normalized sensitivity profile of this configuration is displayed in a 6-month-old infant head model. (b) Localization of the fNIRS channels in our setup registered to a 6-month-old infant AAL template. (c) Table depicting source–detector distances and the brain labels of our setup based on the on the probabilistic spatial registration of the fNIRS channels to a 6-month-old infant AAL template. Ch, channel and S–D, source–detector pair.

fNIRS measurements were performed with a NIRScout system (NIRx Medical Technologies, CA, USA) at wavelengths 760 and 850 nm with a sampling frequency of 8.93 Hz. Sixteen light emitters and 24 detectors were positioned on a stretchy fabric cap (Easycap GmbH, Germany) over frontal, temporal, parietal, and occipital regions of both hemispheres according to the international 10–20 system (Fig. 1). Each pair of an adjacent light emitter and a detector formed a single measurement channel, generating 52 channels for each hemoglobin oxygenation state (i.e., oxyhemoglobin HbO and deoxyhemoglobin HbR). This configuration yielded source–detector separation distances ranging from ∼20 to ∼45  mm (Fig. 1). Nasion, inion, and preauricular points were used as external head landmarks, and caps of two different sizes were employed to adapt to individual head circumference size (i.e., 40 and 42). This approach ensured a consistent cap and optode positioning across infants (i.e., without additional MR images or external coordinate tracking system), so that channels corresponded to comparable anatomical locations. Occipital channels were discarded in all participants for being particularly prone to contain signal artifacts. During data acquisition, the back part of the infants’ head was leaning against the parent’s body and any minor movement resulted in the misplacement of these particular optodes. Data from the remaining 14 sources and 19 detectors (i.e., 46 channels) were analyzed.

The sensitivity profile of the fNIRS probe setup was computed to provide information of the brain areas under investigation. The probe setup was registered to an average 6-month-old infant template^46^ to compute the sensitivity matrix of our source–detector configuration using Toast++.^47^ We obtained the aggregated sensitivity profile of our probe by summing the normalized cortical sensitivity profiles of each individual channel (Fig. 1). Channel positions were defined as the gray matter node which coordinates were closest to the central point of the maximum sensitivity path along each source–detector pair. A 6-month-old average atlas was used to compute a probabilistic spatial registration of the cortical structures underlying each channel.^48^ Channel coordinates were first transformed to the average^48^ T1 template space using advanced normalization tools,^49^ and then registered into the anatomic atlas,^48^ defined by 116 cortical regions based on automated anatomical labeling (AAL).^50^ For each channel, the AAL anatomical labels within a distance of 20 mm were defined, and the percentage of overlap with each AAL region was calculated (see Fig. 1).

### 2.3 Experimental Procedure

We measured infants’ spontaneous hemodynamic activity during natural sleep while leaning on their parents’ lap in a sound-attenuated room. The only source of illumination in the room was the screen of the recording computer, which was attenuated to low brightness levels. Recordings started when infants were relaxed, accustomed to the fNIRS cap, and clear signs of sleep were noticeable by the experimenters and the parents. Over the duration of the recording, parents were asked to remain silent and minimize movements in order to avoid involuntary cap or optode displacement. Recordings of 10 to 25 min were acquired in order to maximize the duration of continuous, motion-free periods.

### 2.4 Data Preprocessing and Quality Assessment

Data preprocessing and analyses were performed in MATLAB (R2012b and R2014b, MathWorks, MA, USA) using in-house scripts as well as third-party toolboxes and algorithms. Optical density changes were calculated from raw light intensity data by computing the negative logarithm of the ratio between detected light intensity at each time point and a reference baseline value (e.g., the mean signal).^51^ Noisy periods at the beginning or/and at the end of the recordings, presumably matching awake activity of the infants (i.e., before the infant was completely asleep and after the infant woke up) were visually identified and removed. As participants were asleep during the acquisition, fNIRS measurements displayed high data quality. Some datasets showed brief, sparse motion-induced artifacts characterized by abrupt amplitude signal changes and/or artifactual signal drifts. These artifacts were corrected using the wavelet-based method described by Patel et al.,^52^ which was adapted for fNIRS data.^53^ Briefly in this method, the time series of individual channels are decomposed in the wavelet domain. Wavelet coefficients existing simultaneously across multiple frequencies characterize non-stationary signal changes caused by low- and high-frequency artifacts. Hence, wavelet coefficients identified as series of local maxima and minima are nulled (i.e., set to zero) to recompose the denoised signal. Optical density data were then converted into HbO and HbR concentration changes by means of the modified Beer–Lambert Law with differential pathlength factors of 5.3 and 4.2.^54^ After this step, all datasets were limited to 5000 samples (i.e., ∼560  s) to ensure homogeneous effect estimate precision in the first level of the analysis (i.e., robust Pearson correlation coefficient). This step was performed by visually identifying the segment of the dataset displaying the best data quality. Temporal filtering and global signal regression were performed simultaneously in a unique nuisance regression model.^55^ Specifically, contributions of high-frequency physiological noise sources (e.g., respiration and cardiac pulsation) were accounted for by including Fourier terms for frequencies above 0.09 Hz in the model. Slow frequency fluctuations and signal drifts were modeled by adding the first four-order Legendre polynomials to the model, for which the spectral power predominantly consists of very low frequencies (0 to 0.004 Hz).^56^ The average fNIRS signal across all channels was also included in the regression model to remove globally occurring hemodynamic processes in cerebral and extracerebral tissues assumed to largely reflect systemic hemodynamic changes.^57^^,^^58^ As HbO and HbR are differently affected by global systemic processes, data of each hemoglobin chromophore were filtered independently by including either the global HbO or HbR signal in the model. Additional information about the codes and parameters used in our preprocessing pipeline are included in the Supplementary Material. The fNIRS datasets employed in this study have been made publicly available and can be found in Ref. 59.

Data quality was evaluated in each participant at each preprocessing step (see the Supplementary Material). We inspected channel time series (e.g., intensity, optical density, and concentration) to detect motion-induced artifacts and signal drifts in the raw data and after wavelet despiking. We assessed the presence of physiological components, such as respiration and cardiac pulsation, in the power spectral density of HbO and HbR prior to temporal filtering. We evaluated the statistical association between time series fluctuations of Hb chromophores (i.e., HbO and HbR) which is expected to be characterized by a strong negative correlation ^60^^,^^61^ and an antiphase state.^62^ These properties describing the intrinsic relationship between HbO and HbR hemodynamic fluctuations have been confirmed in the previous task-based^63^ and resting-state fNIRS studies in infants and adults.^62^^,^^64^ Algorithms that maximize the negative correlation between Hb chromophores have also been proposed as a signal improvement or noise reduction method.^65^ A negative correlation between HbO and HbR signals (i.e., an antiphase state) was considered as a valid indicator of data quality. As part of our data quality assessment routine during preprocessing, we also replicated the results of two previous fNIRS RSFC studies with infants. We replicated the work by Watanabe et al.^62^ showing the expected antiphase state between HbO and HbR signals in each of our three experimental groups. Following the work by Homae et al.,^18^ we performed a hierarchical clustering analysis to spatially group channels based on the degree of similarity between their time series and measured as pairwise temporal correlation. This analysis was computed for HbO and HbR, and we obtained similar spatial clusters as in the original study, with frontal, temporal, and parietal channels of each hemisphere clustering together. Quality assurance figures for each participant and group-level replication analyses are given in the Supplementary Material.

### 2.5 Functional Connectivity Analyses

#### 2.5.1 Temporal group ICA with dual regression

**Fig. 2:**
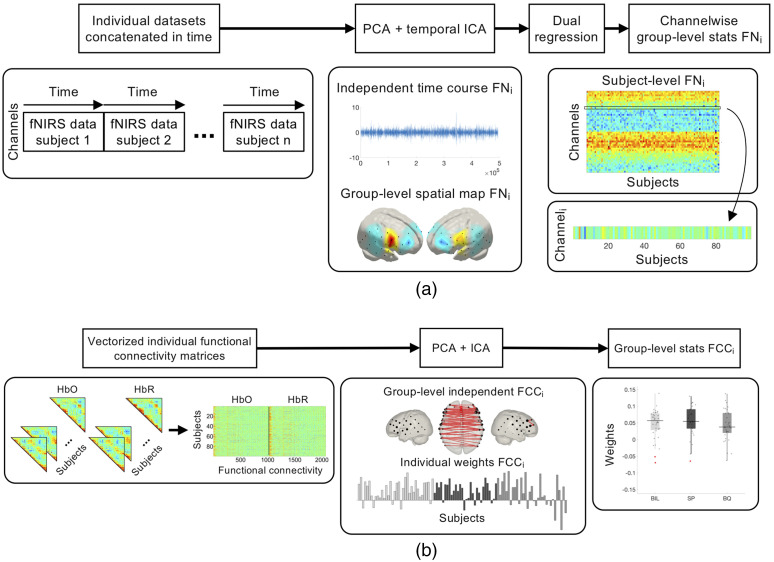
(a) Processing pipeline for tGICA and (b) connICA methods.

Group-level FNs were computed by means of a tGICA approach [Fig. 2(a)]^42^ by temporally concatenating participants’ datasets after time series normalization to zero mean and unit variance—producing a single-group dataset with dimensions [channels (46) × Hb chromophores (2)] × [time points (5000) × participants (99)]. The FastICA algorithm^66^ was applied to the group dataset to extract 15 ICs. This number corresponds to the number of principal components explaining 60% of group data variance, which was established previously using principal component analysis (PCA). The number of ICs was determined using three criteria based on the consistency of the components across different initializations of the ICA algorithm, the anticorrelation between the Hb chromophores, as well as the percentage of data variance explained by each IC (see the Supplementary Material for additional details on ICA model order selection). The subject-specific spatial maps associated with each independent FN were obtained using a dual regression approach. This two-step method involves an initial spatial regression of the tGICA spatial maps to the individual fNIRS datasets to obtain the subject-specific time courses associated with each group-level IC. A linear model fit is computed between the estimated subject-specific time courses and fNIRS datasets to estimate the subject-specific spatial maps.

Statistical analyses were performed channelwise for each FN using a one-way random effects ANOVA with language background as a factor (i.e., BIL, SP, and BQ), resulting in 15 spatial maps of between group differences (i.e., channelwise F-test). Statistical tests were corrected for multiple comparisons at the channel level using the false discovery rate (FDR, q<0.05) method.^67^ Bayesian hypothesis testing was also performed to estimate the relative likelihood of the data under the null and the alternative models.^45^ To quantify the plausibility of the absence of an effect (i.e., evidence of absence), we repeated our group-level statistical analyses for each FN based on a Bayesian ANOVA using the R package BayesFactor.^68^

#### 2.5.2 Connectome-based analyses

A connICA approach was performed based on infants’ functional connectomes.^44^ For each individual, the temporal synchronization between channels was evaluated by computing a pairwise robust Pearson’s correlation between the time courses of the HbO and HbR signals separately at every channel for each infant. Robust Pearson’s correlation reduces the contribution of possible outlier time points (e.g., due to residual motion artifacts after preprocessing) in the correlation estimation.^69^ Briefly, for each i, j element representing the preprocessed time series of channels i and j, a joint weighting matrix is calculated as

$$r\{t\}=\sqrt{{i_{\left\{t\right\}}}^{2}+{j_{\left\{t\right\}}}^{2}}.$$

A weighting function (S) defined as

$$S(\frac{r}{\sigma })=\{\begin{array}{ll} 1-(\frac{r}{\sigma -k}{)}^{2}, & |\frac{r}{\sigma }|<k\\ 0, & |\frac{r}{\sigma }|\geq k \end{array}$$

is applied to each i, j element such that $i_{W}=Si$ and $j_{W}=Sj$. The correlation for each entry of the functional connectivity matrix is computed between the preprocessed weighted signals $i_{W}$, $j_{W}$. The tuning parameter k=4.685 is the value typically employed in the literature which preserves 95% of statistical efficiency in the absence of outliers.^69^ We calculated σ from the medianabsolute deviation (MAD) of the signal r as σ=1.4826 MAD(r), using a constant scale factor that is standard for normally distributed data.^70^ Individual robust functional connectivity matrices representing the temporal association between channels were defined for HbO and HbR, where the $i_{W}$, $j_{W}$ element reflects the robust Pearson’s correlation between channels $i_{W}$ and $j_{W}$. For the sake of simplicity, hereinafter the robust functional connectivity matrices will be referred to as functional connectivity matrices. Individual functional connectivity matrices were converted from r values to z scores by Fisher’s r-to-z transformation and averaged across subjects within each experimental group.

Individual functional connectivity matrices of HbO and HbR were input to a hybrid connectome-based ICA (connICA) [Fig. 2(b)].^44^^,^^71^ The upper triangular part of the symmetric functional connectivity matrices of HbO and HbR were vectorized and concatenated for each individual. These vectors were concatenated in rows to form a group-level functional connectivity matrix of dimensions [99 participants] × [1035 connectivity pairs × 2 Hb chromophores]. The integration of the information on functional connectivity provided by HbO and HbR was done under the premise that similar RSFC patterns should be observed across Hb chromophores.^36^^,^^72^ Next, the FastICA algorithm was applied to the group-level functional connectivity matrix to obtain a set of latent group-level independent FCCs and their corresponding weights in each participant. From this analysis, 11 FCCs were extracted; a number that is equal to the number of principal components necessary to explain 60% of the group data variance. The choice of this parameter is explained in the Supplementary Material. Finally, the individual IC weights were evaluated as random effects. An ANOVA was performed with language background as a factor (i.e., BIL, SP, and BQ) to examine differences across experimental groups in the prominence of the extracted independent functional components. Statistical tests were corrected for multiple comparisons at the component level using the FDR (q<0.05) method. Bayesian ANOVAs were also performed on each FCC using the R package BayesFactor.^45^^,^^68^

## 3 Results

From the 123 infants, who participated in this study, following preprocessing and quality assessment of the fNIRS datasets, we were able to obtain 99 fNIRS recordings with good data quality for HbO and HbR signals. All the participants had recordings with a duration of 9 min, which were input for data analysis with tGICA and connICA. Only those FN and FCC with an interpretable spatial configuration are discussed in the main text. The complete sets of FN and FCC are available in the Supplementary Material. Frequentist and Bayesian tests were used to perform statistical comparisons on the observed FN and FCC as a function of the language background. ${\mathrm{BF}}_{10}$ values were used to quantify the relative likelihood of two competing models (i.e., performance of the model with and without the experimental factor, here language background) based on the information provided by the data. The ranges of the ${\mathrm{BF}}_{10}$ describing effect strength were formulated based on Wetzels and Wagenmakers (2012).^73^ In this formulation, a ${\mathrm{BF}}_{10}$ of value [3 to 10] represents a “substantial evidence for $H_{1}$,” and a ${\mathrm{BF}}_{10}$ of value [1 to 3] is described as “anecdotal evidence for $H_{1}$.” A ${\mathrm{BF}}_{10}$ of value [1] provides “no evidence” for any of the models. This can be interpreted as data being not sufficiently informative. Equivalent ranges are used for describing evidence for $H_{0}$, which can be obtained by calculating ${\mathrm{BF}}_{01}=1{/\mathrm{BF}}_{10}$.

**Fig. 3:**
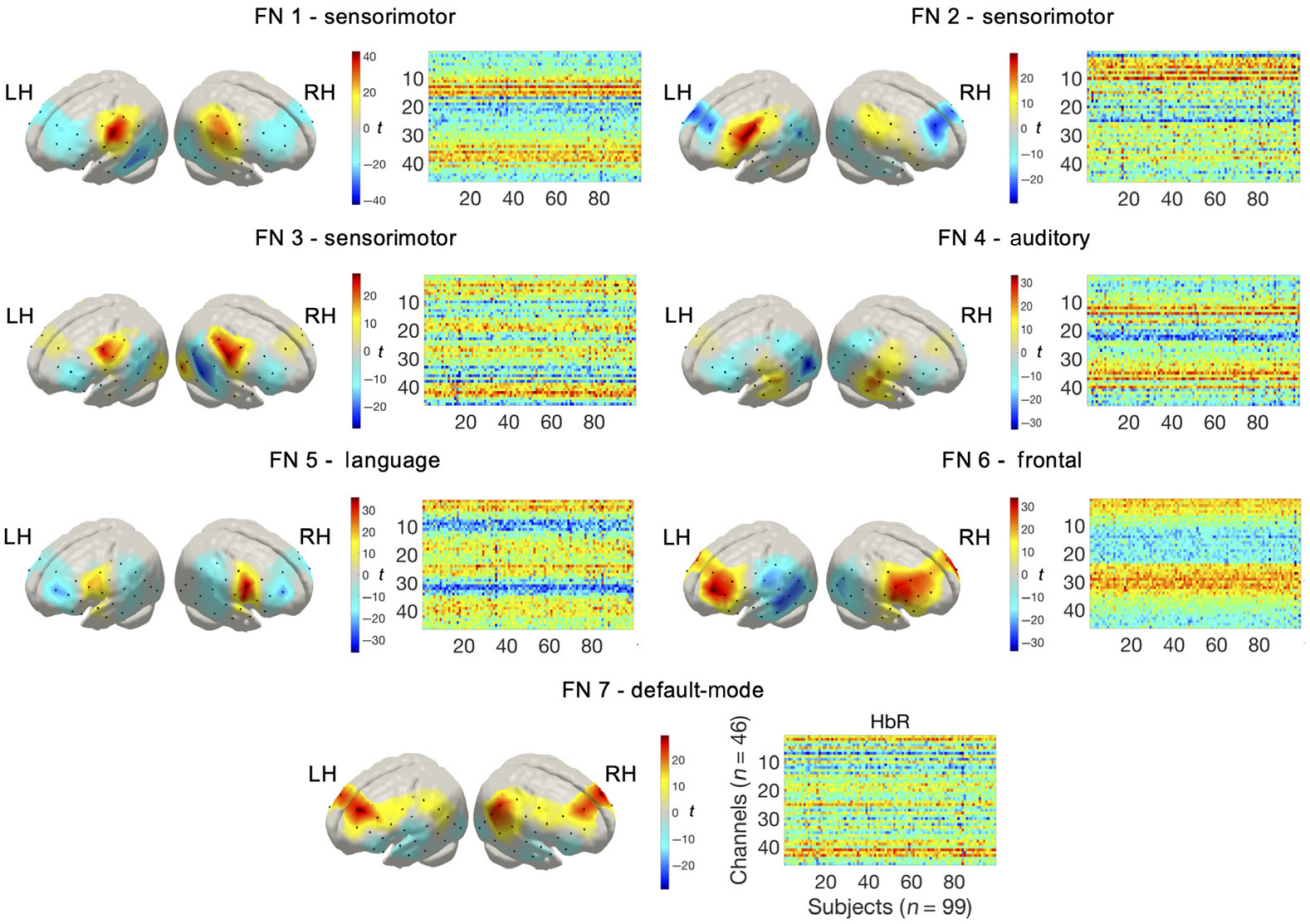
FNs representing the spatial maps derived from the tGICA method (HbR). Colorbar shows the t-value of the channel level one-sample t-test computed for each spatial map.

Group ICA spatial maps representing FNs of temporally independent spontaneous hemodynamic activity are displayed in Fig. 3. FNs are depicted as t-statistical maps from one-sample t-tests on the subject-specific reconstructed spatial maps. The observed networks were robust across multiple realizations of the ICA algorithm based on ICASSO,^74^ showing consistency values (Iq) ranging from 0.49 to 0.91. FNs also exhibited high consistency across HbO and HbR, displaying correlation r values between −0.97 and −0.99, as expected due to hemodynamic physiology (see the Supplementary Material for a full description of these metrics). The first three FNs, labeled as sensorimotor networks (FN 1-3), depict a symmetric pattern over bilateral areas in the precentral and postcentral gyrus. FN 4 and FN 5 cover mainly areas located in the inferior frontal gyrus and the superior temporal gyrus that can be associated with the auditory and the language networks, respectively. Two FNs were observed over frontal regions, including FN 6 and FN 7. FN 6 is confined to regions in the middle and superior frontal gyrus. FN 7 comprised middle frontal areas and areas in the inferior parietal gyrus which can be related to the outer brain regions of the default-mode network that is typically observed in RSFC studies with fMRI. The observed FNs exhibited significant patterns of anticorrelated spontaneous activity. In FN 1-5, anticorrelated patterns were observed involving superior and middle frontal areas, posterior areas in the inferior and middle temporal gyrus, and inferior parietal regions. FN 6 showed anticorrelated activity with posterior temporal and inferior parietal regions. In FN 7, the negative spatial pattern was less prominent than the positive part and included inferior frontal and superior temporal regions.

HbO and HbR FNs were reconstructed to the subject space using dual regression to extract the subject-specific FNs. Between group statistical comparisons were conducted to assess the effect of early bilingual exposure on each channel and network (one-way ANOVA at the channel level, FDR corrected among 46 channels, q<0.05). Significant differences between experimental groups were only observed on isolated channels, with most effects not surviving multiple comparison correction and not being consistent across HbO and HbR. Bayesian tests assessing channelwise group differences revealed an overall higher likelihood of the null hypothesis (i.e., substantial evidence for $H_{0}$, HbO: 602/690 tests, and HbR: 596/690 tests). Substantial evidence for $H_{1}$ denoting differences between groups in FN was found only on isolated channels (HbO: 11/690 tests and HbR: 7/690 tests), and these channels did not show consistency across HbO and HbR (a full description is provided in the Supplementary Material).

**Fig. 4:**
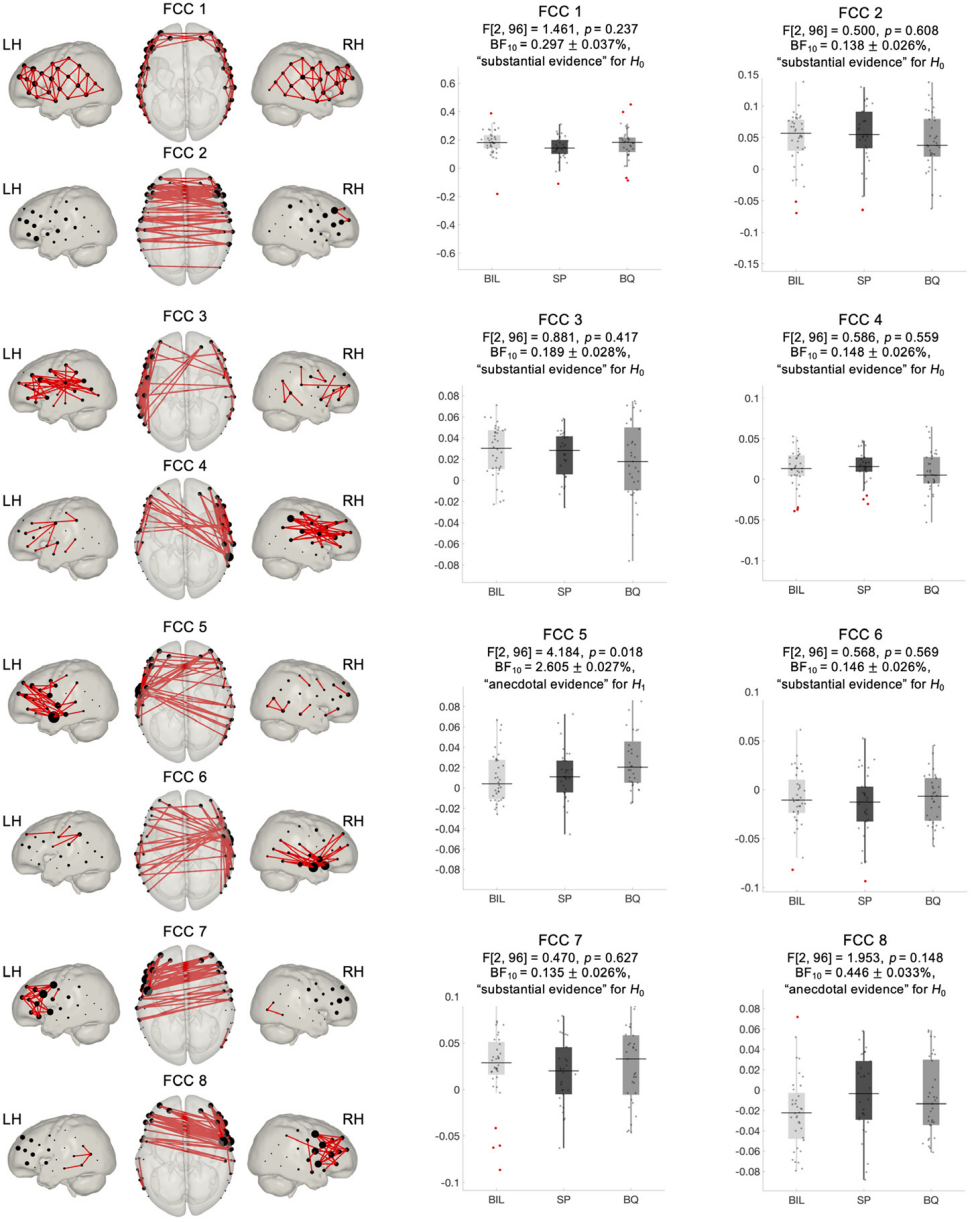
FCCs estimated using connICA and associated statistics (frequentist and Bayesian) assessing between-group differences. Components have been threshold to show only the top 10% of connections (absolute value). Node size was adjusted based on the number of connections reaching each node.

The input of the connICA method is the individual functional connectivity matrices computed based on a robust Pearson’s correlation approach. A high degree of similarity was observed at the individual and at the group level in the configuration of functional connectivity matrices (see the Supplementary Material). A marked negative correlation between HbO and HbR and a stronger correlation between homotopic regions were also evidenced on these matrices. These features were considered indicative of the quality and reliability of the datasets. Group-level FCC extracted from the connICA analysis are depicted in Fig. 4. For the sake of representation, each FCC plot only displays 10% largest positive connections between nodes (i.e., fNIRS channels), with node size representing the number of connections linked to it. FCC showed a high level of robustness based on the ICASSO algorithm, with Iq values between 0.5 and 0.96, and a large degree of similarity between HbO and HbR, with correlation r values between 0.7 and 0.95. FCC 1 is characterized by local, short-range, connections between adjacent nodes. It involves within hemisphere connections between nodes over the whole fNIRS setup, with interhemispheric connections constrained to the most anterior nodes. FCC 2 reflects functional connectivity between homotopic channels across hemispheres. FCC 3 and FCC 4 show a high degree of symmetry, displaying mainly short and long-range within hemisphere connections. FCC 5 and FCC 6 also show a highly symmetric pattern, revealing that the nodes located over the superior temporal gyrus are functional hubs with a large number of intrahemispheric connections between temporal and frontal regions, and interhemispheric connections with frontal and posterior temporo-parietal regions. Finally, FCC 7 and FCC 8 are also highly symmetric with their main functional hubs located in precentral and inferior frontal regions and showing intrahemispheric connections across frontal and precentral regions and interhemispheric connections between frontal, superior temporal, and precentral regions (the full set of extracted FCC is provided in the Supplementary Material).

Statistical analyses assessing significant differences across experimental groups were computed on the individual weights that quantify the prominence of each independent FCC in each individual. A one-way ANOVA at the FCC level indicated no significant differences between monolingual and bilingual infants after multiple comparisons correction by the number of components (FDR corrected among 11 FCC, q<0.05). Bayes factors (${\mathrm{BF}}_{10}$) indicated that, in most FCC, the data were more likely under $H_{0}$ than under $H_{1}$ (Fig. 4). A substantial evidence for $H_{0}$ was observed in 6 out of 8 FCC, whereas FCC 8 showed anecdotal evidence for $H_{0}$. FCC 5 had the largest evidence (anecdotal) for differences across the groups and would be the only FCC to exceed significance (p=0.018) in frequentist F-tests without multiple comparisons correction.

## 4 Discussion

This work evaluated the effect of bilingualism on RSFC based on high-quality fNIRS datasets acquired in 4-month-old monolingual and bilingual infants. To the best of our knowledge, this sample of 99 valid participants with 9 min fNIRS recordings per infant is the largest dataset to study RSFC in this age group. Our analyses identified large-scale group-level RSFC patterns in the 4-month-old infant brain by implementing two data analysis approaches based on ICA that search for independence between, either the time-courses of spontaneous hemodynamic activity measured with fNIRS (i.e., tGICA),^42^^,^^43^ or in the connectivity patterns across multiple individual functional connectivity matrices (connICA).^44^

The main goal of this study was to assess whether an early and continued exposure to a bilingual environment during the first months of life might impact the configuration of the emerging functional connectivity. The human brain’s capacity to adapt to long-term environmental factors manifests prominently during the first stages of development and it is particularly relevant during early language experience. For this reason, the link between long-term exposure to two languages and its effect on cognitive and functional brain development has received great attention in recent years.^75^ Our frequentist group-level analyses showed no significant differences between the RSFC of monolingual and bilingual infants at 4 months of age. Bayesian statistical analyses confirmed that $H_{0}$ (i.e., no group differences) was overall more likely than the alternative model for the two functional connectivity analysis methods. Overall, an effect of linguistic background in RSFC was not observed, suggesting that an early bilingual environment might not affect the configuration of intrinsic functional connectivity at this age. However, FCC 5, in which the highest evidence for $H_{1}$ was observed for both frequentist and Bayesian tests, covers areas that largely overlap with language and auditory regions. One would expect that, if differences due to a dissimilar linguistic background exist, they could potentially manifest in such an FN/component (in fact, one would have concluded so if multiple comparisons correction were not applied). Despite the acquired sample size and fNIRS recording length, these results might indicate that the number of participants might have still been insufficient to robustly detect this effect. Here RSFC was examined in 4-month-old infants, as this is the first point in development, in which the behavioral and neural consequences of a bilingual effect have been described.^2^^–^^4^^,^^8^^,^^10^ Bilingualism has been only shown to modulate RSFC in adult participants thus far,^29^^–^^31^ and evidence of differences between monolingual and bilingual infants in previous neuroimaging studies manifested during explicit language tasks only.^8^^–^^13^ It is, therefore, a possibility that, if differences between monolinguals and bilinguals exist at this age, they might only be observable during the performance of specific linguistic tasks. Further research with monolingual and bilingual infants at different ages should also help clarify if the differences observed in adults’ functional connectivity might only emerge at a later stage in neural development.

The spatial resolution of the current optode setup might have been insufficient to detect subtle variations in RSFC configuration induced by early bilingualism. Variability in head size and shape across participants and the lack of precise spatial information of our probe might have increased spatial variance in channel position on the head, thus limiting the spatial accuracy of our findings and reducing statistical power in group-level analysis. However, a recent work demonstrates that differences in head size and shape do not lead to substantial differences in channel positions and localization in infants (i.e., differences in head size make little difference over the range of head circumferences for a given age).^76^ It is also challenging to ensure a consistent probe positioning when acquiring fNIRS data from infant subjects, as most methods for optode localization require participants to remain motionless for long periods of time, which is generally not suitable. Recently, photogrammetry methods that provide faster and easier optode registration procedures demonstrated their validity and reliability for probe position estimation.^77^^,^^78^ A wider adoption of these methods by the community could help increase within and between-subject reproducibility of fNIRS measurements on developmental populations. The spatial accuracy of our findings might have also been reduced by the use of 6-month-old head templates as opposed to age-matched templates. To the best of our knowledge, the nearest parcellation atlas that is publicly available to 4-months is at 6-months.^48^ We used a 6-month structural atlas, which can be better registered to a parcellation atlas from the same cohort.

Testing sleeping infants might have prevented us from detecting subtle differences in RSFC properties across experimental groups.^79^^,^^80^ Nonetheless, previous studies assessing RSFC in infants have also been conducted with sleeping infants, irrespective of the imaging modality (i.e., fMRI or fNIRS), and were able to identify RSFC differences induced by prenatal and postnatal factors such as premature birth^25^ or socioeconomic status.^28^ Brain imaging techniques are particularly sensitive to motion-induced artifacts commonly observed in acquisitions on awake infants. In our experience, collecting RSFC data from awake infants considerably degrades the reliability of the inferred temporal correlations between voxel or channel time courses.^69^ Because our goal was to collect high-quality RSFC data, we tested participants during natural sleep, which also allowed us to perform longer recordings than those normally reported in the literature. All infants were tested under similar conditions (i.e., immediately after they fall asleep) with the aim of ensuring a homogenous sleep state and minimizing any possible confound due to different arousal states across participants.^21^ However, assessing sleep state through behavioral and/or electrophysiological measures would have been desirable to provide a more accurate control.^80^

The proposed fNIRS data preprocessing and analysis pipeline enabled us to reliably characterize group-level RSFC patterns in 4-month-old infants. FN extracted with tGICA demonstrated a marked bilateral functional relationship between homotopic brain regions in HbO and HbR hemodynamic fluctuations. The spatial configuration of the FN indicates that, at this age, RSFC predominantly consists of synchronous activity between anatomically and functionally similar regions across hemispheres, as already described in the previous works.^18^^,^^81^^–^^83^ We showed FN located in primary sensorimotor (FN 1-3) and auditory regions (FN 4) which have been repeatedly reported in infant studies using fMRI,^28^^,^^81^^,^^83^ but from which evidence from infant studies using optical methods was limited.^18^^,^^39^^,^^72^ FN 5 overlaps with language related regions spreading over the inferior frontal gyrus and superior temporal gyrus. Interestingly, a stronger involvement of the right hemisphere in the auditory (FN 5) and language (FN 4) networks can be observed in Fig. 3, which matches previous observations showing increased activity in the right hemisphere for speech input.^82^^,^^84^^,^^85^ Homotopic areas over frontal regions also demonstrated a high degree of functional synchronization. The spatial organization of FN 6, involving multiple channels within frontal regions and across the midline, supports existing evidence showing that frontal regions become functionally connected during the first year of life.^28^^,^^83^ FN 7 showed a symmetric functional connectivity pattern involving channels in middle frontal and inferior parietal regions, which is partly consistent with the spatial topology of the default-mode network, which exhibits its most prominent activity when the subject is not engaged in any particular task.^19^ Evidence of a developing default-mode network has often been reported in infants with fMRI.^86^ Using fNIRS, evidence of a gradual increase of frontal–temporoparietal connectivity in resting state between 11 and 36 months of age as a prospective precursor of the developing default-mode network has been observed.^87^^,^^88^ However, due to the limited spatial resolution of the current experimental setup, and the inability of fNIRS to measure deep medial and subcortical regions, such as the posterior cingulate cortex and the precuneus, our results should be interpreted with caution.

The connICA method allowed us to identify group-level macroscale properties of functional connectivity based on individual functional connectivity matrices. The functional relationship between fNIRS channels formed coherent interregional ensembles with distinct topological properties of large-scale functional connectivity. The first functional connectome component (FCC 1) showed short-range functional connectivity between adjacent channels, spanning the complete fNIRS setup. This functional connectivity pattern reflecting the intrinsic functional configuration of the infant brain has been shown to progressively decrease over the course of development, whereas long-distance connections tend to increase toward a more distributed functional brain organization.^18^^,^^89^ FCC 2 displayed interhemispheric correlations between homotopic regions. This type of functional connectivity is prevalent in most studies assessing RSFC in infant subjects and has been linked with the interaction between functional and structural brain maturation.^18^^,^^28^^,^^81^^,^^83^ Due to the marked spatial symmetry observed in components FCC 3 to FCC 8, we presented them in pairs in Fig. 4. FCC 3 and FCC 4 displayed mostly intrahemispheric connectivity between anterior and posterior brain regions in the left and right hemispheres, respectively. Similarly, FNs extracted with our tGICA approach also showed patterns of long-range intrahemispheric connectivity. However, evidence from previous studies suggests that at this age this type of connectivity is still immature.^18^^,^^28^ FCC 5 and FCC 6 showed functional hubs in the left and right temporal areas, respectively, which were densely interconnected with inferior frontal and posterior temporal regions within and across hemispheres and mainly covered auditory and language related areas. In FCC 7 and FCC 8, connections converged over channels located in precentral and inferior frontal gyrus, which showed intra- and interhemispheric connections with channels localized in frontal regions. Due to their spatial characteristics, these components might well represent the activity of motor and language networks, which have been consistently identified in infant populations.

Our results showed reliable patterns of correlated and anticorrelated activity within the observed FNs and FCCs. An interesting finding in this study is that the anticorrelated networks observed in our primary FNs (FN 1-5) considerably overlap with the spatial configuration of the FN labeled as default-mode network (FN 7). It is, therefore, a possibility that this activity might reflect the interaction between task-positive and task-negative brain regions. One question that might arise from these findings is whether the observed patterns of negative functional connectivity are the result of our preprocessing pipeline including global signal regression, or if they reflect intrinsic, functionally meaningful properties of network organization.^90^ Recent guidelines suggest that, in infants, regressing out signals from short-separation channels could reduce the impact of confounding physiological signals from extracerebral tissues, increasing the reliability of fNIRS measurements. When short-separation channels are not available in the fNIRS setup, applying a signal processing method to remove physiological confounds (e.g., PCA or global signal regression) is a recommended alternative.^91^ This preprocessing step is necessary to account for the effects of widespread systemic physiological confounds that are commonly observed in fNIRS recordings.^57^^,^^58^^,^^91^ As seen from the individual quality assessment figures (see the Supplementary Material), global signal regression removes physiological components that would otherwise artificially increase global connectivity, thus increasing the risk for false positives. Future work should specifically address what method is more appropriate to remove physiological confounds in developmental populations, where this issue has usually not been considered and where fNIRS setups including short-separation channels are less feasible. We computed FN and FCC without applying global signal regression and found that anticorrelated patterns of functional connectivity were still present. Most previous optical imaging studies assessing RSFC reported only positive correlations or presented both positive and negative correlations in the results but only discussed the former.^38^^,^^39^ This has been mostly due to the lack of a straightforward interpretation of the observed anticorrelated activity in the literature.^90^

## 5 Conclusion

This work describes and compares RSFC in 4-month-old infants considering a large sample size, high-quality datasets, comprehensive data quality assessment, and preprocessing routines. We demonstrate the consistency of our recordings with the expected physiological and functional properties of the fNIRS signal, thus strengthening the reliability of the observed RSFC patterns (i.e., FN and FCC). As for our main theoretical question, group-level statistical comparisons based on frequentist and Bayesian statistics revealed no differences between monolingual and bilingual infants’ RSFC at 4 months of age, although connICA revealed a trend for significant effects in a left-lateralized component overlapping auditory and language-related frontal and temporal regions. In light of previous research that demonstrated neural adaptations in bilingual infants during linguistic tasks at this age, our results suggest that intrinsic functional brain networks are not affected by bilingual experience during the earliest stages of life. Considering previously reported differences in adult monolingual versus bilingual RSFC patterns, in which stage in development changes in RSFC based on language environment begin to appear remains open for future research. Finally, we believe that negative results in correctly motivated, rigorously performed, and comprehensively described research are an important component of scientific literature. In our view, the quality of a research output should not be judged based on the direction or the significance of the observed findings. Negative results can help filling the gaps of current scientific knowledge and are essential for improving research transparency and reproducibility.^92^^,^^93^
