# Supplementary material for: Group-level cortical functional connectivity patterns using fNIRS: assessing the effect of bilingualism in young infants
Source: Neurophotonics. 2021 Jun 12;8(2):025011. doi: 10.1117/1.NPh.8.2.025011 (PMC8200331; doi:10.1117/1.NPh.8.2.025011)
Supplement: Supplementary file 1 [file NPh_008_025011_SD001.pdf]

Supplementary materials for

Group-level cortical functional connectivity patterns using fNIRS: Assessing the effect of bilingualism in young infants

This document includes the following information:

- fNIRS device specifications, and information describing the data preprocessing and analysis steps, parameters and functions employed in this study (supplementary table 1).
- Group level data quality assessment and replication analysis (supplementary figure 1).
- ICA model order selection (supplementary figure 2).
- Supplementary results: temporal group ICA analysis (supplementary figures 3 and 4 and supplementary table 2).
- Supplementary results: example of functional connectivity matrices at the individual and group level (supplementary figure 5).
- Supplementary results: connICA analysis (supplementary figure 6 and supplementary table 3).
- References.
- Data quality assessment figures.

SYSTEM SPECIFICATIONS		
**Company and model**	NIRx NIRScout, CW-NIRS device	
**Wavelengths**	[760, 850]	
**Sampling frequency**	8.93 Hz	
**Number of sources**	16 (14 analysed - occipital excluded)	
**Number of detectors**	24 (19 analysed - occipital excluded)	
**Number of channels**	52 (46 analysed - occipital excluded)	
**Channel localization**	Head-based fiducial locations (10-20 system)	
**Source-Detector distances**	[20 - 45 mm, see main text Fig. 1]	
**STEPS**	**PARAMETERS**	**FUNCTION/TOOLBOX**
Data preprocessing		
**1) Intensity to optical density**		*Homer2*[1]*:* hmrIntensity2OD
**2) Motion detection and censoring**	Visually	*Matlab:* Plot
**3) Motion correction**	[Wavelet: d4; threshold: 0.02; boundary: reflection; chsearch: moderate; nscale: extreme]	*BrainWavelet Toolbox*[2]
**4) Optical density to HbO and HbR**	DPF: [5.3, 4.2]	*Homer2:* hmrOD2conc
**5) Filter and global signal regression**	HPF: 4th order Legendre polynomials; LPF: 0.09Hz	*In house script:* GitHub
Data analysis		
**Temporal group ICA**	15 independent components	*ICASSO Toolbox*[3]
**connICA**	11 independent components	*ConnICA Toolbox*[4]
Statistical analysis		
**One-way analysis of variance - ANOVA**	Language background [Bilingual, Spanish, Basque]	*Matlab:* anova1
**Bayesian statistics - ANOVA**	Language background [Bilingual, Spanish, Basque]	*JASP*

Supplementary Table 1. Information describing the fNIRS device specifications, and the data preprocessing and analysis steps, parameters and functions employed in this study. CW: Continuous wave; DPF: Differential pathlength factor; HPF: High pass filter; LPF: Low pass filter; ICA: Independent component analysis.
In house scripts will be made publicly available at: https://github.com/borjablanco/RS_4months

[1] Homer2 downloaded at:
https://homer-fnirs.org/
[2] Brain Wavelet Toolbox v2.0 is available at:
https://www.brainwavelet.org/downloads/brainwavelet-toolbox/
[3] ICASSO Toolbox 1.21 is available at:
https://research.ics.aalto.fi/ica/icasso/
[4] ConnICA Toolbox 1.0 can be downloaded at:
https://engineering.purdue.edu/ConnplexityLab/publications

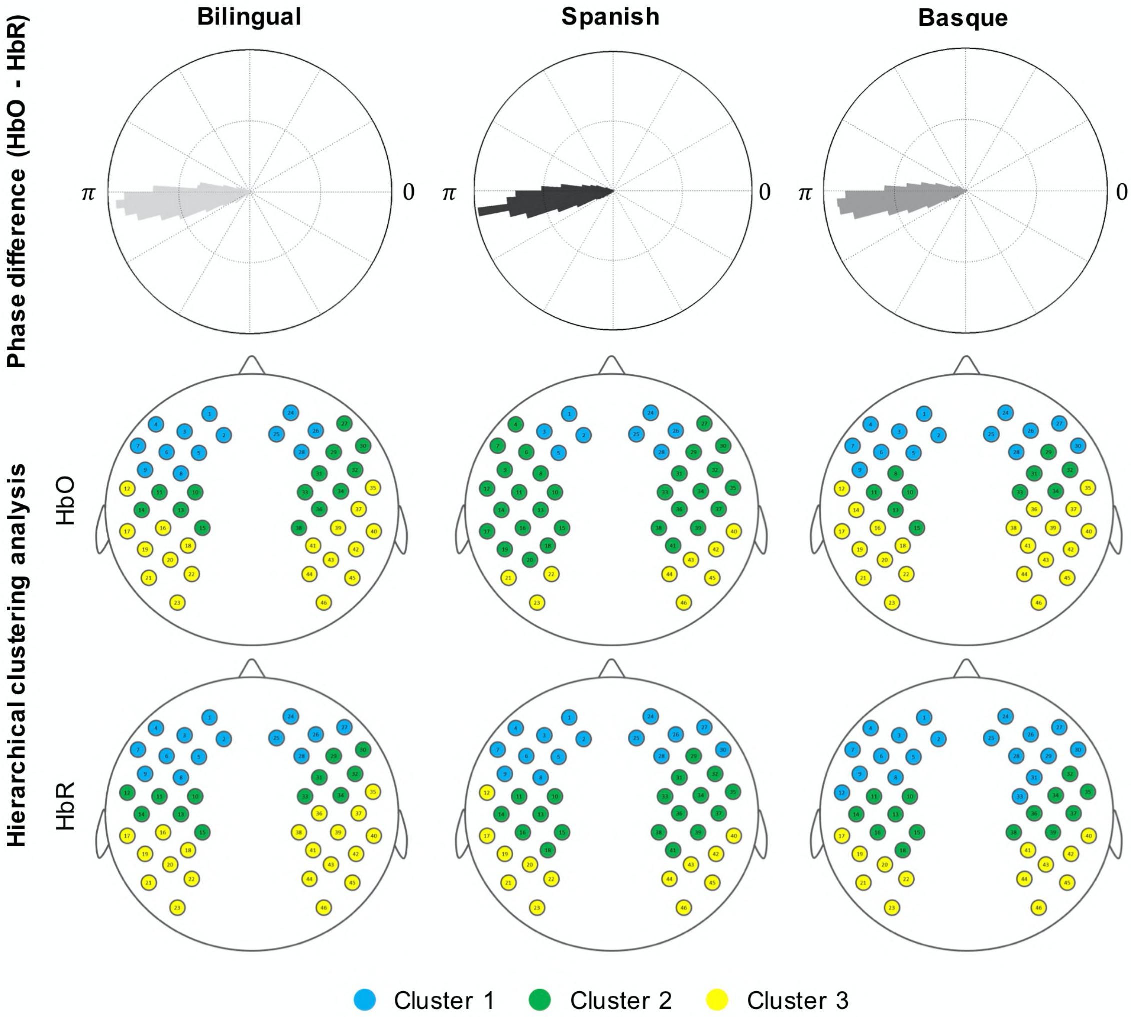

Supplementary Figure 1. Group-level data quality assessment plots replicating two previous infant resting-state functional connectivity studies using fNIRS. First row shows the channelwise average phase difference (hPod value, **Watanabe et al., 2017**) between HbO and HbR in each experimental group. The three groups show a similar pattern characterized by an antiphase state between HbO and HbR, replicating previous outcomes. Second and third rows in this figure show the results of a hierarchical clustering approach (**Homae et al., 2010**) in which channels' time series are clustered based on similarity. A similar cluster configuration can be observed across groups in HbO and HbR. Cluster 1 is formed by channels located in the most anterior part of both hemispheres. Cluster 2 comprises channels located in middle brain regions. Channels located in the most posterior part of the setup are grouped together in cluster 3.

Model order selection for Independent Component Analysis

A recurrent issue in studies using independent component analysis (ICA) to examine resting-state functional connectivity is how to determine the number of IC to be estimated. Here we propose a data-driven approach to determine the number of estimated ICA components by computing two metrics that exploit specific defining properties of ICA estimation and fNIRS data: 1) the robustness of the components across multiple realization of the ICA **(Himberg et al., 2004)** and 2) the assumed similarity between the HbO and HbR derived IC.

First, a valid interpretation of the results requires selecting only those independent components that are considered robust; being robustness defined by the identifiability of the component across multiple runs of the ICA algorithm with different random initializations. A principal component analysis (PCA) is commonly performed prior to ICA to reduce the dimensionality of the data and/or remove noise components. The number of ICA components to be estimated usually corresponds with the number of PCA components that are retained. We evaluated the robustness of the estimated IC across multiple PCA thresholds by using ICASSO **(Himberg et al., 2004; Damaraju et al., 2014)**. Concretely, 100 realizations of ICA were computed for different PCA thresholds (i.e., 60, 65, 70, 75, 80, 85, 90, 95 and 99) representing the percentage of data variance explained. For the two ICA methods employed in the current work (temporal group ICA and connICA), ICASSO quality index (Iq) values were obtained for each component and for each PCA threshold (Supplementary Figure 2 - volcano plots). This index quantifies the robustness of the estimated components across ICA realizations, with values ranging between 0 (low robustness) and 1 (high robustness). In the temporal group ICA approach, higher Iq values (i.e. more robustness) were obtained at the lower PCA thresholds (60% and 65% explained variance). For higher thresholds, the first 15-20 IC also showed high Iq values. However, these values start decaying as the number of IC increased and were particularly low for PCA thresholds above 80%. A similar trend can be observed for connICA. The highest Iq values were obtained at the lower PCA thresholds (60% - 70% explained variance), whereas the Iq values for higher PCA thresholds were only high up to the first 15 IC and then progressively decreased.

Second, the expected statistical association between HbO and HbR chromophores can be used as a metric for evaluating the validity of the IC extracted from the ICA algorithm. A high correlation between the extracted HbO and HbR IC or maps implying consistency across Hb chromophores can be interpreted as a measure of physiological reliability **(Villringer and Chance, 1997; Wolf et al., 2002; Obrig and Villringer, 2003)**. In the temporal group ICA approach, IC are estimated from HbO and HbR time series, which are expected to be negatively correlated. A highly similar spatial configuration in the extracted spatial maps of both chromophores (i.e., channel weights) is expected, but with opposite sign (i.e., negative correlation). In our temporal group ICA analysis HbO and HbR spatial maps displayed a strong negative correlation at the lower PCA thresholds—up to 75% of explained variance (Supplementary Figure 2 - volcano plots). For higher thresholds, correlation between HbO and HbR components remained negative, but values decreased considerably. For the connICA approach, a strong positive correlation between the HbO and HbR independent functional components is expected, as they are estimated from the HbO and HbR functional connectivity matrices, which show alike topology across chromophores. The analysis for the connICA method yielded similar results as the previous method. At the lower PCA thresholds (60% and 65% explained variance) the correlation between HbO and HbR components was notably high ($r \geq 0.7$). At higher PCA thresholds, correlation between components remained positive, but with lower correlation values between HbO and HbR components.

Based on these two metrics (i.e., robustness of IC, and correlation between the extracted IC from HbO and HbR data) an appropriate choice for PCA model order, and in turn for the subsequent ICA method, corresponds to approximately 60% of the explained variance in both approaches (i.e., tGICA and connICA). To reinforce this choice, the variance explained by each IC was computed by calculating the accumulative data variance explained by the components. This analysis showed that for the two approaches with the selected 60% threshold, we are able to explain the largest amount of data variance, while also obtaining the highest values in our

robustness and consistency metrics. At higher PCA thresholds, explaining the same amount of variance would require including components that are not robust, or which do not show the expected association between HbO and HbR. Based on this information, in the temporal group ICA approach we selected 15 principal components corresponding to 60% explained variance. As a reference, a method for automatic dimensionality selection based on Laplace approximation **(Minka et al., 2001)** yielded a result of 27 components (i.e., 73.5% explained variance). For the connICA method we considered 11 principal components, which corresponds to 60% explained variance. For this method the Laplacian approximation produced a value of 17 components (i.e., 66.34% explained variance).

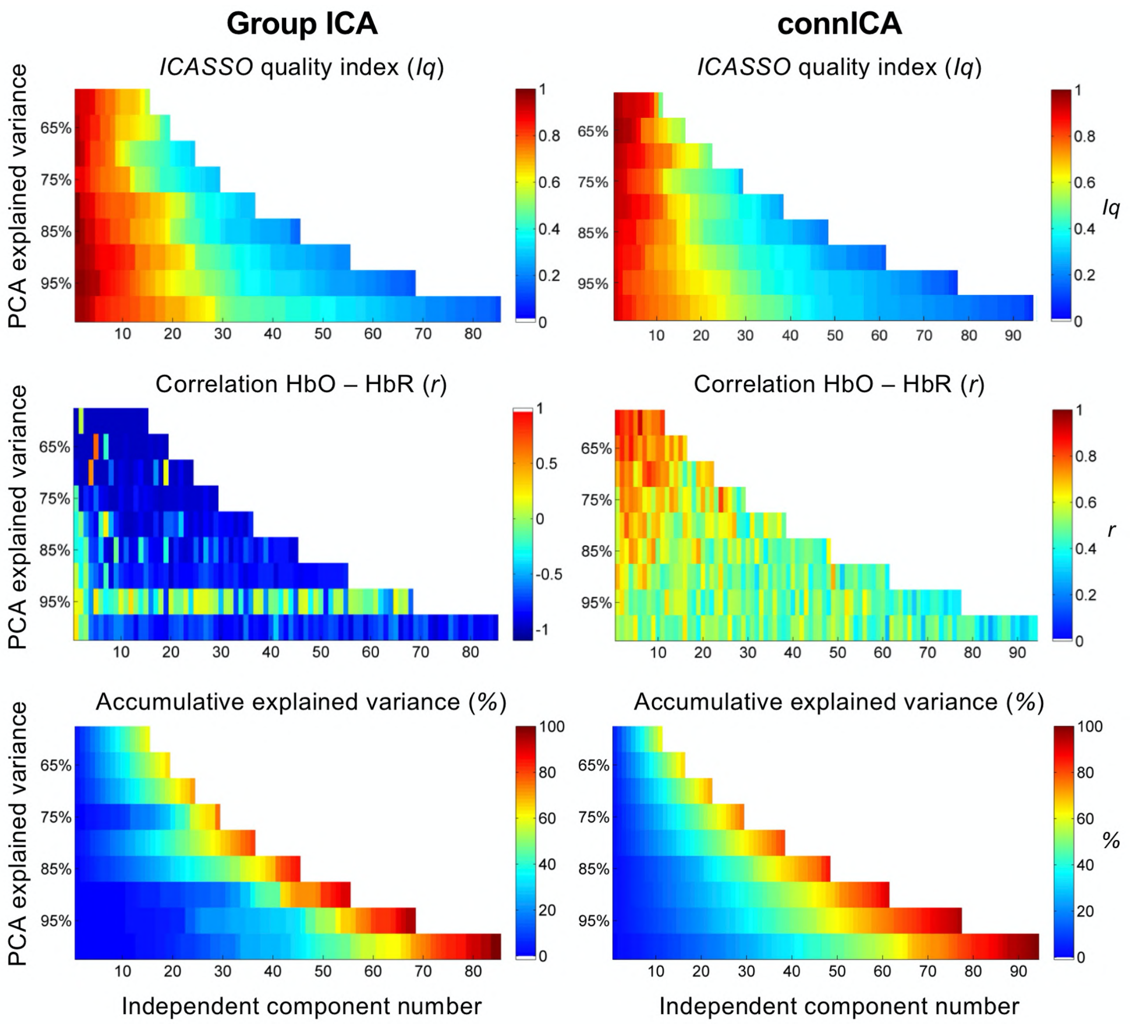

Supplementary Figure 2. Model order selection criteria for temporal group ICA (first column) and connICA (second column) methods at different PCA thresholds indicating the percentage of explained variance (y axis). First row depicts the cluster quality index *(Iq)* for each component as estimated using ICASSO, which represents component stability across multiple ICA realizations. Second row shows the correlation between the estimated HbO and HbR components. Third row shows the components' accumulative explained variance, being the maximum amount of explained variance determined by the specific PCA threshold.

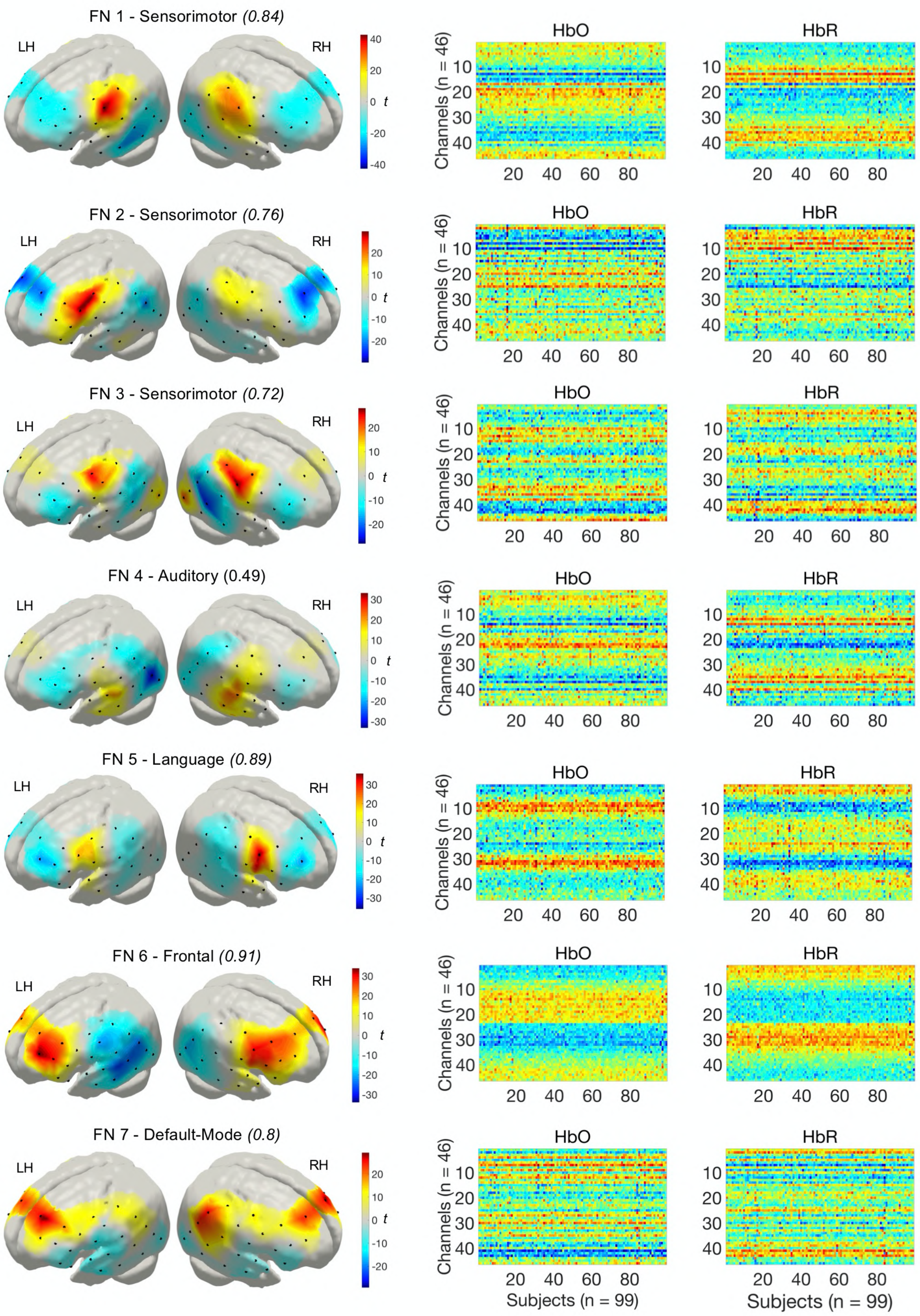

Figure continues in next page

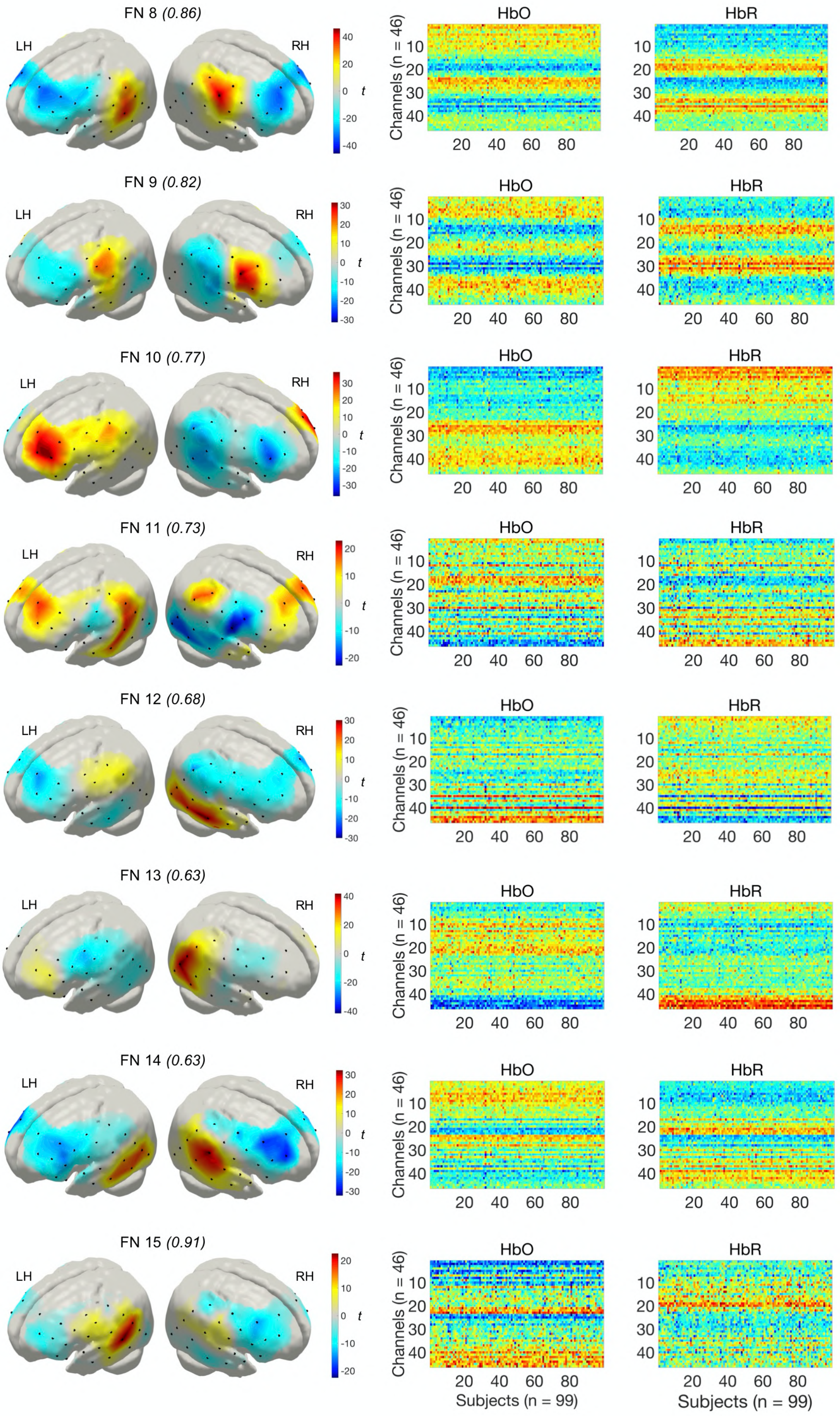

Supplementary Figure 3. Functional networks extracted with the temporal group ICA approach (n = 15, *Iq* values appear in parentheses). Due to the high similarity between HbO and HbR components, the associated spatial maps are displayed for HbR only. Functional networks were reconstructed to the subject space using dual regression. Subject-level maps for HbO and HbR are represented in matrix form in the middle and rightmost columns.

Funtional Network (FN)	*Iq*	*r*	*ssd (%)* total = 100	*ssd (%)* total = 60
FN 1 Sensorimotor	0.84	-0.99	8.8	4.1
FN 2 Sensorimotor	0.76	-0.98	4.3	3.9
FN 3 Sensorimotor	0.72	-0.99	5.5	3.9
FN 4 Auditory	0.49	-0.98	5.6	3.9
FN 5 Language	0.89	-0.99	7.4	4.0
FN 6 Frontal	0.91	-0.99	10.5	4.2
FN 7 Default-Mode	0.80	-0.97	6.2	4.0
FN 8	0.86	-0.99	8.9	4.1
FN 9	0.82	-0.99	7.2	4.0
FN 10	0.77	-0.98	7.3	4.0
FN 11	0.73	-0.98	4.5	3.9
FN 12	0.68	-0.98	5.7	3.9
FN 13	0.63	-0.98	5.1	3.9
FN 14	0.63	-0.99	9.0	4.1
FN 15	0.91	0.02	3.8	3.8

Supplementary Table 2. Temporal group ICA model order evaluation metrics for the PCA threshold selected in this study (i.e., 60% - 15 ICs). Sum of squared differences (ssd) are computed with respect to the data after PCA (total = 100%) and with respect to the original data without PCA (total = 60%). Iq = *ICASSO* cluster quality index; r = correlation coefficient between HbO and HbR spatial maps.

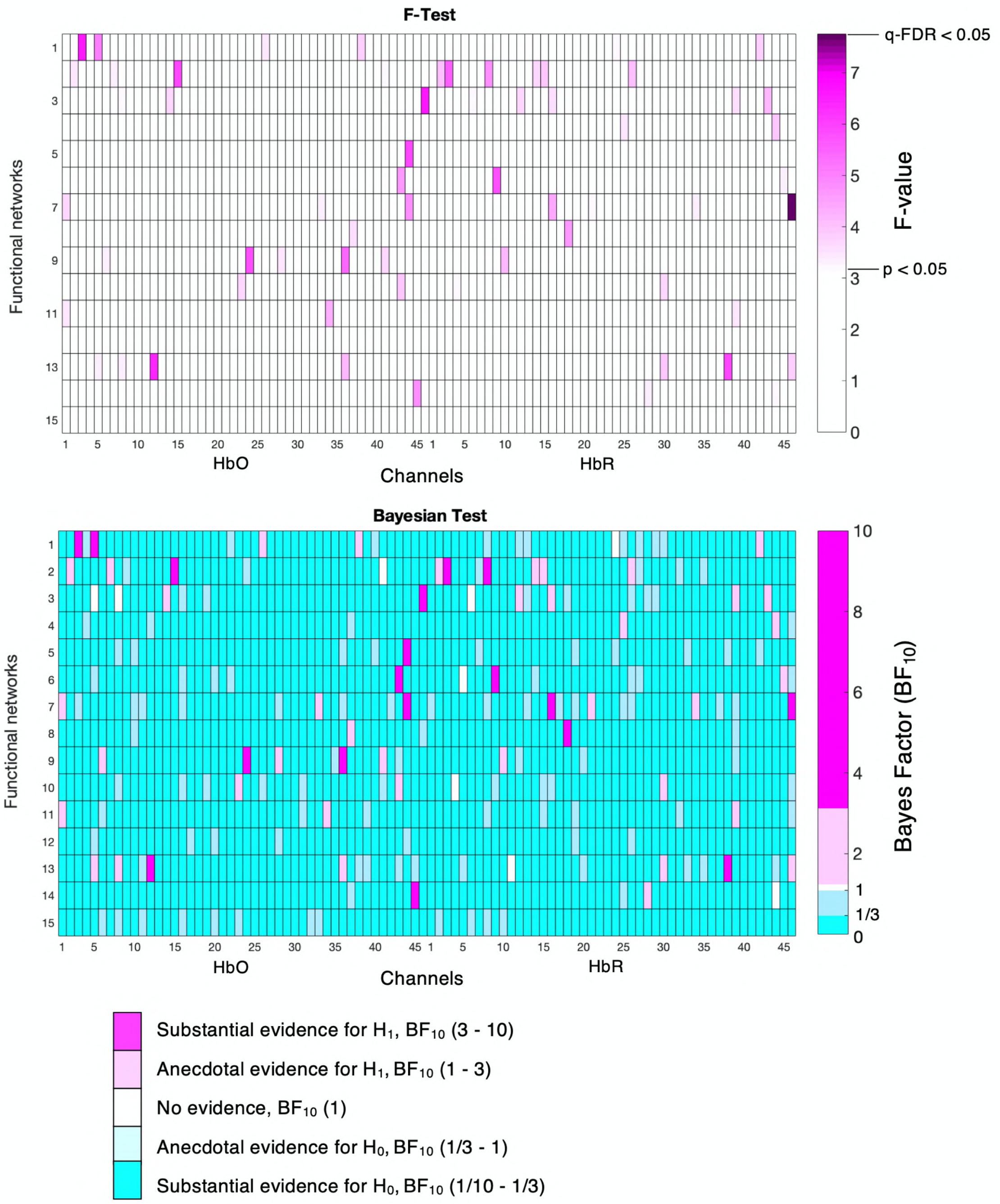

Supplementary Figure 4. Results of channelwise between group statistical comparisons (i.e., frequentist F-test) on the prominence of the extracted functional networks showed weak effects on isolated channels which were not consistent across HbO and HbR and which not survived multiple comparisons correction (FDR corrected among 46 channels, q<0.05). Group comparisons based on Bayesian statistics (**Wetzles and Wagenmakers, 2012; Keysers et al., 2020**) showed an overall increased likelihood for the null model H_0. A partial correspondence between frequentist and Bayesian tests can be observed in those channels showing potential evidence for H_1, but these effects are only present on isolated channels, and are not consistent across HbO and HbR. BF_{10} = Bayes Factor.

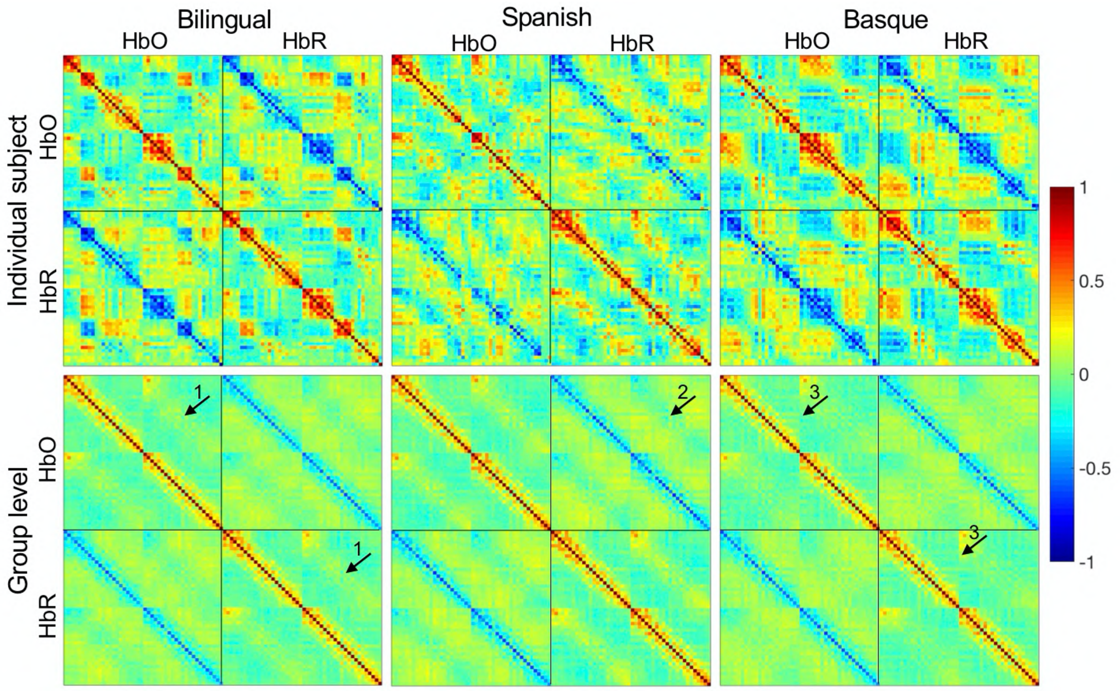

Supplementary Figure 5. Functional connectivity matrices of representative individual subjects (first row) and at the group level (second row) for each experimental group. Functional connectivity matrix for HbO and HbR is shown in the top-left part and bottom-right part, respectively. Functional connectivity matrix representing the correlation between HbO and HbR is shown in the top-right part. Plots are symmetric with respect to the main diagonal. Channels are ordered based on their location, from anterior to posterior and from left to right. This allows visualizing an increased correlation between homotopic channels in HbO and HbR (see arrow 1), an increased negative correlation between homotopic channels between HbO and HbR (see arrow 2) and a clear delimitation of left and right hemispheres (see arrow 3).

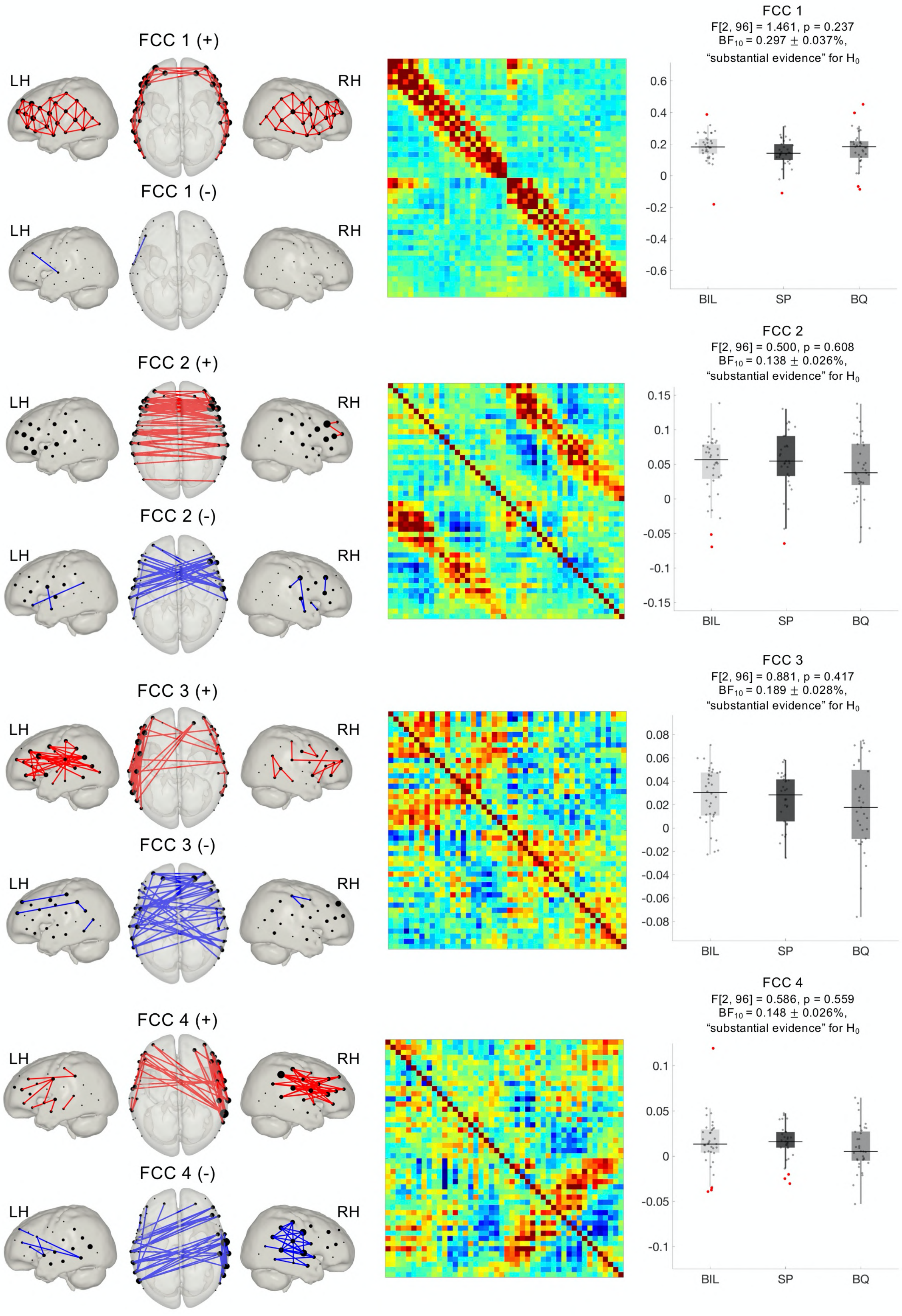

Figure continues in next page

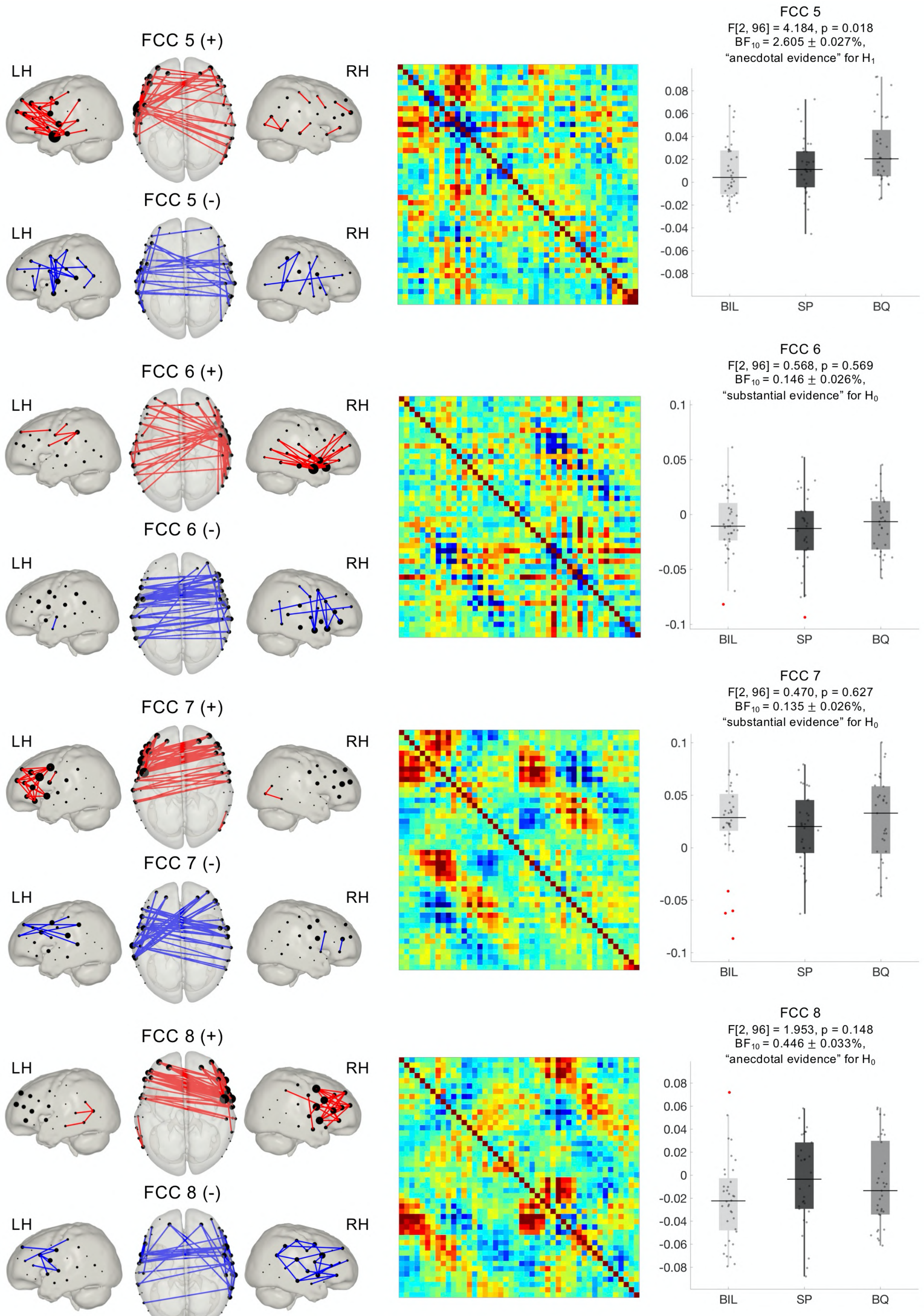

Figure continues in next page

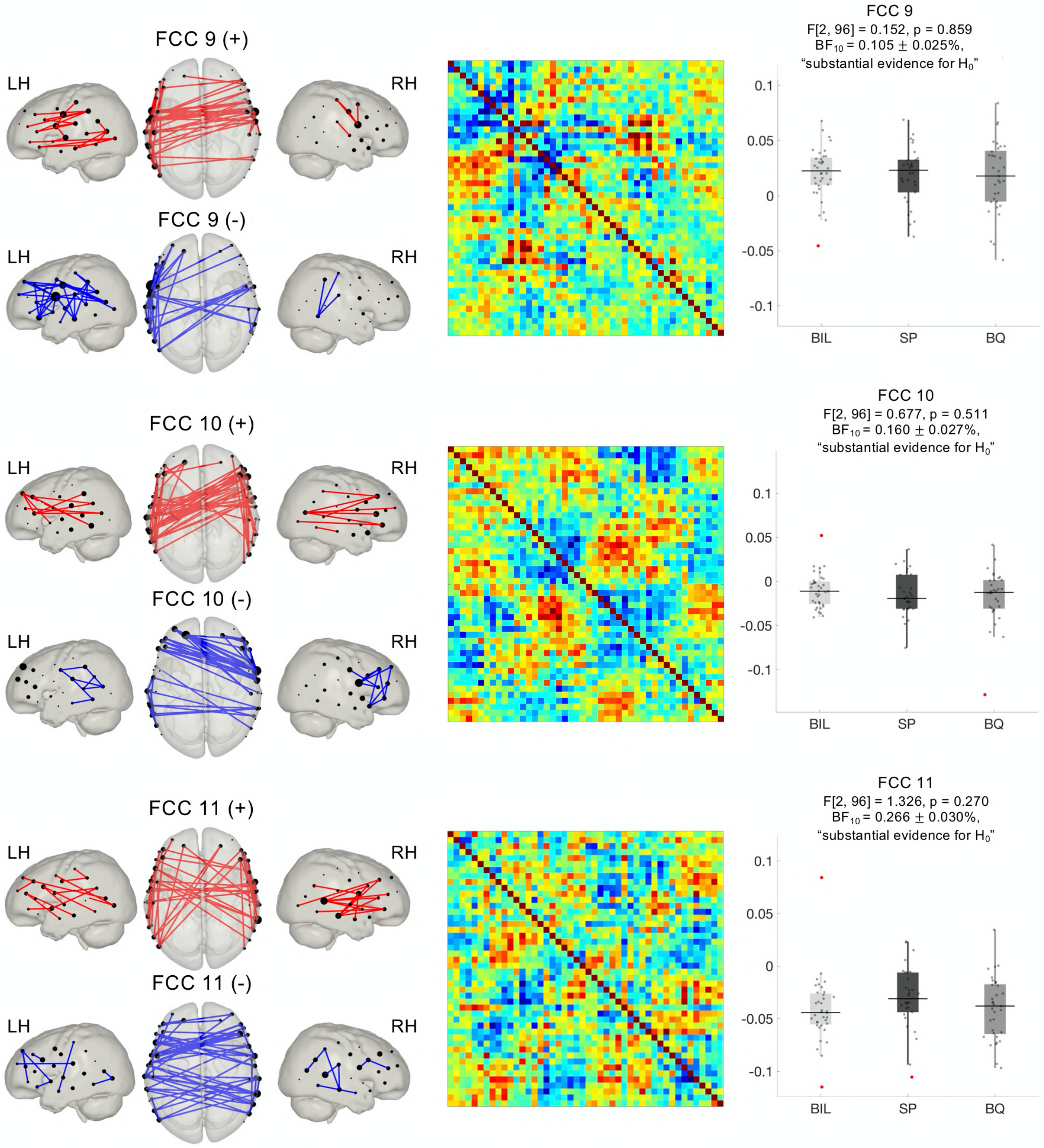

Supplementary Figure 6. Functional connectome components (FCC, positive + and negative -) obtained with the connICA method (n = 11). Leftmost column shows the positive and negative parts of the components which are represented as nodes and edges (top 10% connections) in a brain template. Due to the high similarity between HbO and HbR components, brain maps are shown for HbR only. The middle column shows the components as reconstructed in their original form (i.e., adjacency matrices), with HbO and HbR displayed in the upper and lower triangular sections of the matrix respectively. These matrices represent connectivity values but are not restricted to the Pearson's correlation [-1 1] range **(Amico et al., 2017)**. Statistical comparisons based on frequentist F-test on the weights for each component (right column) revealed no differences between groups (FDR corrected among 11 components, q<0.05). The analysis based on Bayesian statistics showed substantial evidence for H_0 in 9 out of 11 FCC. FCC 5 showed anecdotal evidence for H_1 and FCC 8 demonstrated anecdotal evidence for H_0.

**Funtional Connectome Component (FCC)**	***Iq***	***r***	***ssd (100 %)***	***ssd (60 %)***
FCC 1	0.96	0.95	24	5.5
FCC 2	0.91	0.88	7.2	5.5
FCC 3	0.50	0.70	7.4	5.4
FCC 4	0.94	0.72	7.8	5.4
FCC 5	0.91	0.80	7.3	5.5
FCC 6	0.91	0.83	7.3	5.4
FCC 7	0.90	0.90	8.8	5.6
FCC 8	0.84	0.84	7.3	5.4
FCC 9	0.88	0.72	7.6	5.5
FCC 10	0.90	0.74	8.0	5.4
FCC 11	0.71	0.67	7.6	5.4

Supplementary Table 3. Model order evaluation metrics for the PCA threshold selected in the connICA approach (i.e., 60% - 11 independent components). Sum of squared differences (ssd) are computed with respect to the data after PCA (total = 100%) and with respect to the original data without PCA (total = 60%). Iq = *ICASSO* cluster quality index; r = correlation coefficient between HbO and HbR components.

References

Amico E, Marinazzo D, Di Perri C, Heine L, Annen J, Martial C, Dzemidzic M, Kirsch M, Bonhomme V, Laureys S, Goñi J. 2017. Mapping the functional connectome traits of levels of consciousness. *Neuroimage,* 148, 201-211. https://doi.org/10.1016/j.neuroimage.2017.01.020.

Damaraju E, Caprihan A, Lowe JR, Allen EA, Calhoun VD, Phillips JP. 2014. Functional connectivity in the developing brain: a longitudinal study from 4 to 9 months of age. *Neuroimage,* 84, 169–80. https://doi.org/10.1016/j.neuroimage.2013.08.038.

Himberg J, Hyvärinen A, Esposito F. 2004. Validating the independent components of neuroimaging time series via clustering and visualization. *Neuroimage,* 22(3), 1214-1222. https://doi.org/10.1016/j.neuroimage.2004.03.027.

Homae F, Watanabe H, Otobe T, Nakano T, Go T, Konishi Y, Taga G. 2010. Development of global cortical networks in early infancy. *J. Neurosci.* 30(14), 4877–4882. https://doi.org/10.1523/JNEUROSCI.5618-09.2010.

Keysers C, Gazzola V, Wagenmakers E. 2020. Using Bayes factor hypothesis testing in neuroscience to establish evidence of absence. *Nat Neurosci* 23, 788–799. https://doi.org/10.1038/s41593-020-0660-4.

Minka TP. 2001. Automatic choice of dimensionality for PCA. Adv*. Neural Inf. Process Syst*. 598-604.

Morey RD, Rouder JN, Jamil T, Urbanek S, Forner K, Ly A, 2018. BayesFactor.

Obrig H, Villringer A. 2003. Beyond the visible - Imaging the human brain with light. *J. Cereb. Blood Flow Metab.* 23(1), 1–18. https://doi.org/10.1097/01.WCB.0000043472.45775.29.

Villringer A, Chance B. 1997. Non-invasive optical spectroscopy and imaging of human brain function. *Trends Neurosci*. 20(10), 435-442. https://doi.org/10.1016/S0166-2236(97)01132-6.

Watanabe H, Shitara Y, Aoki Y, Inoue T, Tsuchida S, Takahashi N, Taga G. 2017. Hemoglobin phase of oxygenation and deoxygenation in early brain development measured using fNIRS. *Proc. Natl. Acad. Sci. USA,* 114(9), E1737–E1744. https://doi.org/10.1073/pnas.1616866114.

Wetzels R, Wagenmakers EJ. 2012. A default Bayesian hypothesis test for correlations and partial correlations. *Psychon. Bull. Rev.* 19(6), 1057-1064. https://doi.org/10.3758/s13423-012-0295-x.

Wolf M, Wolf U, Toronov V, Michalos A, Paunescu LA, Choi JH, Gratton E. 2002. Different time evolution of oxyhemoglobin and deoxyhemoglobin concentration changes in the visual and motor cortices during functional stimulation: a near-infrared spectroscopy study. *Neuroimage,* 16(3), 704-712. https://doi.org/10.1006/nimg.2002.1128.

Data quality assessment figures

For each participant (n=99), two figures comprising various plots of the different steps of our data quality assessment routine are displayed in this supplementary material. The last five figures on Pages 215-219, represent the time series for the five datasets that were discarded due to insufficient data qualty. A brief explanation of the figures is provided below.

Figure 1

This figure presents the time series of the complete recording (560 seconds) for the two wavelengths (760 and 850 nm) used by our fNIRS system.

Top row: Raw intensity data.
Middle row: Optical density data after conversion from raw intensity data.
Bottom row: Optical density data after artifact correction using the wavelet-based despike method (Patel et al., 2014).

Figure 2

This figure shows various data quality assessment plots at different preprocessing steps. Rows 1 and 2 display the HbO and HbR time series. The third row shows the power spectral density of HbO and HbR concentration data at each preprocessing step. In the bottom row, and for each column (i.e., preprocessing step), leftmost plot shows the functional connectivity matrix. In this plot, the functional connectivity matrix for HbO and HbR is shown in the top-left part and bottom- right part, respectively. The functional connectivity matrix representing the correlation between HbO and HbR is shown in the top-right part (expected negative). Note that these matrices are symmetric with respect to the main diagonal. For preprocessed data, after filtering and global signal regression, the standard functional connectivity matrix and the functional connectivity matrix computed using robust regression (Santosa et al., 2017) are presented. The histogram plots in polar coordinates presented on the right part show the phase difference between HbO and HbR time-series (hPod value, Watanabe et al., 2017) at each preprocessing step.

Left column: raw HbO and HbR data.
Middle column: HbO and HbR time series after filtering.
Right column: HbO and HbR preprocessed data, after filtering and global signal regression.

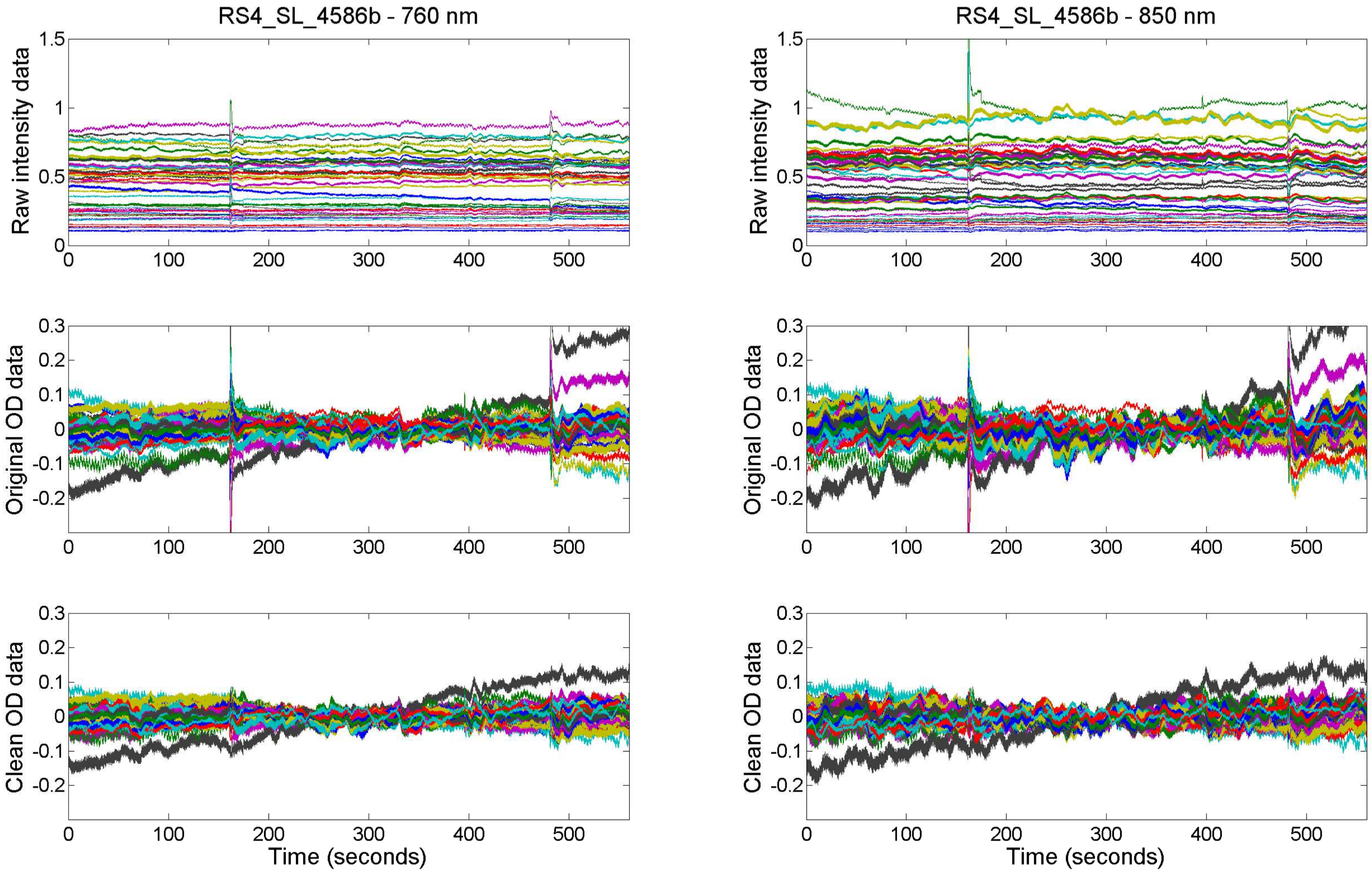

RS4_SL_4586b - 760 nm
RS4_SL_4586b - 850 nm
Raw intensity data
Original OD data
Clean OD data
Time (seconds)

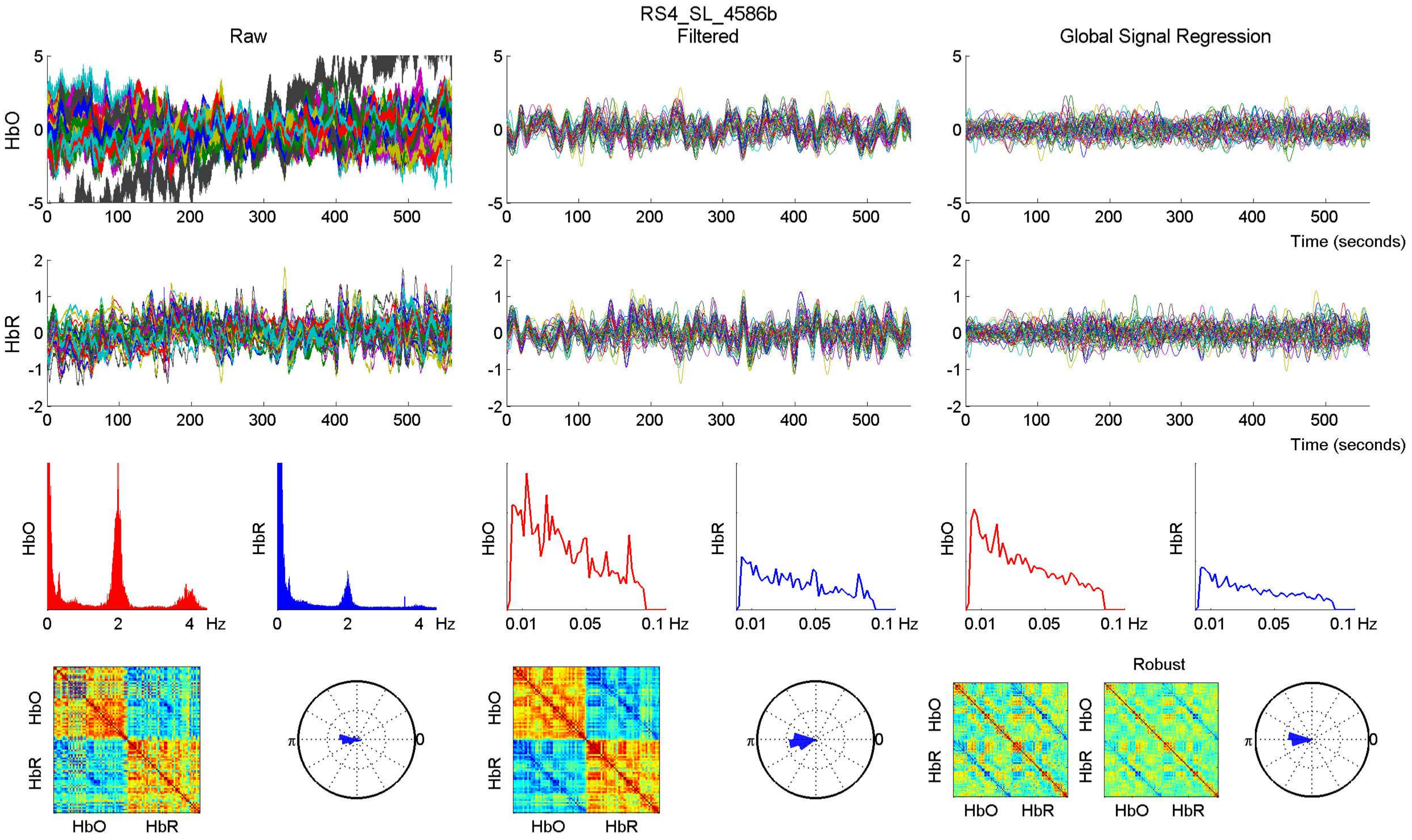
RS4_SL_4586b
Raw
Filtered
Global Signal Regression
HbO
HbR
Time (seconds)
Hz
Robust

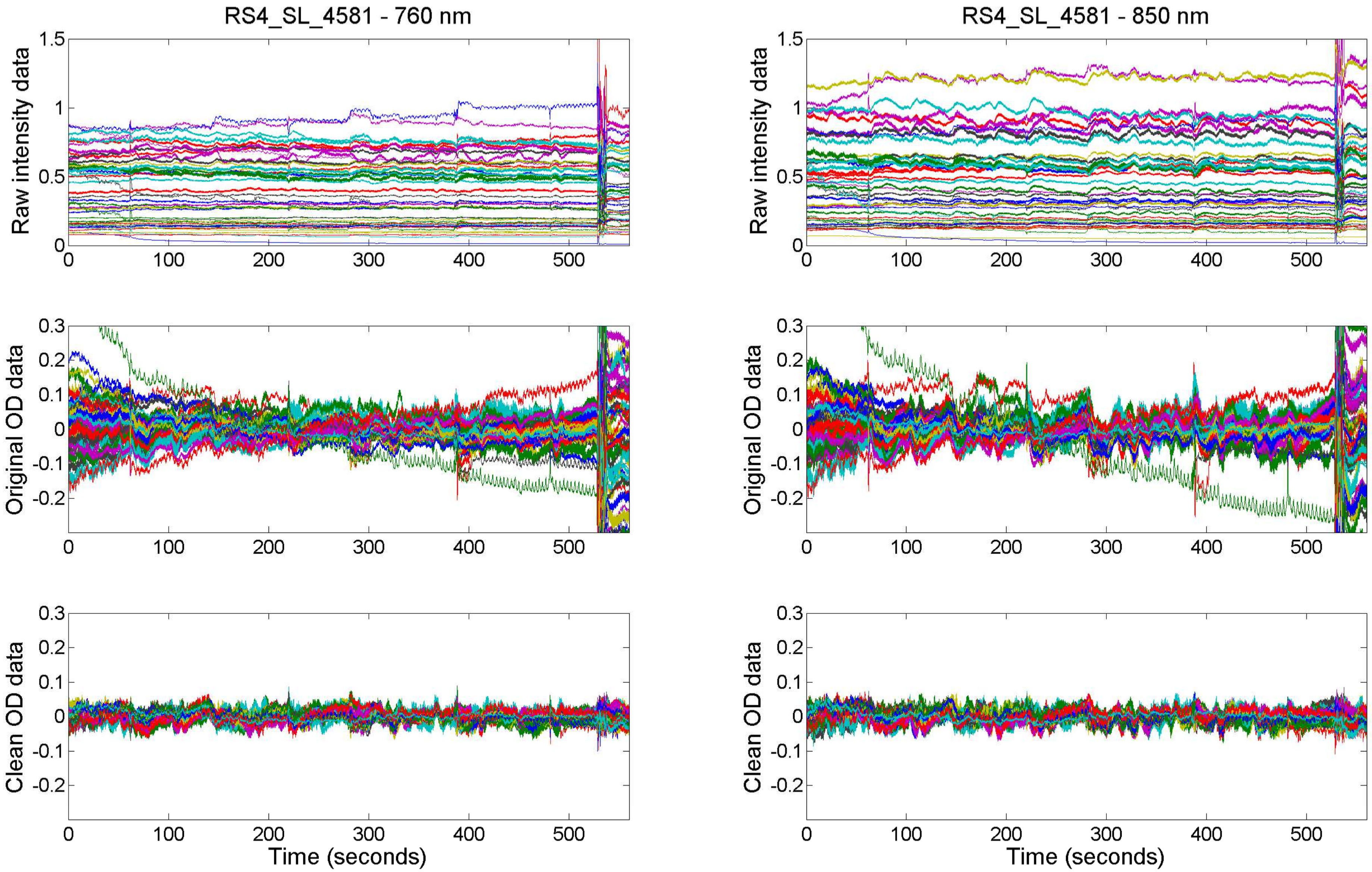
RS4_SL_4581 - 760 nm
RS4_SL_4581 - 850 nm
Raw intensity data
Original OD data
Clean OD data
Time (seconds)
0
100
200
300
400
500
1.5
1
0.5
0.3
0.2
0.1
-0.1
-0.2

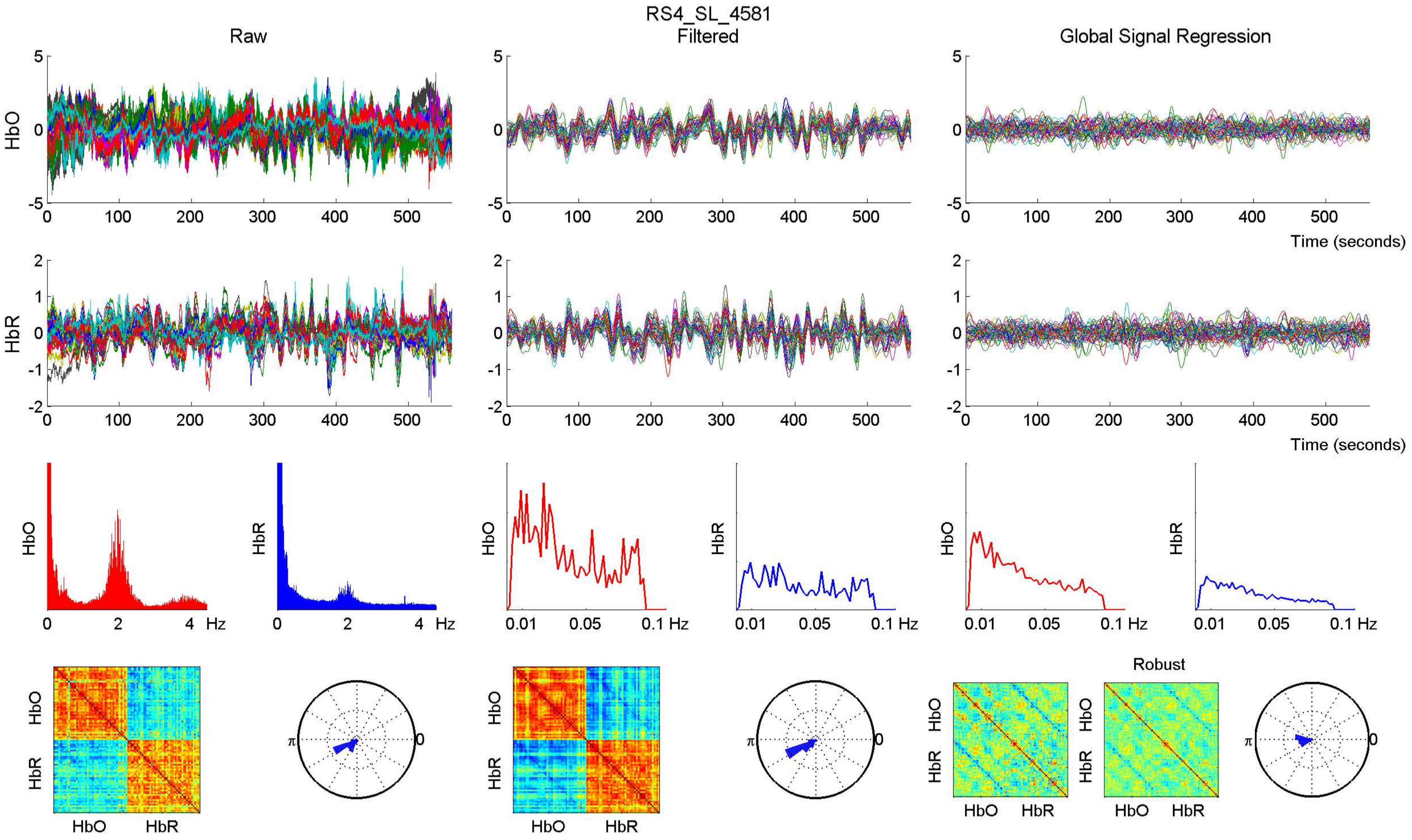

RS4_SL_4581
Raw
Filtered
Global Signal Regression
HbO
HbR
Time (seconds)
Hz
0.01
0.05
0.1 Hz
Robust
HbO
HbR
π
0

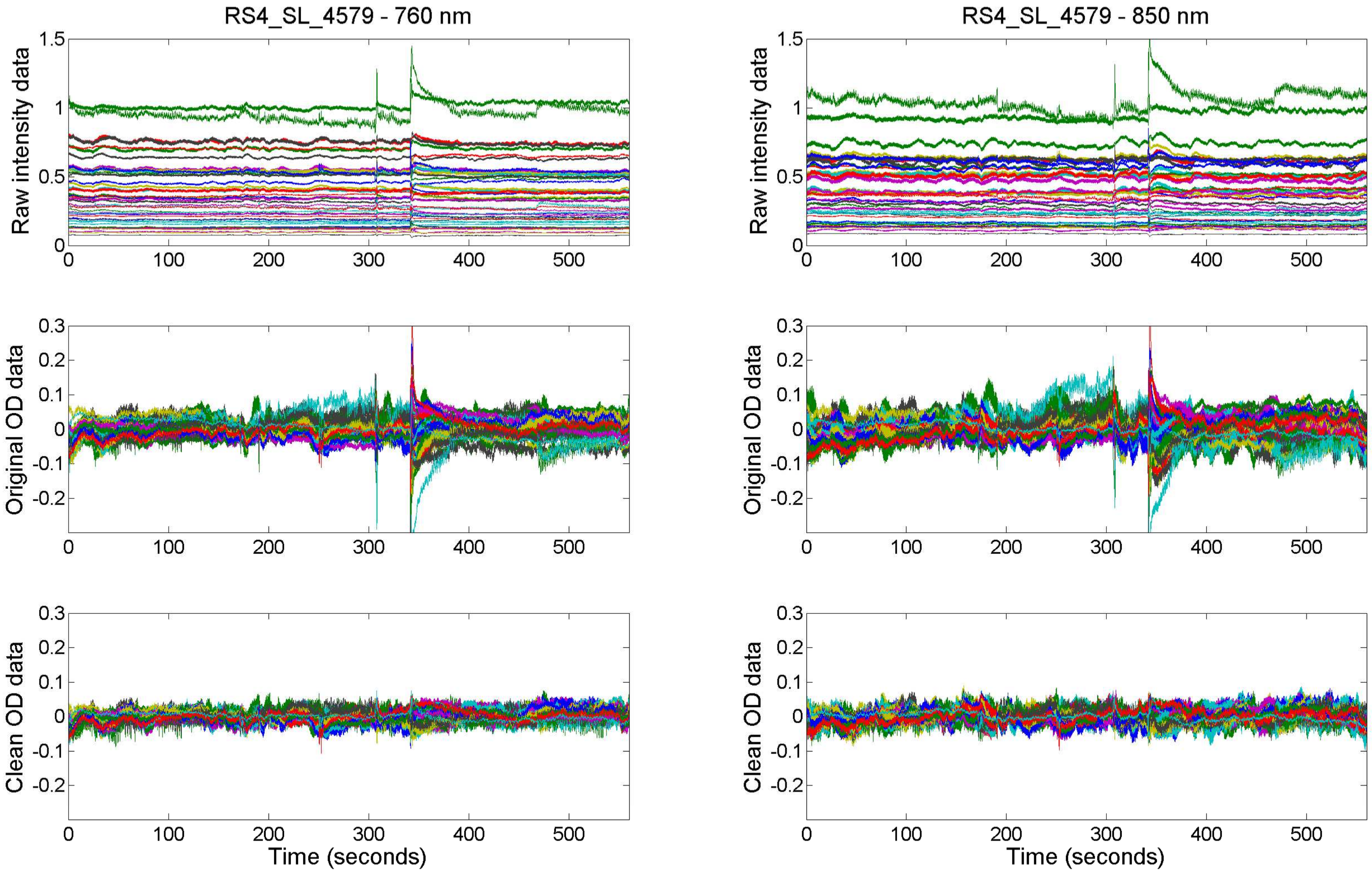
RS4_SL_4579 - 760 nm
RS4_SL_4579 - 850 nm
Raw intensity data
Original OD data
Clean OD data
Time (seconds)
0
100
200
300
400
500
1.5
1
0.5
0.3
0.2
0.1
-0.1
-0.2

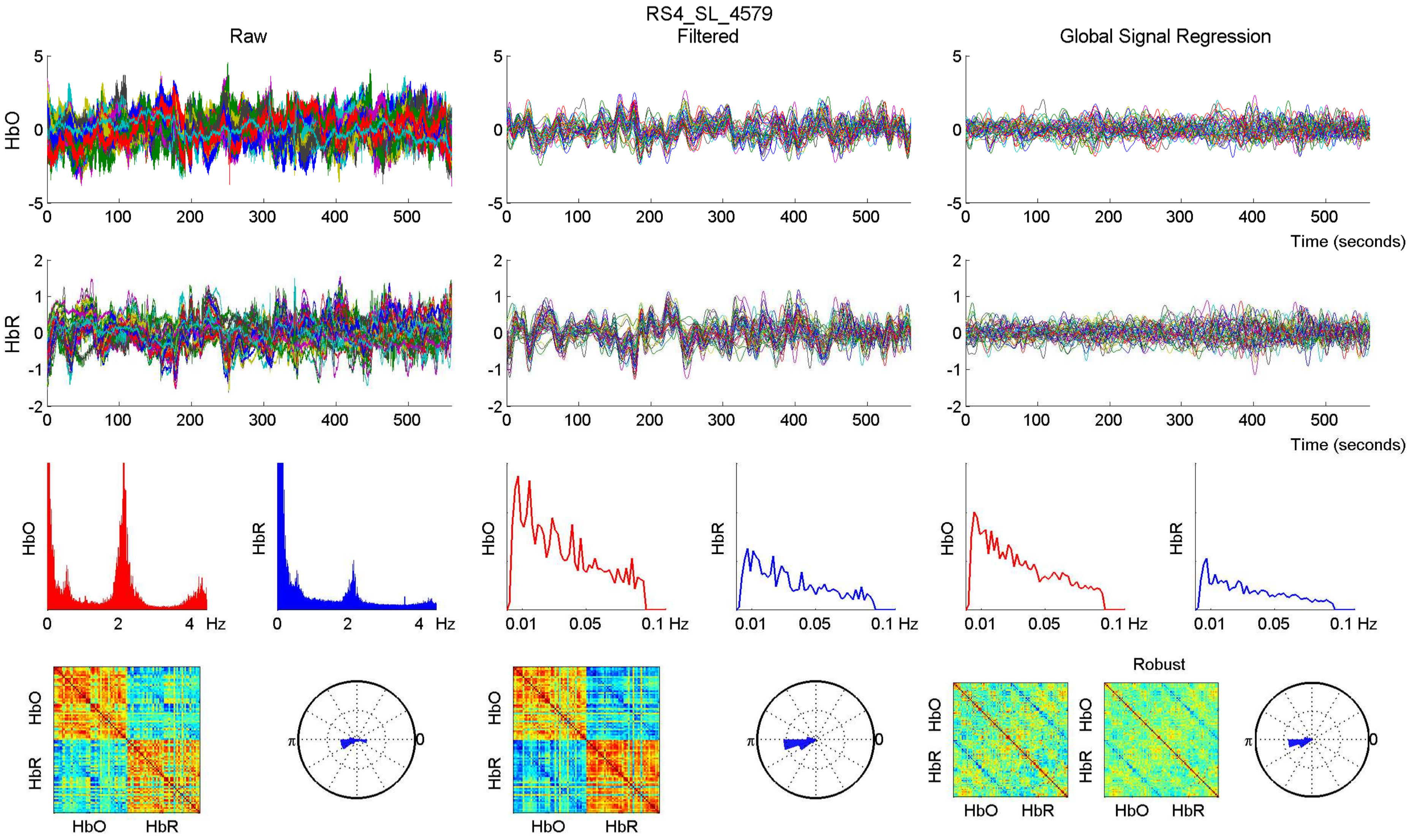
RS4_SL_4579
Raw
Filtered
Global Signal Regression
HbO
HbR
Time (seconds)
Hz
Robust
HbO
HbR

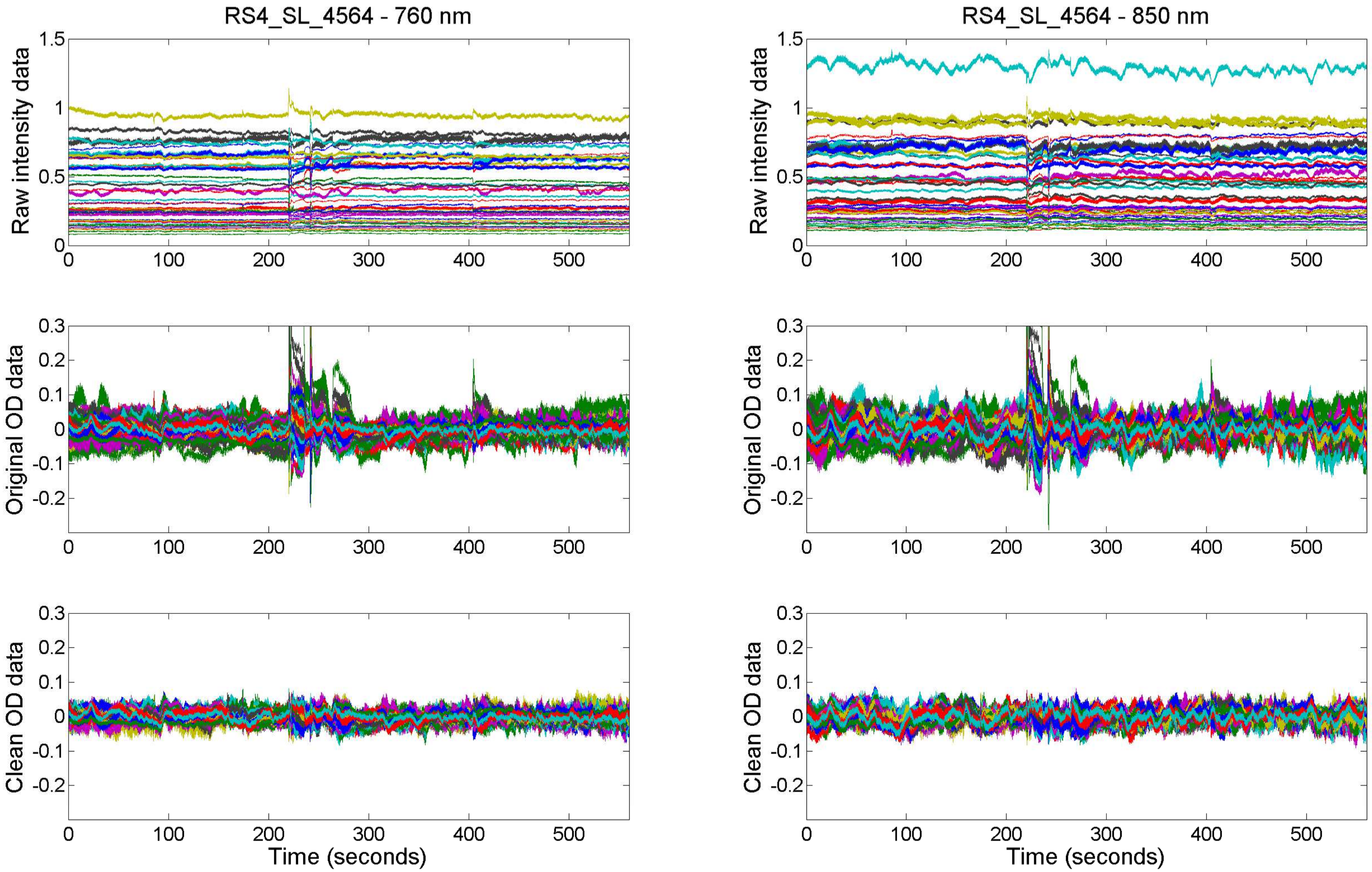
RS4_SL_4564 - 760 nm
RS4_SL_4564 - 850 nm
Raw intensity data
Original OD data
Clean OD data
Time (seconds)
Time (seconds)

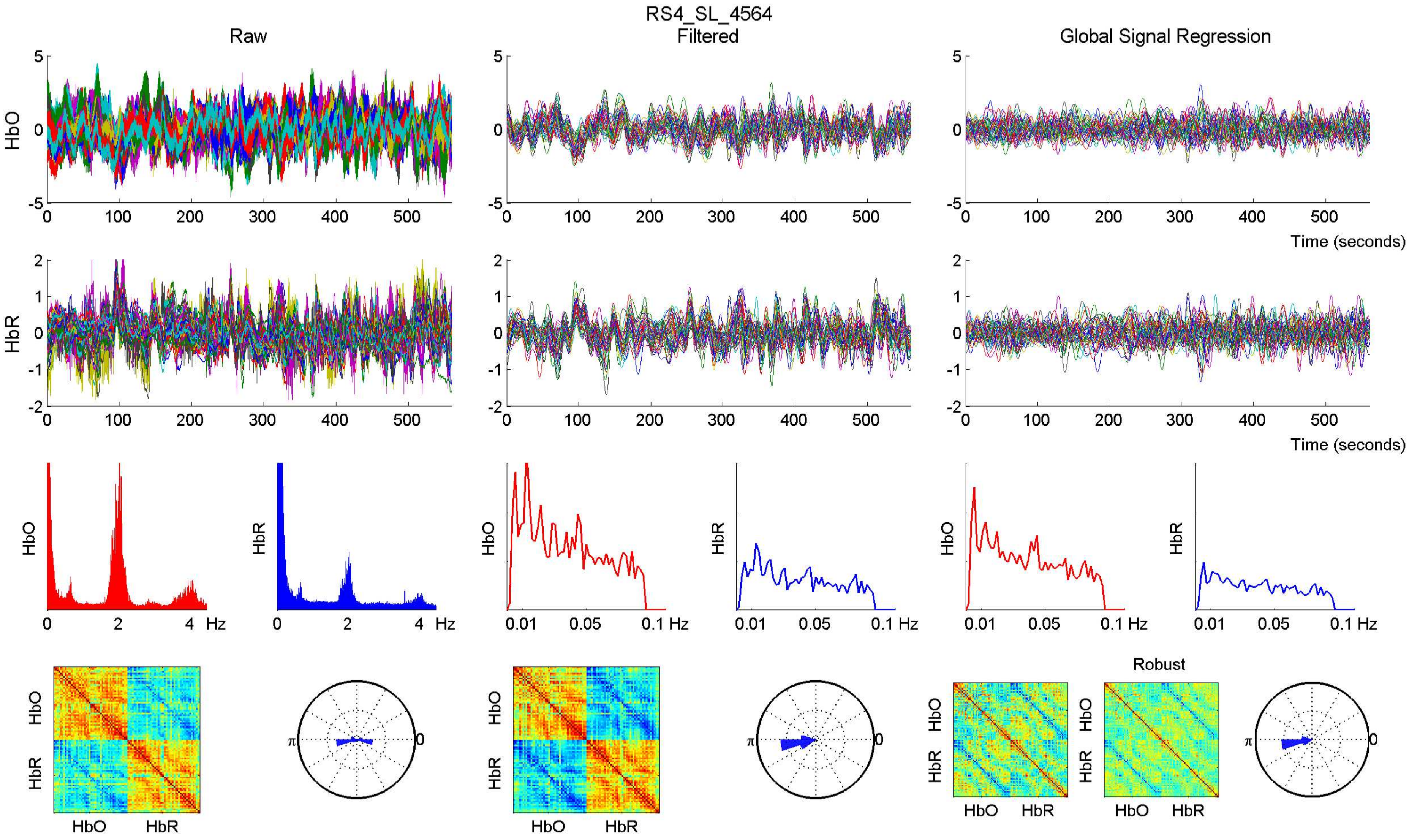
RS4_SL_4564
Raw
Filtered
Global Signal Regression
HbO
HbR
Time (seconds)
Hz
Robust
HbO HbR

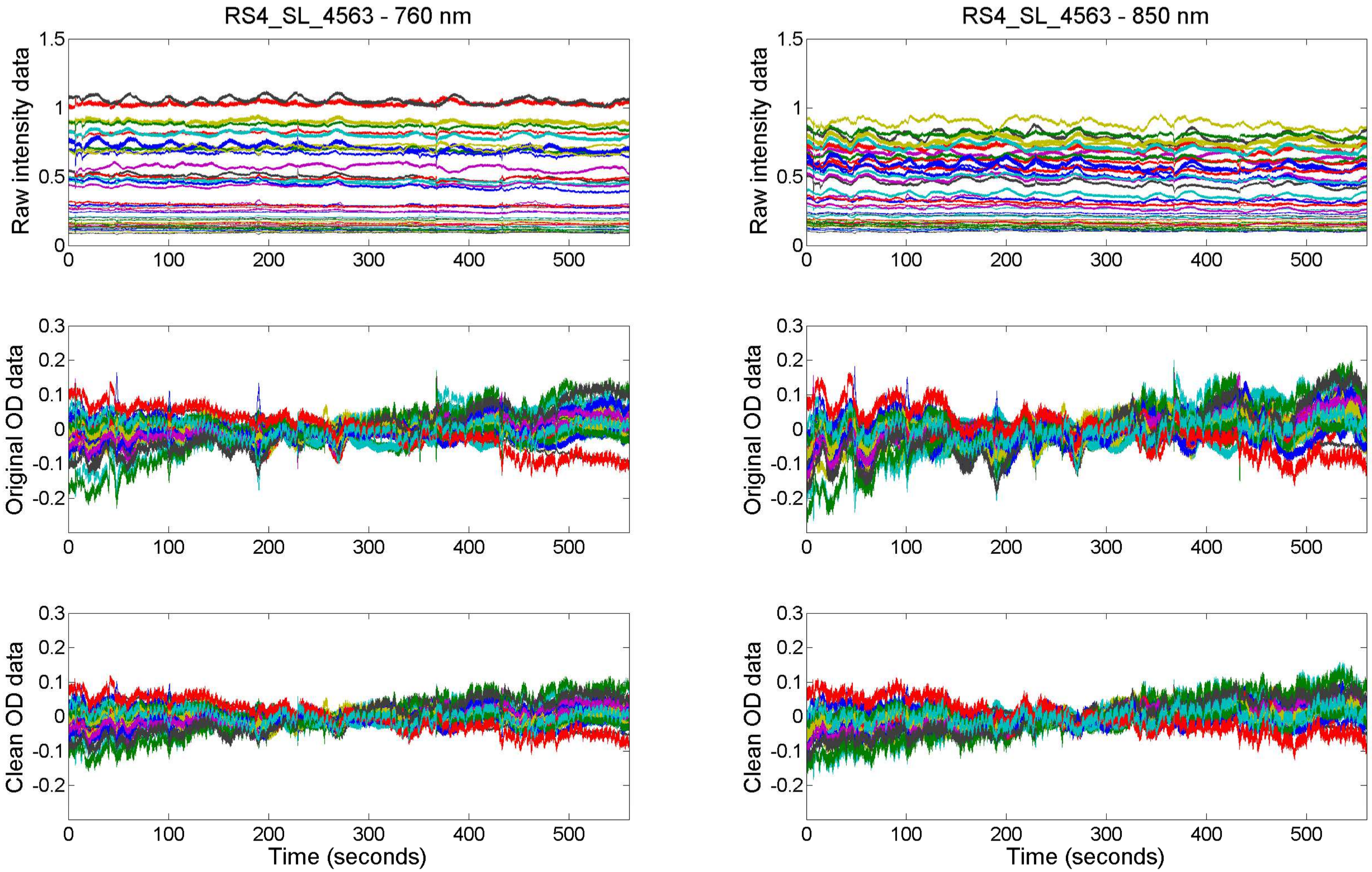
RS4_SL_4563 - 760 nm
RS4_SL_4563 - 850 nm
Raw intensity data
Original OD data
Clean OD data
Time (seconds)
Time (seconds)

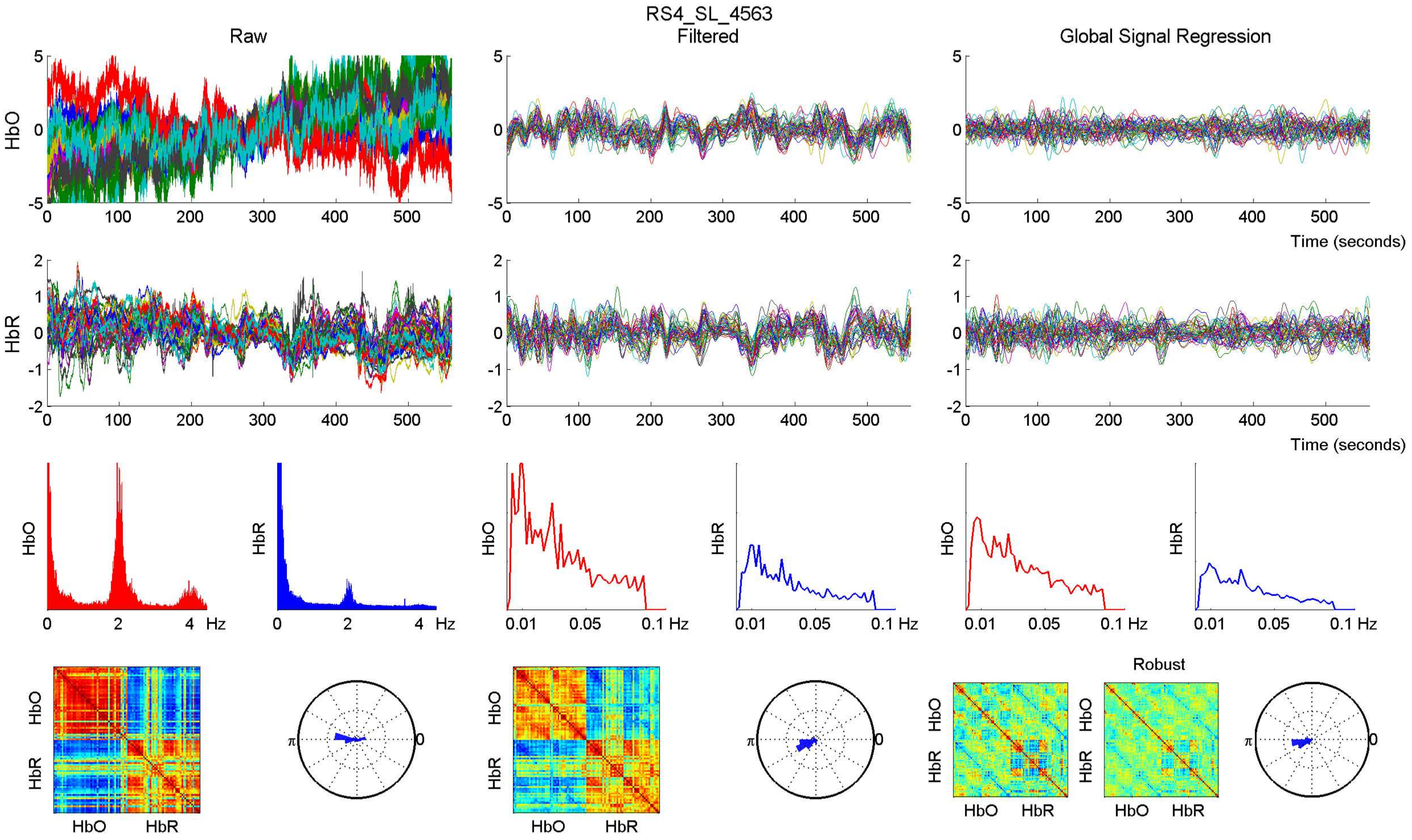

RS4_SL_4563
Raw
Filtered
Global Signal Regression
HbO
HbR
Time (seconds)
Hz
Robust

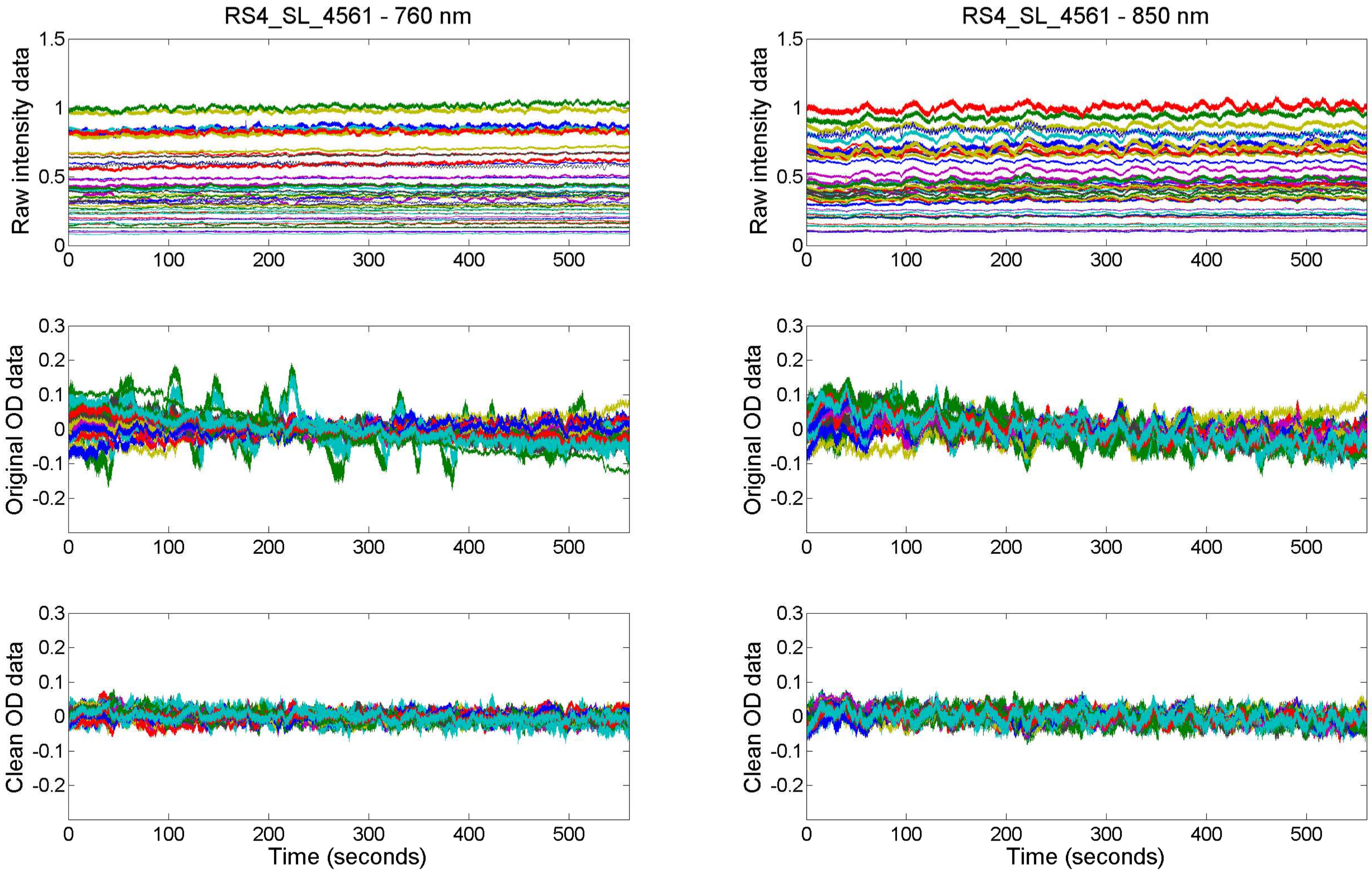
RS4_SL_4561 - 760 nm
RS4_SL_4561 - 850 nm
Raw intensity data
Original OD data
Clean OD data
Time (seconds)

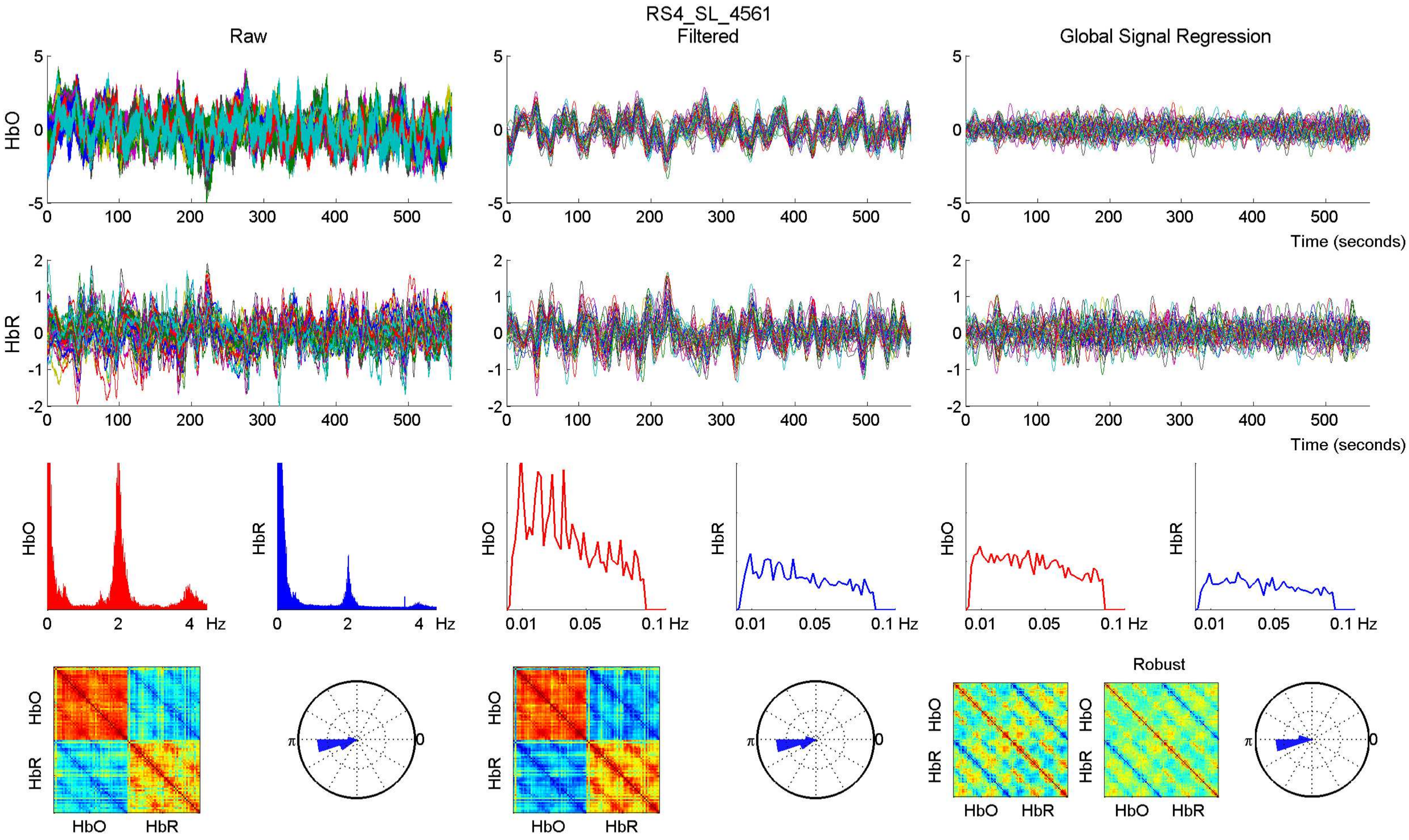

RS4_SL_4561
Raw
Filtered
Global Signal Regression
HbO
HbR
Time (seconds)
Hz
Robust
π
0

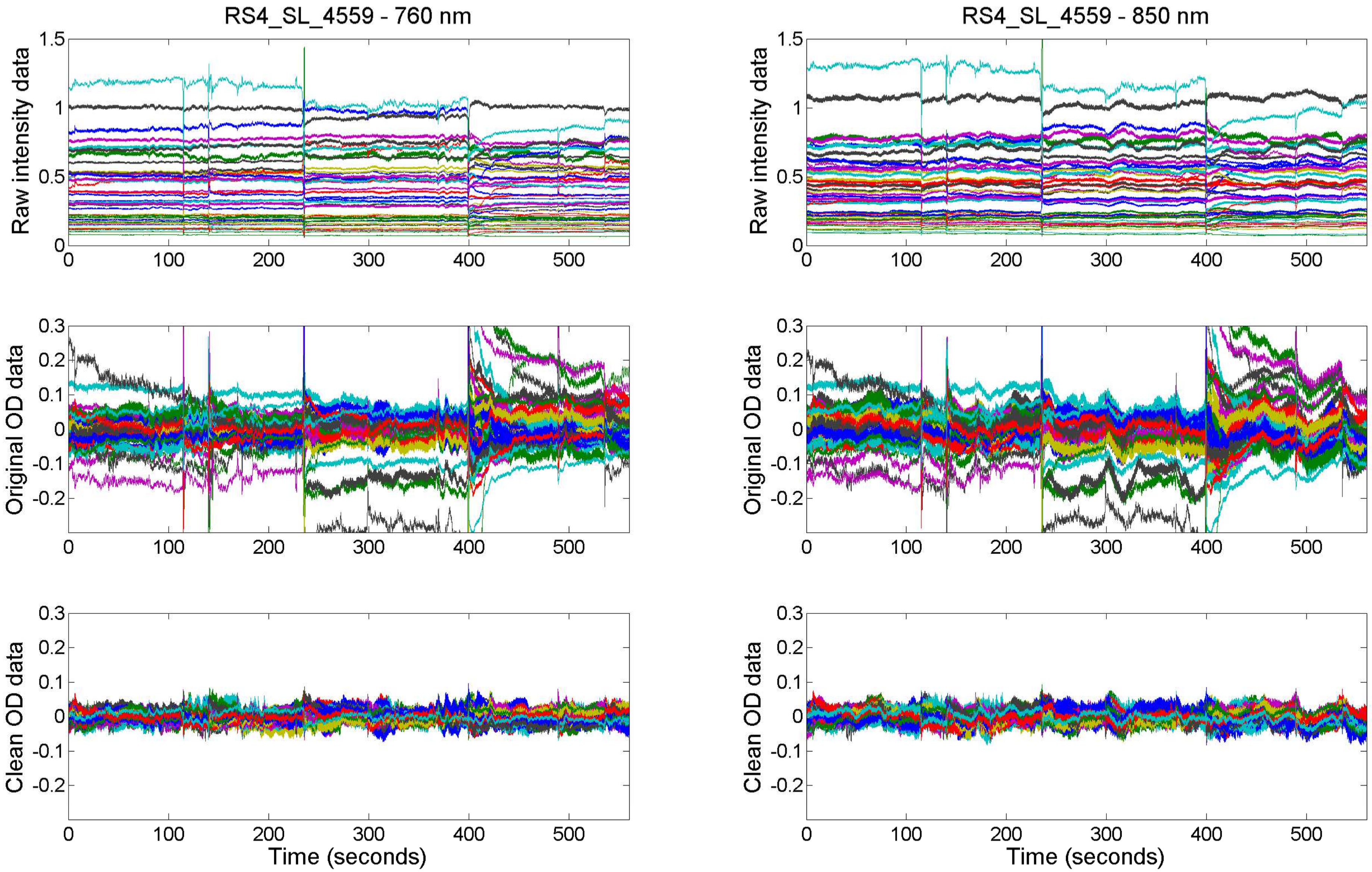

RS4_SL_4559 - 760 nm
RS4_SL_4559 - 850 nm
Raw intensity data
Original OD data
Clean OD data
Time (seconds)

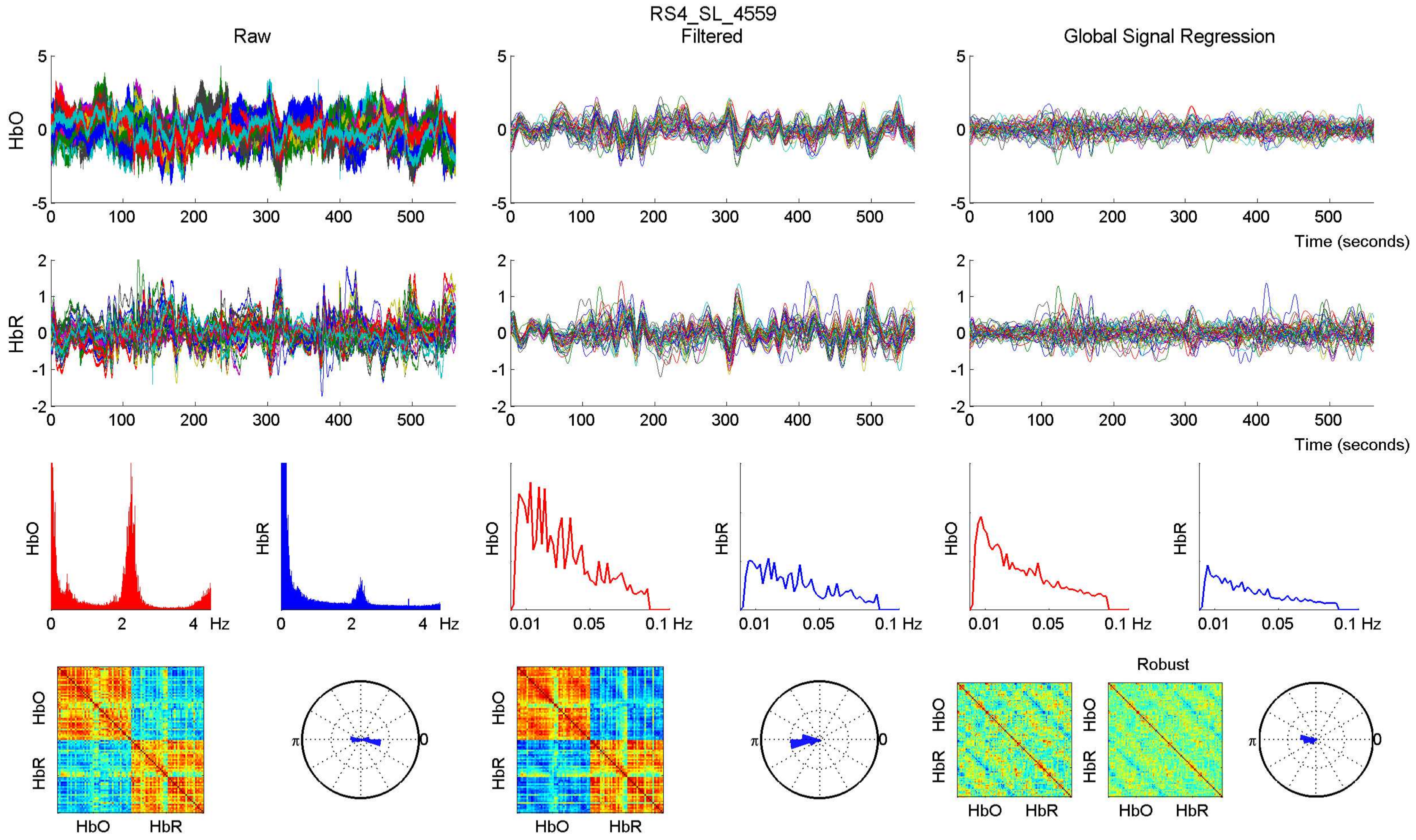
RS4_SL_4559
Raw
Filtered
Global Signal Regression
HbO
HbR
Time (seconds)
Hz
0.01
0.05
0.1 Hz
Robust
π
0

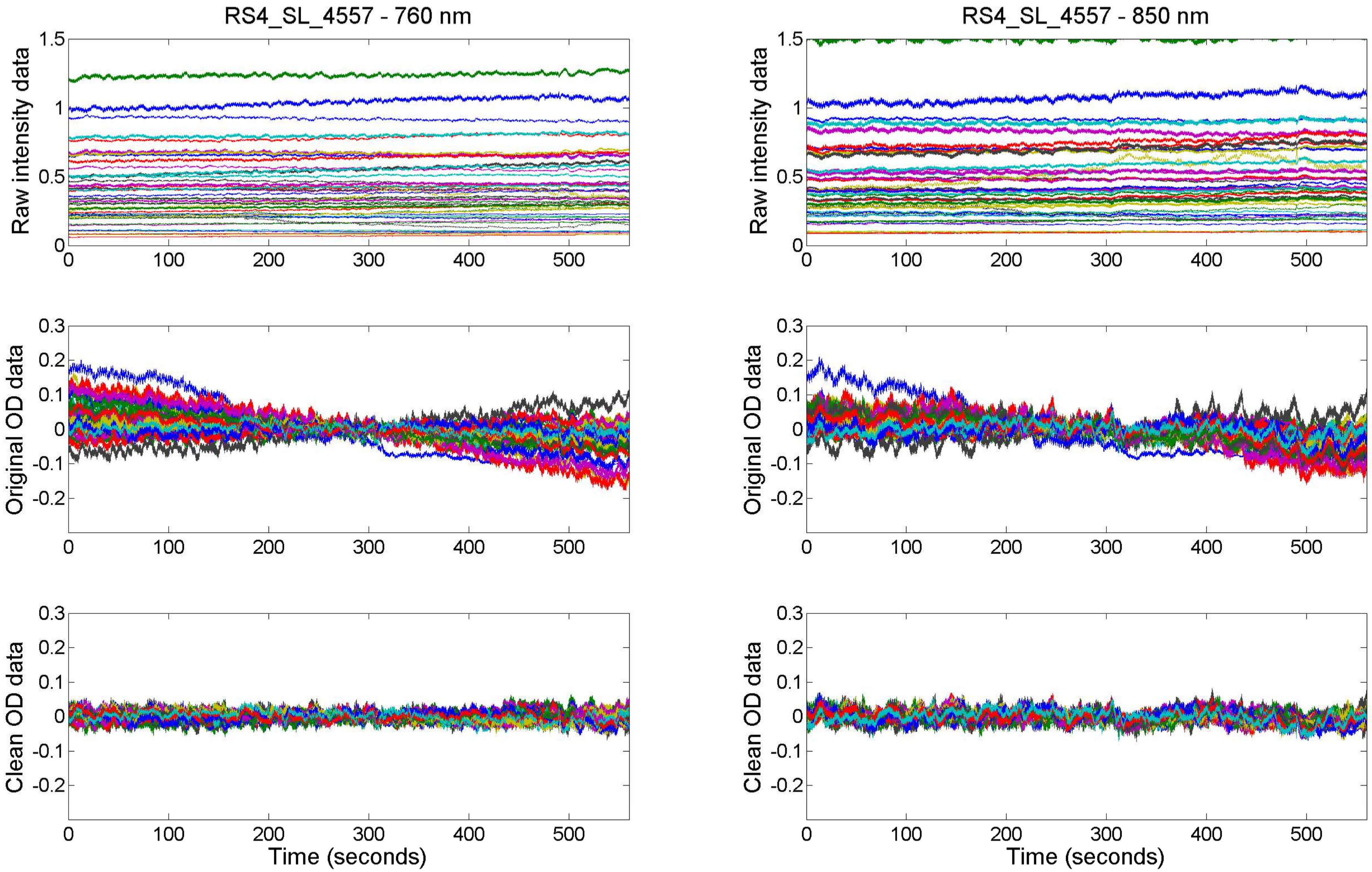

RS4_SL_4557 - 760 nm
RS4_SL_4557 - 850 nm
Raw intensity data
Original OD data
Clean OD data
Time (seconds)

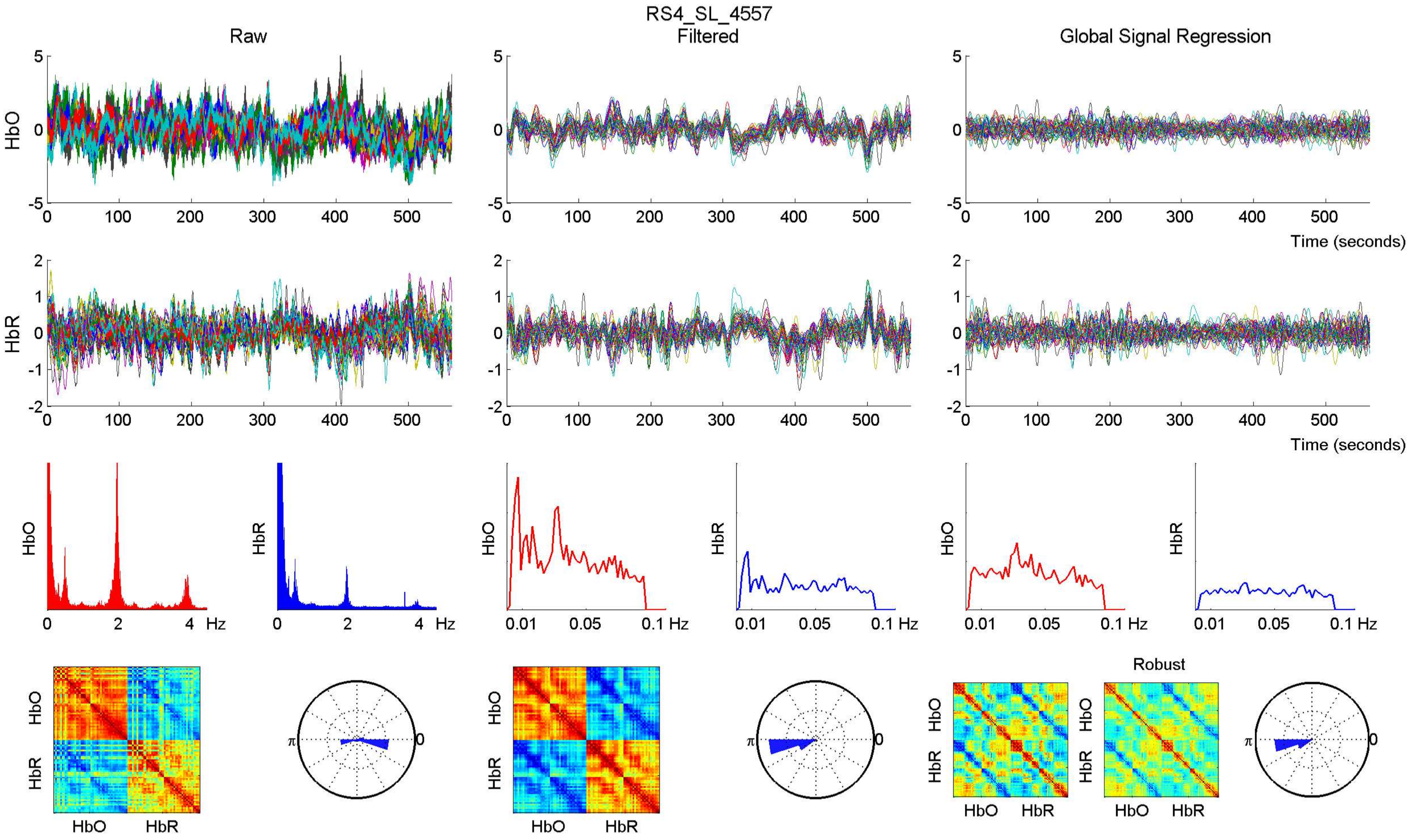

RS4_SL_4557
Raw
Filtered
Global Signal Regression
HbO
HbR
Time (seconds)
Hz
Robust

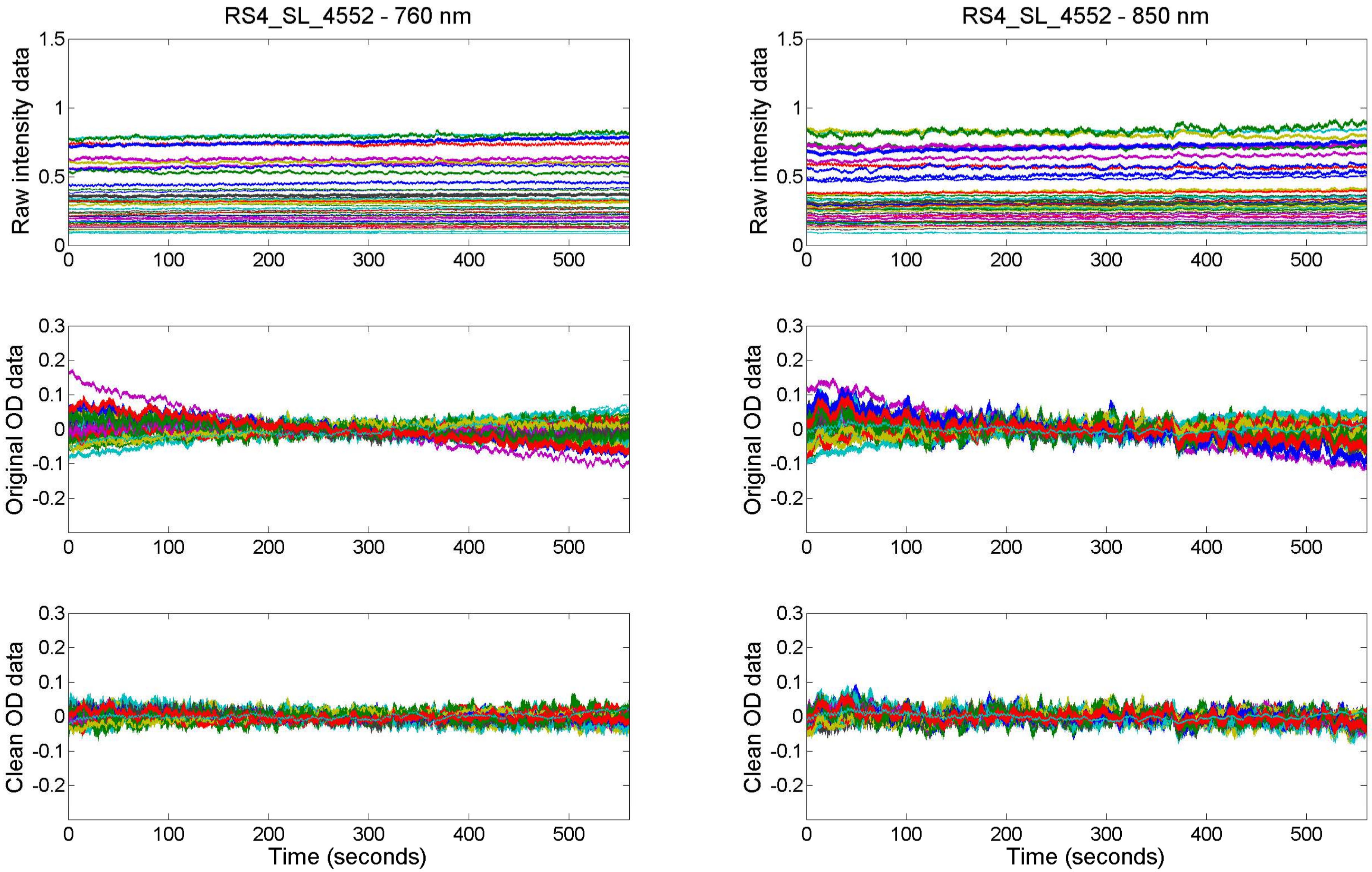
RS4_SL_4552 - 760 nm
RS4_SL_4552 - 850 nm
Raw intensity data
Original OD data
Clean OD data
Time (seconds)
0
100
200
300
400
500
1.5
1
0.5
0.3
0.2
0.1
-0.1
-0.2

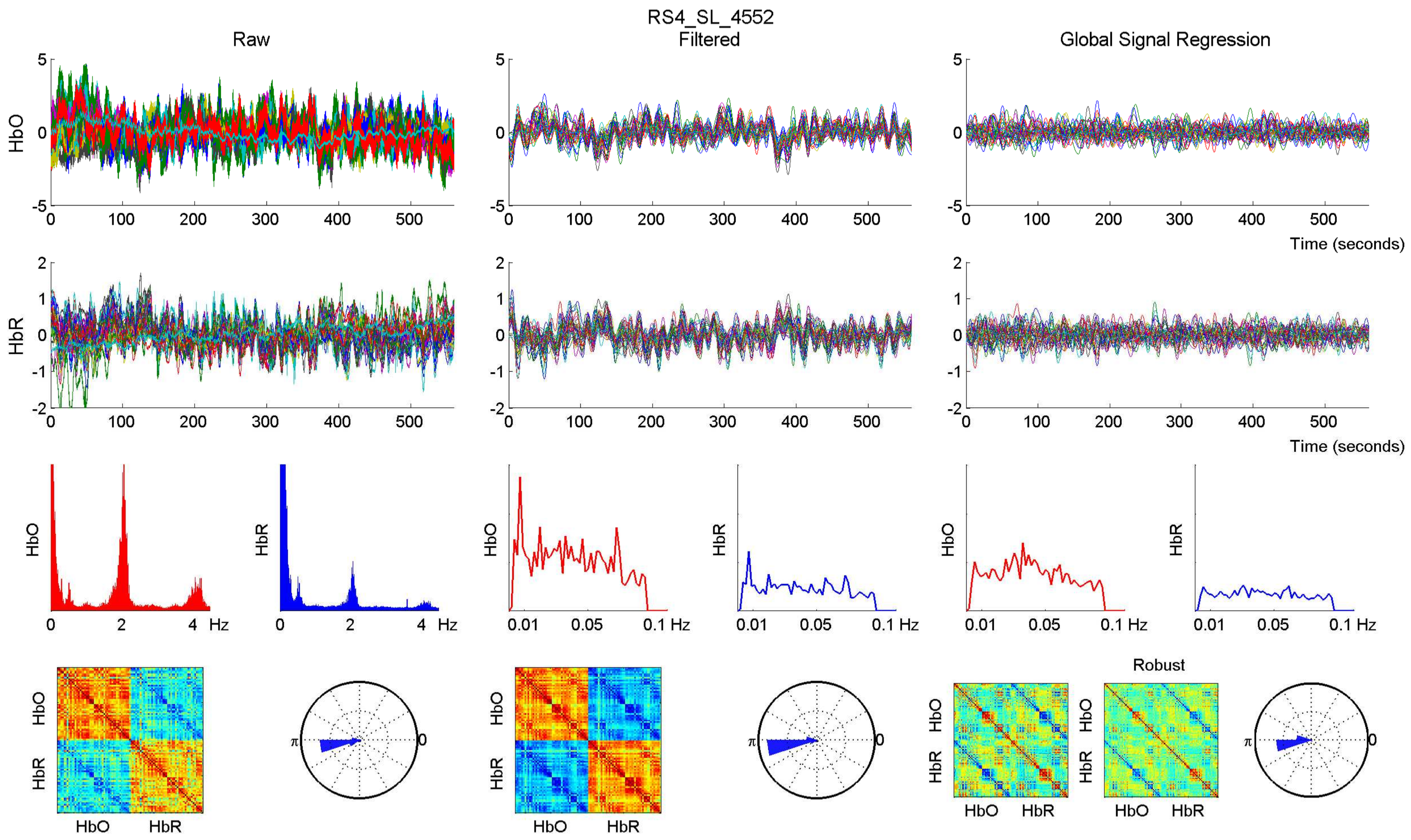

RS4_SL_4552
Raw
Filtered
Global Signal Regression
HbO
HbR
Time (seconds)
Hz
Robust

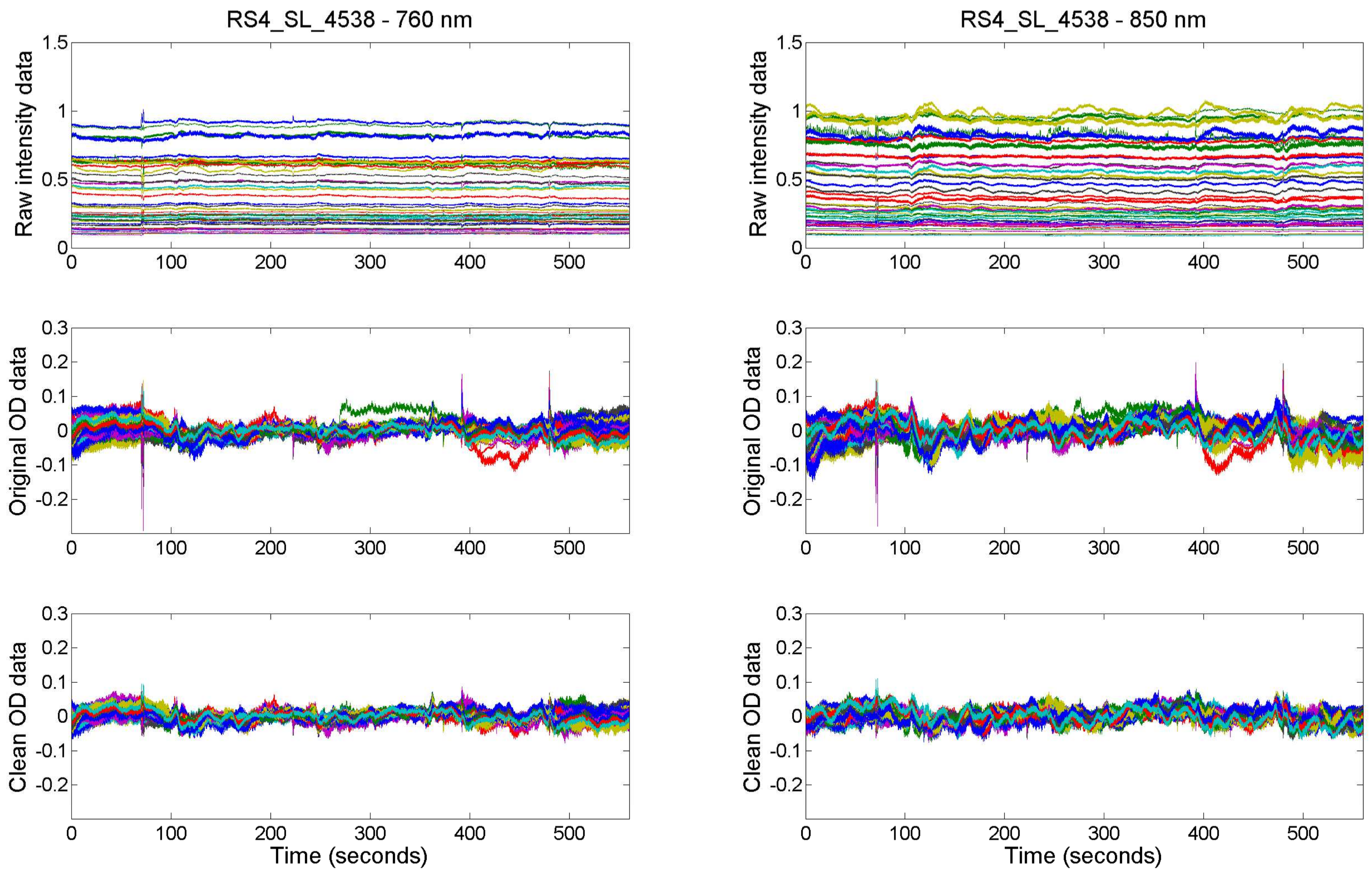
RS4_SL_4538 - 760 nm
RS4_SL_4538 - 850 nm
Raw intensity data
Original OD data
Clean OD data
Time (seconds)
1.5
1
0.5
0
0.3
0.2
0.1
-0.1
-0.2
100
200
300
400
500

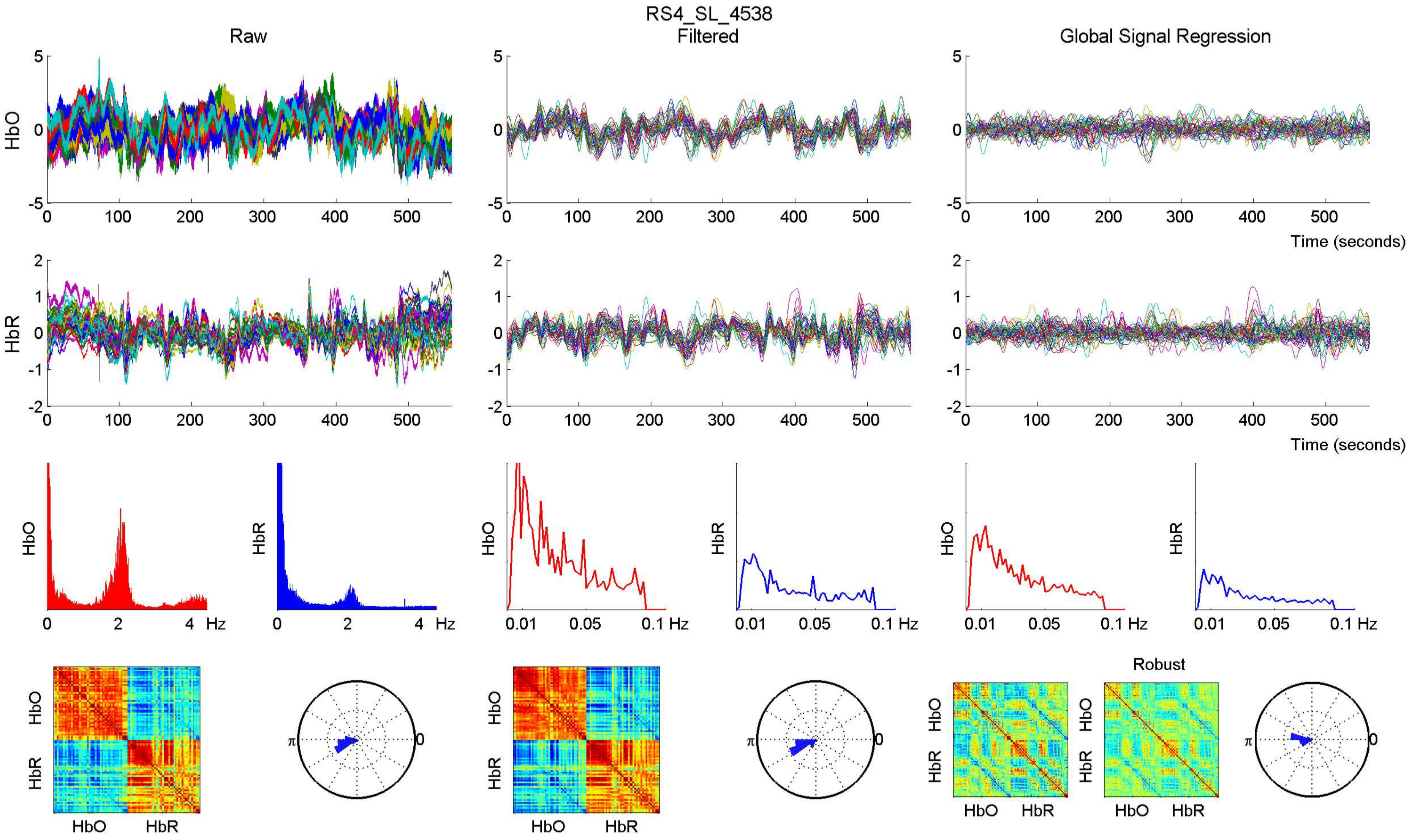

RS4_SL_4538
Raw
Filtered
Global Signal Regression
HbO
HbR
Time (seconds)
Hz
Robust

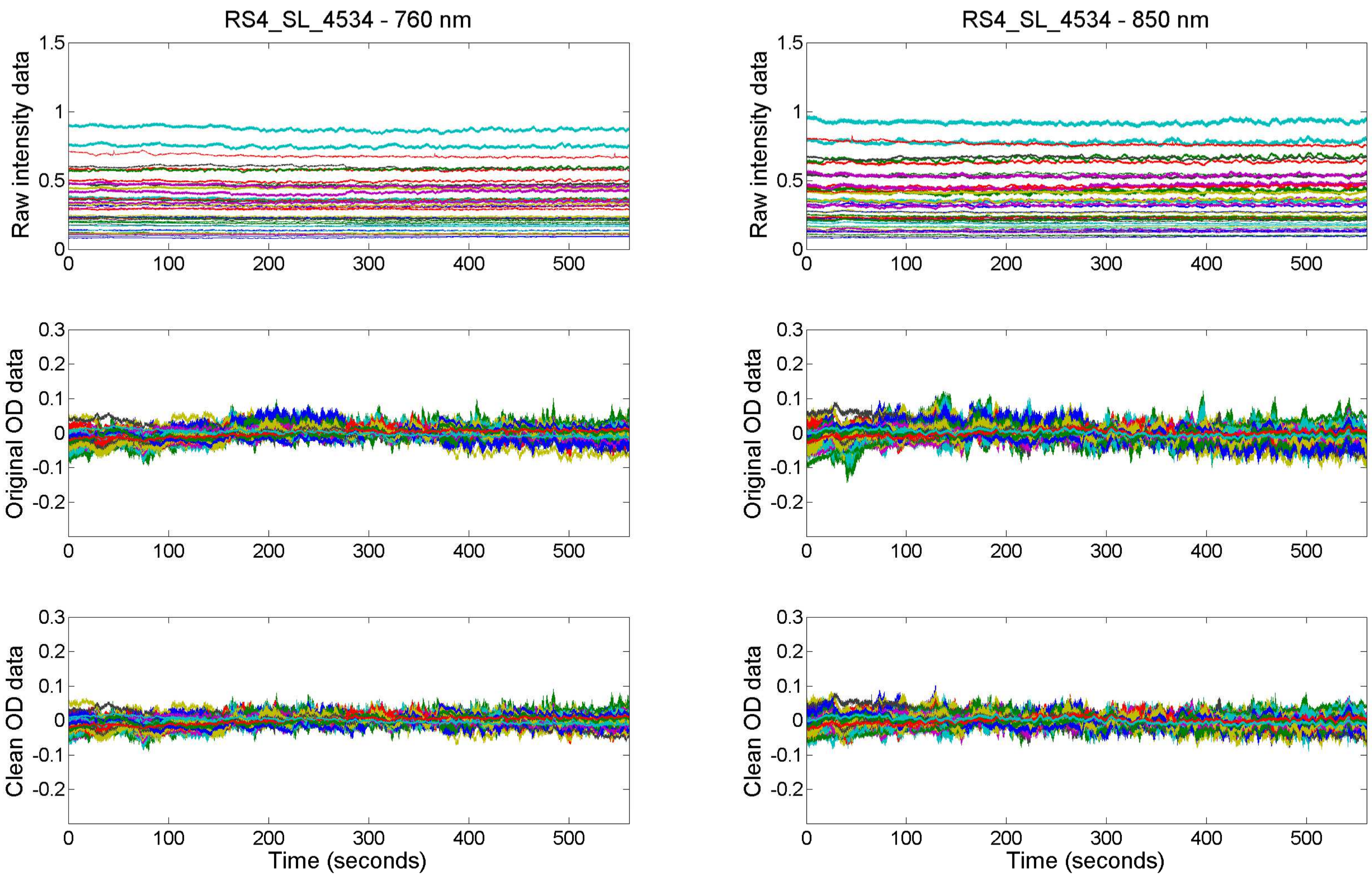
RS4_SL_4534 - 760 nm
RS4_SL_4534 - 850 nm
Raw intensity data
Original OD data
Clean OD data
Time (seconds)

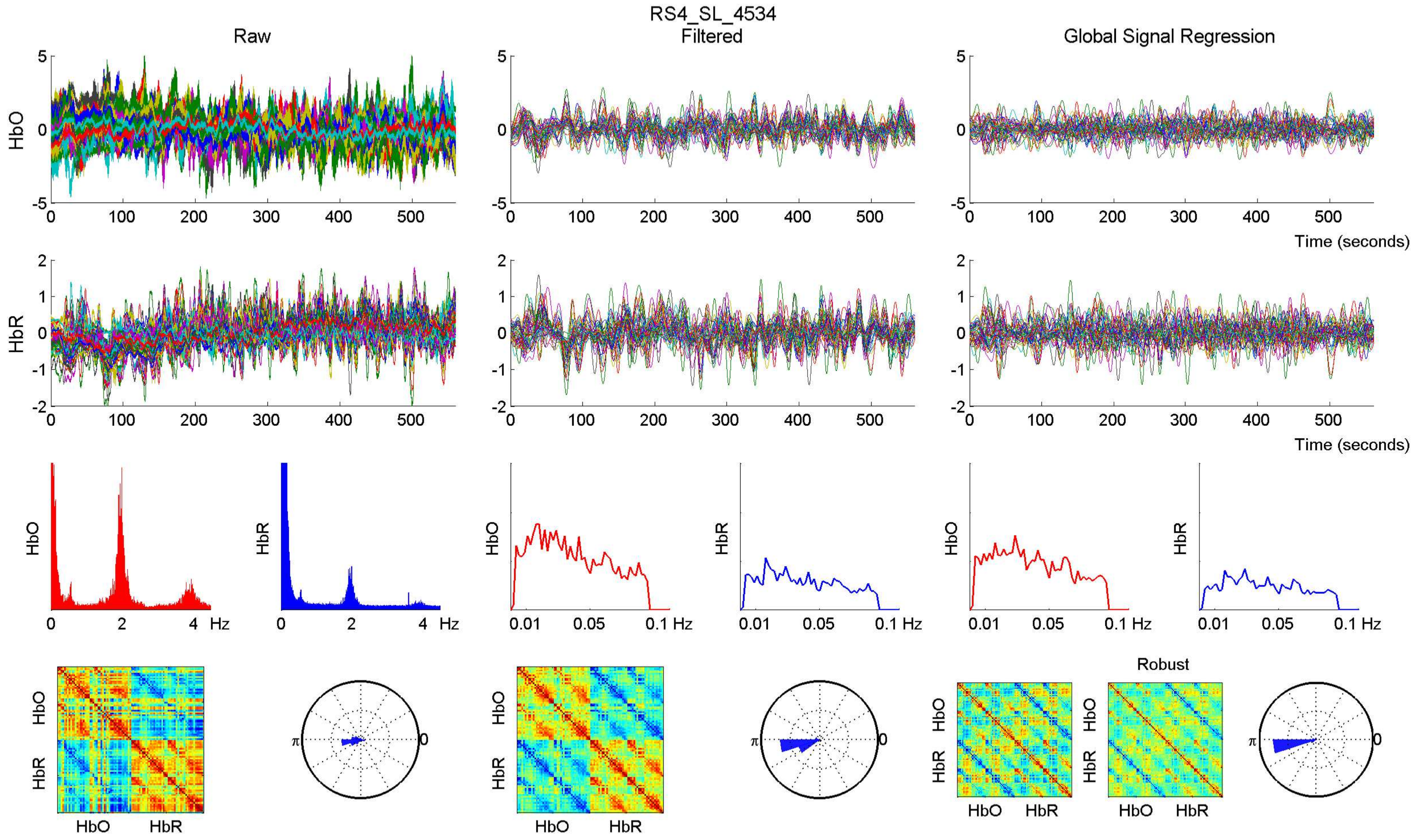
RS4_SL_4534
Raw
Filtered
Global Signal Regression
HbO
HbR
Time (seconds)
Hz
Robust

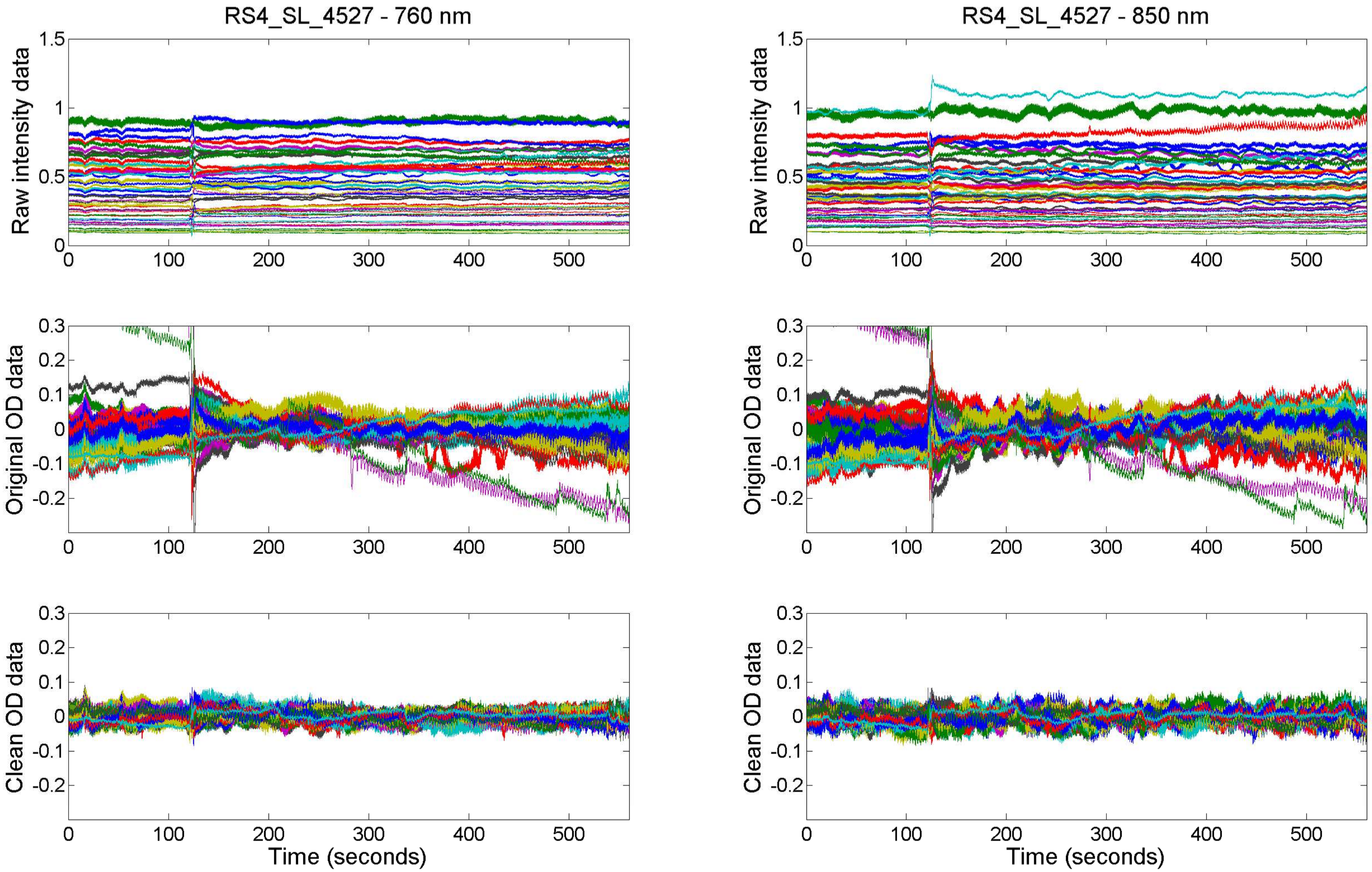
RS4_SL_4527 - 760 nm
RS4_SL_4527 - 850 nm
Raw intensity data
Original OD data
Clean OD data
Time (seconds)
1.5
1
0.5
0
0.3
0.2
0.1
-0.1
-0.2
100
200
300
400
500

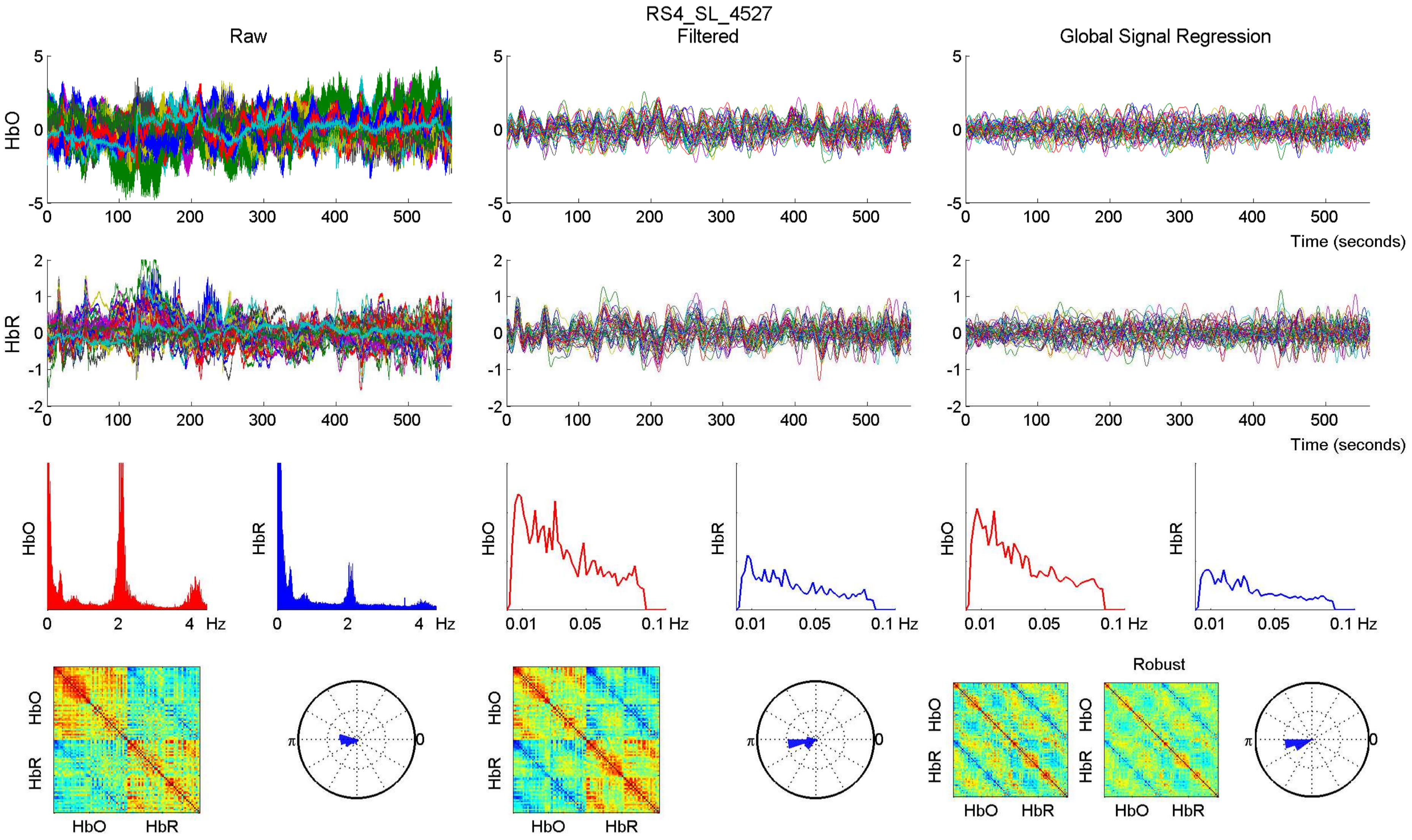
RS4_SL_4527
Raw
Filtered
Global Signal Regression
HbO
HbR
Time (seconds)
Hz
Robust

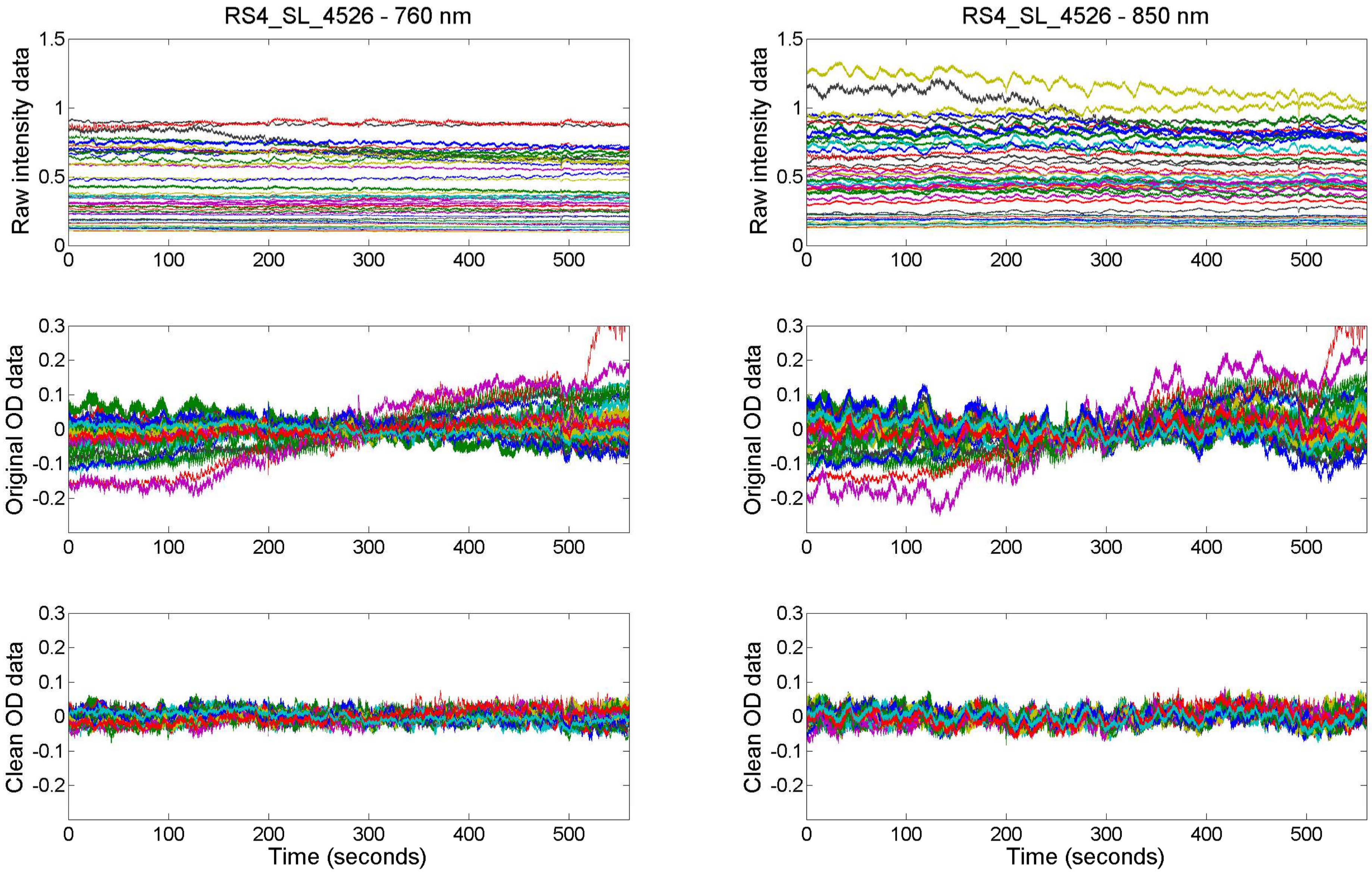

RS4_SL_4526 - 760 nm
RS4_SL_4526 - 850 nm
Raw intensity data
Original OD data
Clean OD data
Time (seconds)

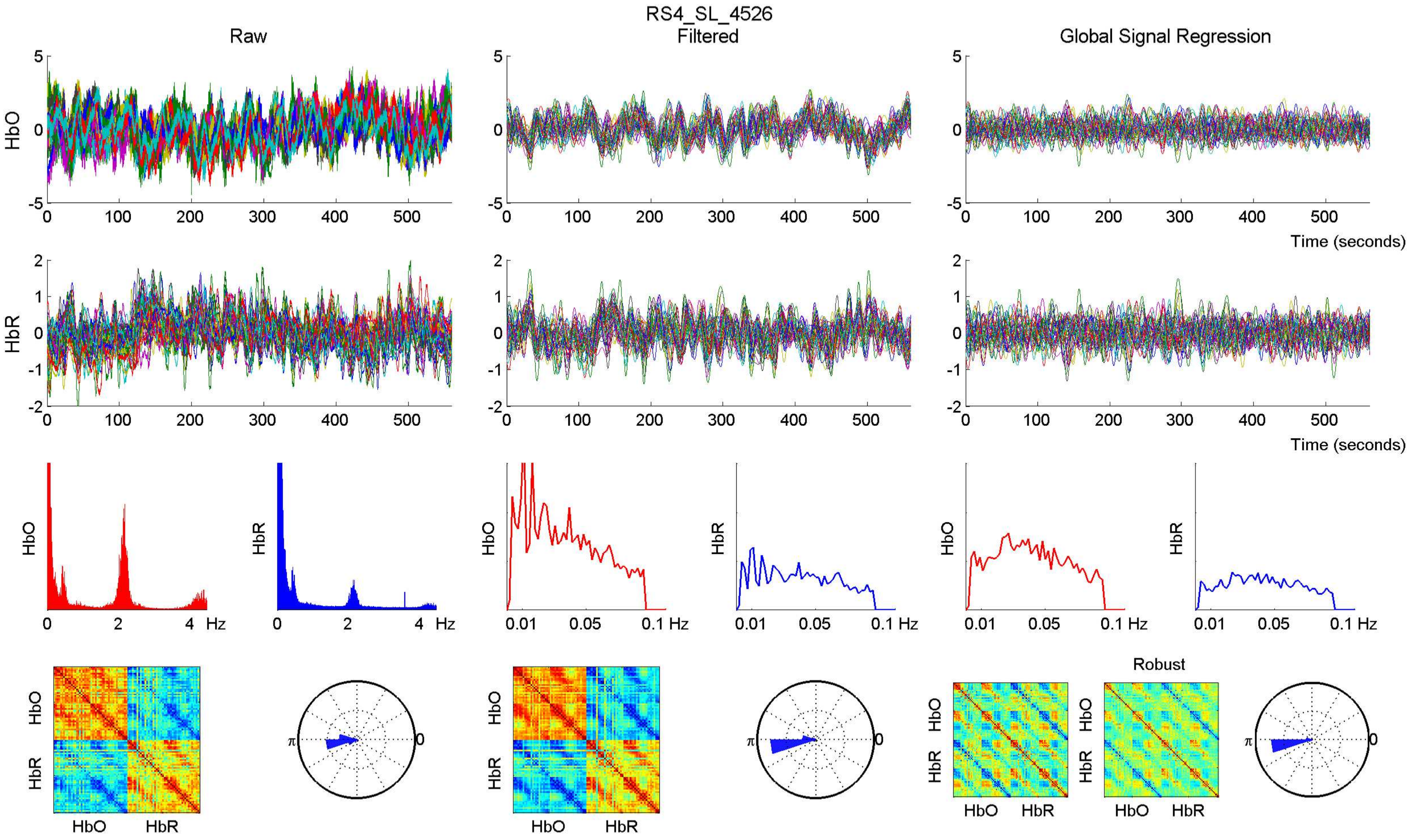

RS4_SL_4526
Raw
Filtered
Global Signal Regression
HbO
HbR
Time (seconds)
Hz
Robust
HbO
HbR

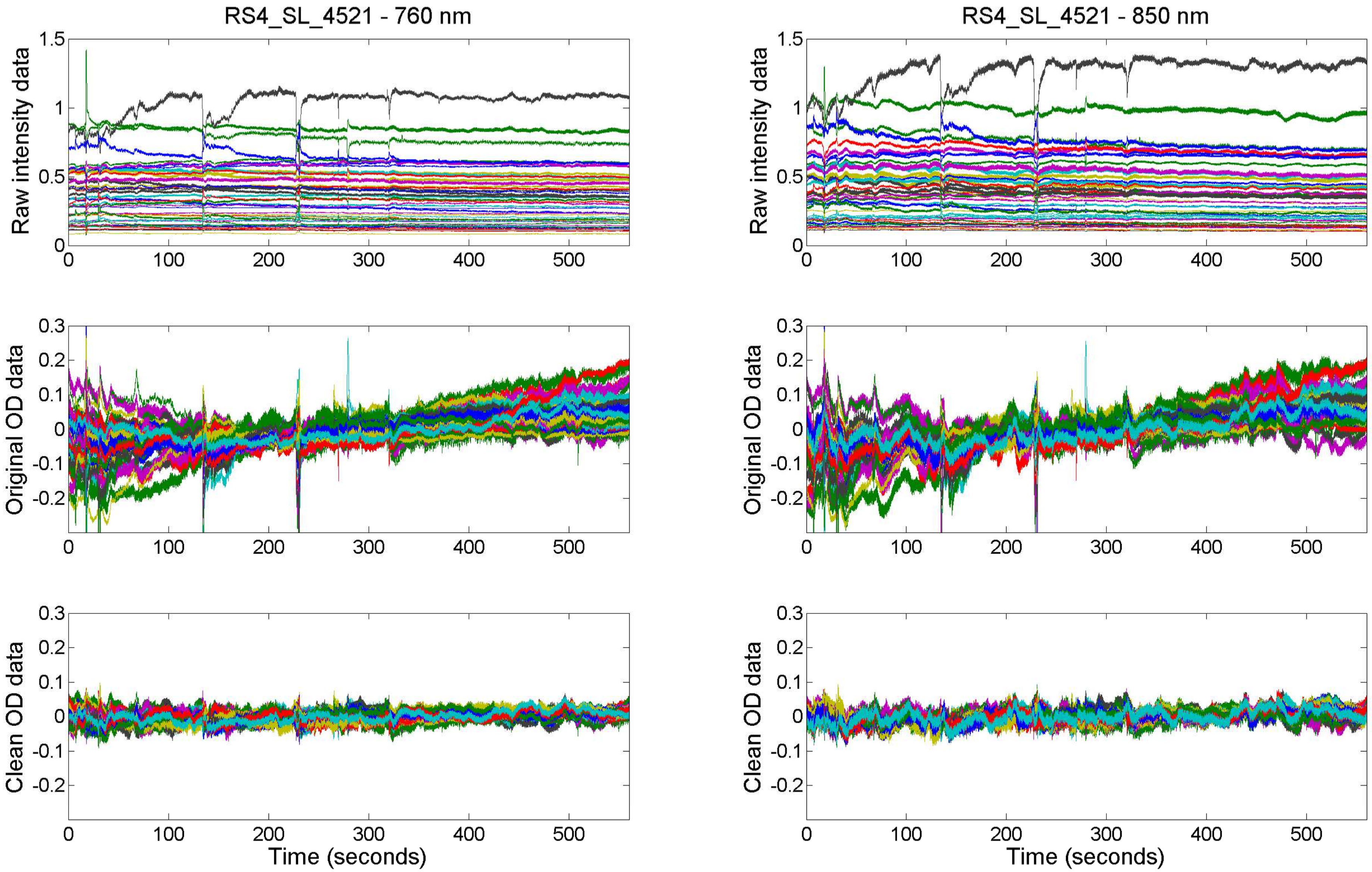

RS4_SL_4521 - 760 nm
RS4_SL_4521 - 850 nm
Raw intensity data
Original OD data
Clean OD data
Time (seconds)

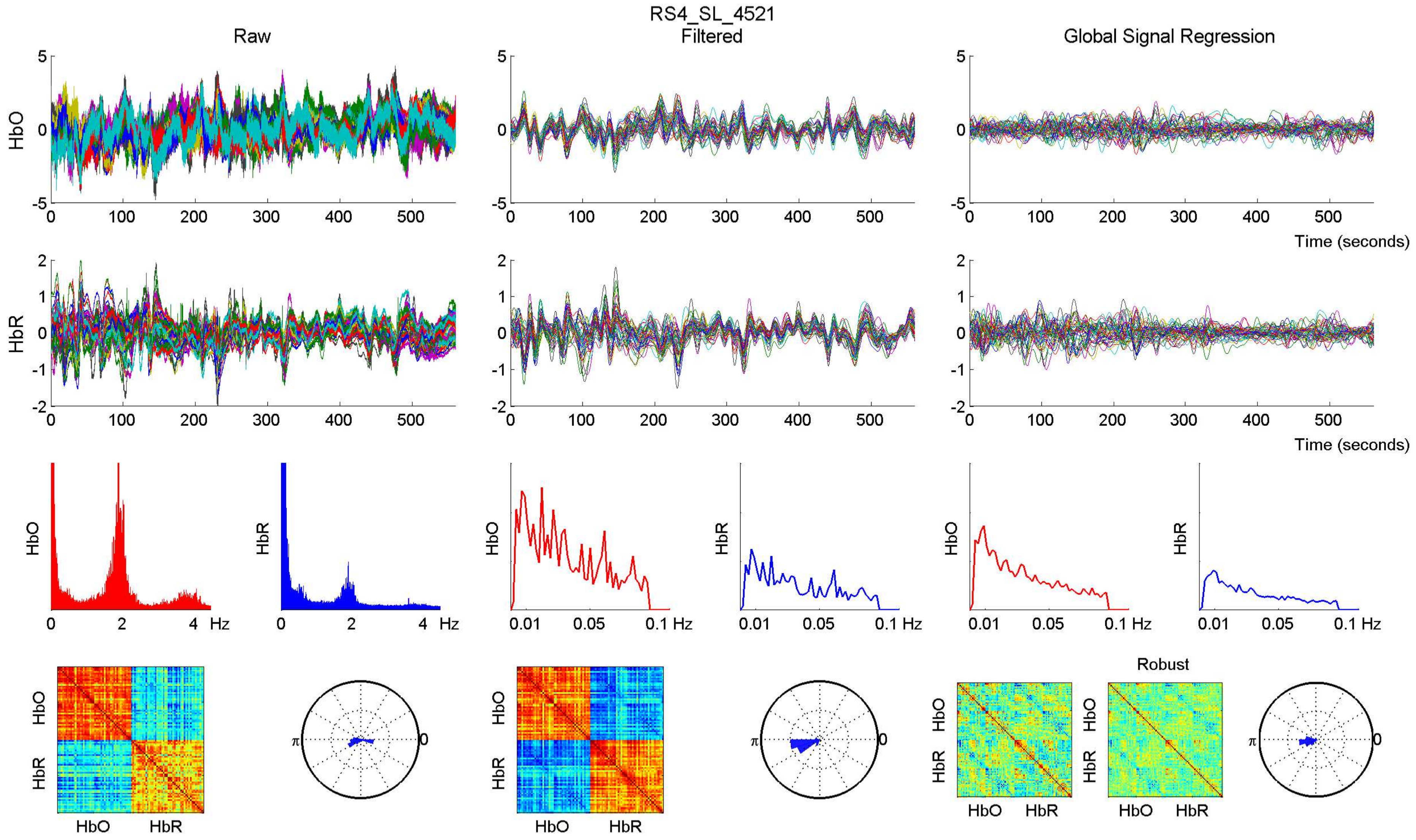

RS4_SL_4521
Raw
Filtered
Global Signal Regression
HbO
HbR
Time (seconds)
Hz
0.01
0.05
0.1 Hz
Robust
HbO
HbR
π
0

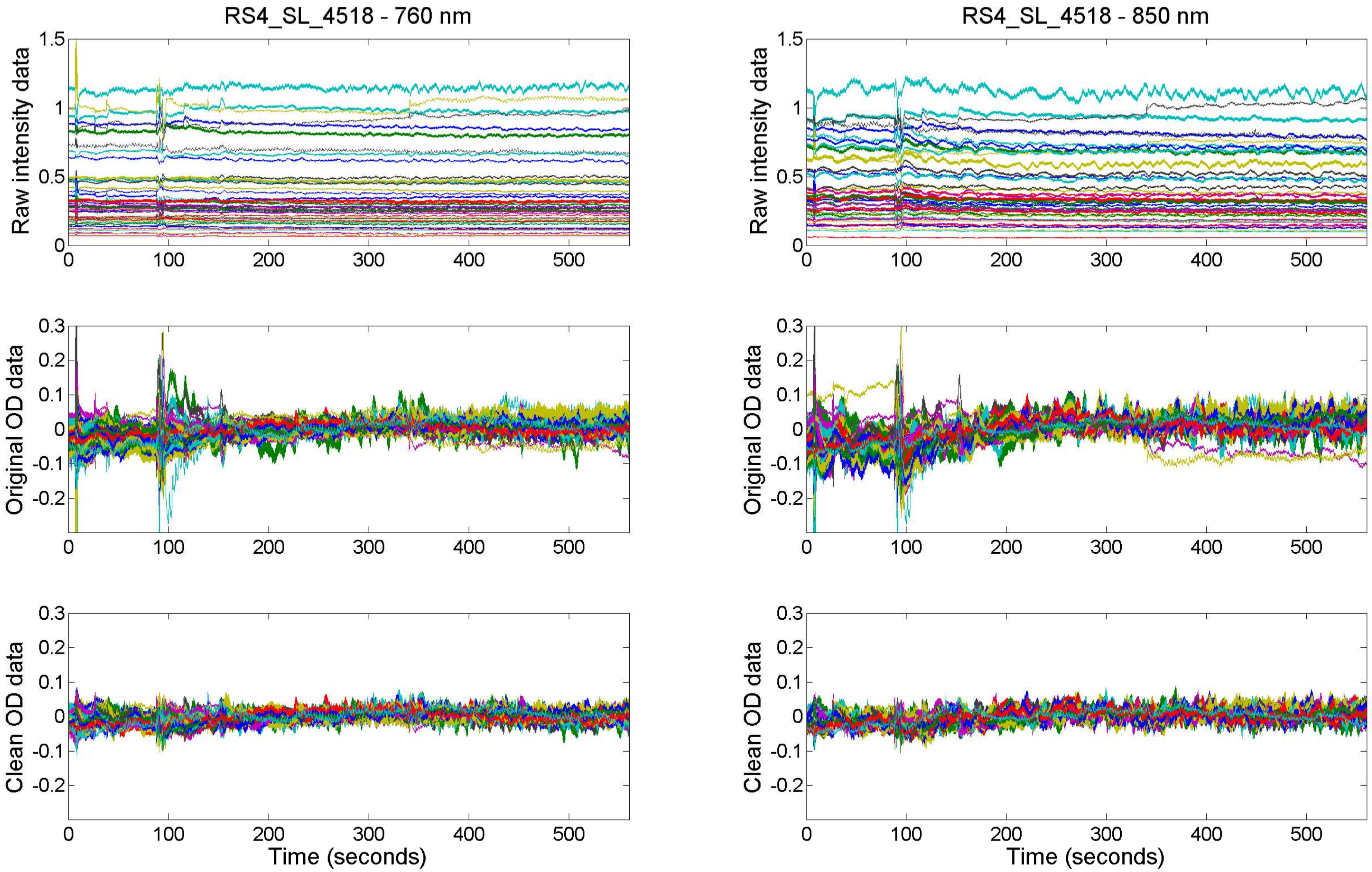

RS4_SL_4518 - 760 nm
RS4_SL_4518 - 850 nm
Raw intensity data
Original OD data
Clean OD data
Time (seconds)

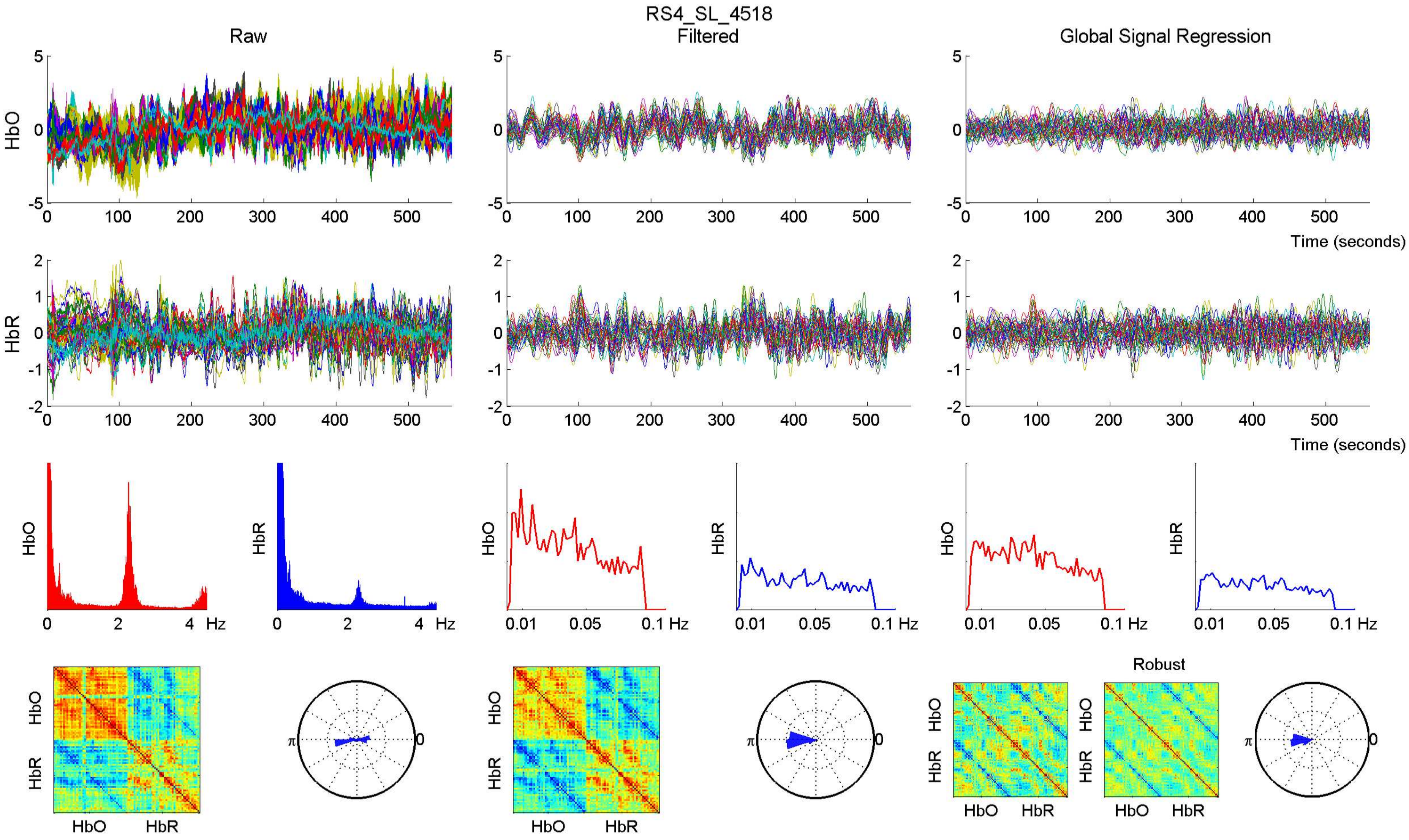

RS4_SL_4518
Raw
Filtered
Global Signal Regression
HbO
HbR
Time (seconds)
Hz
Robust

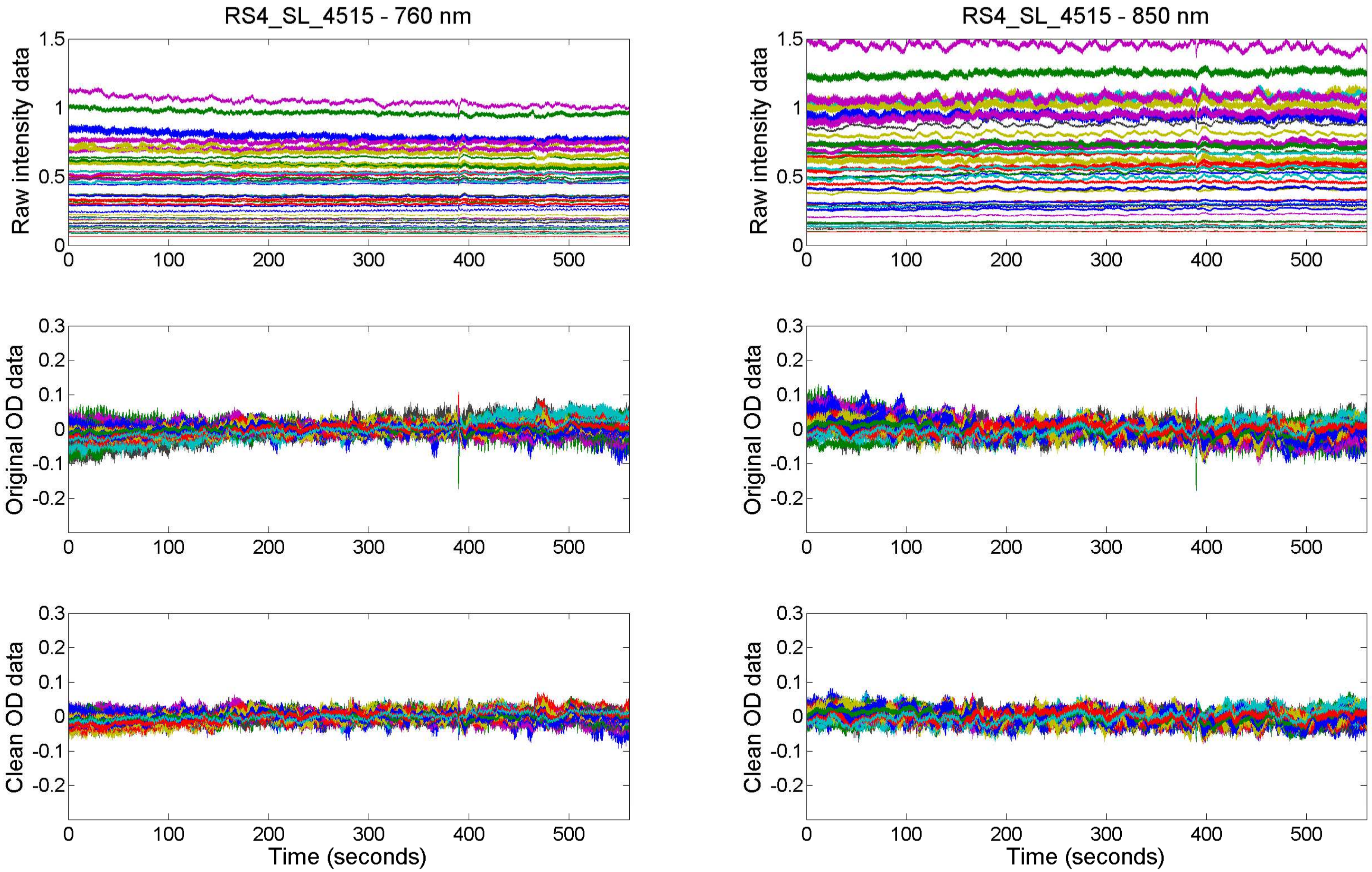

RS4_SL_4515 - 760 nm
RS4_SL_4515 - 850 nm
Raw intensity data
Original OD data
Clean OD data
Time (seconds)

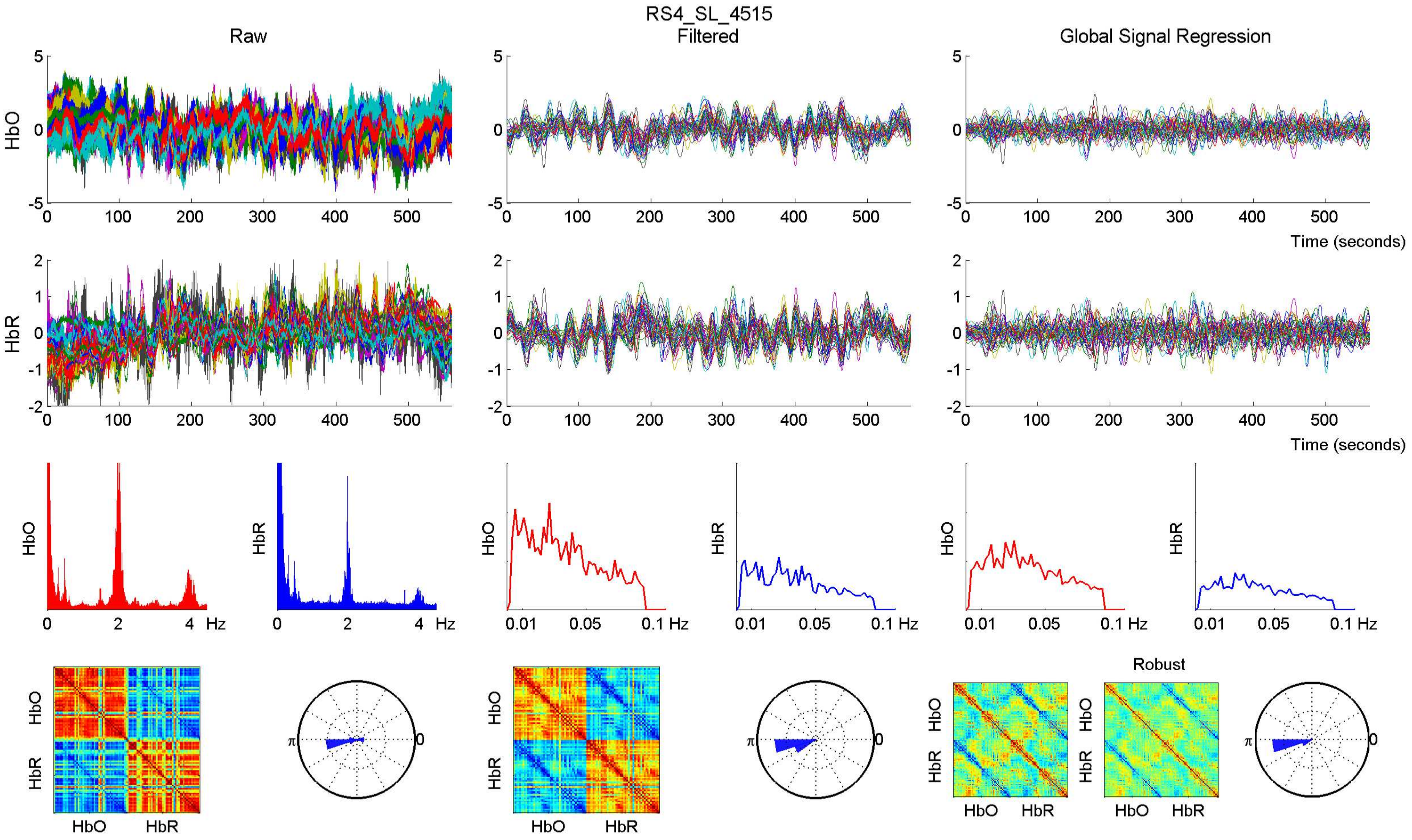

RS4_SL_4515
Raw
Filtered
Global Signal Regression
HbO
HbR
Time (seconds)
Hz
0.01
0.05
0.1 Hz
Robust
HbO
HbR
π
0

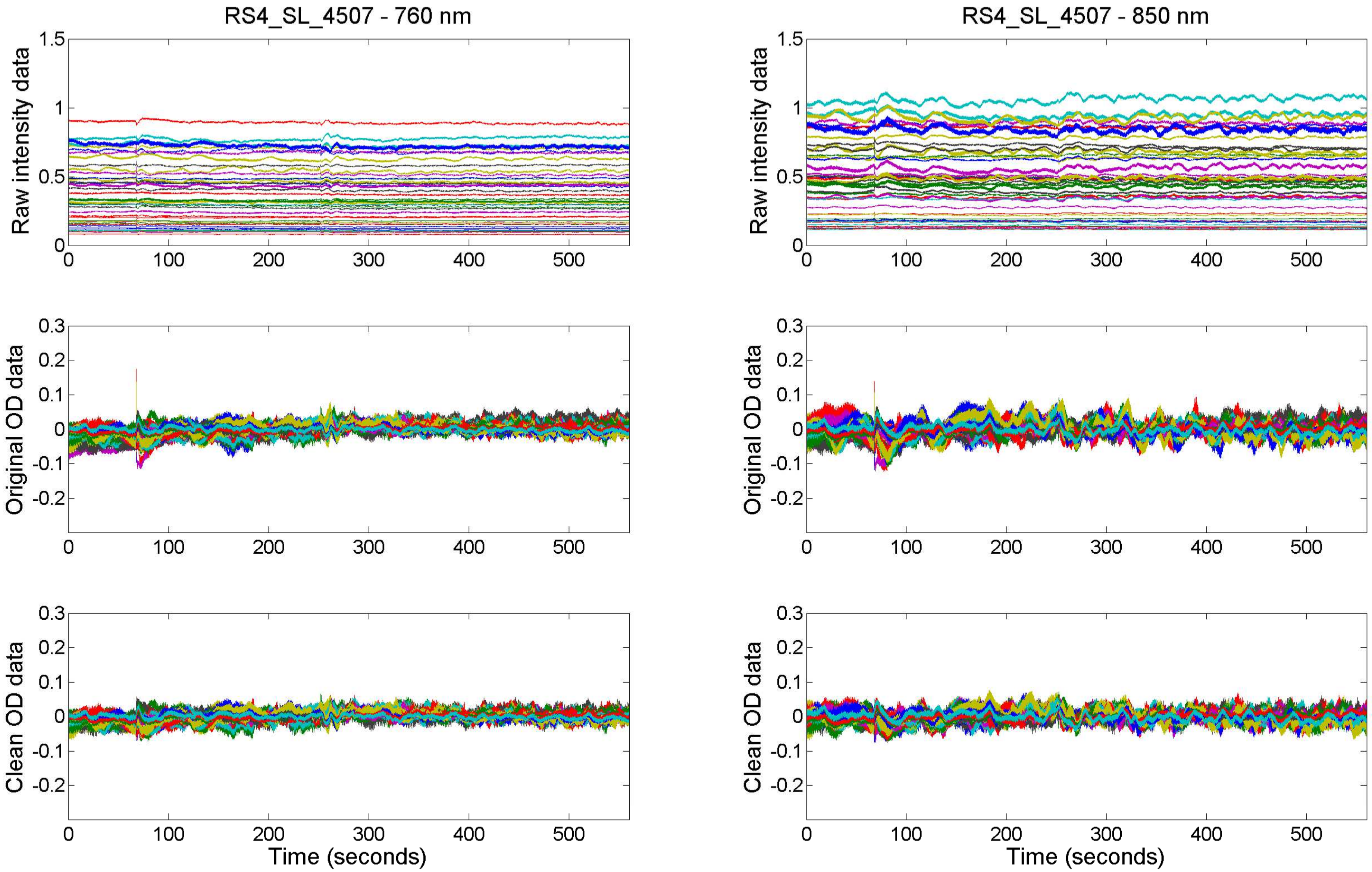
RS4_SL_4507 - 760 nm
RS4_SL_4507 - 850 nm
Raw intensity data
Original OD data
Clean OD data
Time (seconds)
Raw intensity data
Original OD data
Clean OD data
Time (seconds)

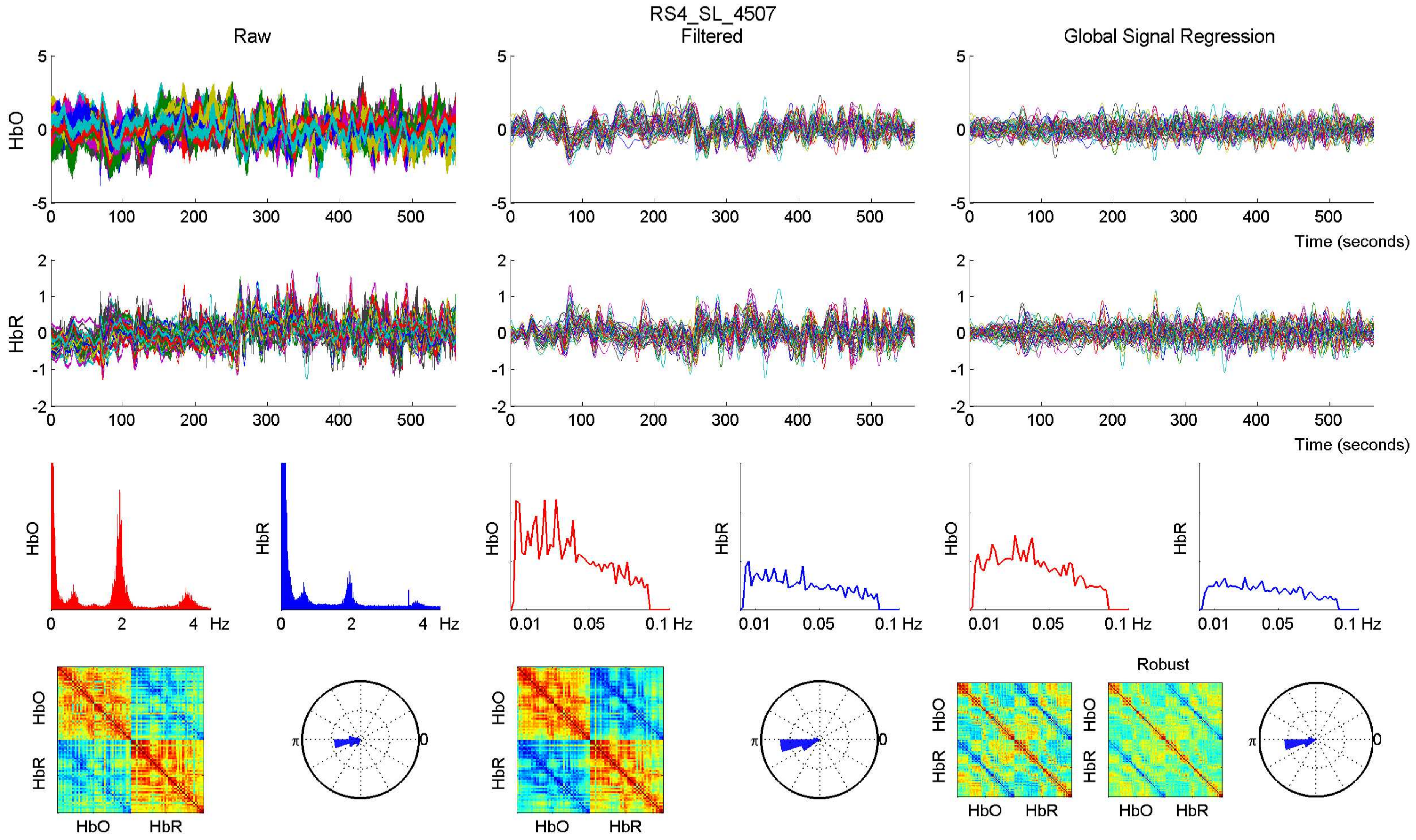

RS4_SL_4507
Raw
Filtered
Global Signal Regression
HbO
HbR
Time (seconds)
Hz
Robust

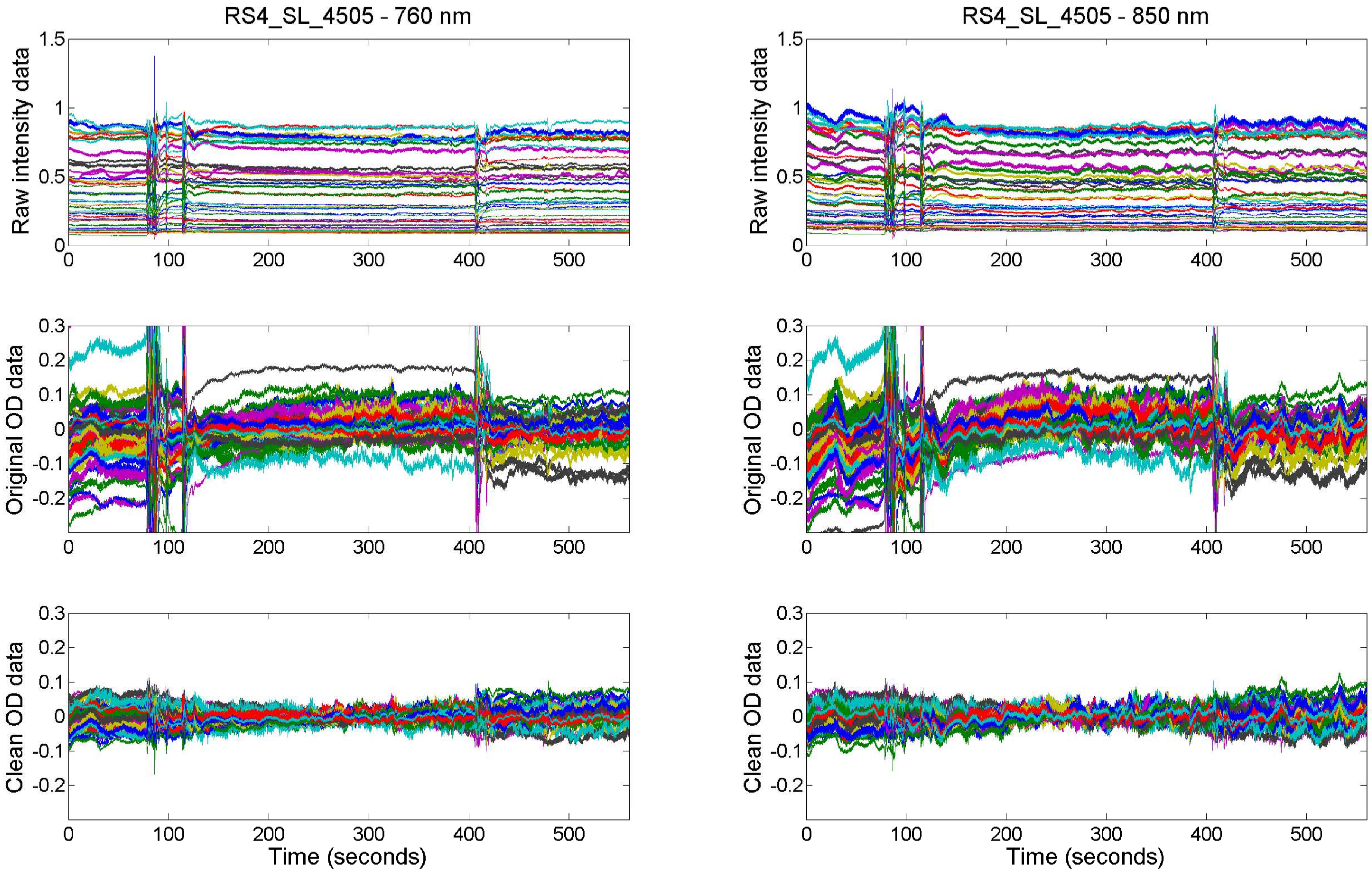
RS4_SL_4505 - 760 nm
RS4_SL_4505 - 850 nm
Raw intensity data
Original OD data
Clean OD data
Time (seconds)
0
0.5
1
1.5
-0.2
-0.1
0.1
0.2
0.3
100
200
300
400
500

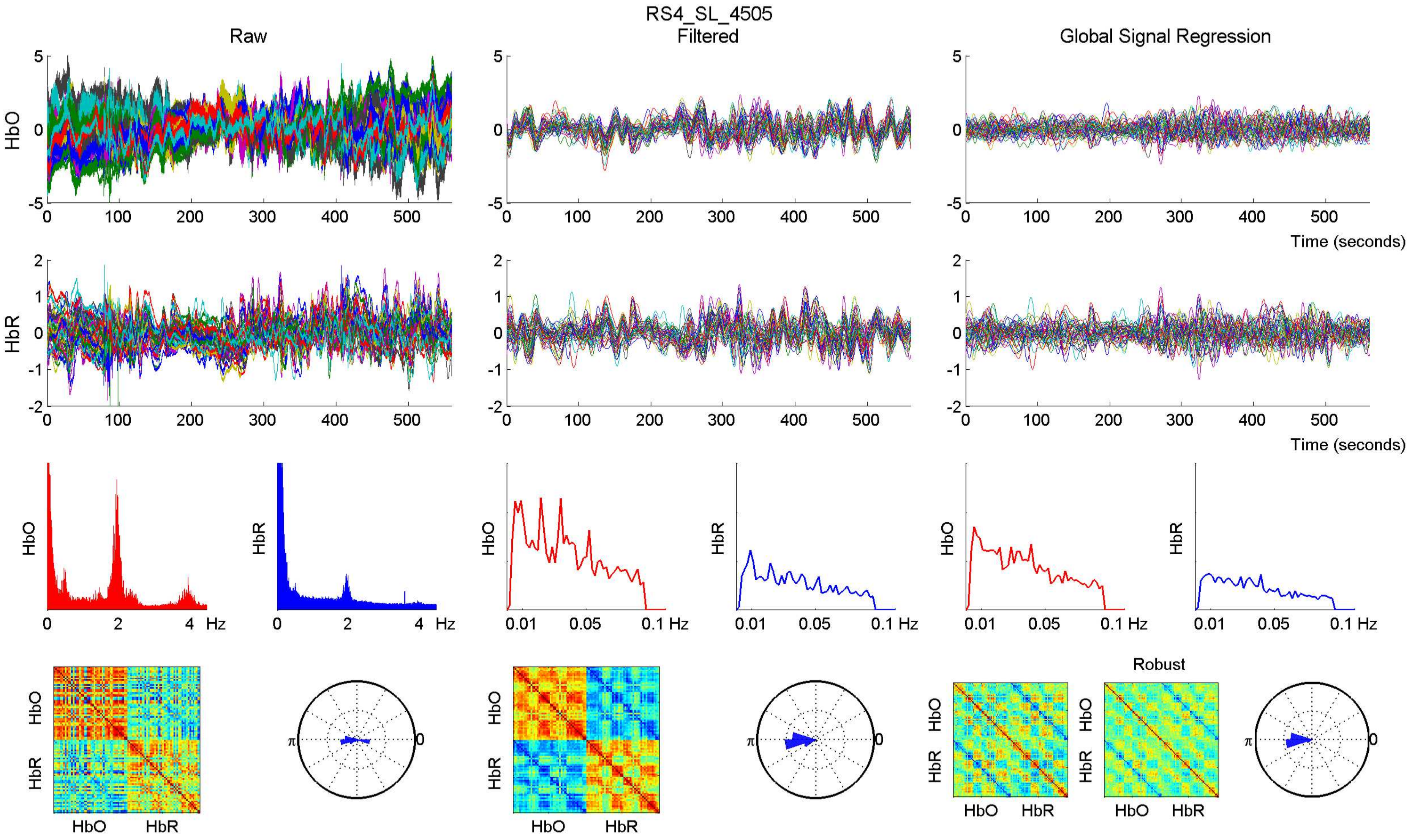
RS4_SL_4505
Raw
Filtered
Global Signal Regression
HbO
HbR
Time (seconds)
Hz
0.01
0.05
0.1 Hz
Robust
π
0

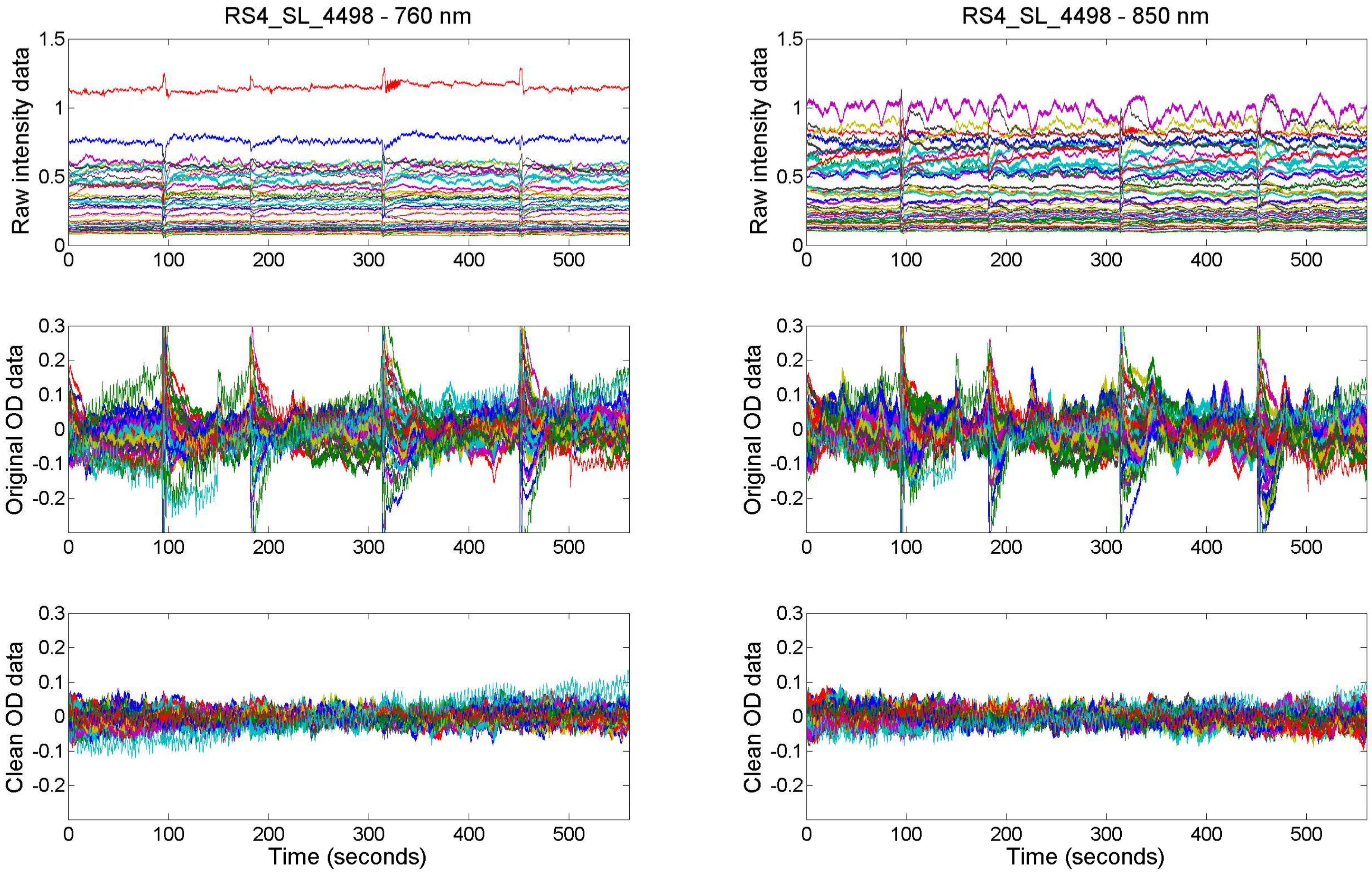
RS4_SL_4498 - 760 nm
RS4_SL_4498 - 850 nm
Raw intensity data
Original OD data
Clean OD data
Time (seconds)

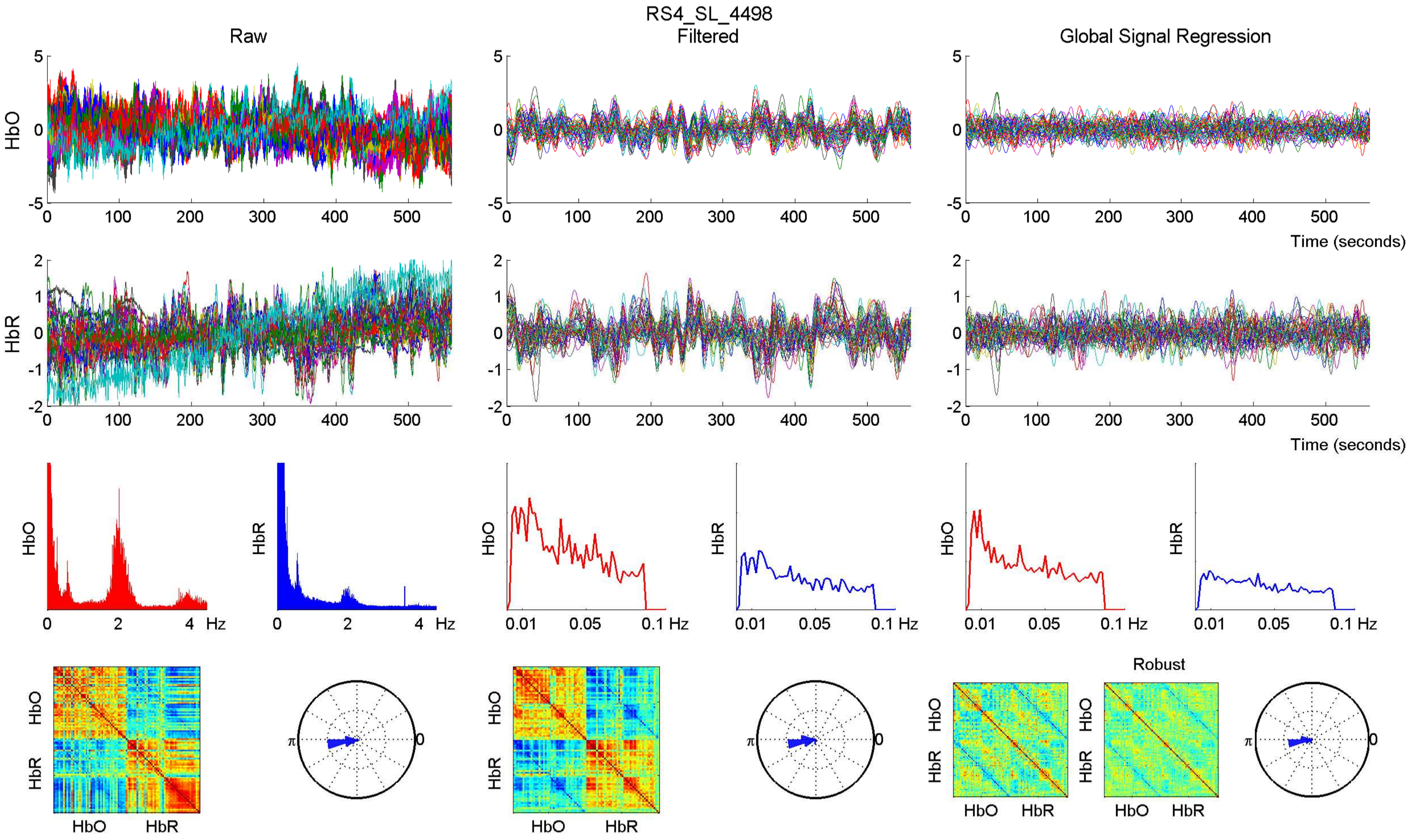

RS4_SL_4498
Raw
Filtered
Global Signal Regression
HbO
HbR
Time (seconds)
Hz
Robust

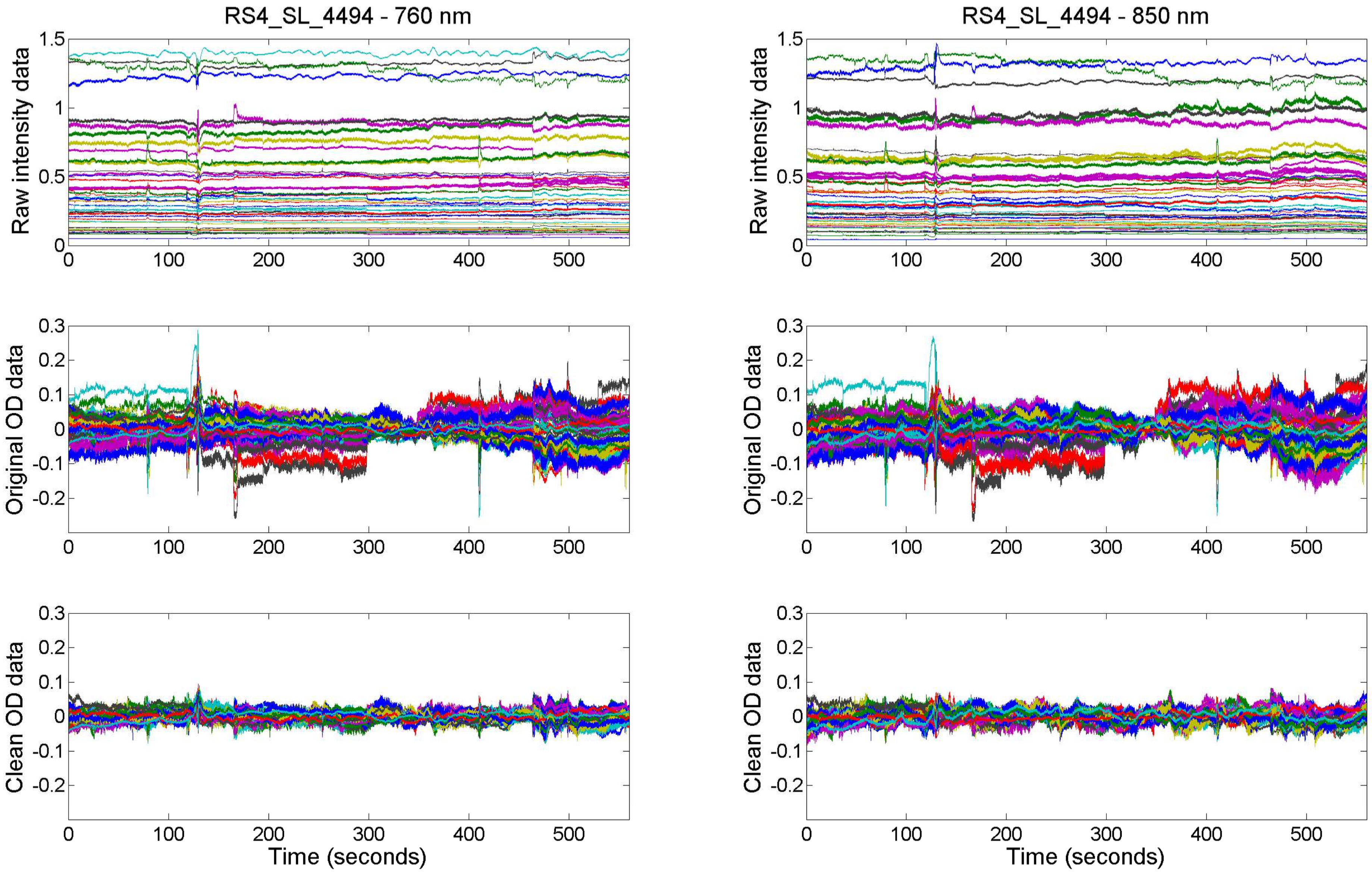

RS4_SL_4494 - 760 nm
RS4_SL_4494 - 850 nm
Raw intensity data
Original OD data
Clean OD data
Time (seconds)

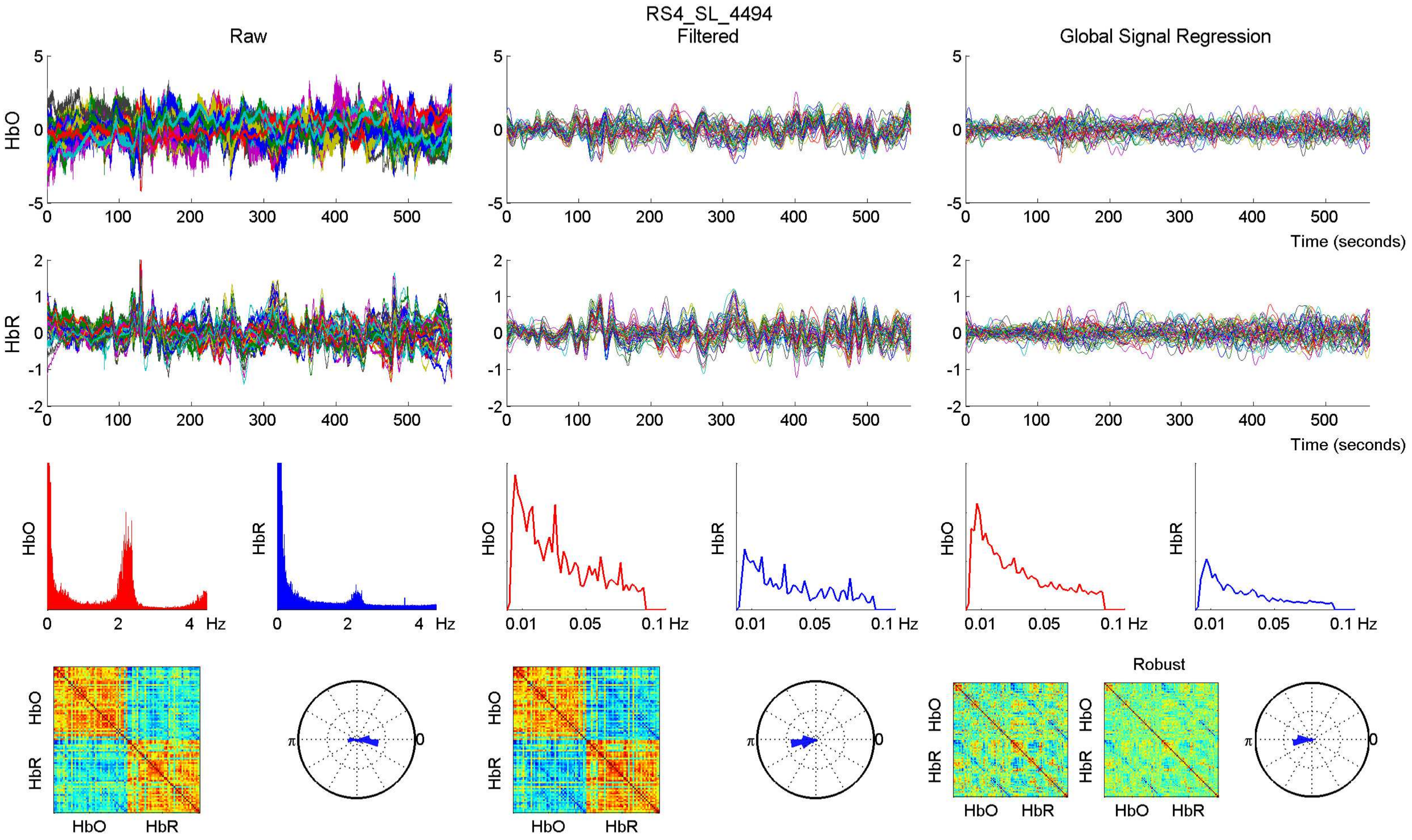

RS4_SL_4494
Raw
Filtered
Global Signal Regression
HbO
HbR
Time (seconds)
Hz
Robust

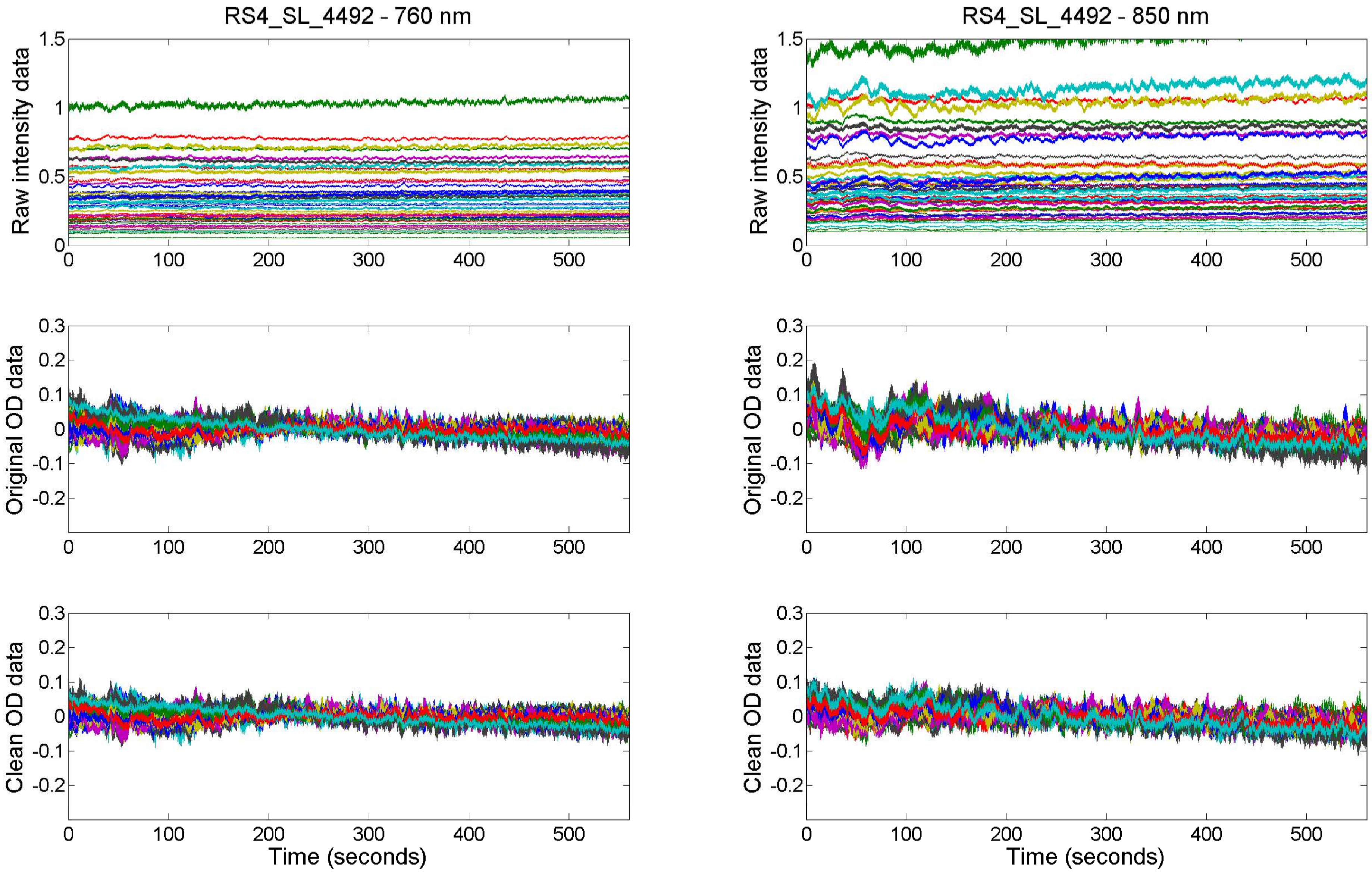
RS4_SL_4492 - 760 nm
RS4_SL_4492 - 850 nm
Raw intensity data
Original OD data
Clean OD data
Time (seconds)
0
100
200
300
400
500
1.5
1
0.5
0.3
0.2
0.1
-0.1
-0.2

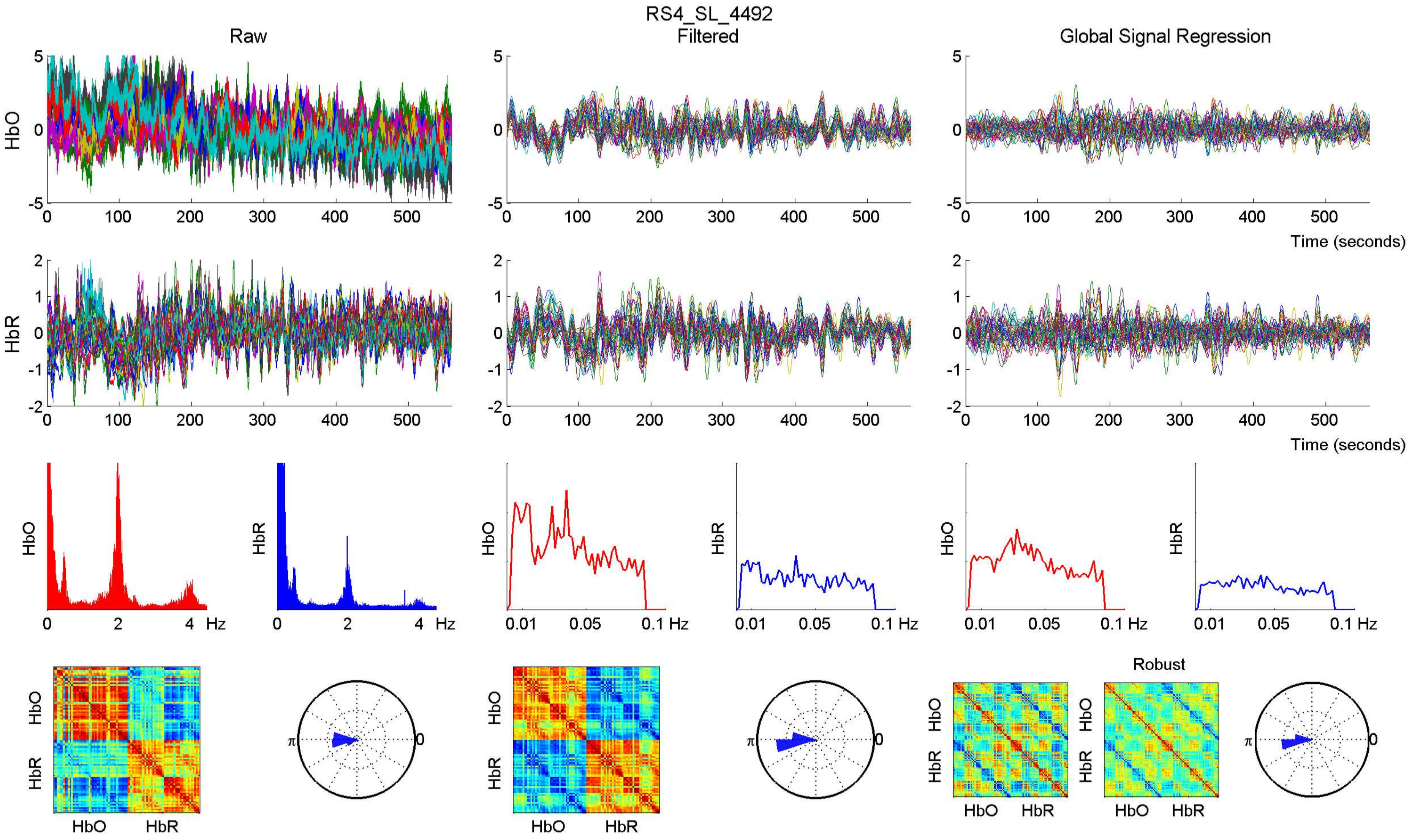
RS4_SL_4492
Raw
Filtered
Global Signal Regression
HbO
HbR
Time (seconds)
Hz
Robust

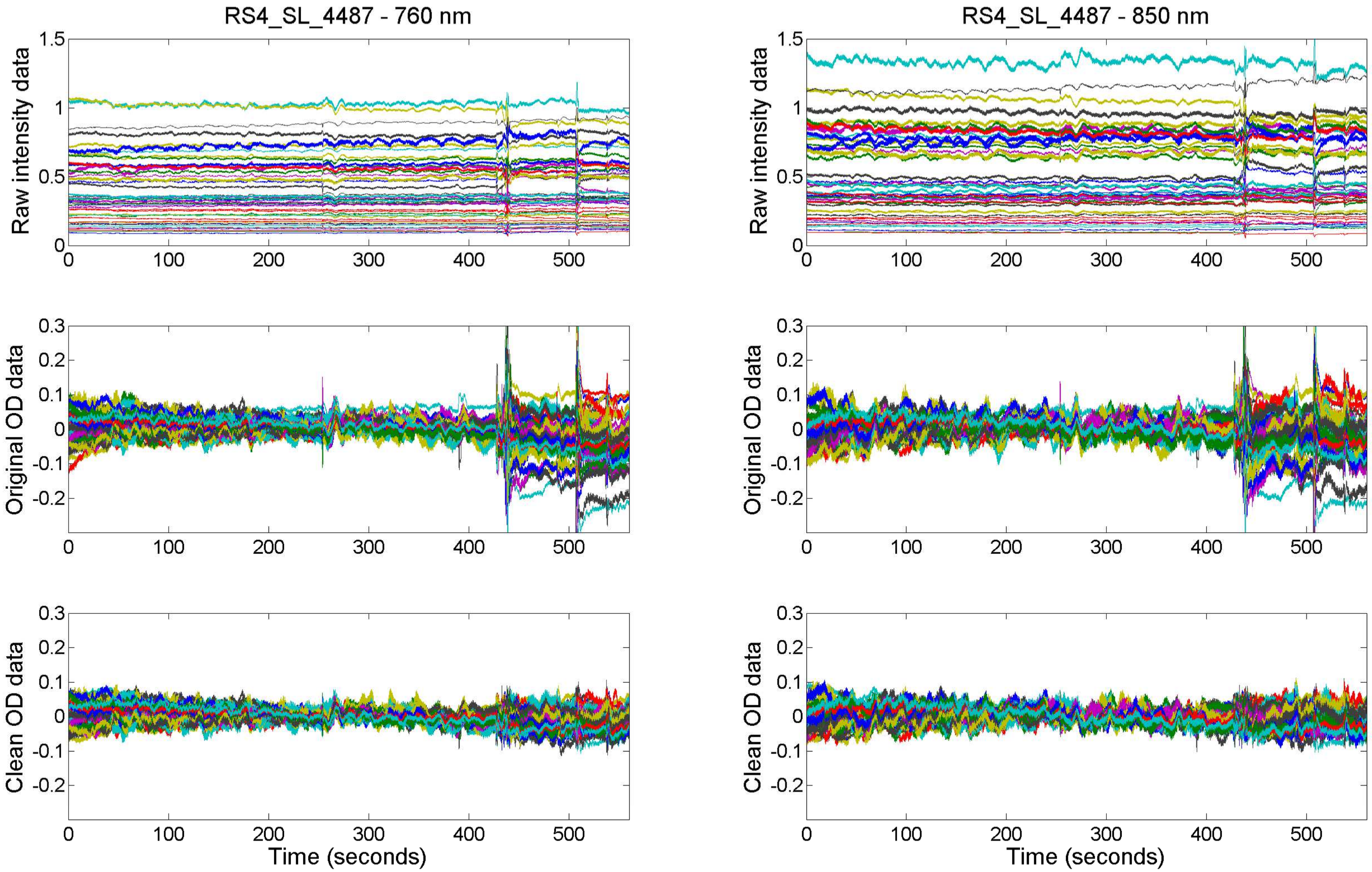
RS4_SL_4487 - 760 nm
RS4_SL_4487 - 850 nm
Raw intensity data
Original OD data
Clean OD data
Time (seconds)
1.5
1
0.5
0
0.3
0.2
0.1
-0.1
-0.2
100
200
300
400
500

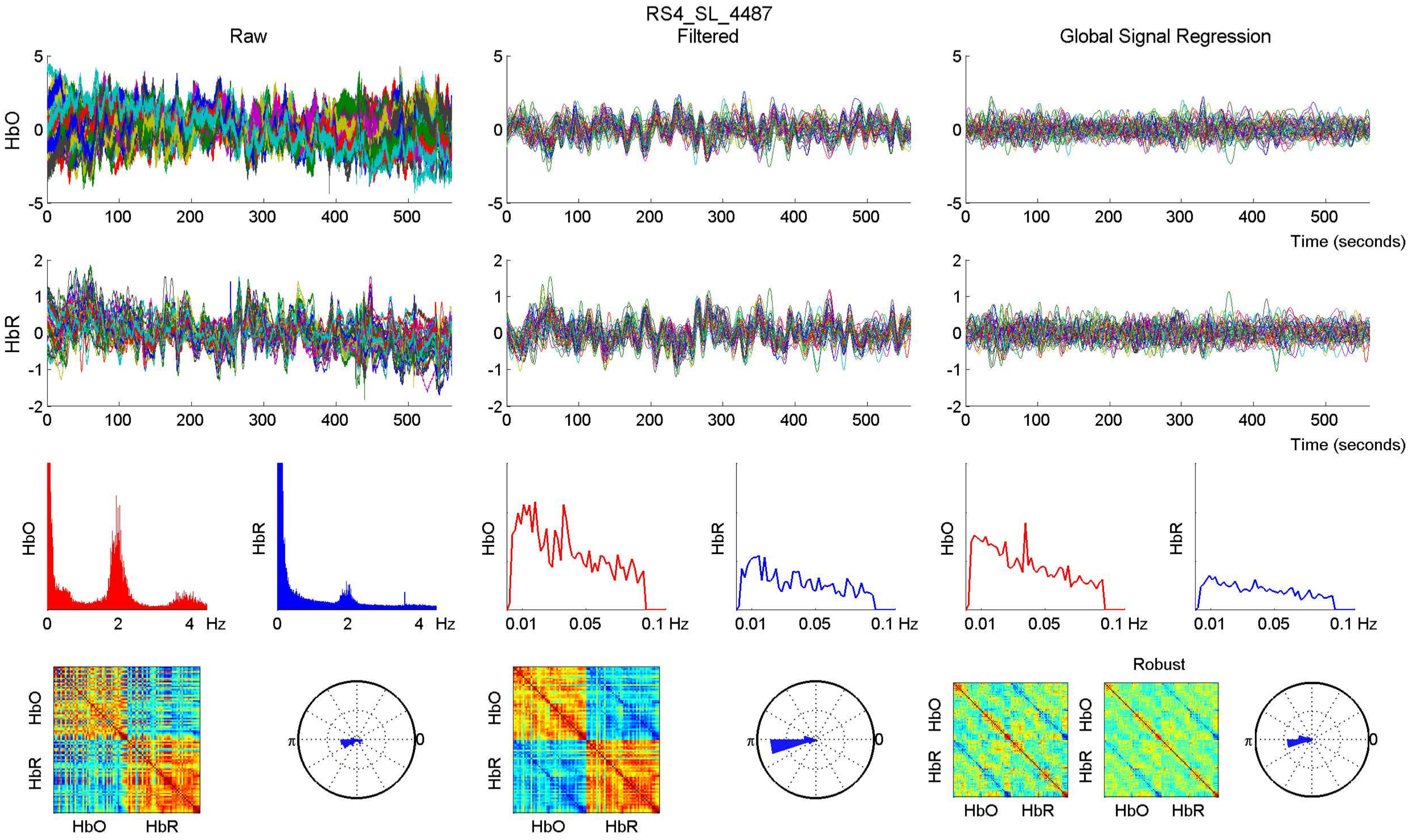
RS4_SL_4487
Raw
Filtered
Global Signal Regression
HbO
HbR
Time (seconds)
Hz
Robust
HbO
HbR

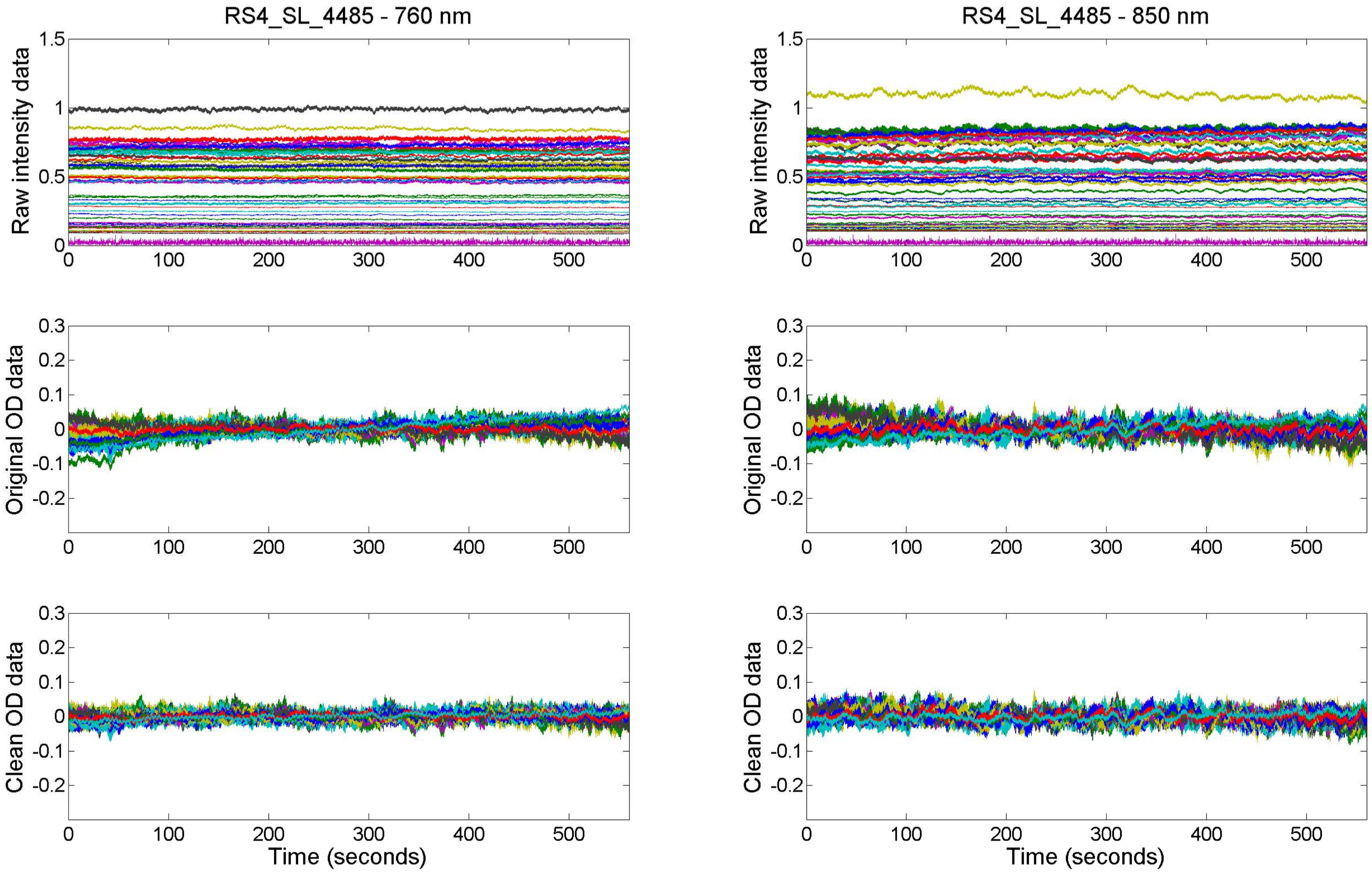
RS4_SL_4485 - 760 nm
RS4_SL_4485 - 850 nm
Raw intensity data
Original OD data
Clean OD data
Time (seconds)
0
100
200
300
400
500
1.5
1
0.5
0
0.3
0.2
0.1
-0.1
-0.2

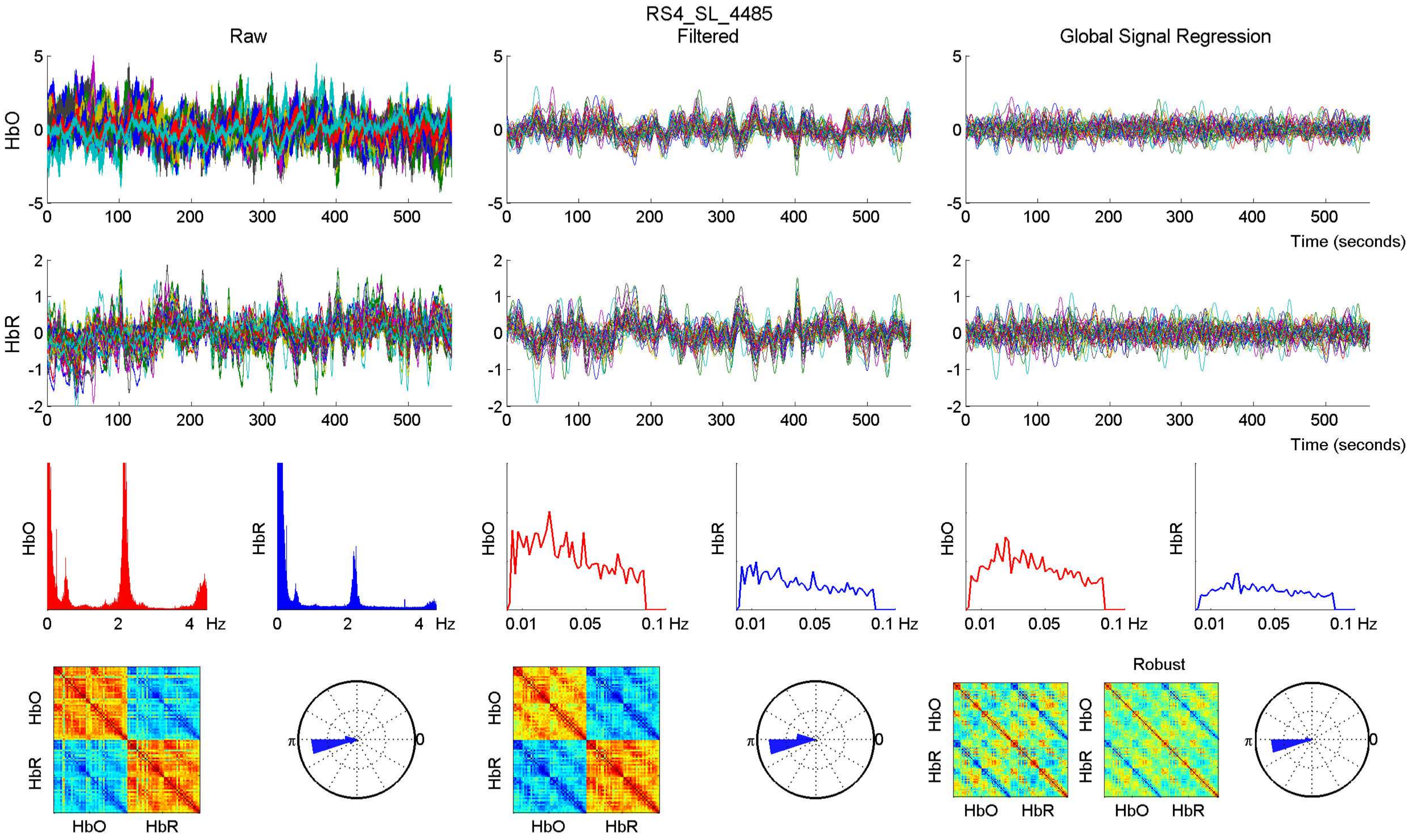

RS4_SL_4485
Raw
Filtered
Global Signal Regression
HbO
HbR
Time (seconds)
Hz
0.01
0.05
0.1 Hz
Robust
π
0

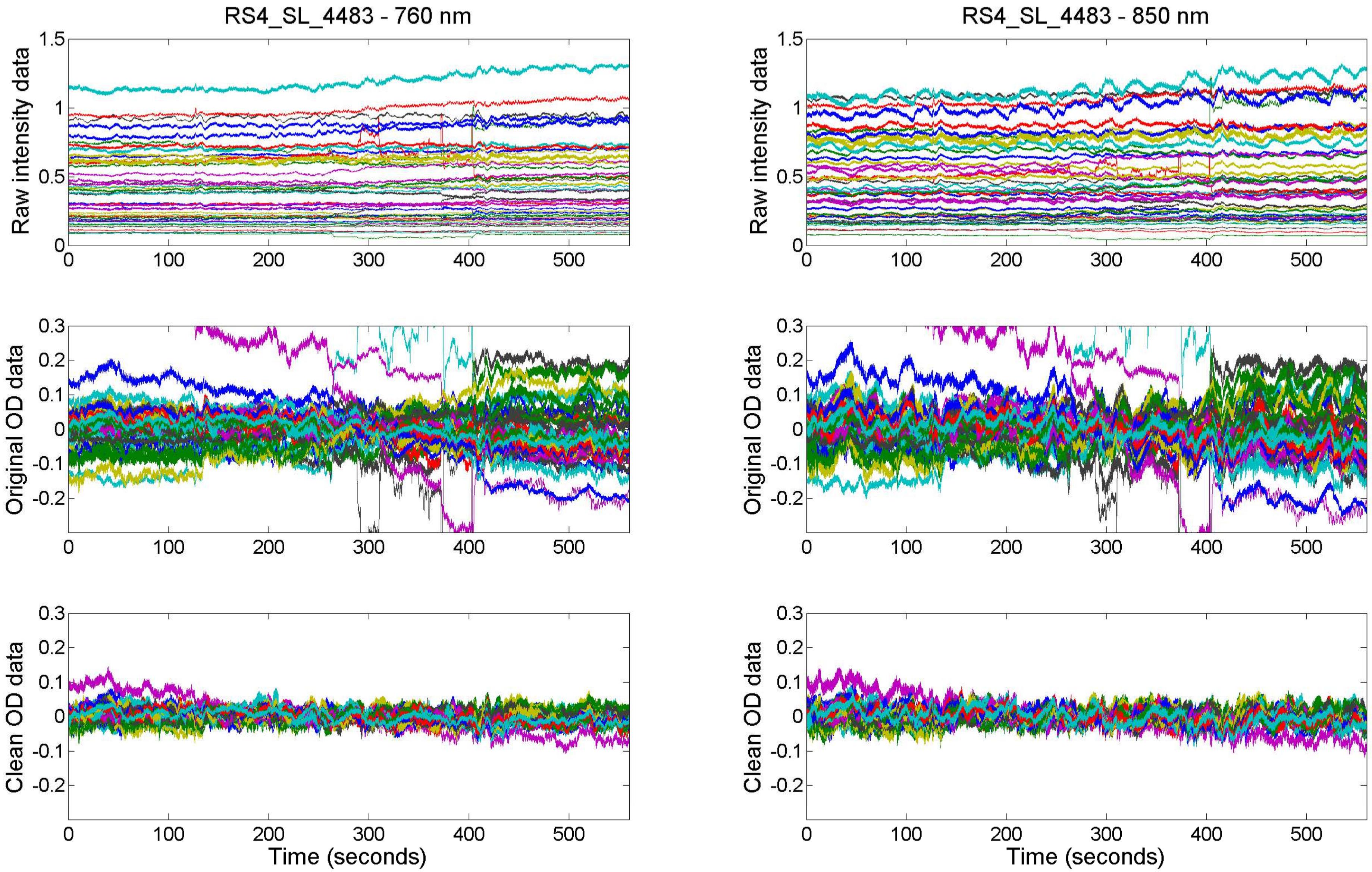

RS4_SL_4483 - 760 nm
RS4_SL_4483 - 850 nm
Raw intensity data
Original OD data
Clean OD data
Time (seconds)

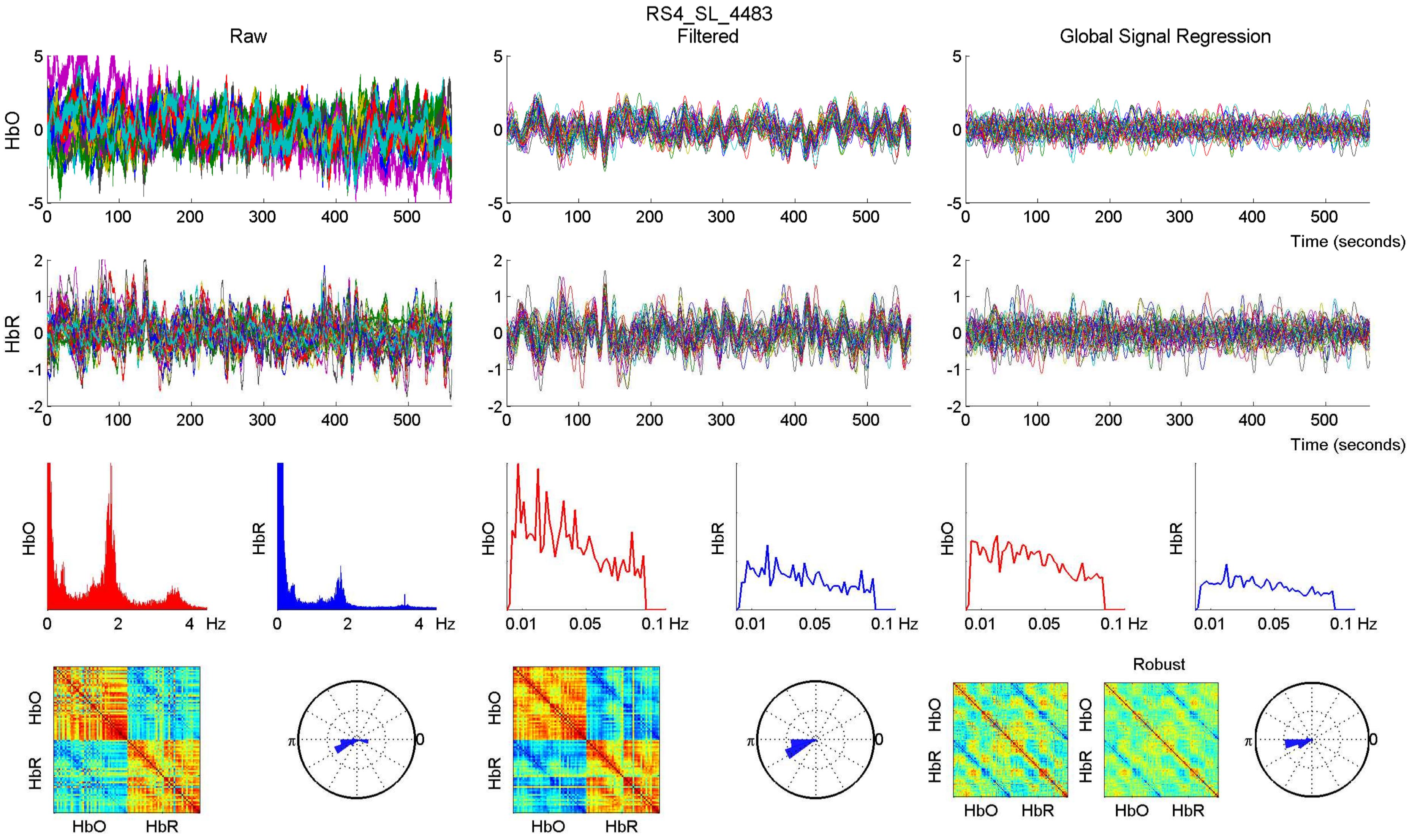

RS4_SL_4483
Raw
Filtered
Global Signal Regression
HbO
HbR
Time (seconds)
Hz
Robust
HbO
HbR

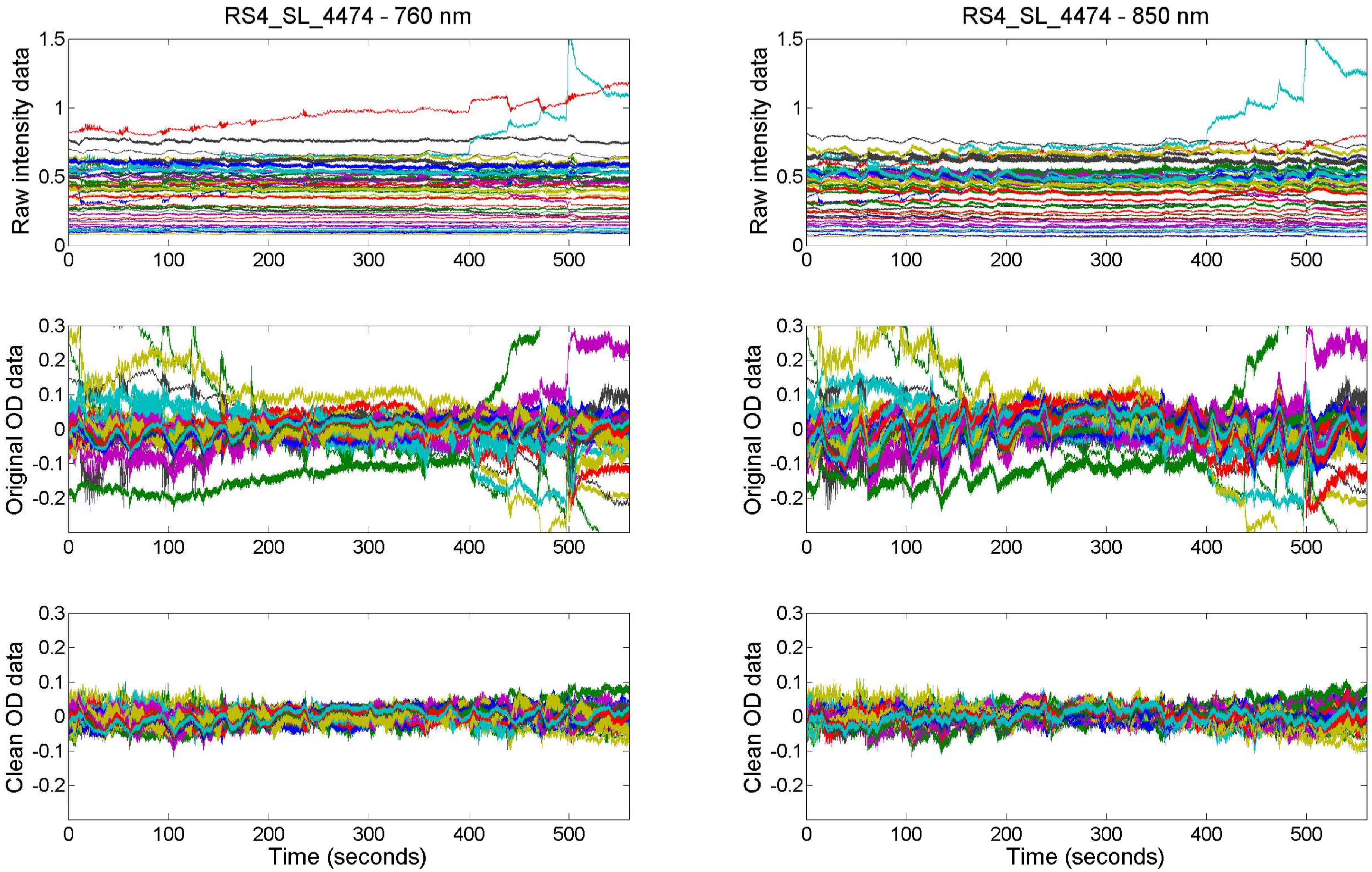

RS4_SL_4474 - 760 nm
RS4_SL_4474 - 850 nm
Raw intensity data
Original OD data
Clean OD data
Time (seconds)

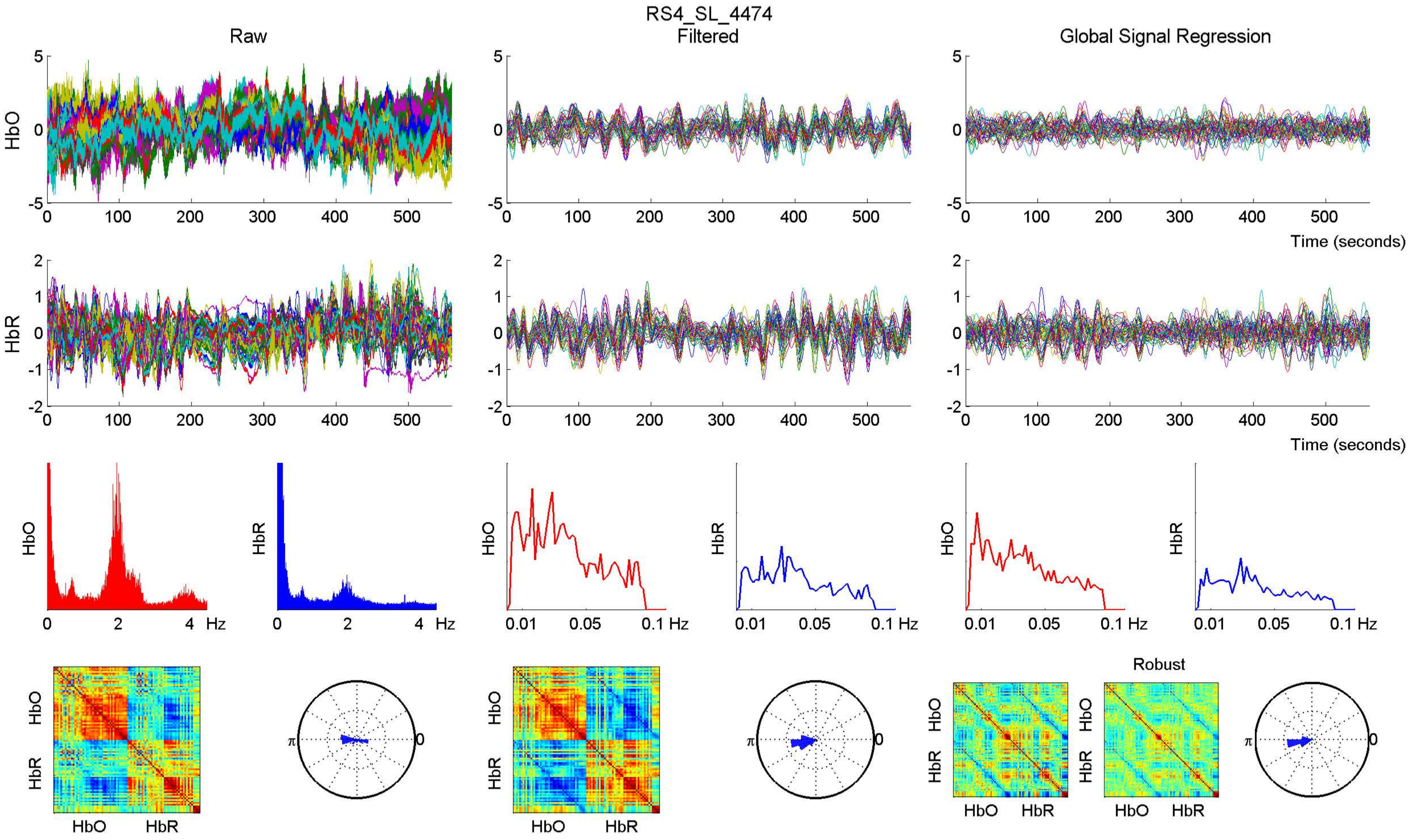

RS4_SL_4474
Raw
Filtered
Global Signal Regression
HbO
HbR
Time (seconds)
Hz
0.01
0.05
0.1 Hz
Robust
π
0
HbO
HbR

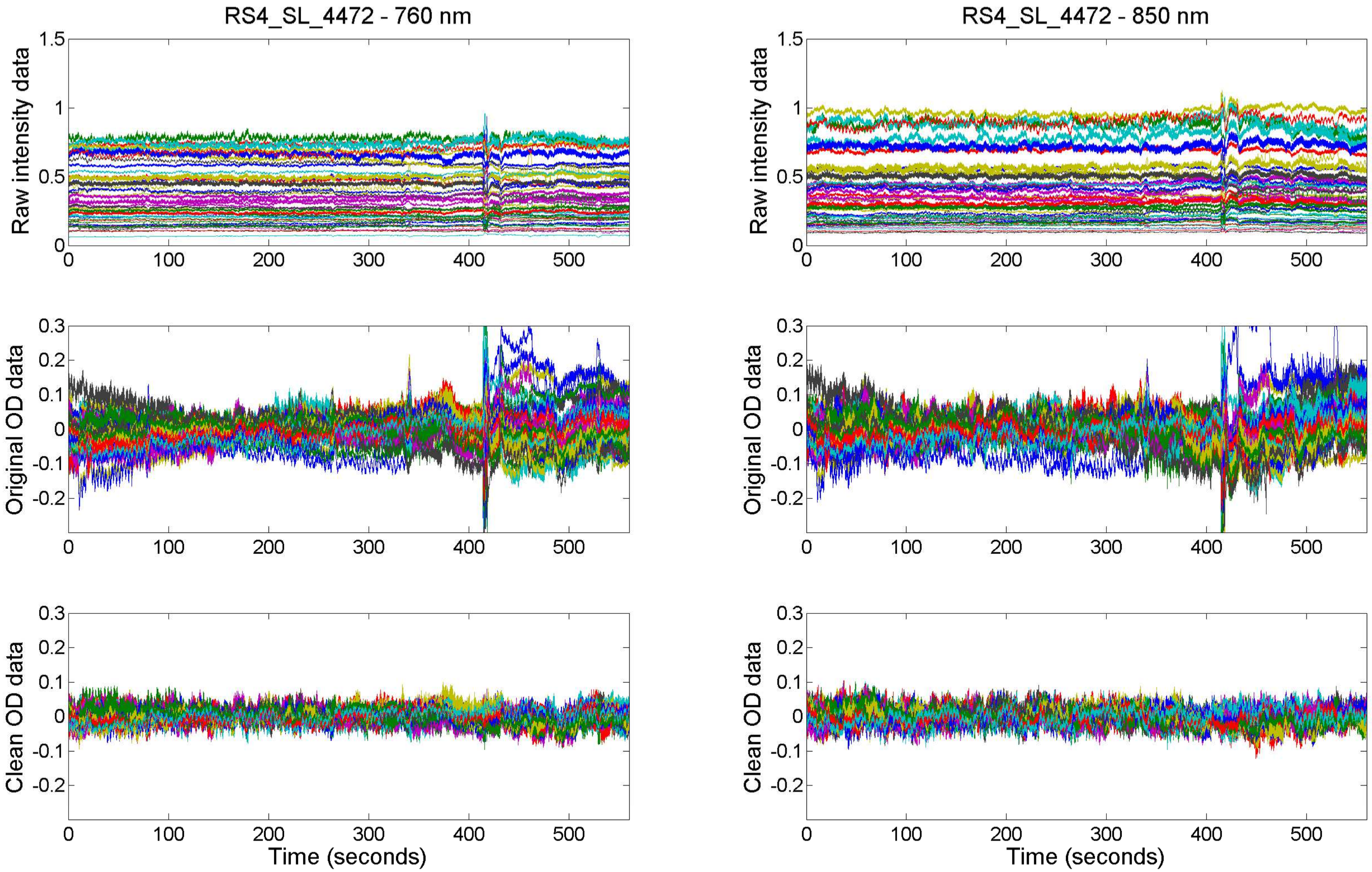

RS4_SL_4472 - 760 nm
RS4_SL_4472 - 850 nm
Raw intensity data
Original OD data
Clean OD data
Time (seconds)
1.5
1
0.5
0
0.3
0.2
0.1
-0.1
-0.2
100
200
300
400
500

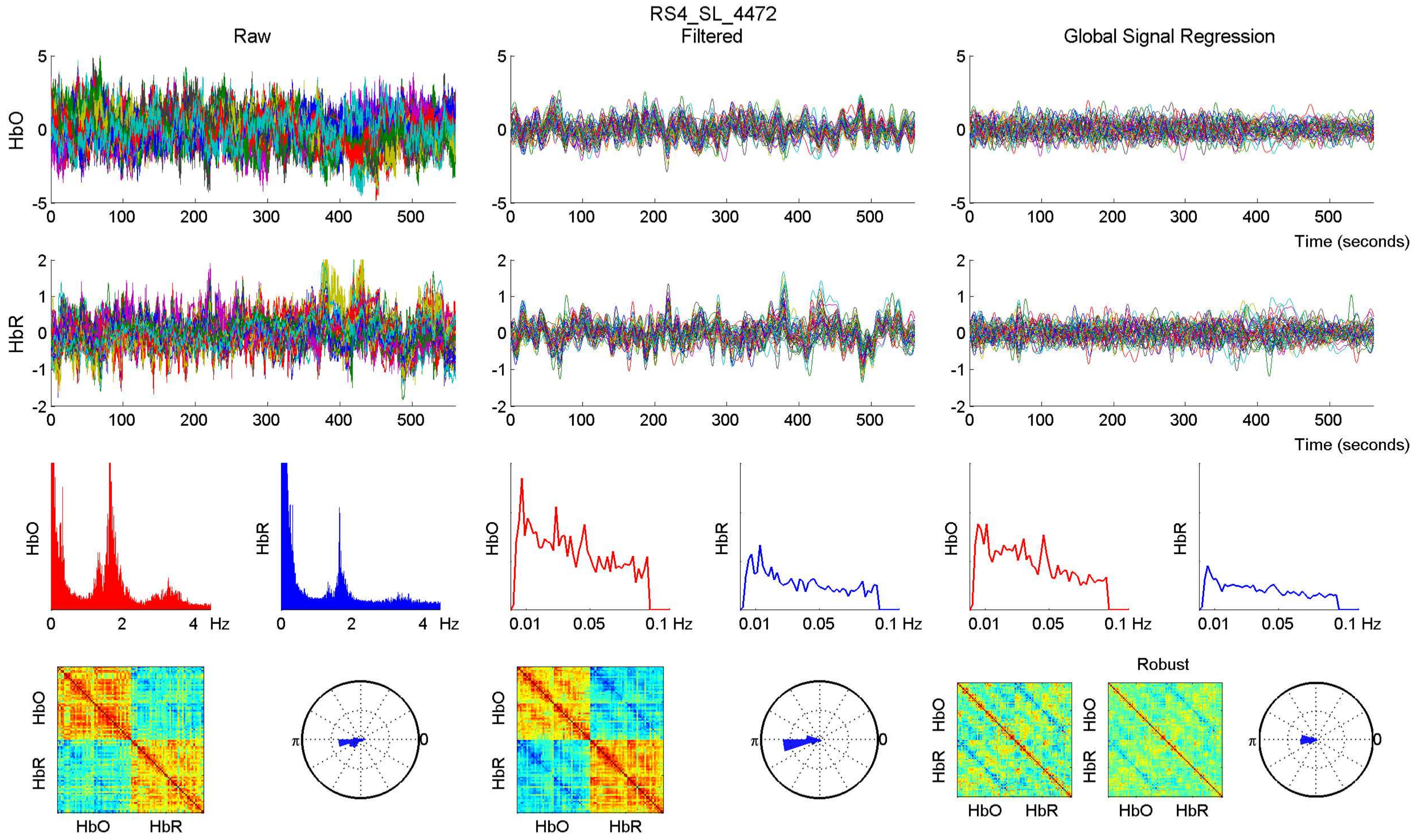

RS4_SL_4472
Raw
Filtered
Global Signal Regression
HbO
HbR
Time (seconds)
Hz
Robust

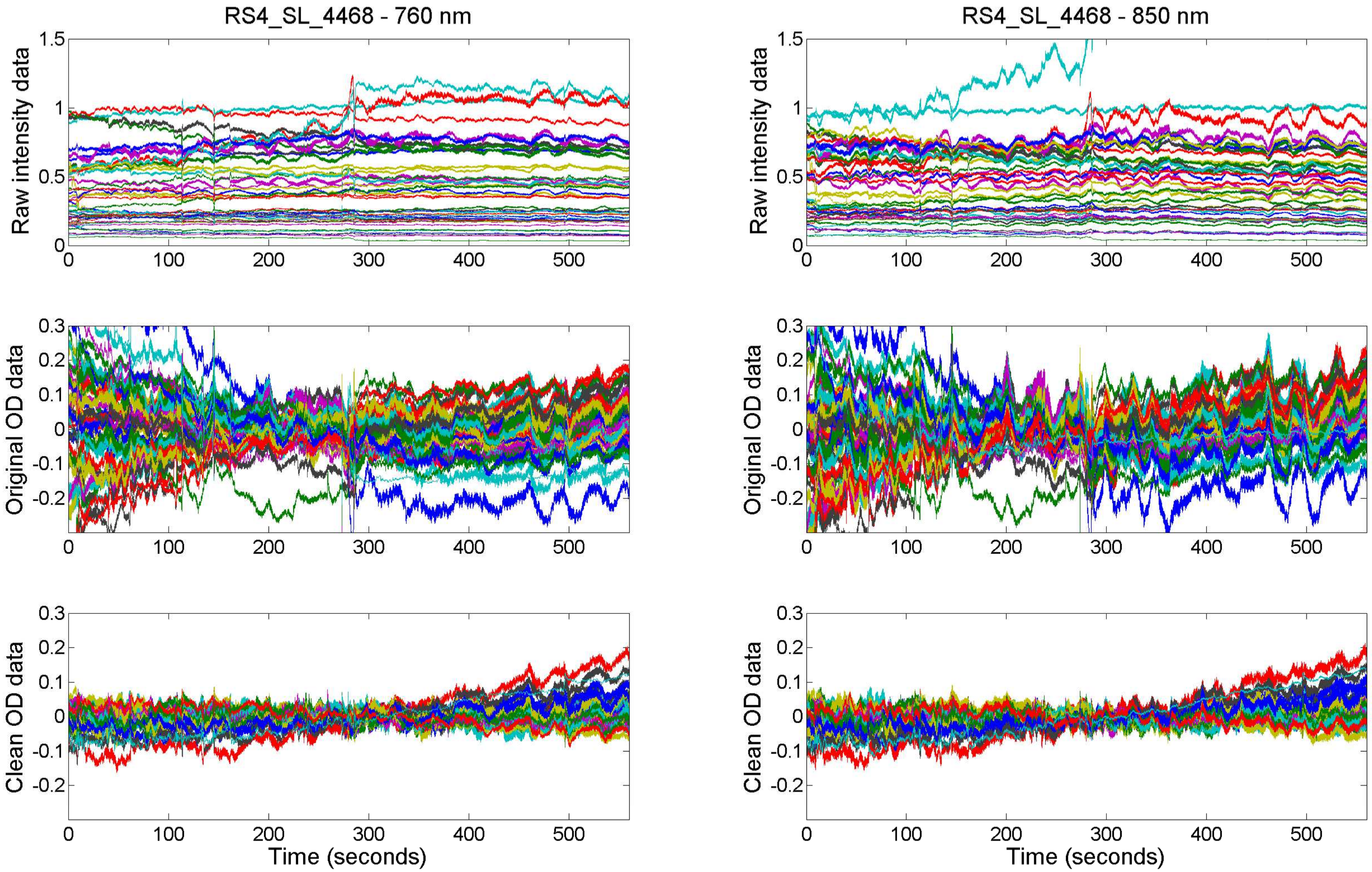

RS4_SL_4468 - 760 nm
RS4_SL_4468 - 850 nm
Raw intensity data
Original OD data
Clean OD data
Time (seconds)

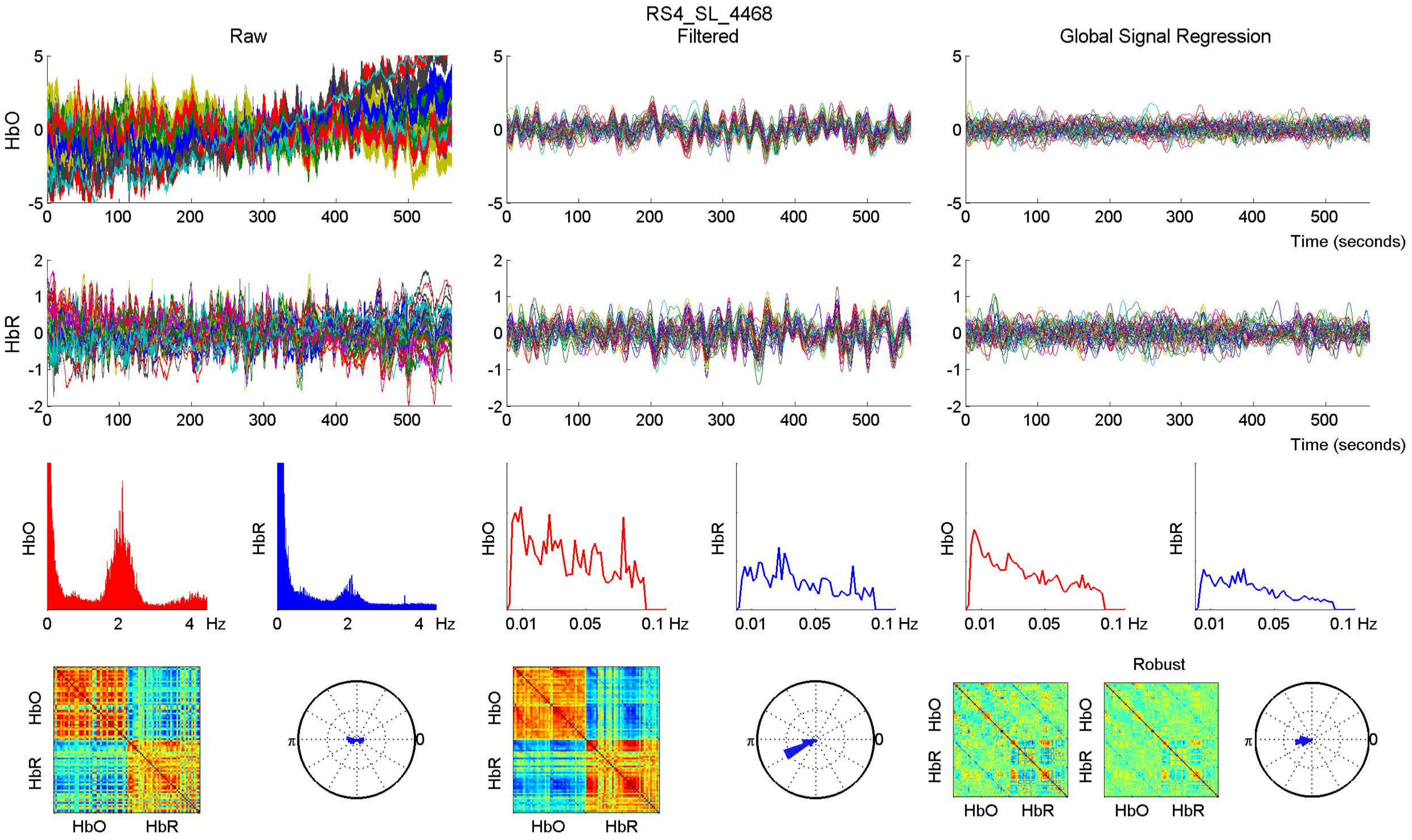

RS4_SL_4468
Raw
Filtered
Global Signal Regression
HbO
HbR
Time (seconds)
Hz
Robust

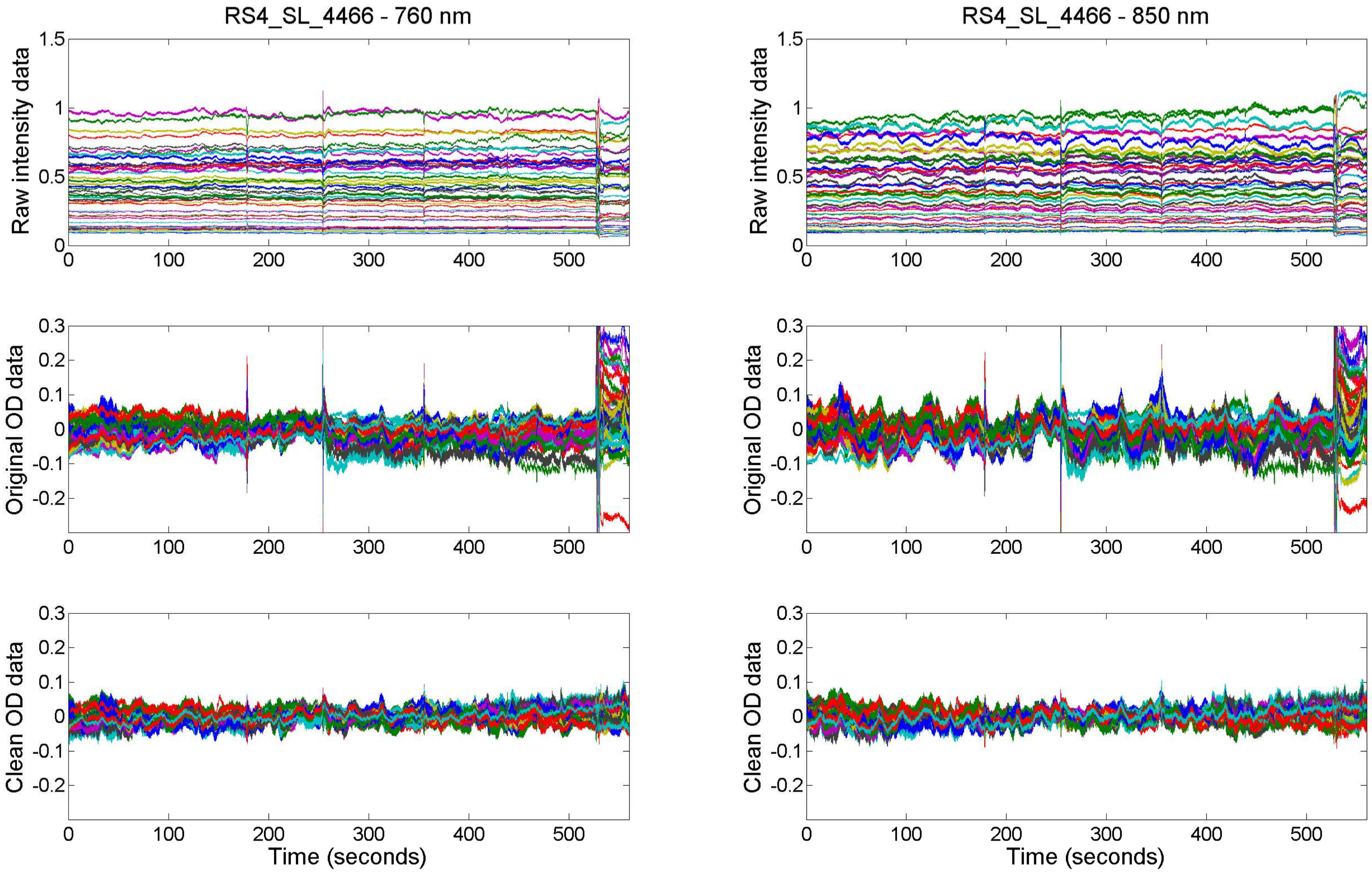
RS4_SL_4466 - 760 nm
RS4_SL_4466 - 850 nm
Raw intensity data
Original OD data
Clean OD data
Time (seconds)
1.5
1
0.5
0
0.3
0.2
0.1
-0.1
-0.2
100
200
300
400
500

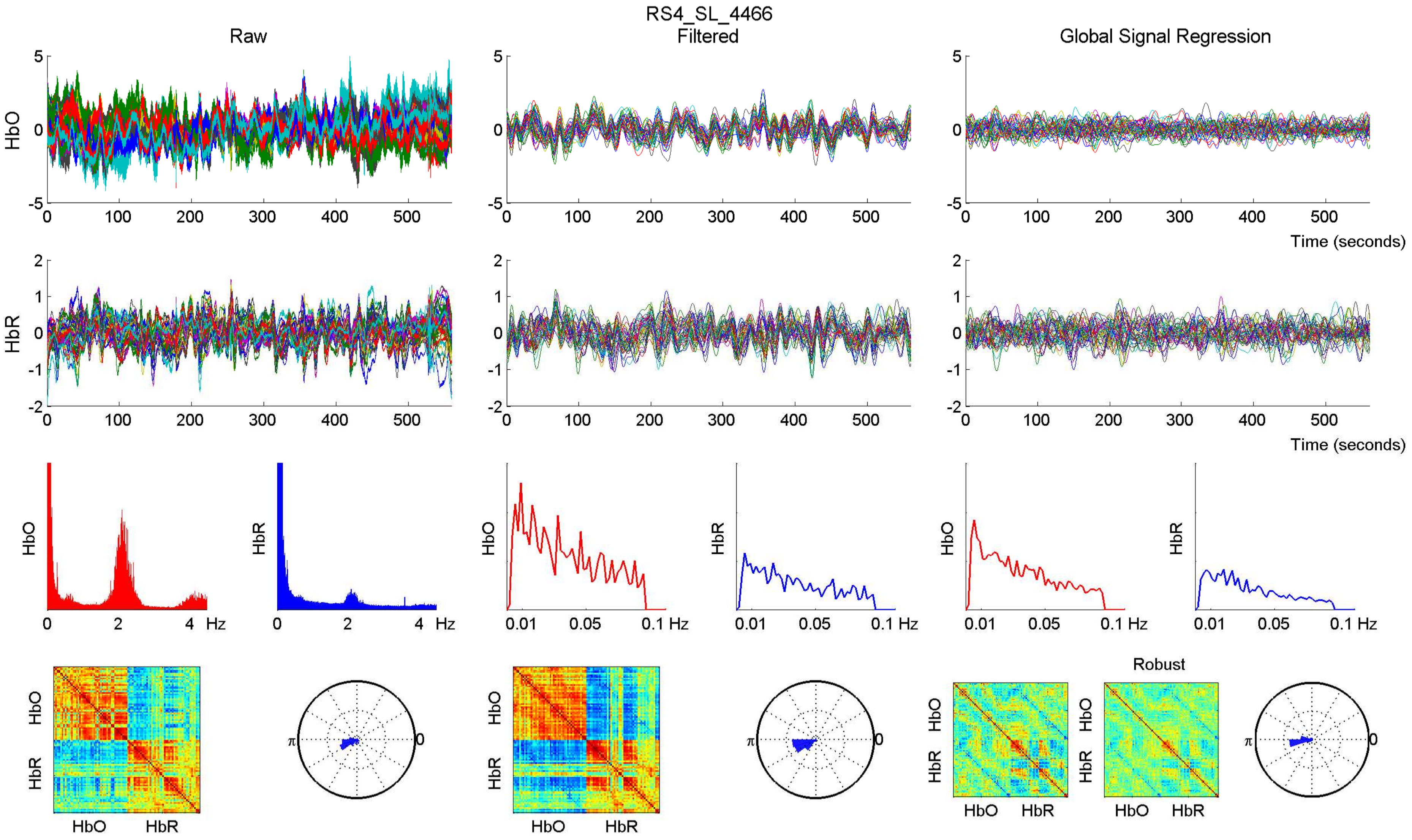
RS4_SL_4466
Raw
Filtered
Global Signal Regression
HbO
HbR
Time (seconds)
Hz
Robust

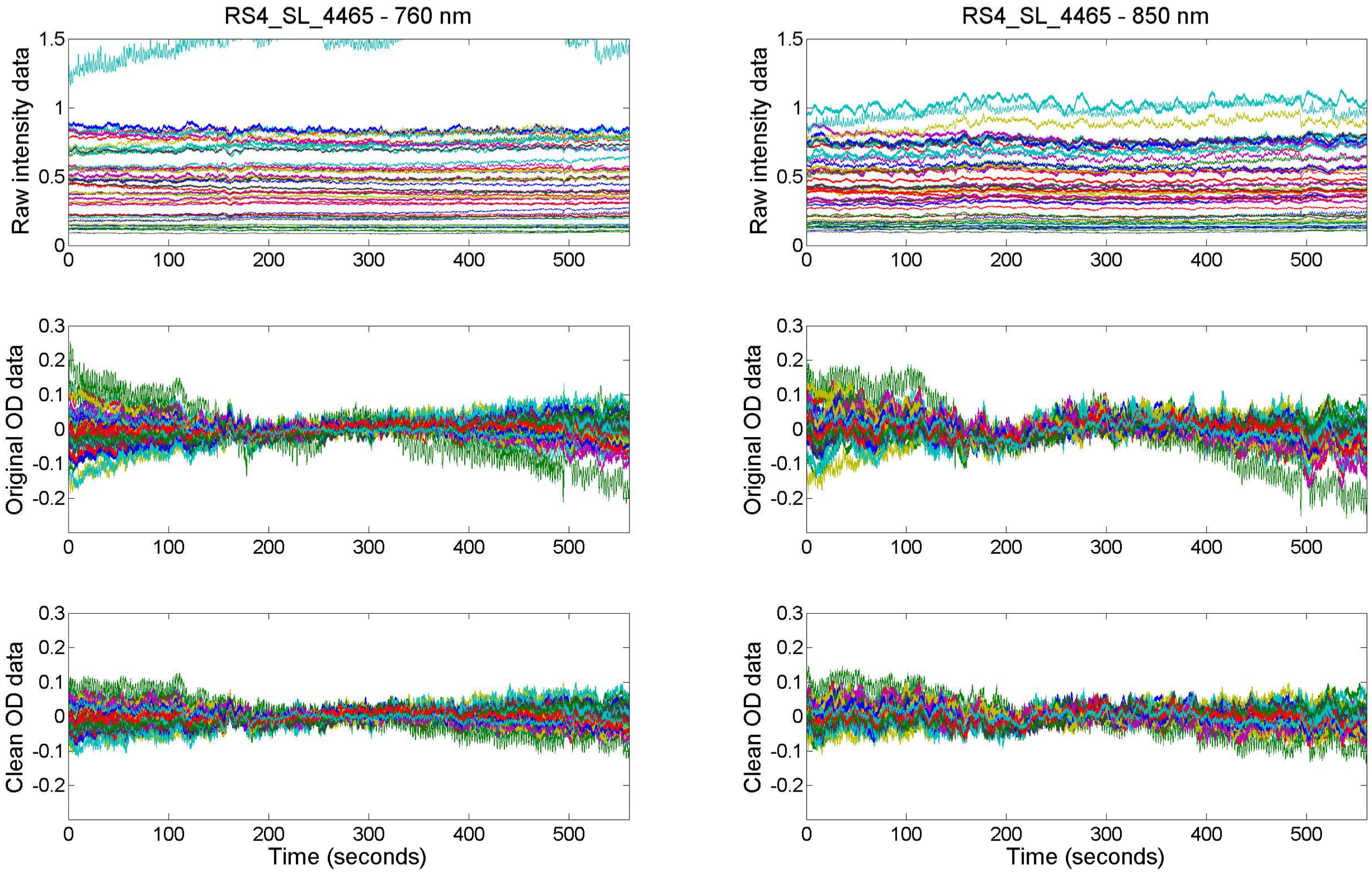

RS4_SL_4465 - 760 nm
RS4_SL_4465 - 850 nm
Raw intensity data
Original OD data
Clean OD data
Time (seconds)

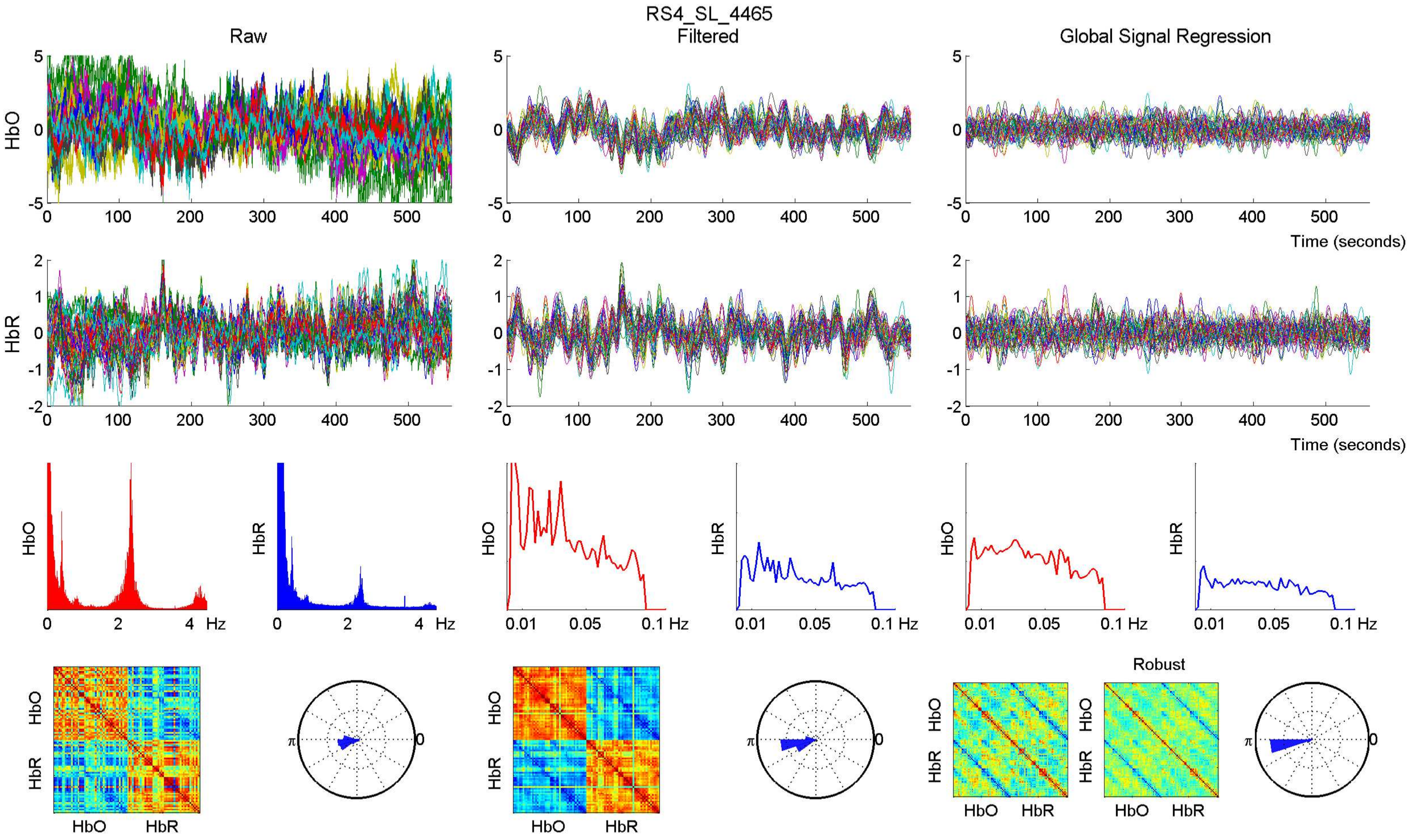
RS4_SL_4465
Raw
Filtered
Global Signal Regression
HbO
HbR
Time (seconds)
Hz
0.01
0.05
0.1 Hz
Robust
HbO
HbR
π
0

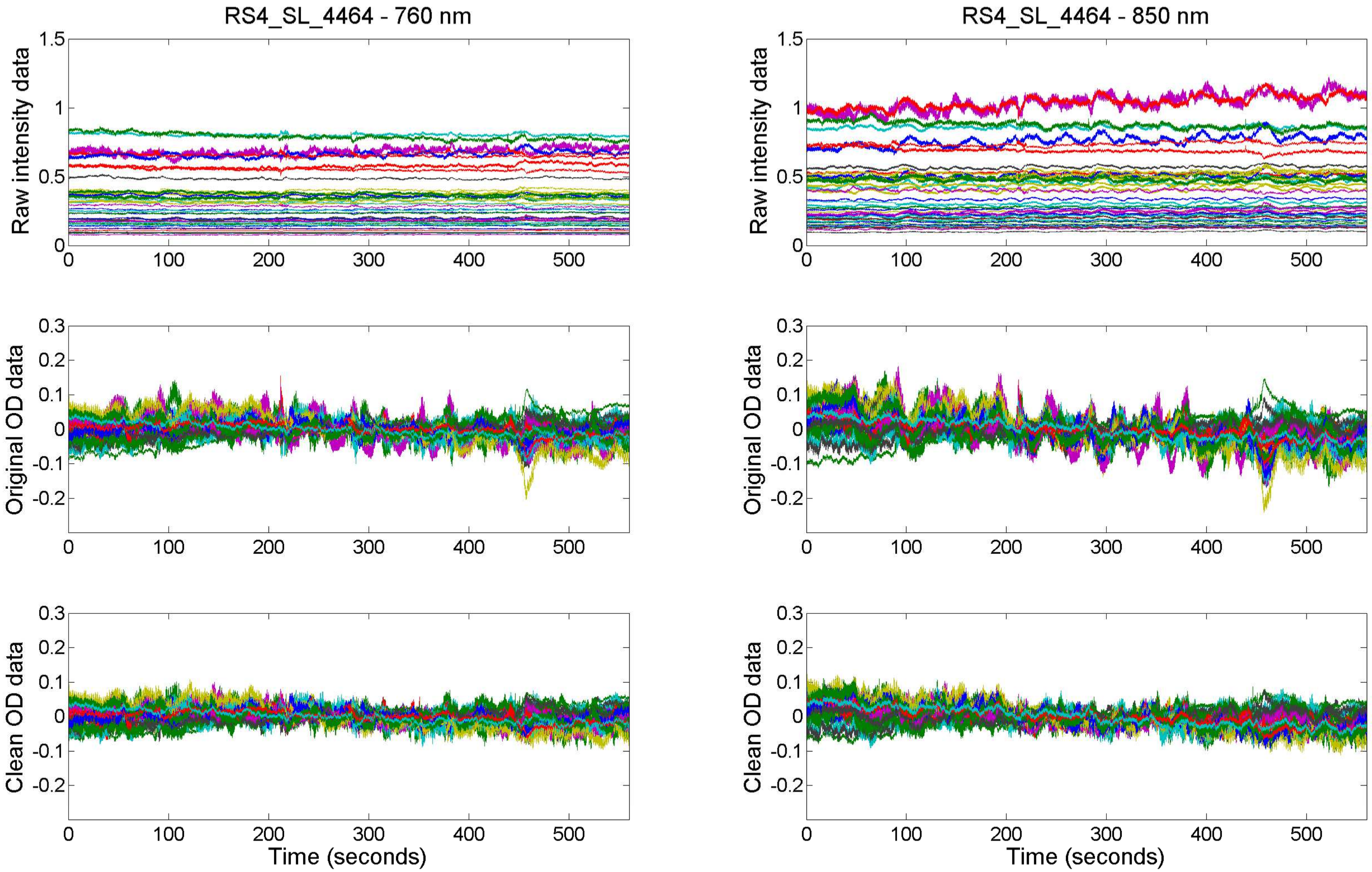
RS4_SL_4464 - 760 nm
RS4_SL_4464 - 850 nm
Raw intensity data
Original OD data
Clean OD data
Time (seconds)

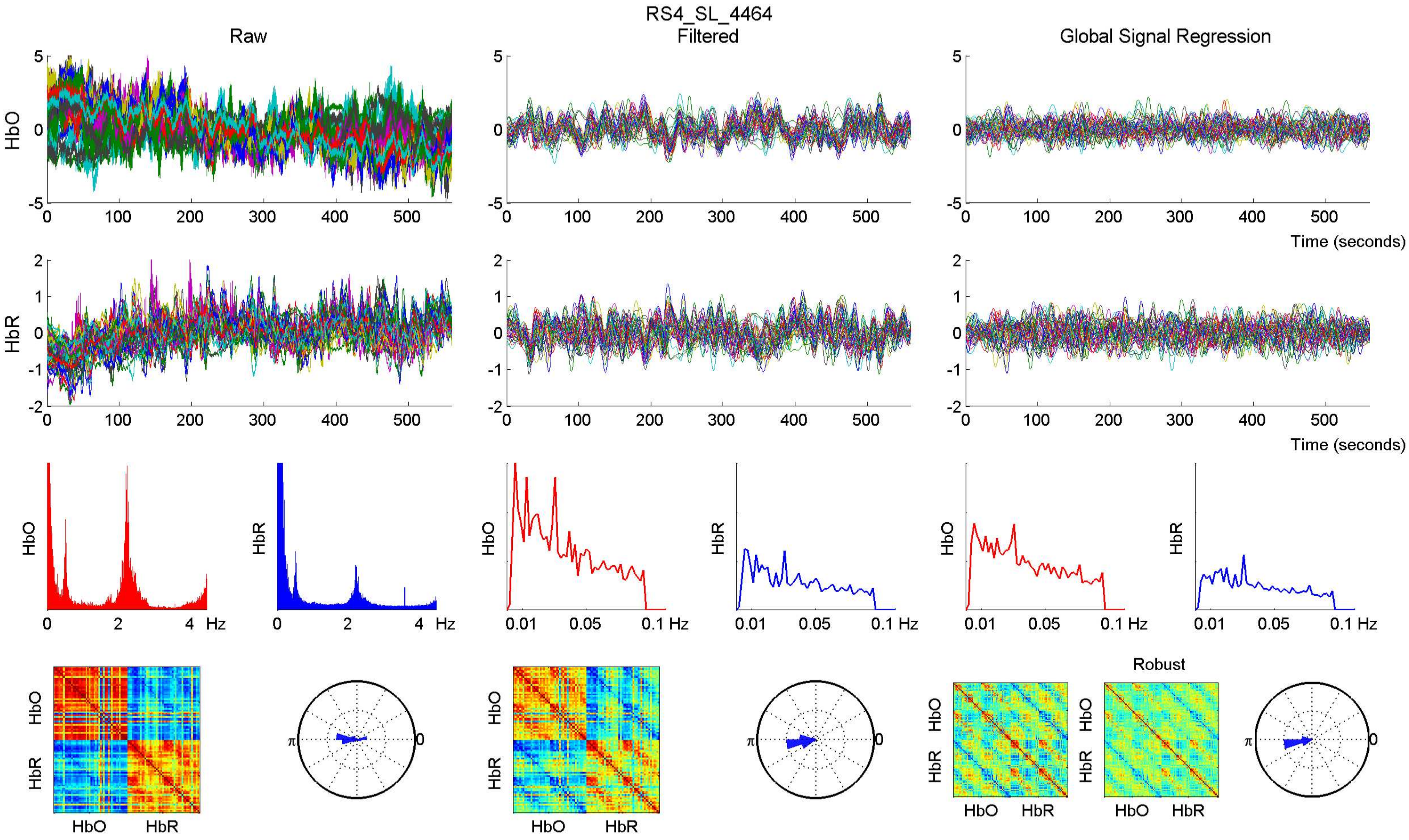

RS4_SL_4464
Raw
Filtered
Global Signal Regression
HbO
HbR
Time (seconds)
Hz
Robust

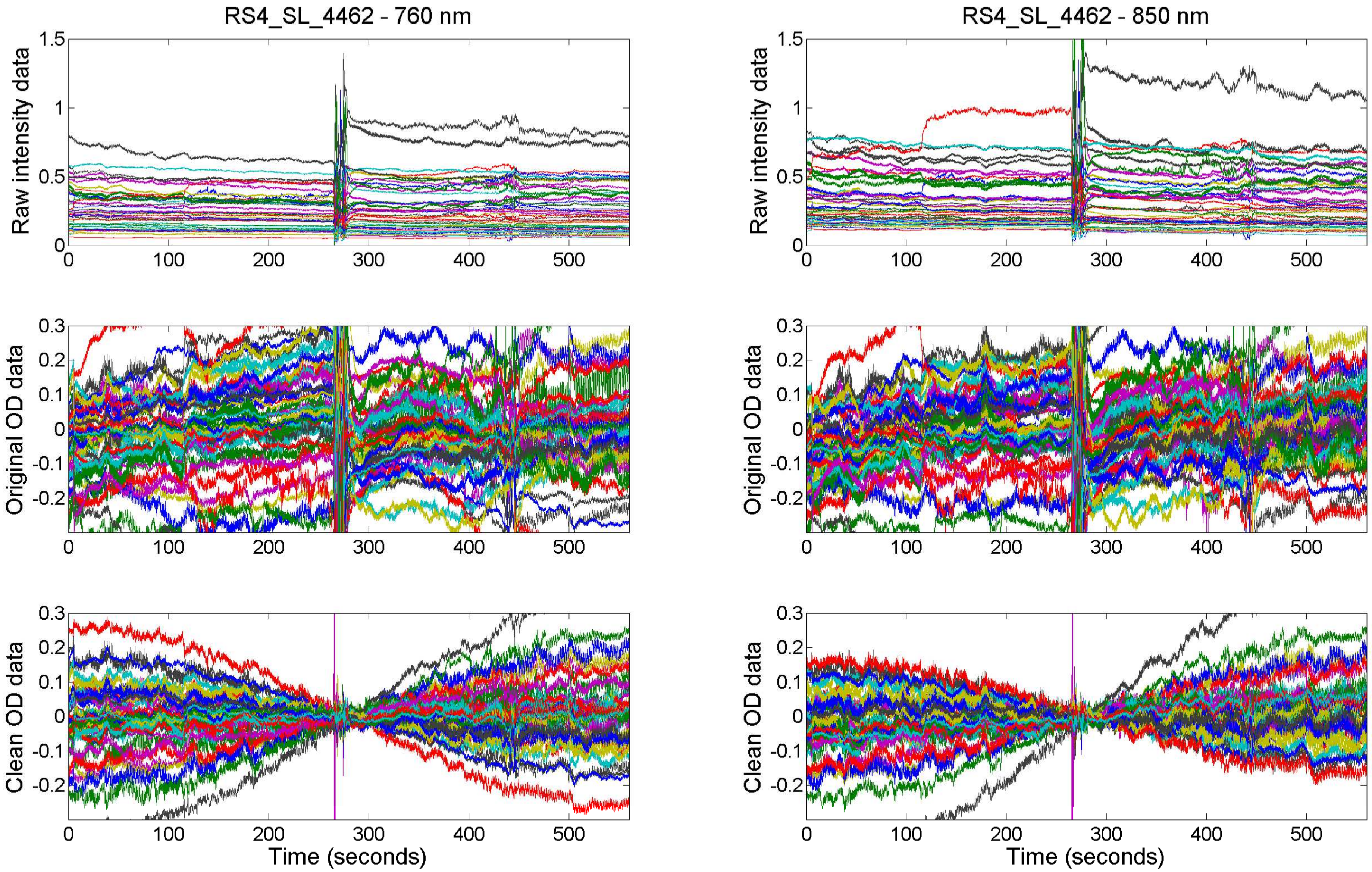

RS4_SL_4462 - 760 nm
RS4_SL_4462 - 850 nm
Raw intensity data
Original OD data
Clean OD data
Time (seconds)

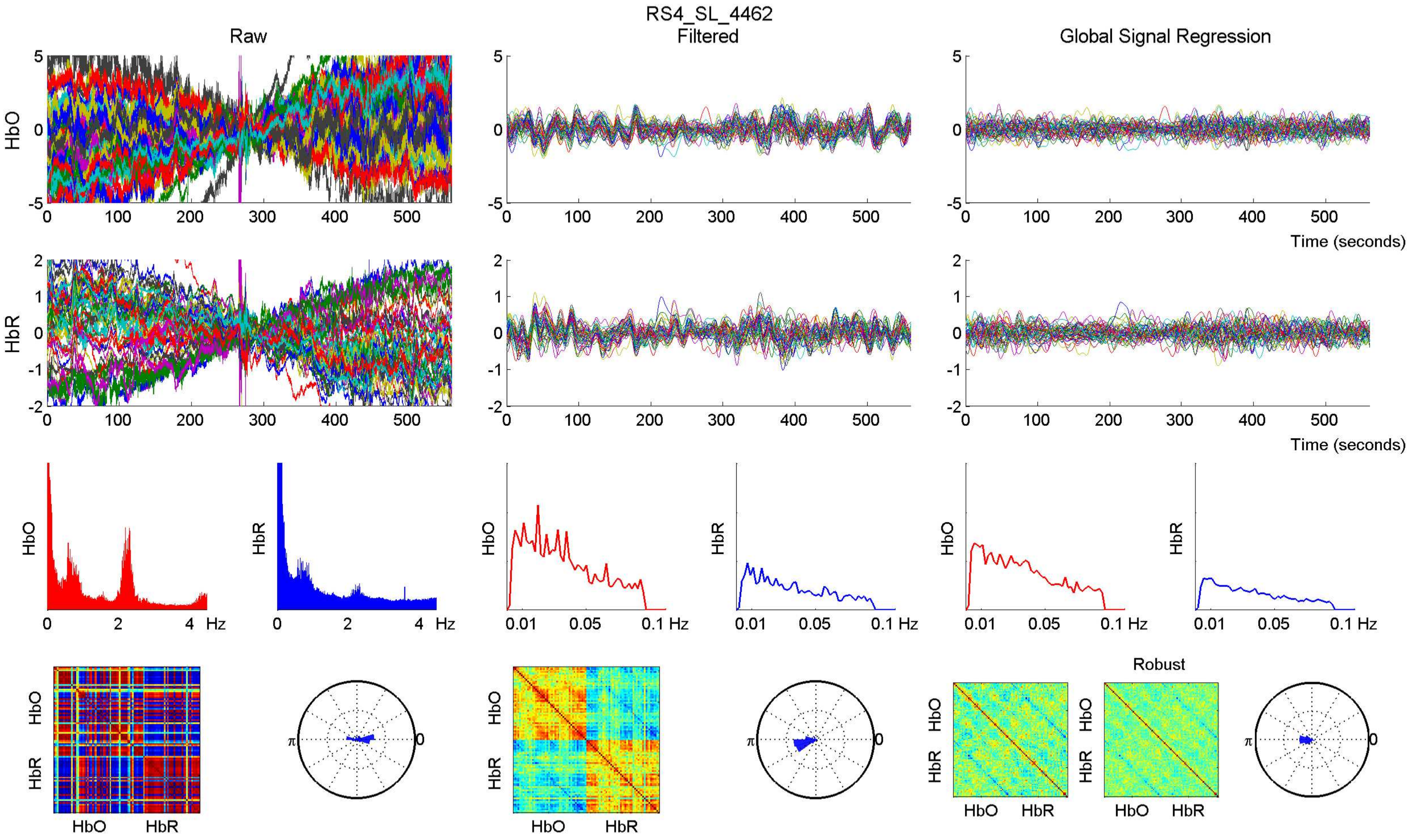
RS4_SL_4462
Raw
Filtered
Global Signal Regression
HbO
HbR
Time (seconds)
Hz
Robust

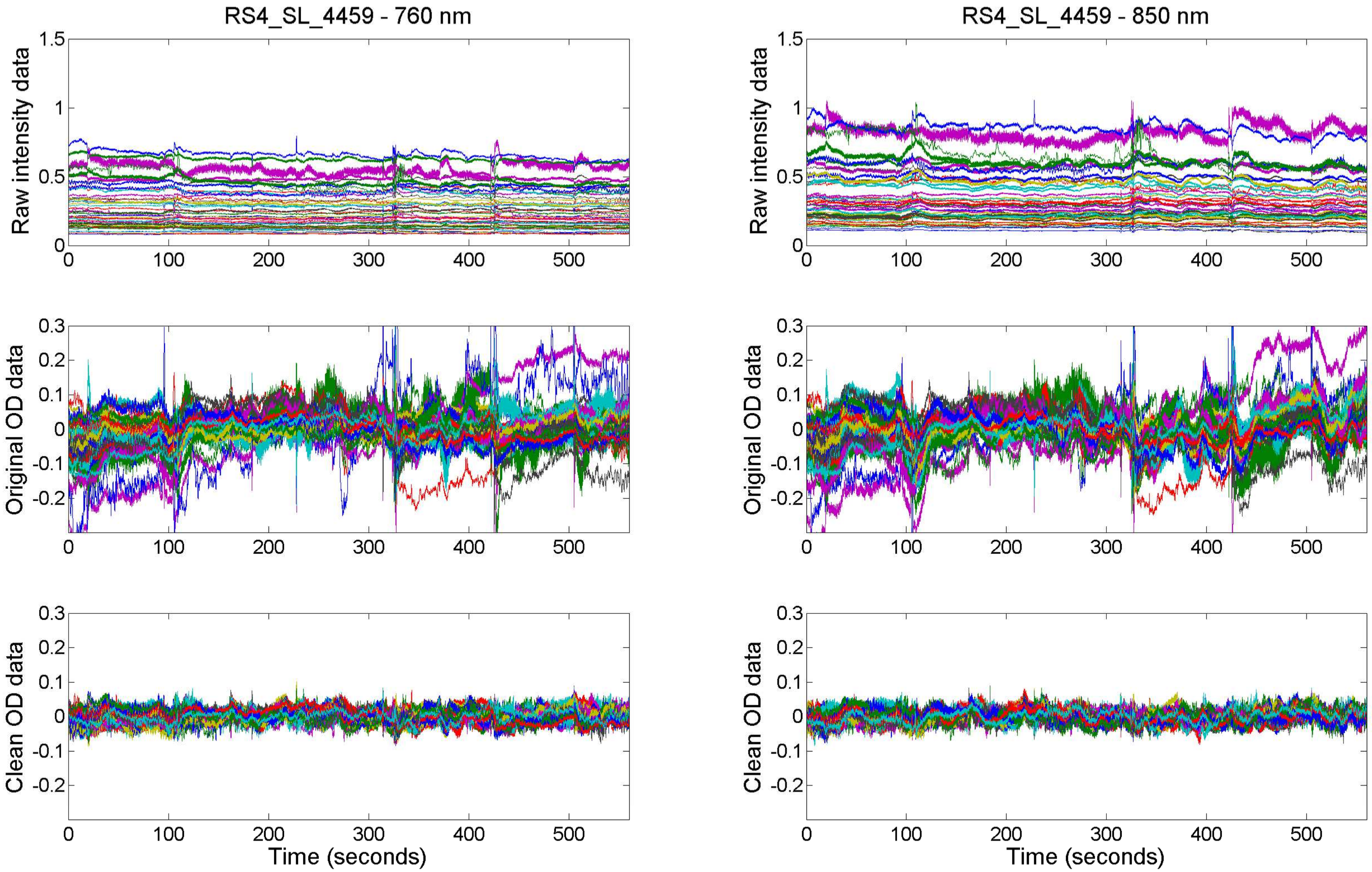

RS4_SL_4459 - 760 nm
RS4_SL_4459 - 850 nm
Raw intensity data
Original OD data
Clean OD data
Time (seconds)

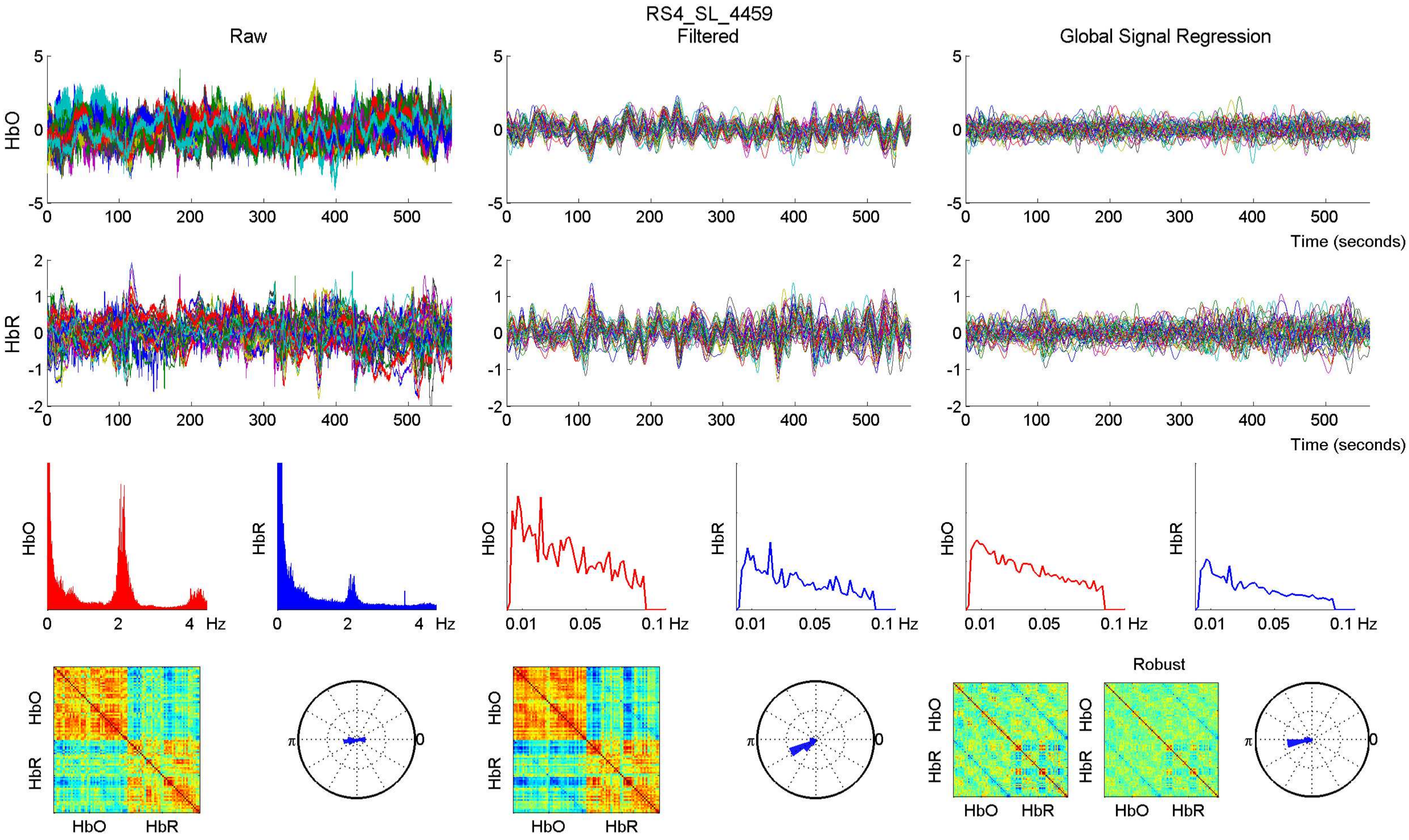

RS4_SL_4459
Raw
Filtered
Global Signal Regression
HbO
HbR
Time (seconds)
Hz
Robust
HbO
HbR

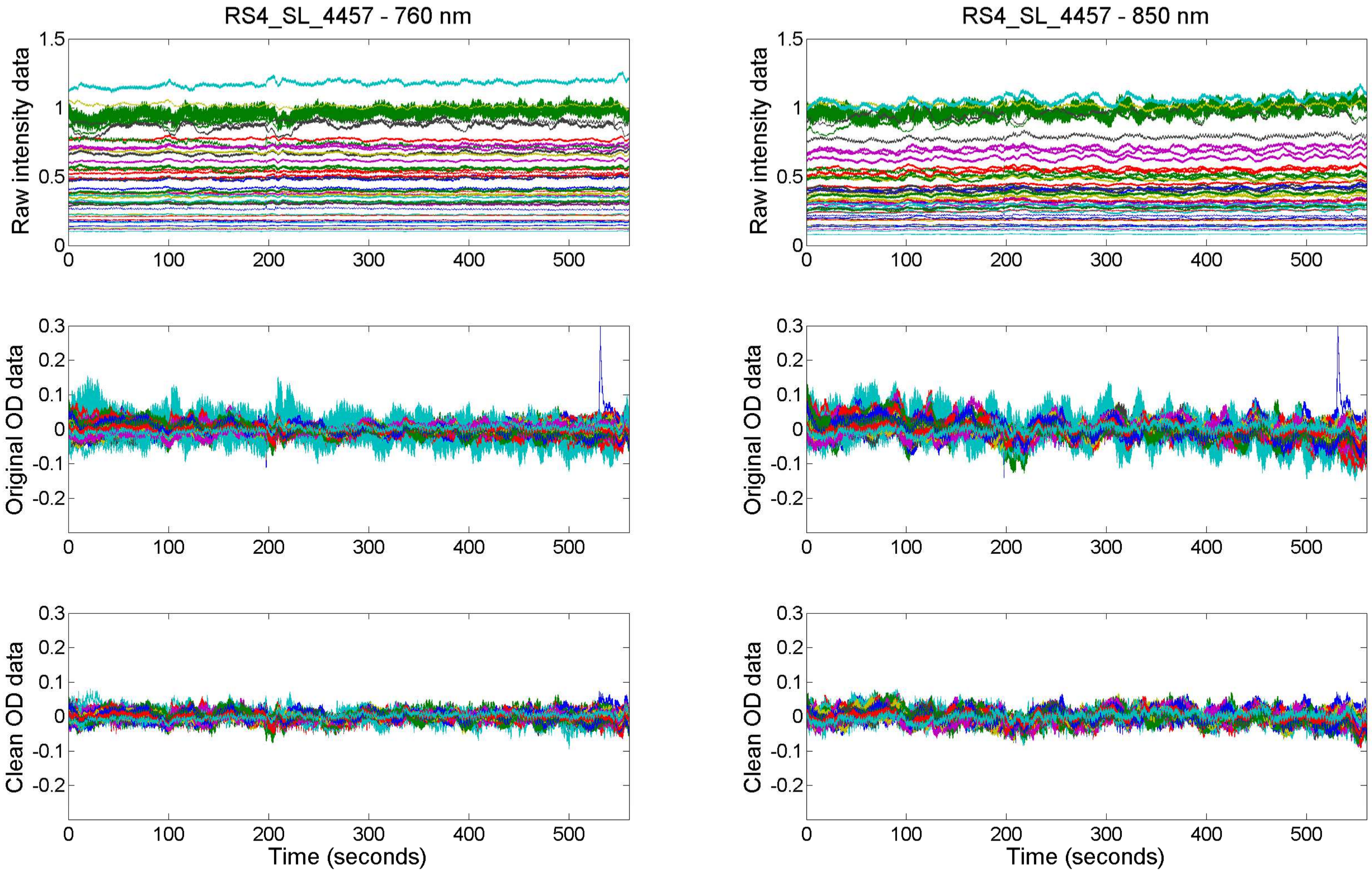

RS4_SL_4457 - 760 nm
RS4_SL_4457 - 850 nm
Raw intensity data
Original OD data
Clean OD data
Time (seconds)

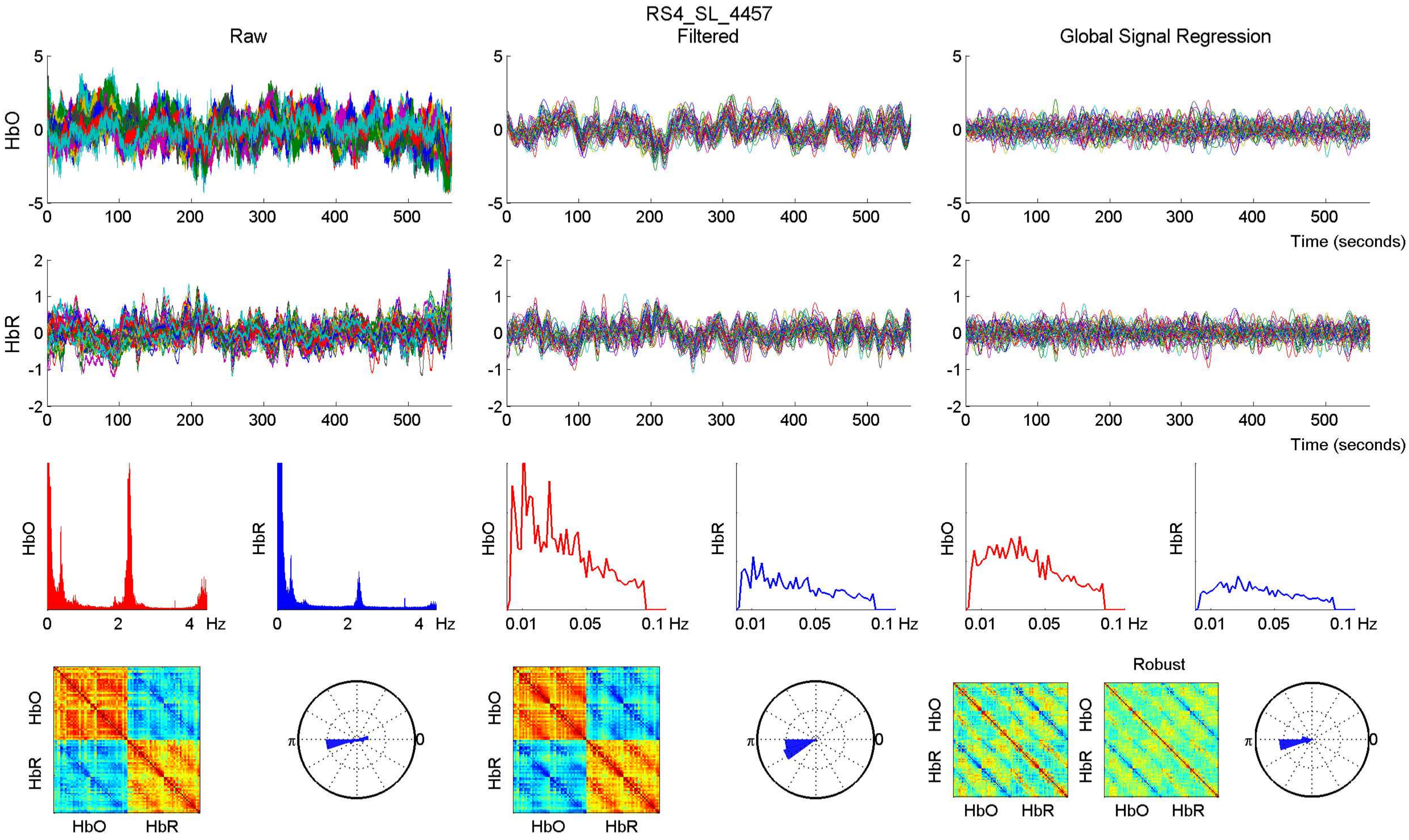

RS4_SL_4457
Raw
Filtered
Global Signal Regression
HbO
HbR
Time (seconds)
Hz
Robust

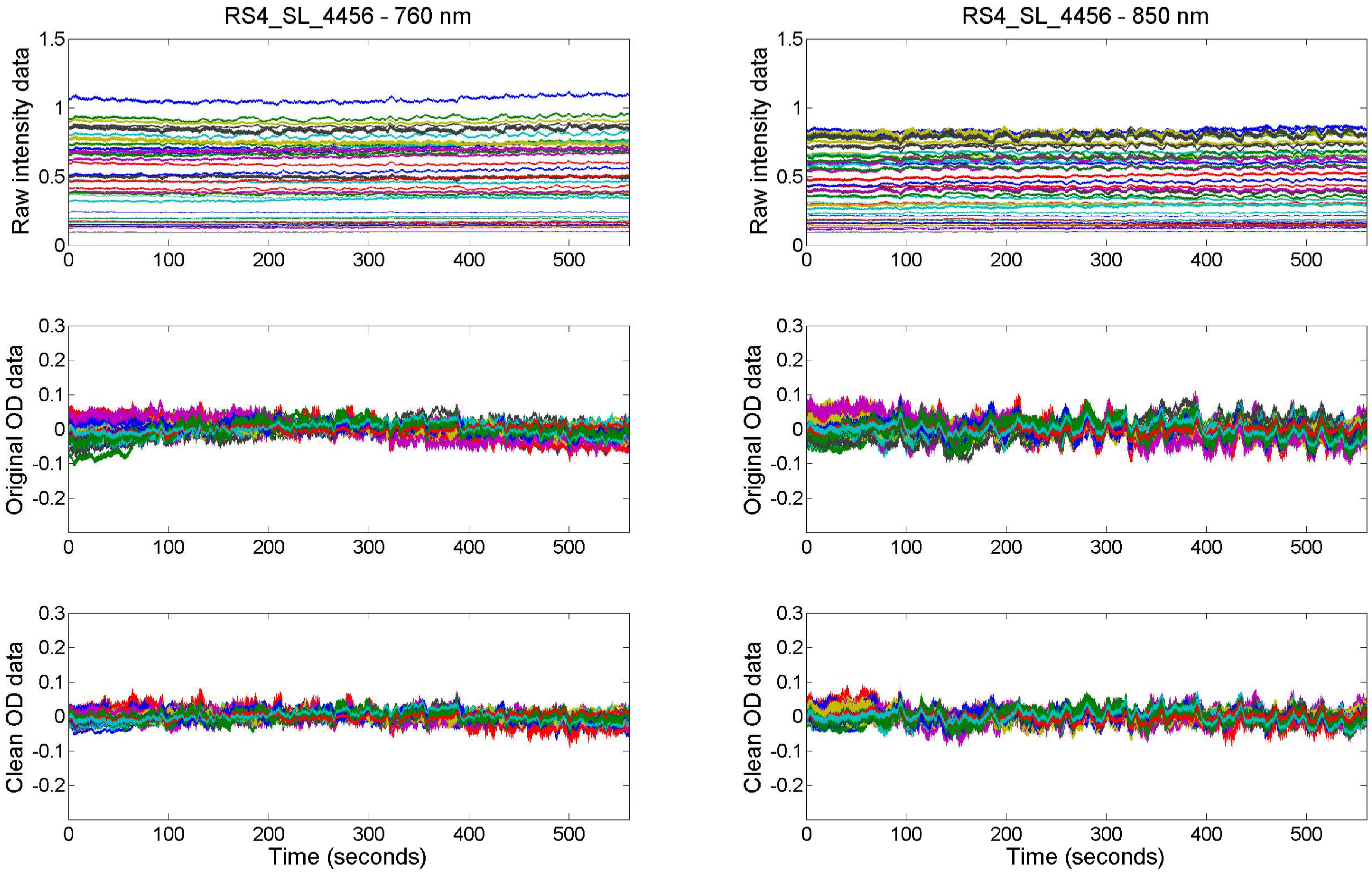

RS4_SL_4456 - 760 nm
RS4_SL_4456 - 850 nm
Raw intensity data
Original OD data
Clean OD data
Time (seconds)

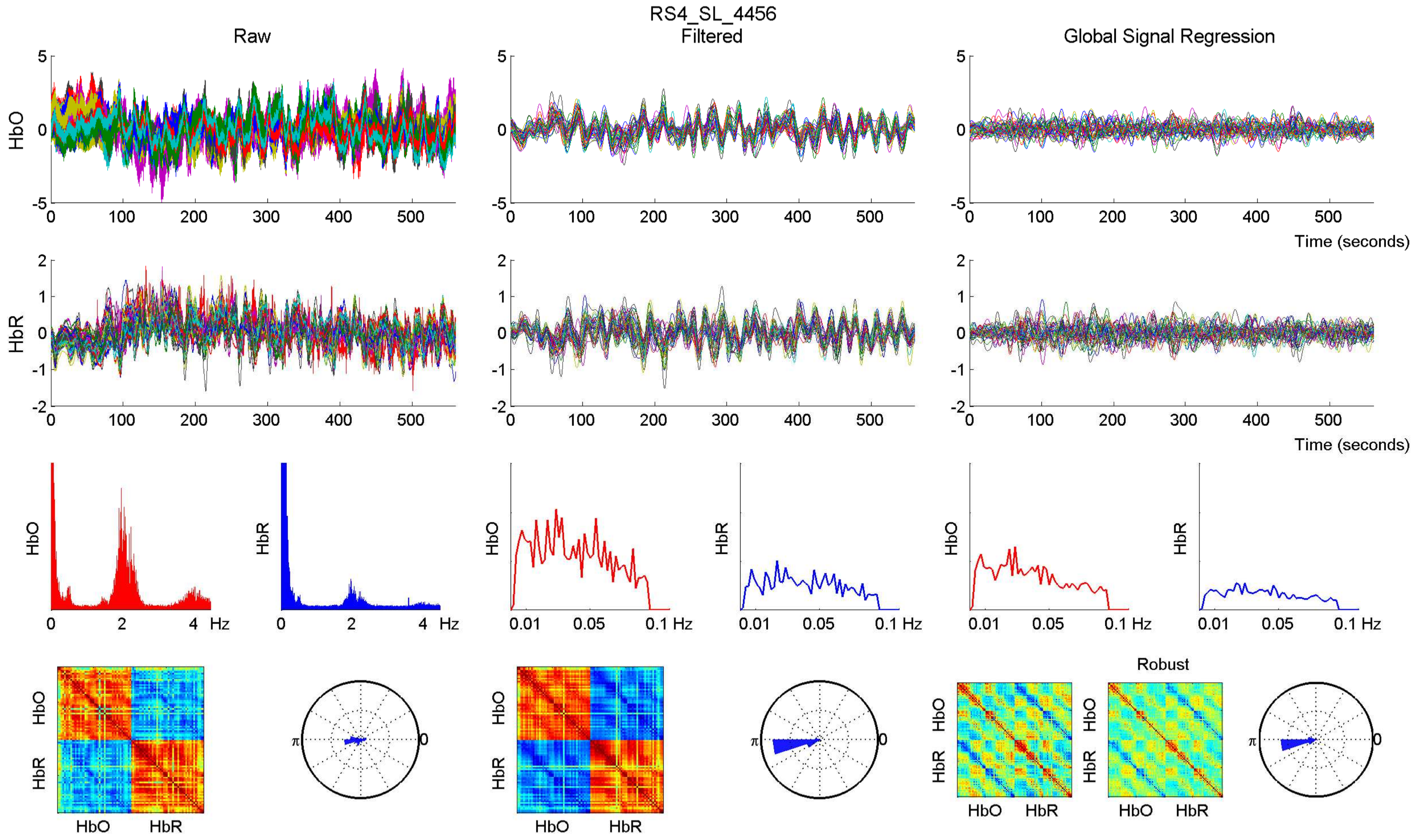
RS4_SL_4456
Raw
Filtered
Global Signal Regression
HbO
HbR
Time (seconds)
Hz
Robust
HbO
HbR

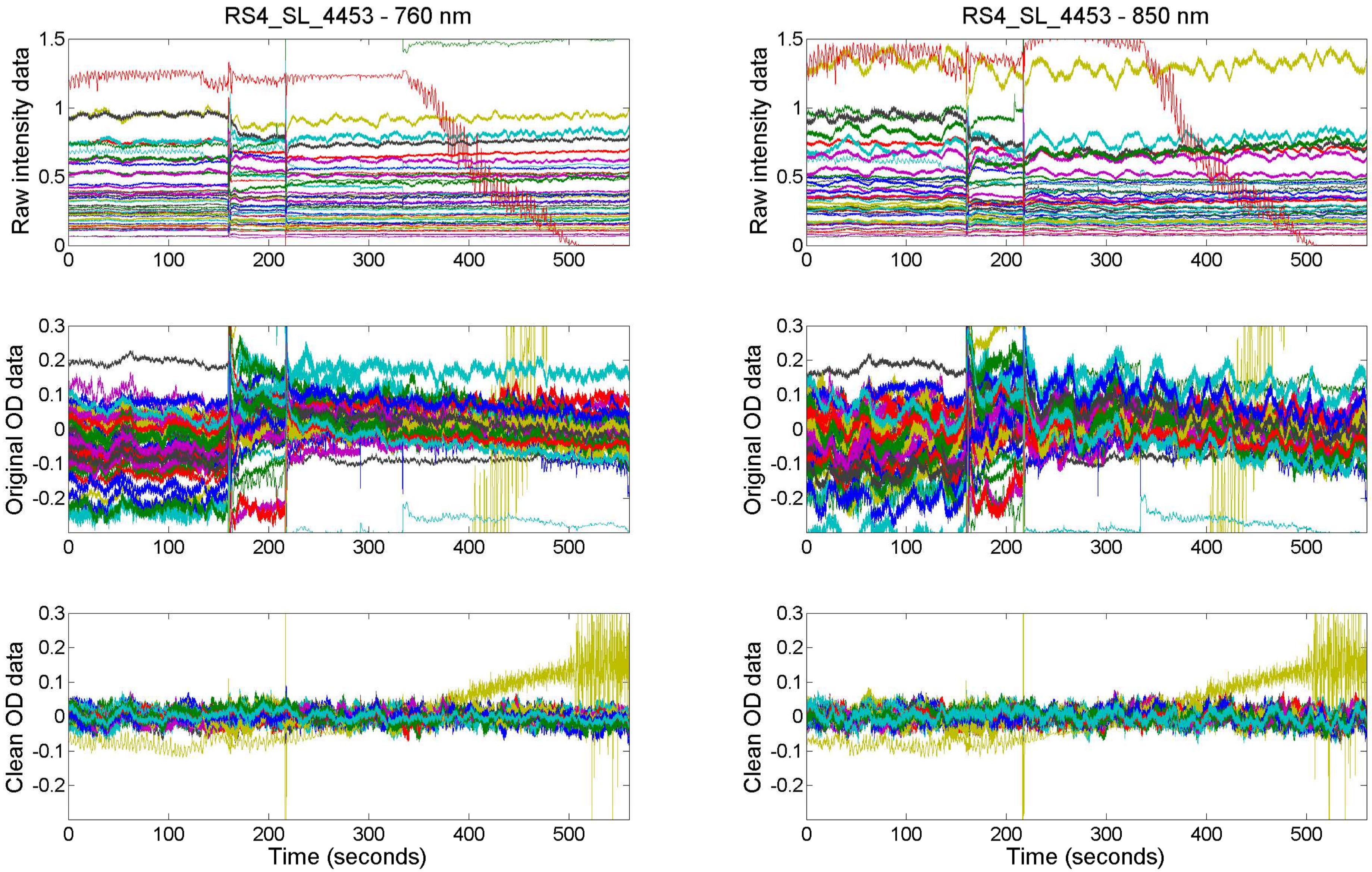

RS4_SL_4453 - 760 nm
RS4_SL_4453 - 850 nm
Raw intensity data
Original OD data
Clean OD data
Time (seconds)

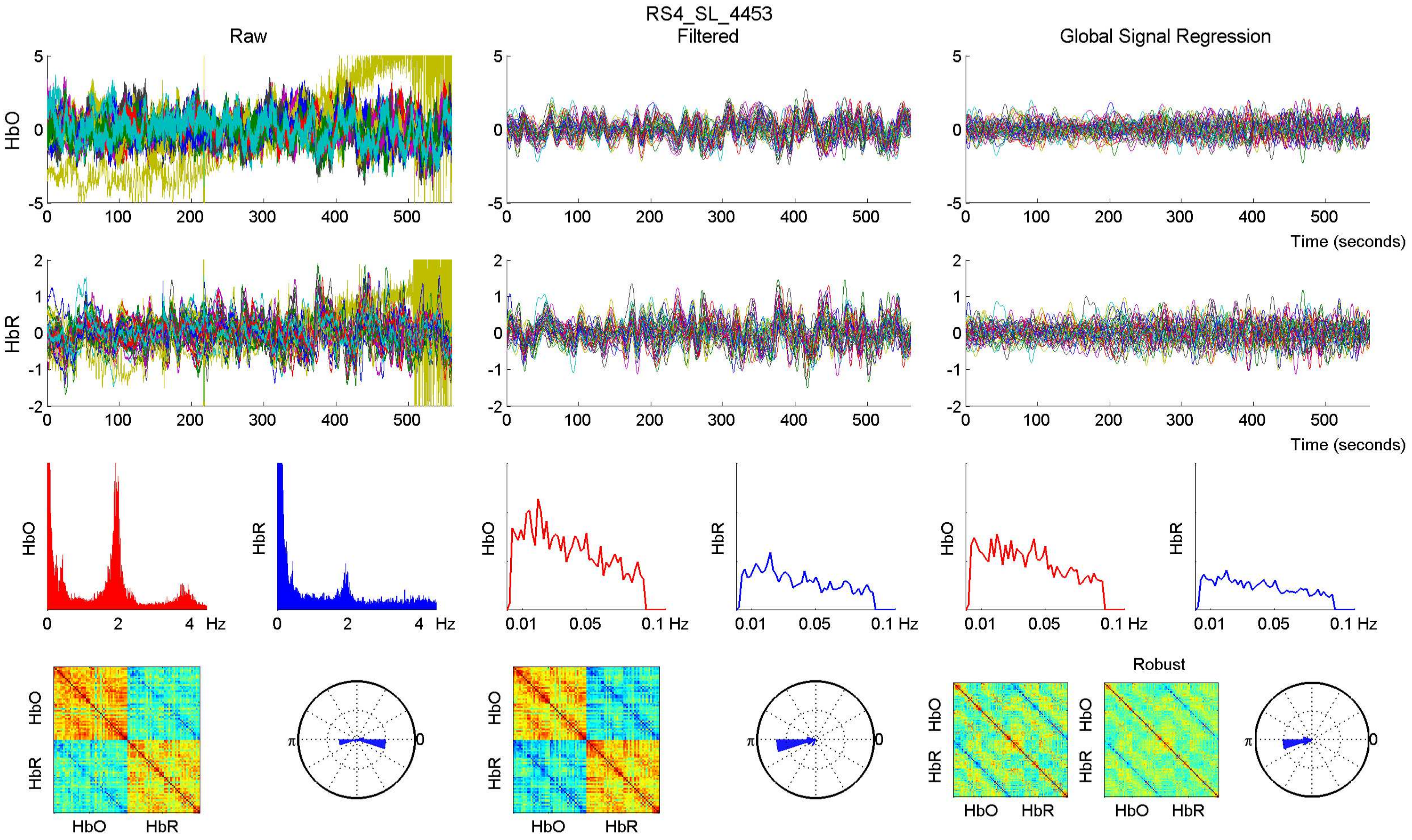

RS4_SL_4453
Raw
Filtered
Global Signal Regression
HbO
HbR
Time (seconds)
Hz
Robust

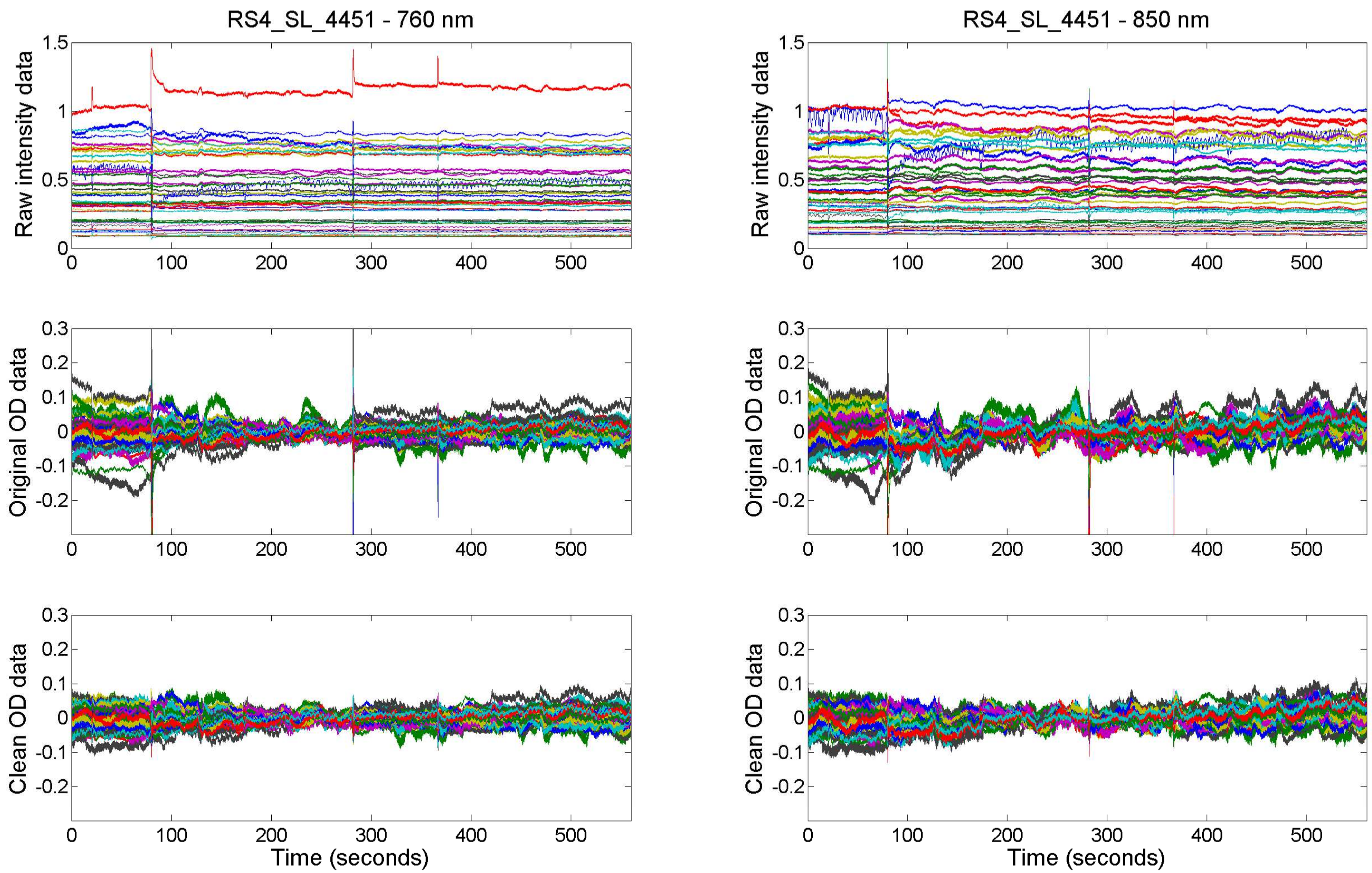

RS4_SL_4451 - 760 nm
RS4_SL_4451 - 850 nm
Raw intensity data
Original OD data
Clean OD data
Time (seconds)

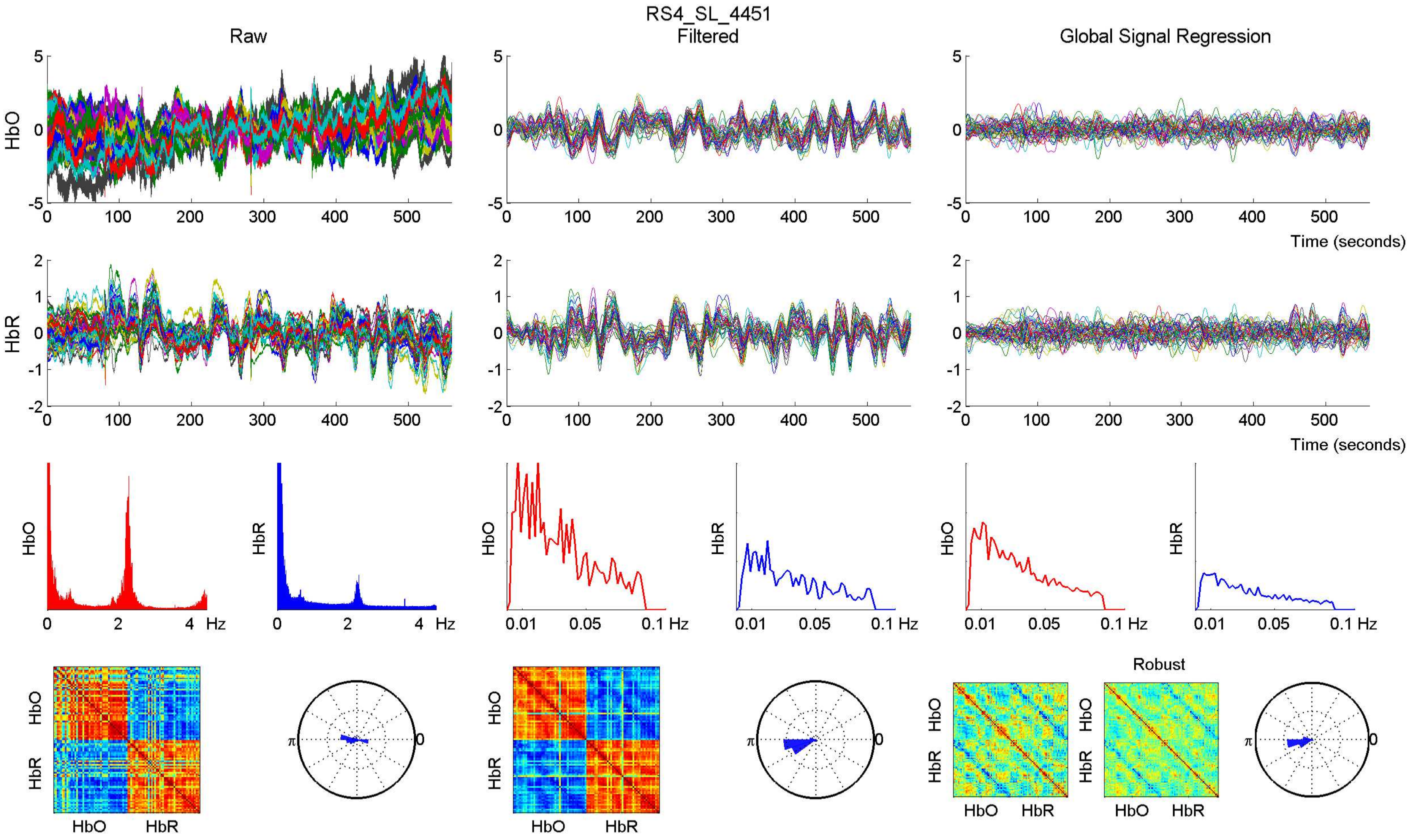

RS4_SL_4451
Raw
Filtered
Global Signal Regression
HbO
HbR
Time (seconds)
Hz
0.01
0.05
0.1 Hz
Robust
HbO
HbR
π
0

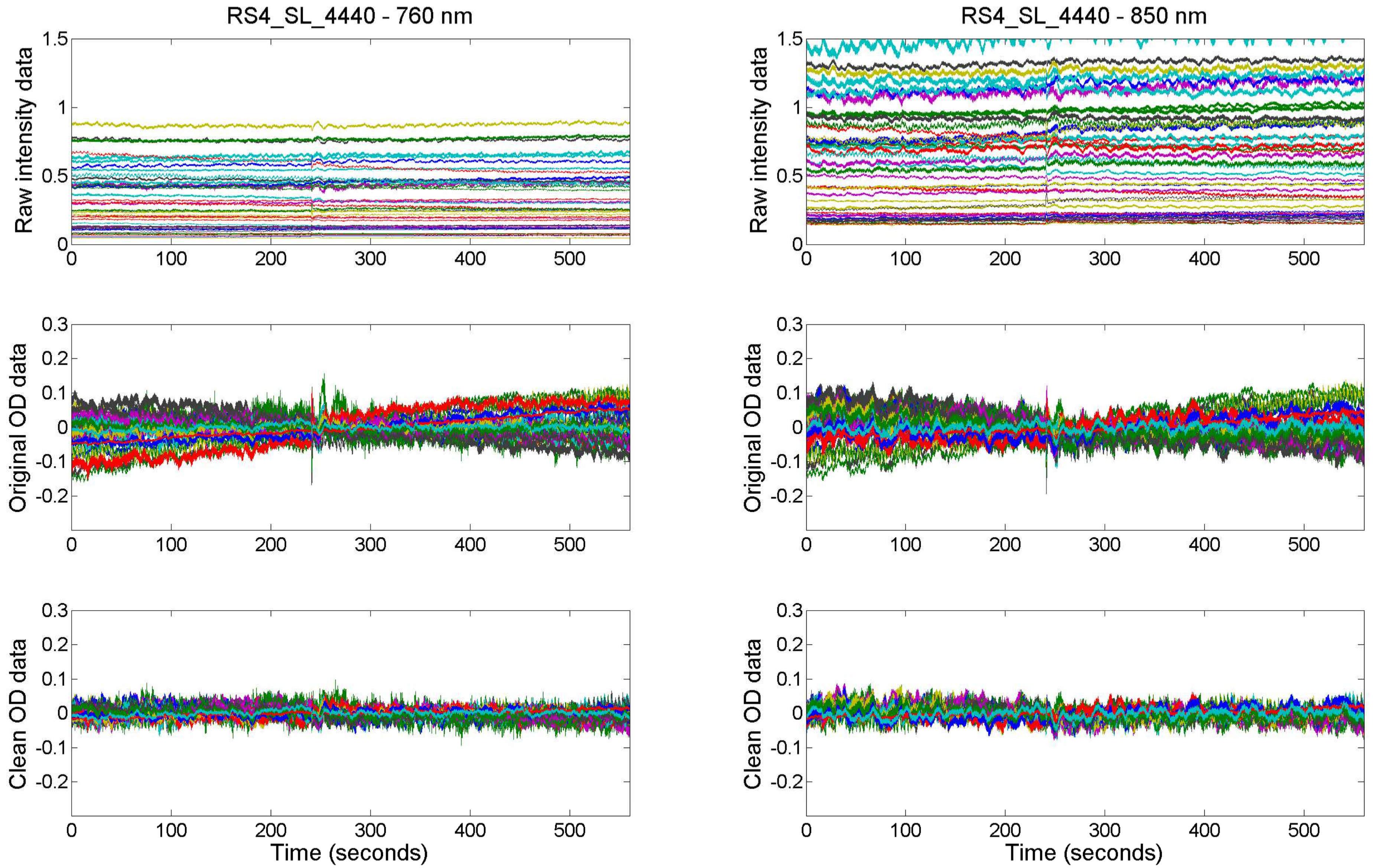
RS4_SL_4440 - 760 nm
RS4_SL_4440 - 850 nm
Raw intensity data
Original OD data
Clean OD data
Time (seconds)
0
100
200
300
400
500
1.5
1
0.5
0.3
0.2
0.1
-0.1
-0.2

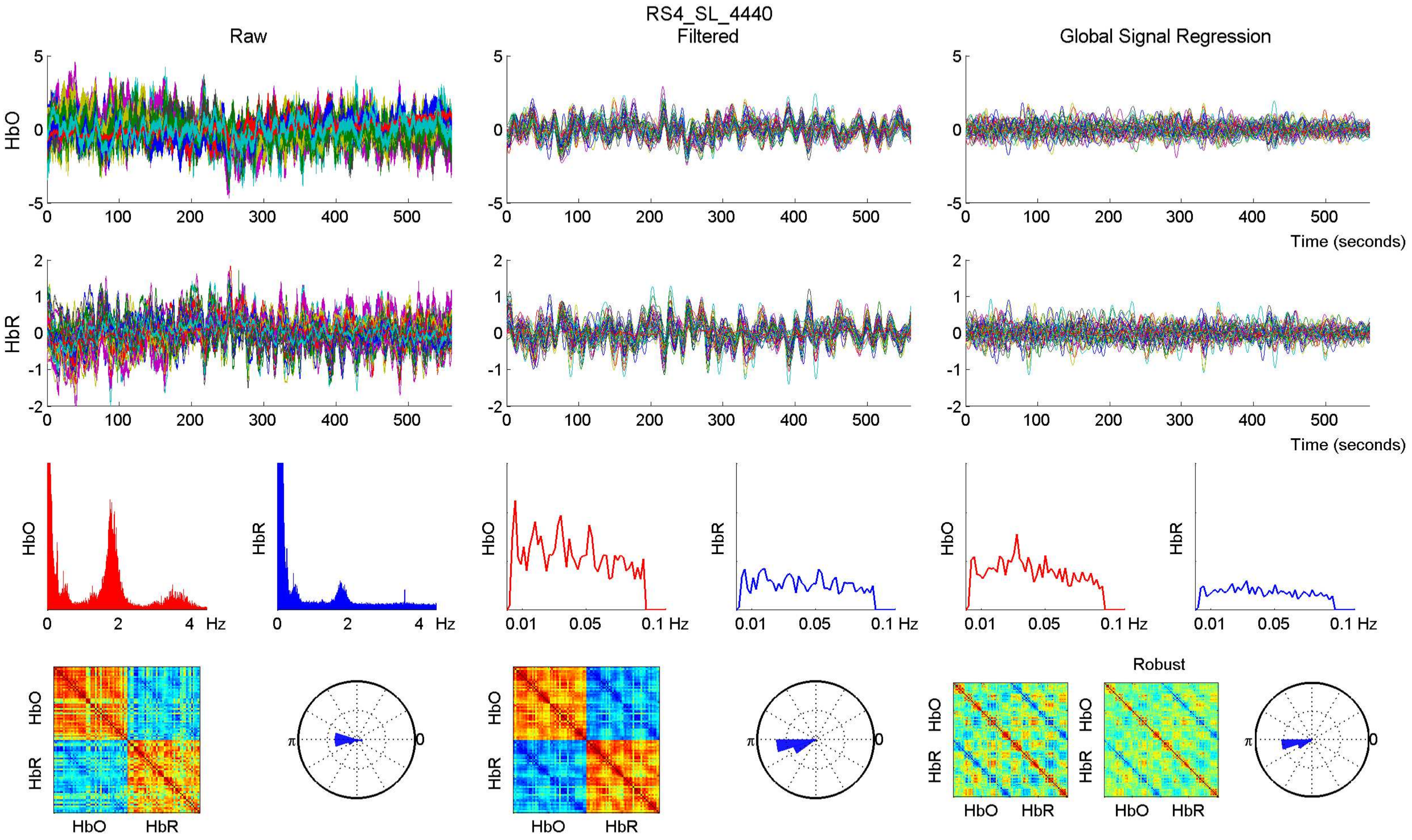

RS4_SL_4440
Raw
Filtered
Global Signal Regression
HbO
HbR
Time (seconds)
Hz
0.01
0.05
0.1 Hz
Robust
HbO
HbR
π
0

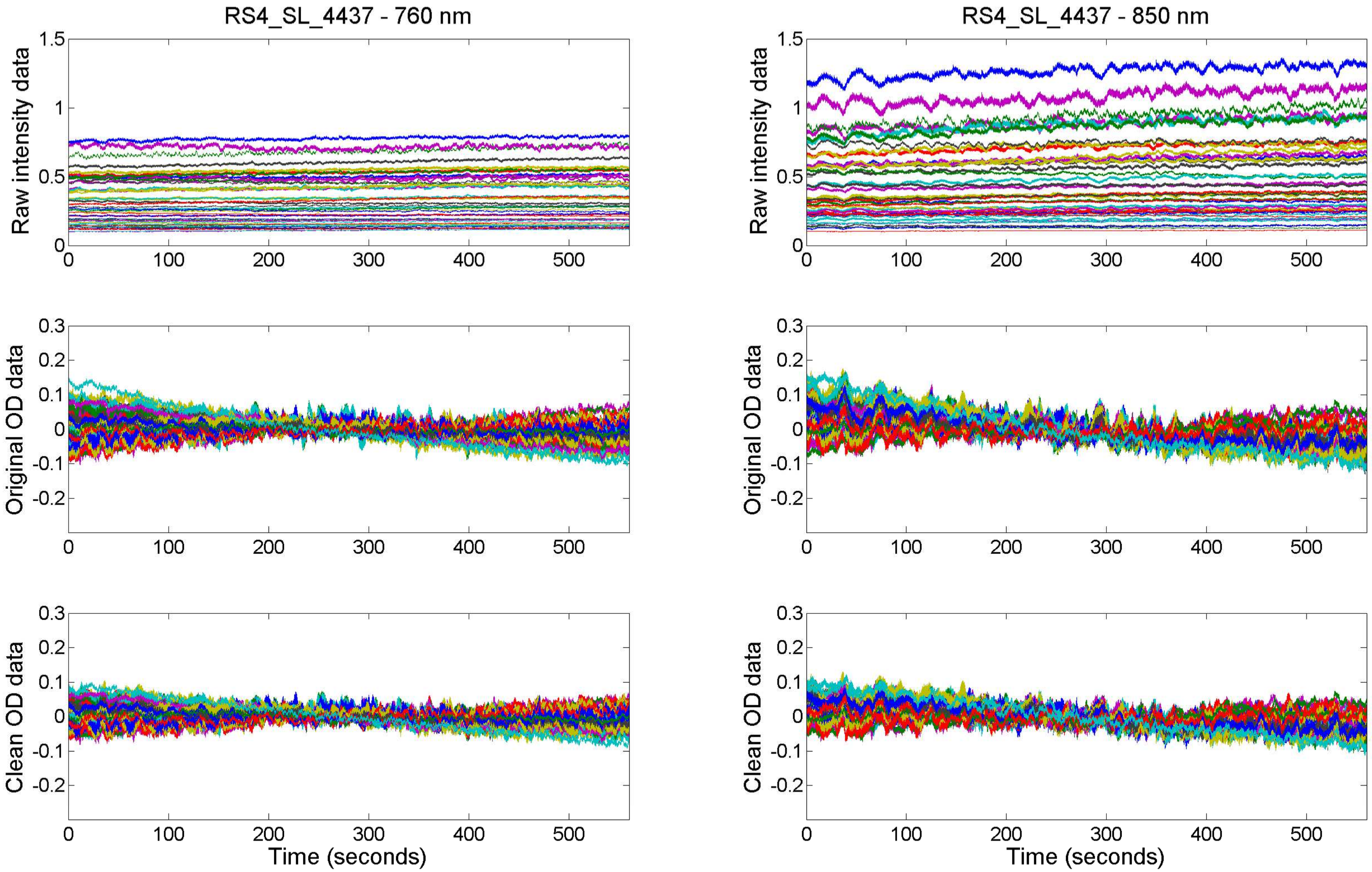

RS4_SL_4437 - 760 nm
RS4_SL_4437 - 850 nm
Raw intensity data
Original OD data
Clean OD data
Time (seconds)

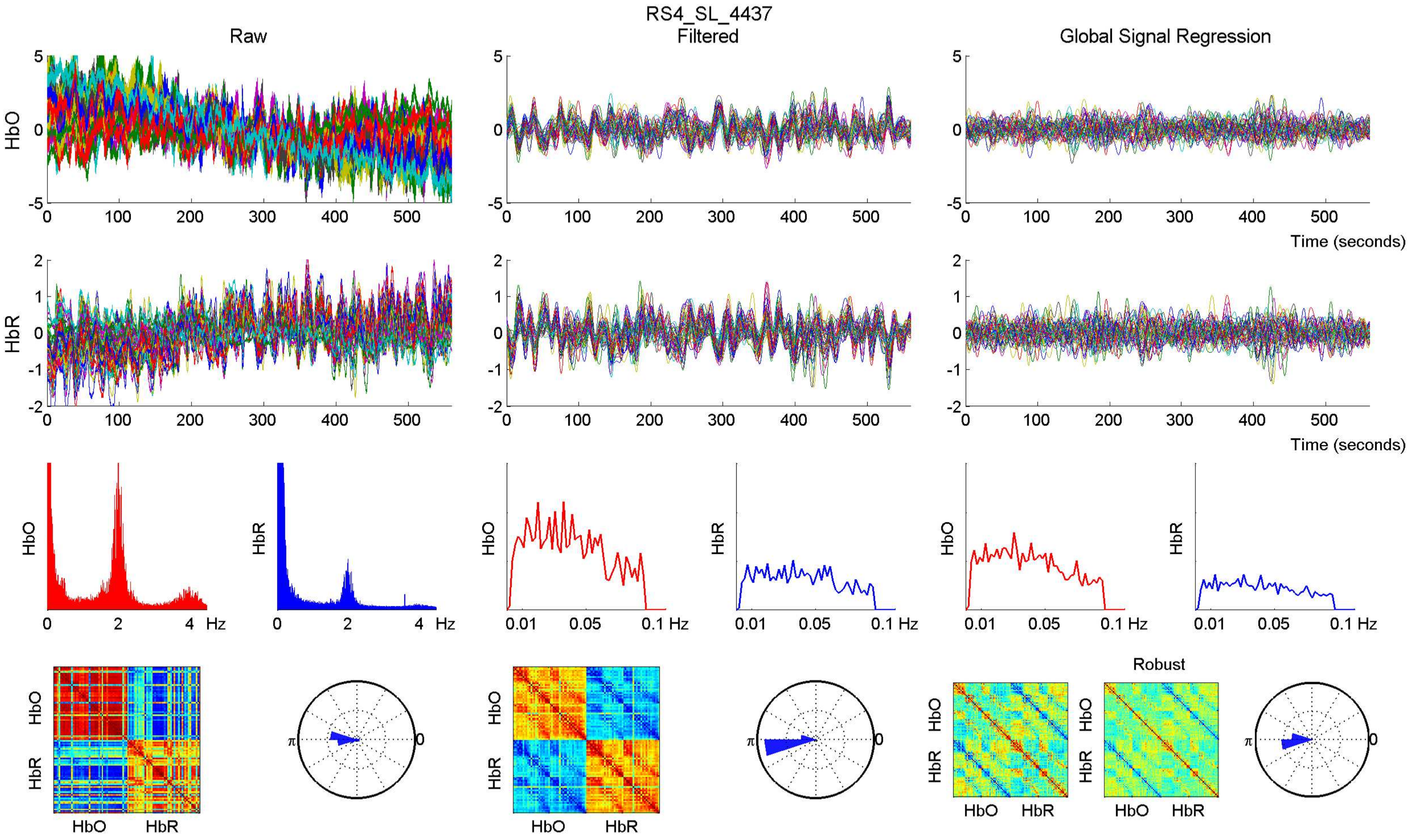

RS4_SL_4437
Raw
Filtered
Global Signal Regression
HbO
HbR
Time (seconds)
Hz
Robust

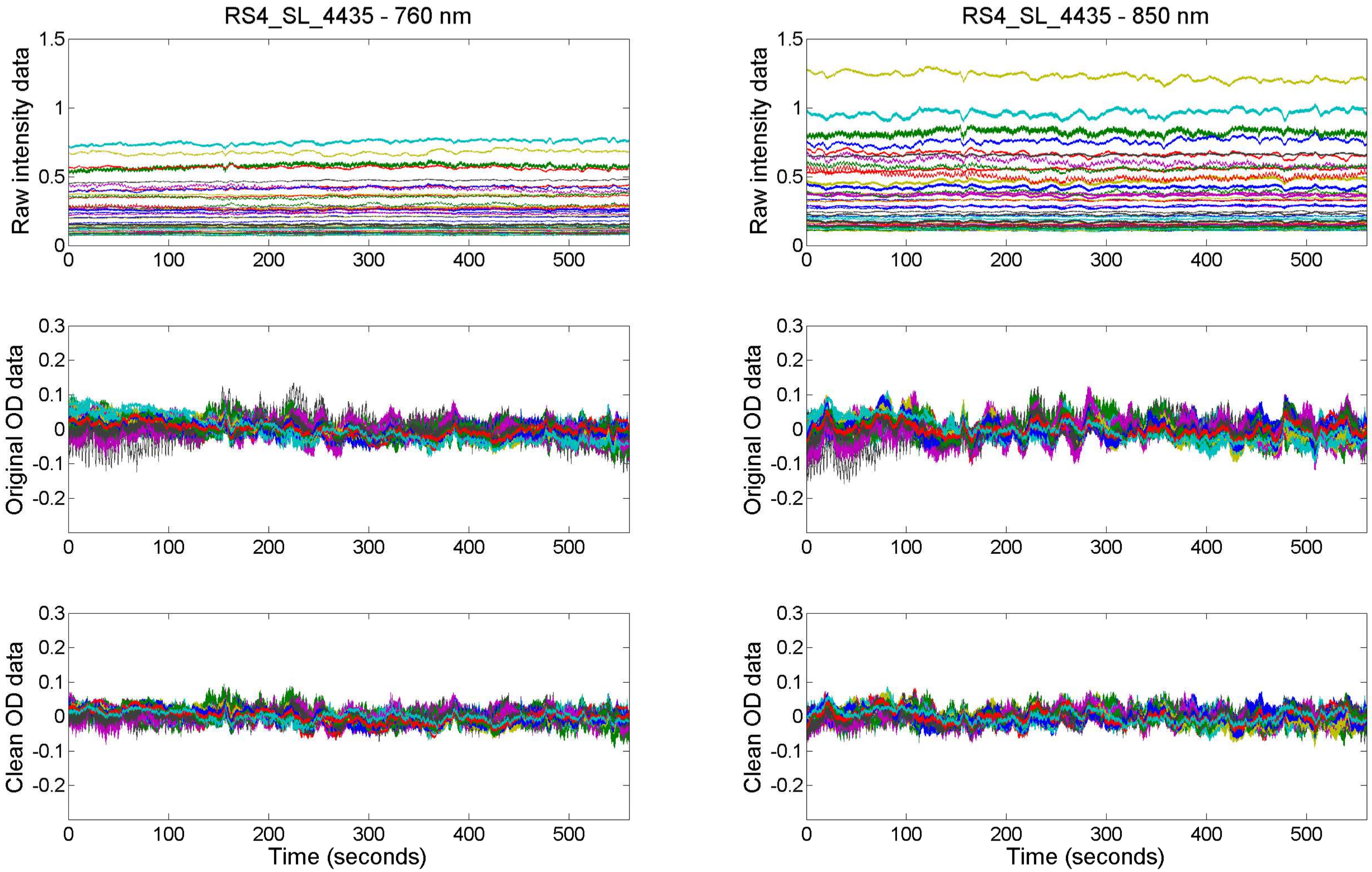
RS4_SL_4435 - 760 nm
RS4_SL_4435 - 850 nm
Raw intensity data
Original OD data
Clean OD data
Time (seconds)
1.5
1
0.5
0
0.3
0.2
0.1
-0.1
-0.2
100
200
300
400
500

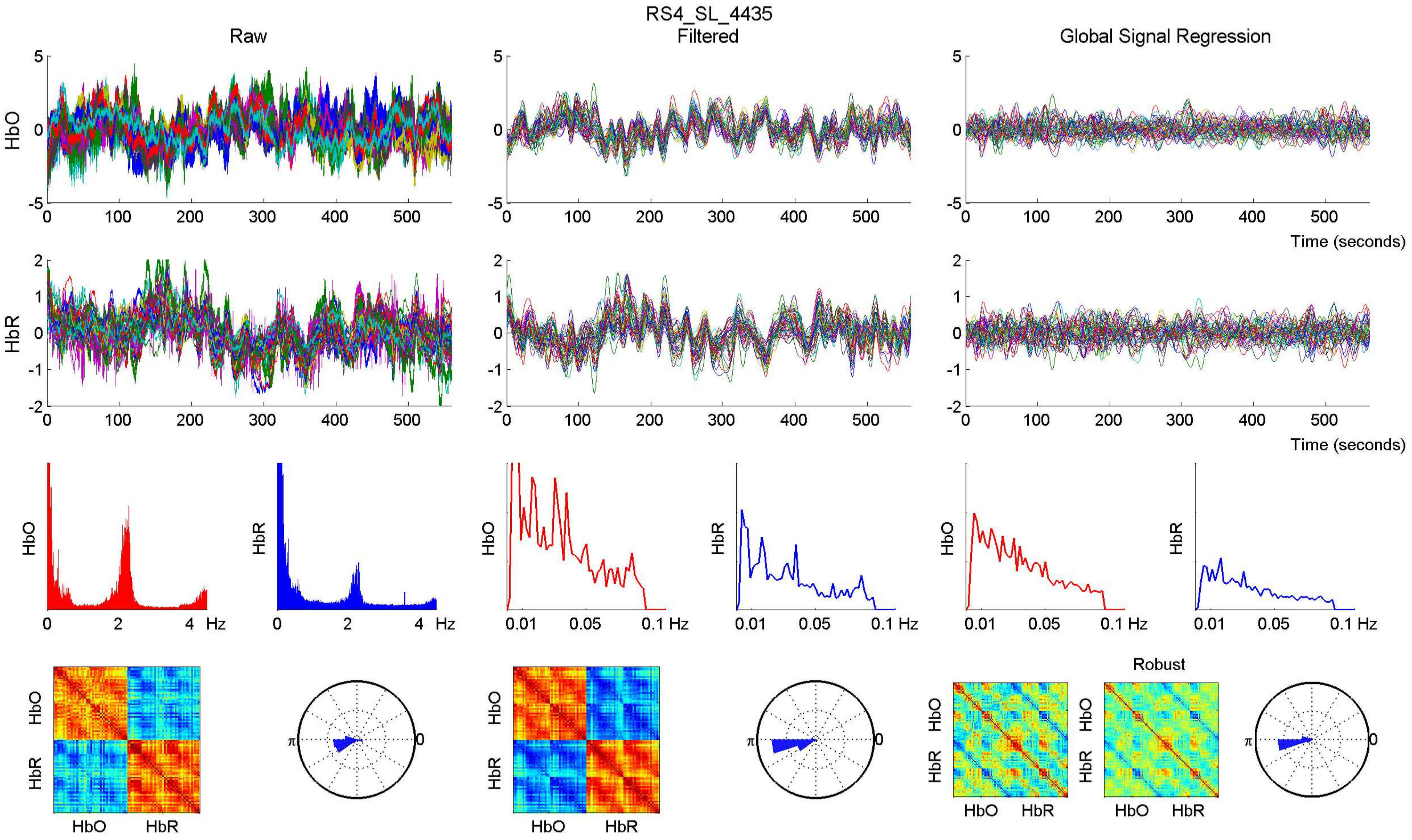

RS4_SL_4435
Raw
Filtered
Global Signal Regression
HbO
HbR
Time (seconds)
Hz
Robust

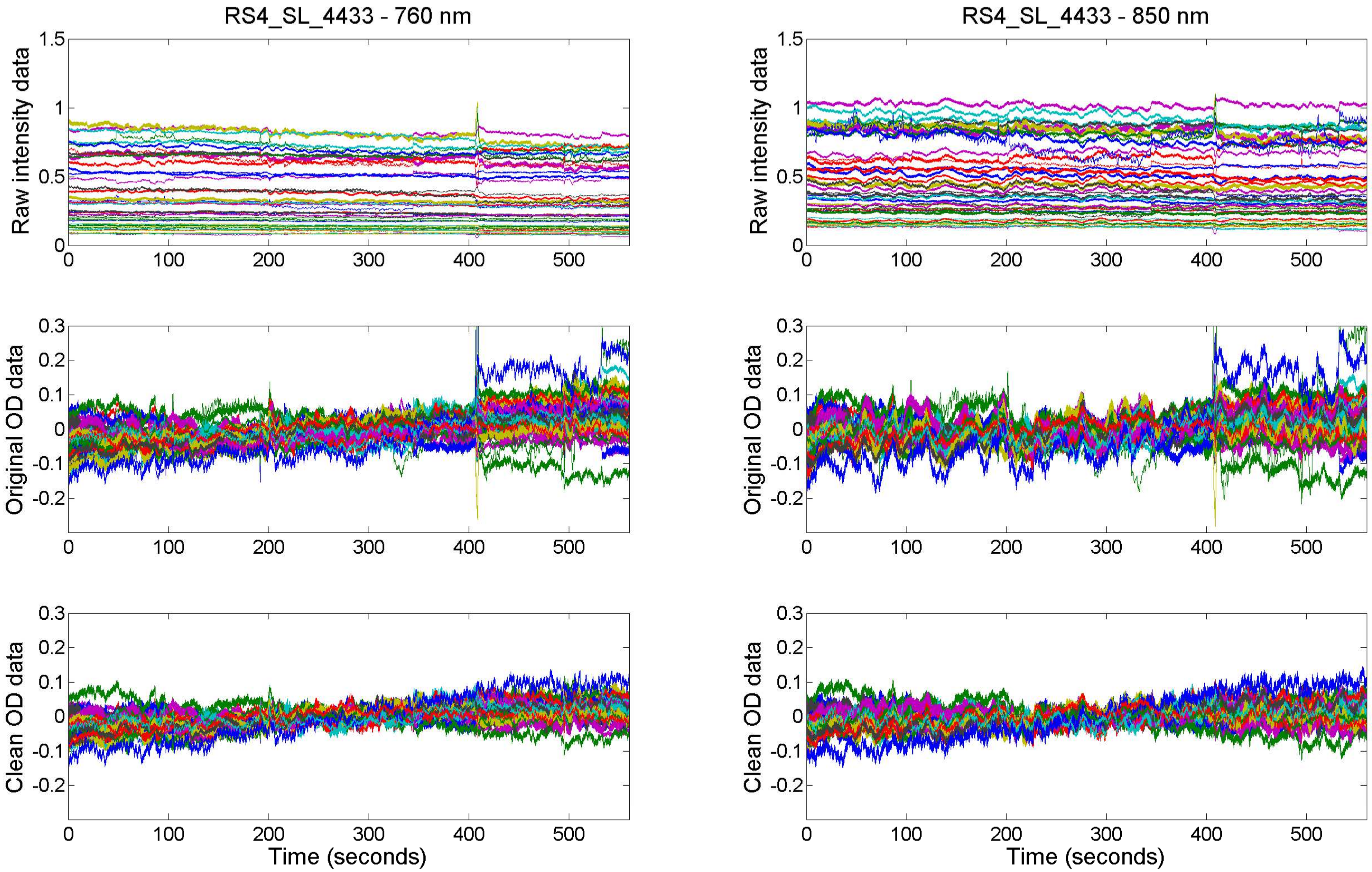
RS4_SL_4433 - 760 nm
RS4_SL_4433 - 850 nm
Raw intensity data
Original OD data
Clean OD data
Time (seconds)

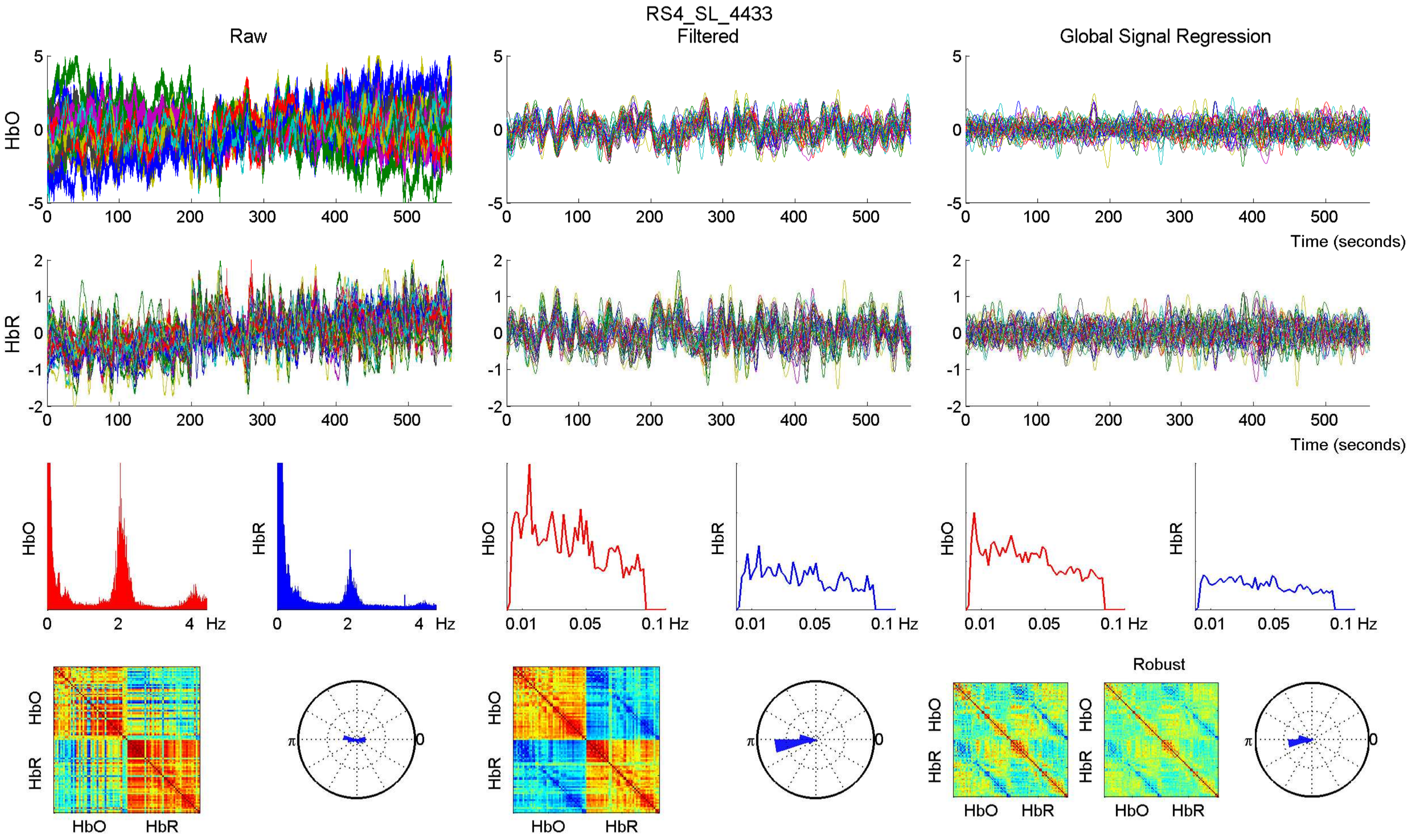

RS4_SL_4433
Raw
Filtered
Global Signal Regression
HbO
HbR
Time (seconds)
Hz
Robust

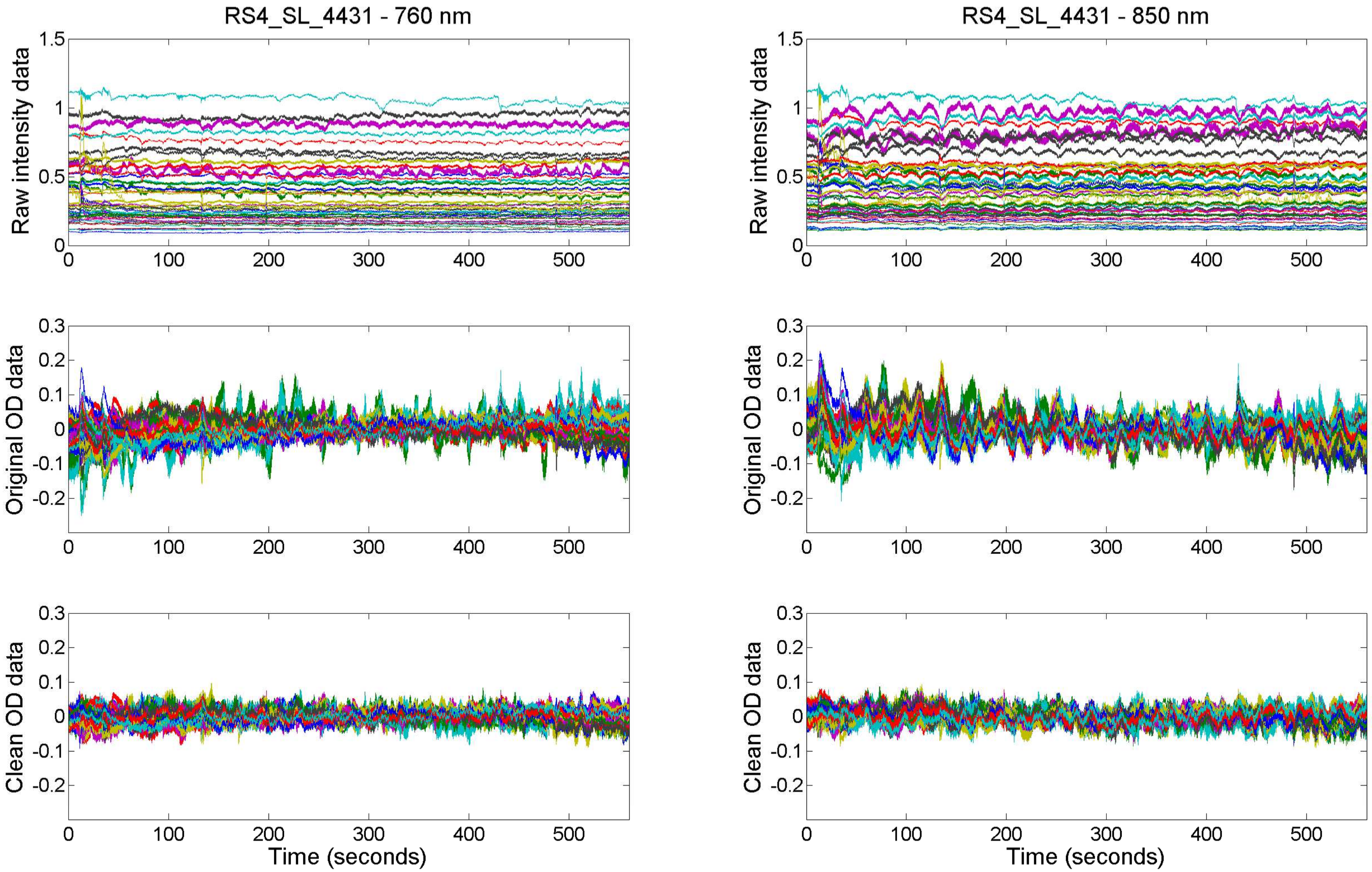
RS4_SL_4431 - 760 nm
RS4_SL_4431 - 850 nm
Raw intensity data
Original OD data
Clean OD data
Time (seconds)
Time (seconds)

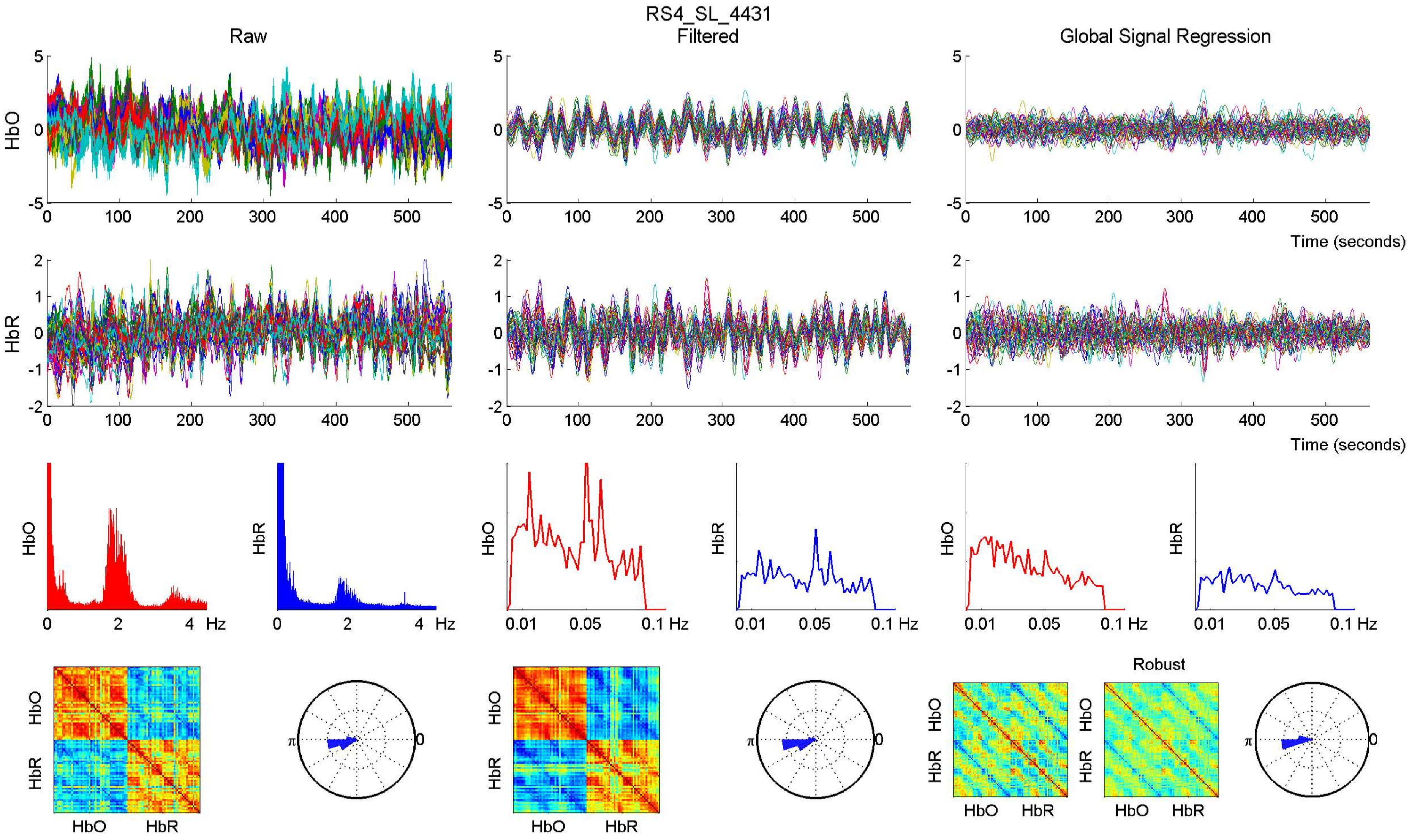
RS4_SL_4431
Raw
Filtered
Global Signal Regression
HbO
HbR
Time (seconds)
Time (seconds)
0 2 4 Hz
0.01 0.05 0.1 Hz
Robust
HbO HbR
π
0

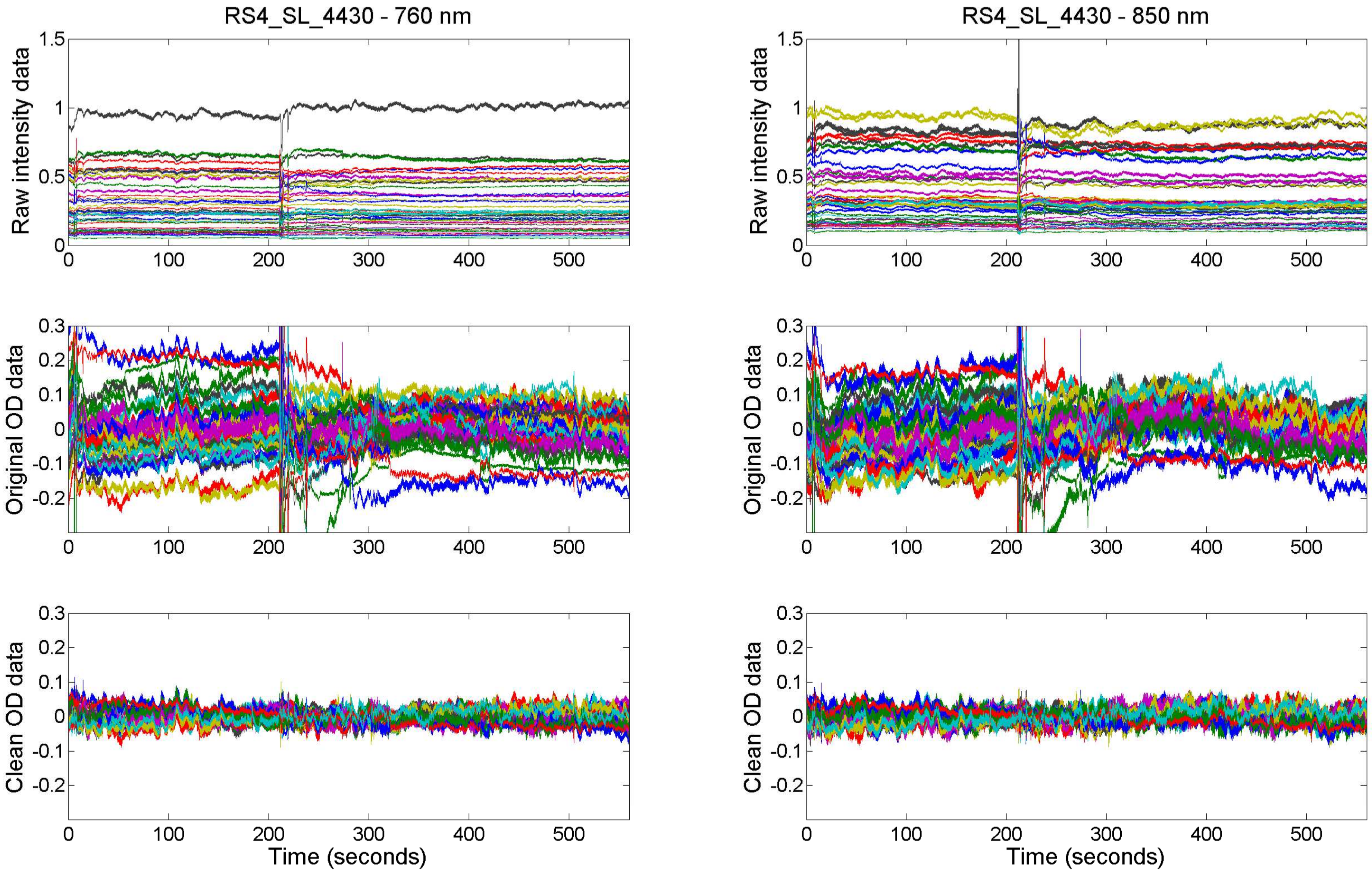
RS4_SL_4430 - 760 nm
RS4_SL_4430 - 850 nm
Raw intensity data
Original OD data
Clean OD data
Time (seconds)
0
100
200
300
400
500
1.5
1
0.5
0.3
0.2
0.1
-0.1
-0.2

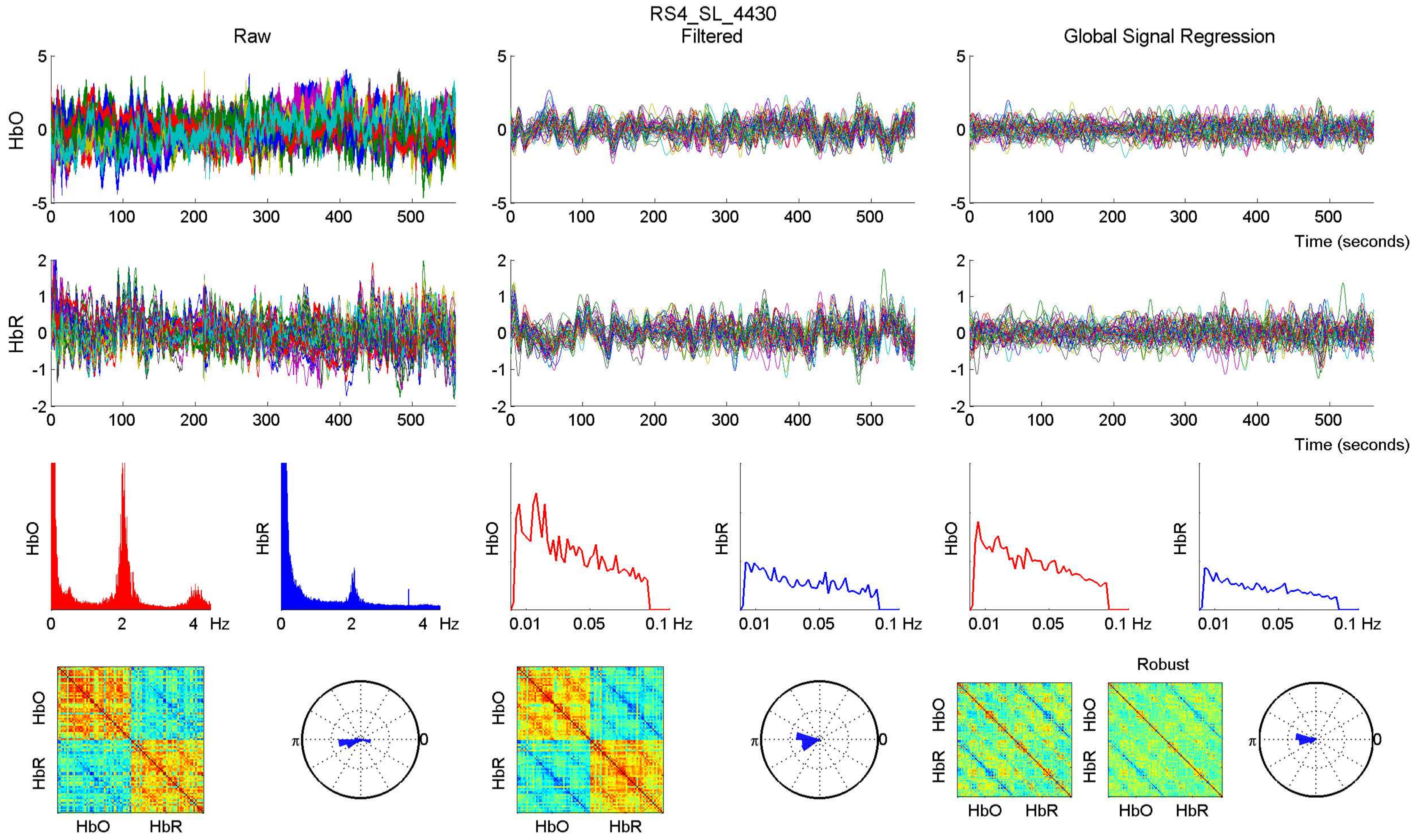
RS4_SL_4430
Raw
Filtered
Global Signal Regression
HbO
HbR
Time (seconds)
Hz
Robust

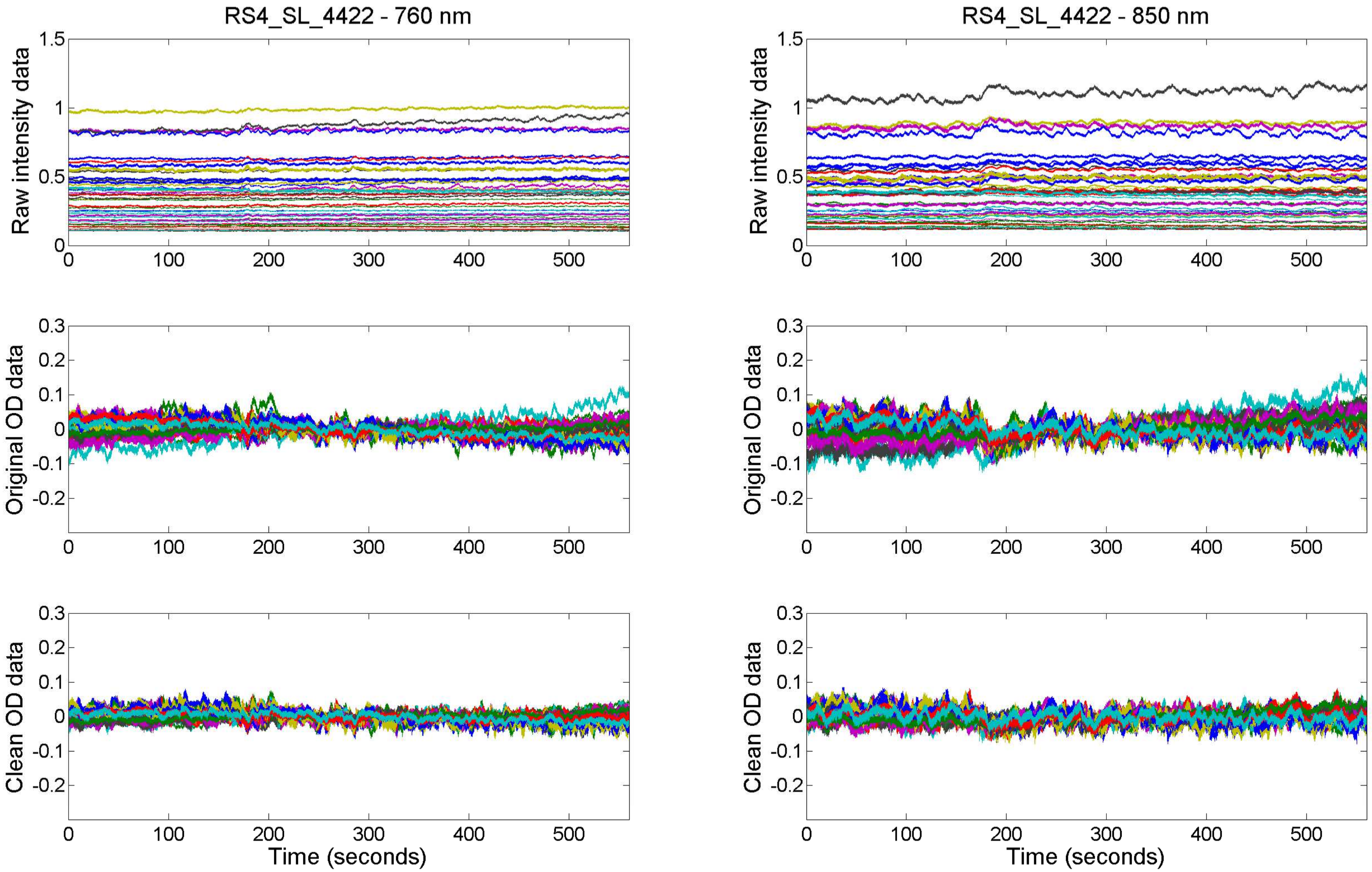

RS4_SL_4422 - 760 nm
RS4_SL_4422 - 850 nm
Raw intensity data
Original OD data
Clean OD data
Time (seconds)
0
100
200
300
400
500
1.5
1
0.5
0.3
0.2
0.1
-0.1
-0.2

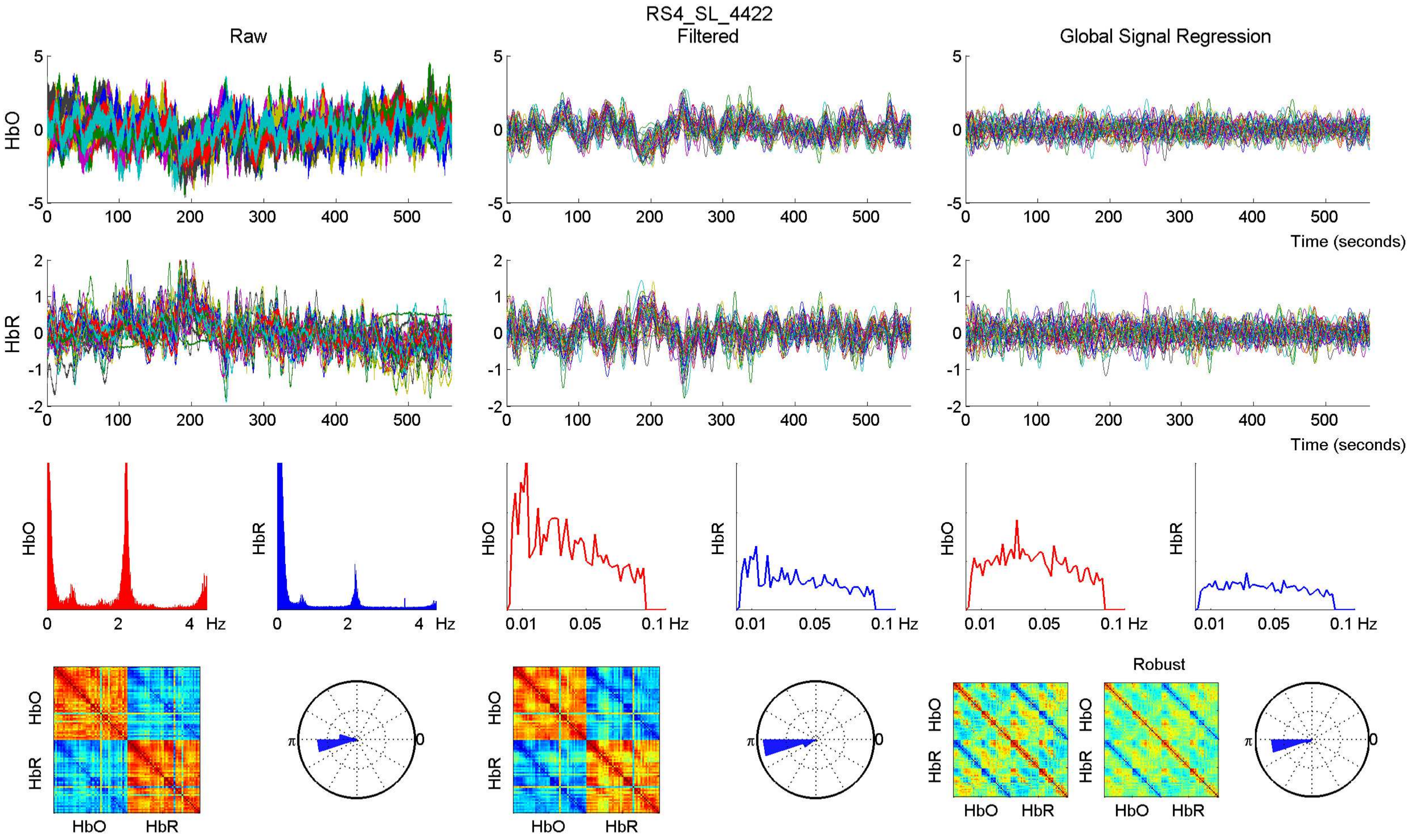

RS4_SL_4422
Raw
Filtered
Global Signal Regression
HbO
HbR
Time (seconds)
Time (seconds)
Hz
0.1 Hz
Robust
HbO
HbR

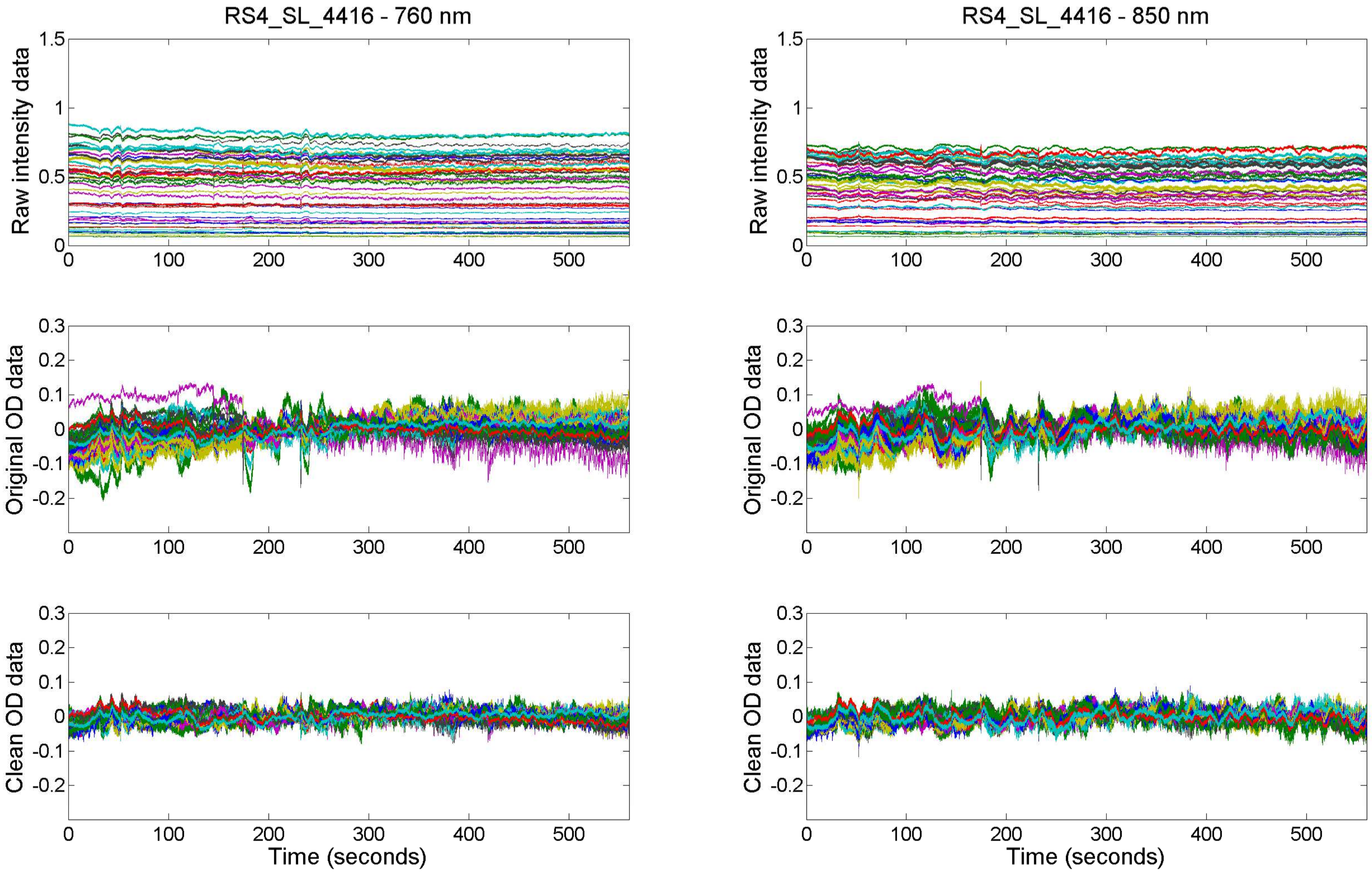

RS4_SL_4416 - 760 nm
RS4_SL_4416 - 850 nm
Raw intensity data
Original OD data
Clean OD data
Time (seconds)

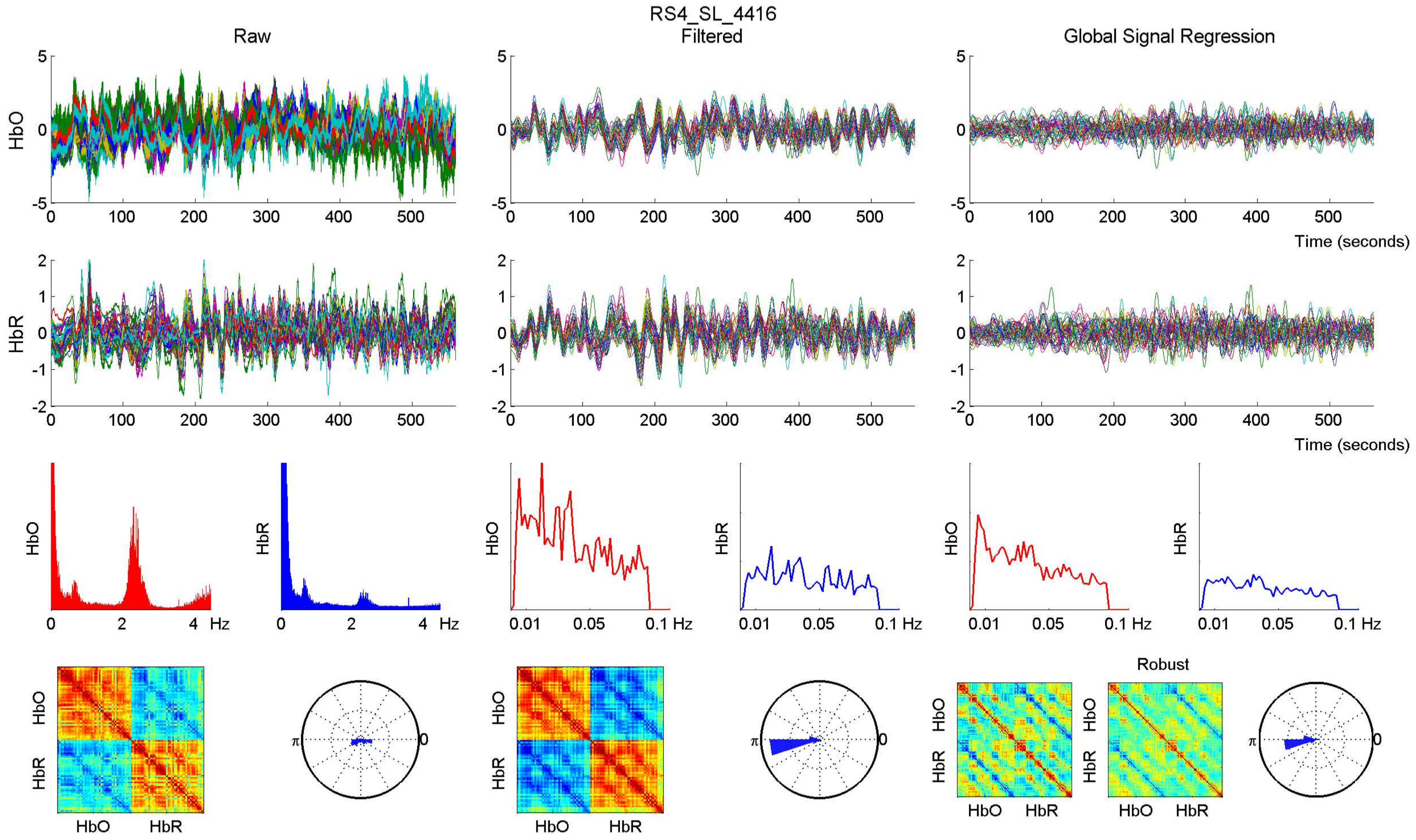

RS4_SL_4416
Raw
Filtered
Global Signal Regression
HbO
HbR
Time (seconds)
Hz
Robust

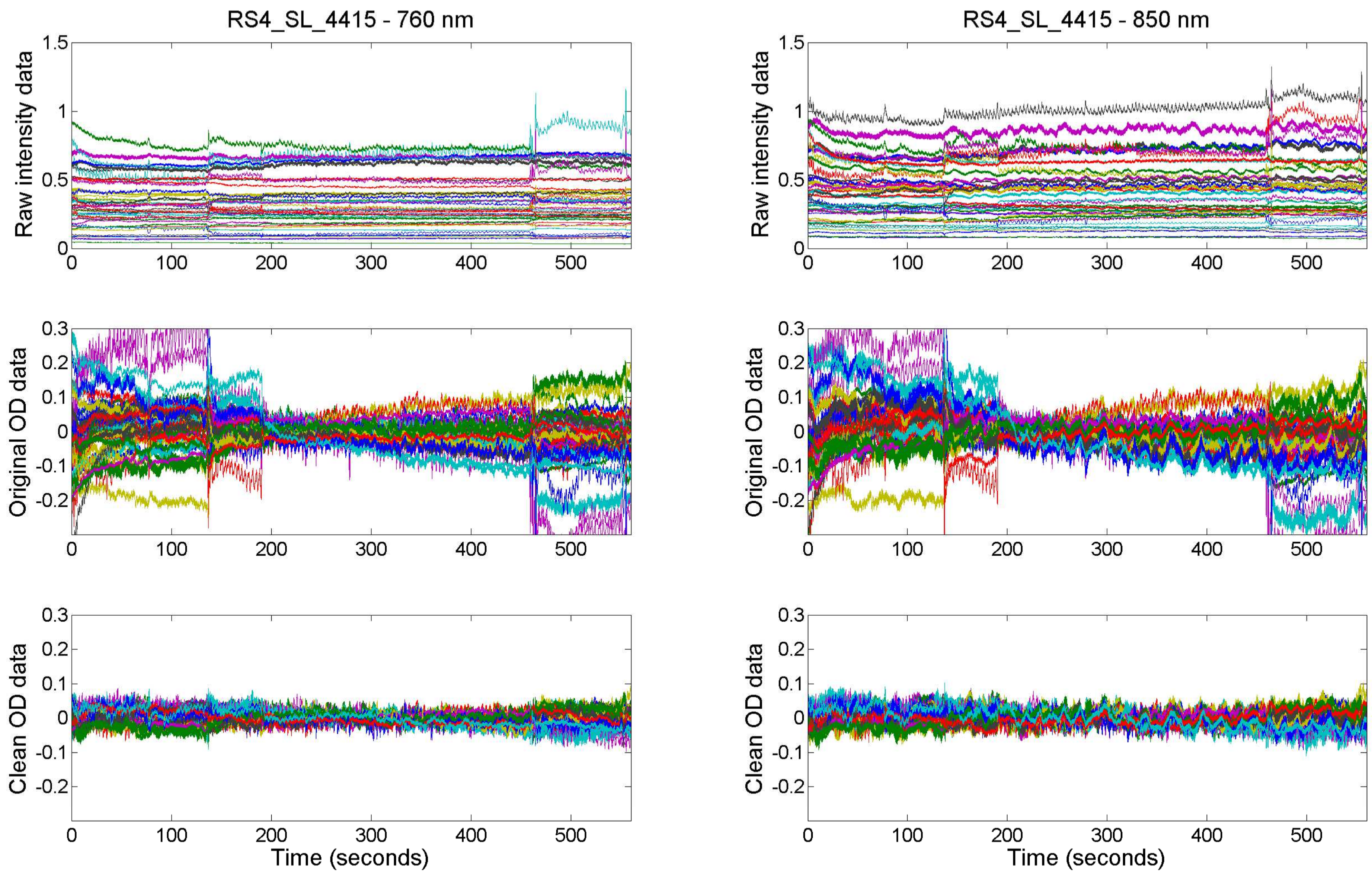

RS4_SL_4415 - 760 nm
RS4_SL_4415 - 850 nm
Raw intensity data
Original OD data
Clean OD data
Time (seconds)

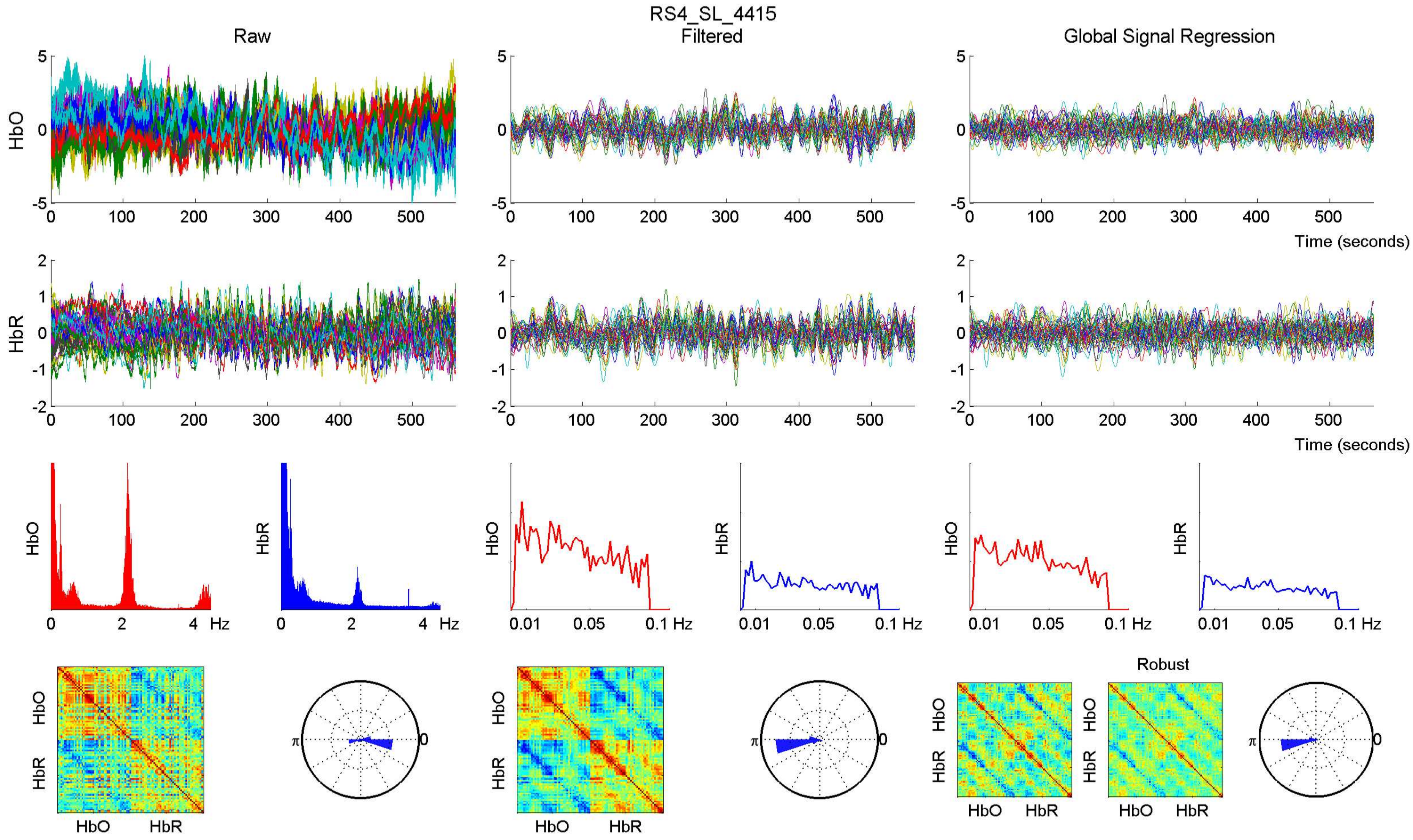
RS4_SL_4415
Raw
Filtered
Global Signal Regression
HbO
HbR
Time (seconds)
Hz
0.01
0.05
0.1 Hz
Robust
HbO
HbR

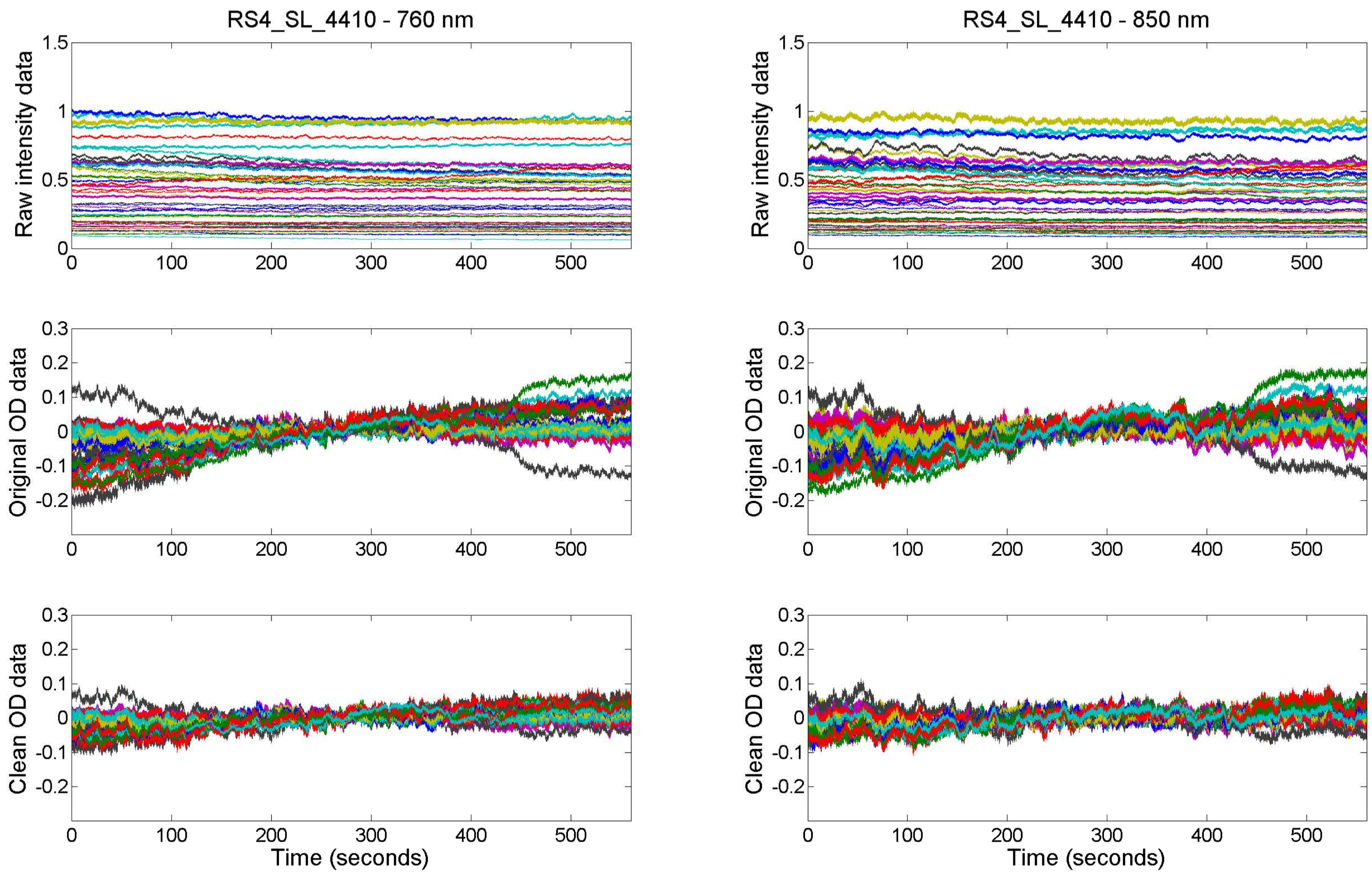
RS4_SL_4410 - 760 nm
RS4_SL_4410 - 850 nm
Raw intensity data
Original OD data
Clean OD data
Time (seconds)
0
100
200
300
400
500
1.5
1
0.5
0.3
0.2
0.1
-0.1
-0.2

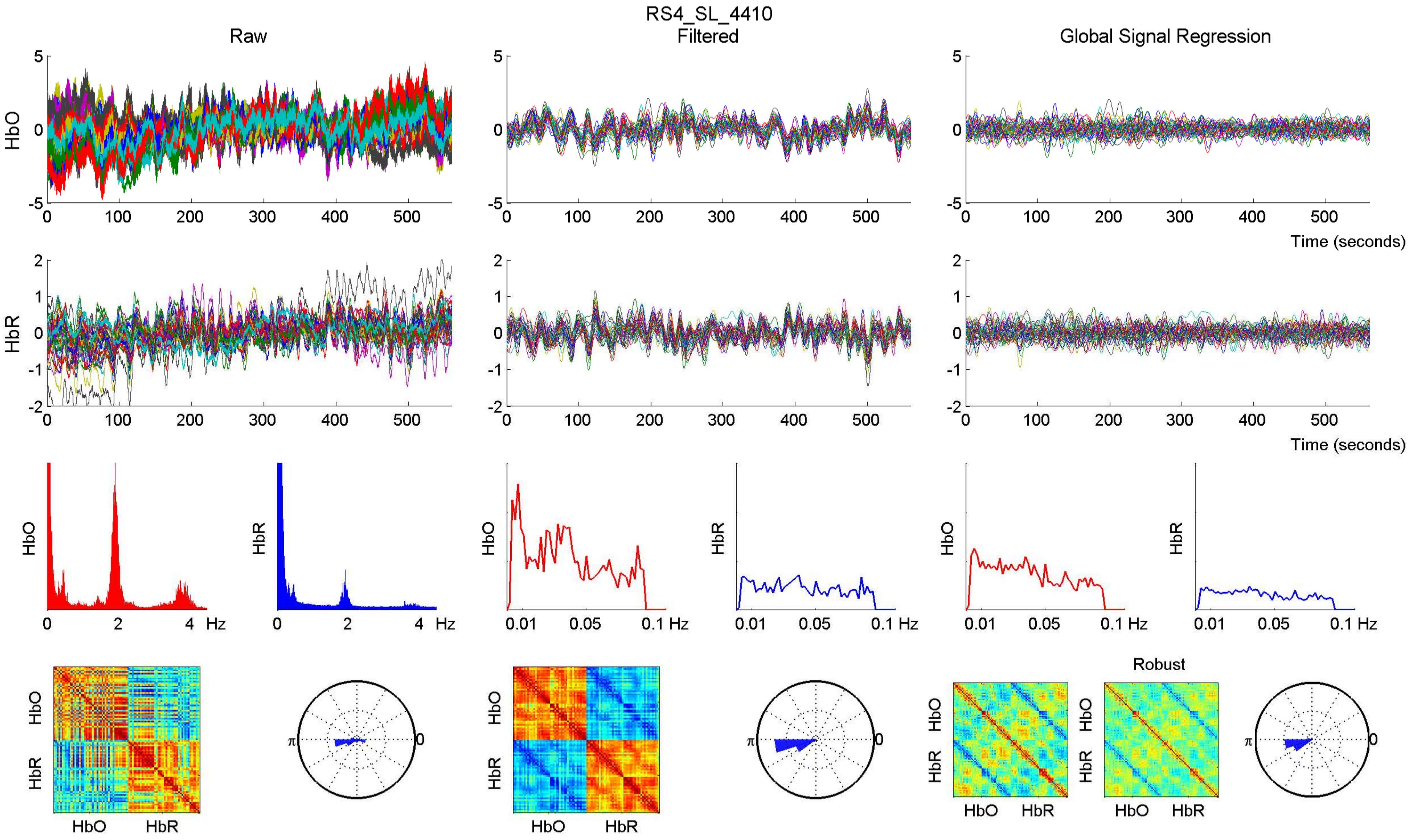

RS4_SL_4410
Raw
Filtered
Global Signal Regression
HbO
HbR
Time (seconds)
Hz
0.01
0.05
0.1 Hz
Robust
π
0

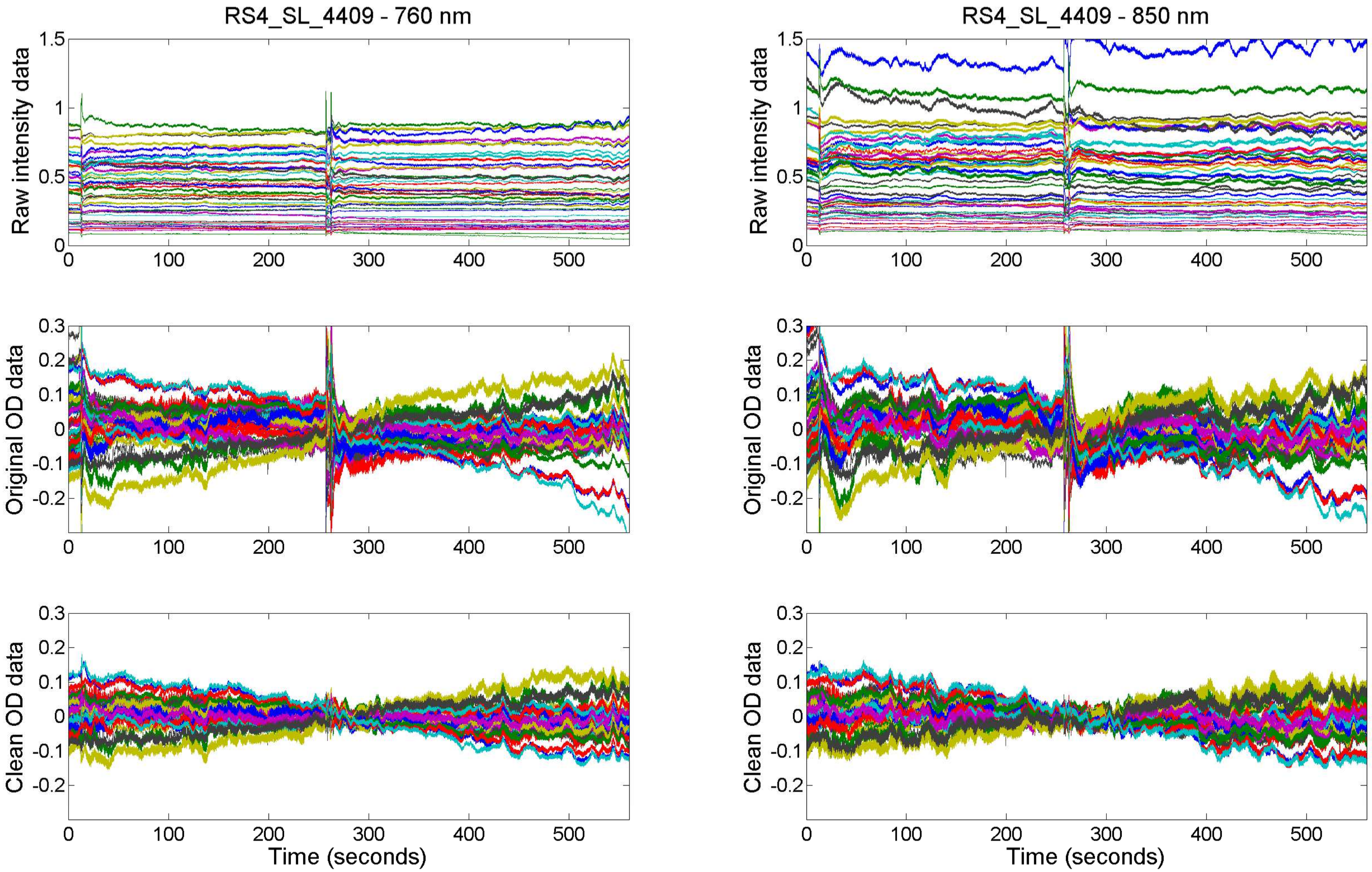
RS4_SL_4409 - 760 nm
RS4_SL_4409 - 850 nm
Raw intensity data
Original OD data
Clean OD data
Time (seconds)

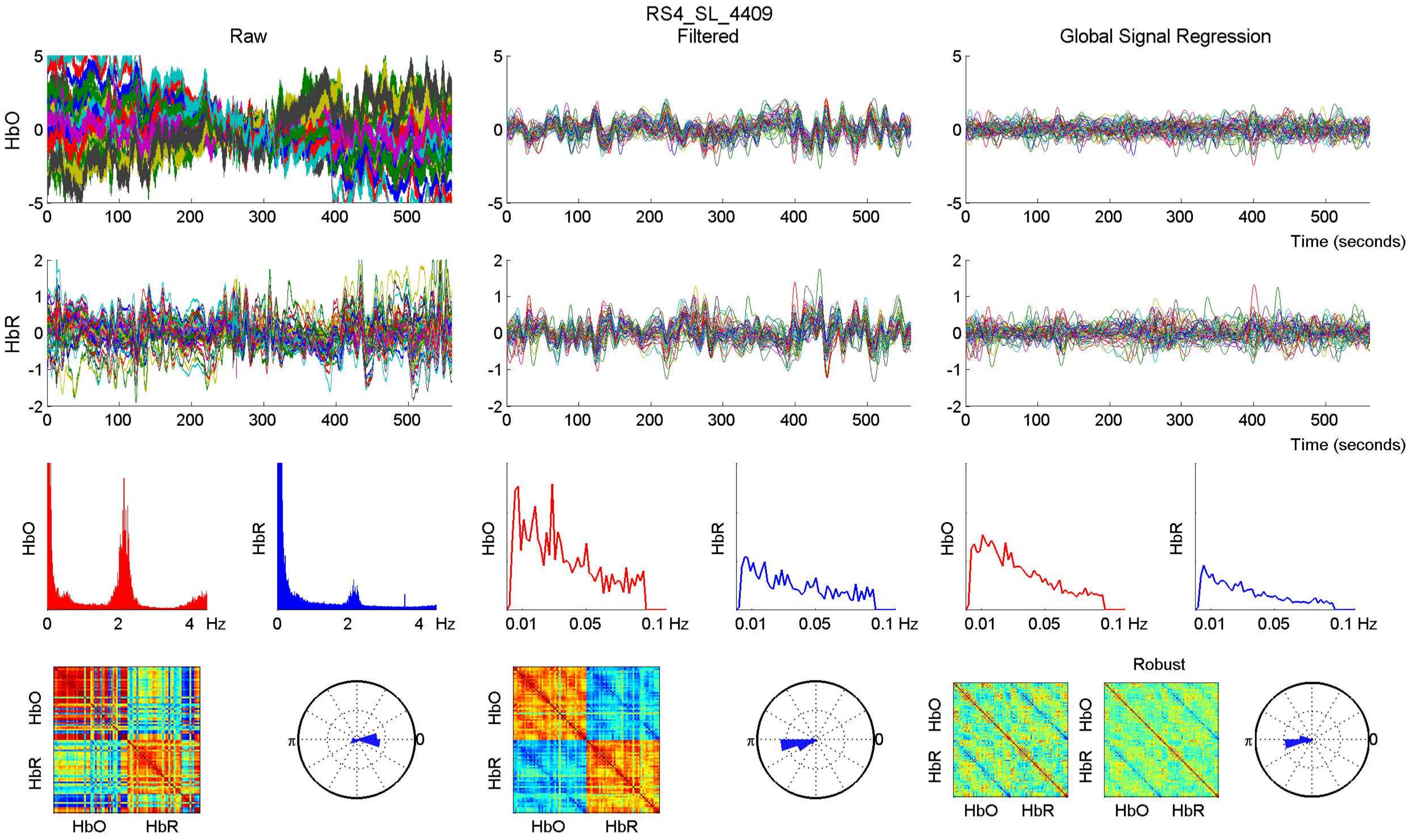

RS4_SL_4409
Raw
Filtered
Global Signal Regression
HbO
HbR
Time (seconds)
Hz
Robust
HbO
HbR

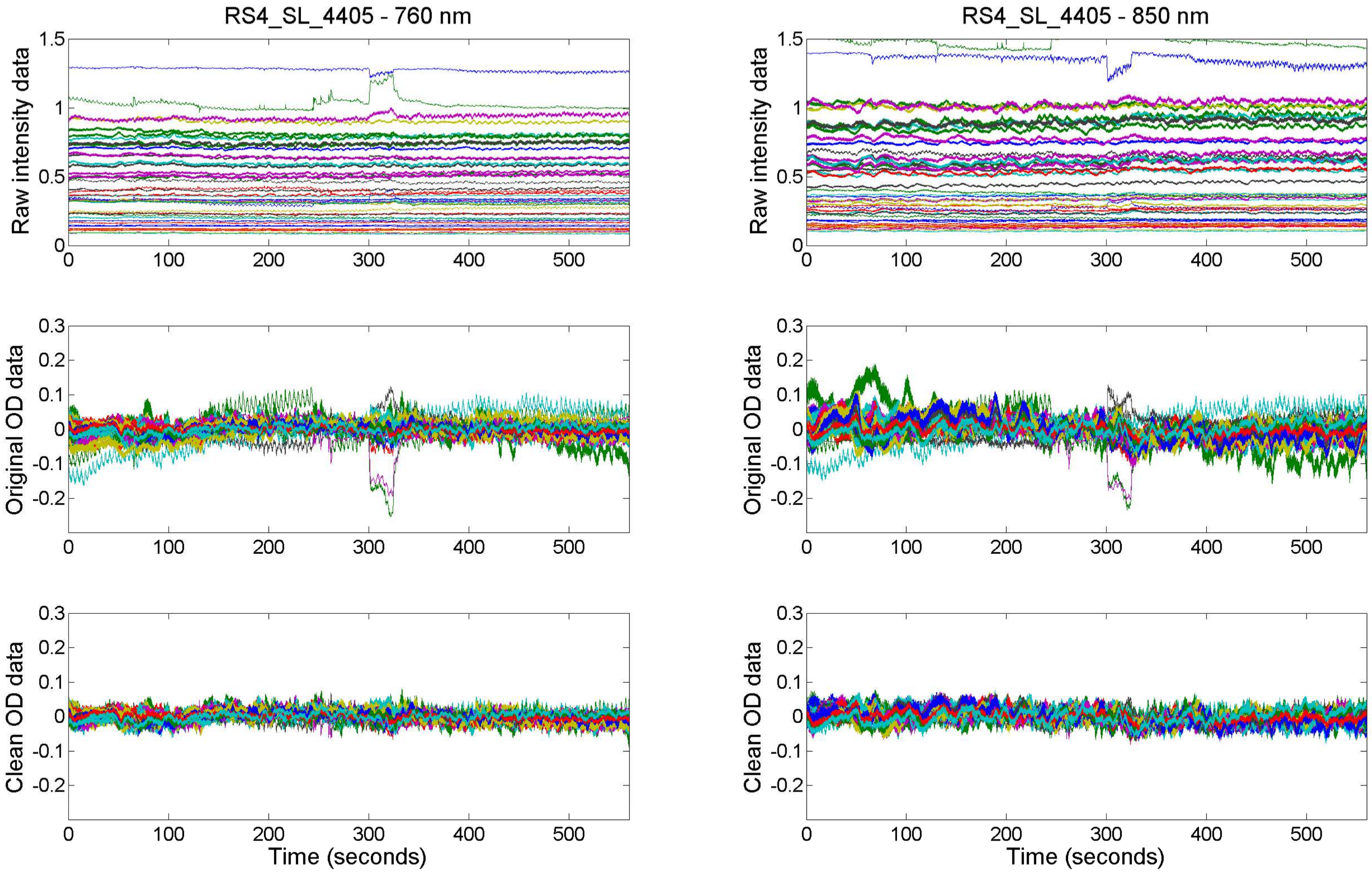

RS4_SL_4405 - 760 nm
RS4_SL_4405 - 850 nm
Raw intensity data
Original OD data
Clean OD data
Time (seconds)

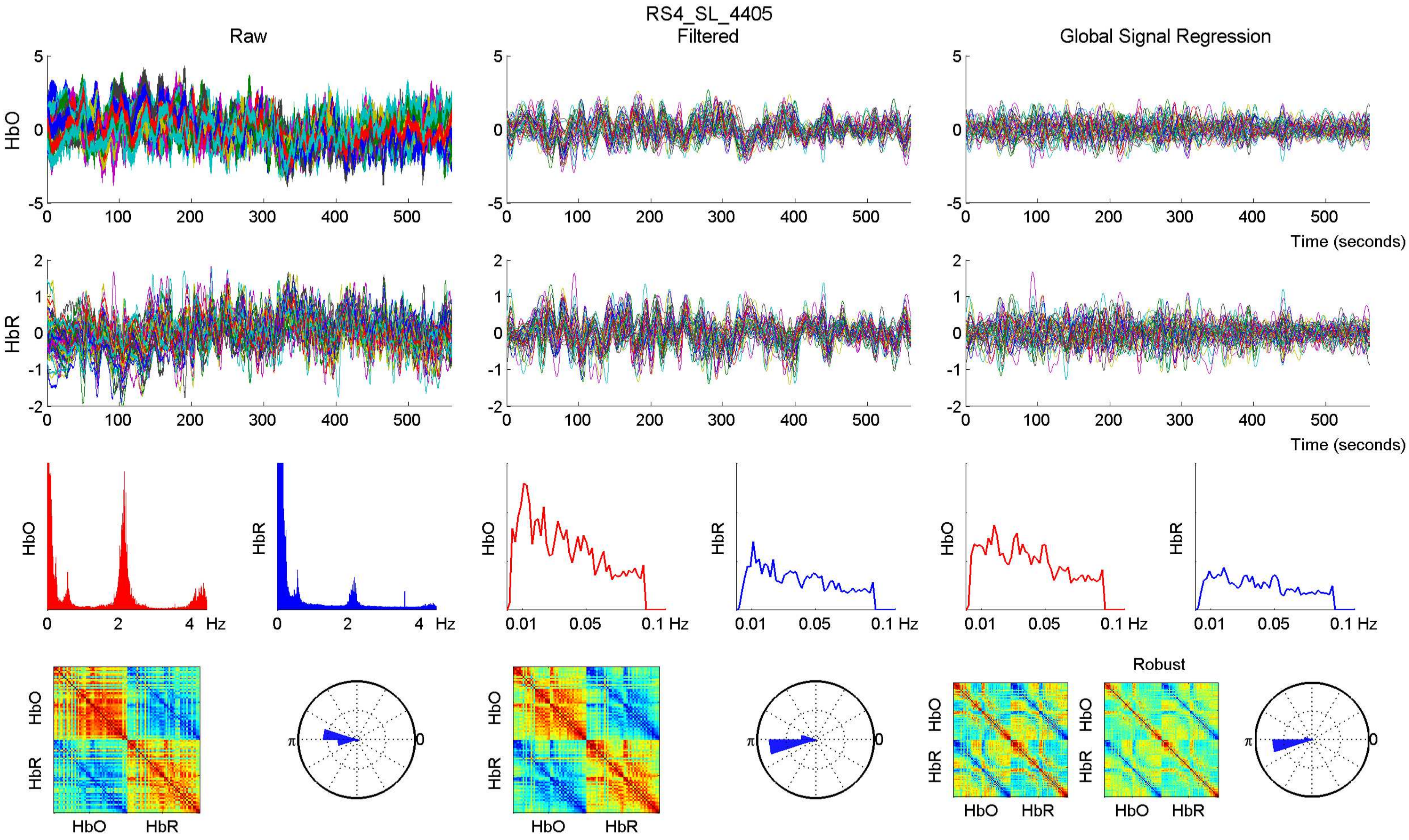

RS4_SL_4405
Raw
Filtered
Global Signal Regression
HbO
HbR
Time (seconds)
Hz
0.01
0.05
0.1 Hz
Robust
π
0

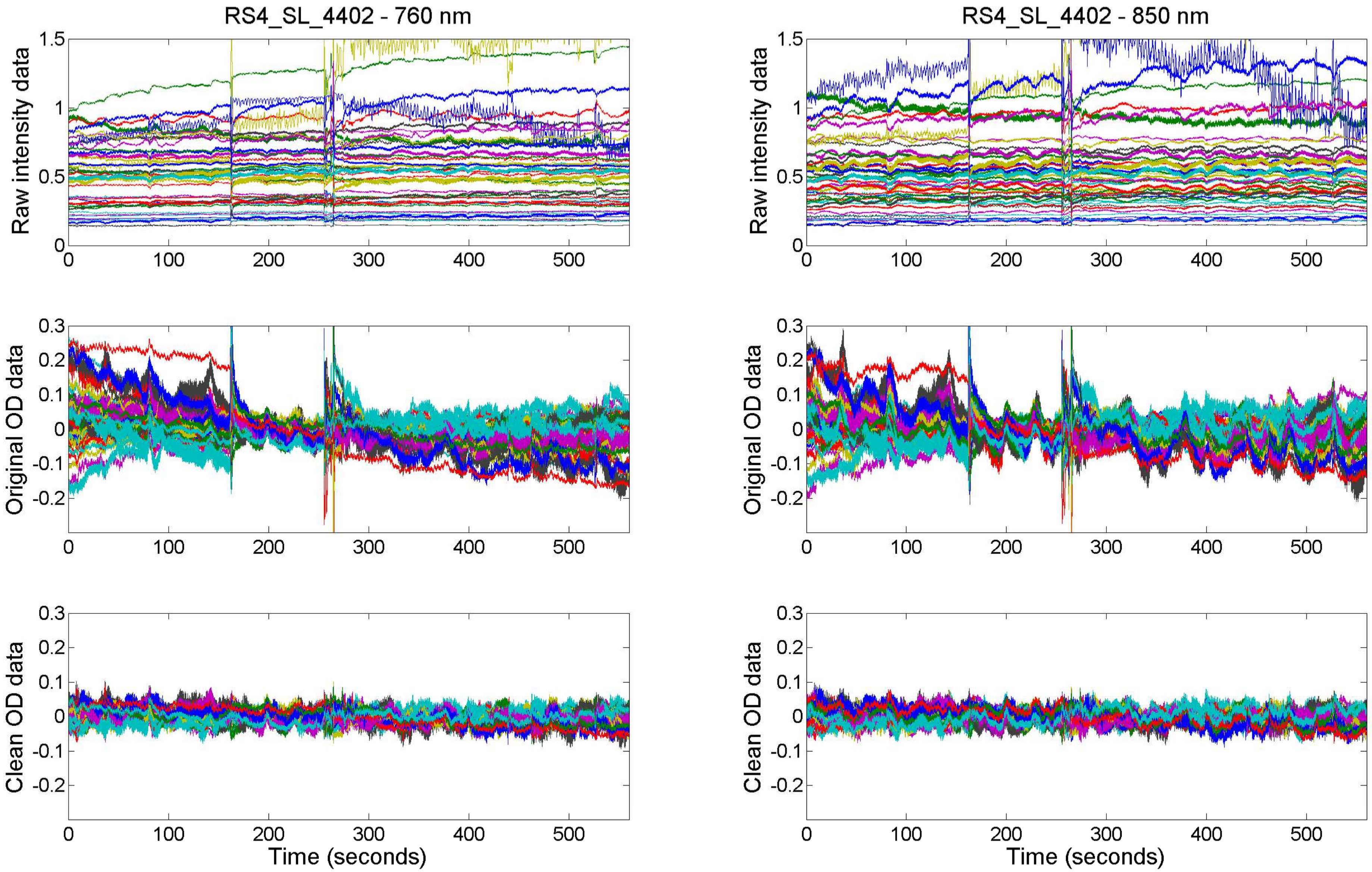
RS4_SL_4402 - 760 nm
RS4_SL_4402 - 850 nm
Raw intensity data
Original OD data
Clean OD data
Time (seconds)
0
0.5
1
1.5
-0.2
-0.1
0.1
0.2
0.3
100
200
300
400
500

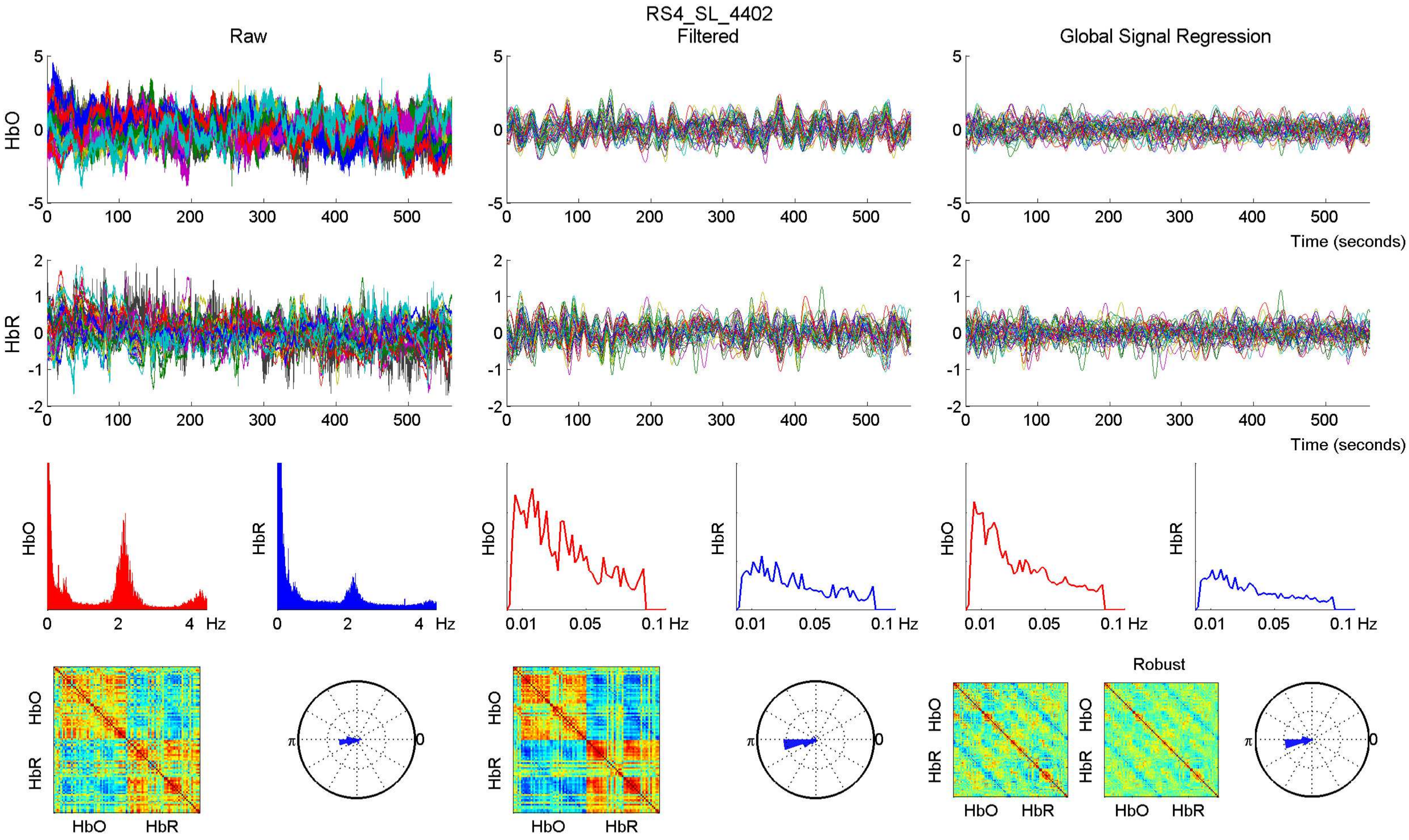

RS4_SL_4402
Raw
Filtered
Global Signal Regression
HbO
HbR
Time (seconds)
Hz
0.01
0.05
0.1 Hz
Robust
π
0

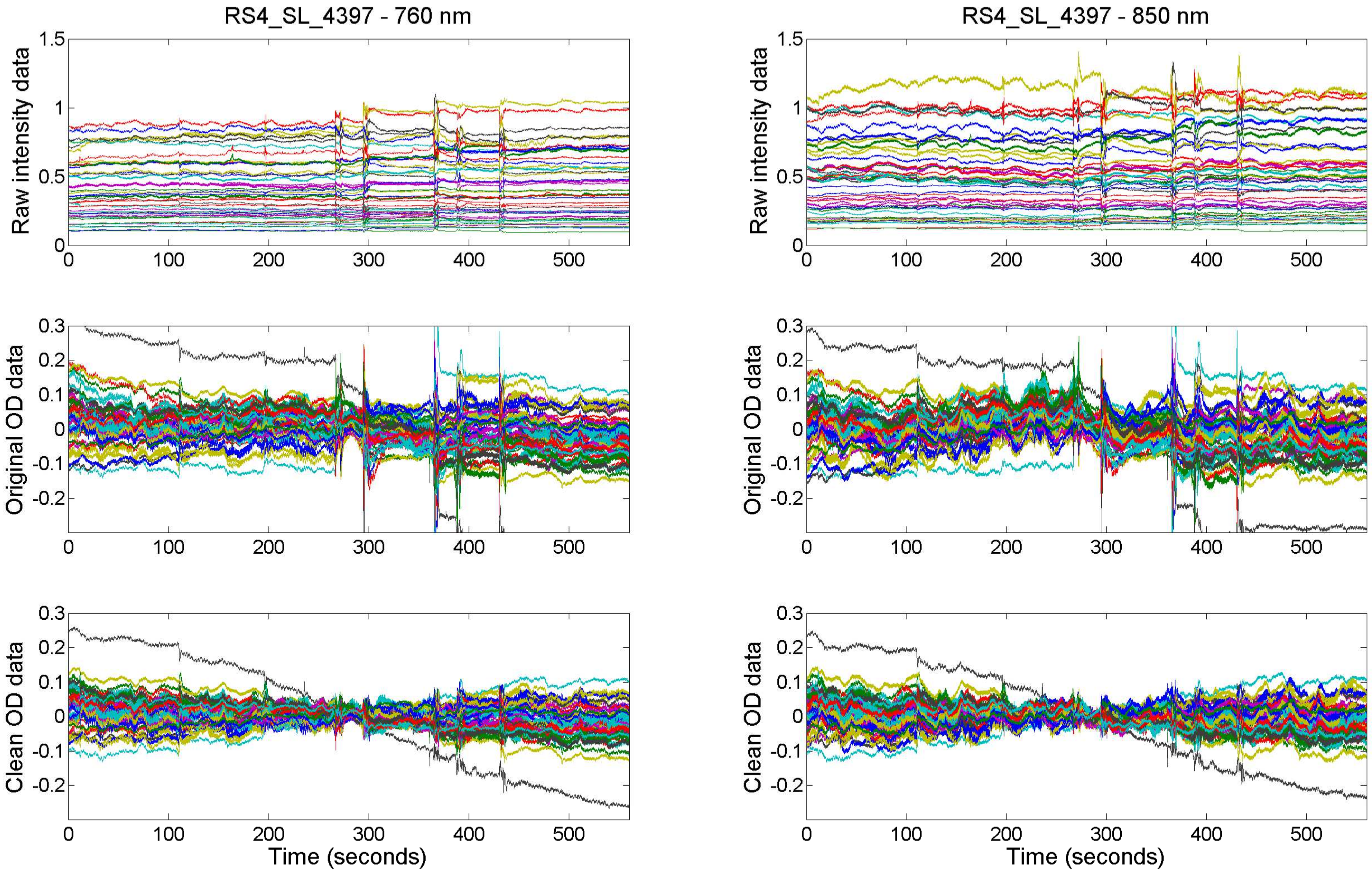

RS4_SL_4397 - 760 nm
RS4_SL_4397 - 850 nm
Raw intensity data
Original OD data
Clean OD data
Time (seconds)

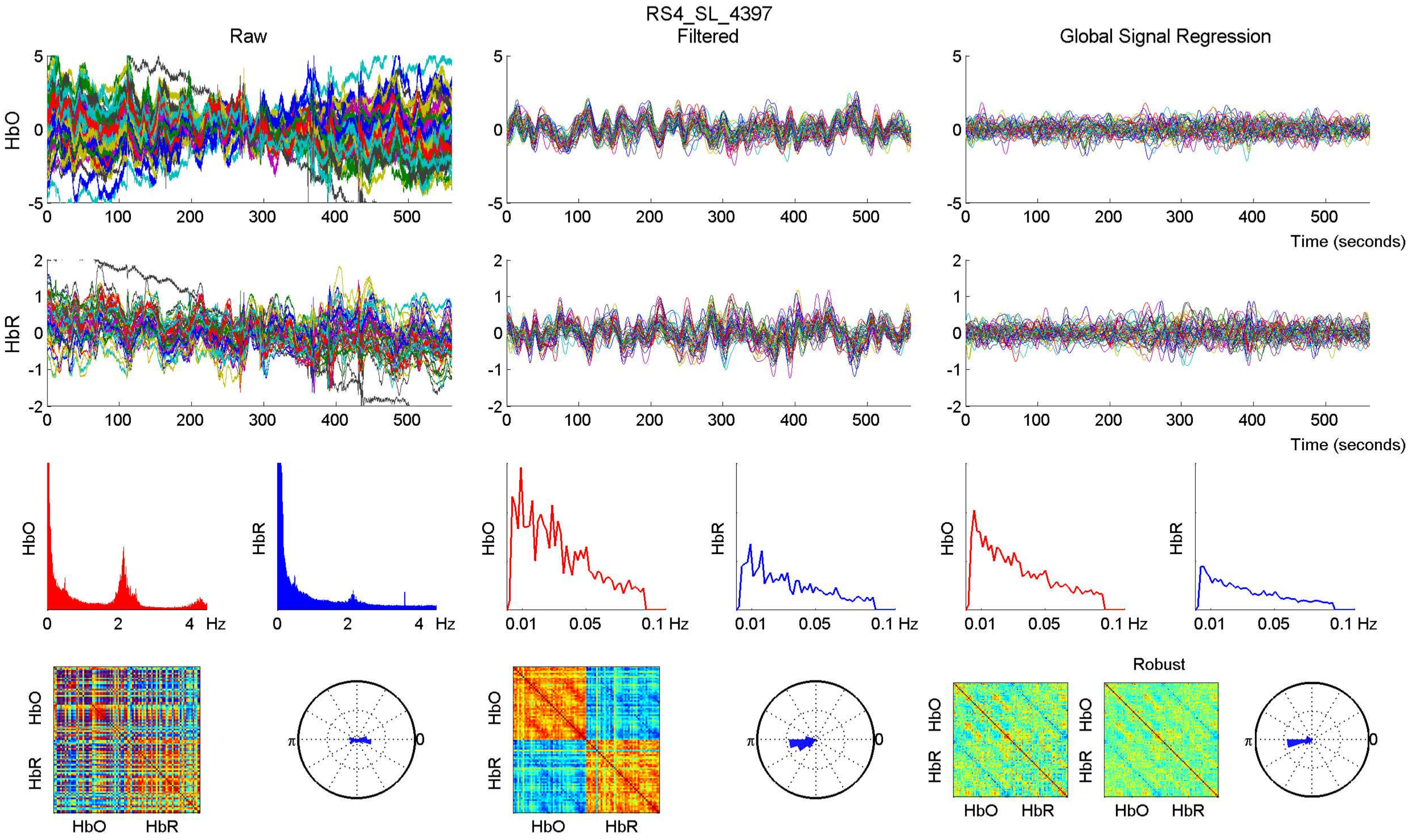

RS4_SL_4397
Raw
Filtered
Global Signal Regression
HbO
HbR
Time (seconds)
Hz
Robust

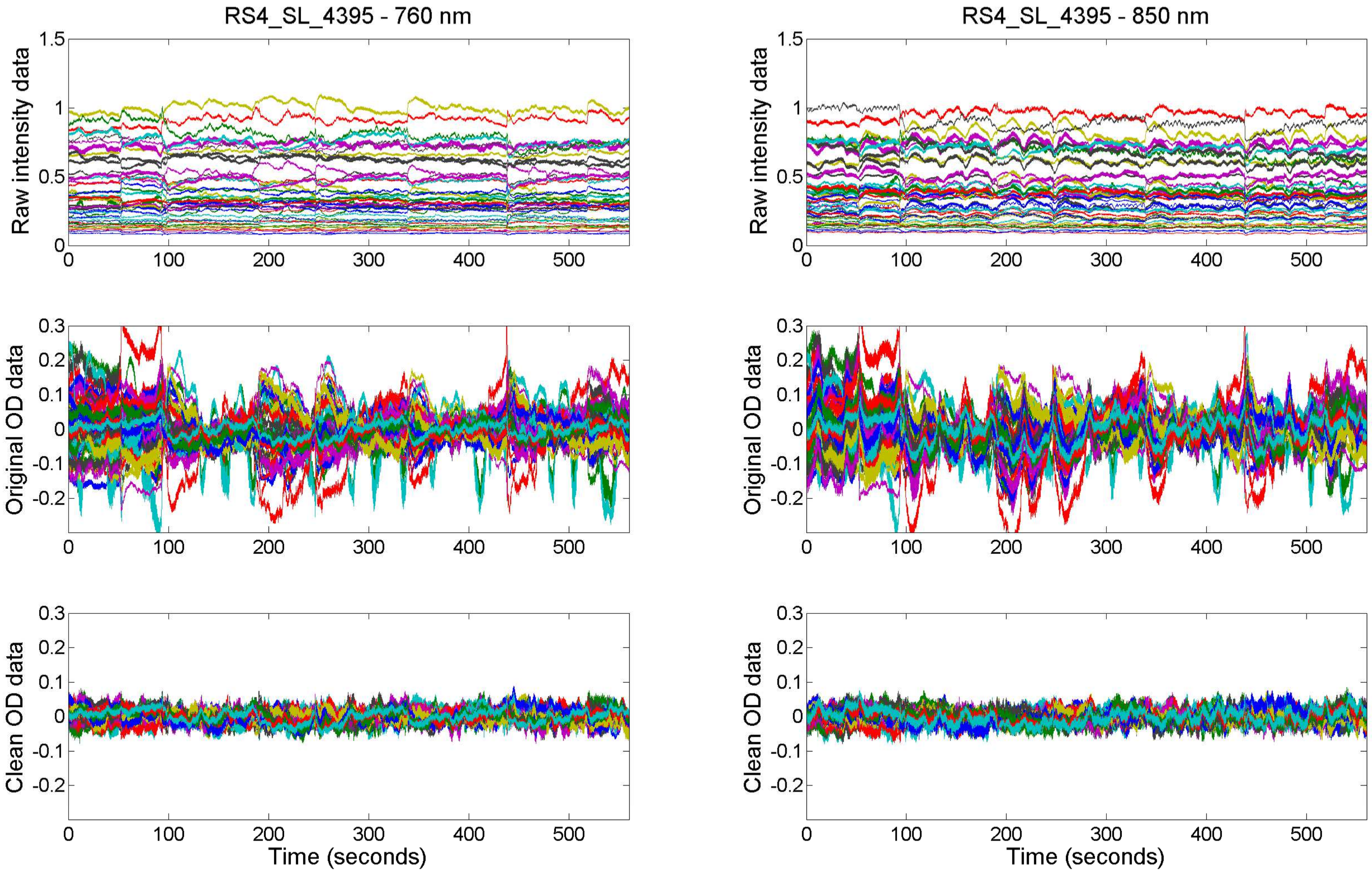

RS4_SL_4395 - 760 nm
RS4_SL_4395 - 850 nm
Raw intensity data
Original OD data
Clean OD data
Time (seconds)

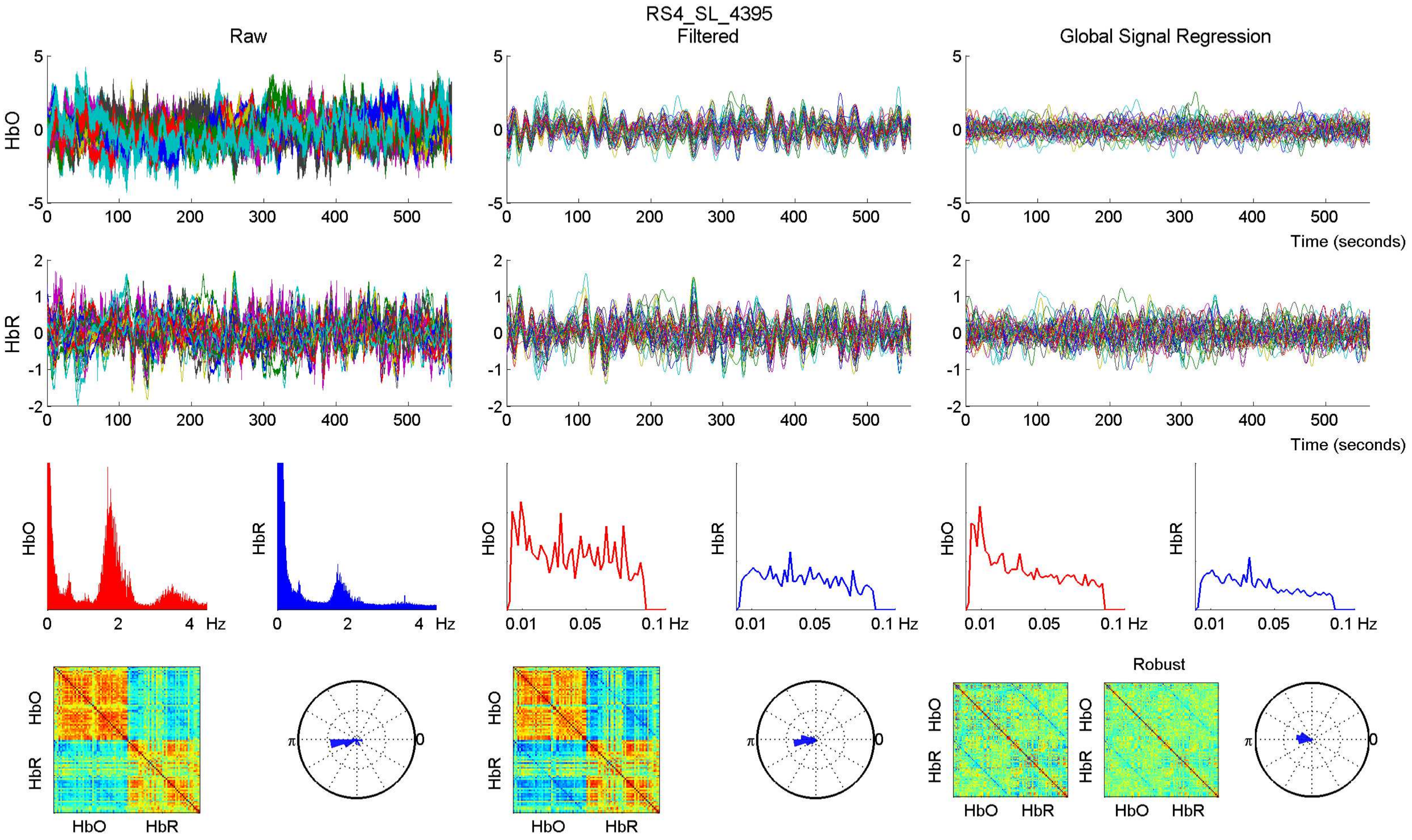

RS4_SL_4395
Raw
Filtered
Global Signal Regression
HbO
HbR
Time (seconds)
Hz
Robust

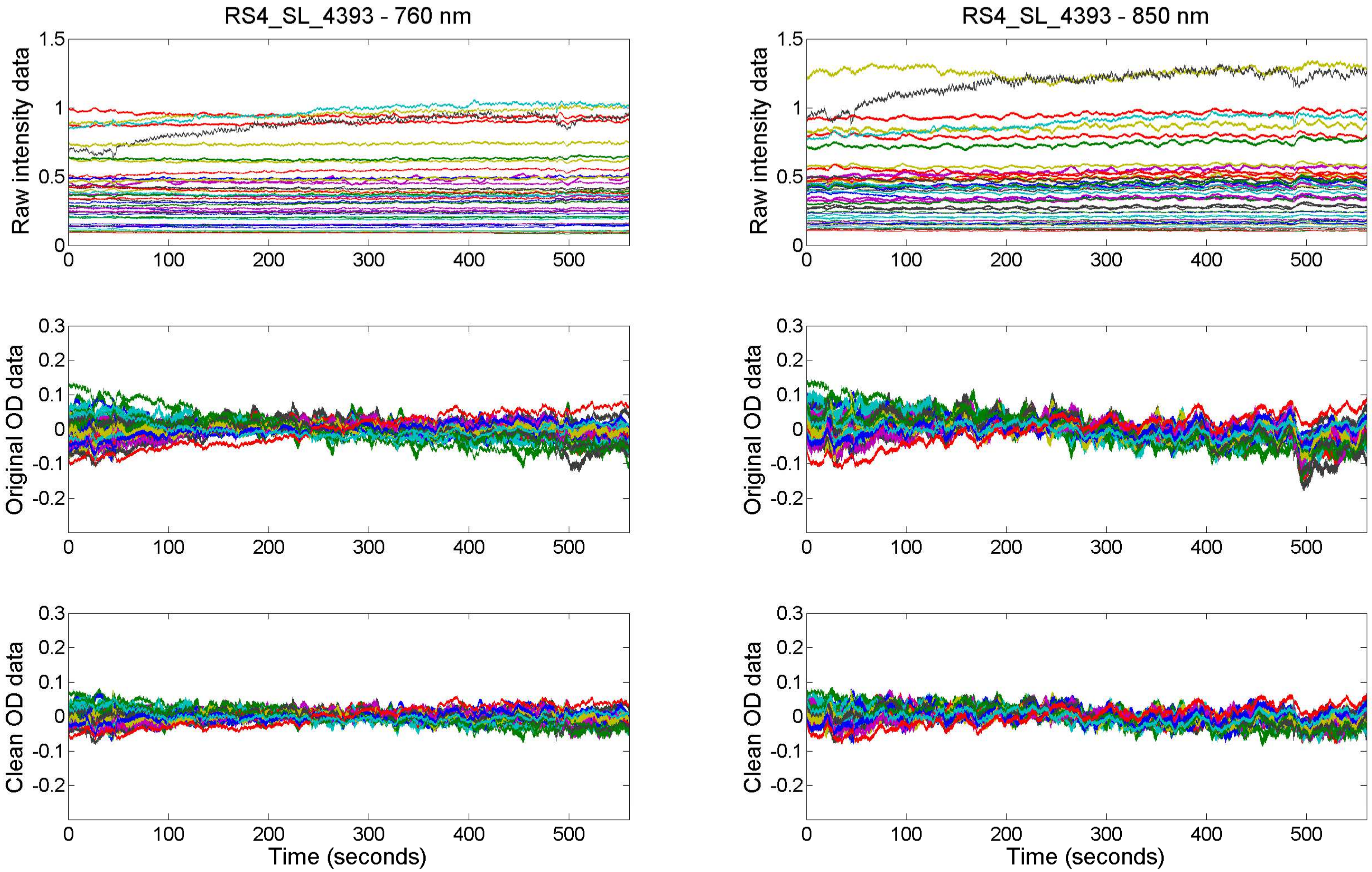

RS4_SL_4393 - 760 nm
RS4_SL_4393 - 850 nm
Raw intensity data
Original OD data
Clean OD data
Time (seconds)

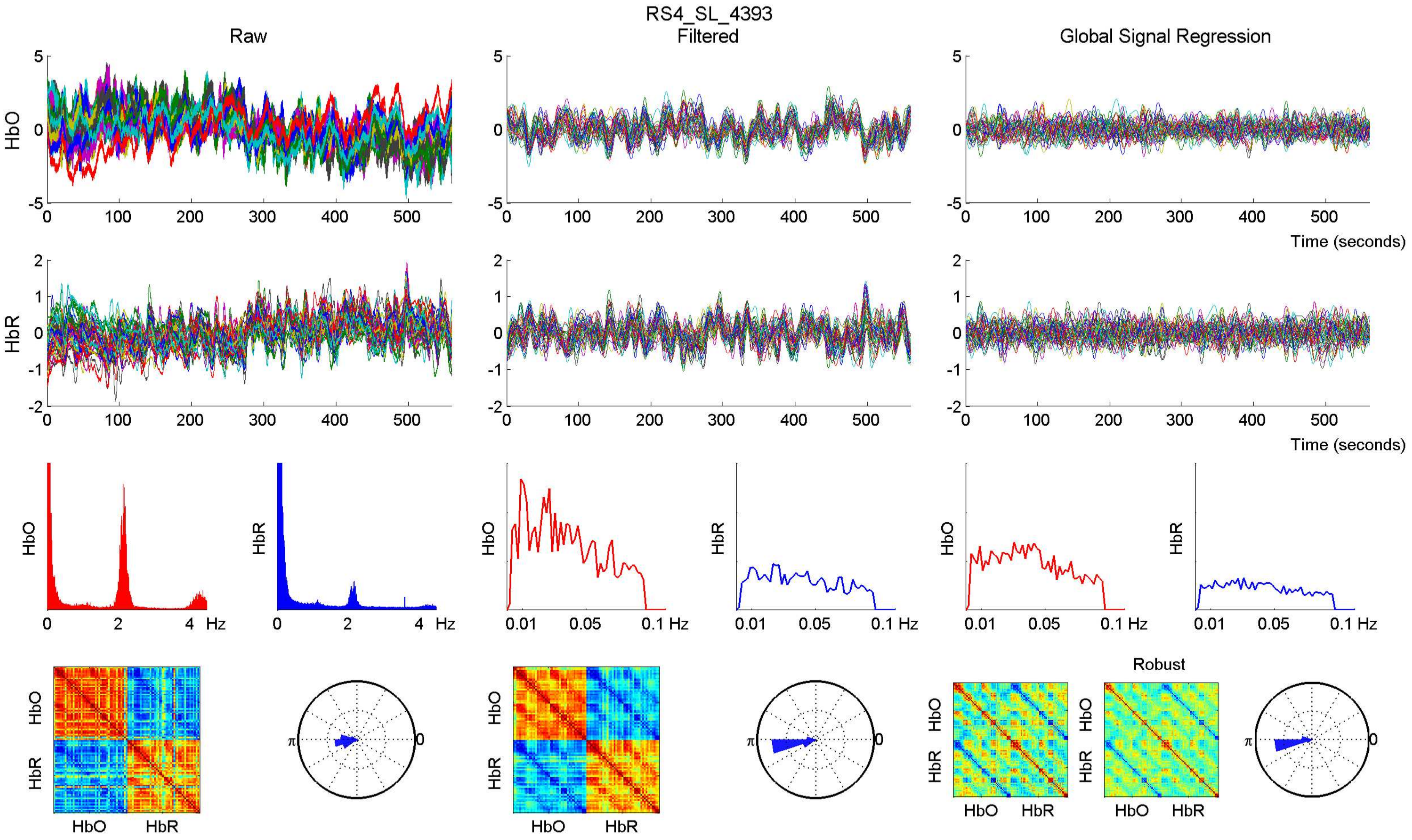
RS4_SL_4393
Raw
Filtered
Global Signal Regression
HbO
HbR
Time (seconds)
Hz
0.01
0.05
0.1 Hz
Robust
π
0

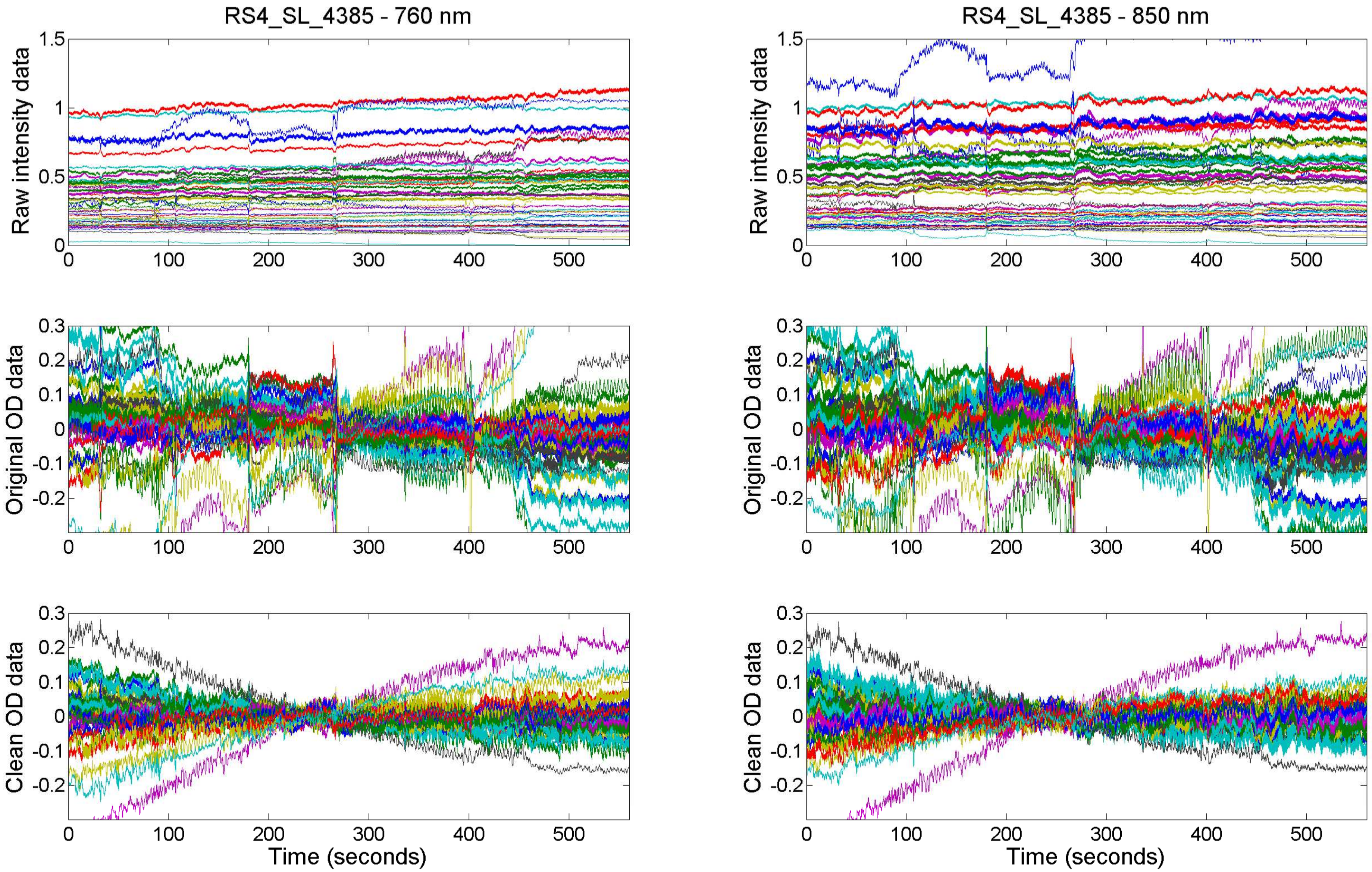
RS4_SL_4385 - 760 nm
RS4_SL_4385 - 850 nm
Raw intensity data
Original OD data
Clean OD data
Time (seconds)

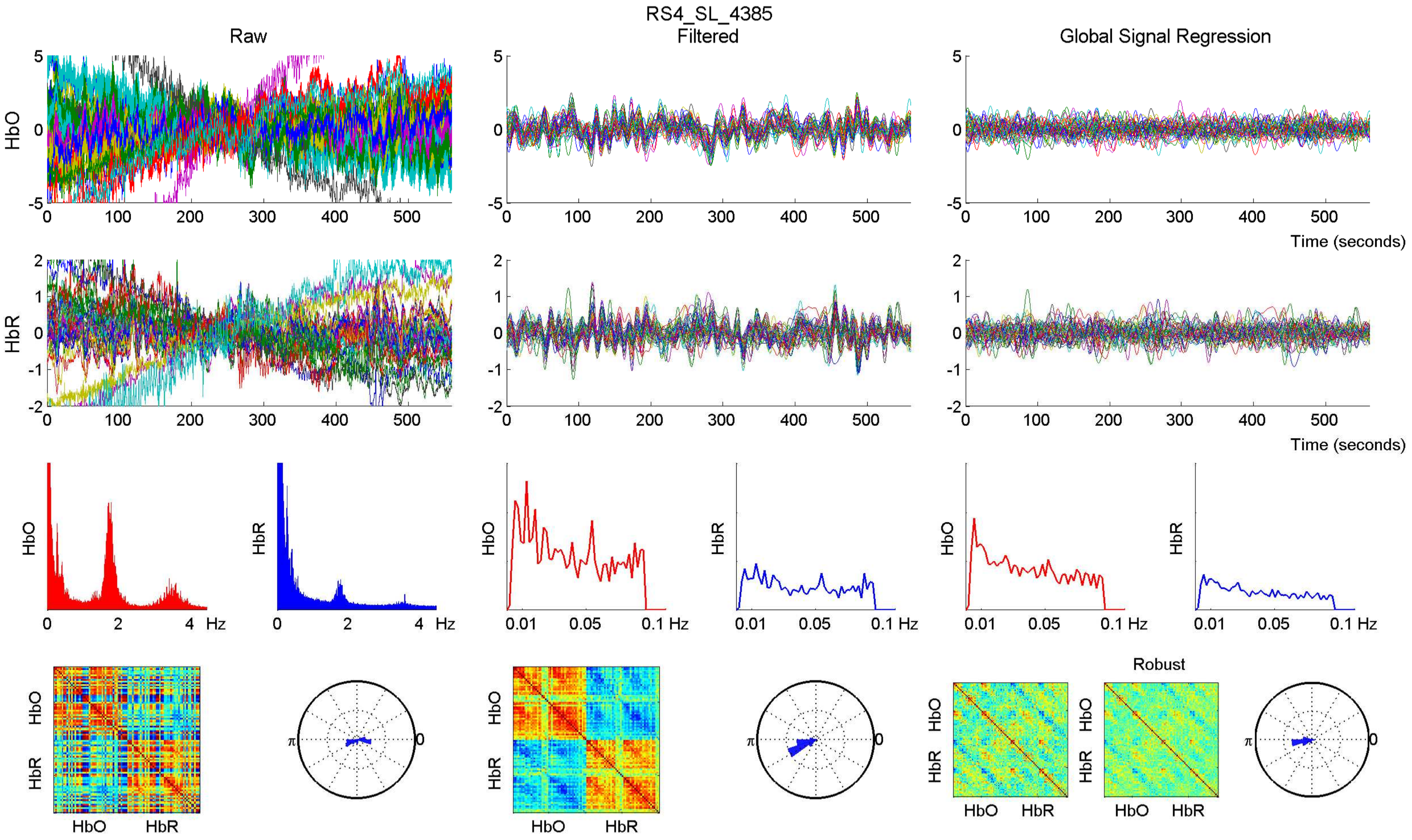

RS4_SL_4385
Raw
Filtered
Global Signal Regression
HbO
HbR
Time (seconds)
Hz
0.01
0.05
0.1 Hz
Robust
π
0

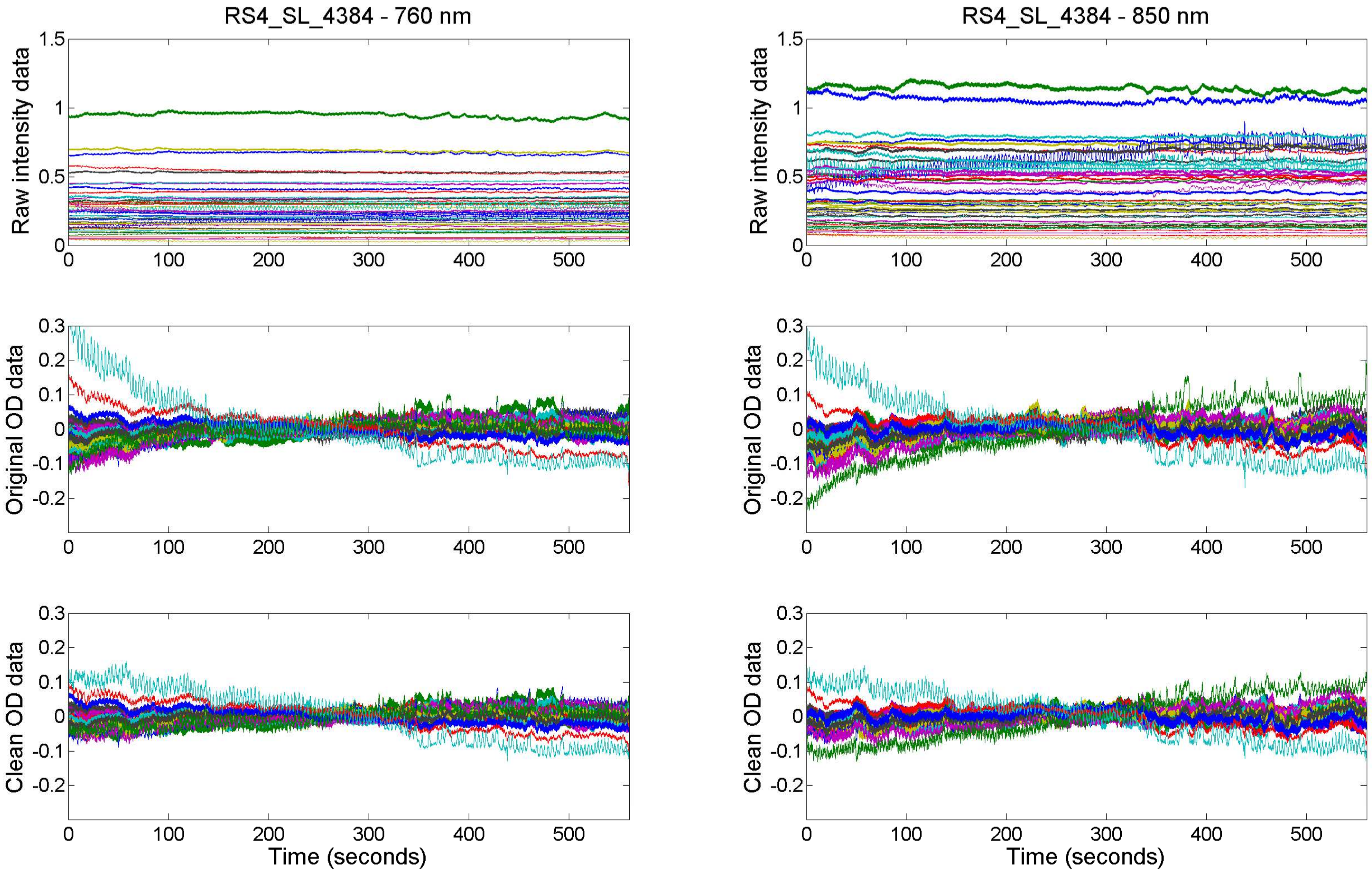
RS4_SL_4384 - 760 nm
RS4_SL_4384 - 850 nm
Raw intensity data
Original OD data
Clean OD data
Time (seconds)
0
100
200
300
400
500
1.5
1
0.5
0.3
0.2
0.1
-0.1
-0.2

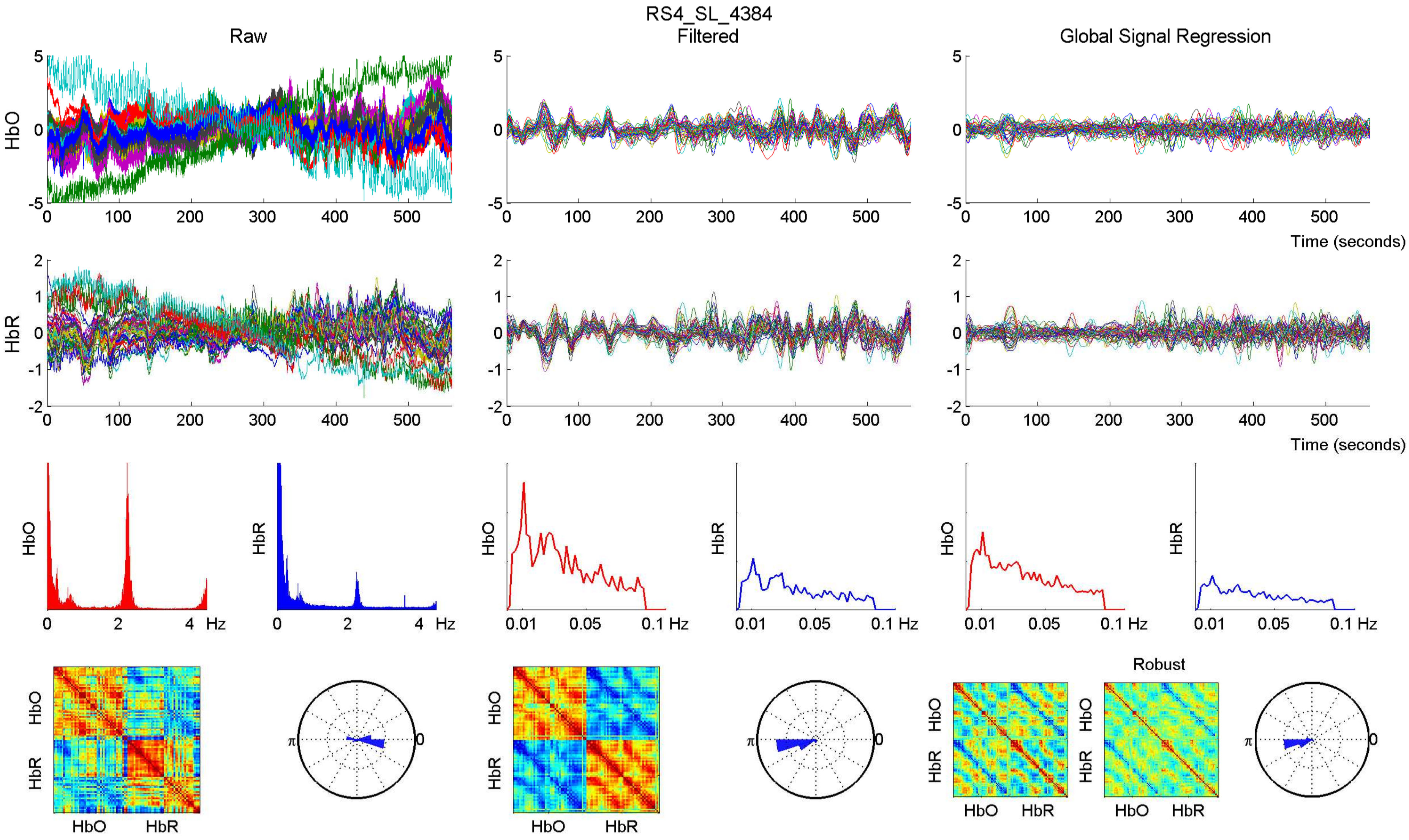
RS4_SL_4384
Raw
Filtered
Global Signal Regression
HbO
HbR
Time (seconds)
Hz
Robust

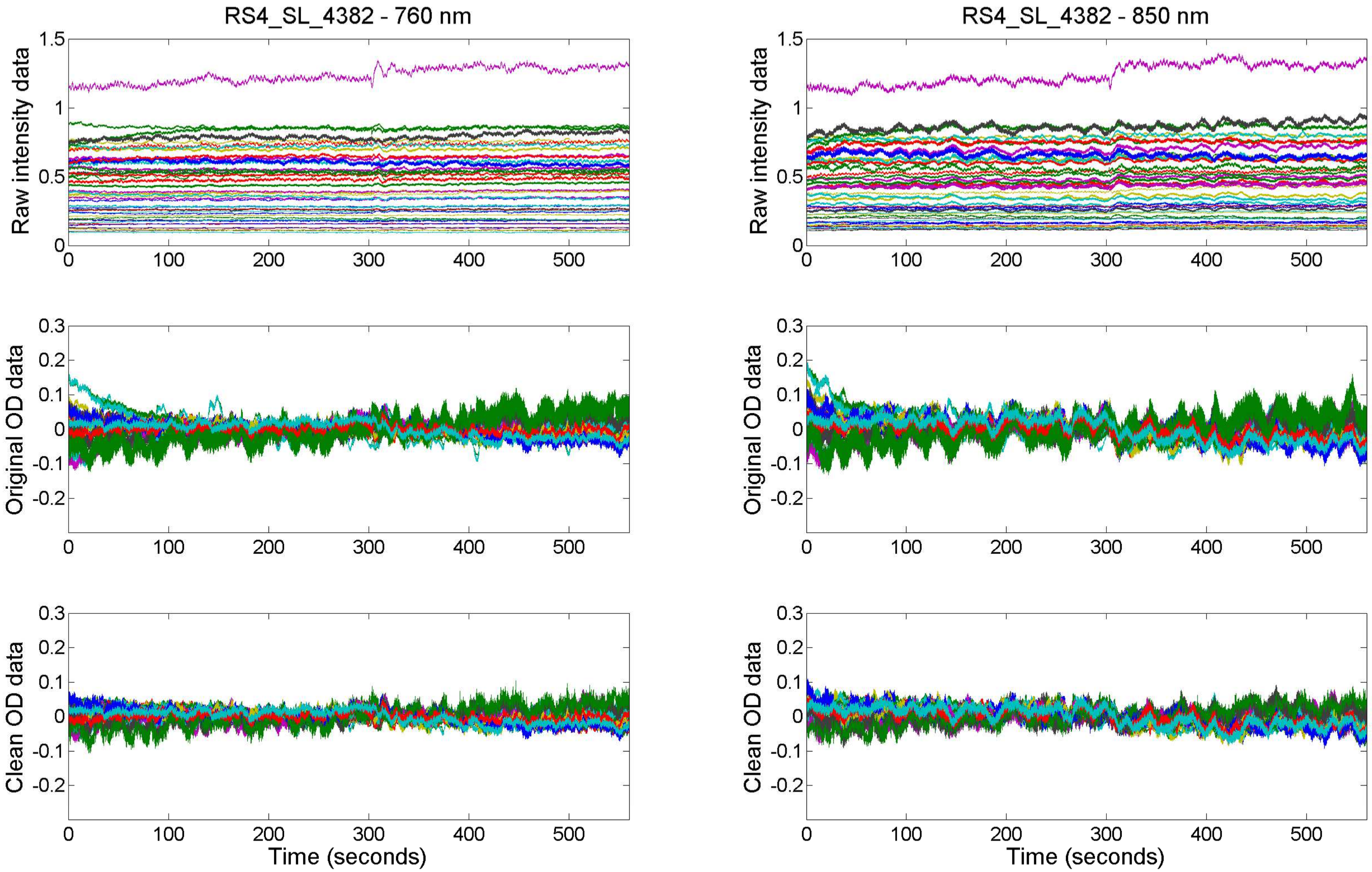
RS4_SL_4382 - 760 nm
RS4_SL_4382 - 850 nm
Raw intensity data
Original OD data
Clean OD data
Time (seconds)
0
100
200
300
400
500
1.5
1
0.5
0.3
0.2
0.1
-0.1
-0.2

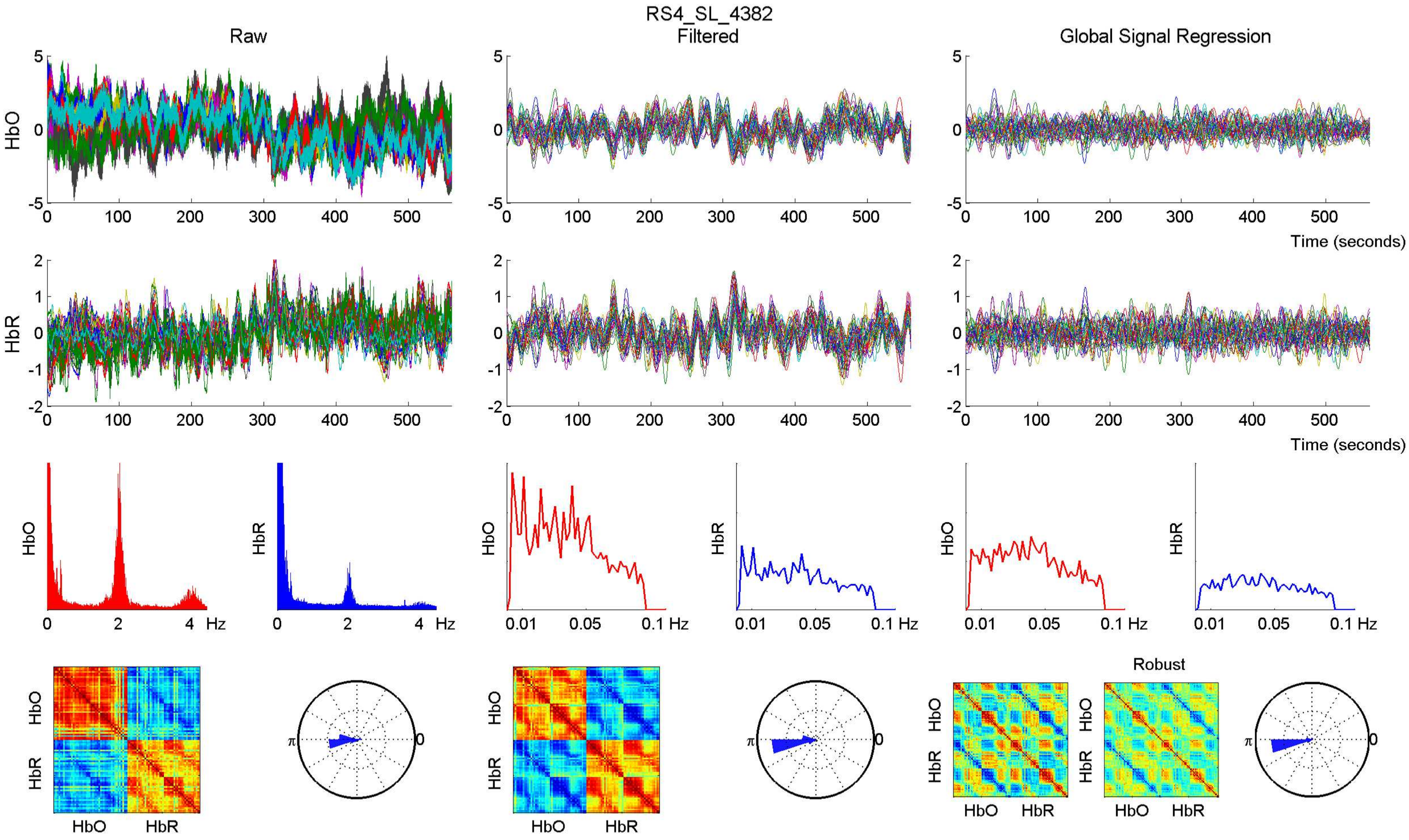
RS4_SL_4382
Raw
Filtered
Global Signal Regression
HbO
HbR
Time (seconds)
Hz
0.01
0.05
0.1 Hz
Robust
π
0

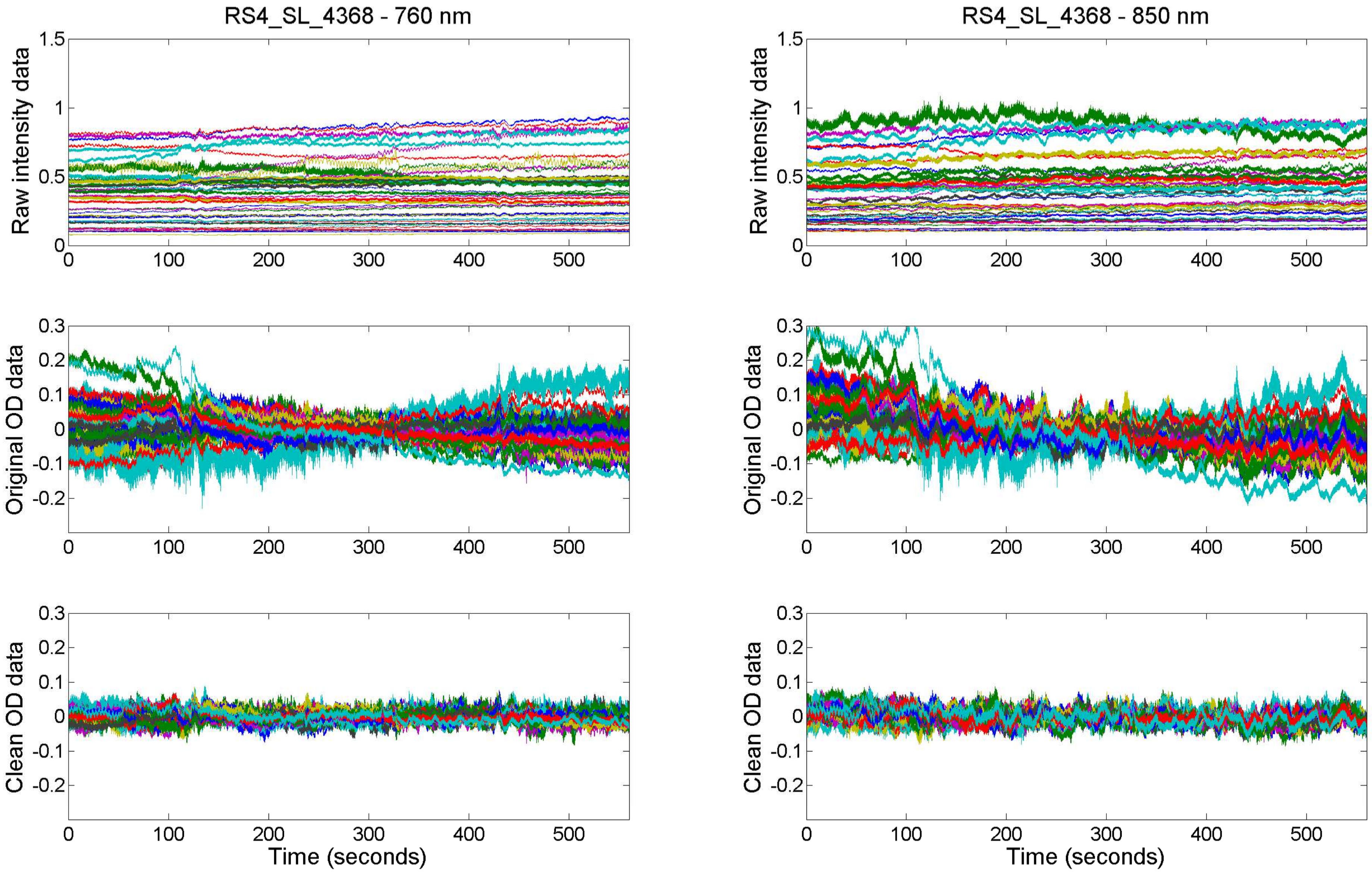
RS4_SL_4368 - 760 nm
RS4_SL_4368 - 850 nm
Raw intensity data
Original OD data
Clean OD data
Time (seconds)
0
100
200
300
400
500
1.5
1
0.5
0.3
0.2
0.1
-0.1
-0.2

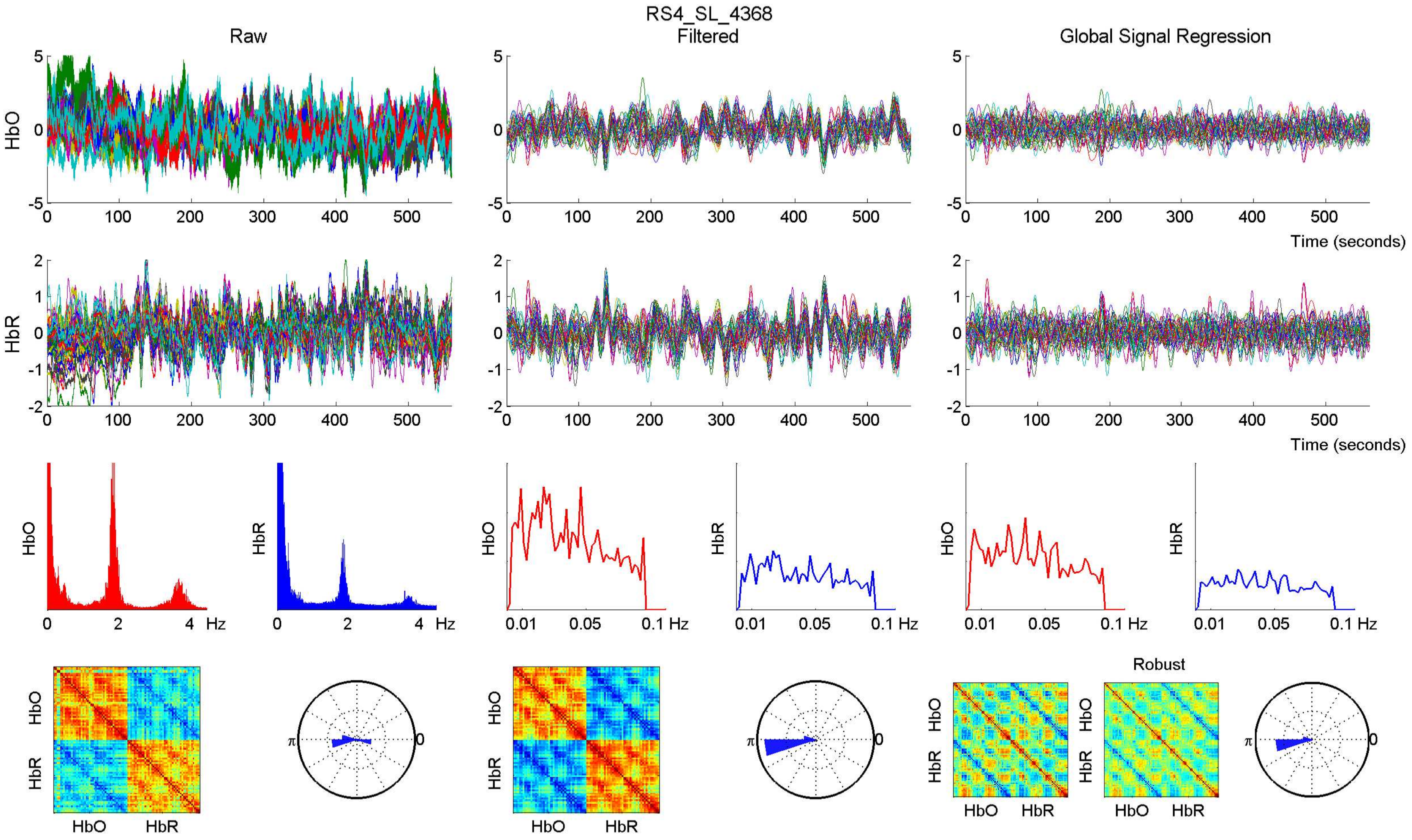

RS4_SL_4368
Raw
Filtered
Global Signal Regression
HbO
HbR
Time (seconds)
Hz
0.01
0.05
0.1 Hz
Robust
HbO
HbR
π
0

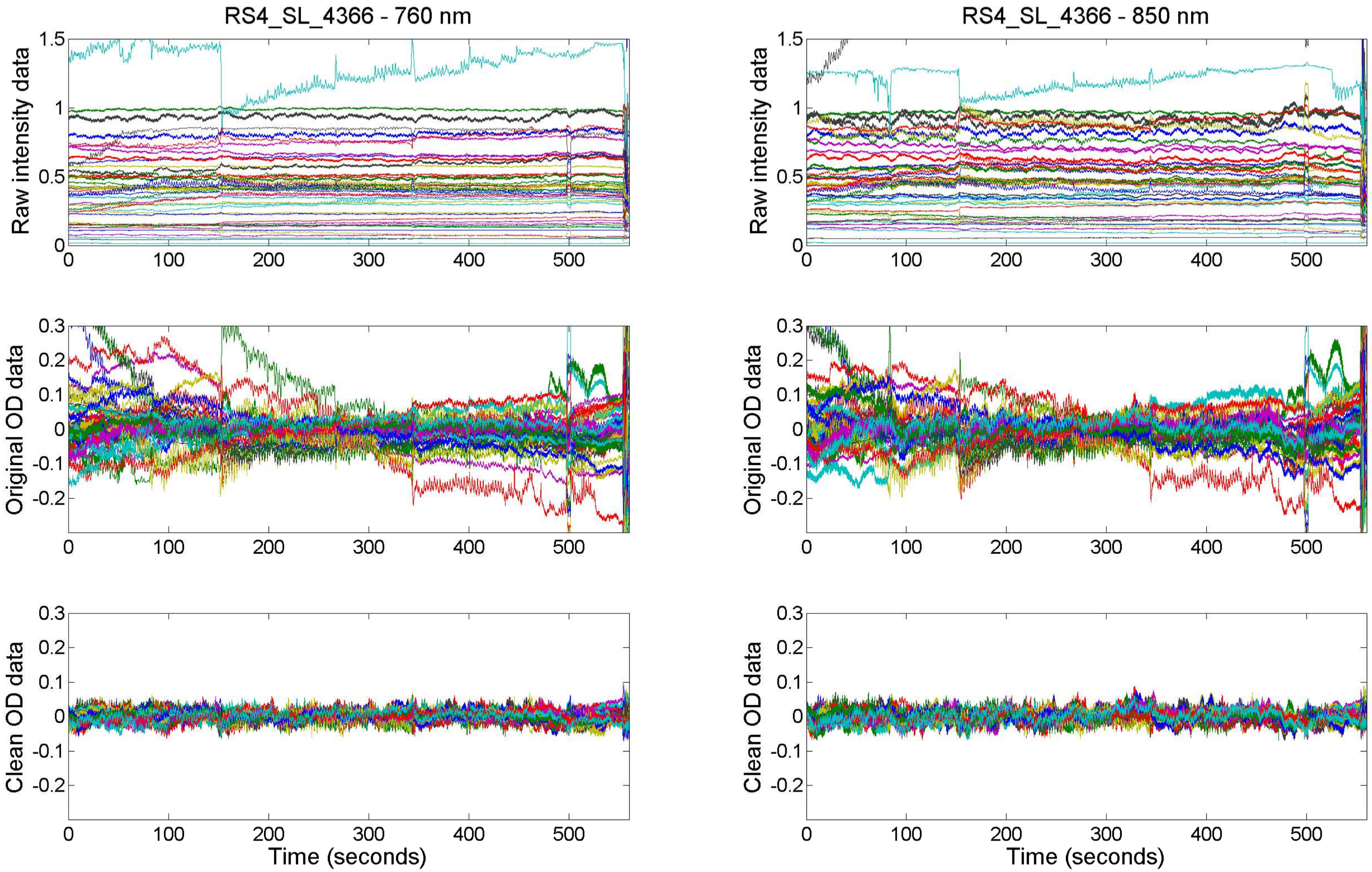

RS4_SL_4366 - 760 nm
RS4_SL_4366 - 850 nm
Raw intensity data
Original OD data
Clean OD data
Time (seconds)

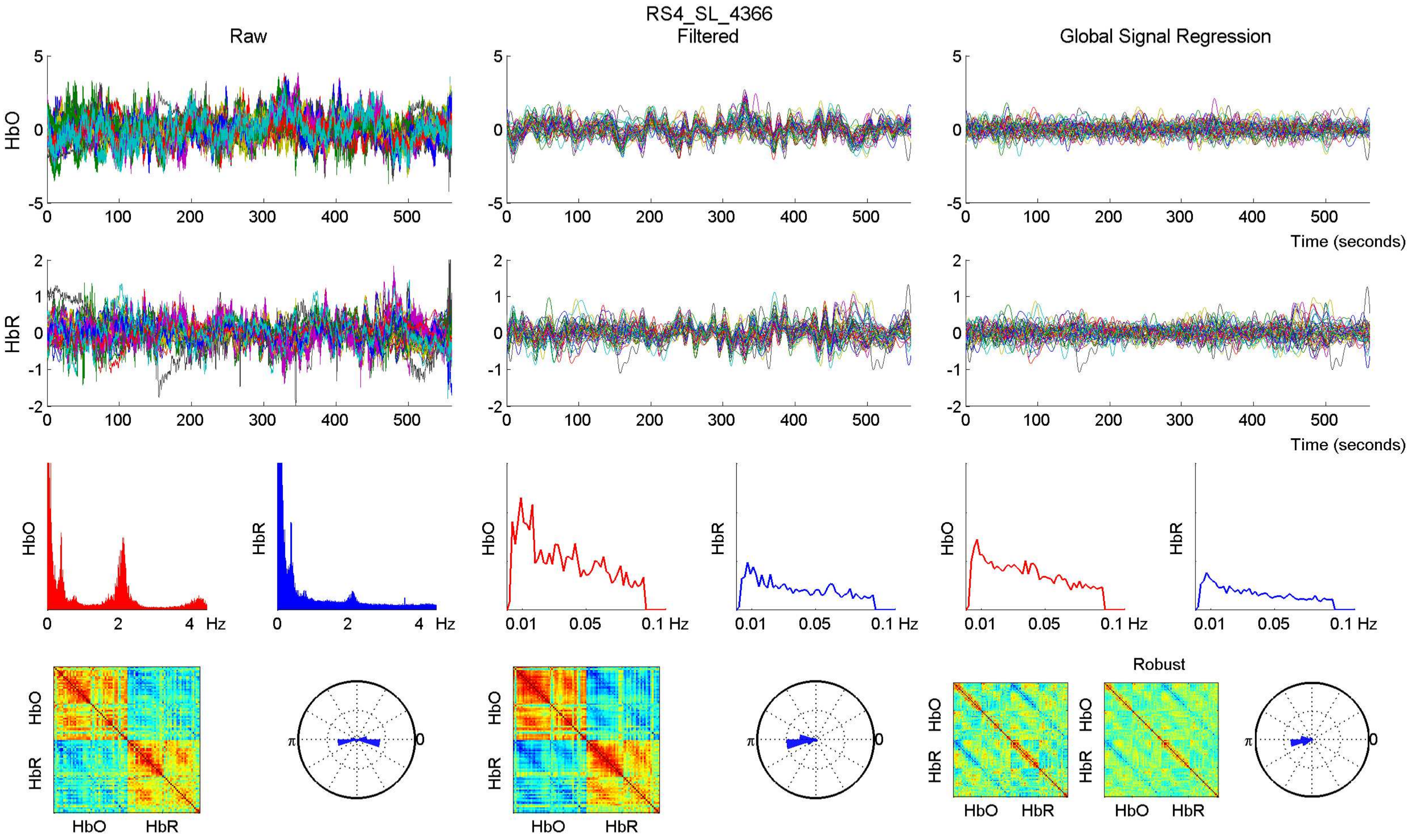

RS4_SL_4366
Raw
Filtered
Global Signal Regression
HbO
HbR
Time (seconds)
Hz
Robust

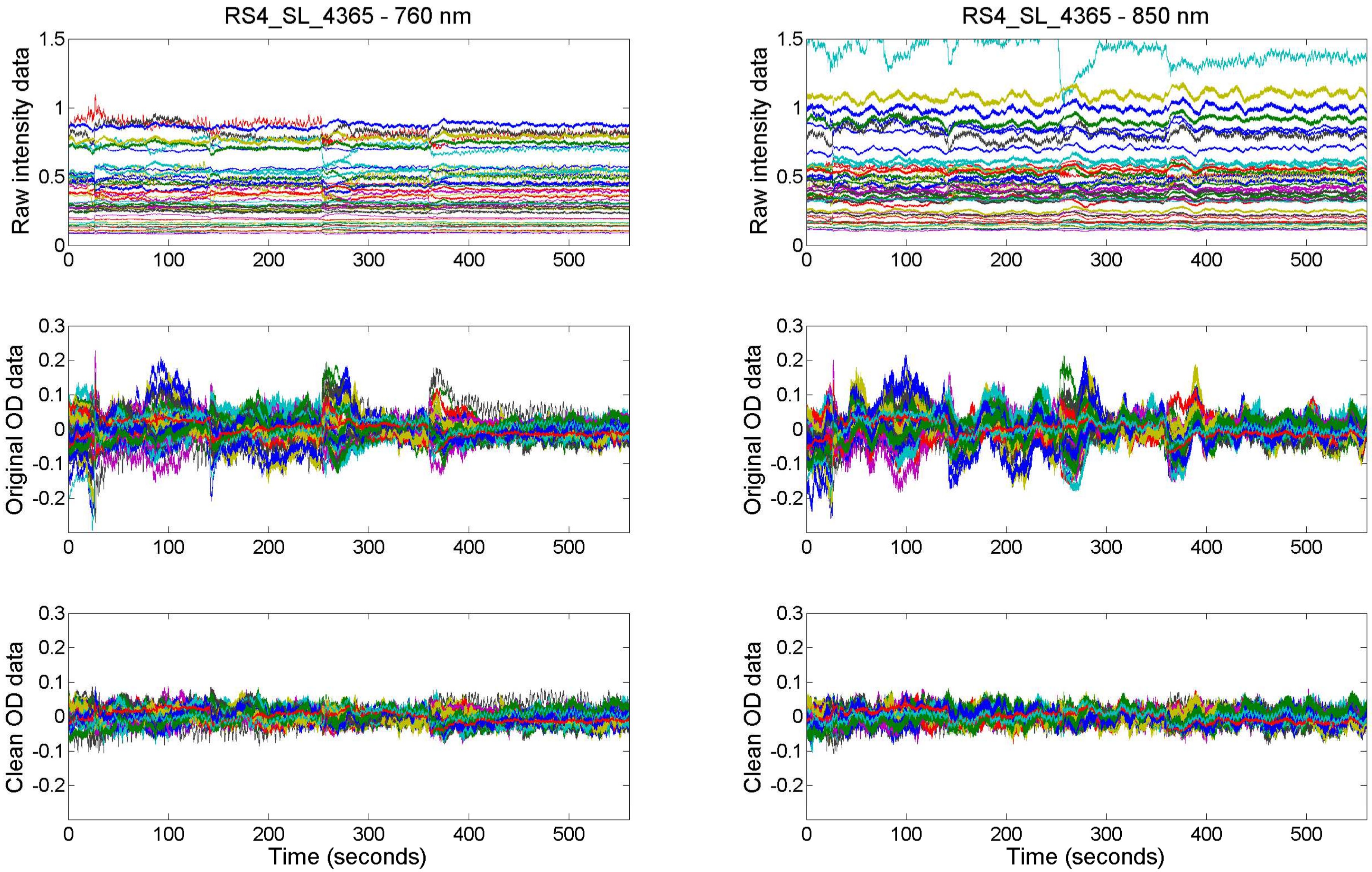

RS4_SL_4365 - 760 nm
RS4_SL_4365 - 850 nm
Raw intensity data
Original OD data
Clean OD data
Time (seconds)

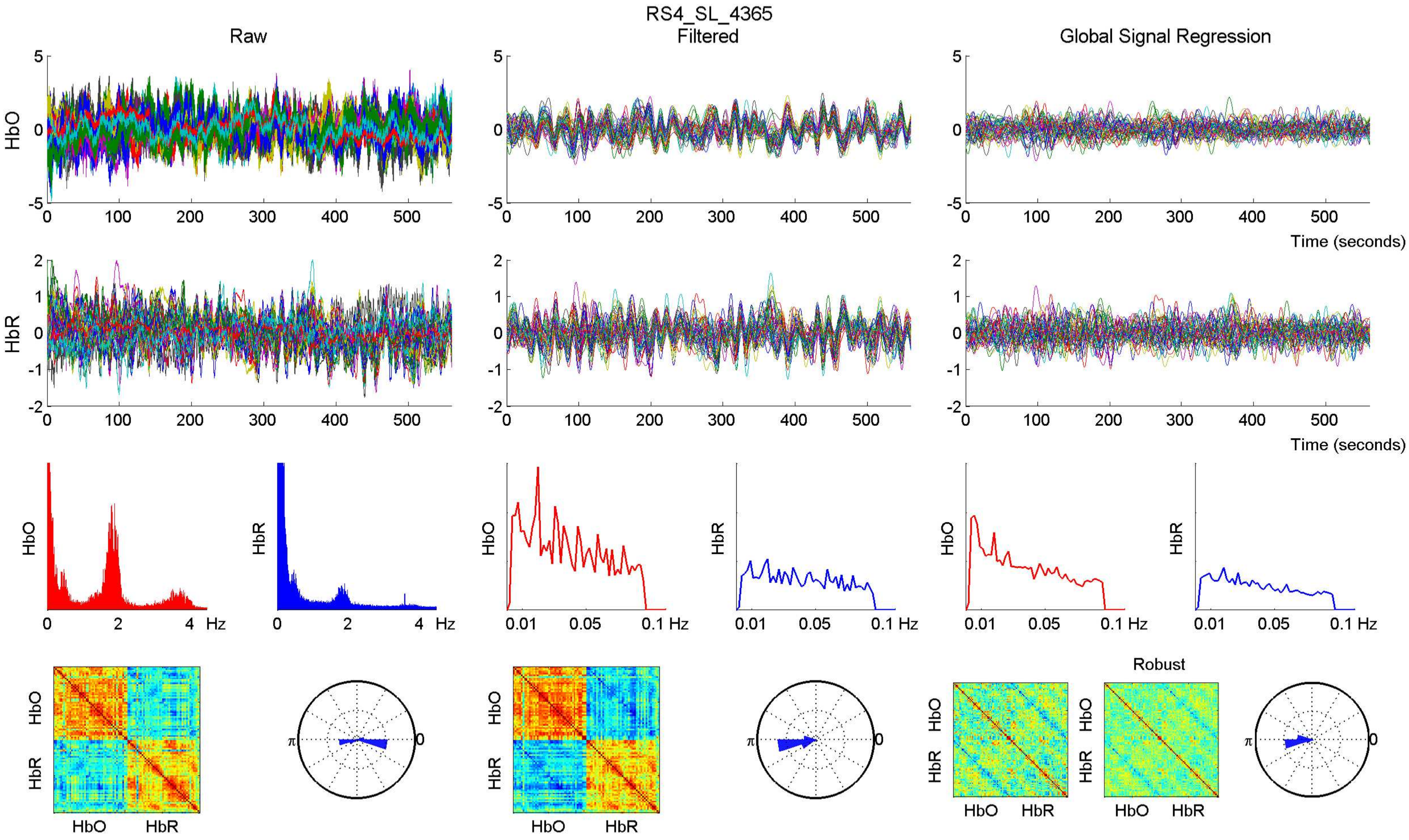

RS4_SL_4365
Raw
Filtered
Global Signal Regression
HbO
HbR
Time (seconds)
Hz
Robust

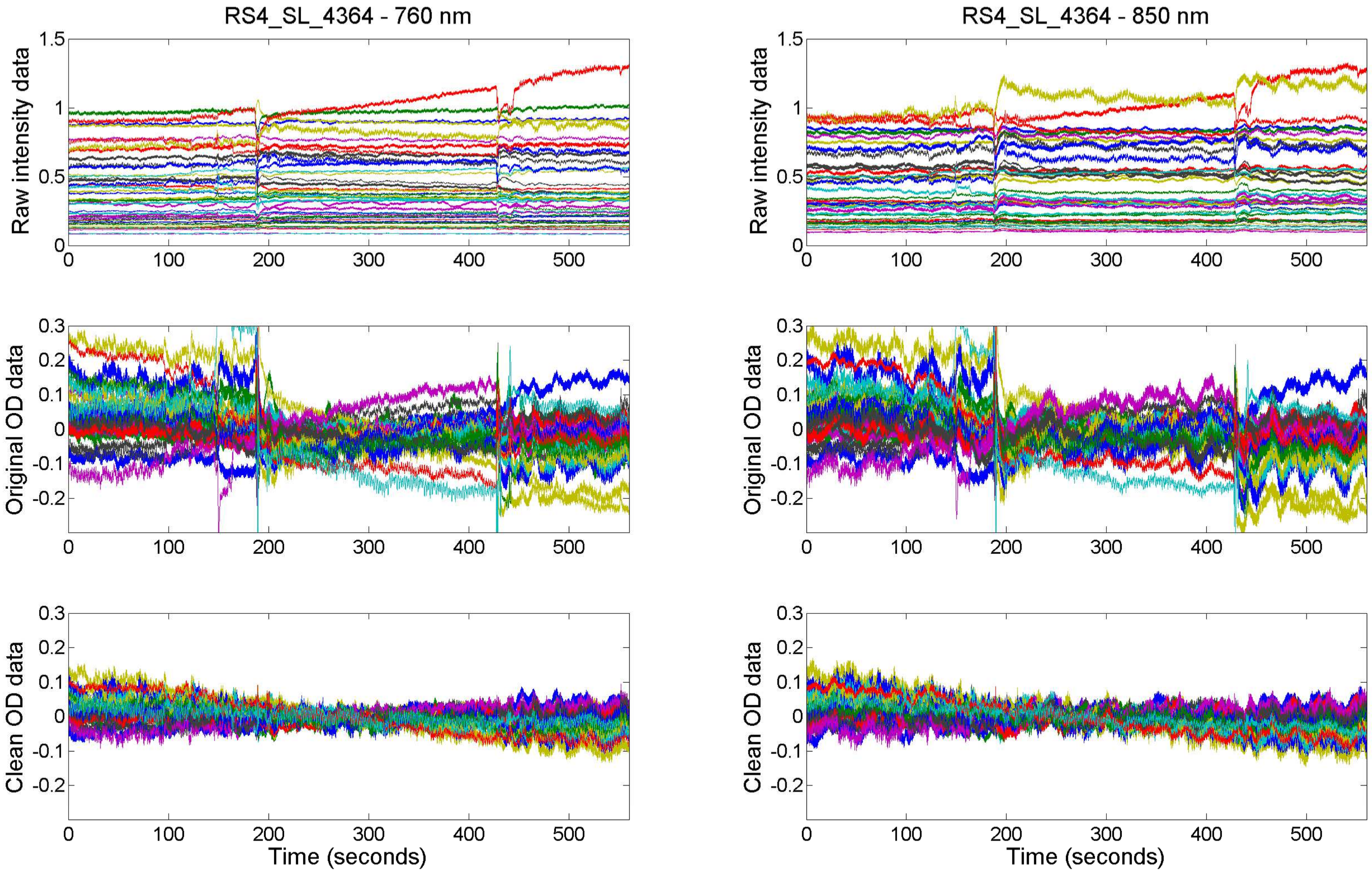
RS4_SL_4364 - 760 nm
RS4_SL_4364 - 850 nm
Raw intensity data
Original OD data
Clean OD data
Time (seconds)
1.5
1
0.5
0
0.3
0.2
0.1
-0.1
-0.2
100
200
300
400
500

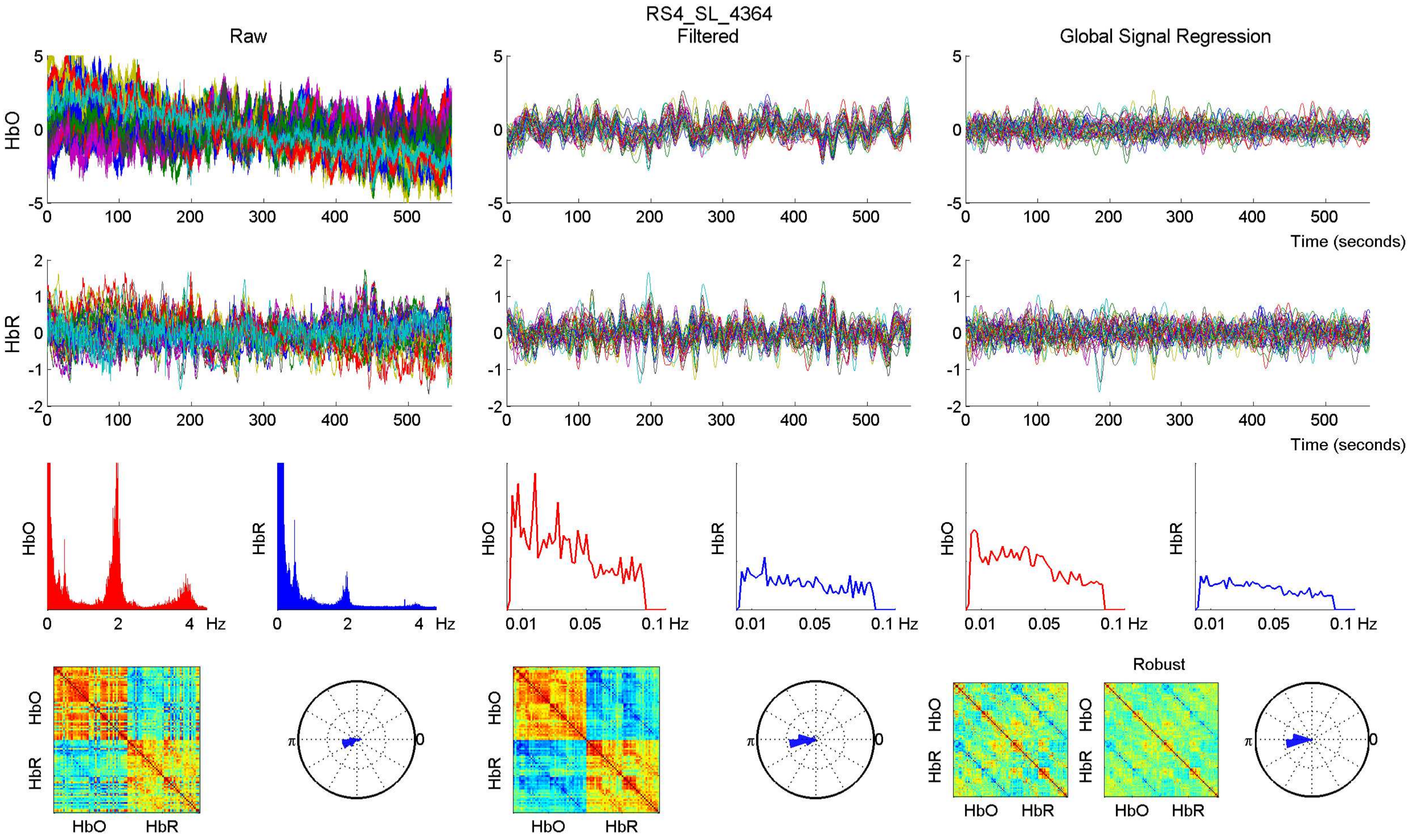
RS4_SL_4364
Raw
Filtered
Global Signal Regression
HbO
HbR
Time (seconds)
Hz
Robust

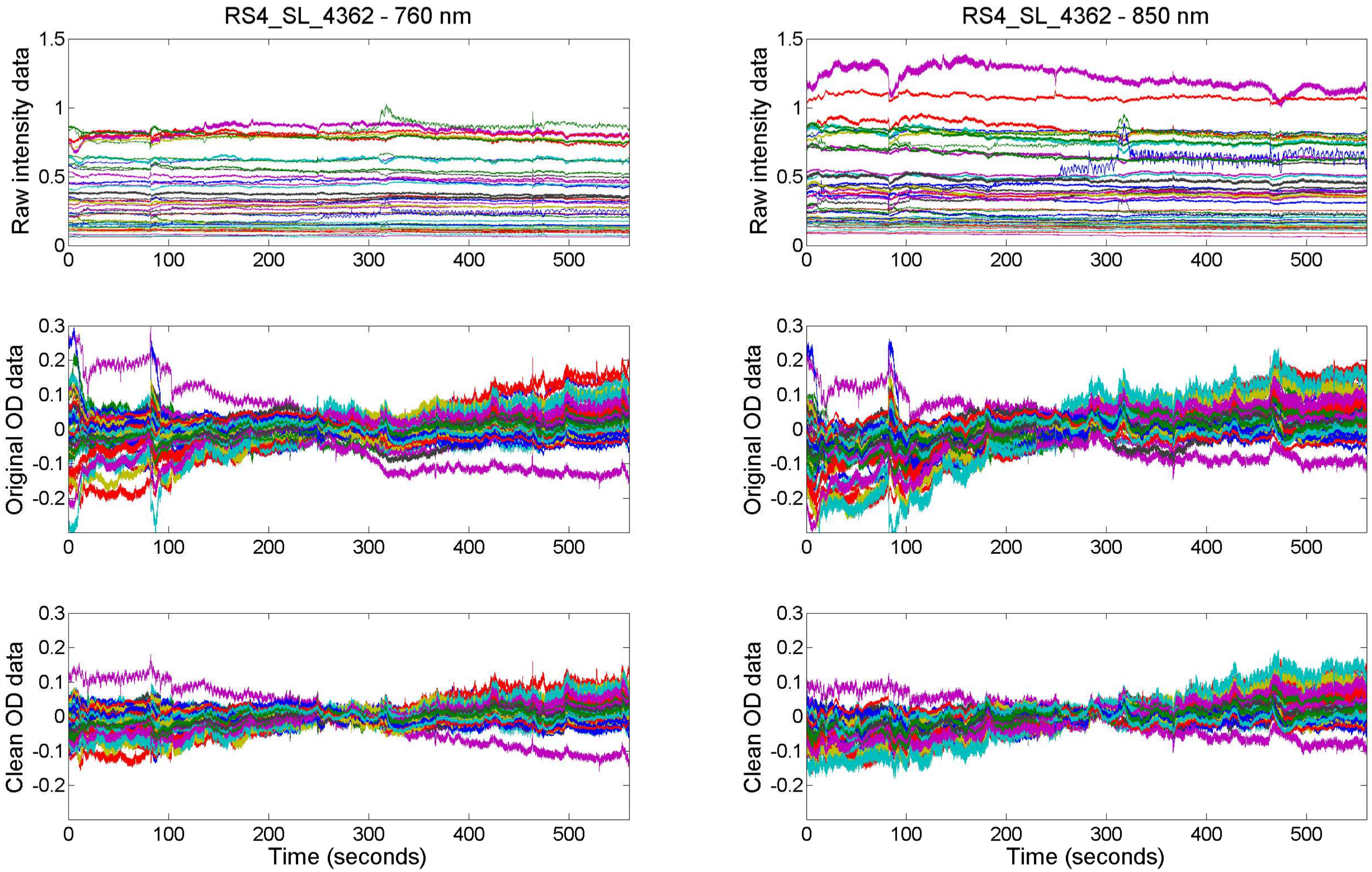

RS4_SL_4362 - 760 nm
RS4_SL_4362 - 850 nm
Raw intensity data
Original OD data
Clean OD data
Time (seconds)

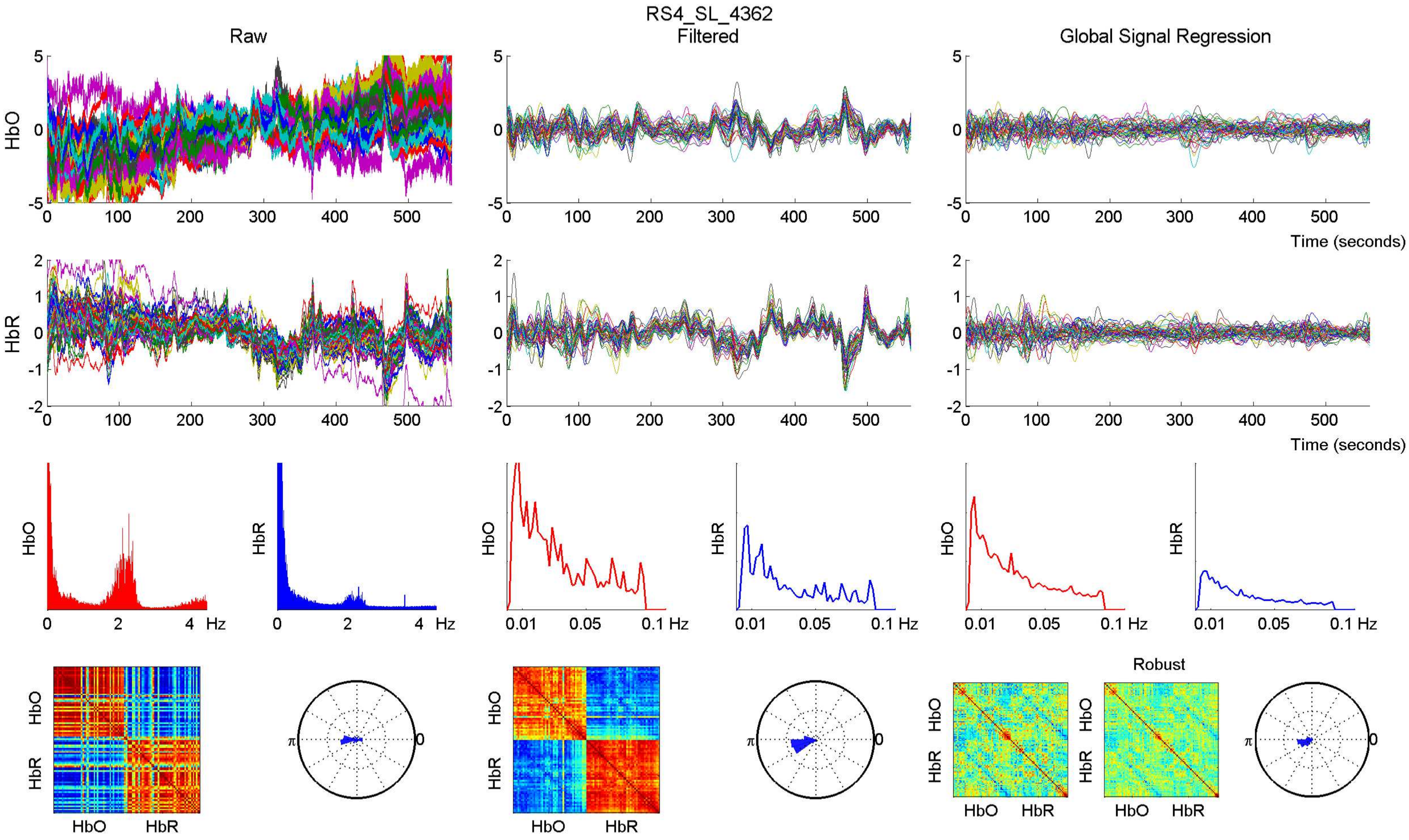

RS4_SL_4362
Raw
Filtered
Global Signal Regression
HbO
HbR
Time (seconds)
Hz
0.01
0.05
0.1 Hz
Robust
HbO
HbR

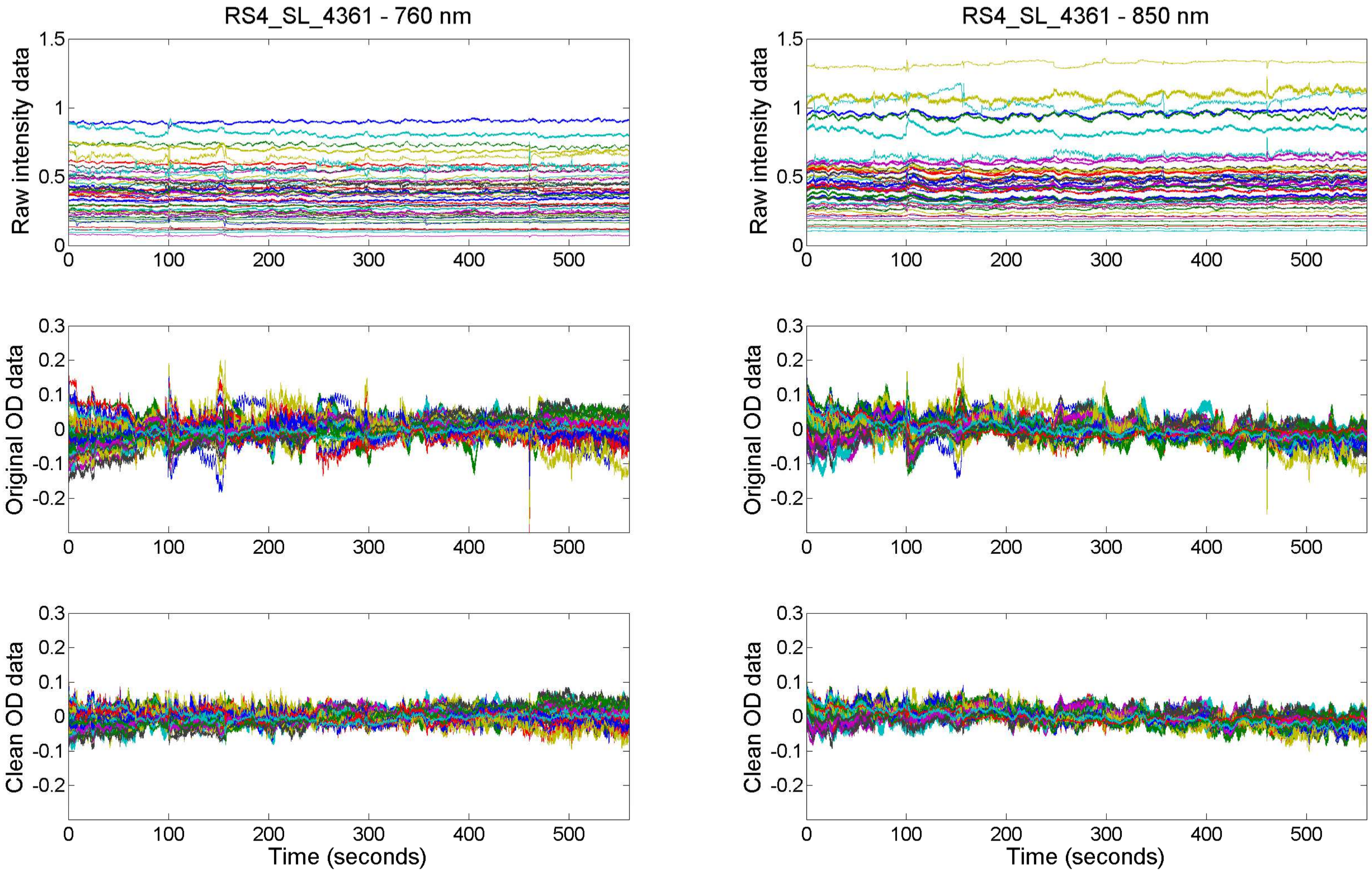
RS4_SL_4361 - 760 nm
RS4_SL_4361 - 850 nm
Raw intensity data
Original OD data
Clean OD data
Time (seconds)

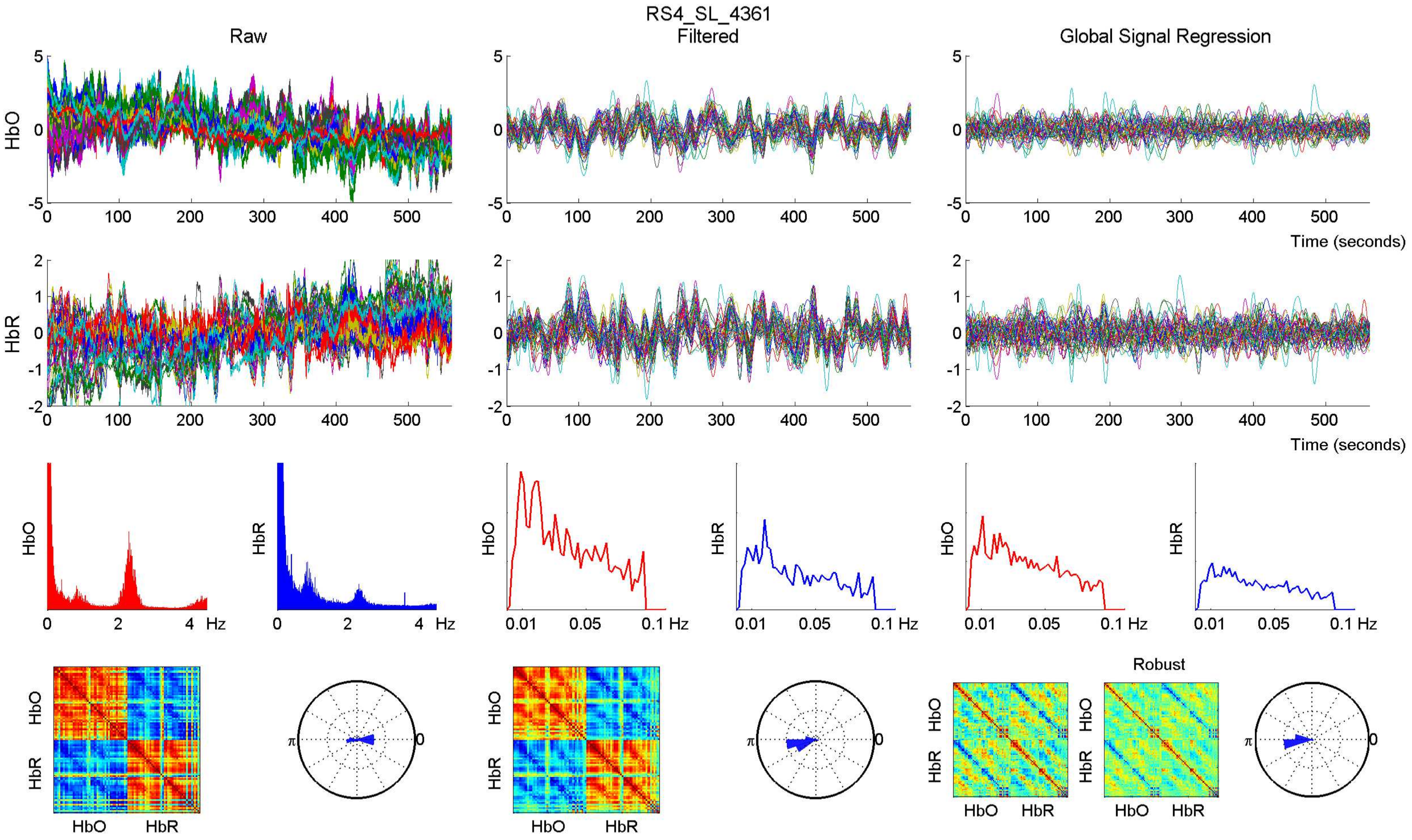

RS4_SL_4361
Raw
Filtered
Global Signal Regression
HbO
HbR
Time (seconds)
Hz
Robust

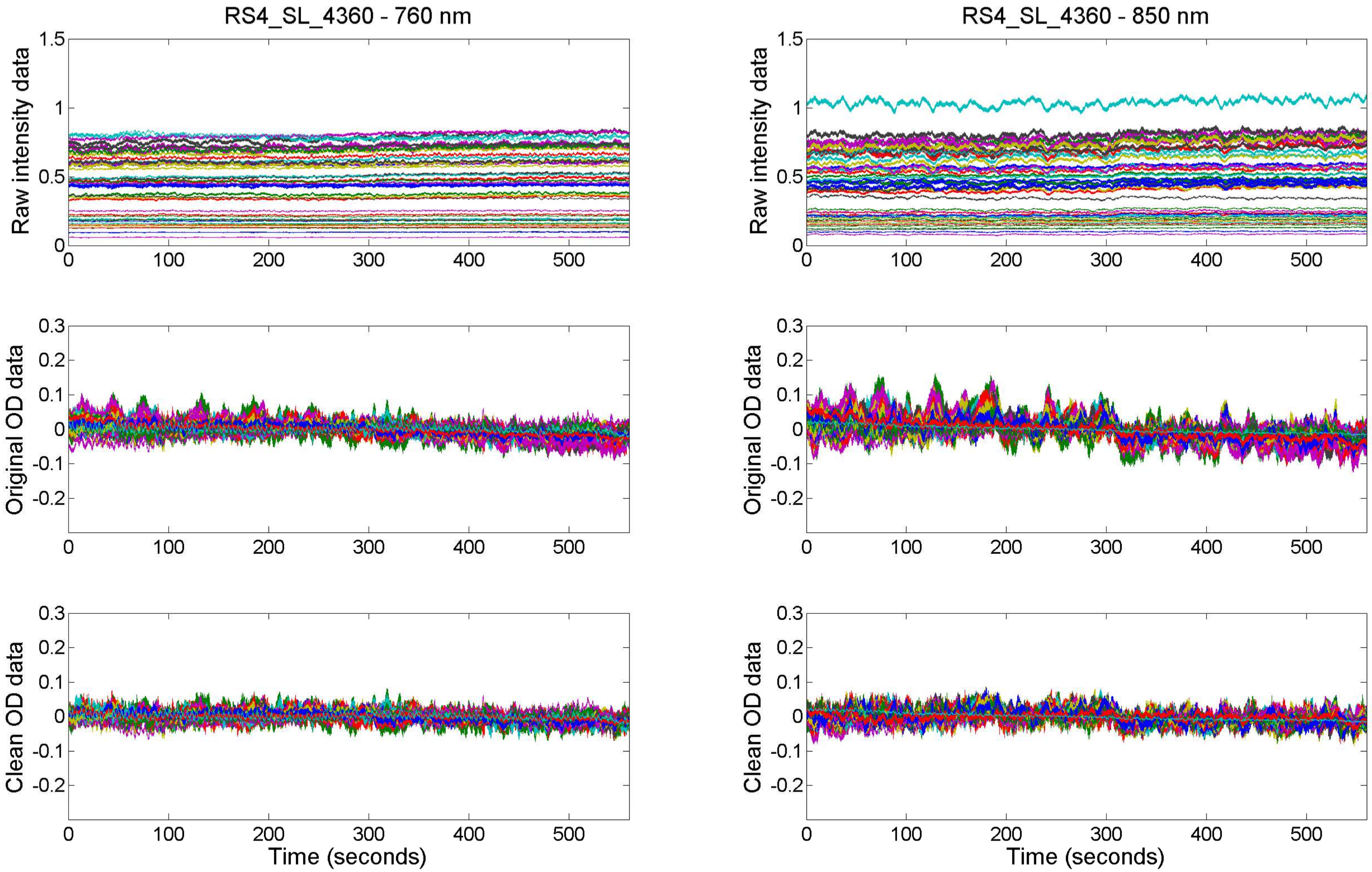

RS4_SL_4360 - 760 nm
RS4_SL_4360 - 850 nm
Raw intensity data
Original OD data
Clean OD data
Time (seconds)

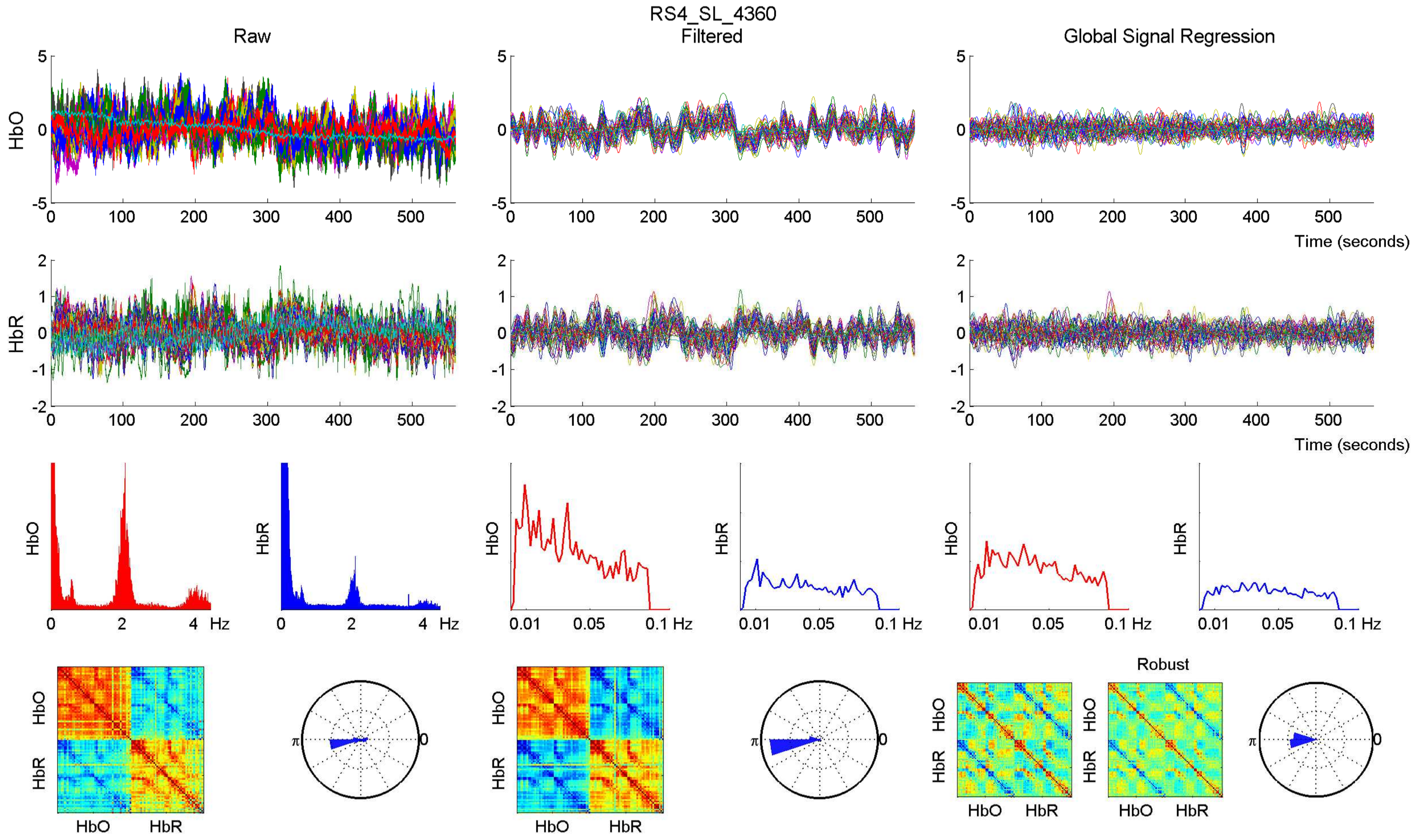
RS4_SL_4360
Raw
Filtered
Global Signal Regression
HbO
HbR
Time (seconds)
Hz
Robust

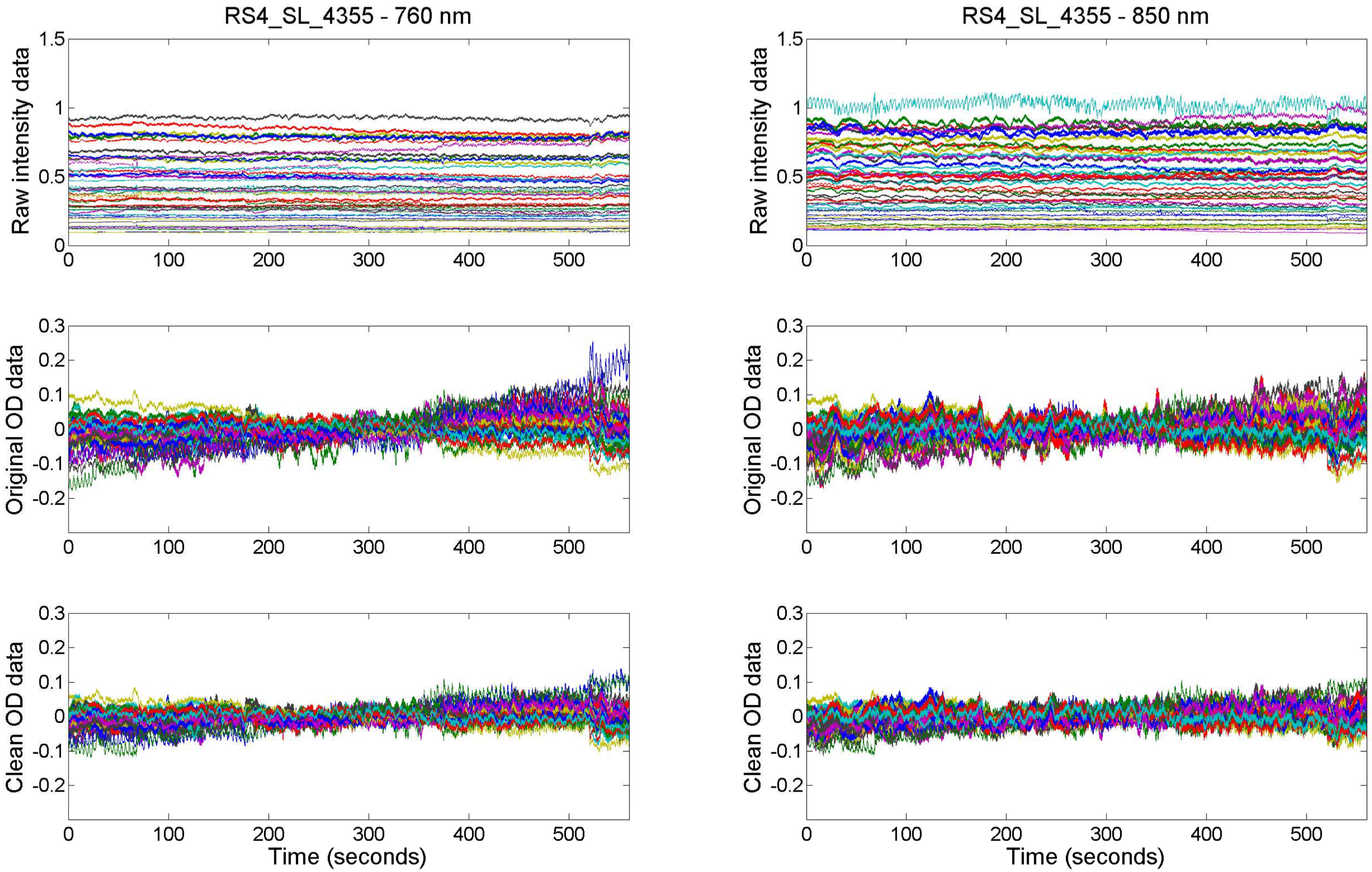

RS4_SL_4355 - 760 nm
RS4_SL_4355 - 850 nm
Raw intensity data
Original OD data
Clean OD data
Time (seconds)

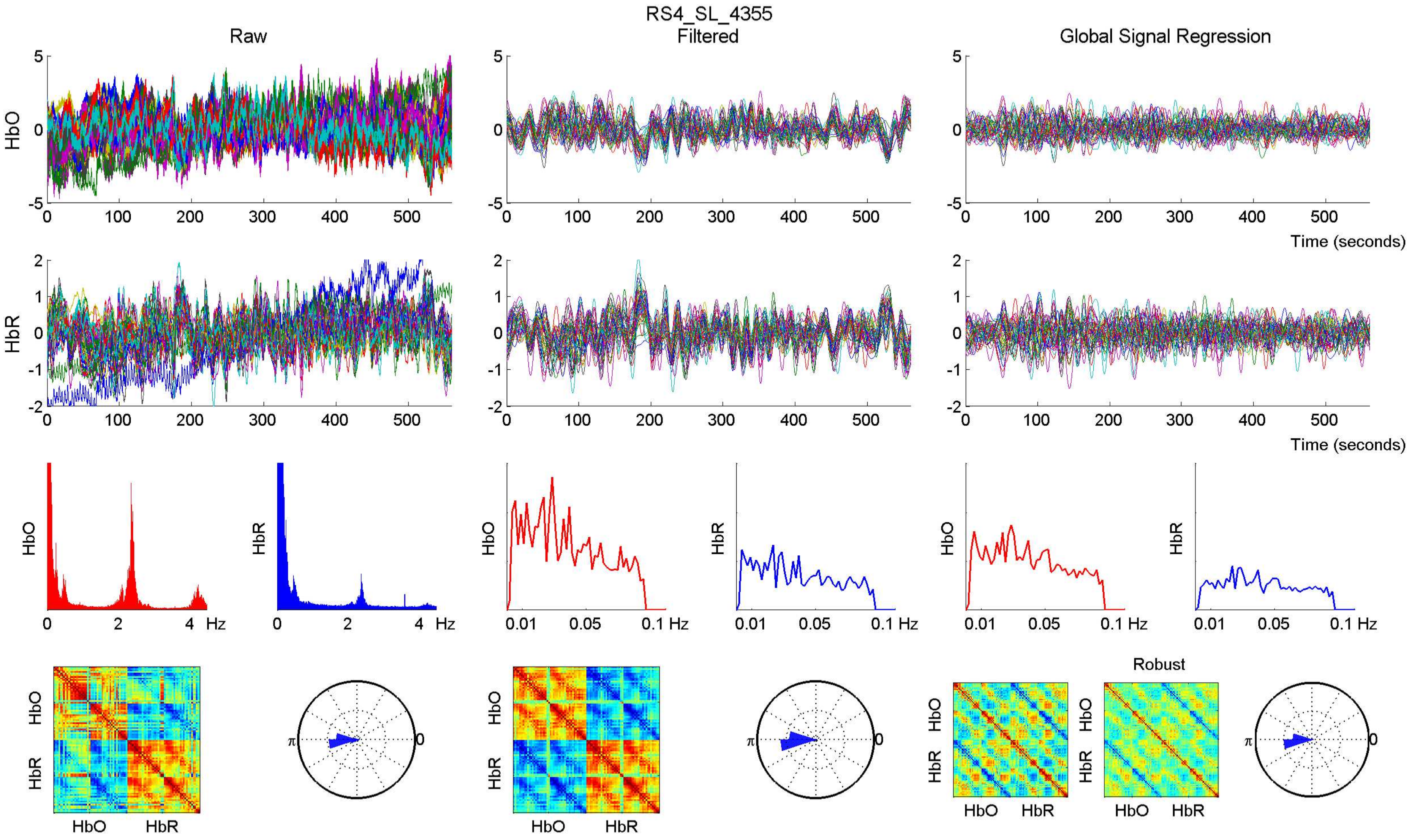

RS4_SL_4355
Raw
Filtered
Global Signal Regression
HbO
HbR
Time (seconds)
Hz
Robust

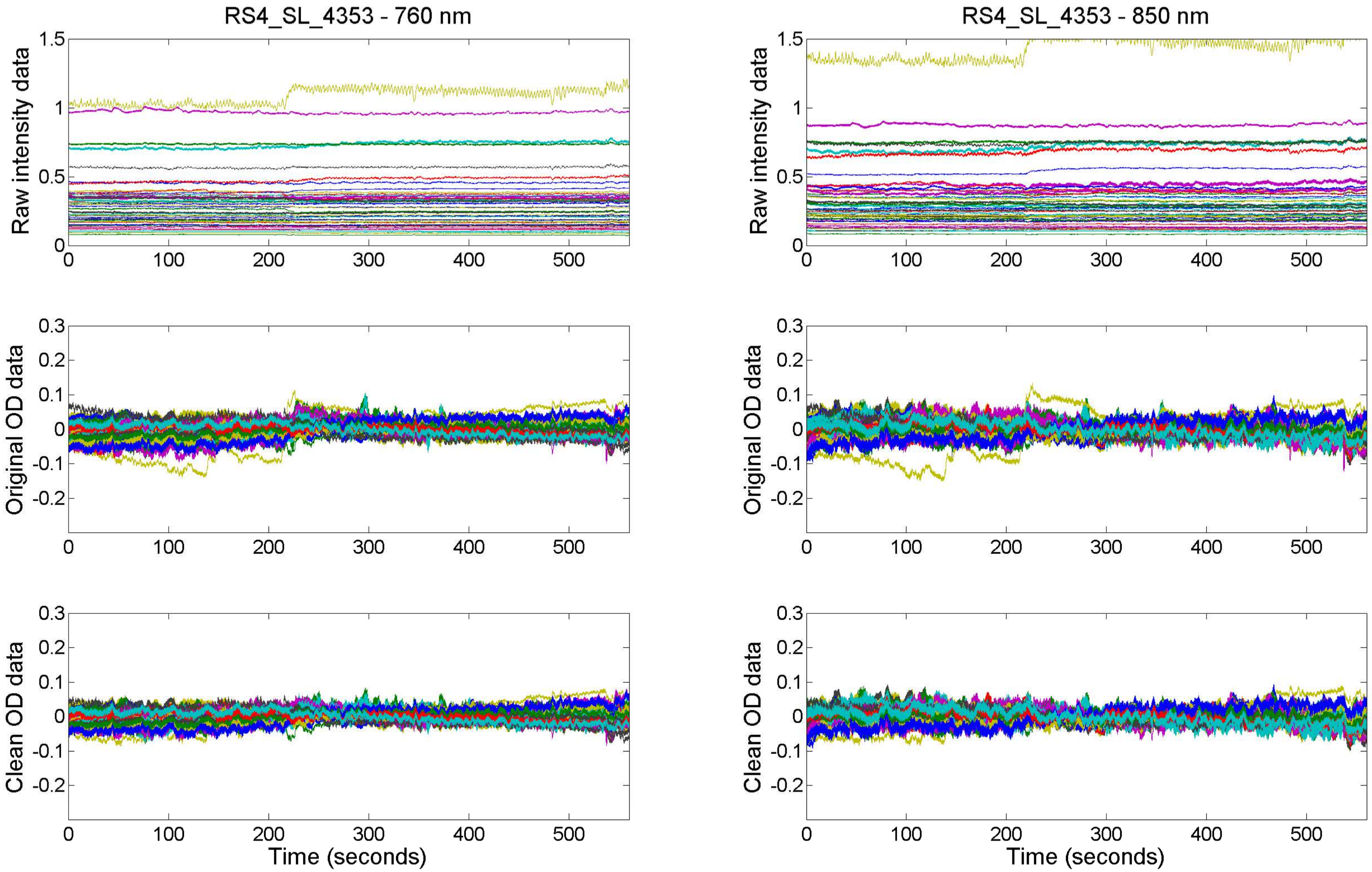
RS4_SL_4353 - 760 nm
RS4_SL_4353 - 850 nm
Raw intensity data
Original OD data
Clean OD data
Time (seconds)
1.5
1
0.5
0
0.3
0.2
0.1
-0.1
-0.2
100
200
300
400
500

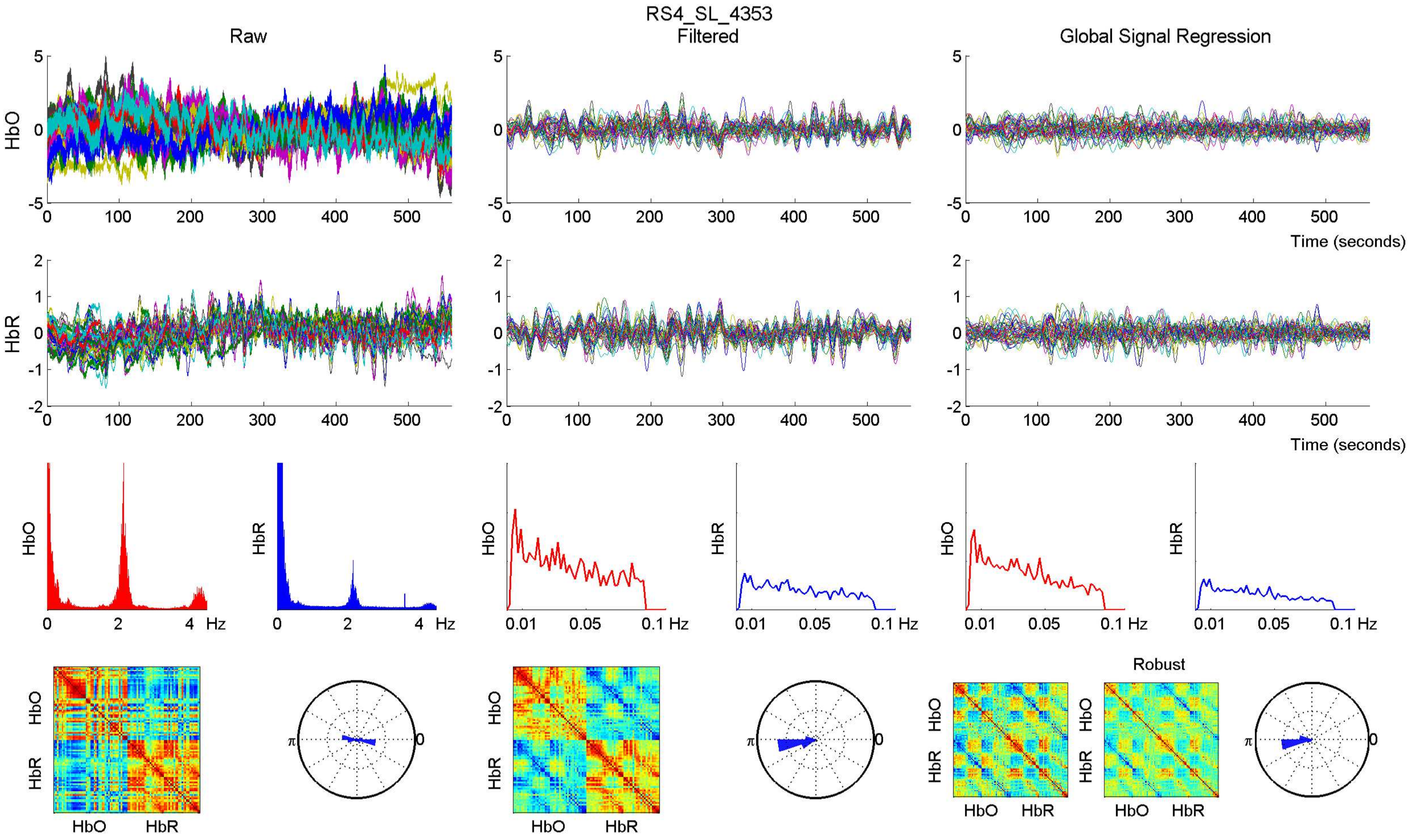

RS4_SL_4353
Raw
Filtered
Global Signal Regression
HbO
HbR
Time (seconds)
Hz
Robust

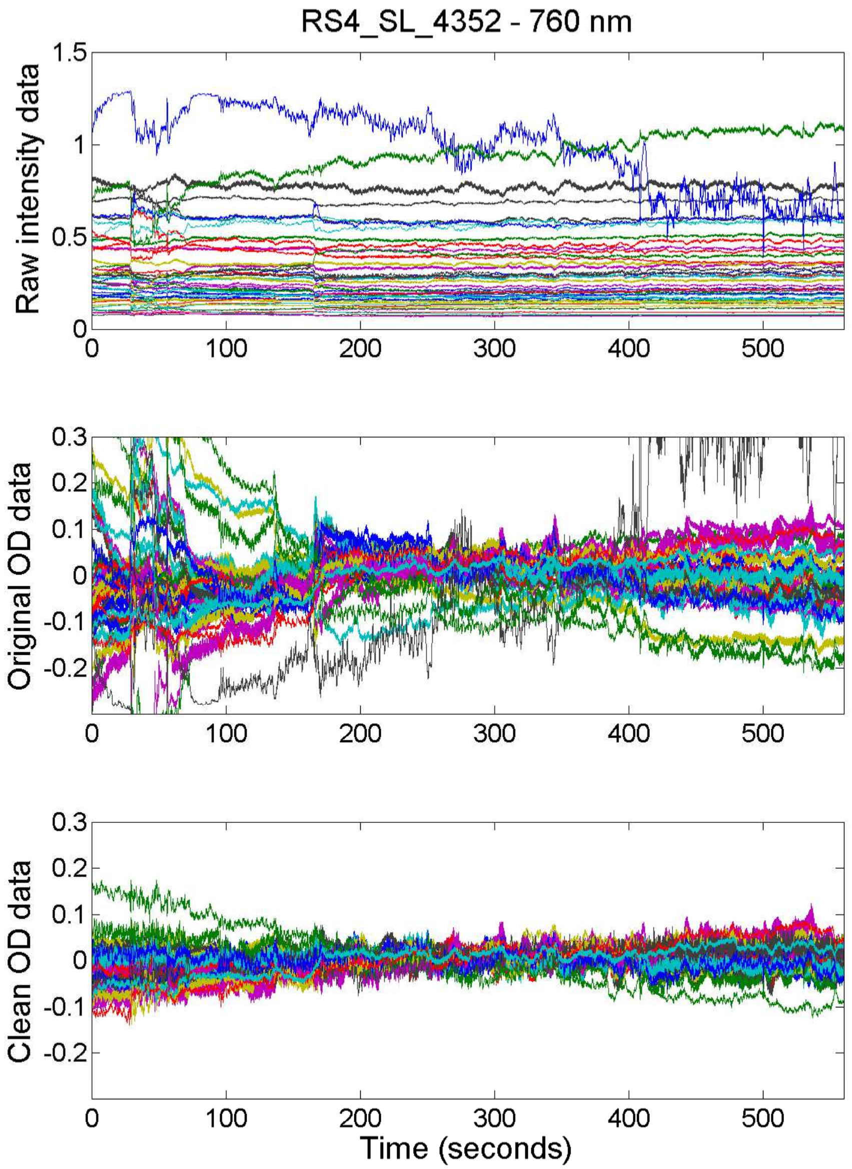
RS4_SL_4352 - 760 nm
Raw intensity data
Original OD data
Clean OD data
Time (seconds)

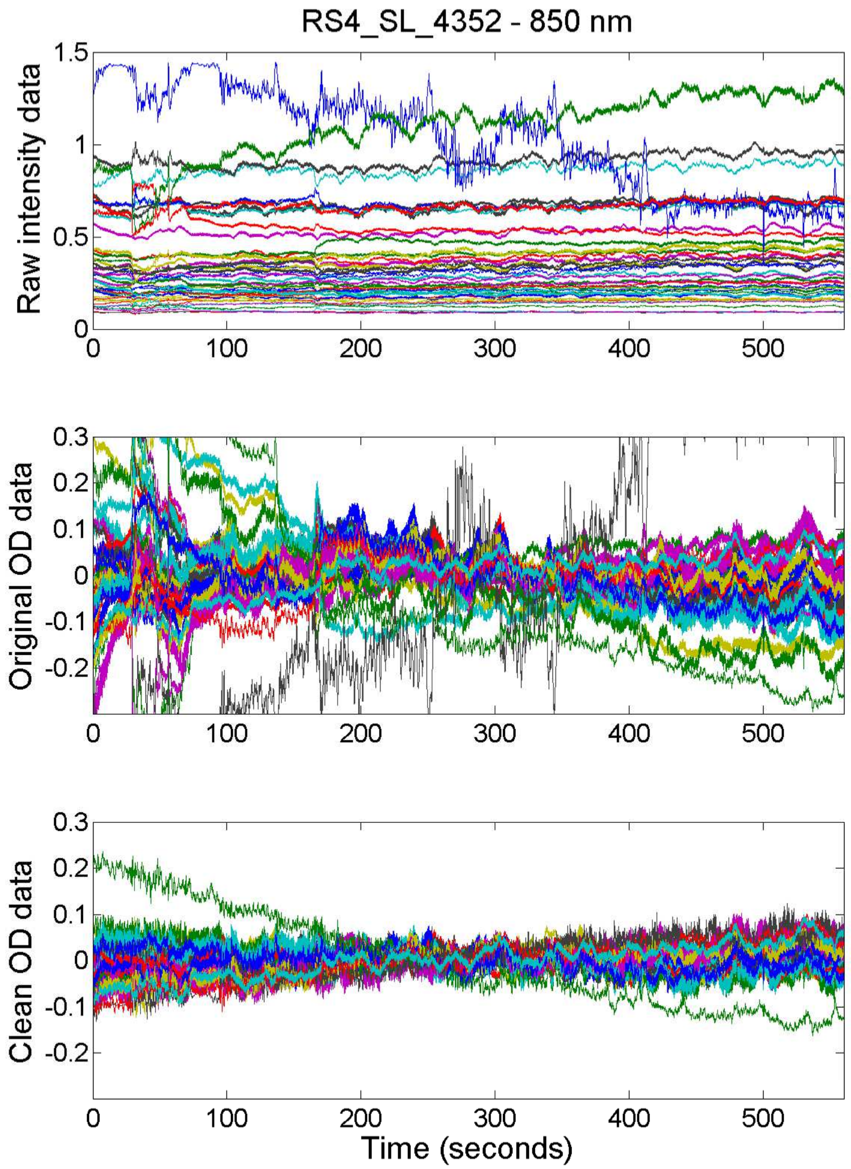
RS4_SL_4352 - 850 nm
Raw intensity data
Original OD data
Clean OD data
Time (seconds)

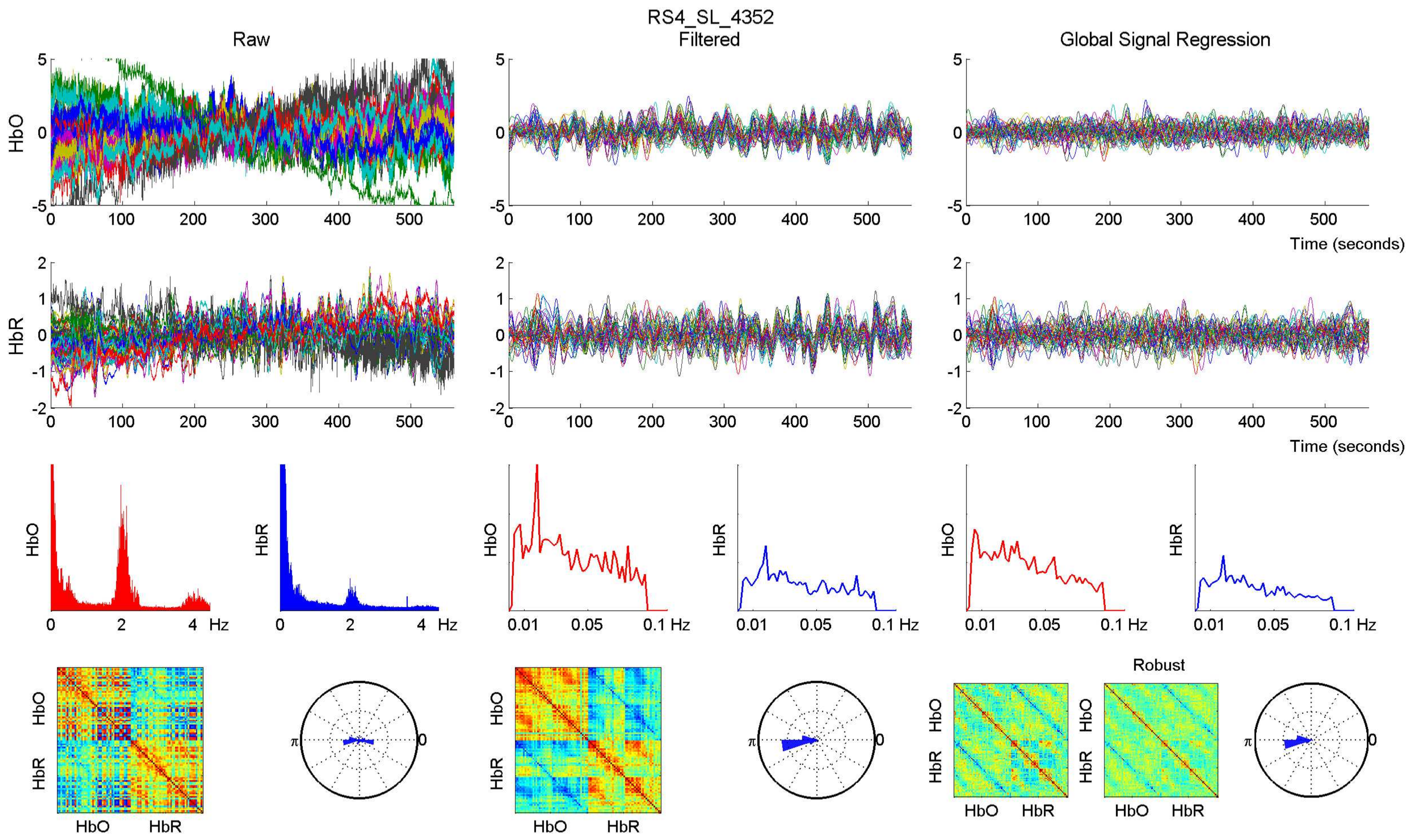

RS4_SL_4352
Raw
Filtered
Global Signal Regression
HbO
HbR
Time (seconds)
Hz
Robust

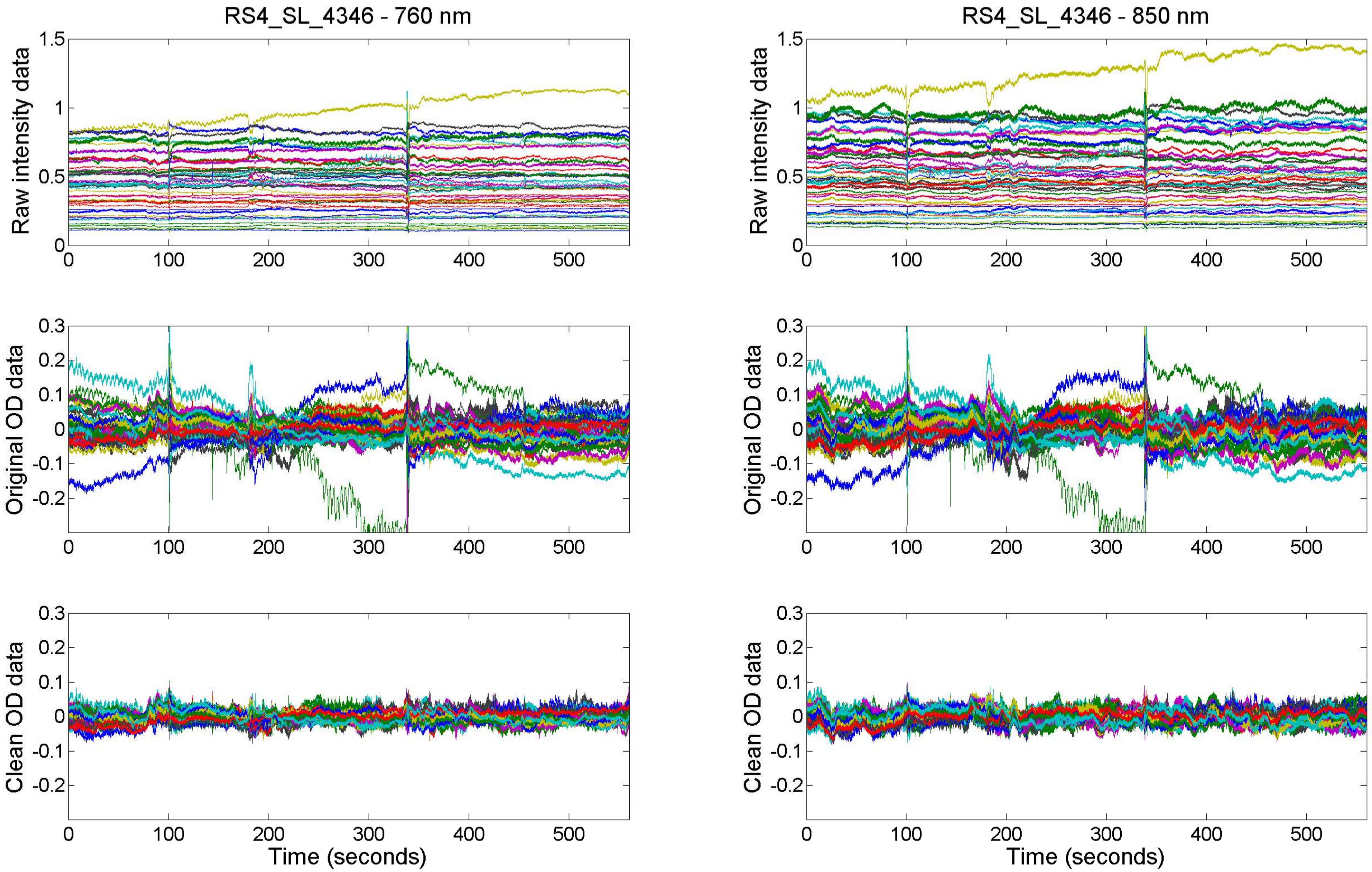

RS4_SL_4346 - 760 nm
RS4_SL_4346 - 850 nm
Raw intensity data
Original OD data
Clean OD data
Time (seconds)

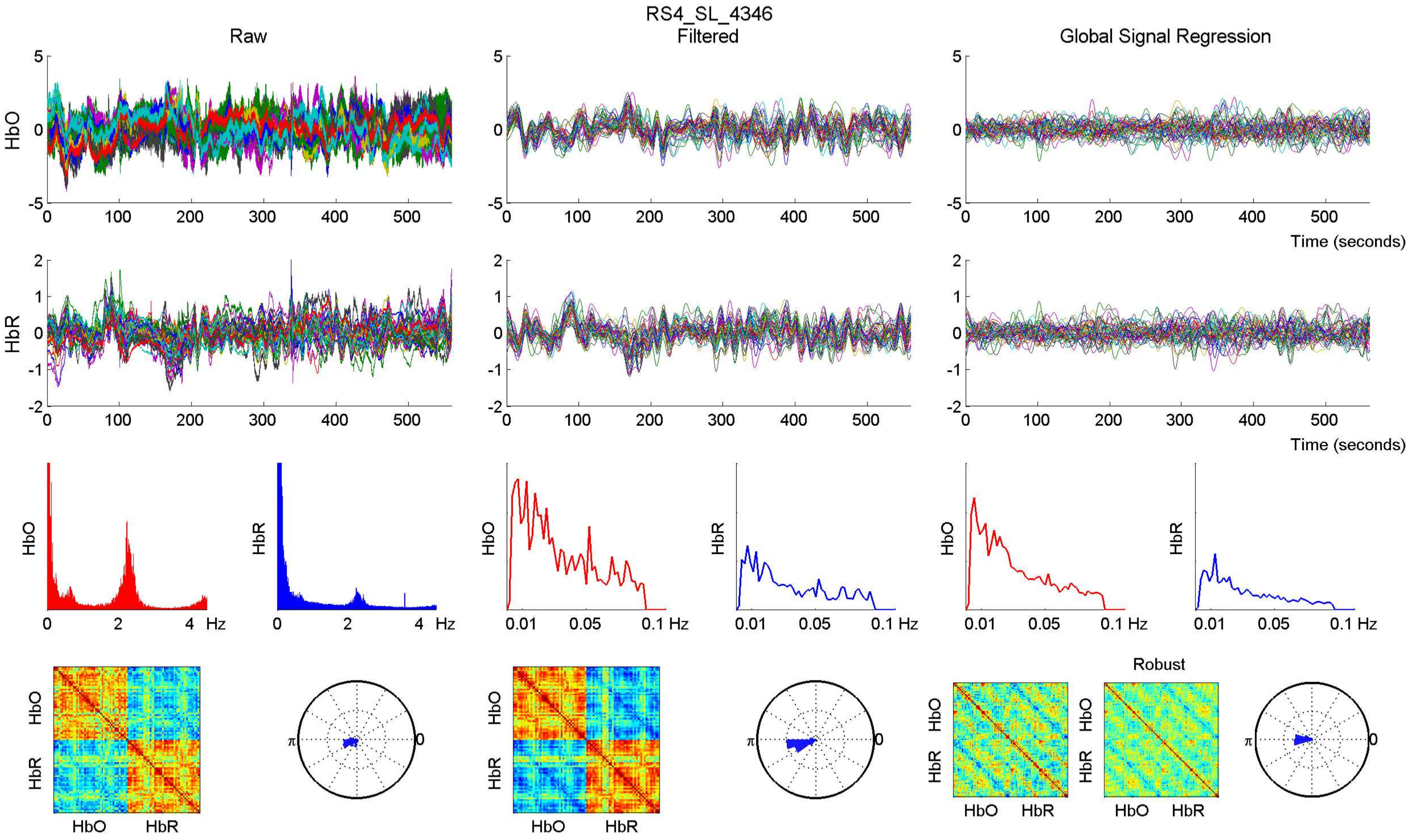

RS4_SL_4346
Raw
Filtered
Global Signal Regression
HbO
HbR
Time (seconds)
Hz
Robust

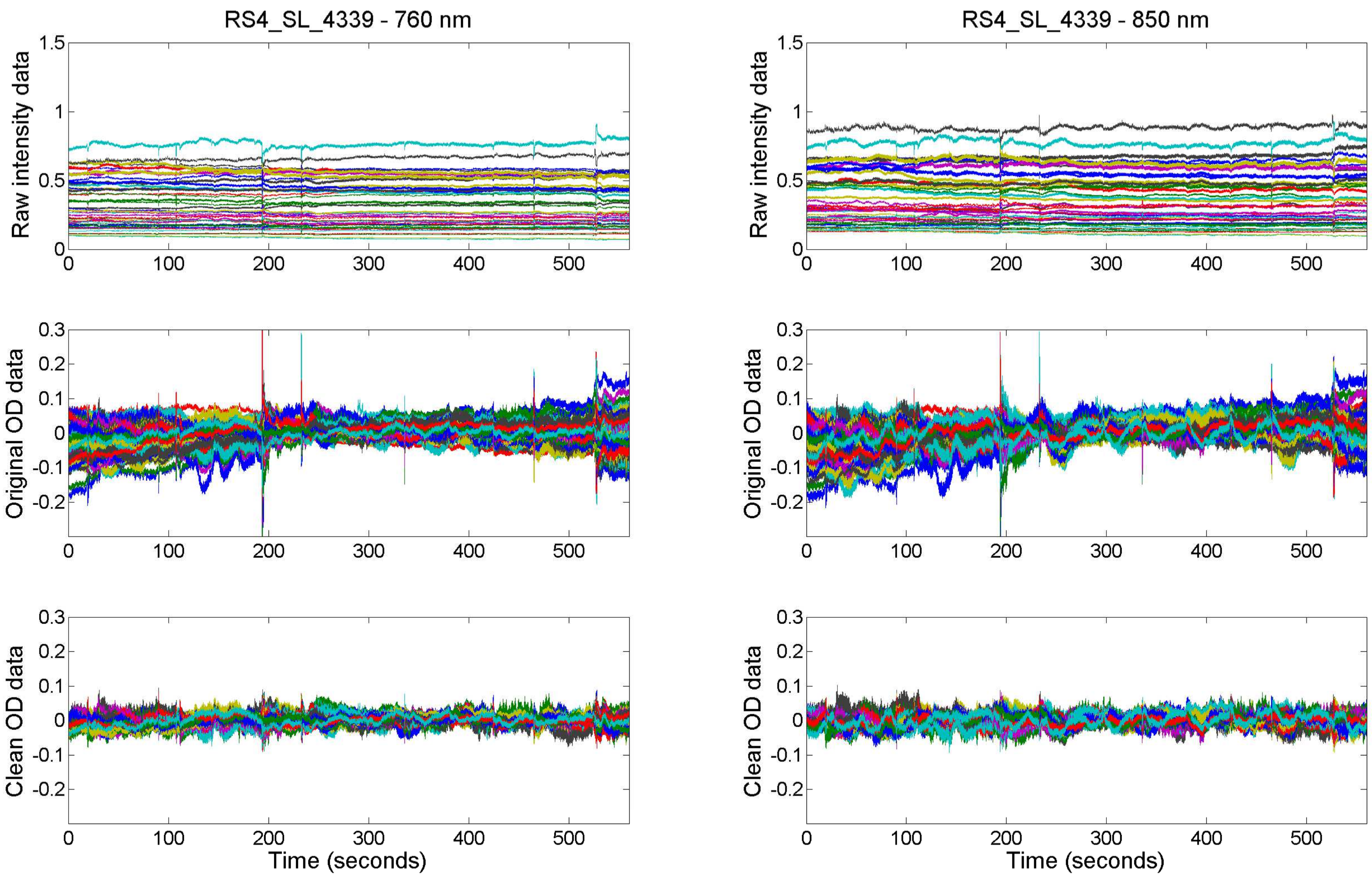
RS4_SL_4339 - 760 nm
RS4_SL_4339 - 850 nm
Raw intensity data
Original OD data
Clean OD data
Time (seconds)
0
100
200
300
400
500
1.5
1
0.5
0.3
0.2
0.1
-0.1
-0.2

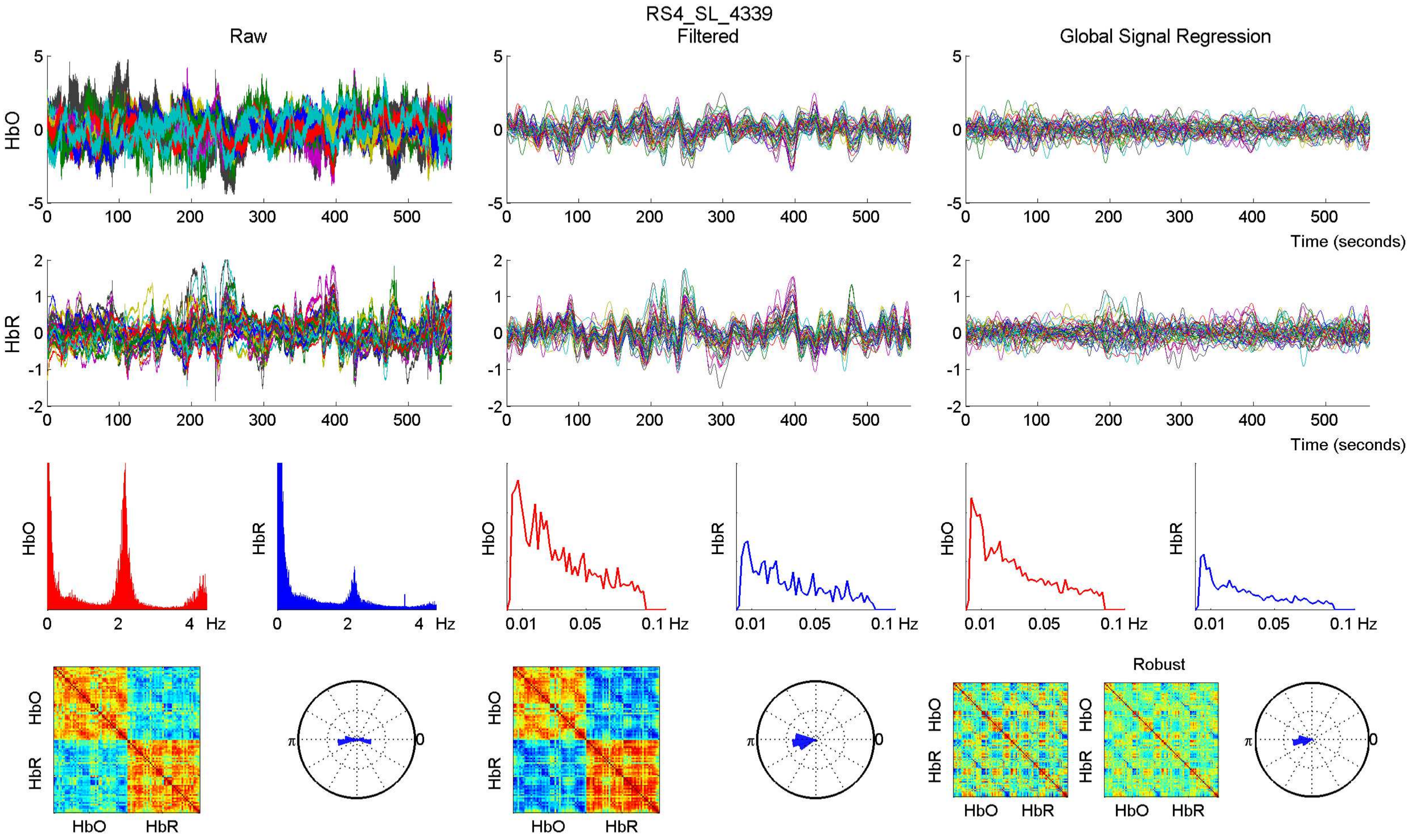
RS4_SL_4339
Raw
Filtered
Global Signal Regression
HbO
HbR
Time (seconds)
Hz
Robust
HbO
HbR

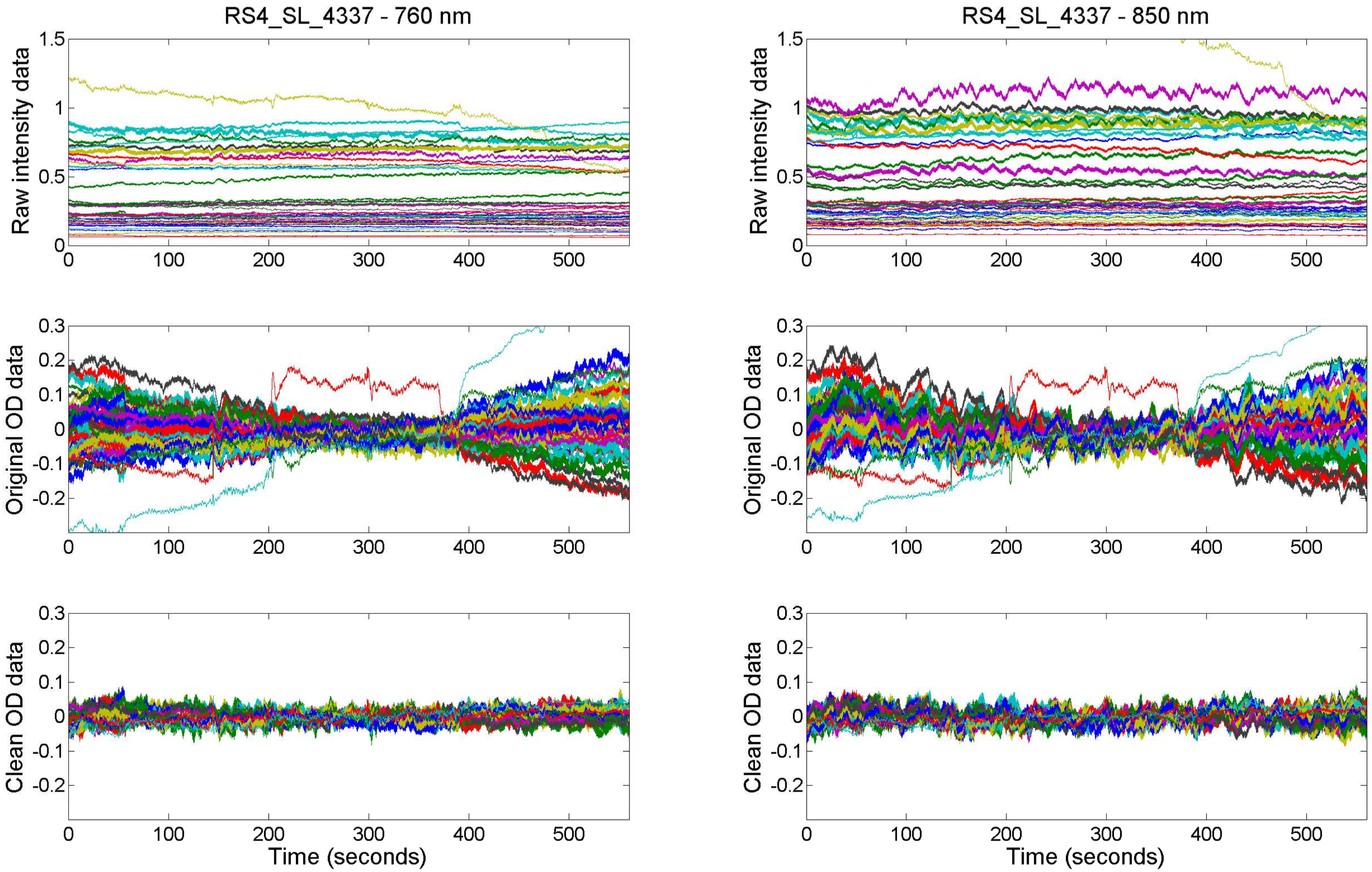

RS4_SL_4337 - 760 nm
RS4_SL_4337 - 850 nm
Raw intensity data
Original OD data
Clean OD data
Time (seconds)
0
100
200
300
400
500
1.5
1
0.5
0.3
0.2
0.1
-0.1
-0.2

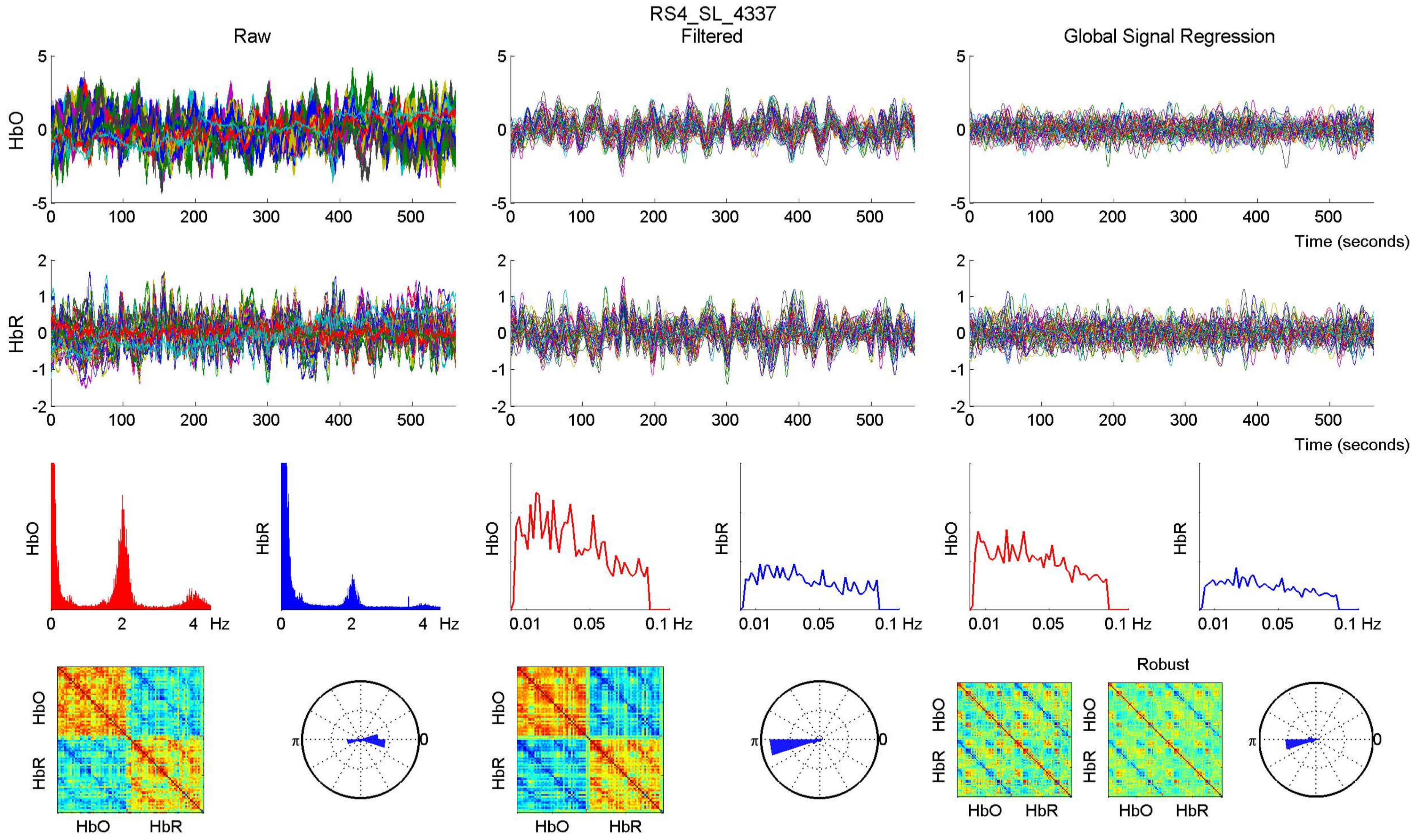
RS4_SL_4337
Raw
Filtered
Global Signal Regression
HbO
HbR
Time (seconds)
Hz
Robust
HbO
HbR

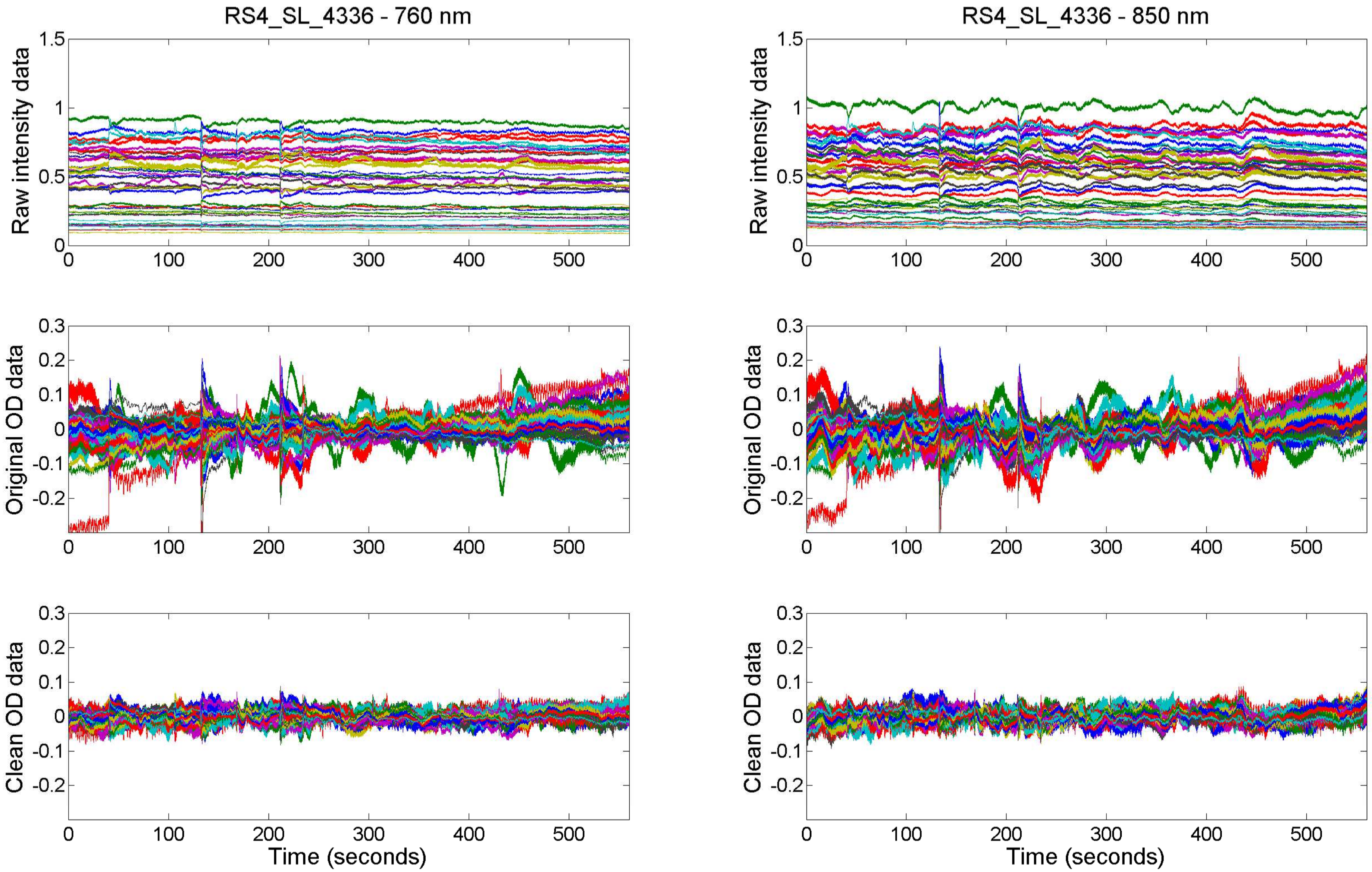
RS4_SL_4336 - 760 nm
RS4_SL_4336 - 850 nm
Raw intensity data
Original OD data
Clean OD data
Time (seconds)
1.5
1
0.5
0
0.3
0.2
0.1
-0.1
-0.2
100
200
300
400
500

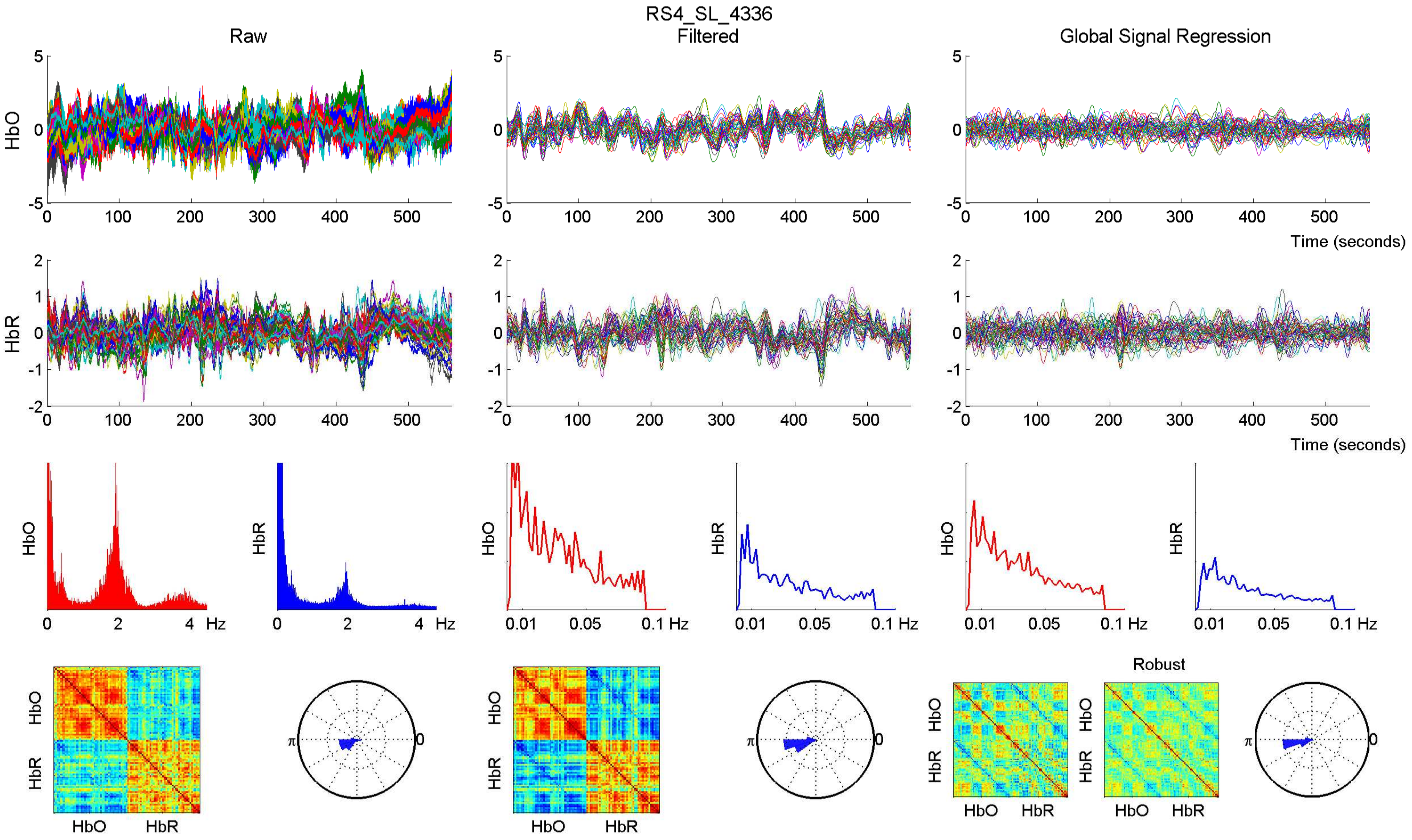

RS4_SL_4336
Raw
Filtered
Global Signal Regression
HbO
HbR
Time (seconds)
Hz
0.01
0.05
0.1 Hz
Robust
HbO
HbR
π
0

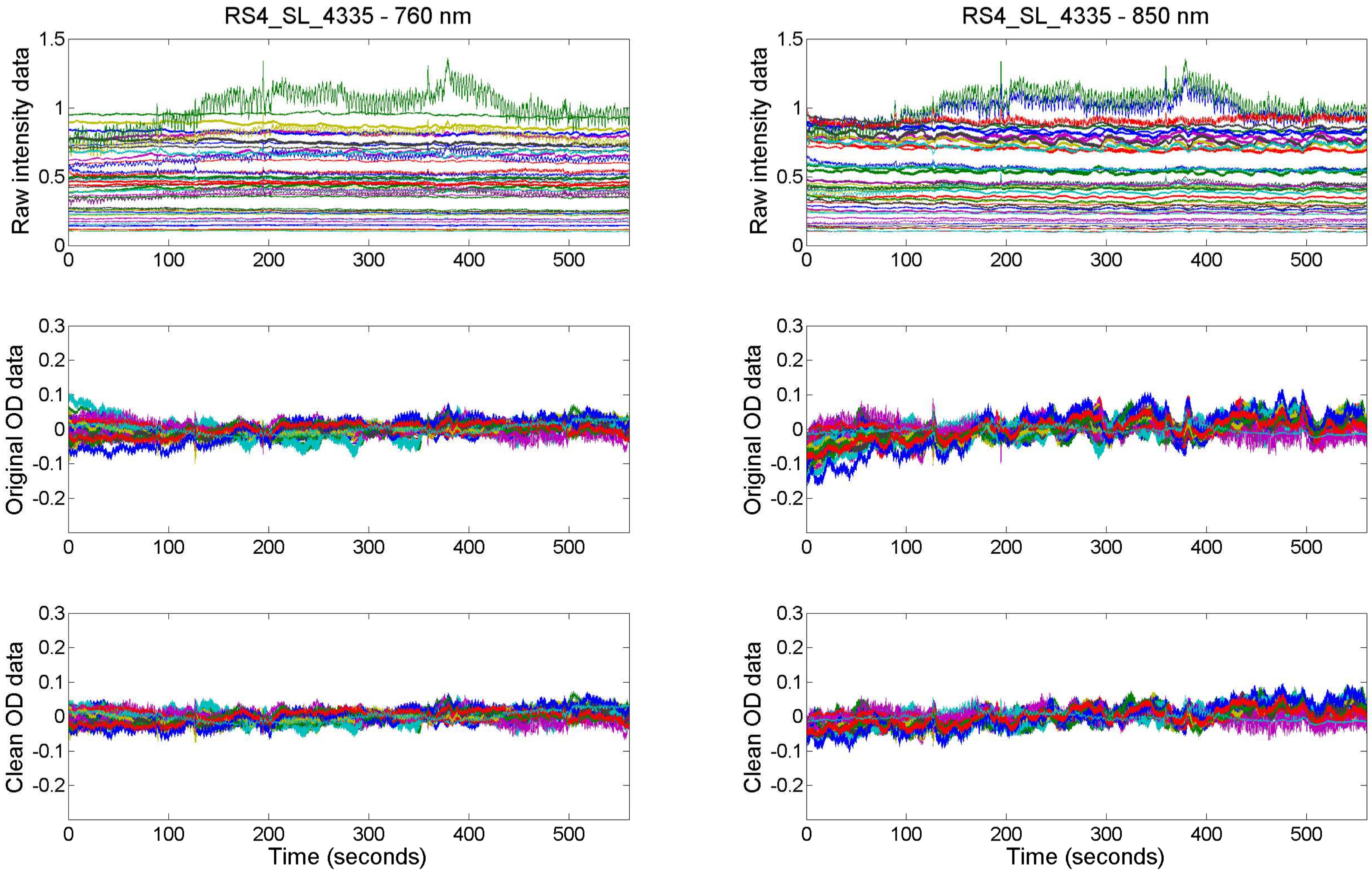

RS4_SL_4335 - 760 nm
RS4_SL_4335 - 850 nm
Raw intensity data
Original OD data
Clean OD data
Time (seconds)

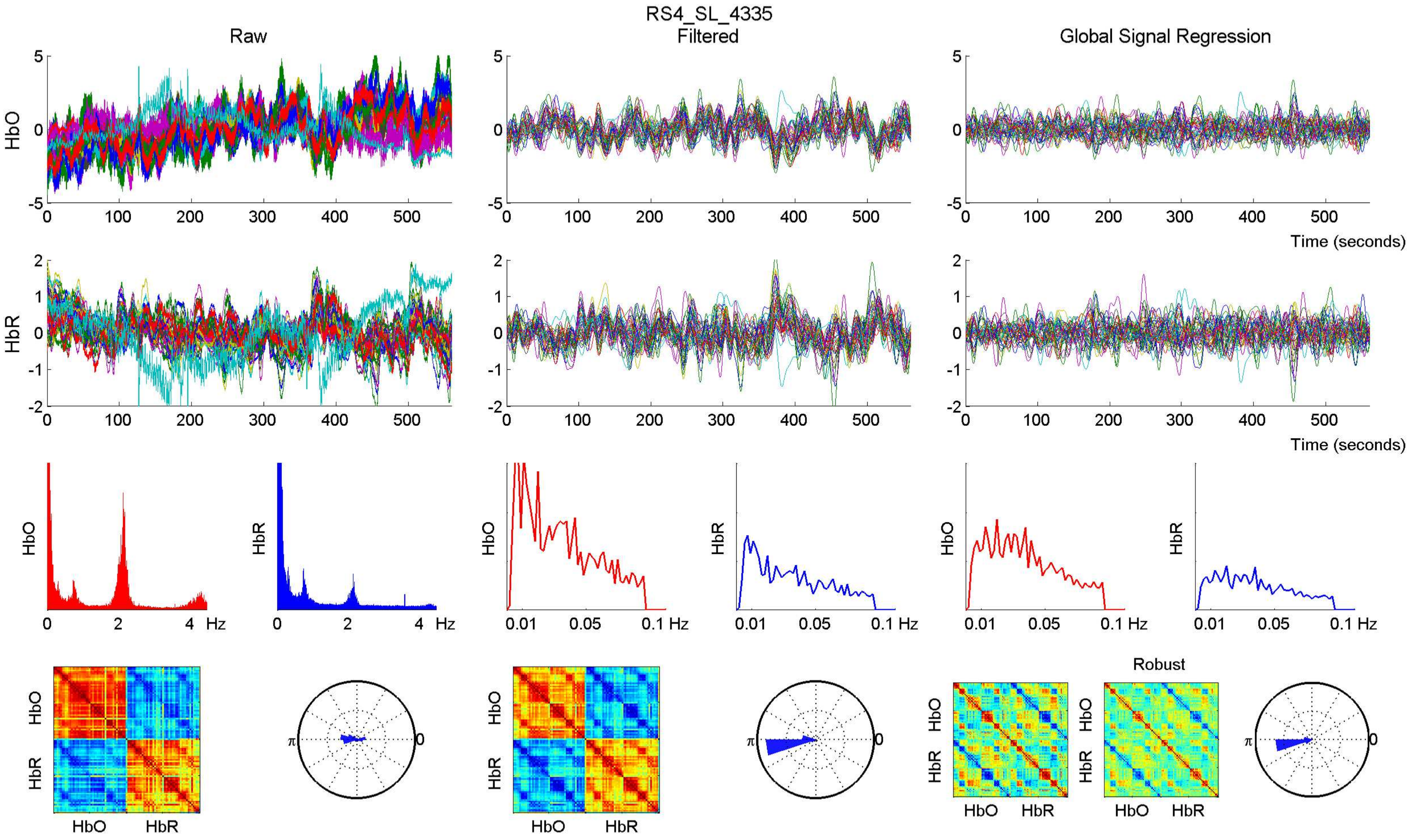

RS4_SL_4335
Raw
Filtered
Global Signal Regression
HbO
HbR
Time (seconds)
Hz
Robust

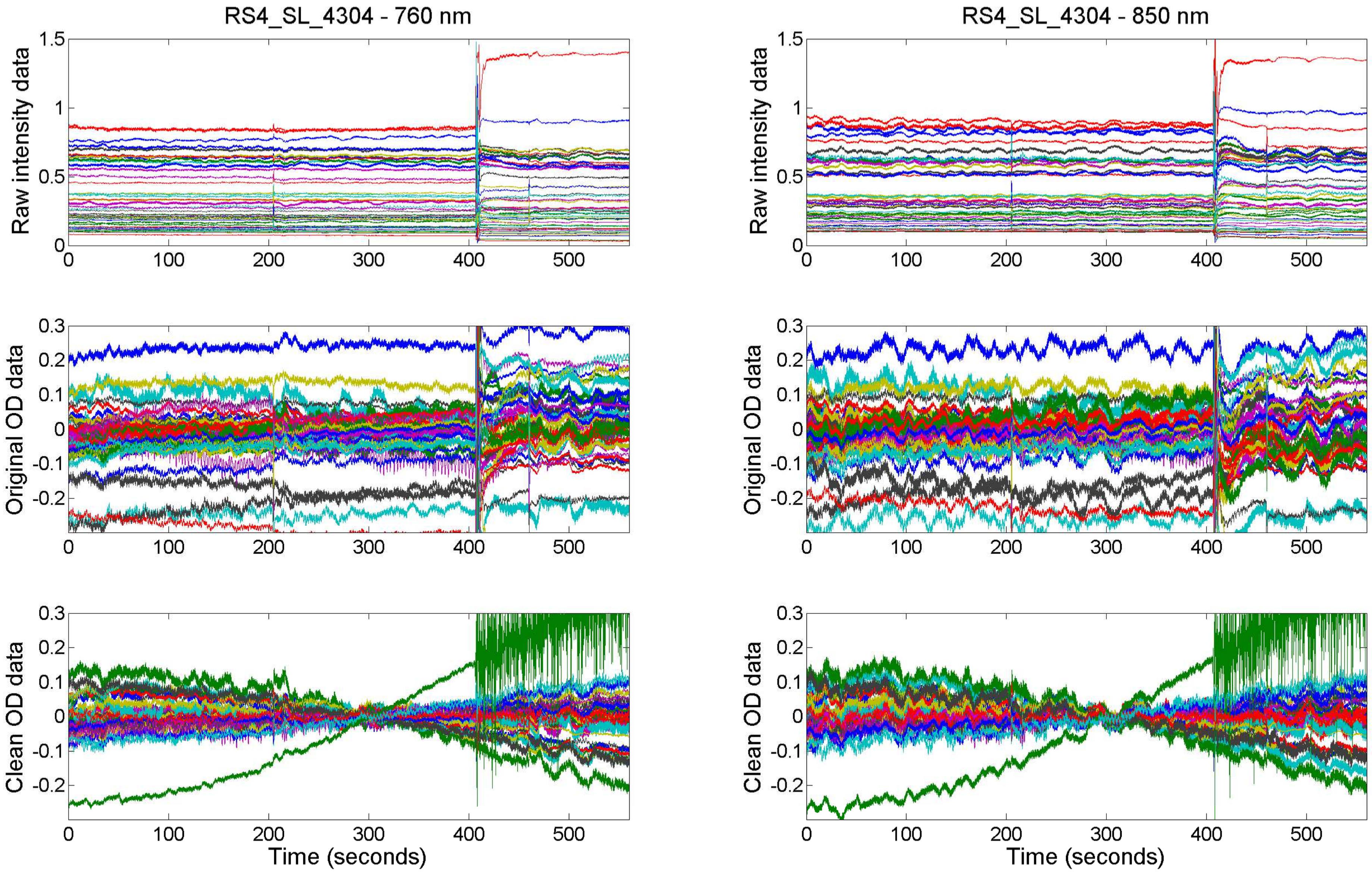
RS4_SL_4304 - 760 nm
RS4_SL_4304 - 850 nm
Raw intensity data
Original OD data
Clean OD data
Time (seconds)
1.5
1
0.5
0
0.3
0.2
0.1
-0.1
-0.2
100
200
300
400
500

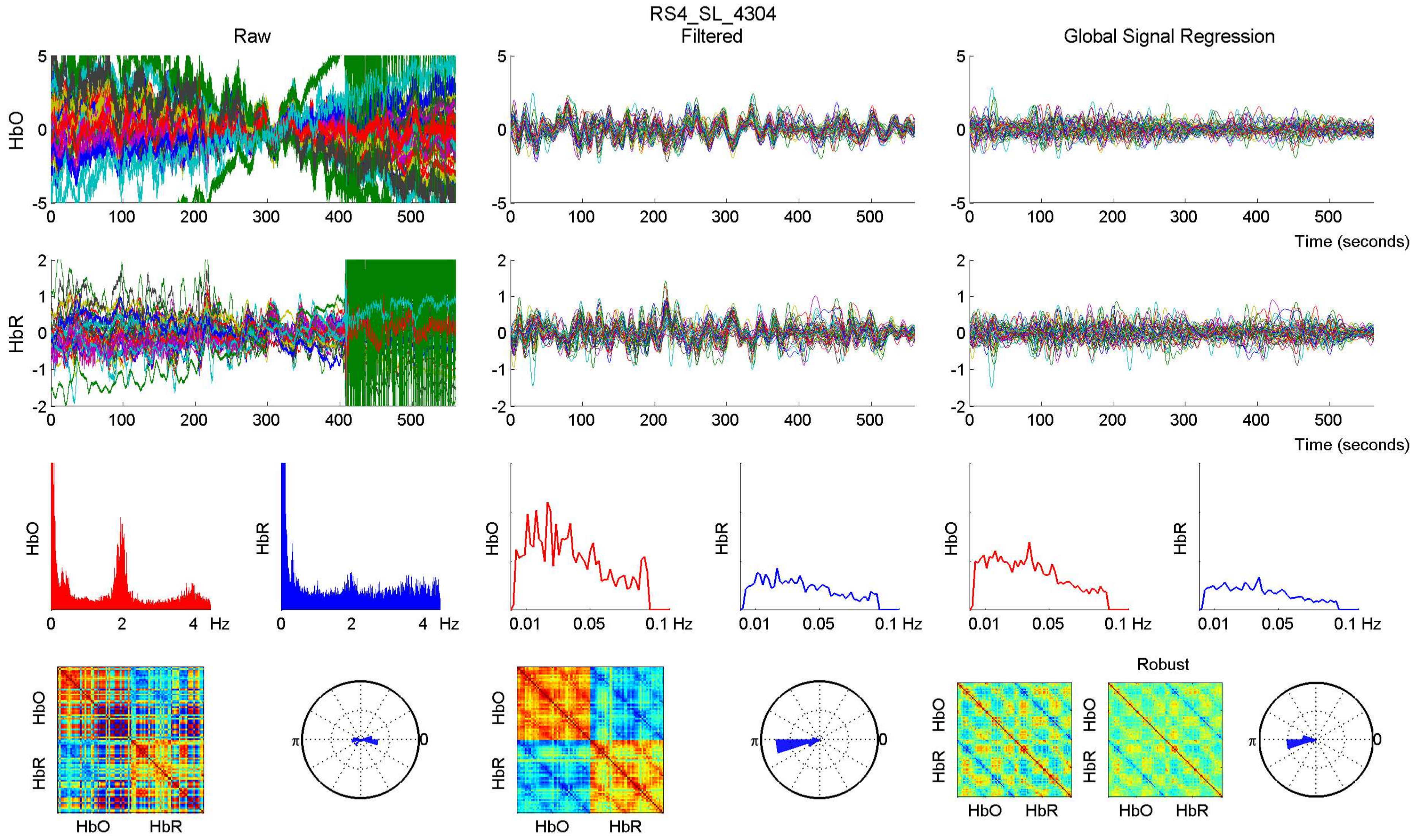

RS4_SL_4304
Raw
Filtered
Global Signal Regression
HbO
HbR
Time (seconds)
Hz
Robust

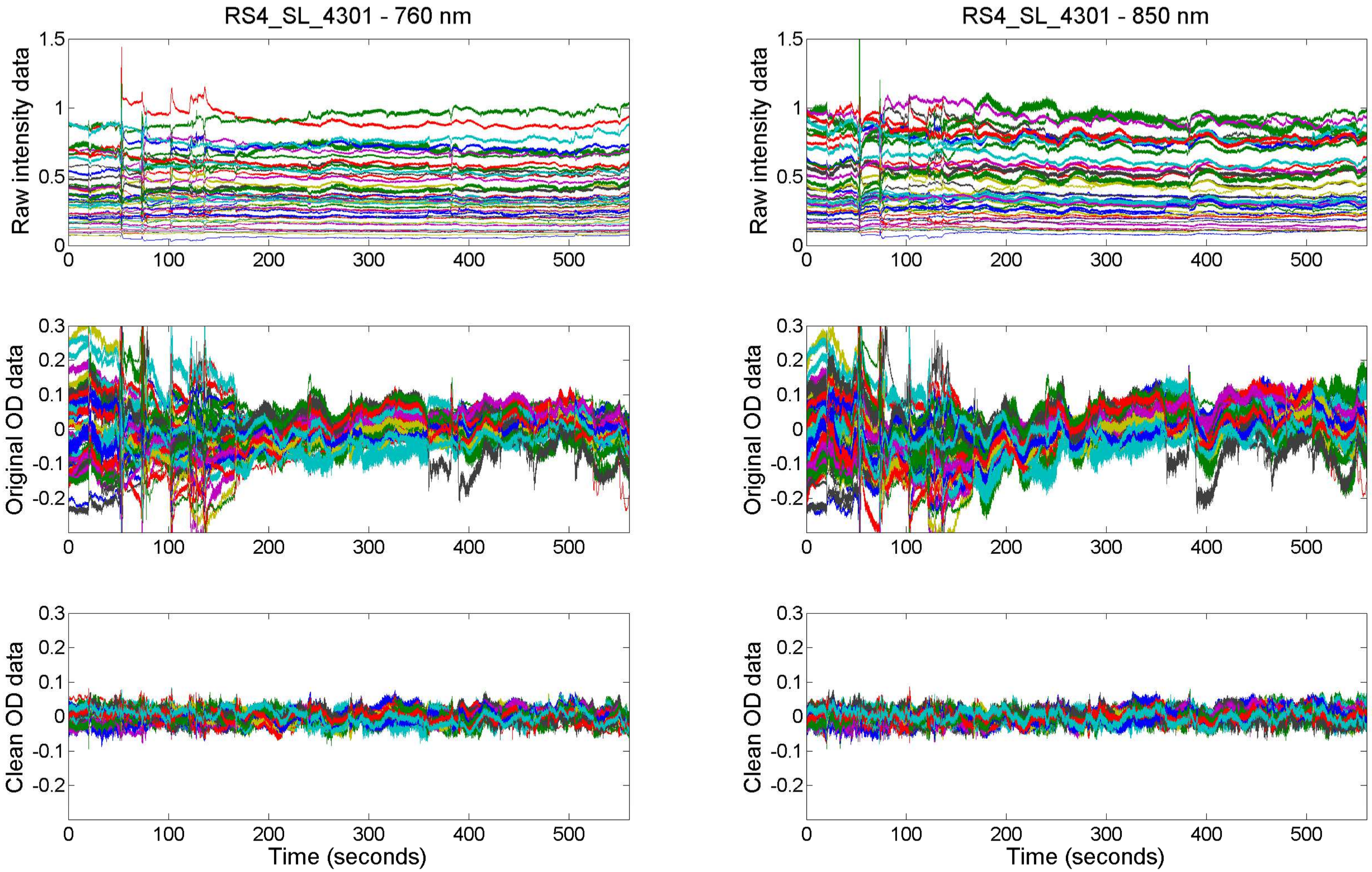

RS4_SL_4301 - 760 nm
RS4_SL_4301 - 850 nm
Raw intensity data
Original OD data
Clean OD data
Time (seconds)

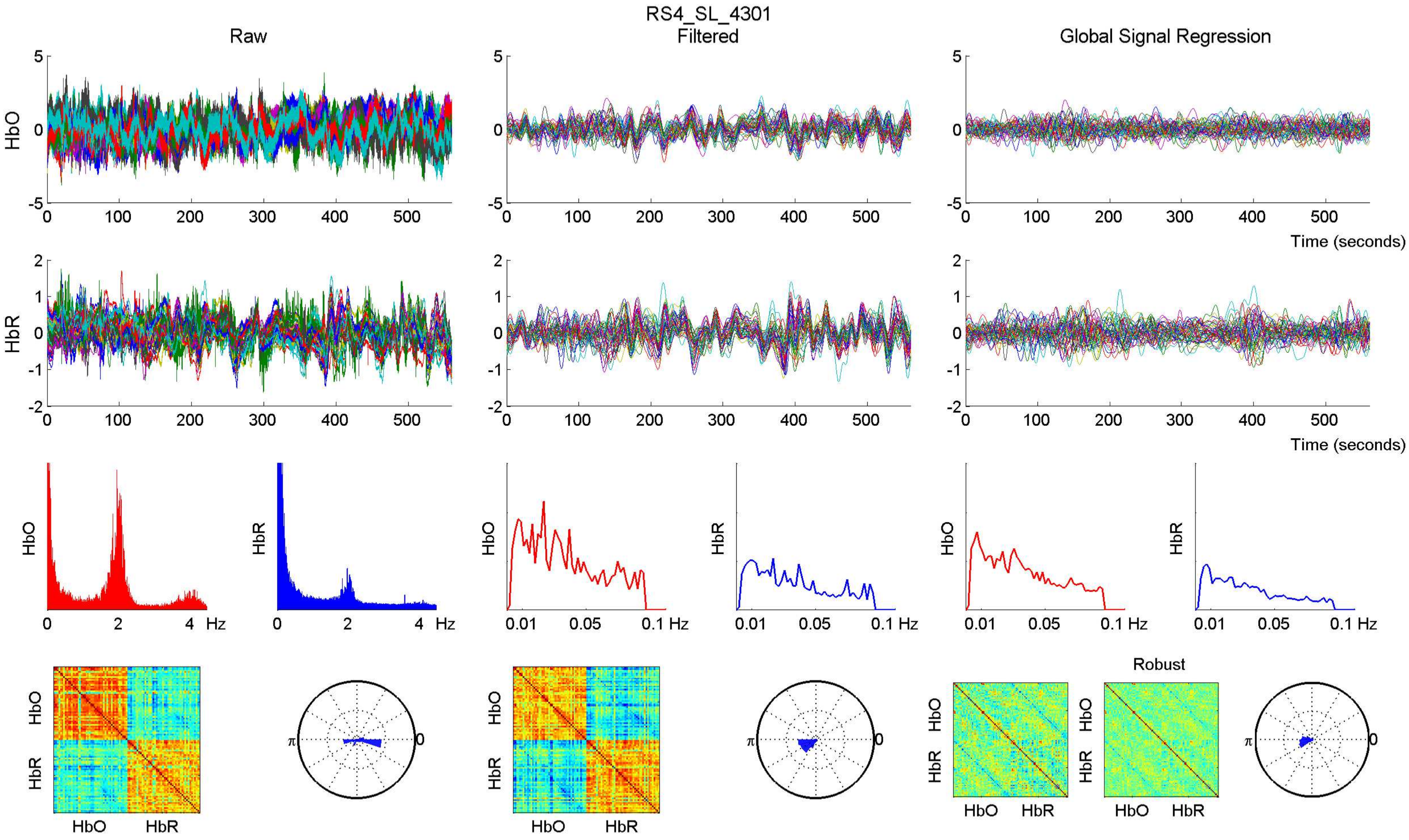

RS4_SL_4301
Raw
Filtered
Global Signal Regression
HbO
HbR
Time (seconds)
Hz
Robust
HbO
HbR

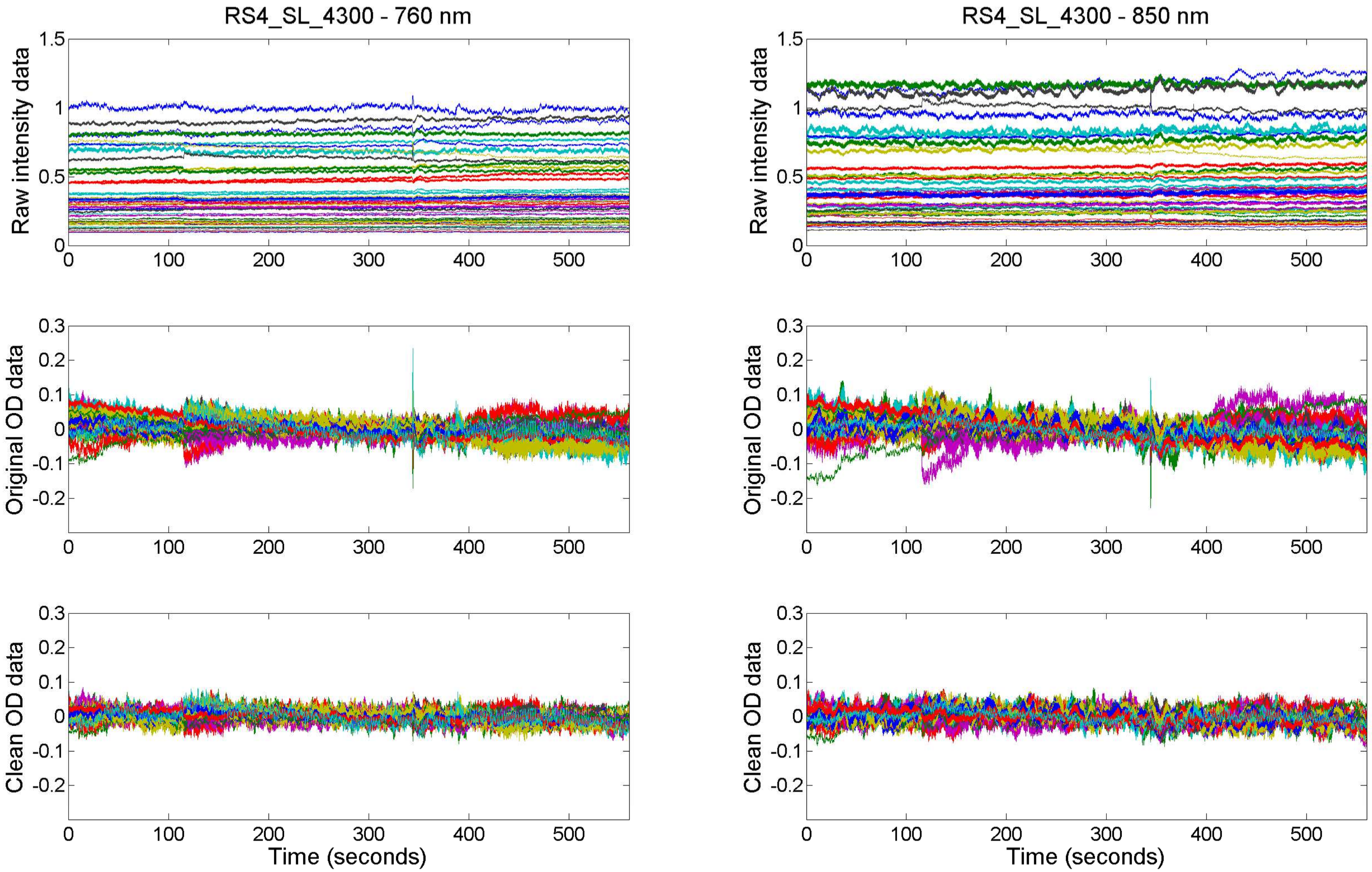
RS4_SL_4300 - 760 nm
RS4_SL_4300 - 850 nm
Raw intensity data
Original OD data
Clean OD data
Time (seconds)

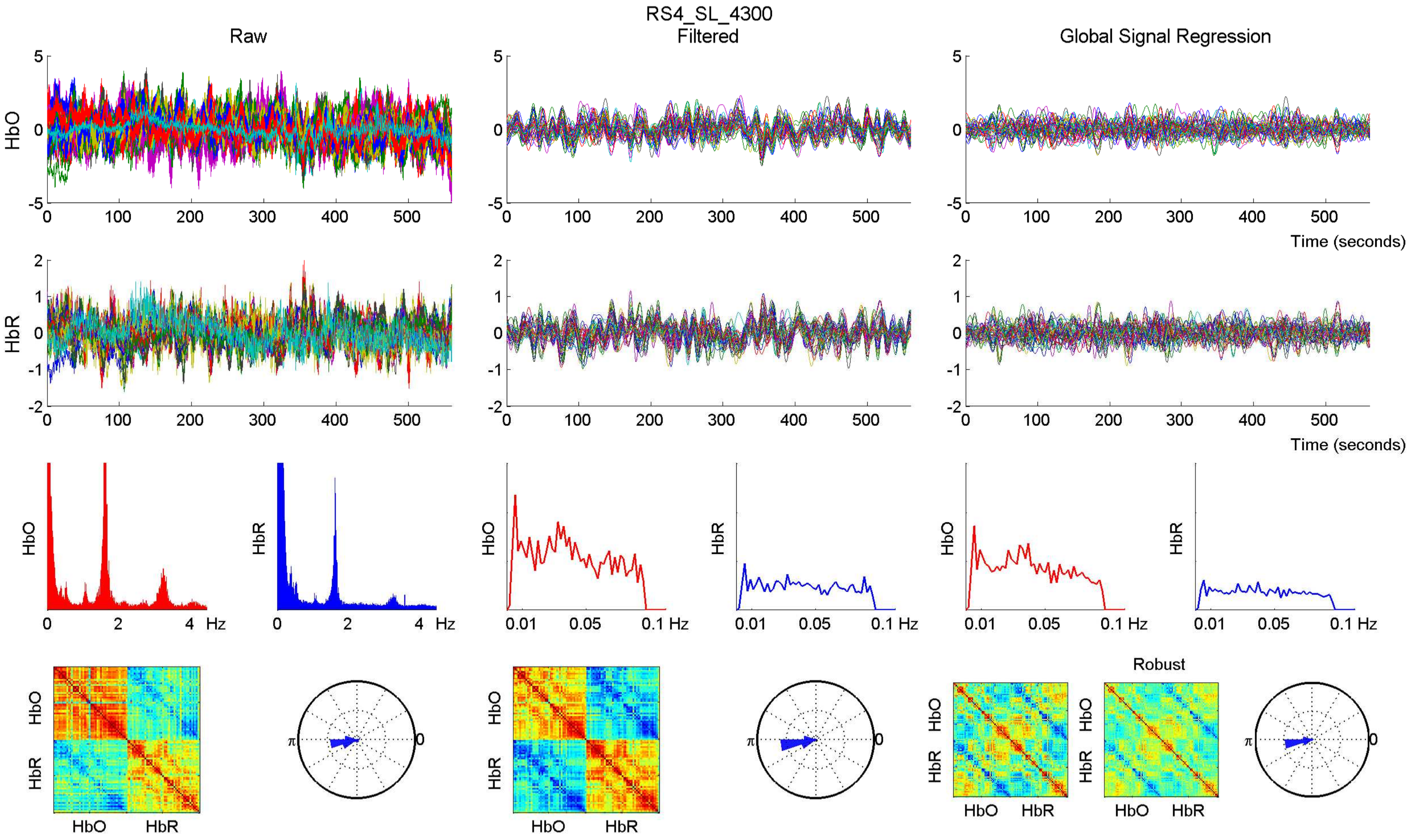

RS4_SL_4300
Raw
Filtered
Global Signal Regression
HbO
HbR
Time (seconds)
Hz
0.01
0.05
0.1 Hz
Robust
HbO
HbR
π
0

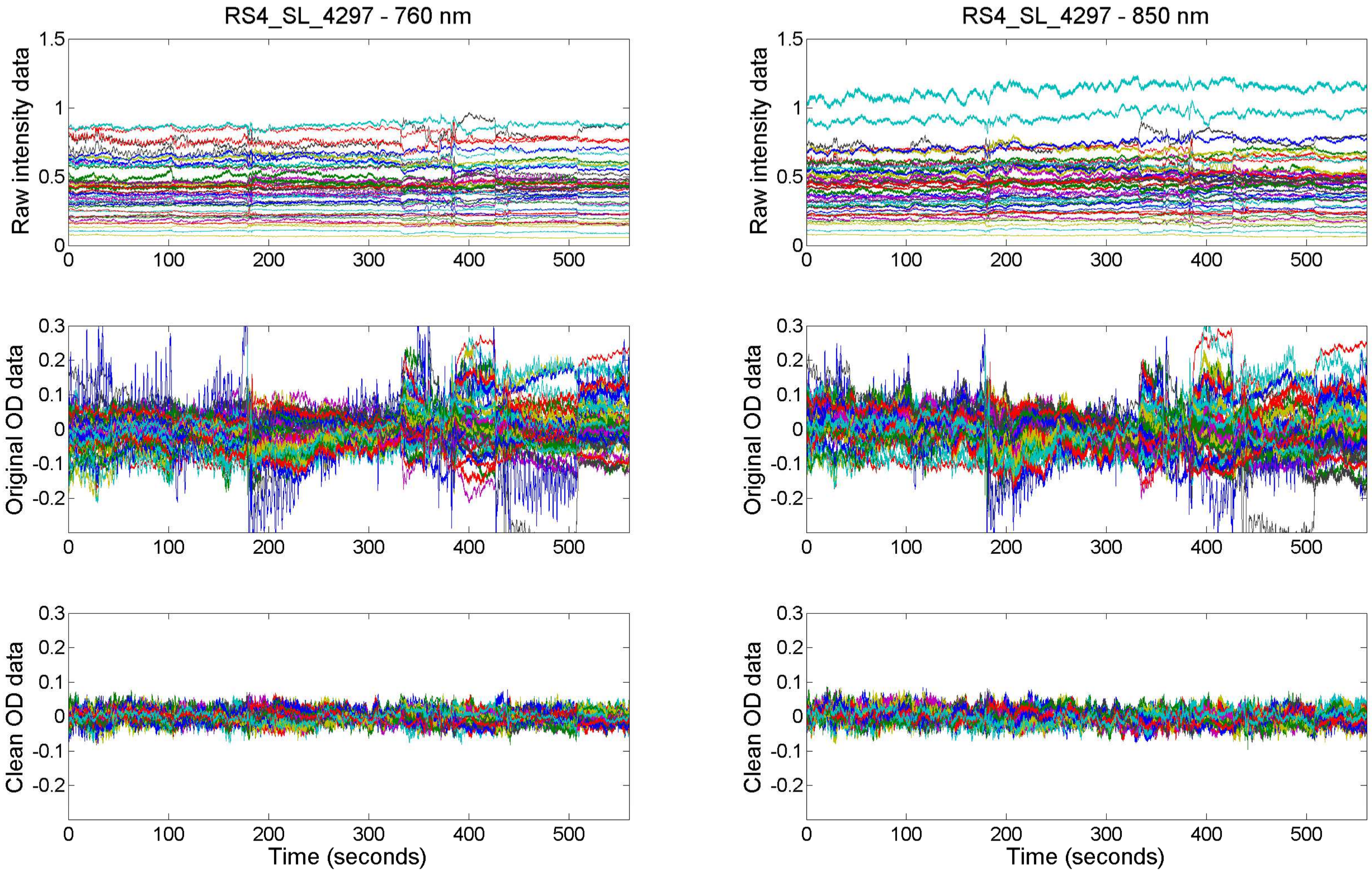
RS4_SL_4297 - 760 nm
RS4_SL_4297 - 850 nm
Raw intensity data
Original OD data
Clean OD data
Time (seconds)

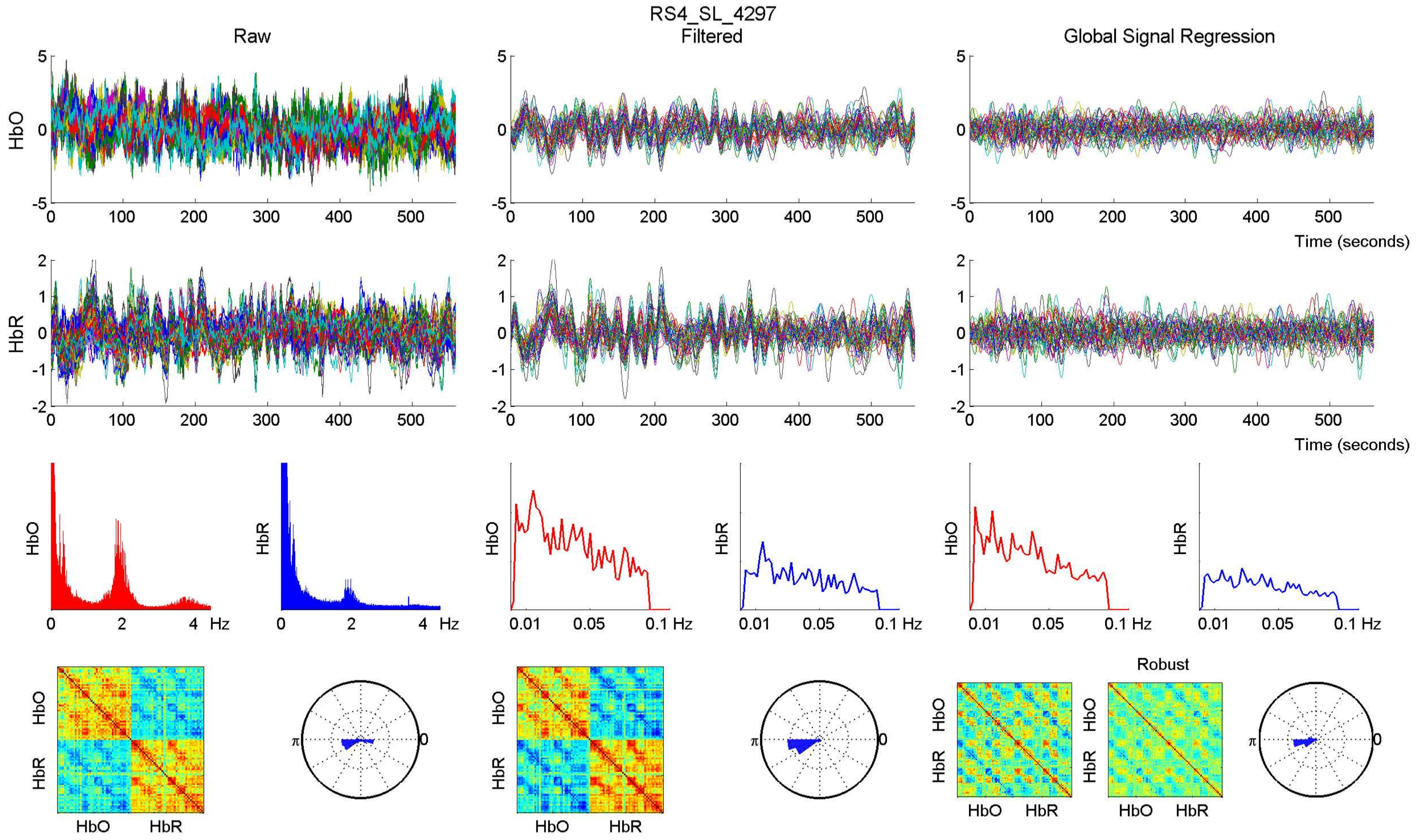
RS4_SL_4297
Raw
Filtered
Global Signal Regression
HbO
HbR
Time (seconds)
Hz
Robust
HbO
HbR

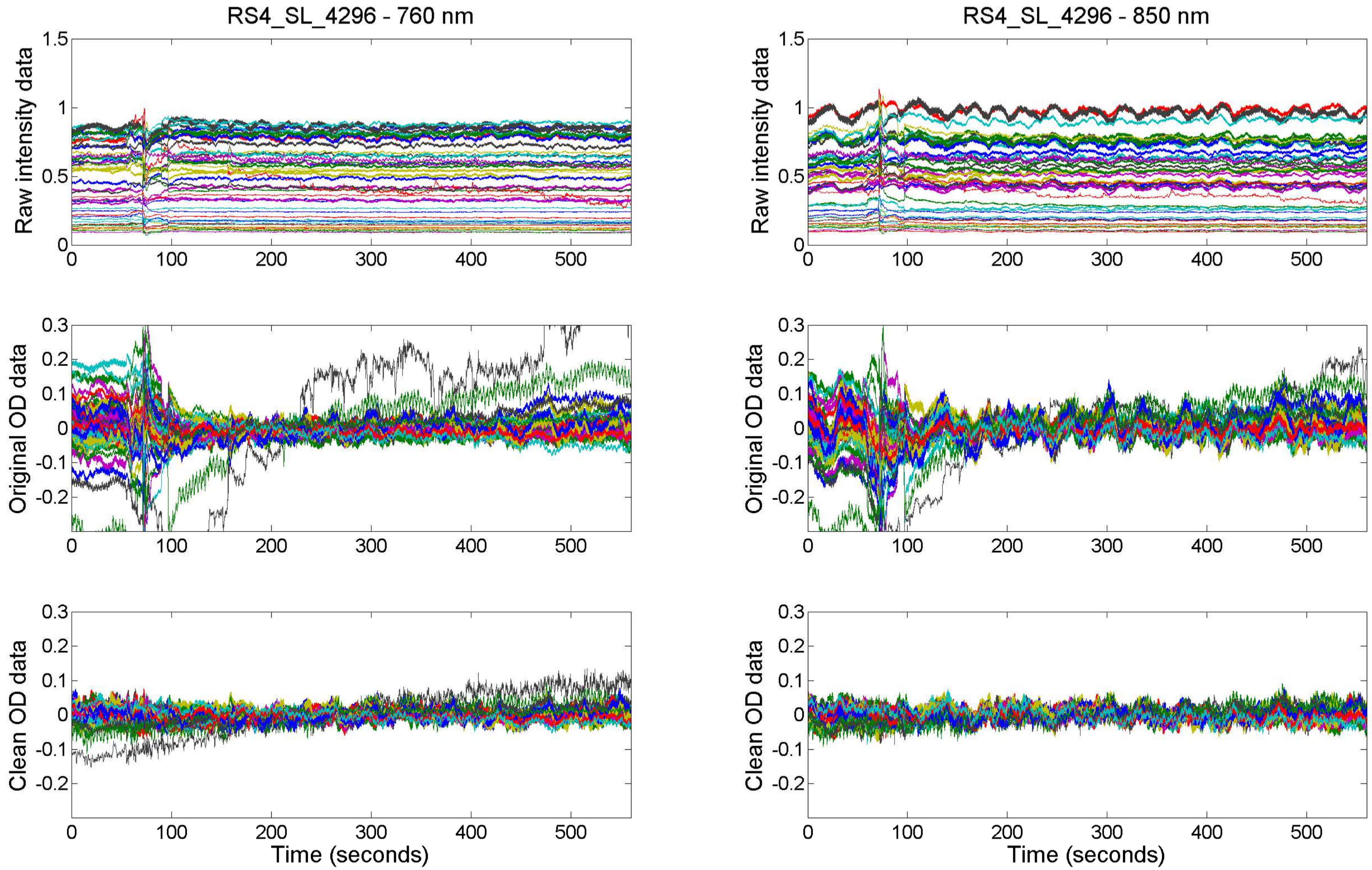
RS4_SL_4296 - 760 nm
RS4_SL_4296 - 850 nm
Raw intensity data
Original OD data
Clean OD data
Time (seconds)
Time (seconds)

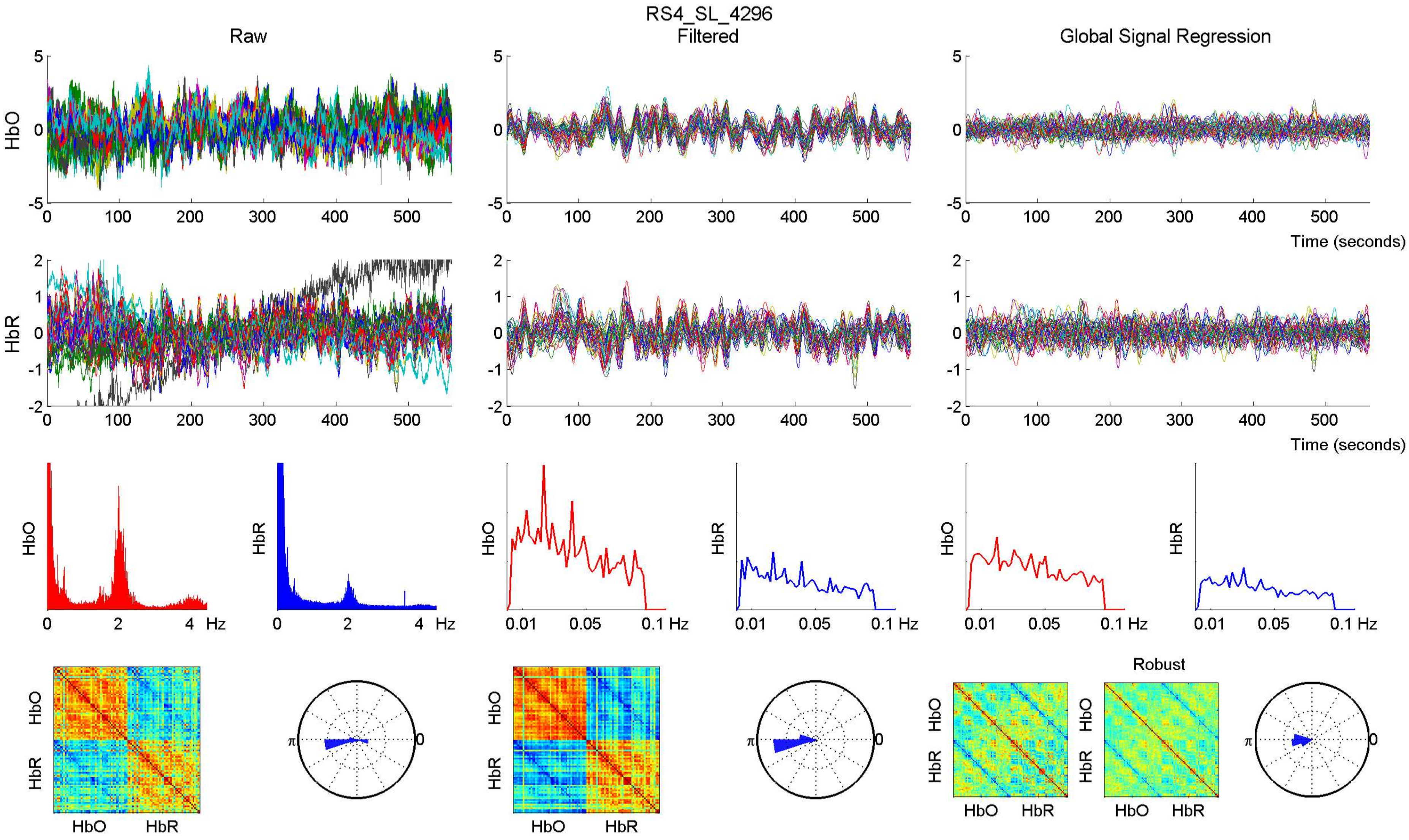
RS4_SL_4296
Raw
Filtered
Global Signal Regression
HbO
HbR
Time (seconds)
Hz
Robust
HbO
HbR

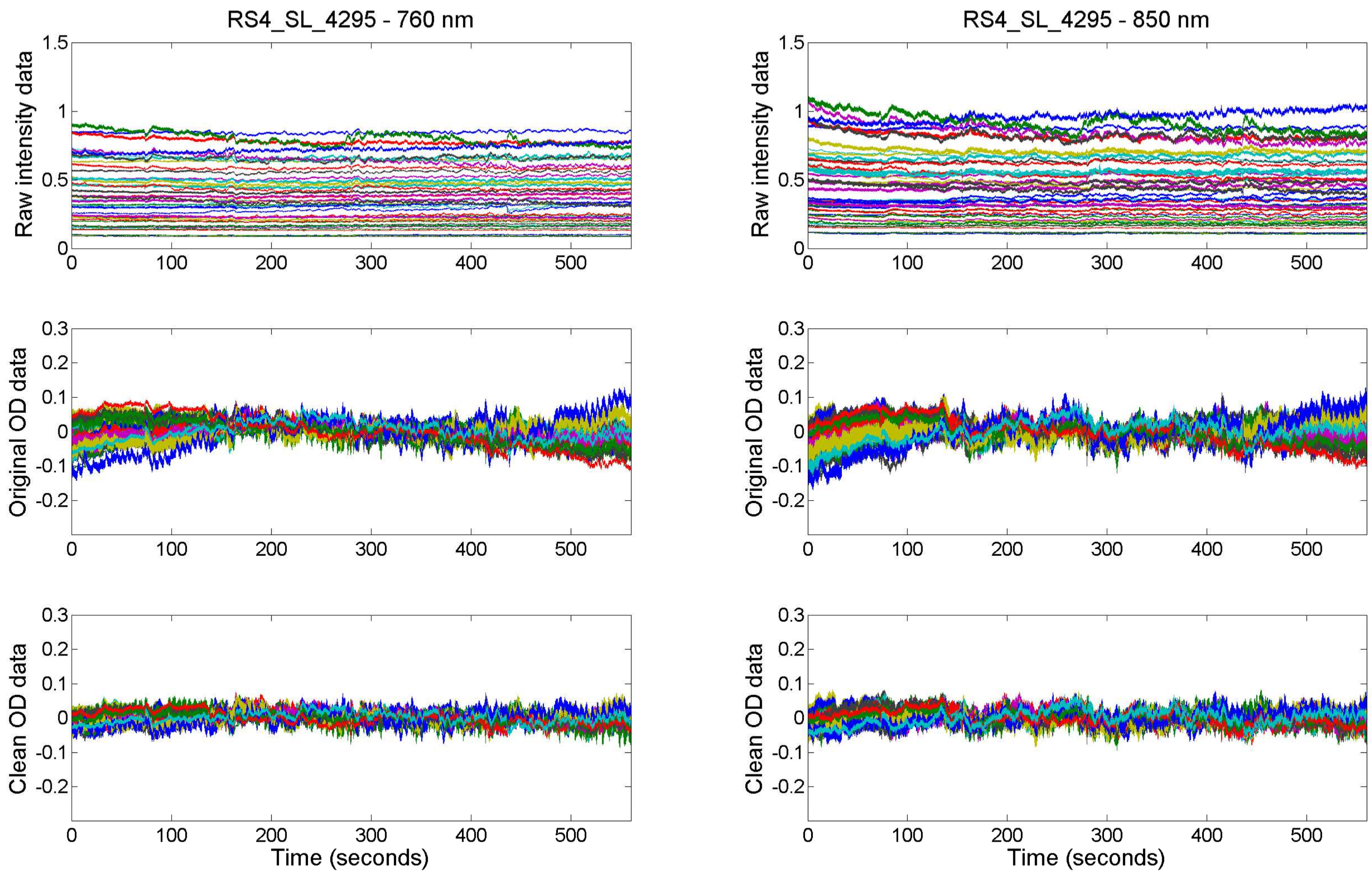
RS4_SL_4295 - 760 nm
RS4_SL_4295 - 850 nm
Raw intensity data
Original OD data
Clean OD data
Time (seconds)
1.5
1
0.5
0
0.3
0.2
0.1
-0.1
-0.2
100
200
300
400
500

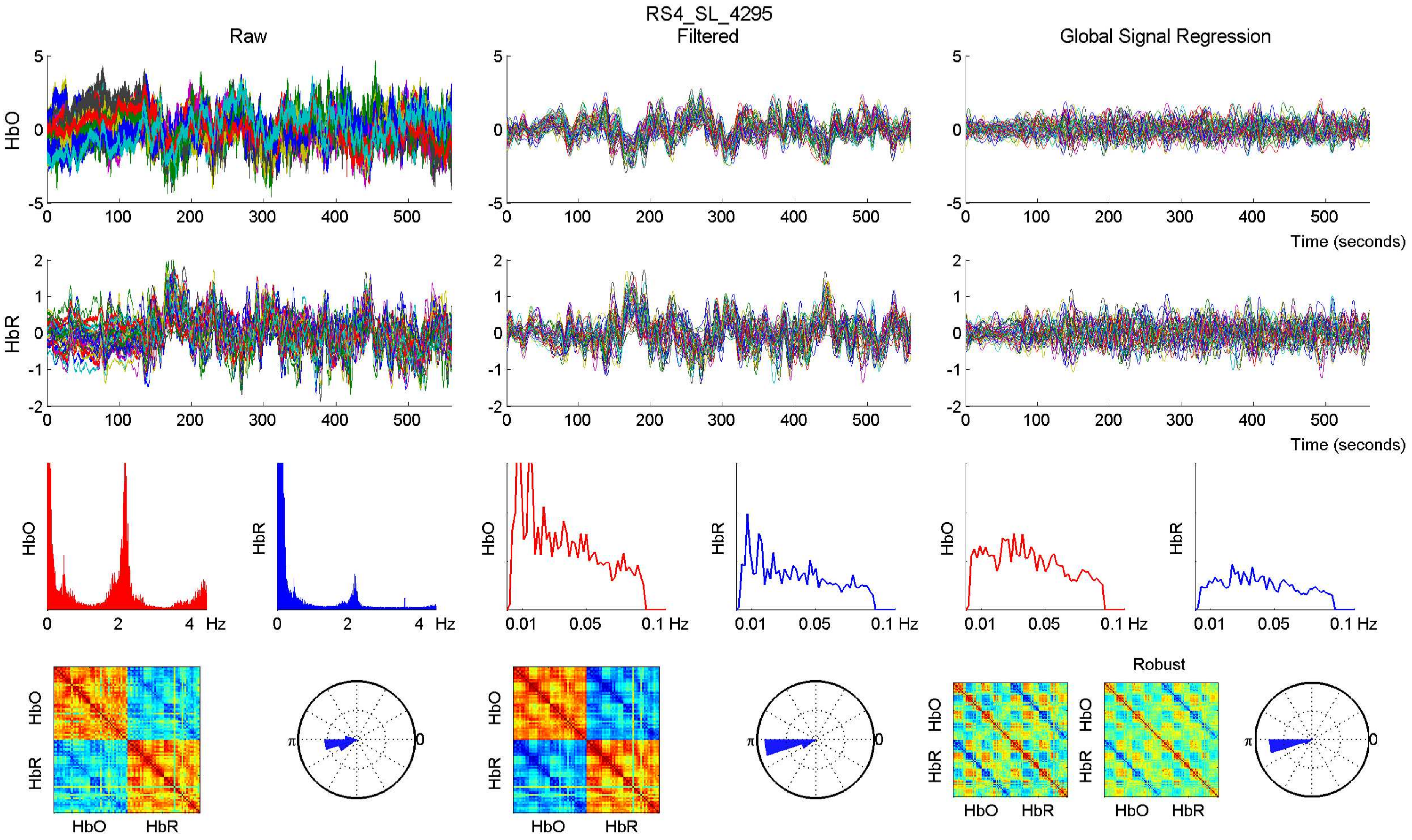

RS4_SL_4295
Raw
Filtered
Global Signal Regression
HbO
HbR
Time (seconds)
Hz
Robust

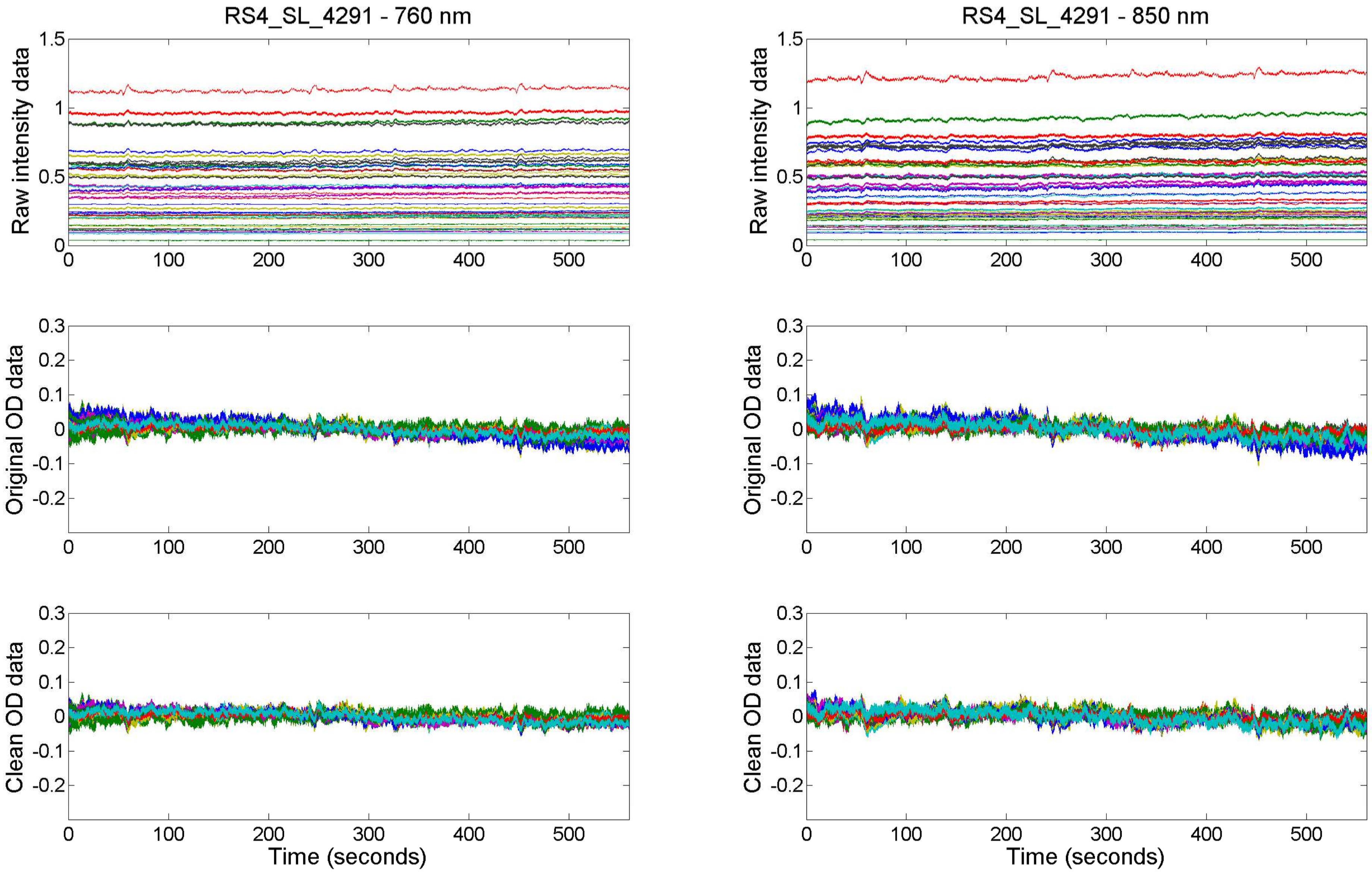
RS4_SL_4291 - 760 nm
RS4_SL_4291 - 850 nm
Raw intensity data
Original OD data
Clean OD data
Time (seconds)
0
100
200
300
400
500
1.5
1
0.5
0.3
0.2
0.1
-0.1
-0.2

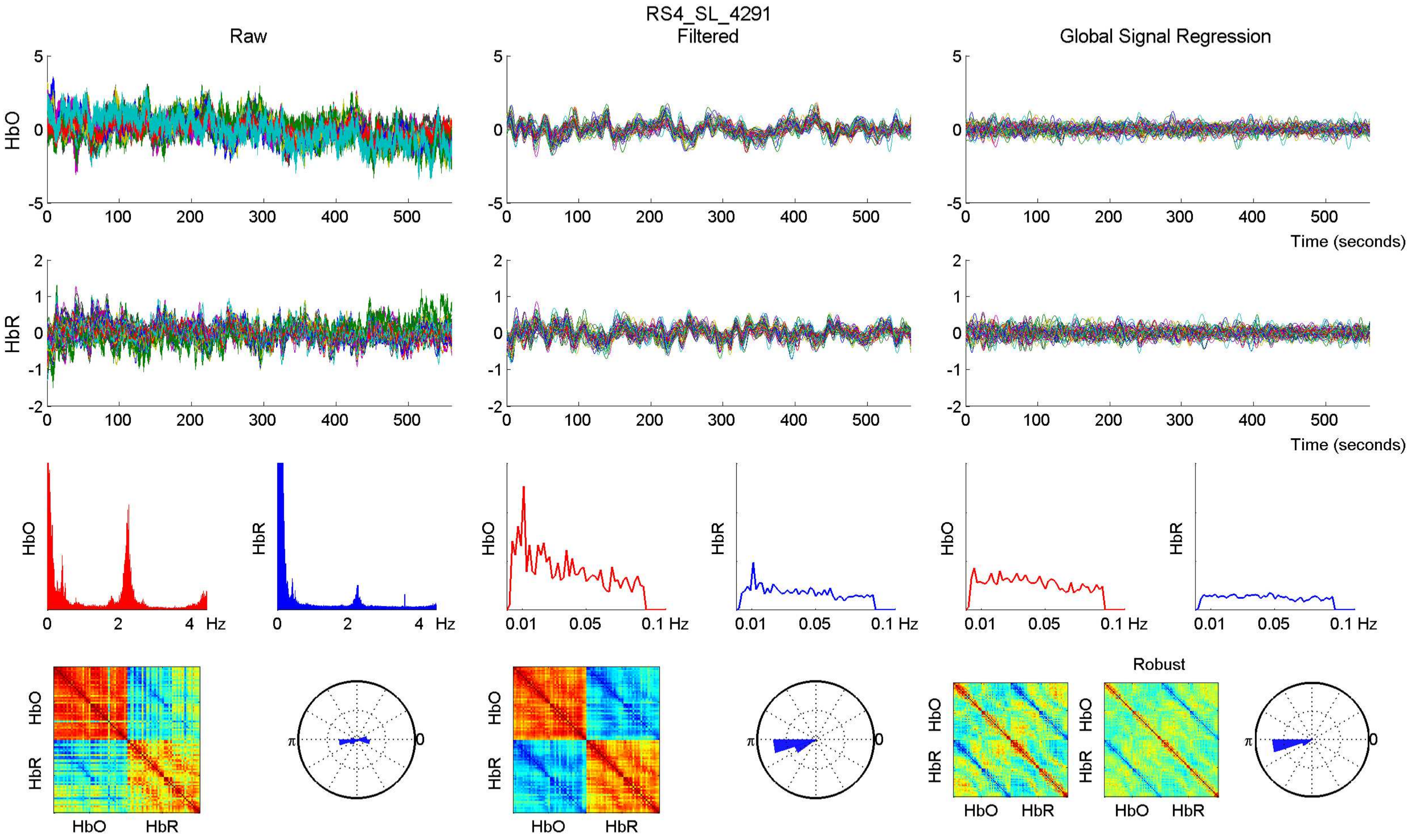
RS4_SL_4291
Raw
Filtered
Global Signal Regression
HbO
HbR
Time (seconds)
Hz
0.01
0.05
0.1 Hz
Robust
HbO
HbR

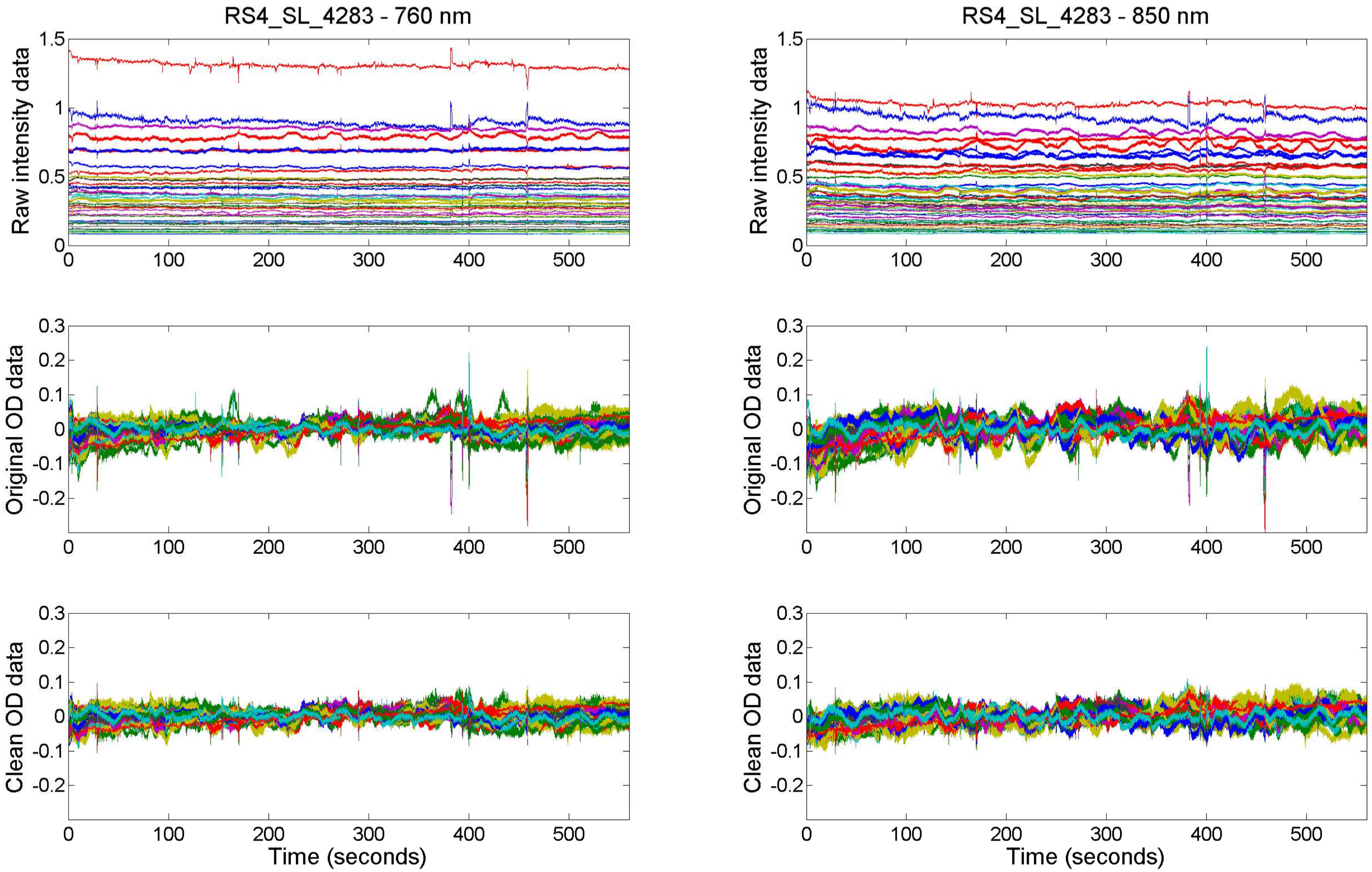
RS4_SL_4283 - 760 nm
RS4_SL_4283 - 850 nm
Raw intensity data
Original OD data
Clean OD data
Time (seconds)
1.5
1
0.5
0
0.3
0.2
0.1
-0.1
-0.2
100
200
300
400
500

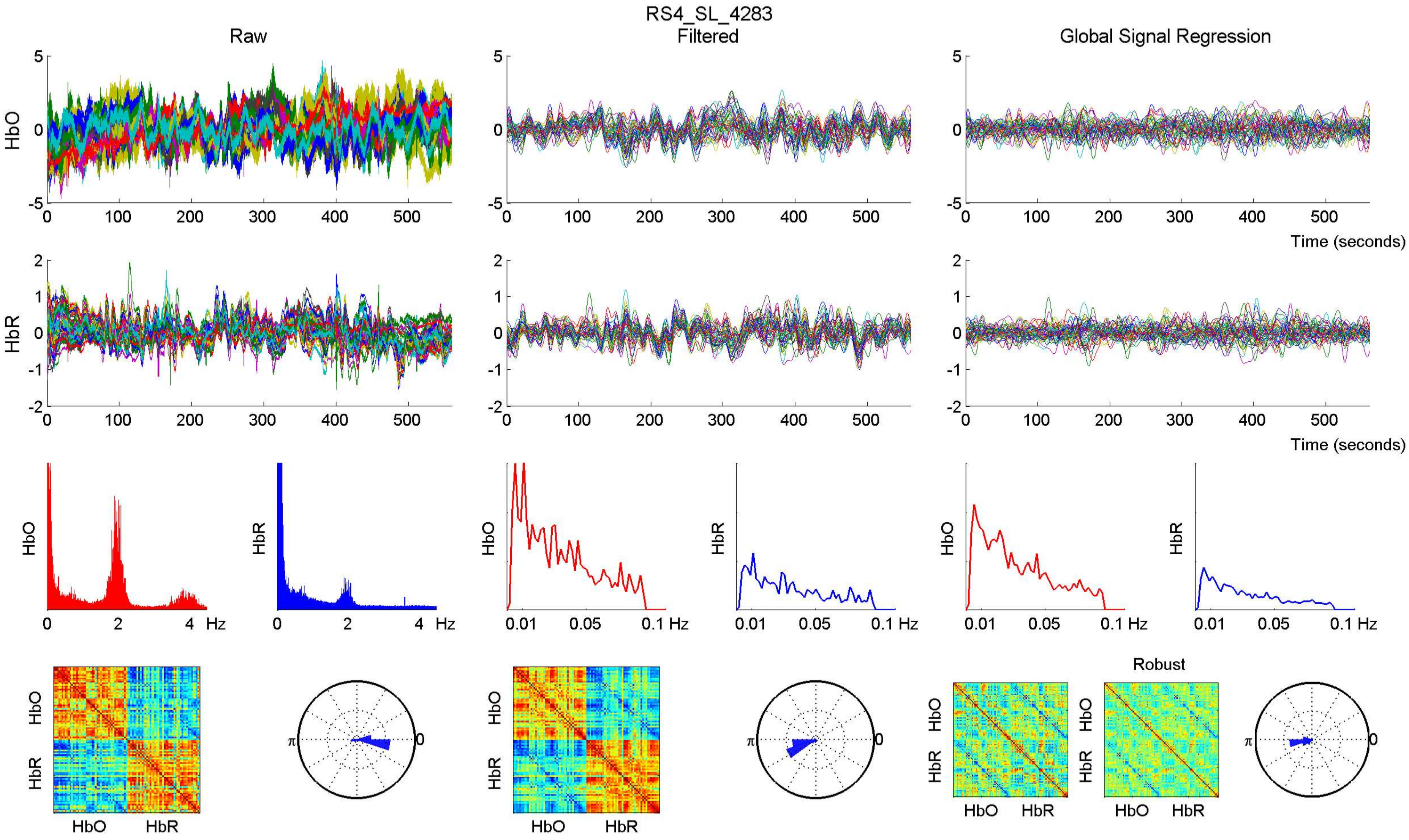
RS4_SL_4283
Raw
Filtered
Global Signal Regression
HbO
HbR
Time (seconds)
Hz
Robust
HbO
HbR

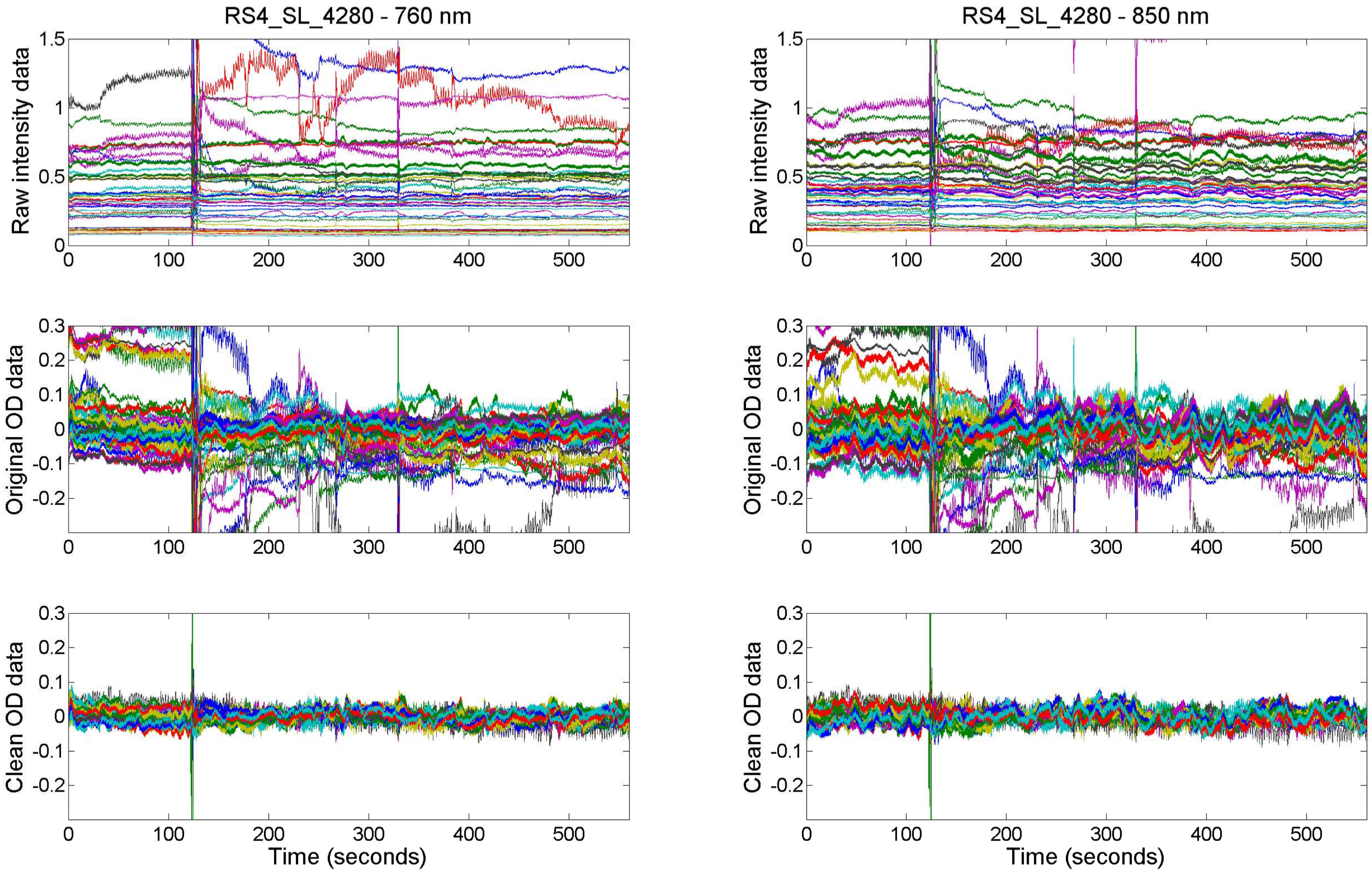
RS4_SL_4280 - 760 nm
RS4_SL_4280 - 850 nm
Raw intensity data
Original OD data
Clean OD data
Time (seconds)
0
100
200
300
400
500
1.5
1
0.5
0.3
0.2
0.1
-0.1
-0.2

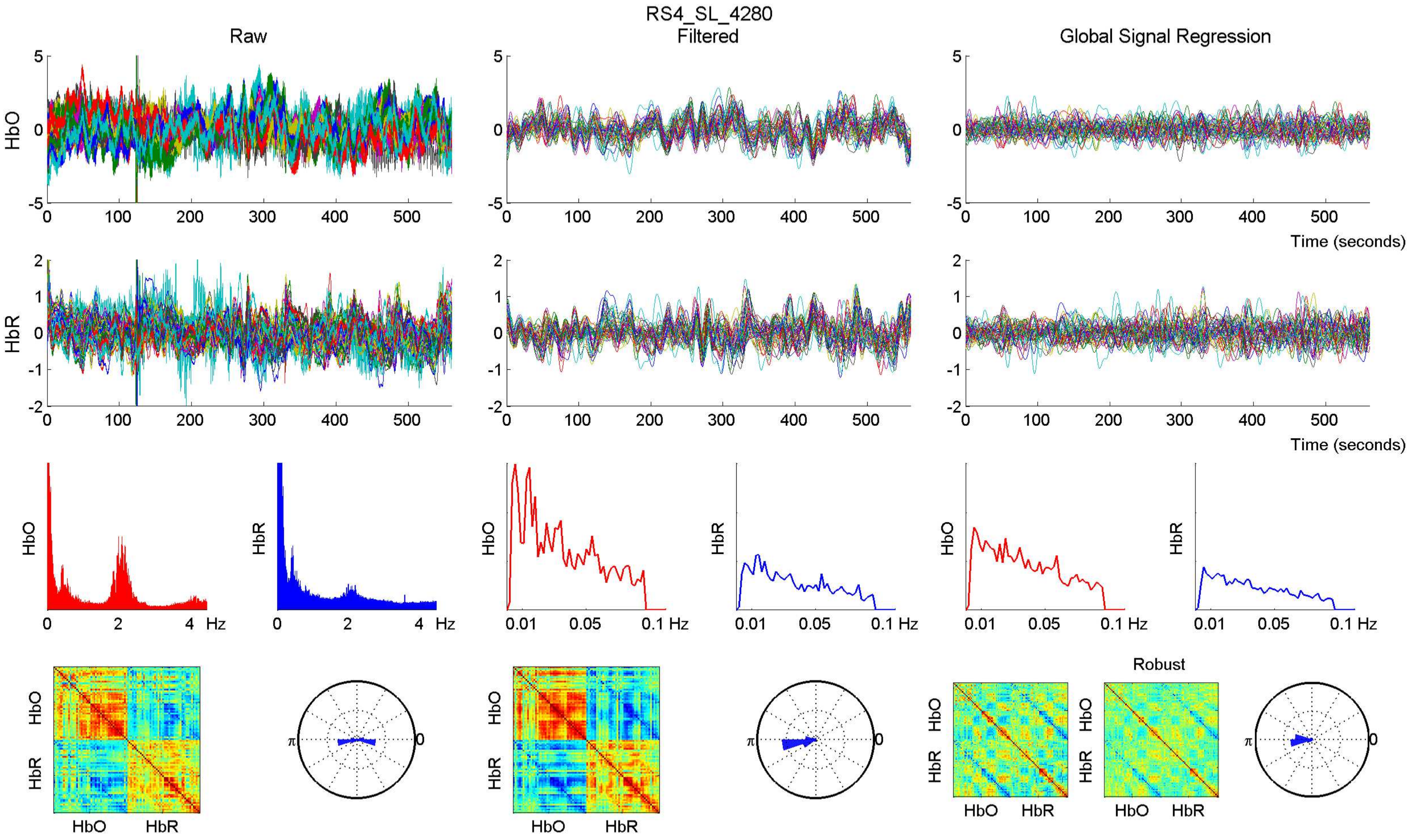
RS4_SL_4280
Raw
Filtered
Global Signal Regression
HbO
HbR
Time (seconds)
Hz
Robust

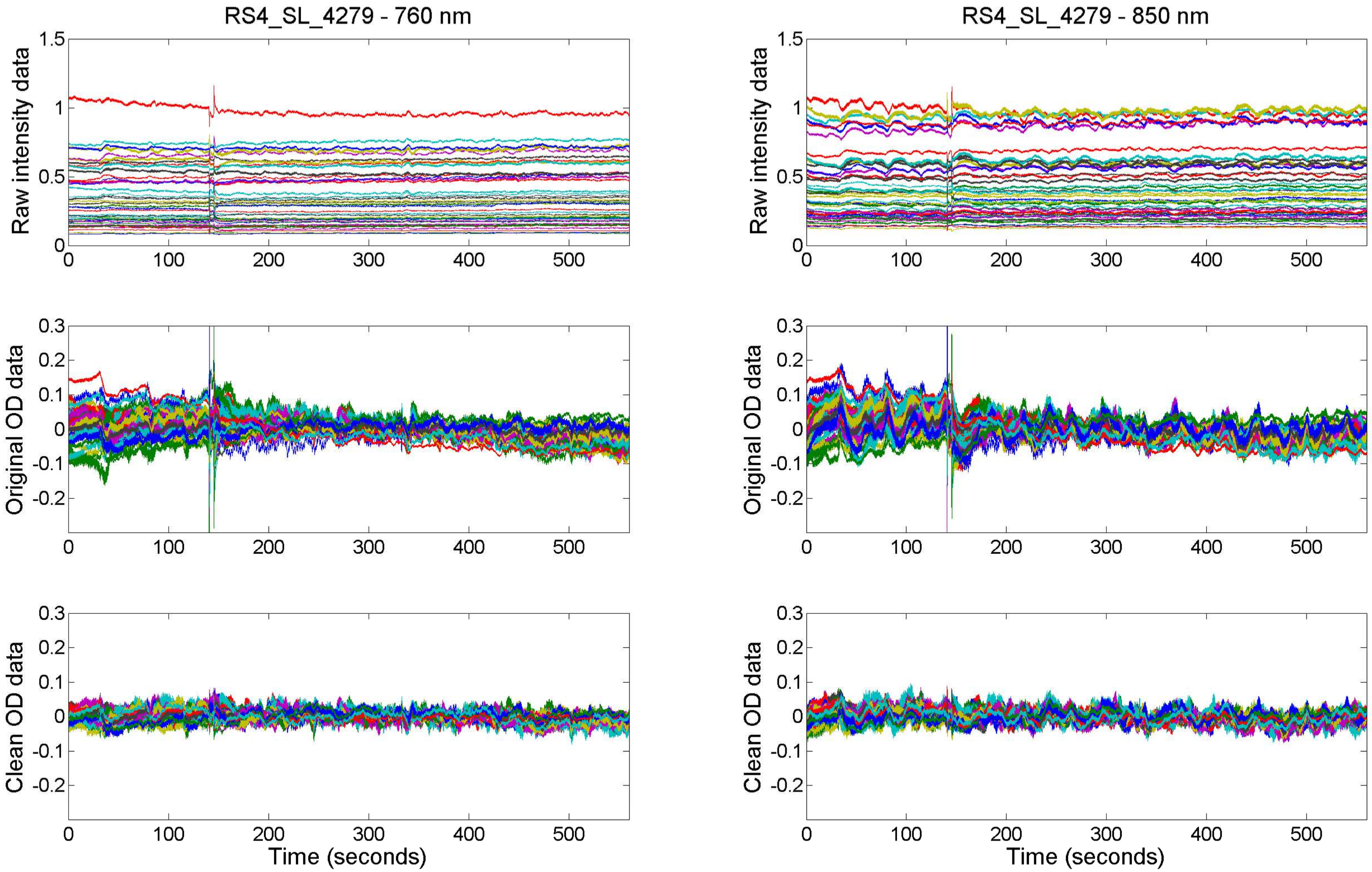

RS4_SL_4279 - 760 nm
RS4_SL_4279 - 850 nm
Raw intensity data
Original OD data
Clean OD data
1.5
1
0.5
0
0.3
0.2
0.1
-0.1
-0.2
100
200
300
400
500
Time (seconds)

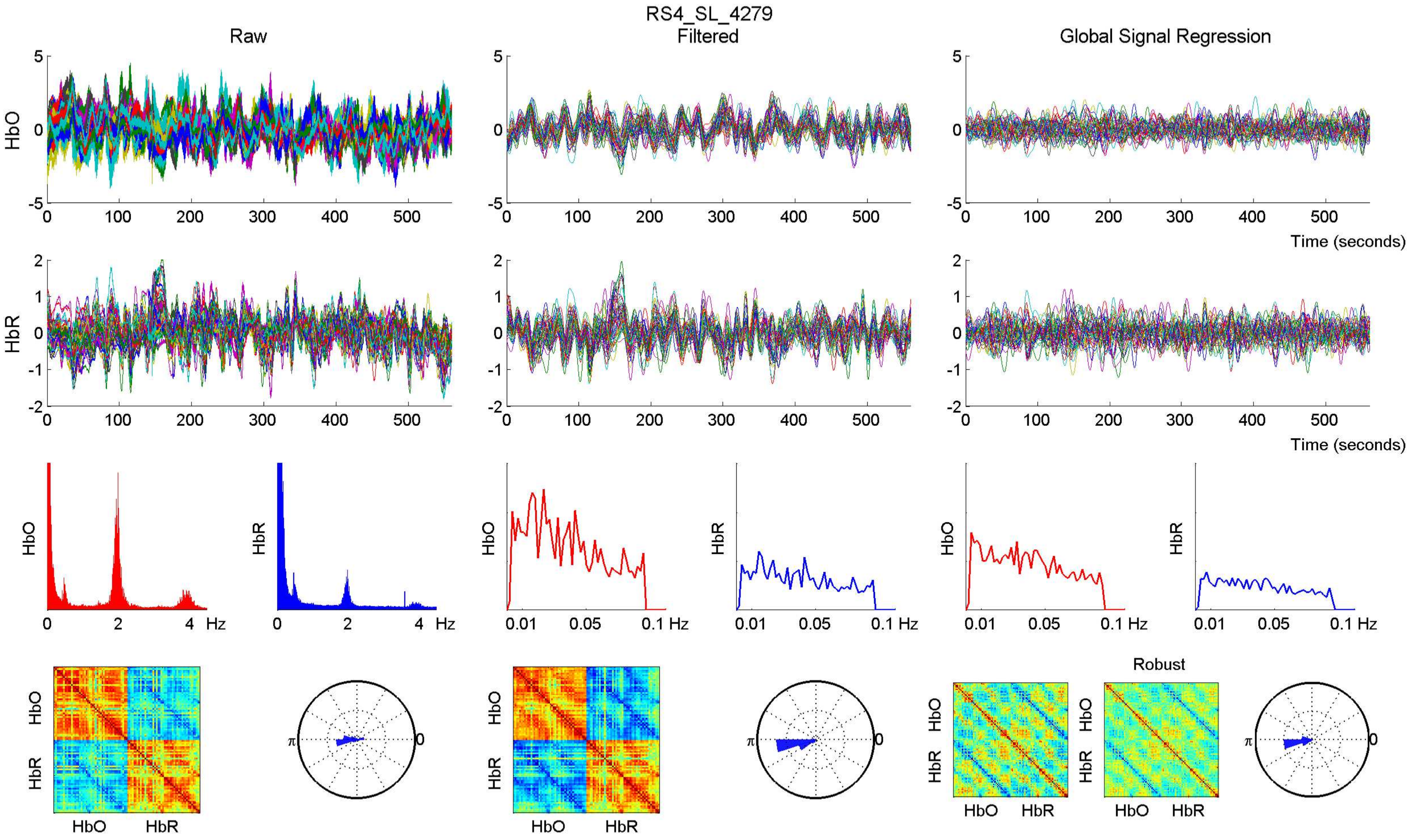

RS4_SL_4279
Raw
Filtered
Global Signal Regression
HbO
HbR
Time (seconds)
Hz
Robust

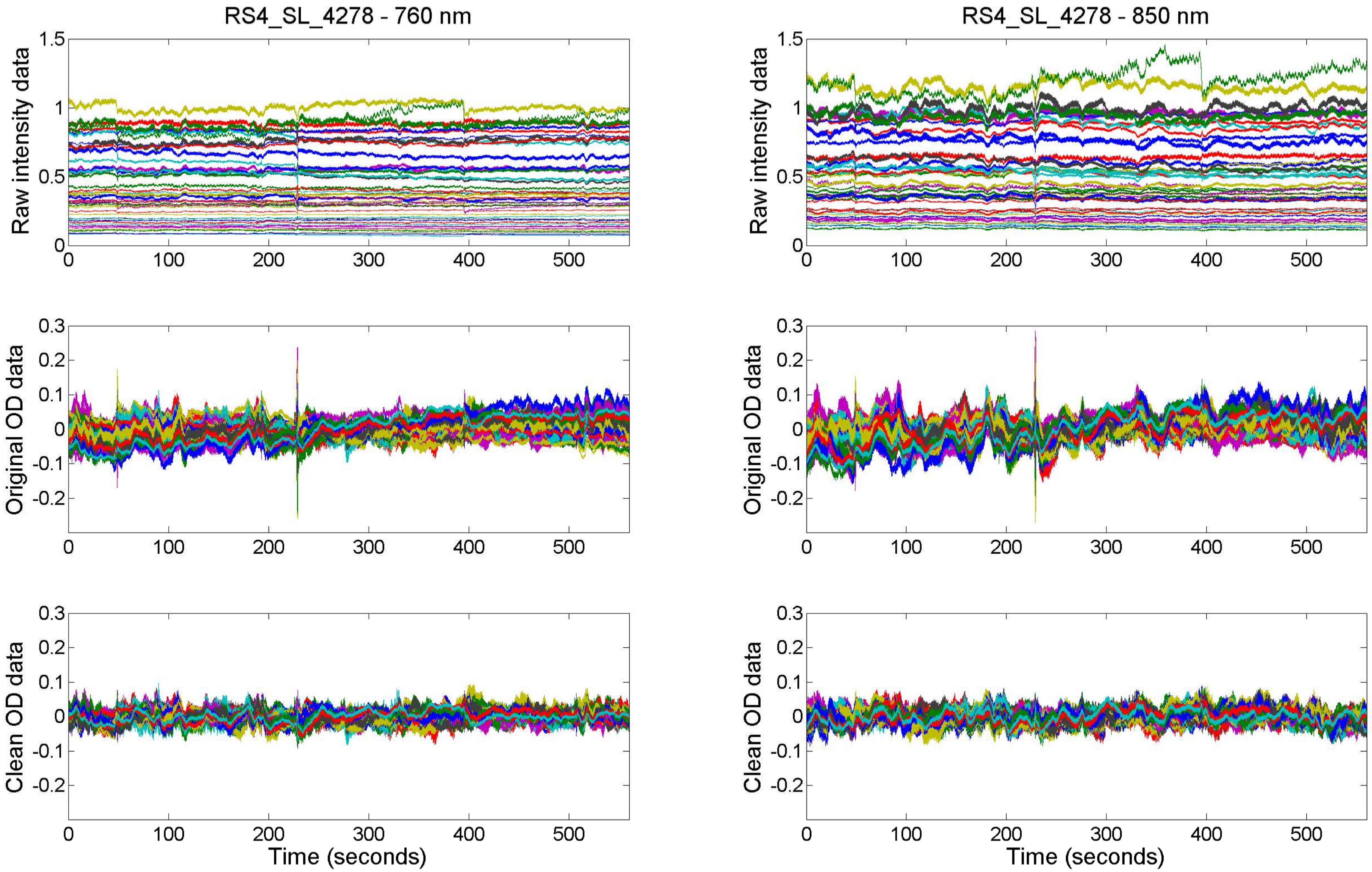
RS4_SL_4278 - 760 nm
RS4_SL_4278 - 850 nm
Raw intensity data
Original OD data
Clean OD data
Time (seconds)
0
100
200
300
400
500
1.5
1
0.5
0.3
0.2
0.1
-0.1
-0.2

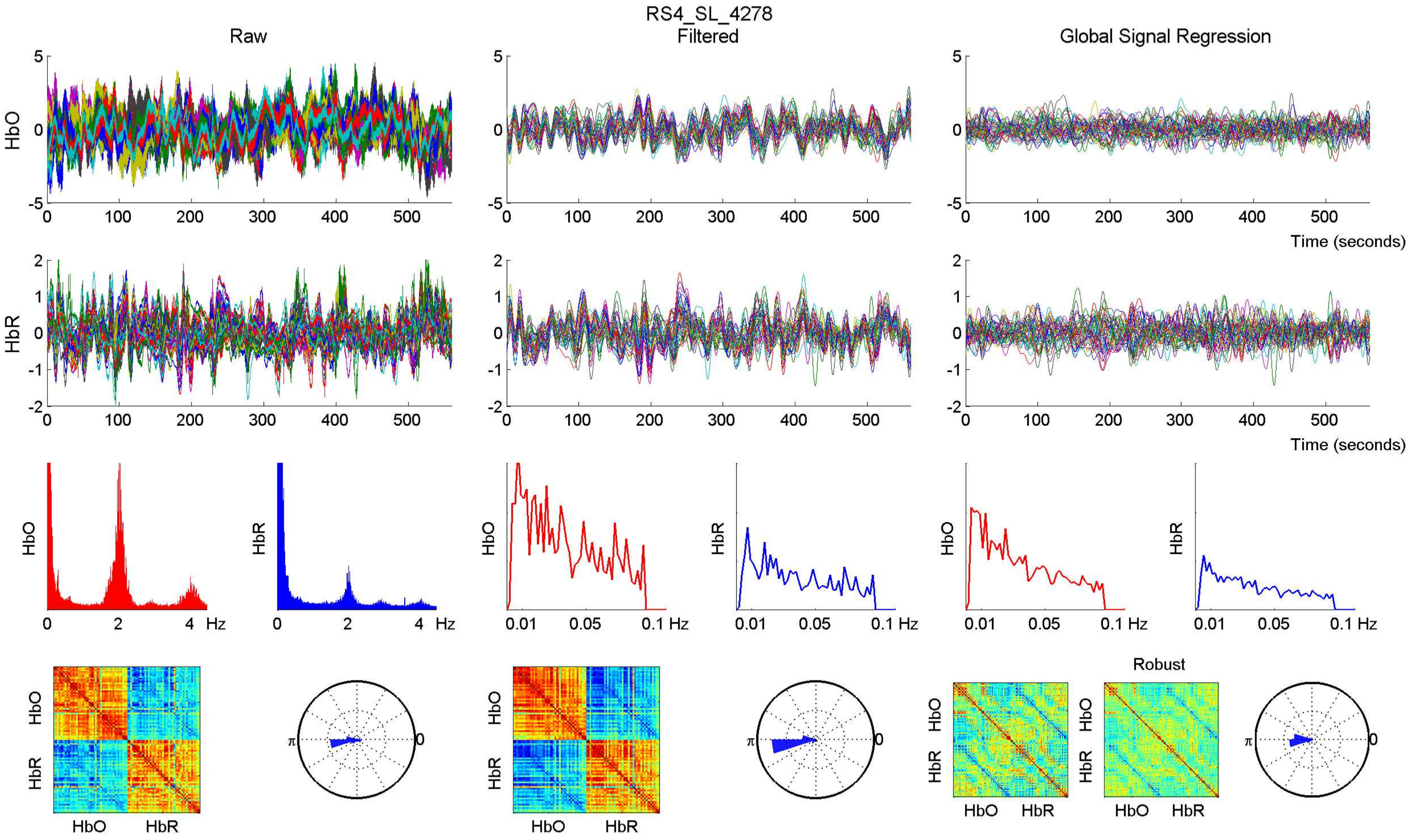

RS4_SL_4278
Raw
Filtered
Global Signal Regression
HbO
HbR
Time (seconds)
Hz
Robust

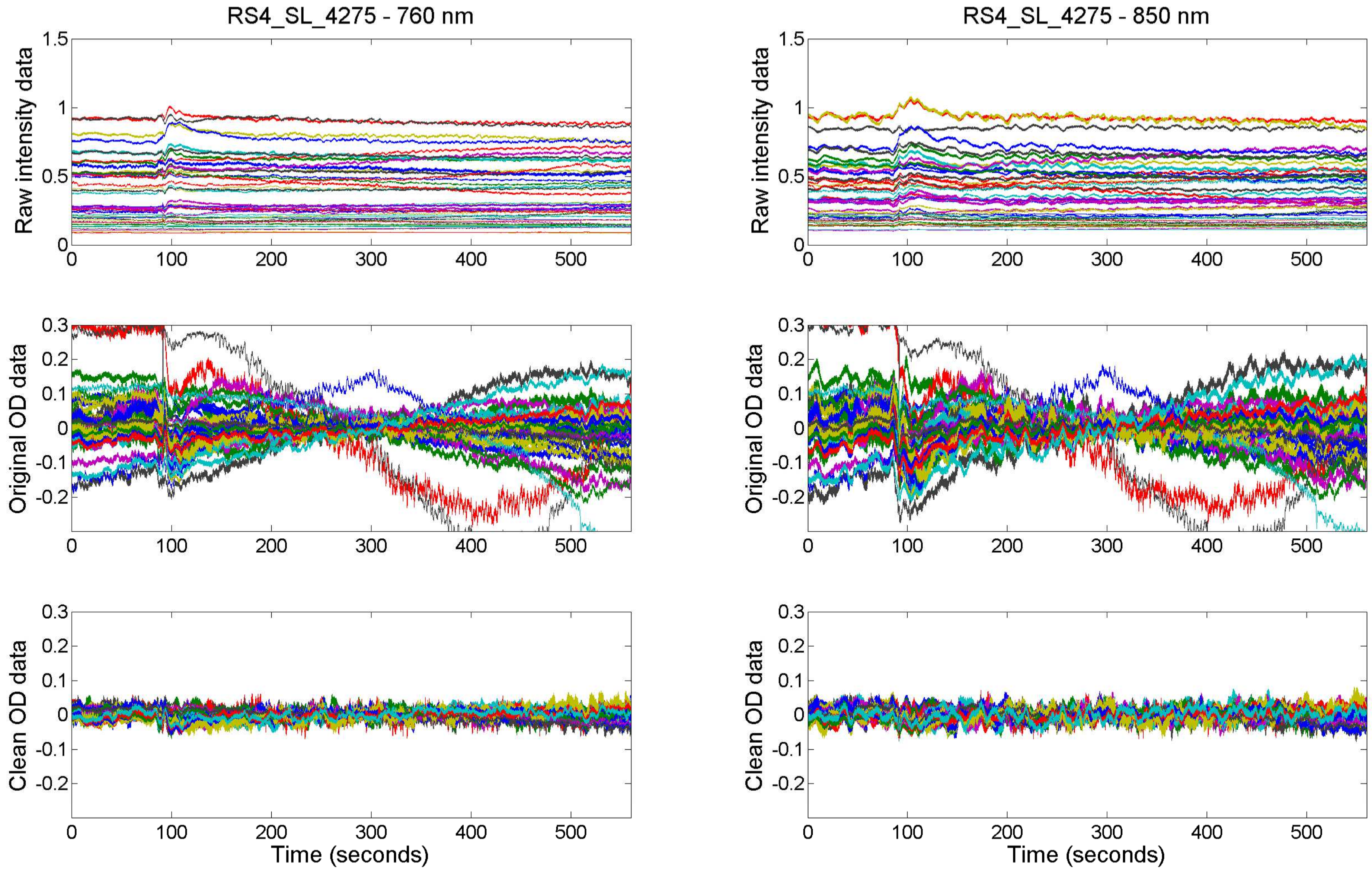

RS4_SL_4275 - 760 nm
RS4_SL_4275 - 850 nm
Raw intensity data
Original OD data
Clean OD data
Time (seconds)

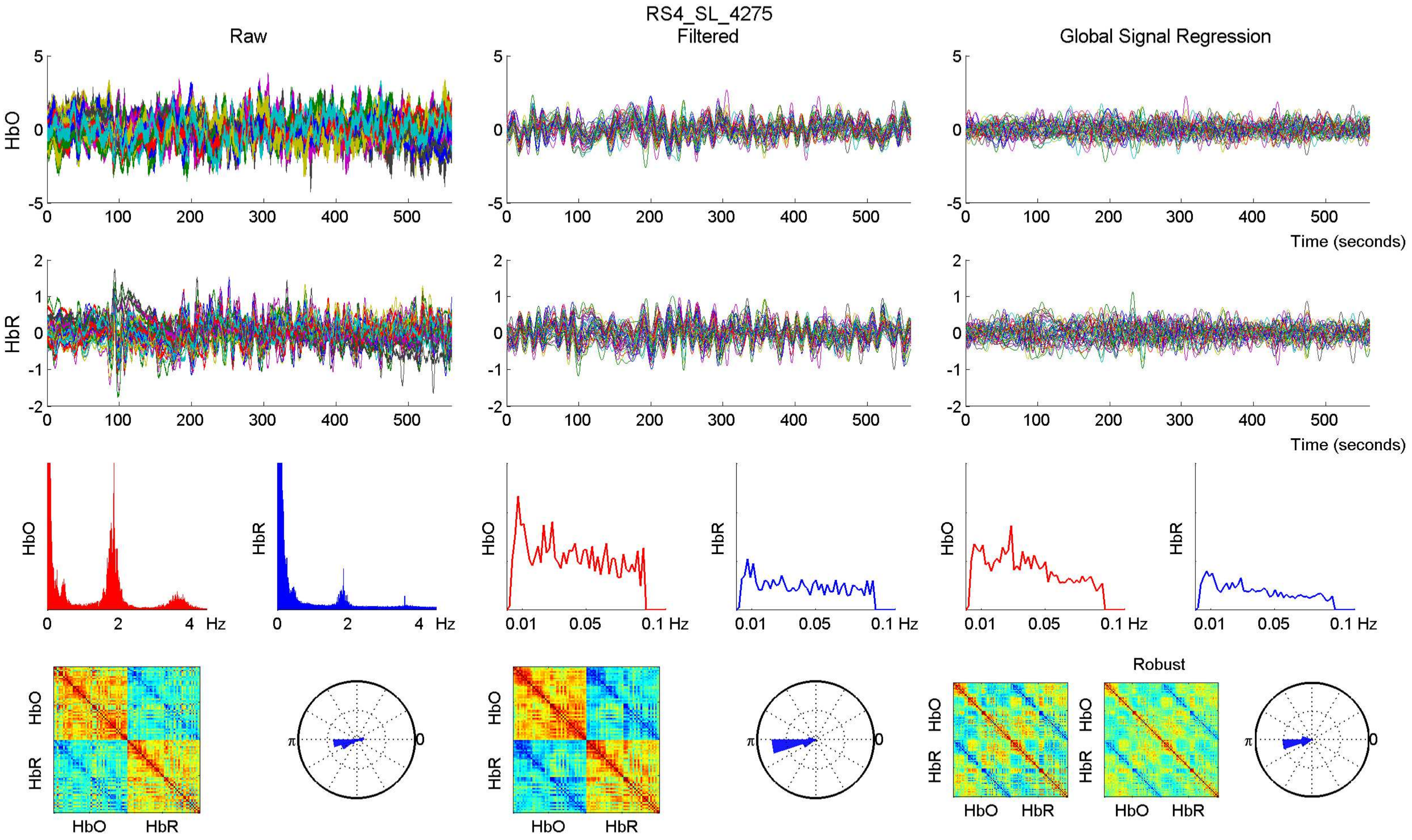
RS4_SL_4275
Raw
Filtered
Global Signal Regression
HbO
HbR
Time (seconds)
Hz
Robust

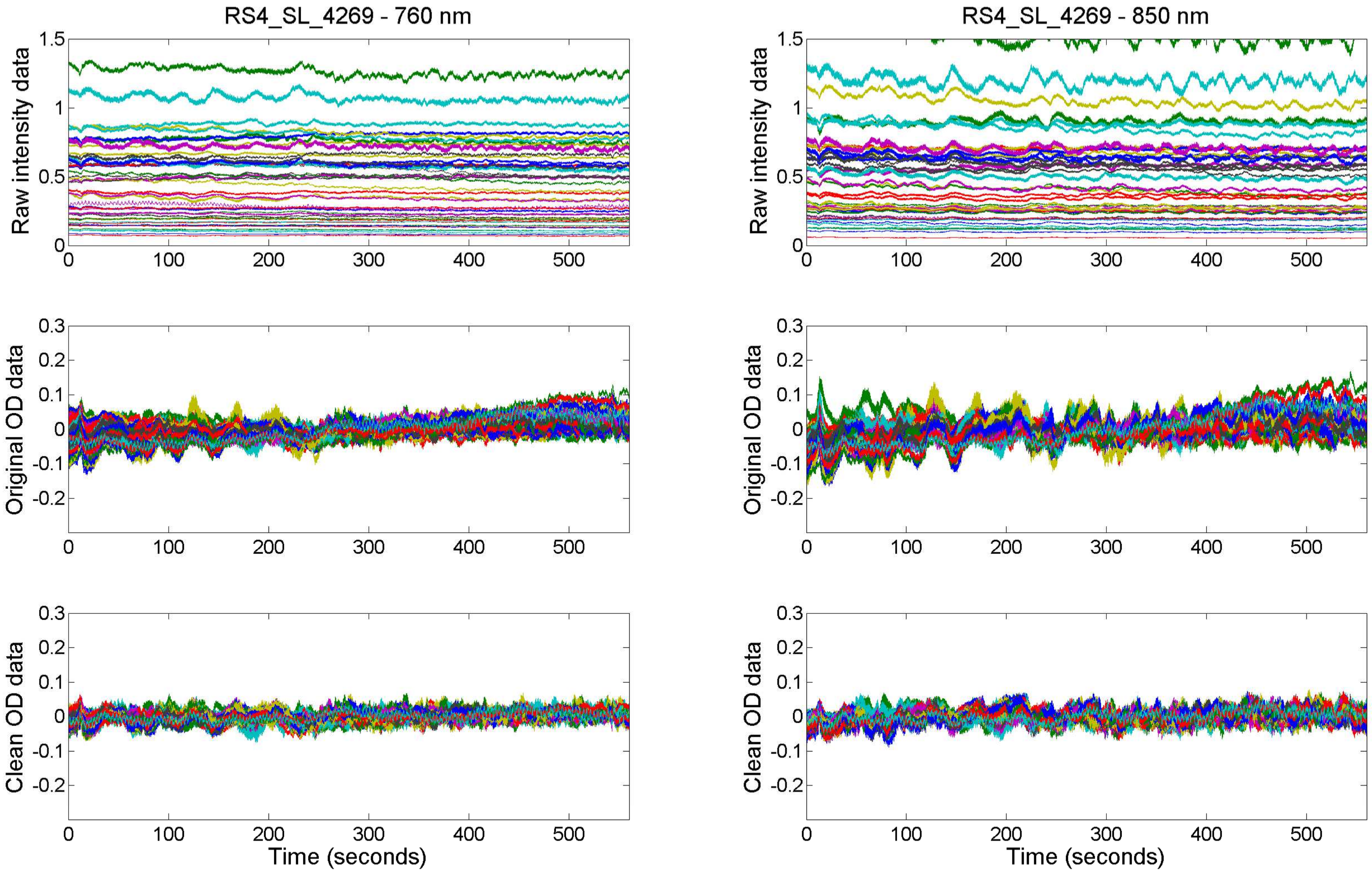
RS4_SL_4269 - 760 nm
RS4_SL_4269 - 850 nm
Raw intensity data
Original OD data
Clean OD data
Time (seconds)
0
100
200
300
400
500
0
0.5
1
1.5
-0.2
-0.1
0.1
0.2
0.3

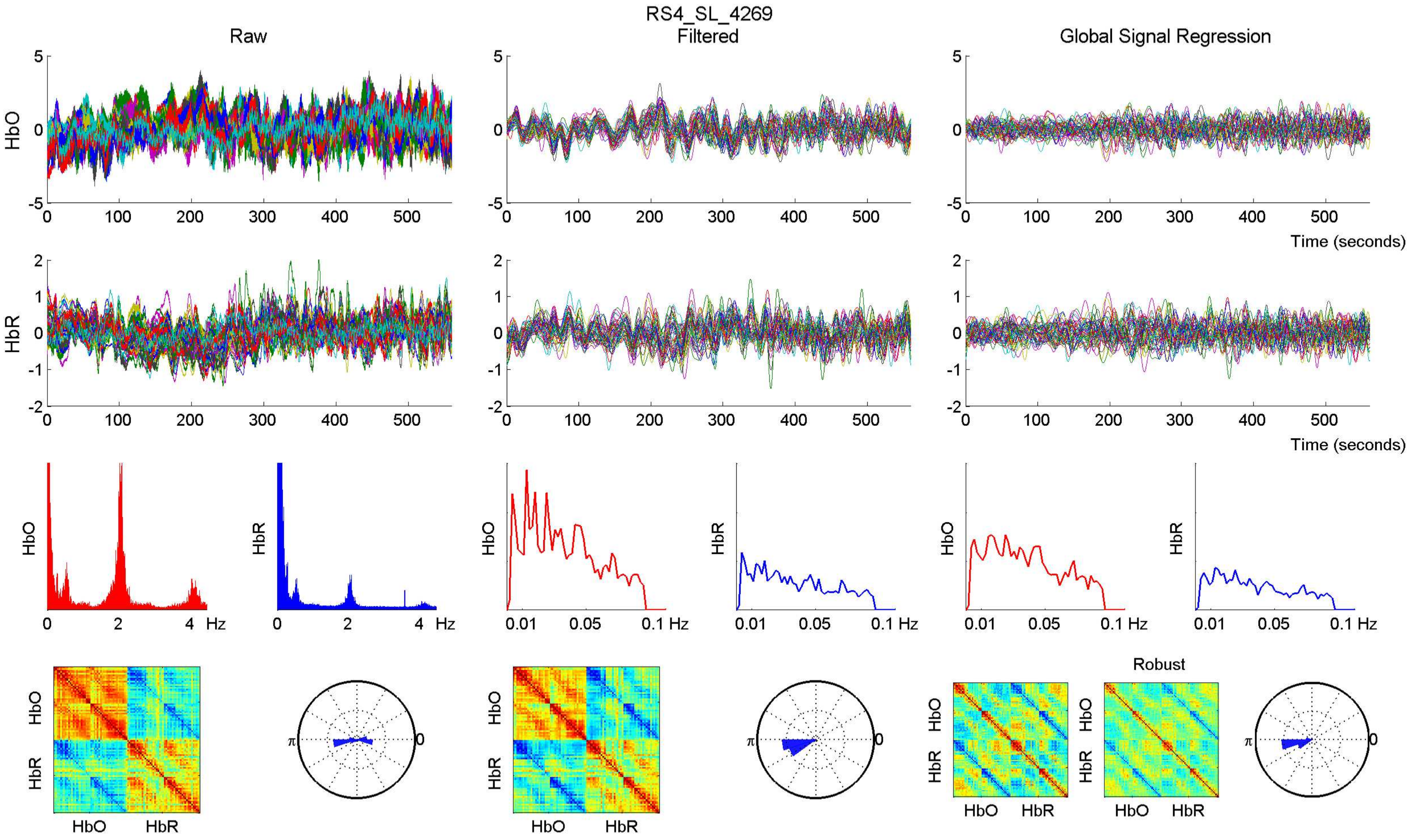

RS4_SL_4269
Raw
Filtered
Global Signal Regression
HbO
HbR
Time (seconds)
Hz
0.01
0.05
0.1 Hz
Robust
π
0

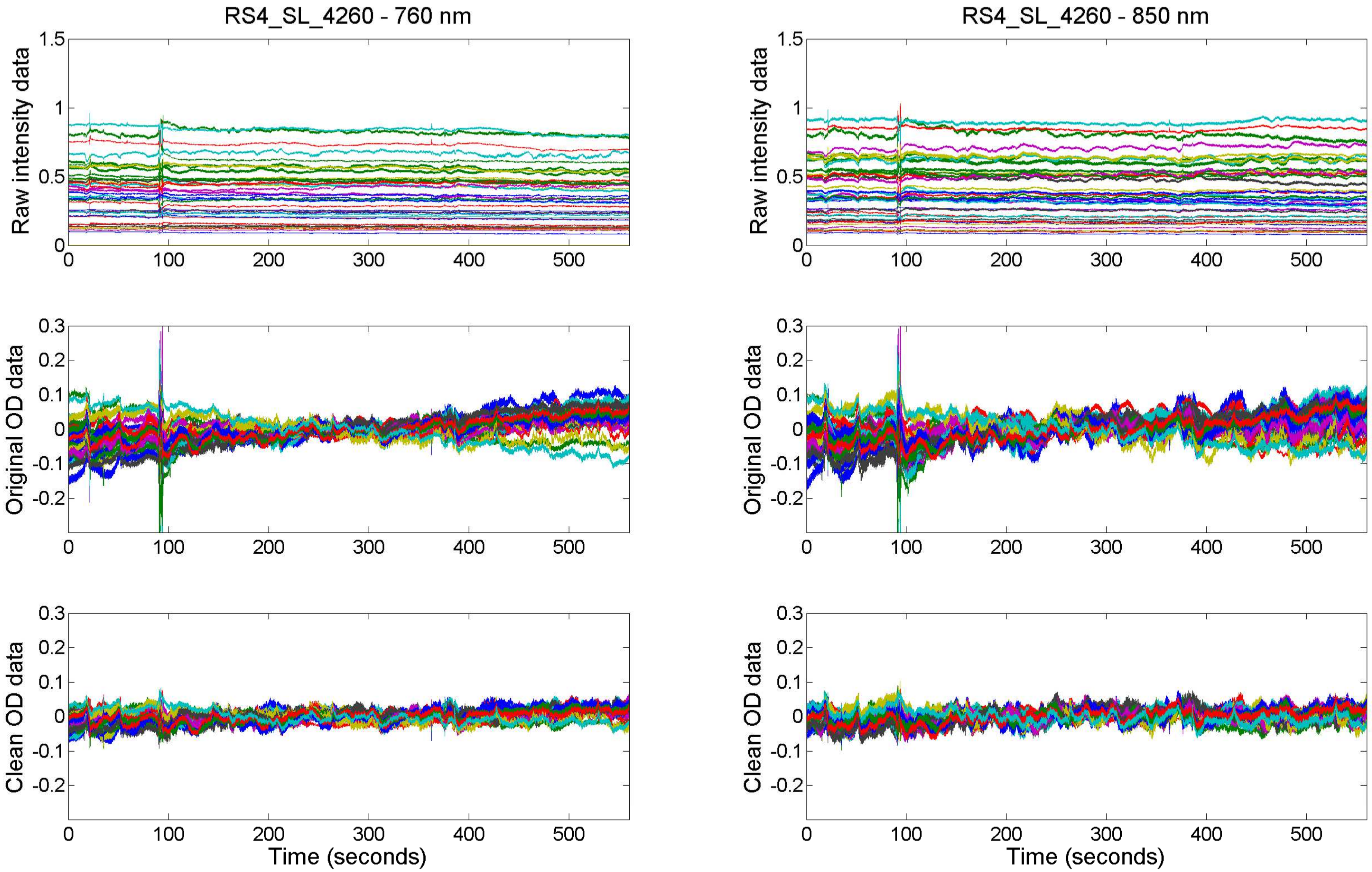
RS4_SL_4260 - 760 nm
RS4_SL_4260 - 850 nm
Raw intensity data
Original OD data
Clean OD data
Time (seconds)

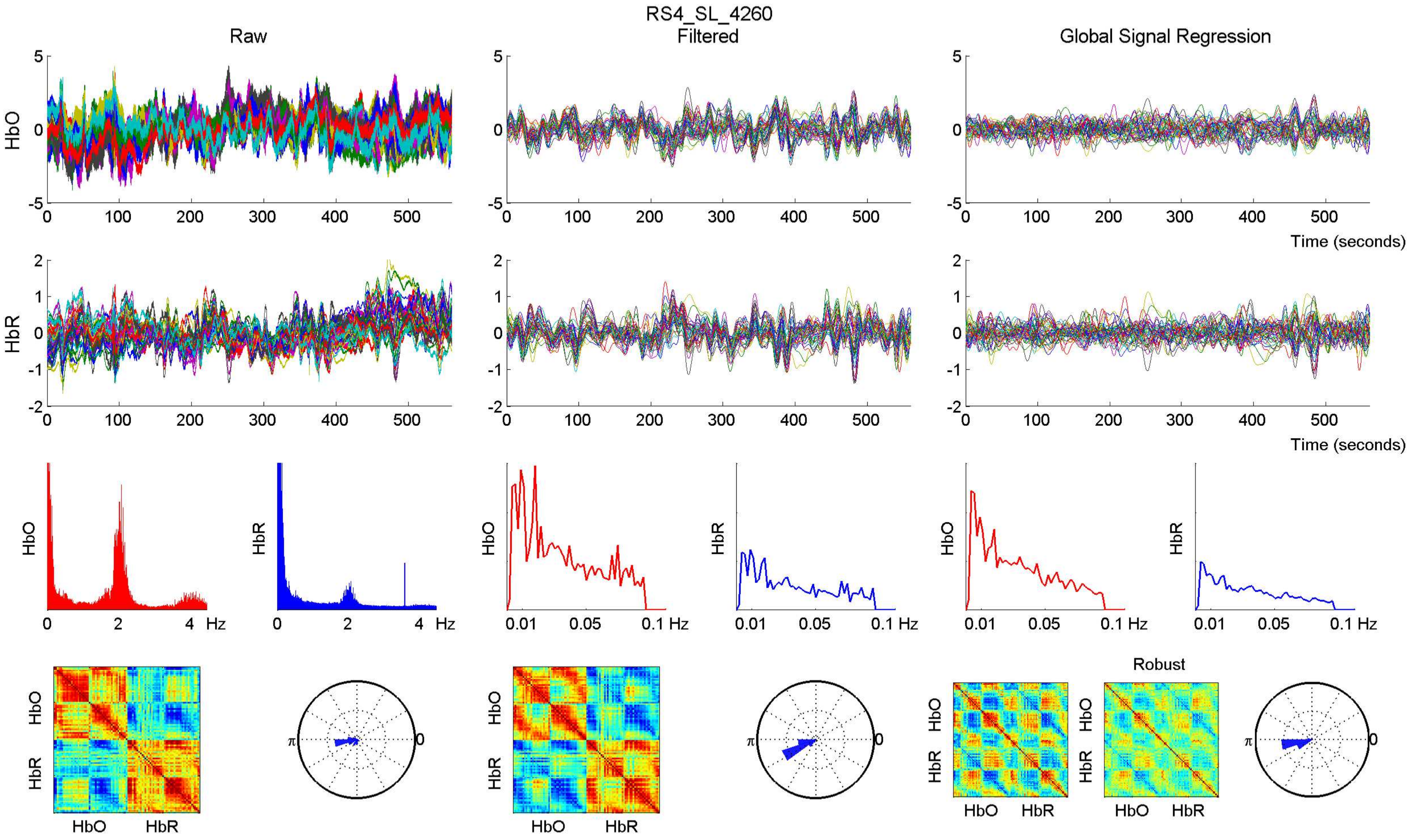

RS4_SL_4260
Raw
Filtered
Global Signal Regression
HbO
HbR
Time (seconds)
Hz
Robust

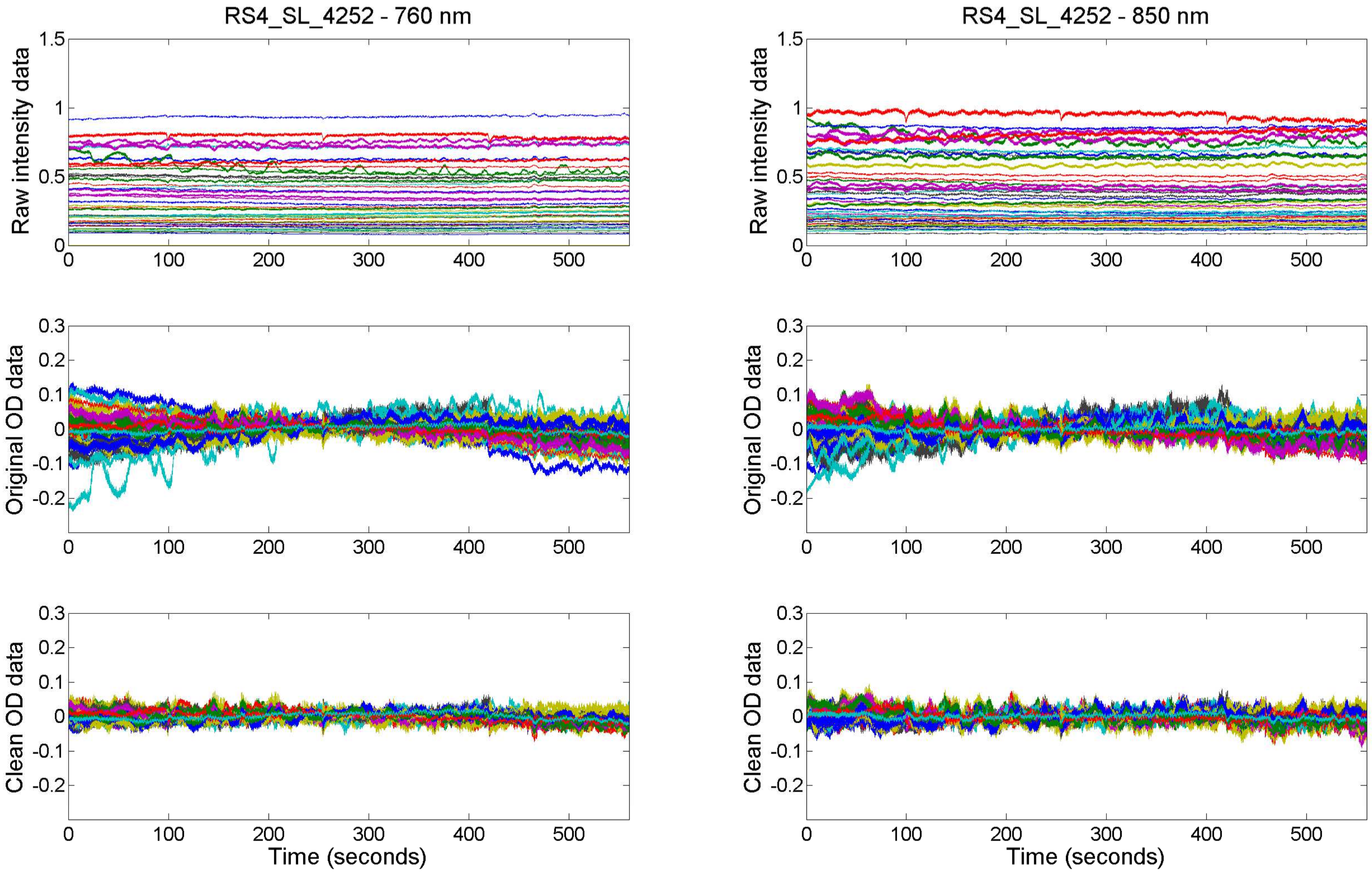
RS4_SL_4252 - 760 nm
RS4_SL_4252 - 850 nm
Raw intensity data
Original OD data
Clean OD data
Time (seconds)

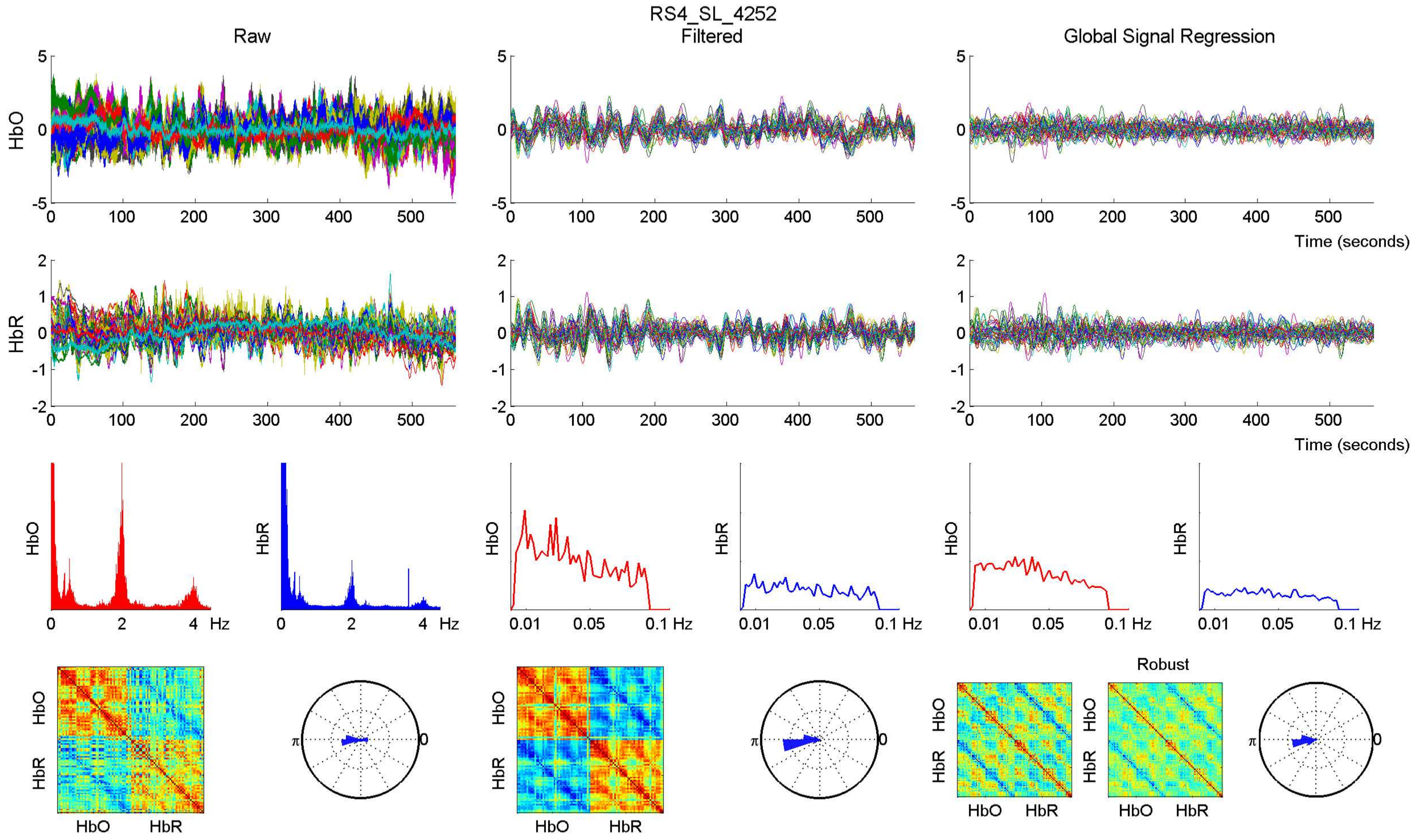

RS4_SL_4252
Raw
Filtered
Global Signal Regression
HbO
HbR
Time (seconds)
Hz
Robust

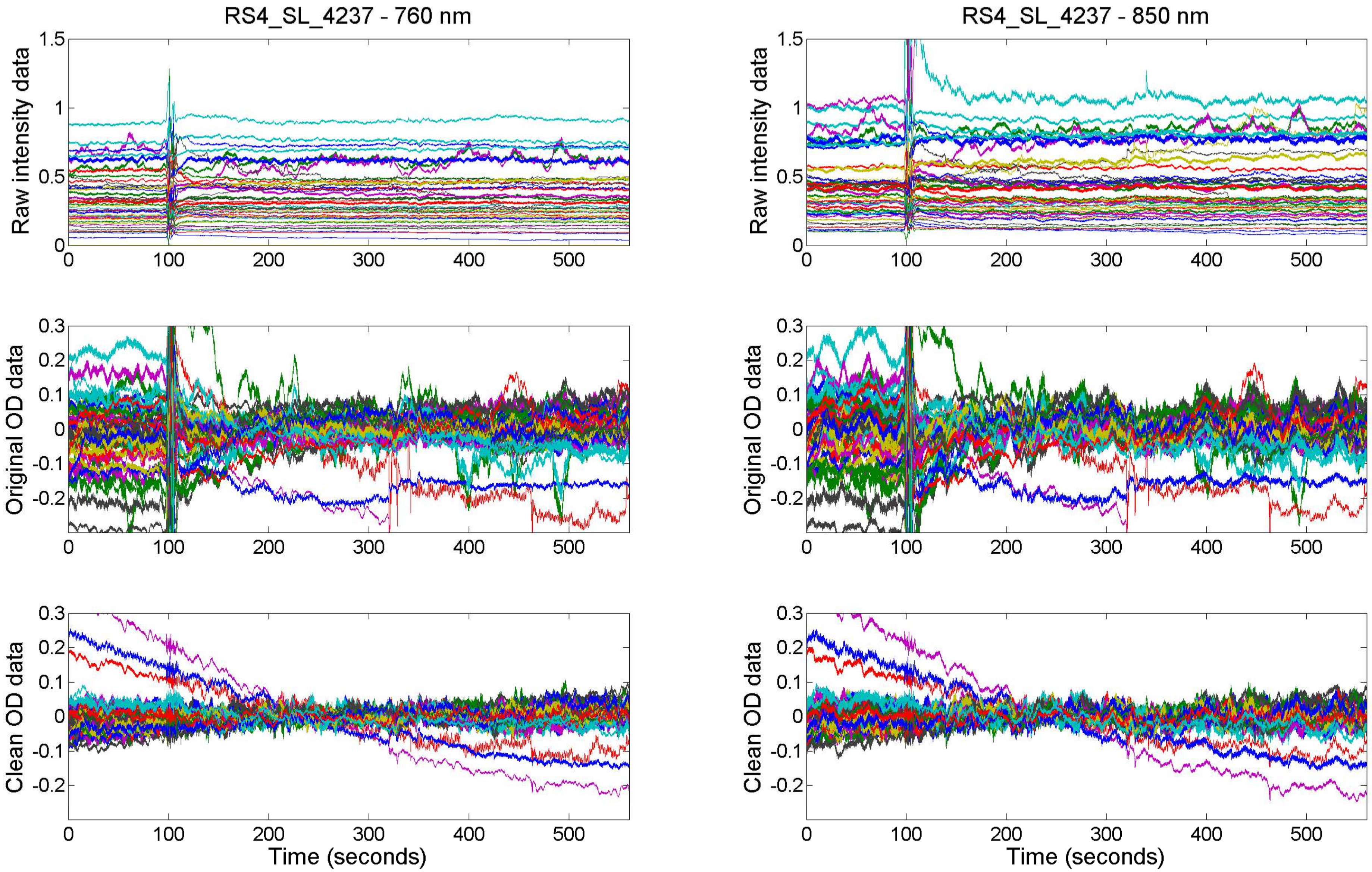

RS4_SL_4237 - 760 nm
RS4_SL_4237 - 850 nm
Raw intensity data
Original OD data
Clean OD data
Time (seconds)

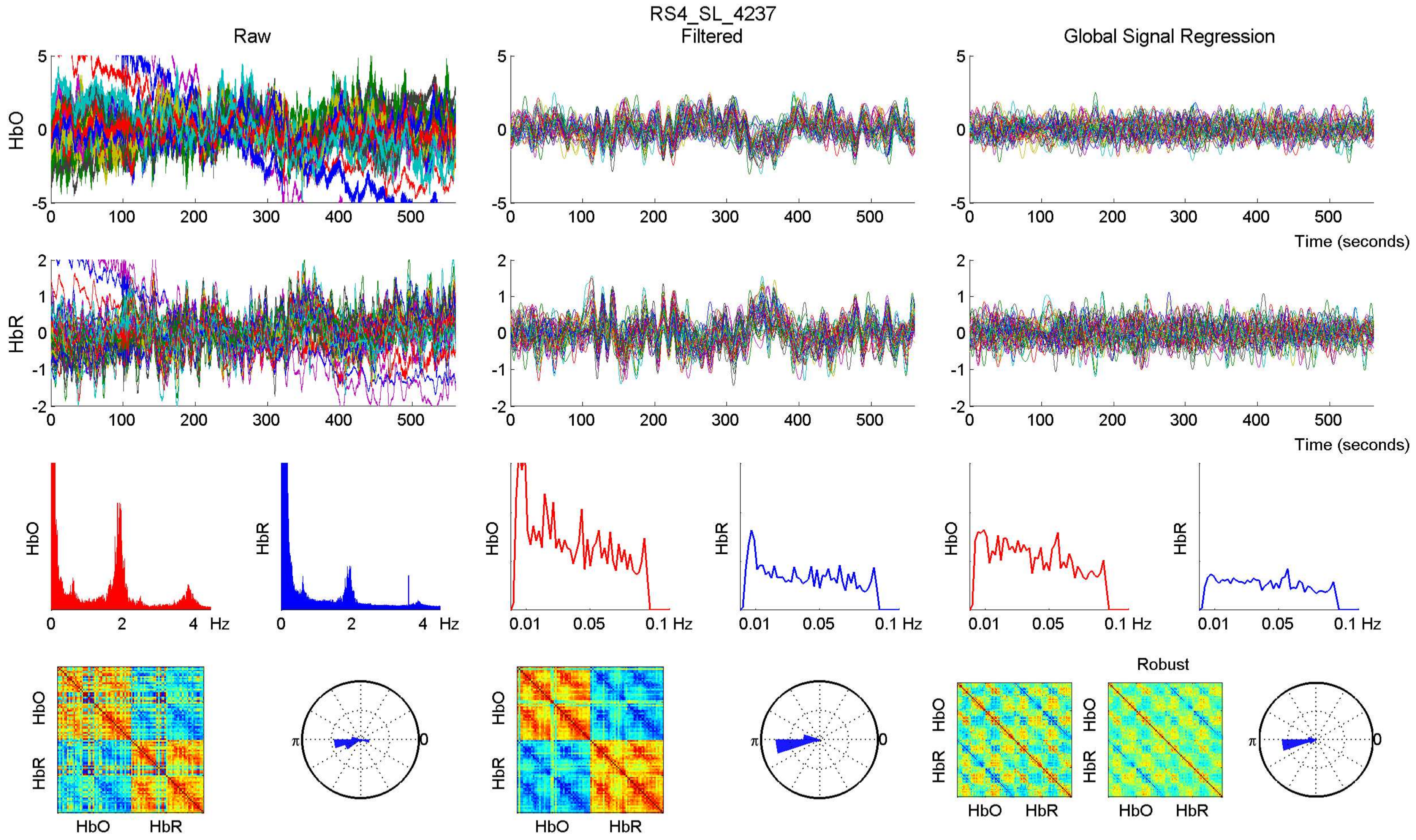

RS4_SL_4237
Raw
Filtered
Global Signal Regression
HbO
HbR
Time (seconds)
Hz
Robust

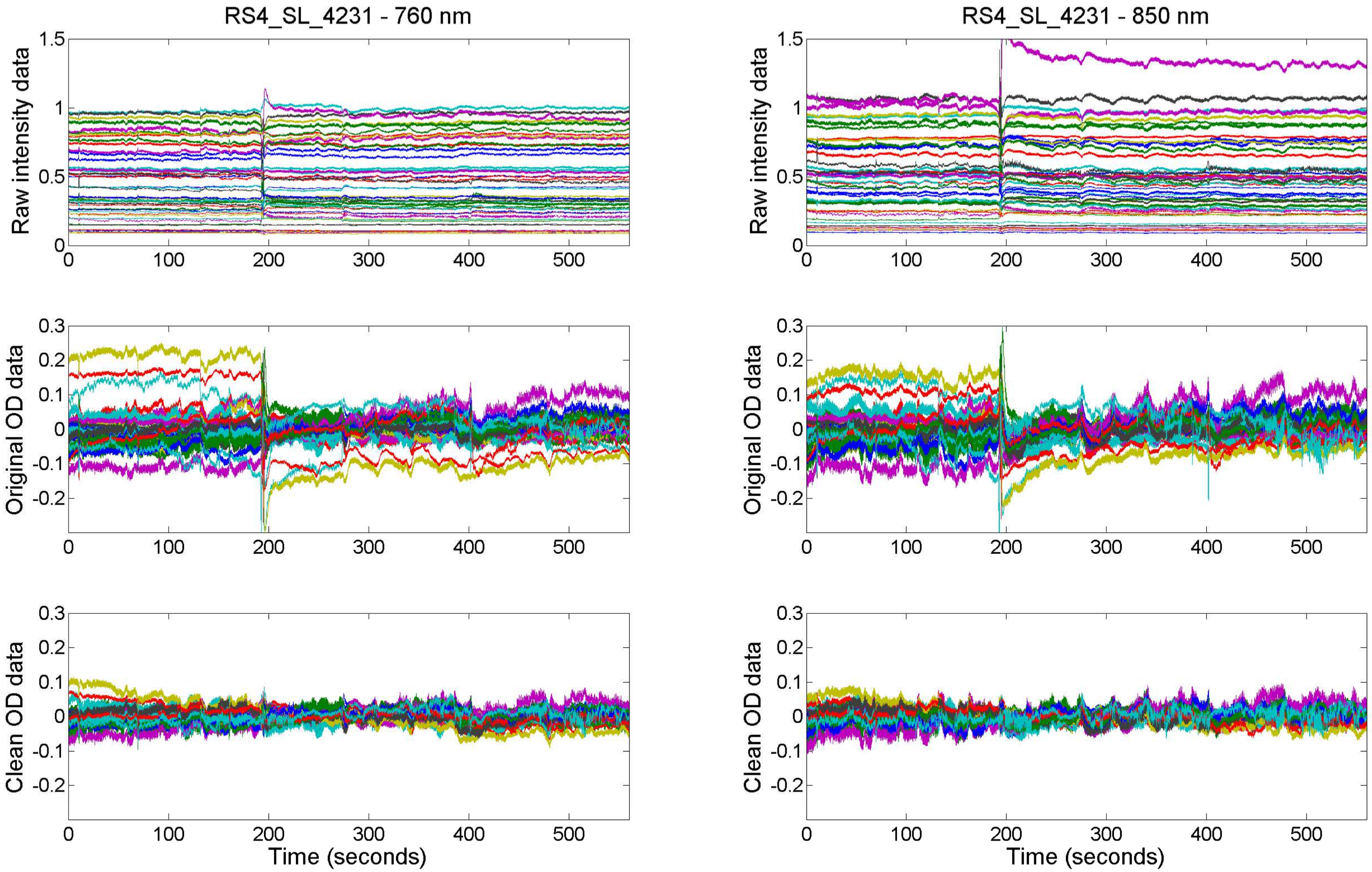
RS4_SL_4231 - 760 nm
RS4_SL_4231 - 850 nm
Raw intensity data
Original OD data
Clean OD data
Time (seconds)
1.5
1
0.5
0
0.3
0.2
0.1
-0.1
-0.2
100
200
300
400
500

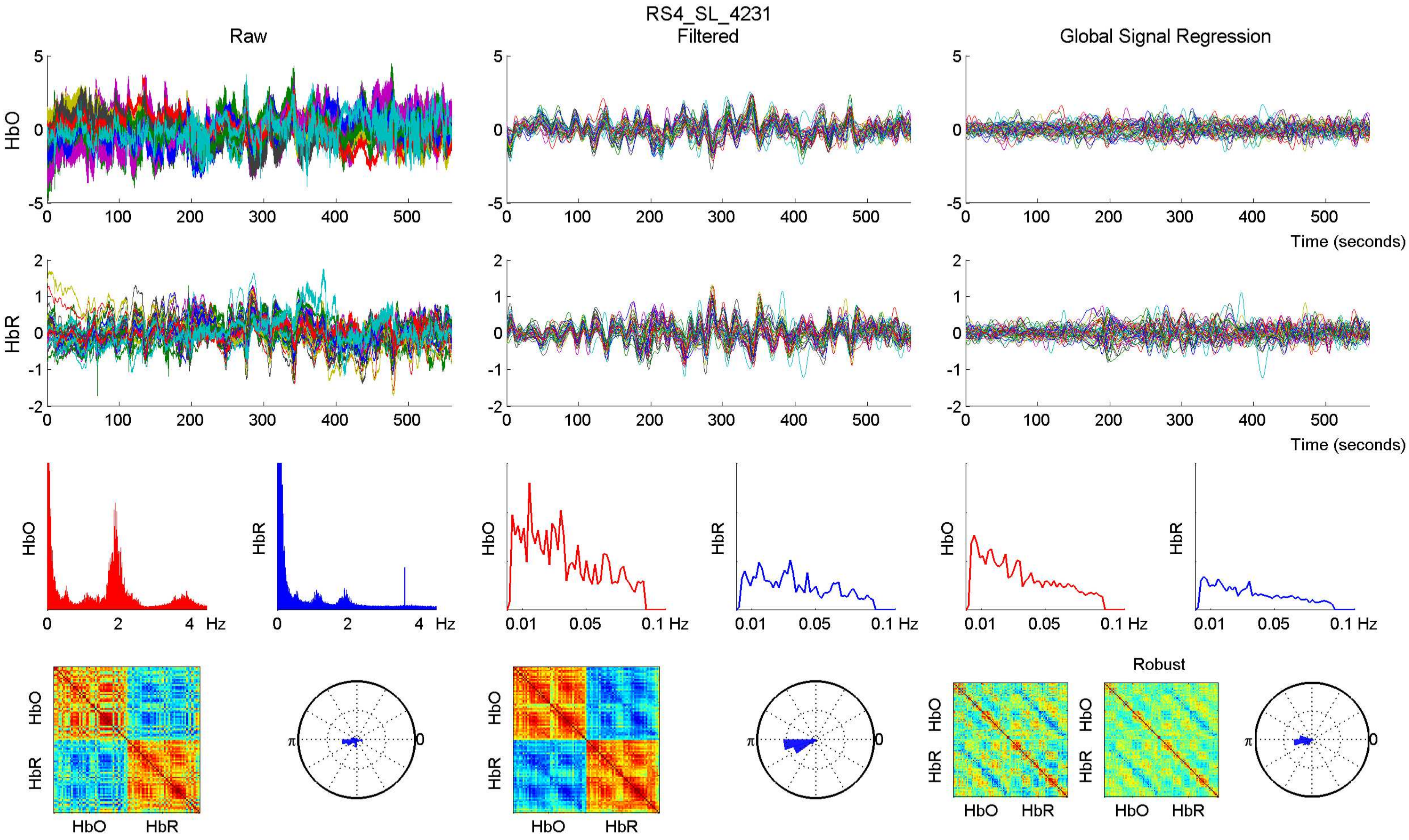

RS4_SL_4231
Raw
Filtered
Global Signal Regression
HbO
HbR
Time (seconds)
Hz
Robust

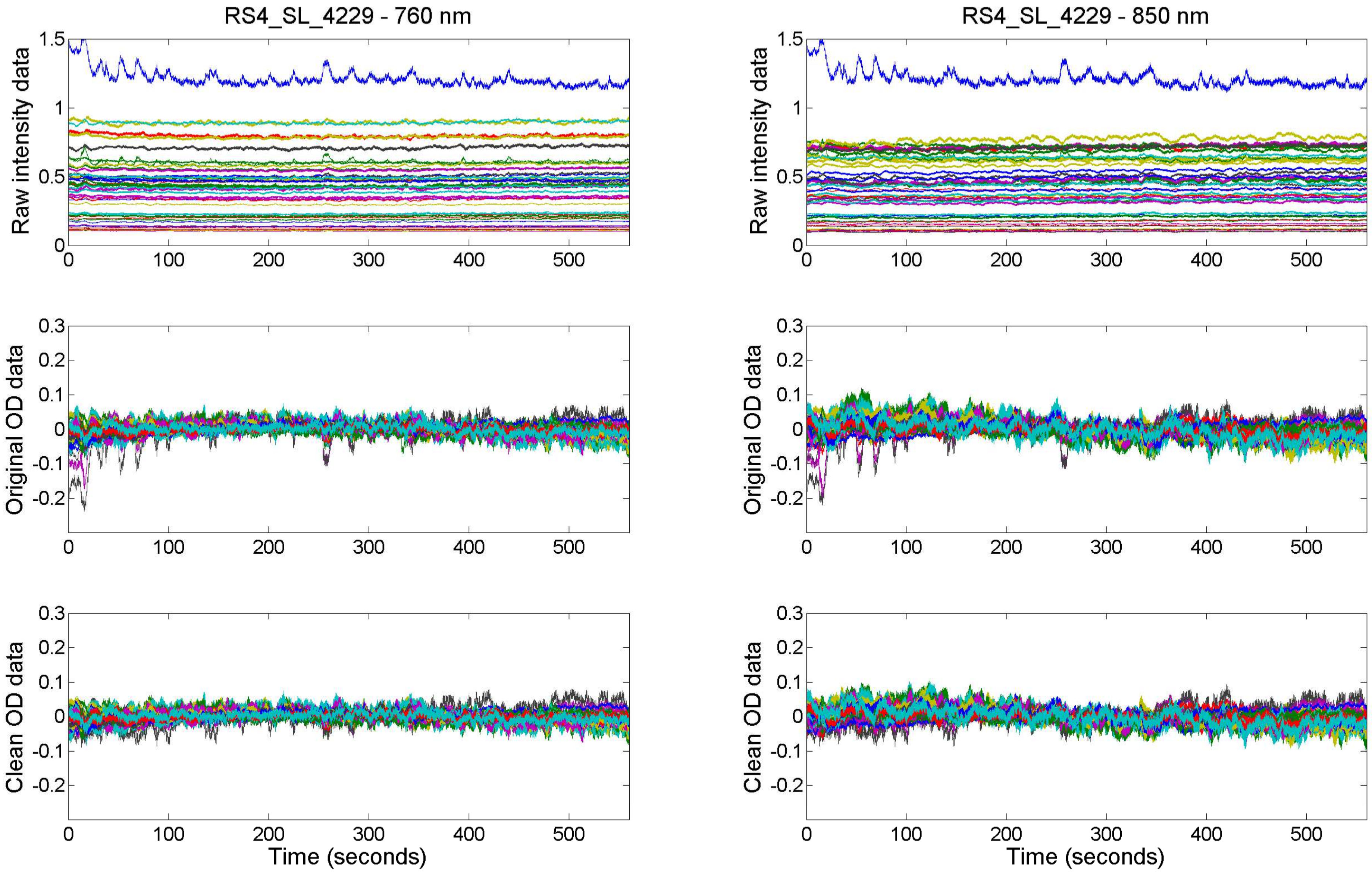
RS4_SL_4229 - 760 nm
RS4_SL_4229 - 850 nm
Raw intensity data
Original OD data
Clean OD data
Time (seconds)
1.5
1
0.5
0
0.3
0.2
0.1
-0.1
-0.2
100
200
300
400
500

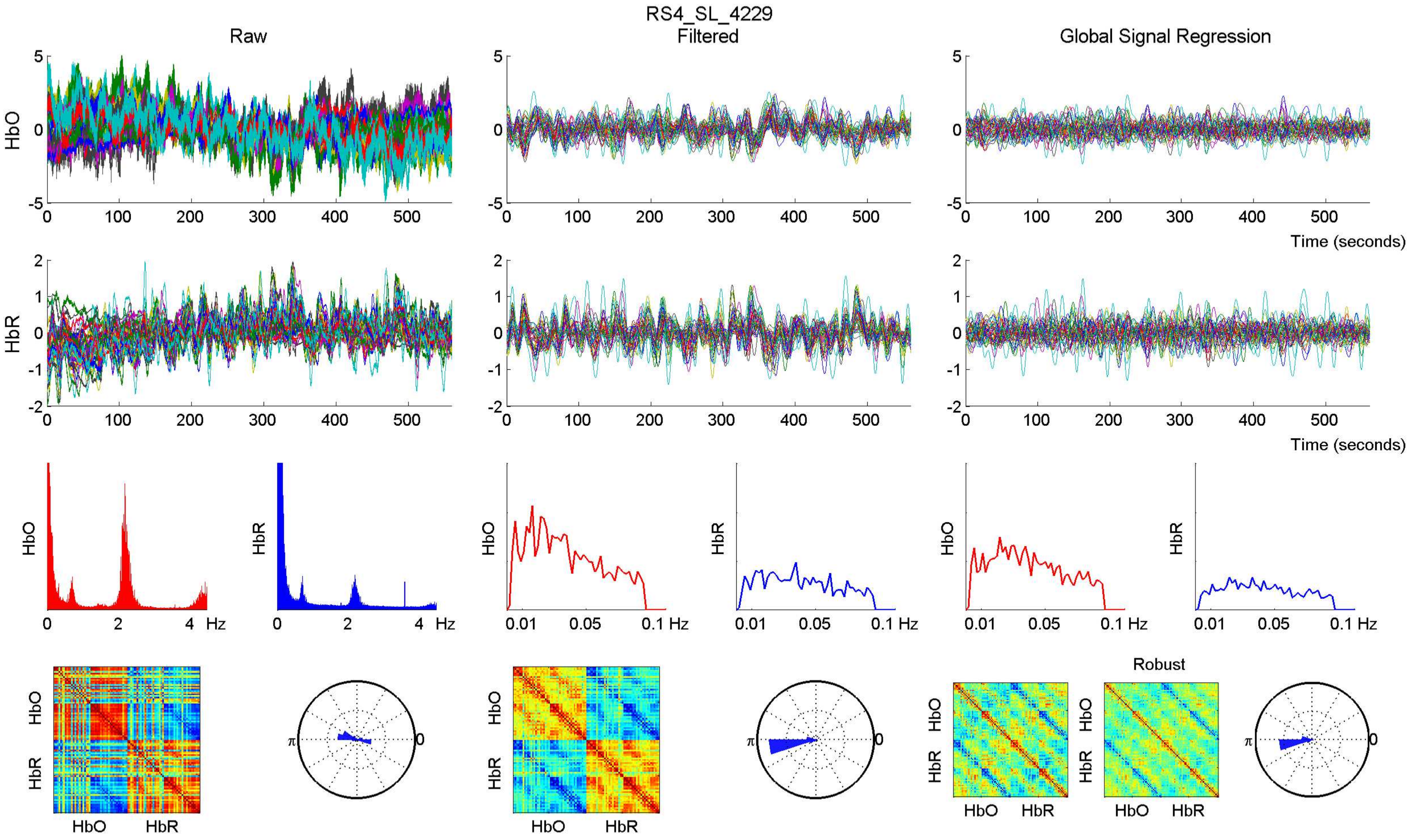
RS4_SL_4229
Raw
Filtered
Global Signal Regression
HbO
HbR
Time (seconds)
Hz
Robust

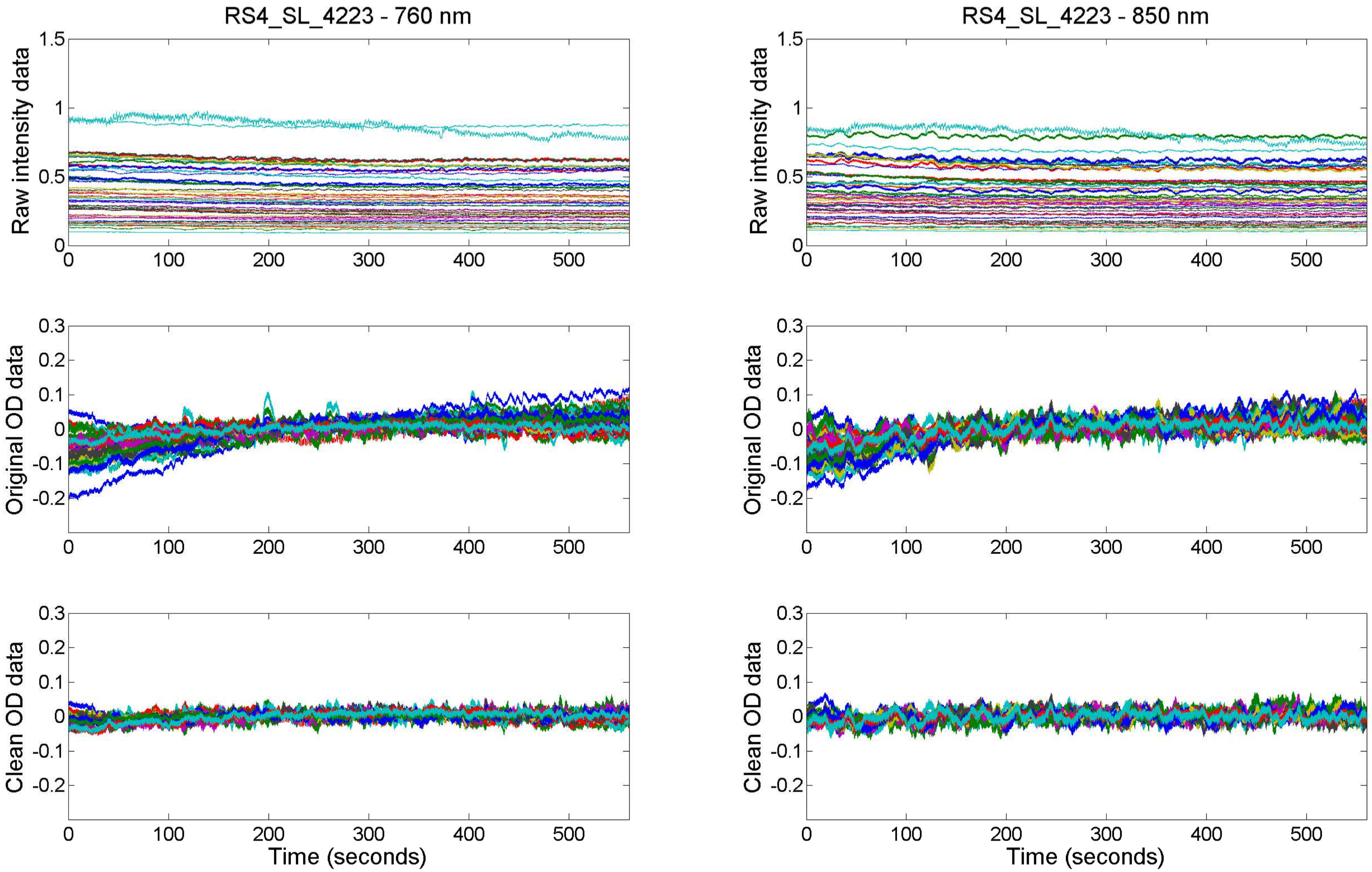
RS4_SL_4223 - 760 nm
RS4_SL_4223 - 850 nm
Raw intensity data
Original OD data
Clean OD data
Time (seconds)

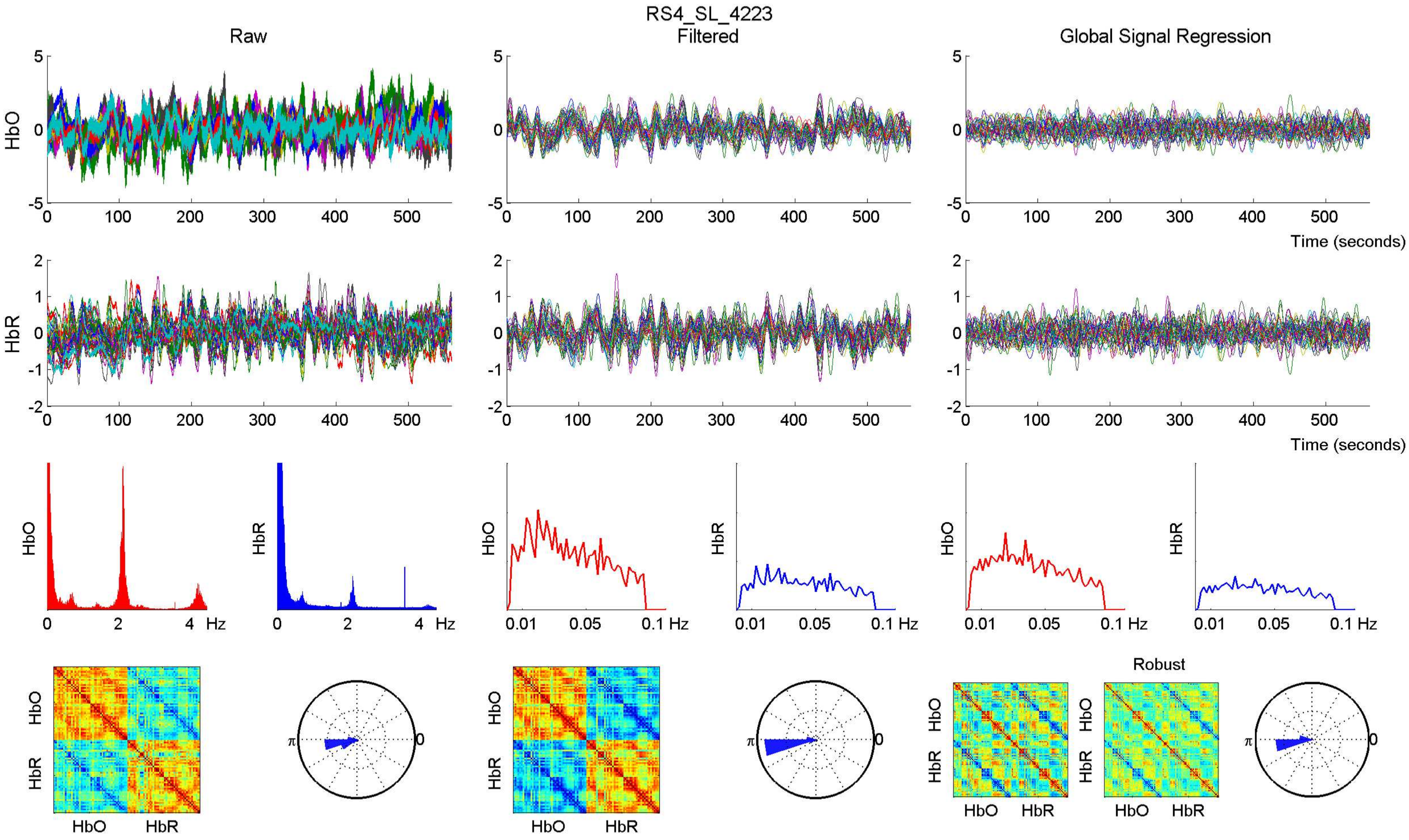
RS4_SL_4223
Raw
Filtered
Global Signal Regression
HbO
HbR
Time (seconds)
Hz
Robust
HbO
HbR

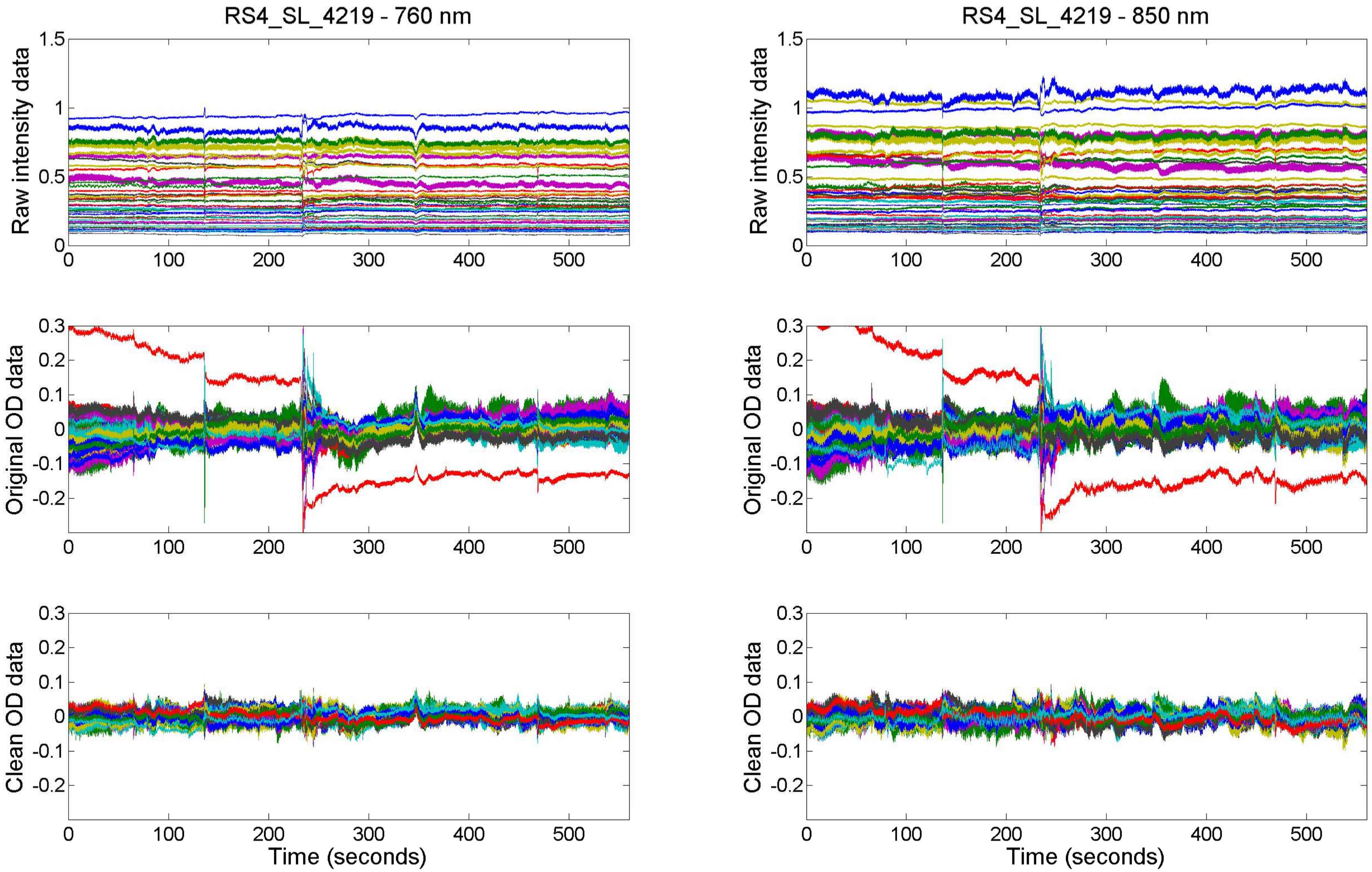

RS4_SL_4219 - 760 nm
RS4_SL_4219 - 850 nm
Raw intensity data
Original OD data
Clean OD data
Time (seconds)

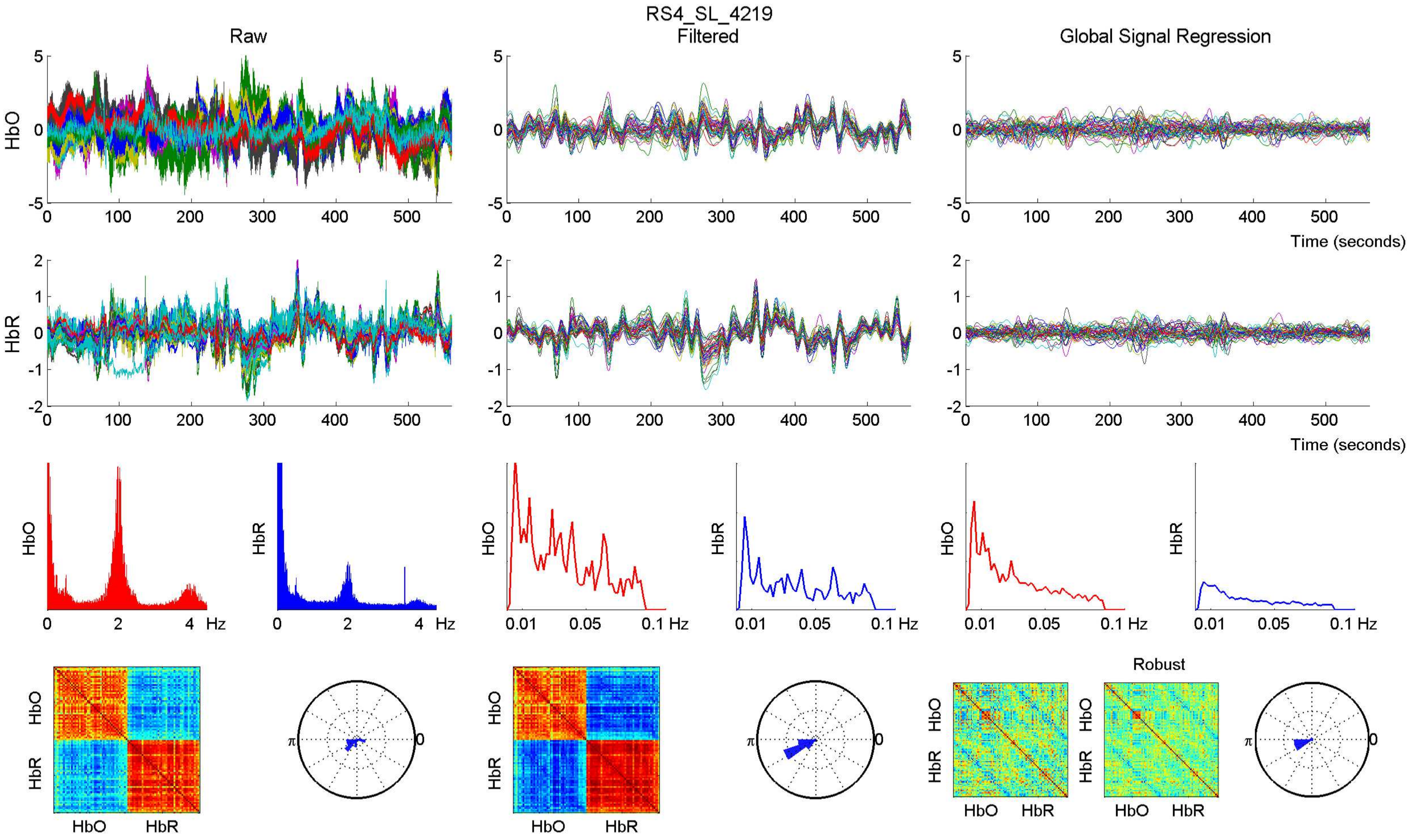

RS4_SL_4219
Raw
Filtered
Global Signal Regression
HbO
HbR
Time (seconds)
Hz
Robust

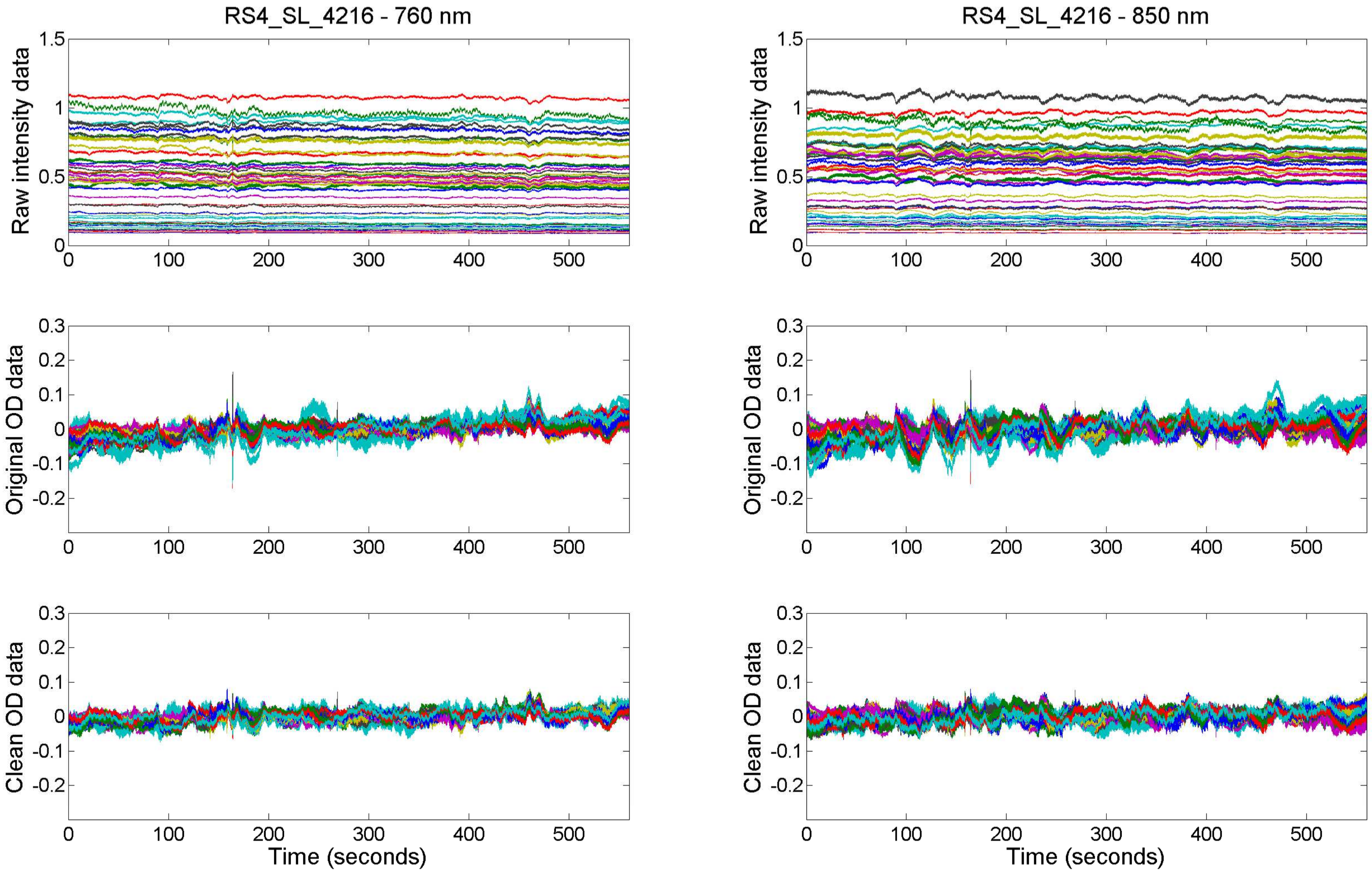

RS4_SL_4216 - 760 nm
RS4_SL_4216 - 850 nm
Raw intensity data
Original OD data
Clean OD data
Time (seconds)
0
100
200
300
400
500
1.5
1
0.5
0.3
0.2
0.1
-0.1
-0.2

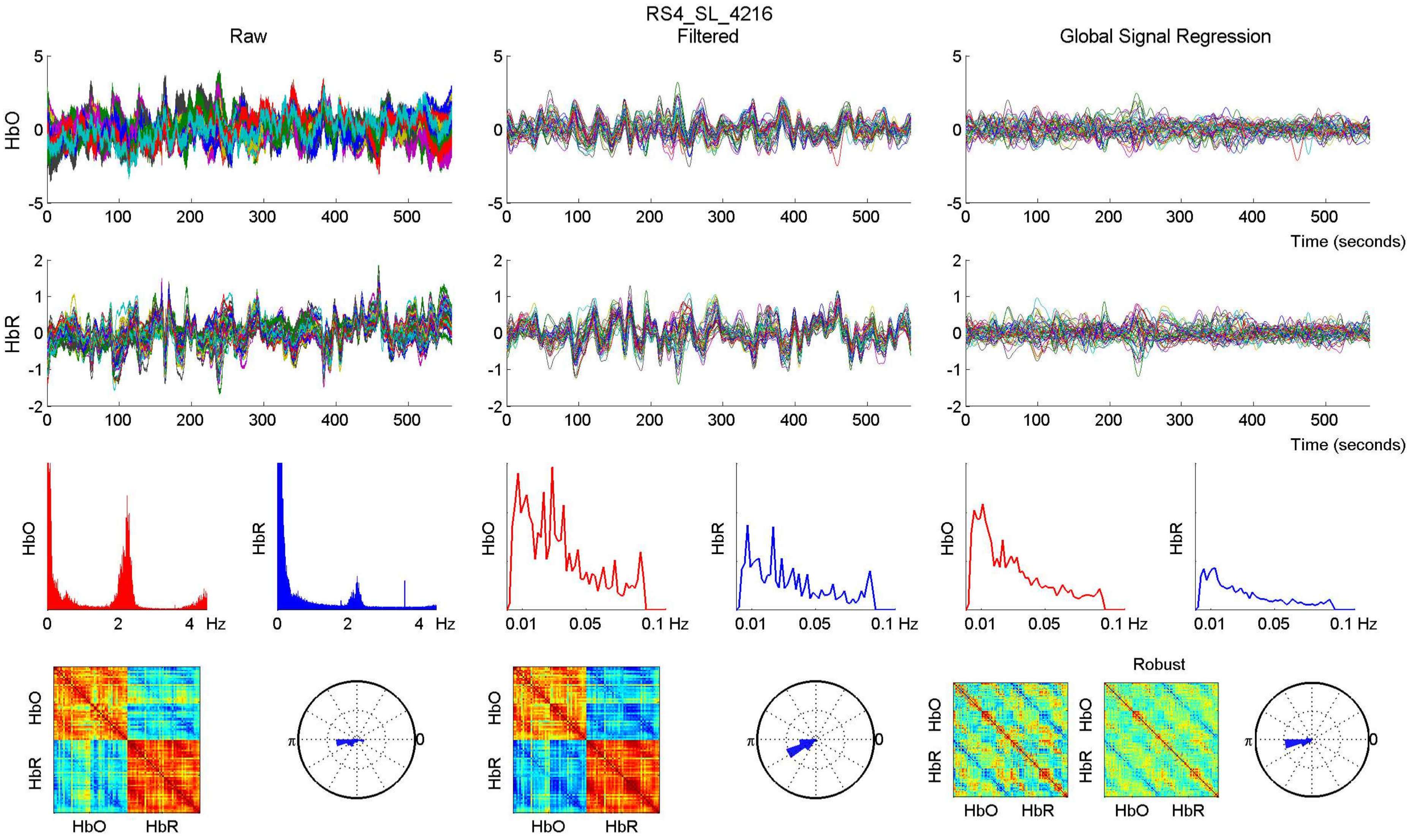
RS4_SL_4216
Raw
Filtered
Global Signal Regression
HbO
HbR
Time (seconds)
Hz
Robust

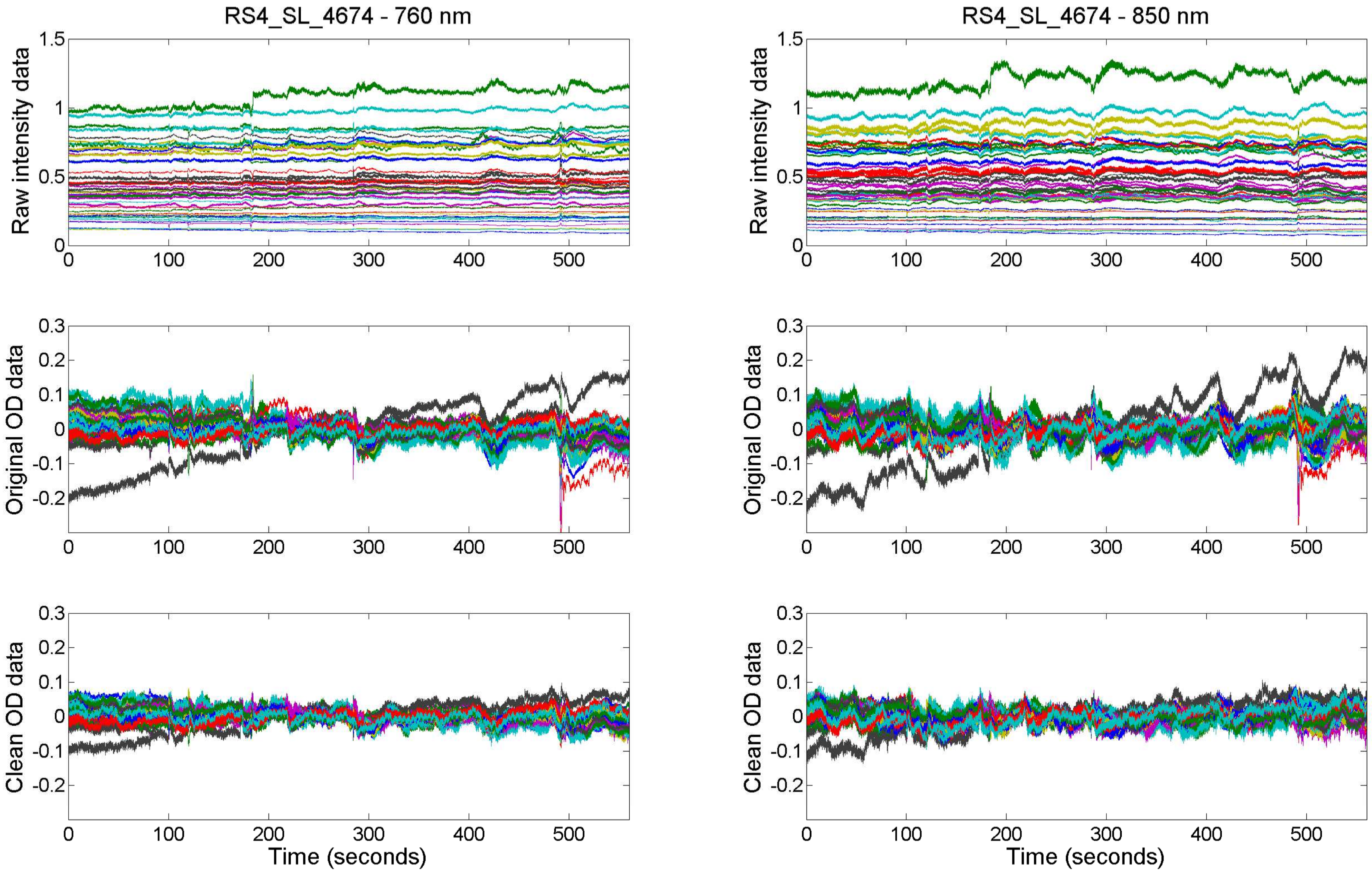
RS4_SL_4674 - 760 nm
RS4_SL_4674 - 850 nm
Raw intensity data
Original OD data
Clean OD data
Time (seconds)
1.5
1
0.5
0
0.3
0.2
0.1
-0.1
-0.2
100
200
300
400
500

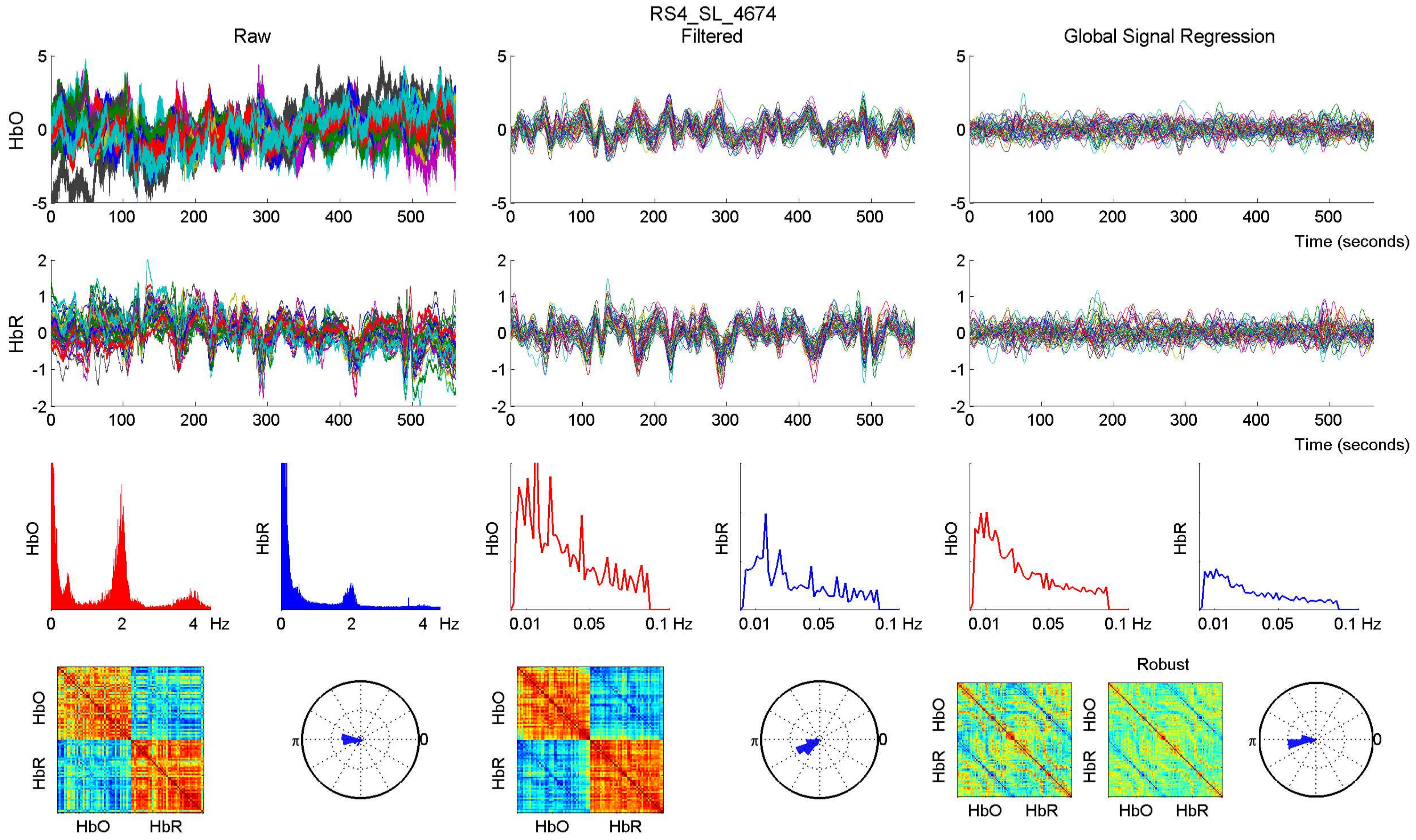
RS4_SL_4674
Raw
Filtered
Global Signal Regression
HbO
HbR
Time (seconds)
Hz
Robust

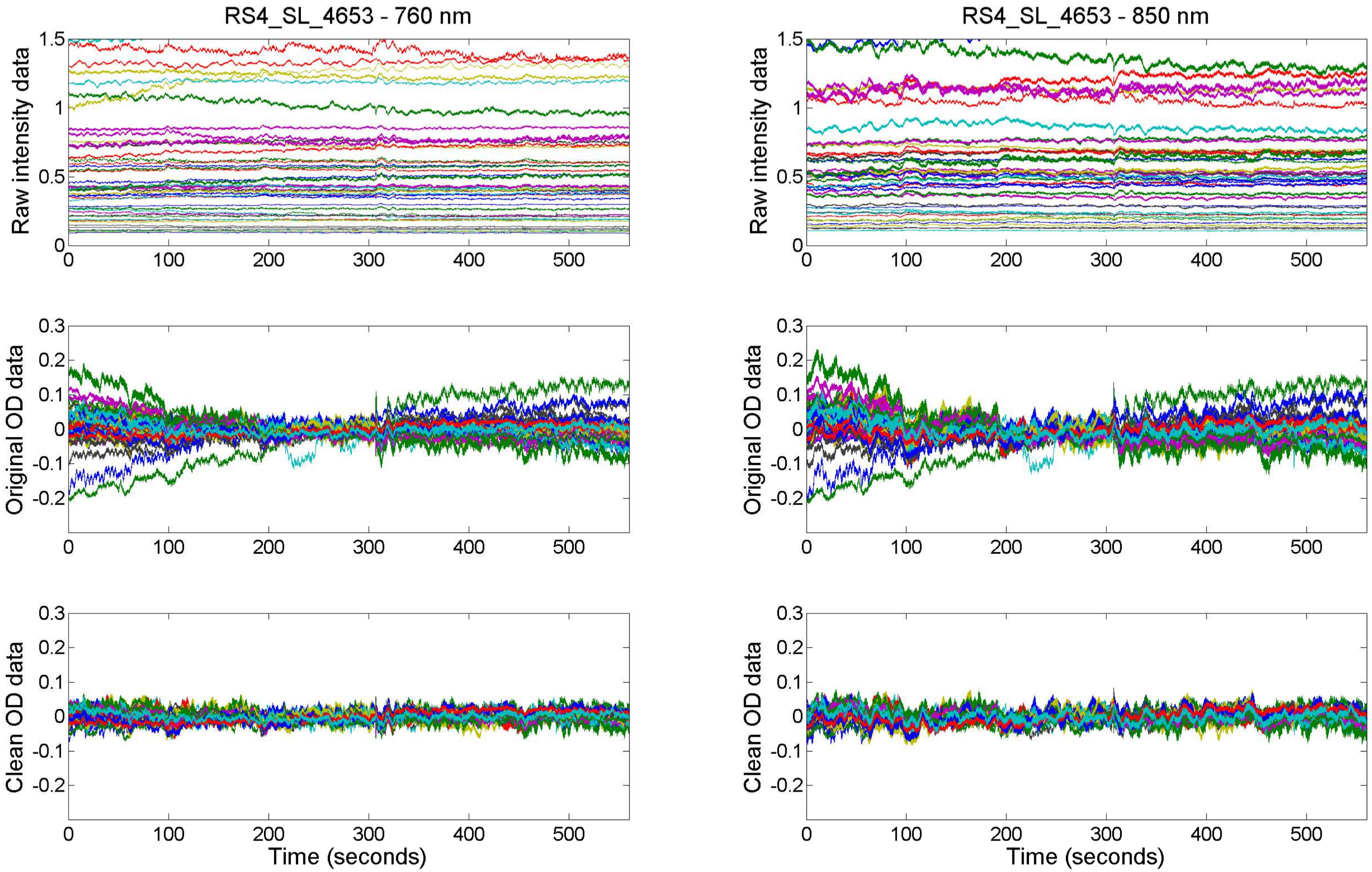

RS4_SL_4653 - 760 nm
RS4_SL_4653 - 850 nm
Raw intensity data
Original OD data
Clean OD data
Time (seconds)

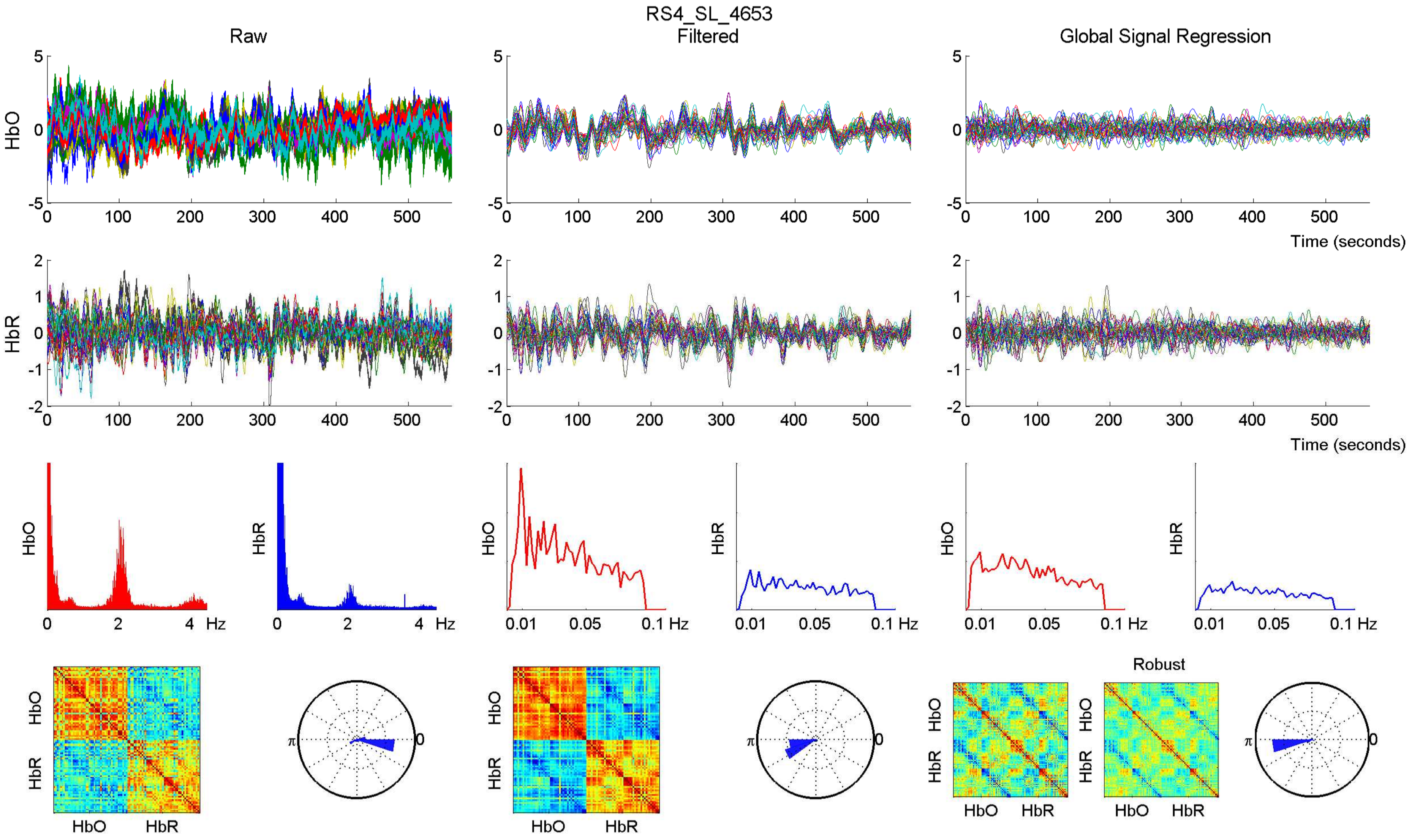

RS4_SL_4653
Raw
Filtered
Global Signal Regression
HbO
HbR
Time (seconds)
Hz
Robust

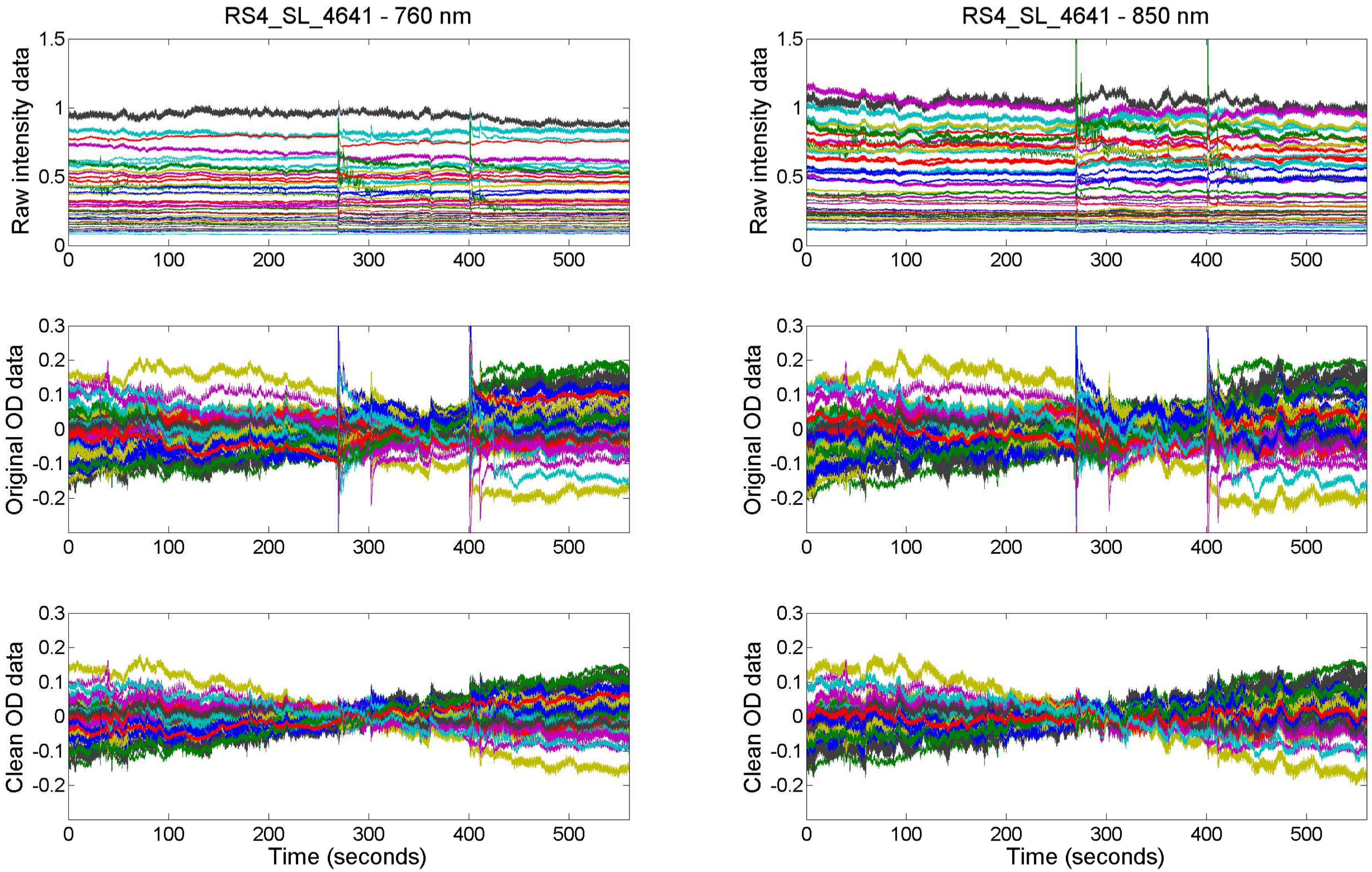

RS4_SL_4641 - 760 nm
RS4_SL_4641 - 850 nm
Raw intensity data
Original OD data
Clean OD data
Time (seconds)

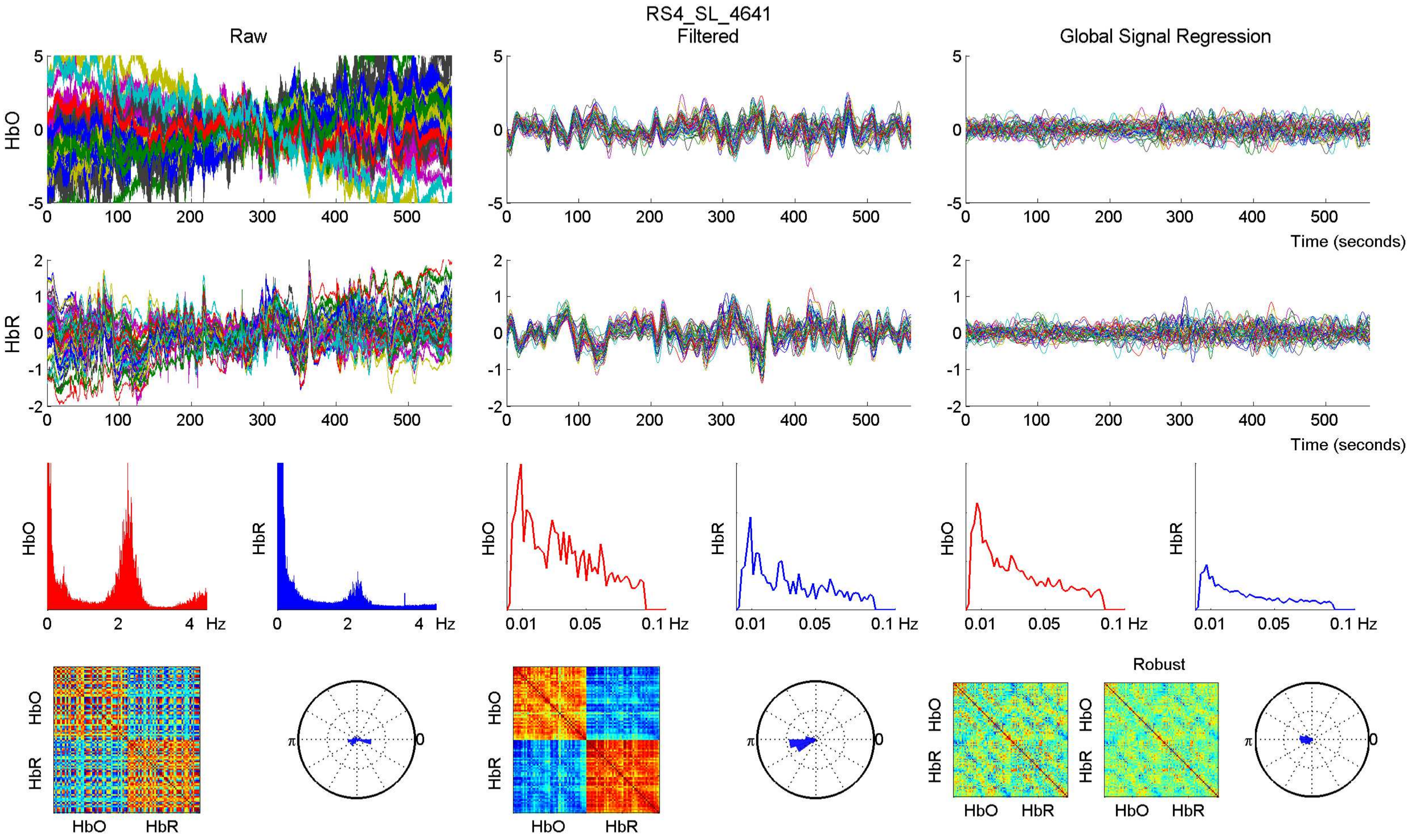
RS4_SL_4641
Raw
Filtered
Global Signal Regression
HbO
HbR
Time (seconds)
Hz
Robust
π
0

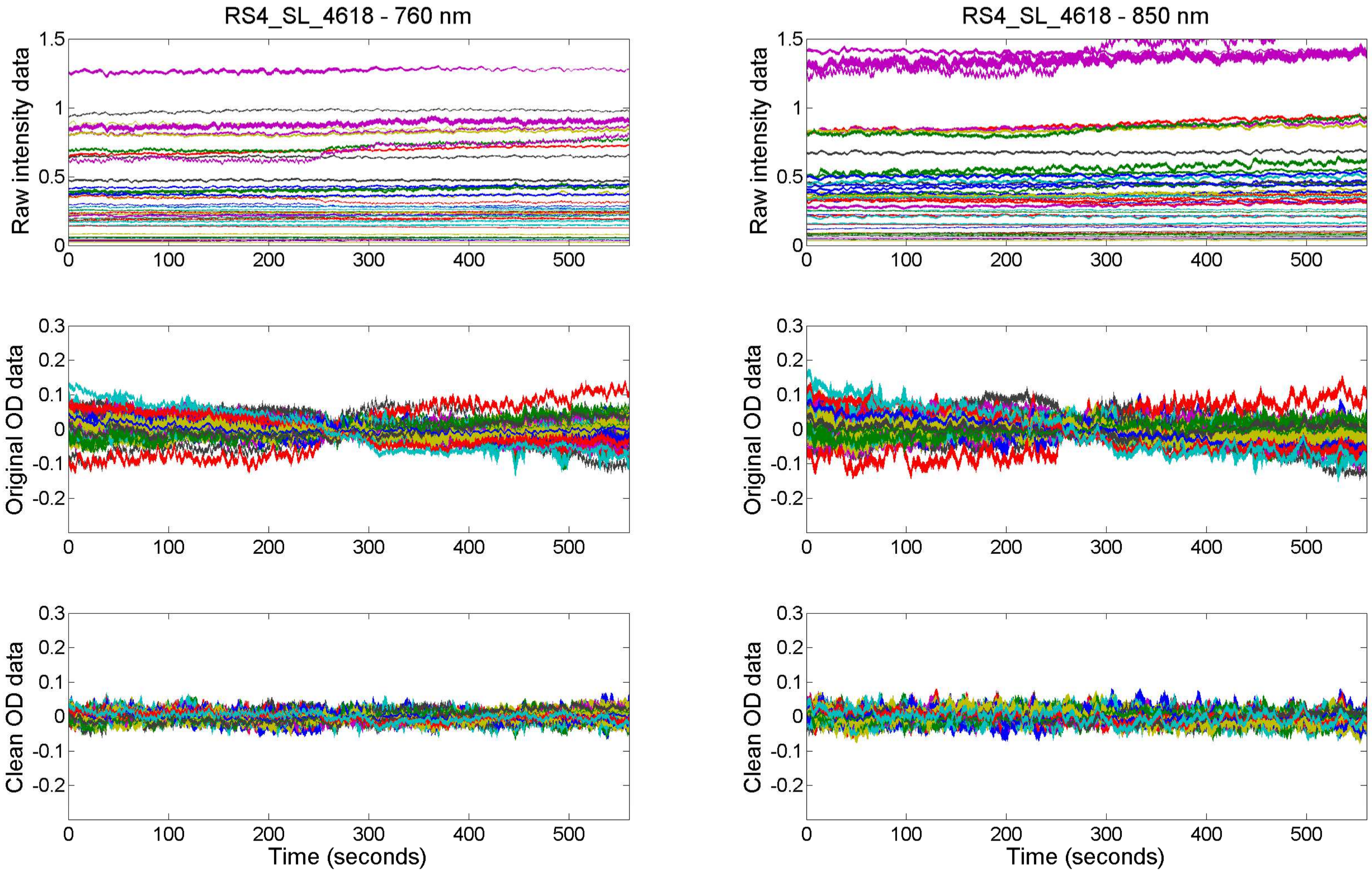

RS4_SL_4618 - 760 nm
RS4_SL_4618 - 850 nm
Raw intensity data
Original OD data
Clean OD data
Time (seconds)

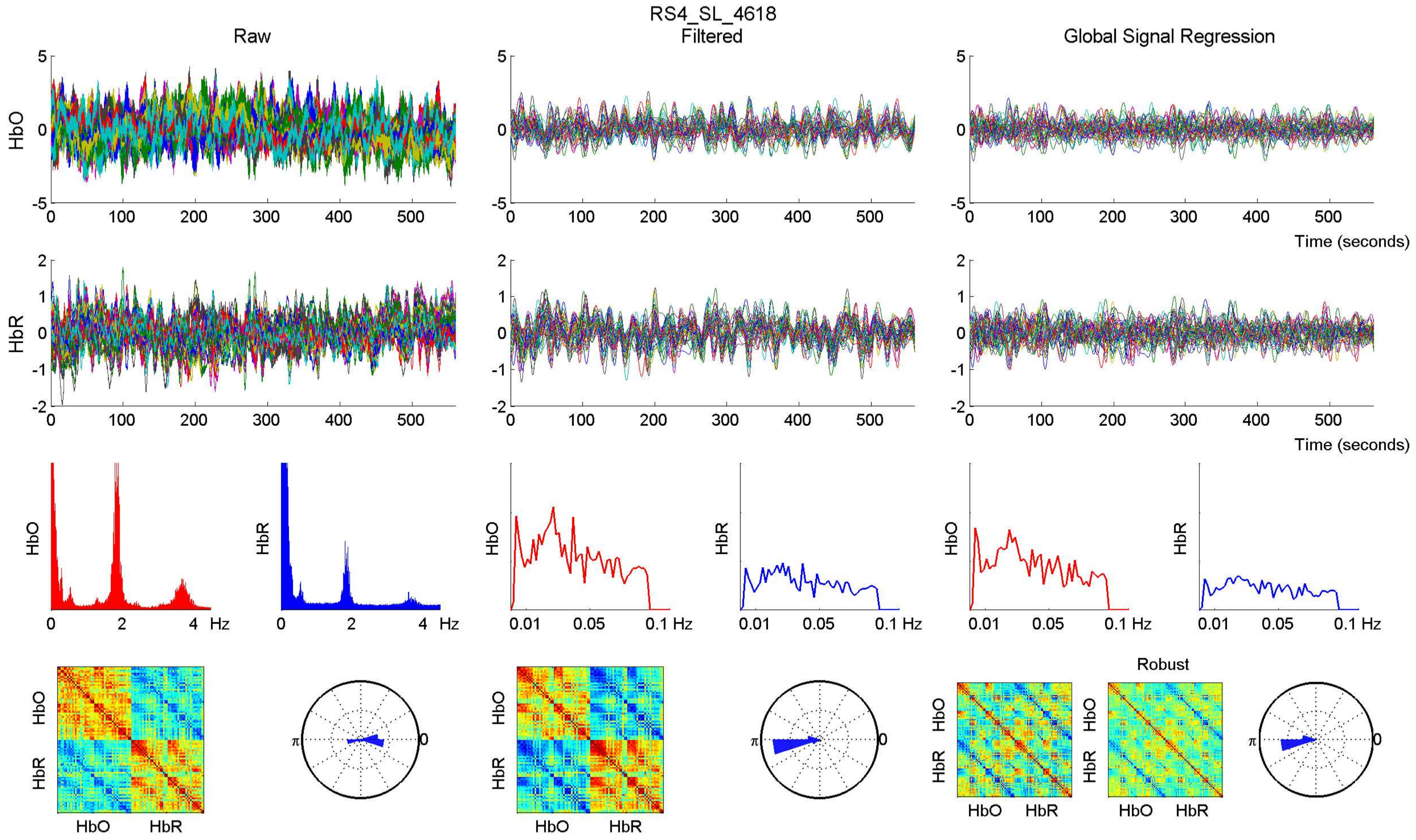
RS4_SL_4618
Raw
Filtered
Global Signal Regression
HbO
HbR
Time (seconds)
Hz
Robust

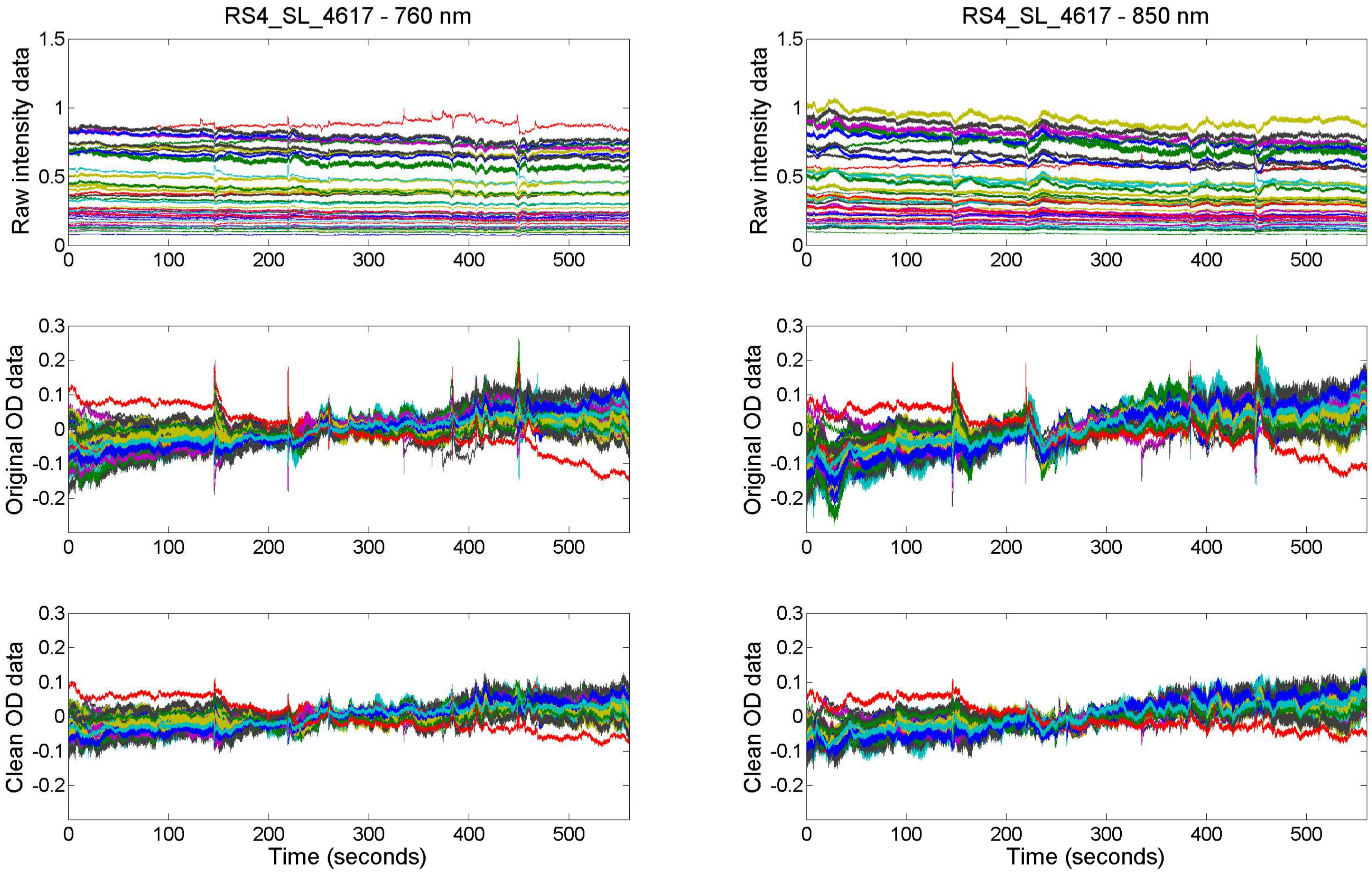
RS4_SL_4617 - 760 nm
RS4_SL_4617 - 850 nm
Raw intensity data
Original OD data
Clean OD data
Time (seconds)
1.5
1
0.5
0
0.3
0.2
0.1
-0.1
-0.2
100
200
300
400
500

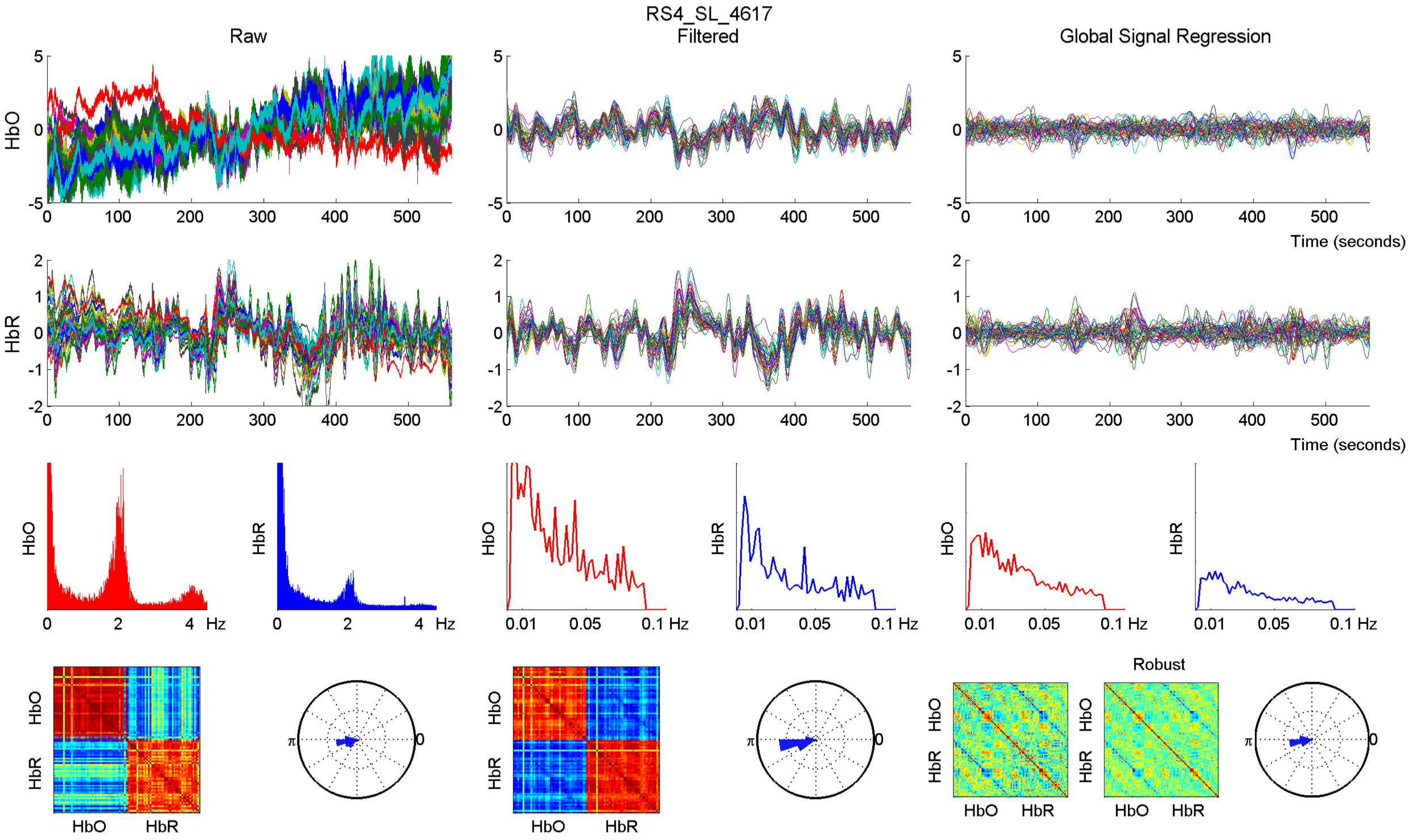
RS4_SL_4617
Raw
Filtered
Global Signal Regression
HbO
HbR
Time (seconds)
Hz
Robust
HbO
HbR

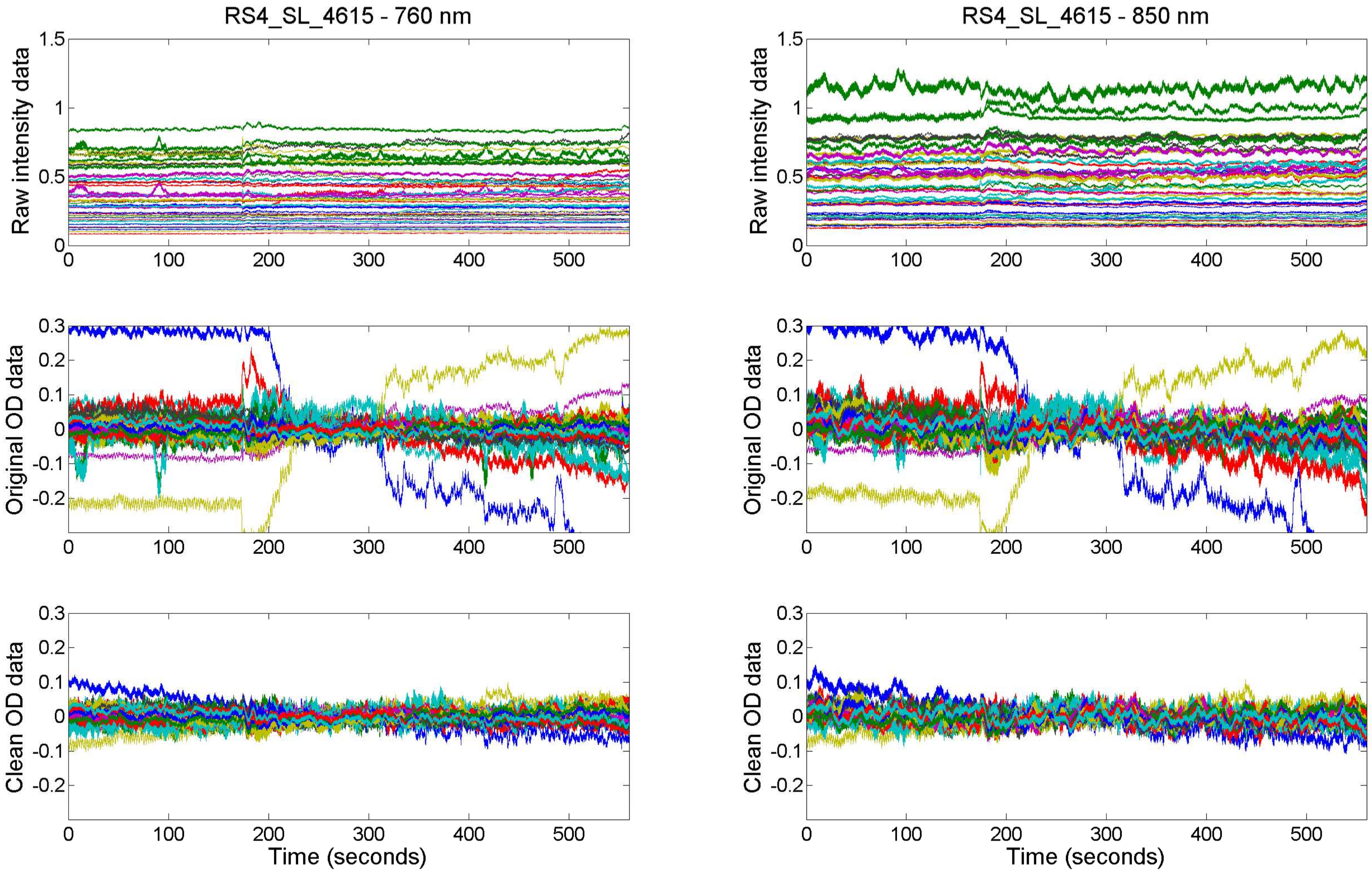
RS4_SL_4615 - 760 nm
RS4_SL_4615 - 850 nm
Raw intensity data
Original OD data
Clean OD data
Time (seconds)
0
100
200
300
400
500
1.5
1
0.5
0.3
0.2
0.1
-0.1
-0.2

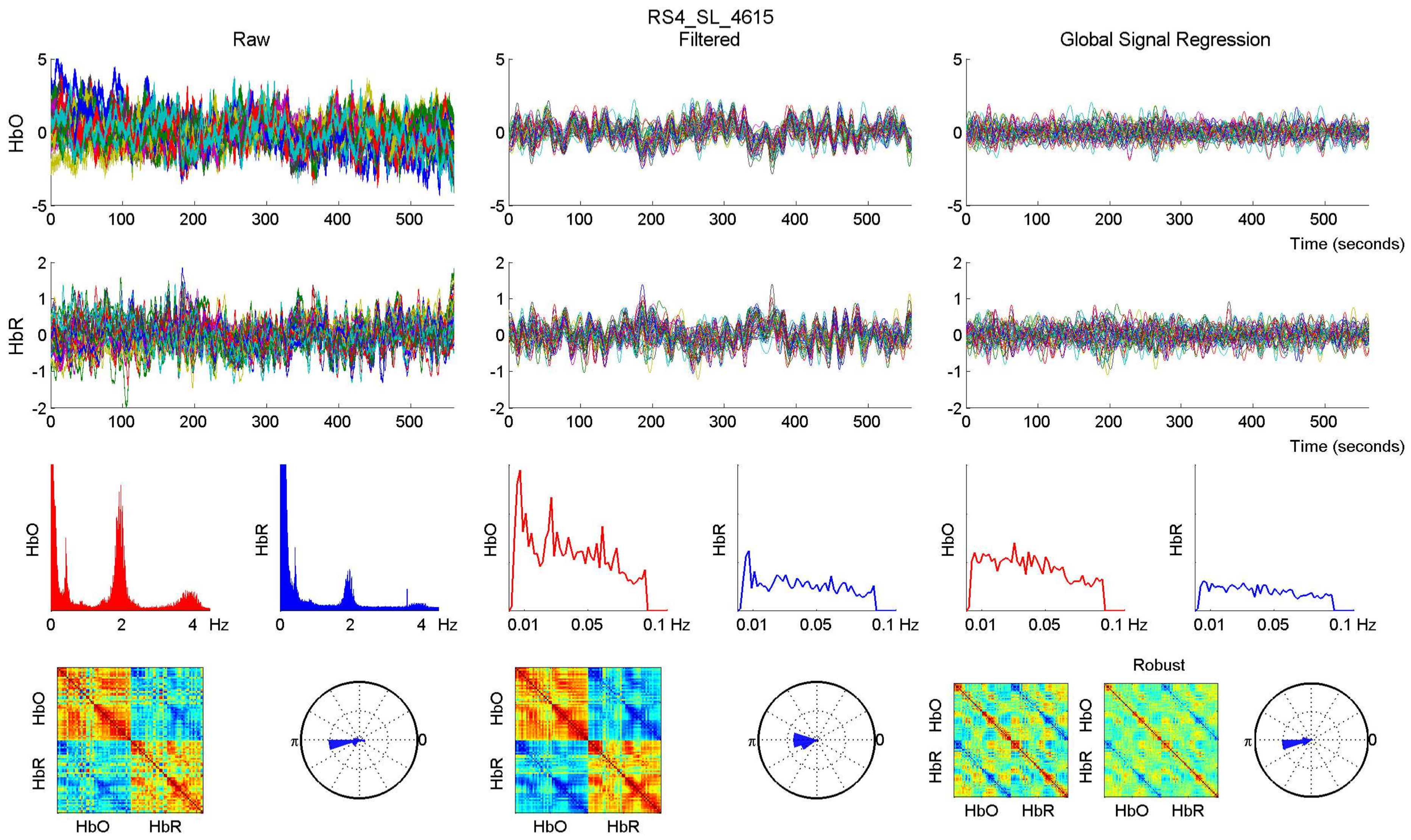
RS4_SL_4615
Raw
Filtered
Global Signal Regression
HbO
HbR
Time (seconds)
Hz
Robust

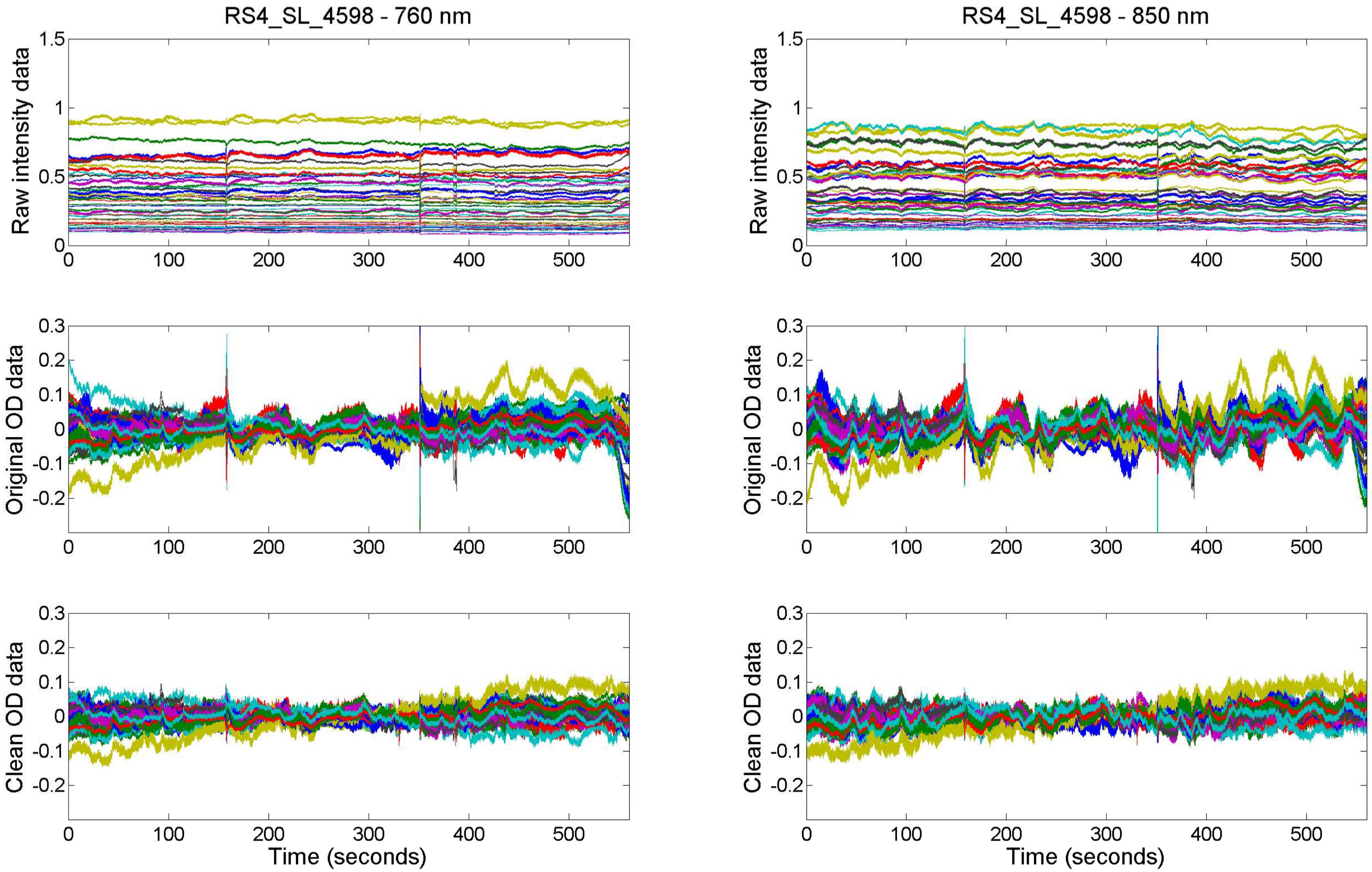
RS4_SL_4598 - 760 nm
RS4_SL_4598 - 850 nm
Raw intensity data
Original OD data
Clean OD data
Time (seconds)

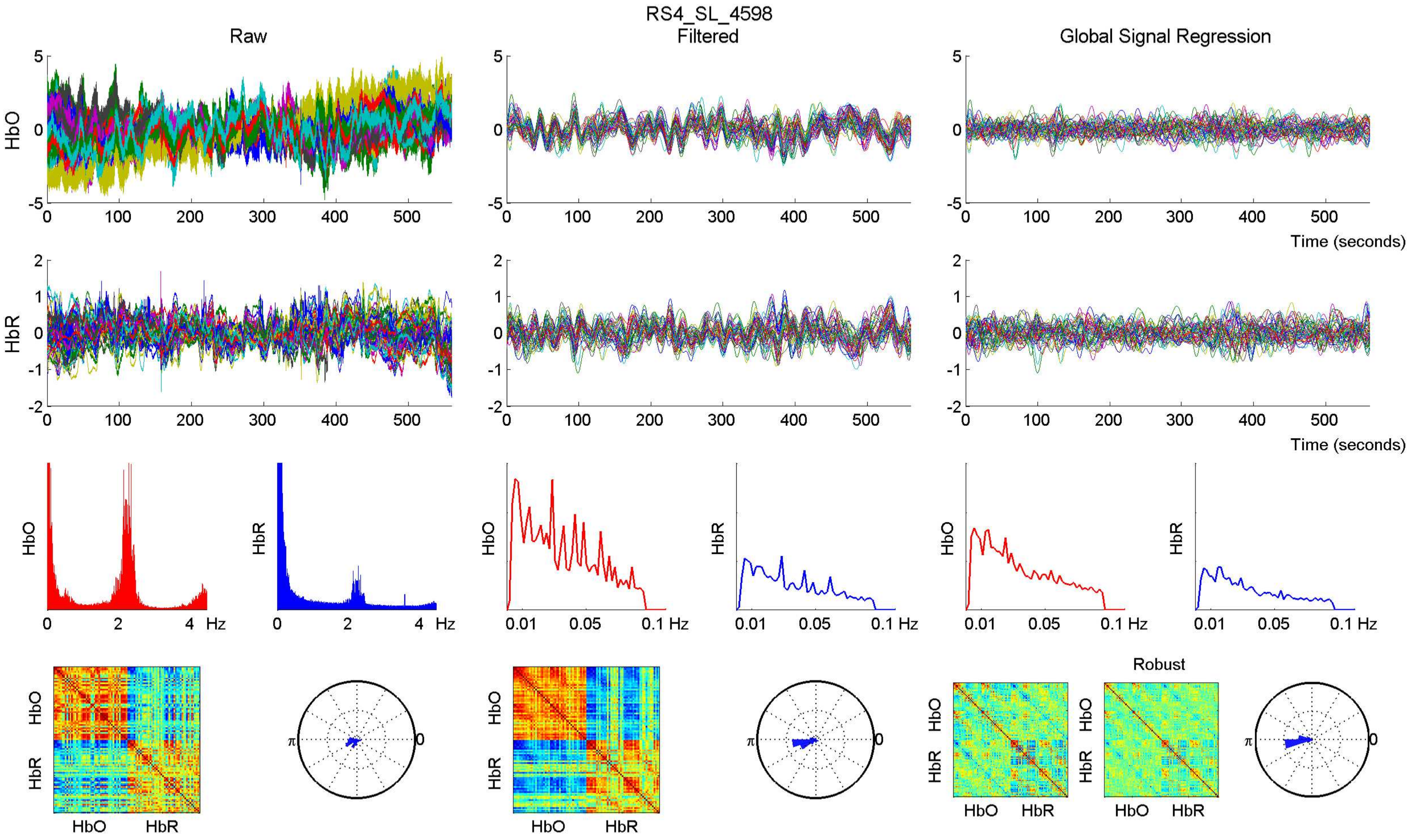
RS4_SL_4598
Raw
Filtered
Global Signal Regression
HbO
HbR
Time (seconds)
Hz
0.01
0.05
0.1 Hz
HbO
HbR
Robust
π
0

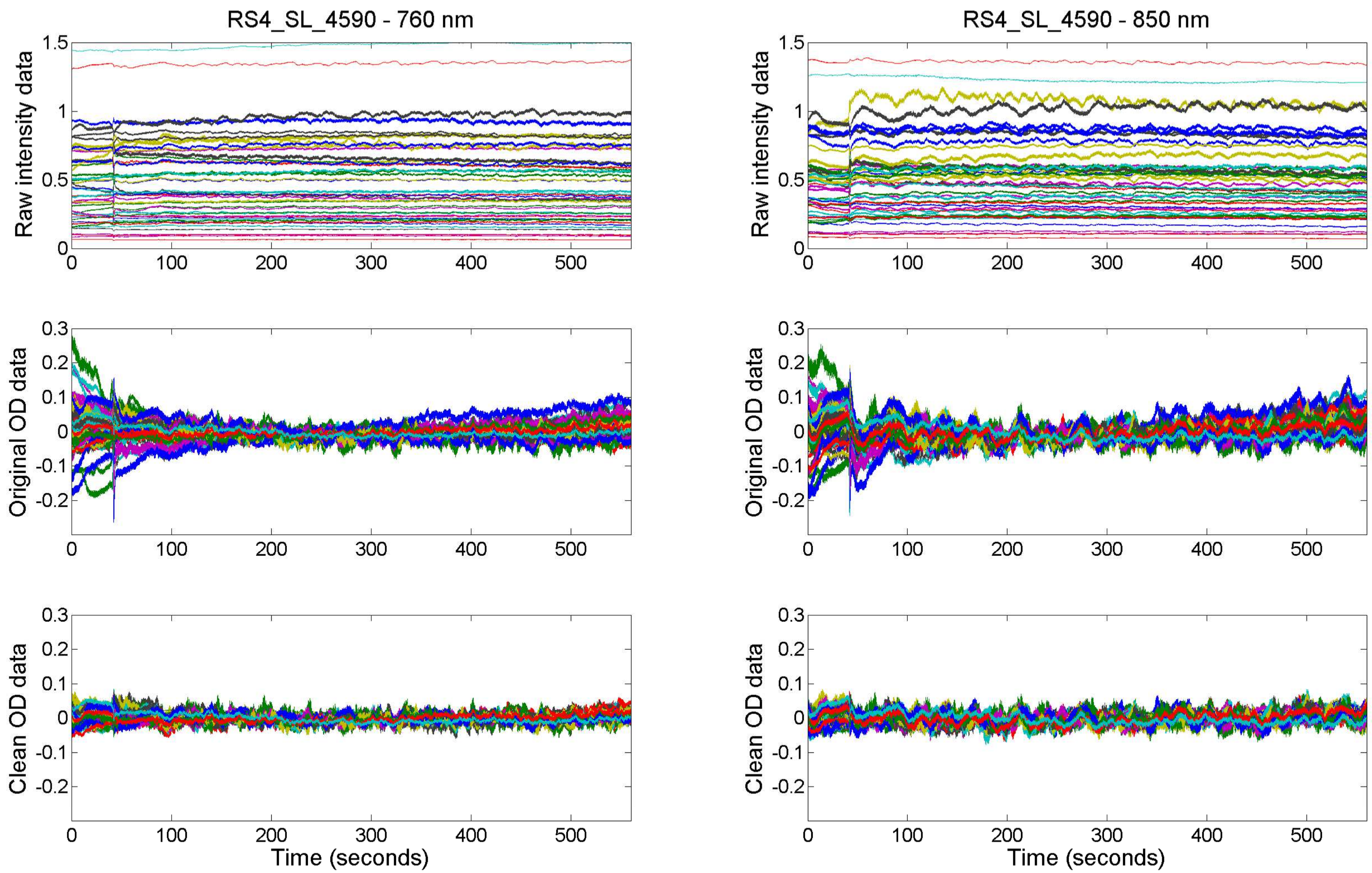

RS4_SL_4590 - 760 nm
RS4_SL_4590 - 850 nm
Raw intensity data
Original OD data
Clean OD data
Time (seconds)

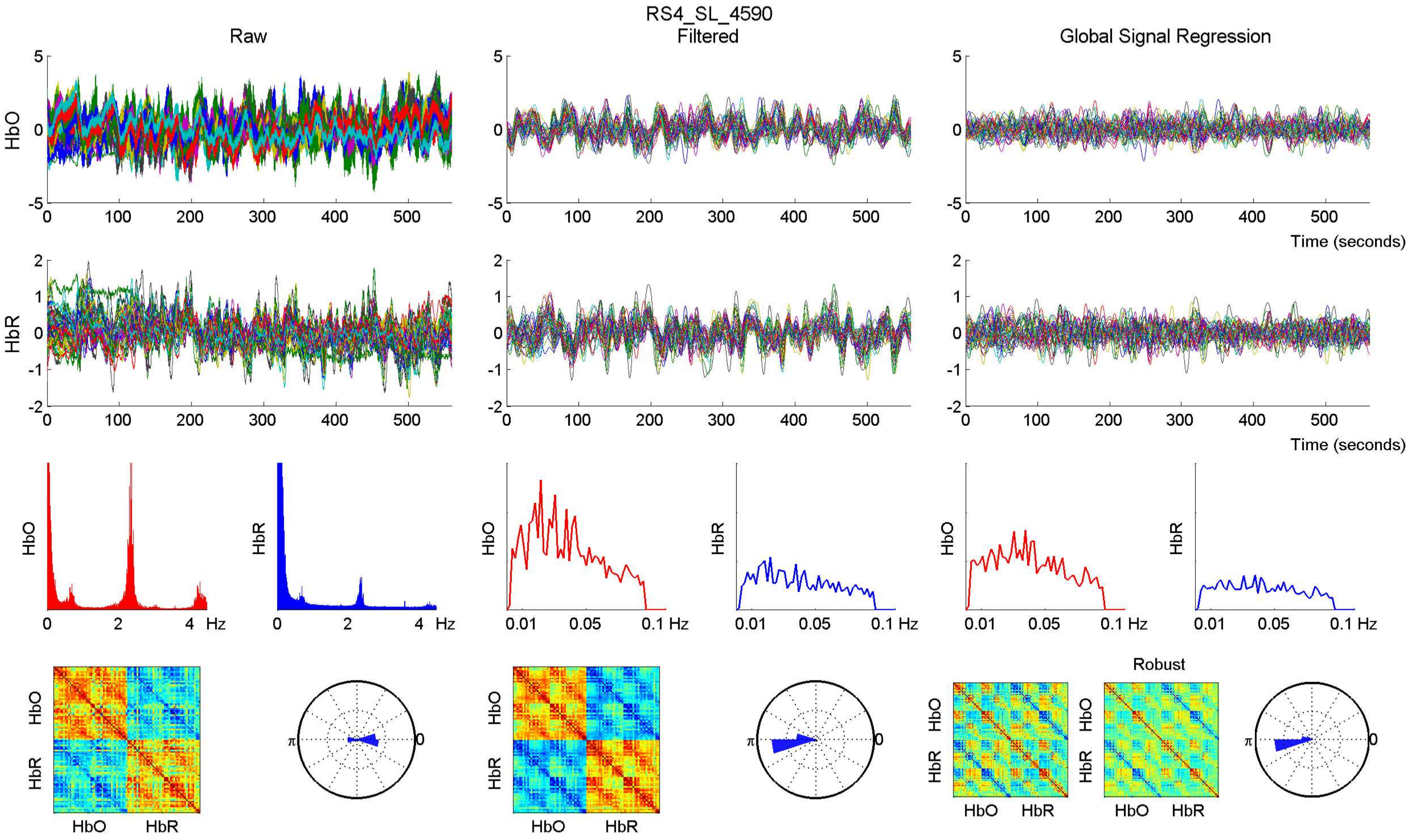

RS4_SL_4590
Raw
Filtered
Global Signal Regression
HbO
HbR
Time (seconds)
Hz
Robust
HbO
HbR

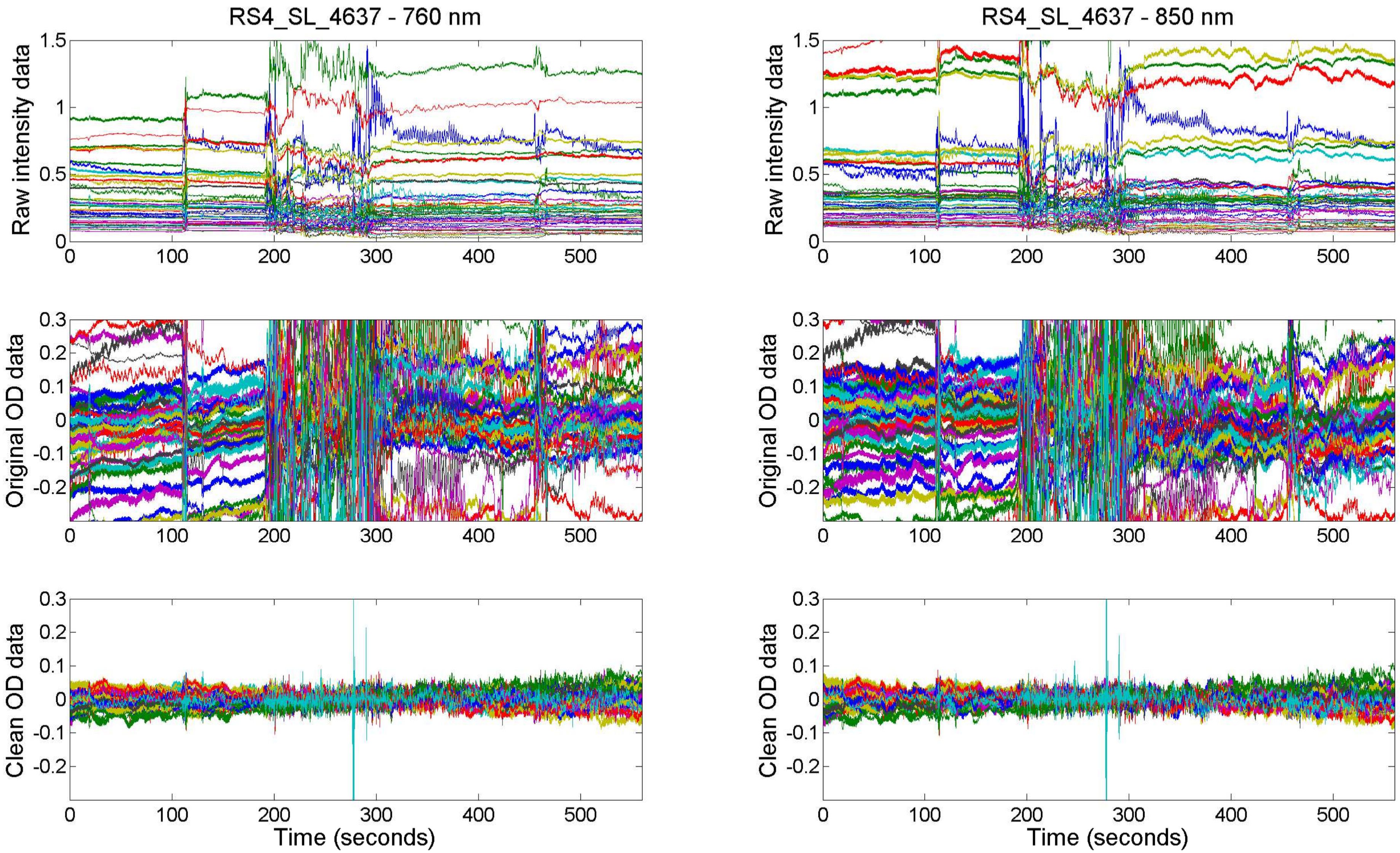

RS4_SL_4637 - 760 nm
RS4_SL_4637 - 850 nm
Raw intensity data
Original OD data
Clean OD data
Time (seconds)

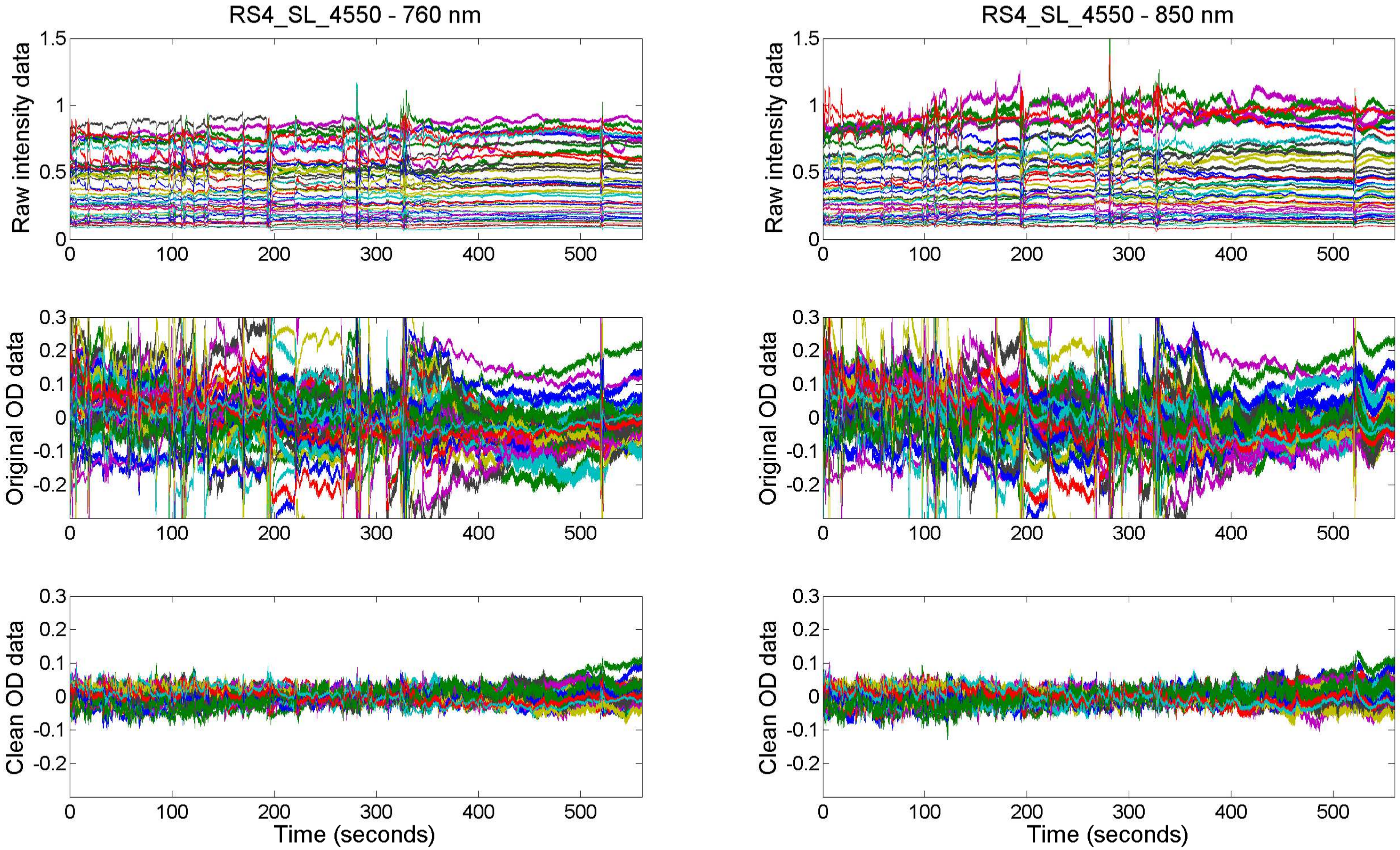
RS4_SL_4550 - 760 nm
RS4_SL_4550 - 850 nm
Raw intensity data
Original OD data
Clean OD data
Time (seconds)
0
100
200
300
400
500
1.5
1
0.5
0.3
0.2
0.1
-0.1
-0.2

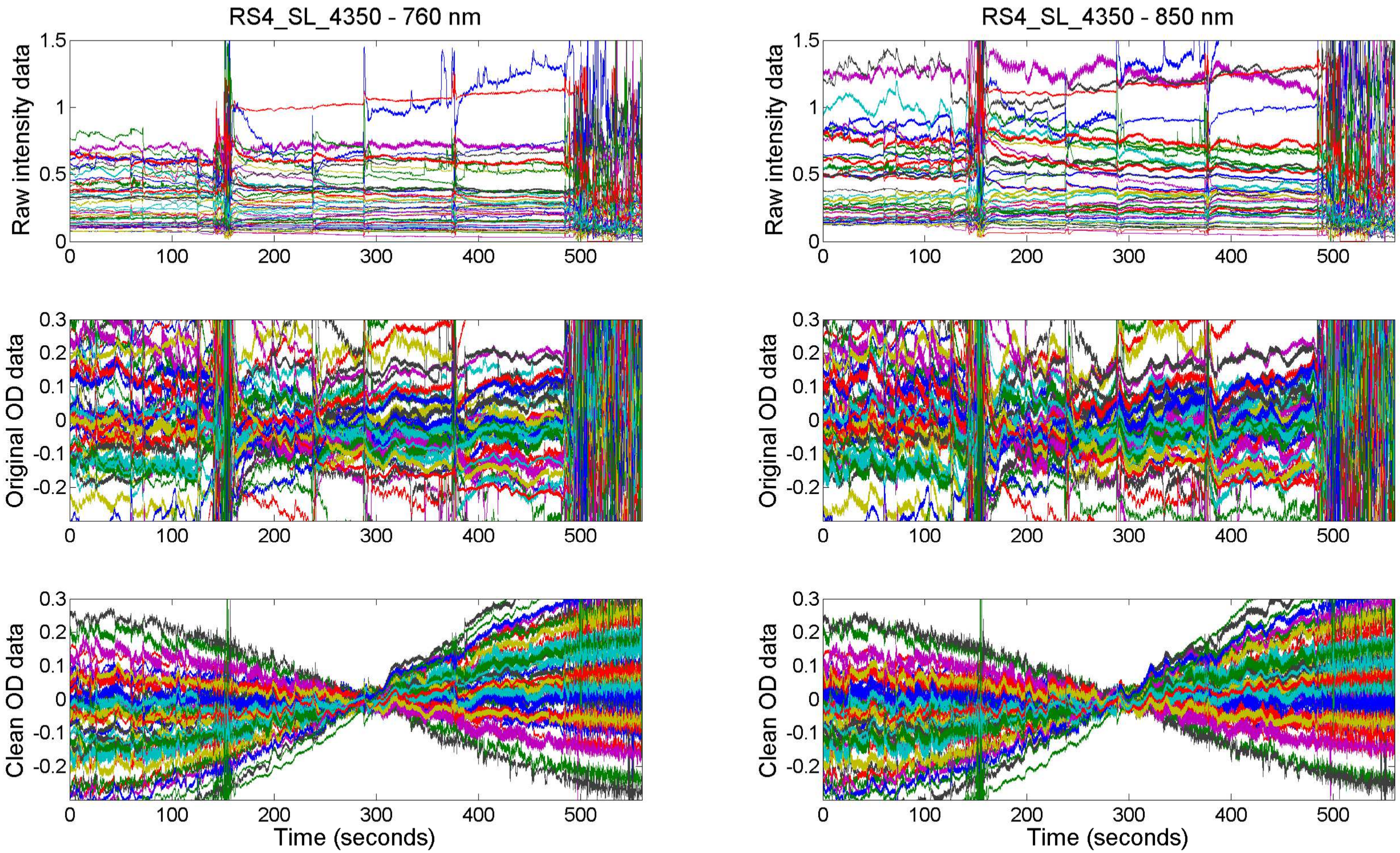
RS4_SL_4350 - 760 nm
RS4_SL_4350 - 850 nm
Raw intensity data
Original OD data
Clean OD data
Time (seconds)

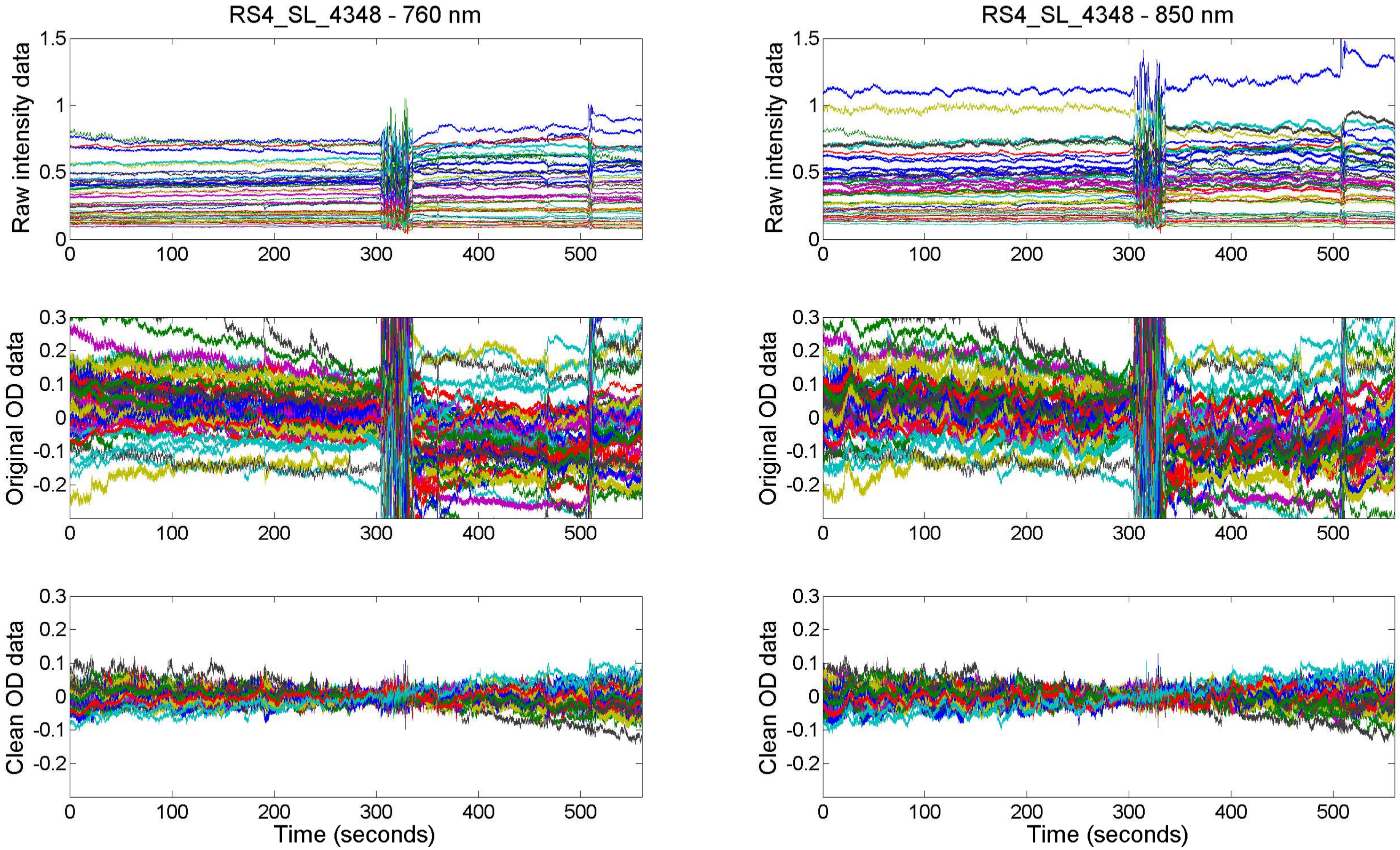
RS4_SL_4348 - 760 nm
RS4_SL_4348 - 850 nm
Raw intensity data
Original OD data
Clean OD data
Time (seconds)
0
100
200
300
400
500
1.5
1
0.5
0.3
0.2
0.1
-0.1
-0.2

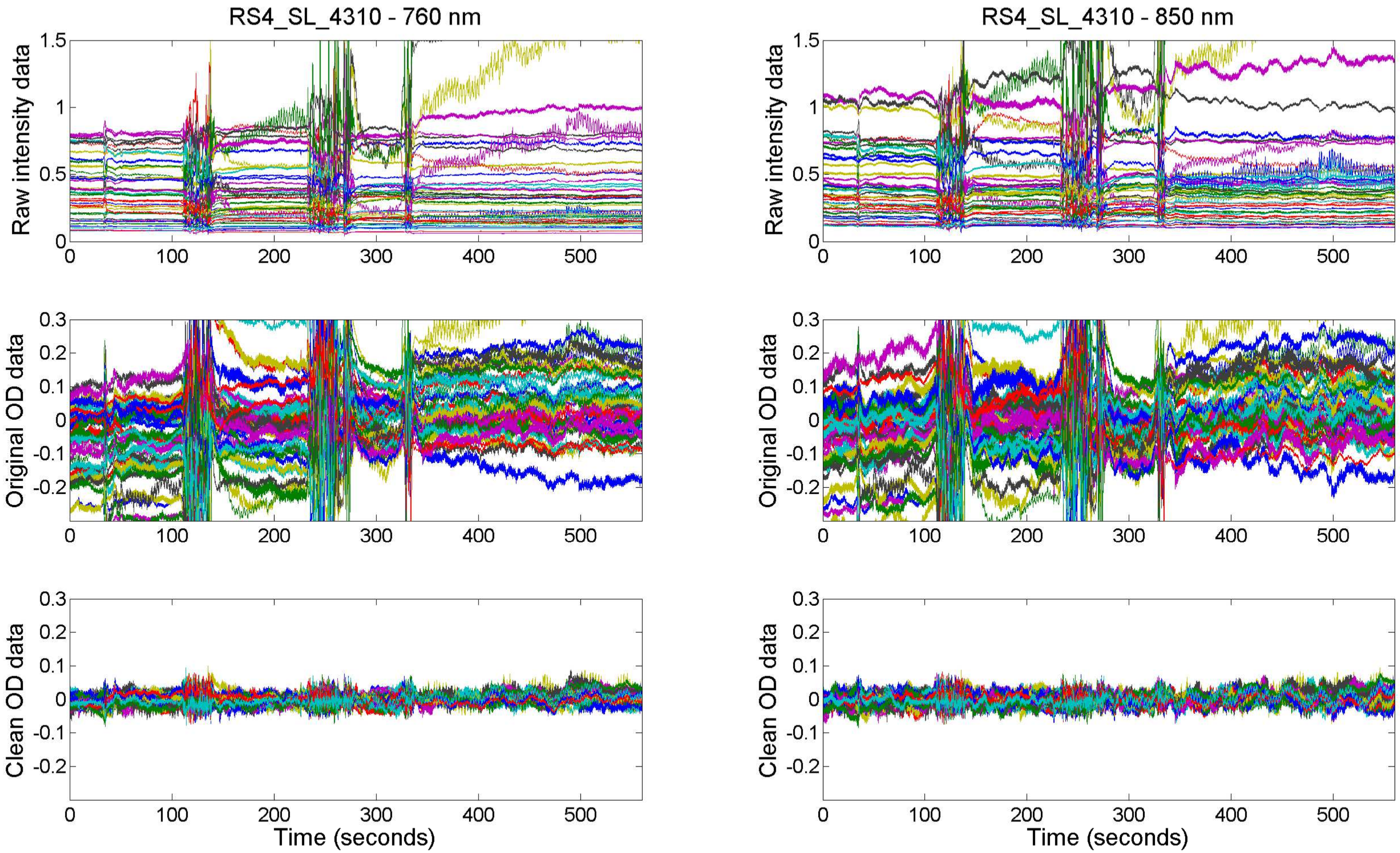
RS4_SL_4310 - 760 nm
RS4_SL_4310 - 850 nm
Raw intensity data
Original OD data
Clean OD data
Time (seconds)
0
100
200
300
400
500
1.5
1
0.5
0.3
0.2
0.1
-0.1
-0.2
